# Supplementary material for: Elevated nuclear TDP-43 induces constitutive exon skipping
Source: Mol Neurodegener. 2024 Jun 9;19:45. doi: 10.1186/s13024-024-00732-w (PMC11163724; doi:10.1186/s13024-024-00732-w)
Supplement: Supplementary file 1 — Supplementary Material 1 [file 13024_2024_732_MOESM1_ESM.pdf]

SUPPLEMENTARY FIGURES

Title: Elevated nuclear TDP-43 induces constitutive exon skipping

Authors: Rogger P. Carmen-Orozco[1,2,*], William Tsao[1,2,*], Yingzhi Ye[3,*], Irika R. Sinha[1,2], Koping Chang[1], Vickie Trinh[1,2], William Chung[1], Kyra Bowden[1], Juan C. Troncoso[1], Seth Blackshaw[2,4,5], Lindsey R. Hayes[5], Shuying Sun[3], Philip C. Wong[1,2], Jonathan P. Ling[1,#]

Affiliations:

Departments of [1]Pathology, [2]Neuroscience, [3]Physiology, [4]Ophthalmology and [5]Neurology, Johns Hopkins School of Medicine, Baltimore, MD 21205

* indicates equal contribution

[#] indicates corresponding author

Correspondence to Jonathan Ling (jling@jhu.edu)

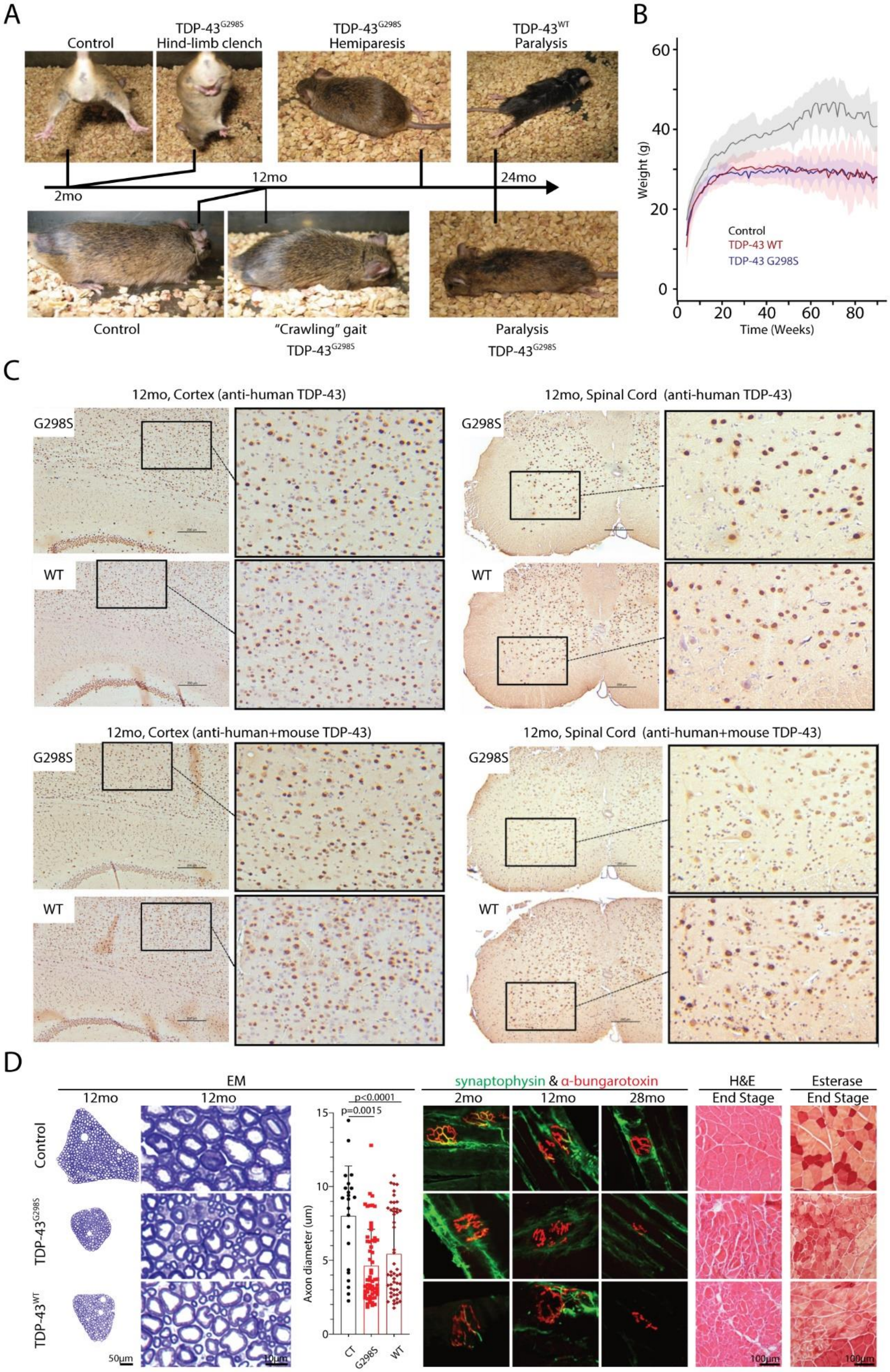

A
Control
TDP-43G298S Hind-limb clench
TDP-43G298S Hemiparesis
TDP-43WT Paralysis
2mo
12mo
24mo
Control
"Crawling" gait TDP-43G298S
Paralysis TDP-43G298S
B
Weight (g)
Time (Weeks)
Control
TDP-43 WT
TDP-43 G298S
C
12mo, Cortex (anti-human TDP-43)
12mo, Spinal Cord (anti-human TDP-43)
12mo, Cortex (anti-human+mouse TDP-43)
12mo, Spinal Cord (anti-human+mouse TDP-43)
G298S
WT
D
EM
12mo
Control
TDP-43G298S
TDP-43WT
50μm
10μm
p<0.0001
p=0.0015
Axon diameter (um)
CT
G298S
WT
synaptophysin & α-bungarotoxin
2mo
28mo
H&E End Stage
Esterase End Stage
100μm

Supplementary Figure 1. Pathological findings in TDP-43WT and TDP-43G298S transgenic models. Animals exhibited marked motor disabilities starting with hind-limb clench at 2 months of age, progressing through a crawling gait at 12 month and continue with hemiparesis at 18 months, and up to end stage paralysis at around 24 months (**A**). Reduction in motor capabilities was accompanied by a decrease in weight compared to controls (**B**). Immunohistochemical images showed TDP-43 confined to the nucleus of cortical and spinal cord cells. Also, no cytoplasmic inclusions or TDP-43 clearance was observed. The upper panel shows immunohistochemistry using an antibody against TDP-43 and a in the bottom an antibody against human and mouse TDP-43 (**C**). In order to assess the lumbar L3 ventral roots of both wild-type (WT) and G298S transgenic mice at 12 months, Toluene-stained Epon thick sections were utilized. The L3 roots from transgenic mice exhibited smaller ventral roots than the non-transgenic animals. Further examination of the electron microscopy thick sections (EM) of the ventral roots in G298S and WT transgenic mice revealed a reduced number of large myelinated axons, resulting in the increased visibility of smaller axons. Quantitative analysis revealed statistical significance when comparing WT or G298S axon diameter against the control group. However, no significant differences were found between G298S and WT ($p = 0.2838$, ANOVA with Tukey's multiple comparisons test was conducted using GraphPad Prism v8.3.0, axon diameter was considered as the average between the largest and smaller axon diameter). Staining of synaptophysin (green) and α-bungarotoxin (red) on gastrocnemius muscles revealed a progressive denervation of neuromuscular junctions in both transgenic mice. While a significant number of junctions appeared well-innervated in the 2-month-old transgenic mice, denervated junctions were notably observed in the 12-month-old transgenic mice. At the end-stage of symptoms, transgenic mice experienced muscle degeneration to the extent that it was difficult to detect end plates by α-bungarotoxin-staining. We analyzed quadriceps muscle at their end-stage because the motor neurons innervating quadriceps muscle are in the L1 to L3 region of the spinal cord in mice. As the transgenic mice aged, H&E staining showed a progressively increased disorganization and angulation. Additionally, esterase staining indicated denervated fibers (darker staining) with the fiber muscle architecture completely altered in transgenic animals (**D**).

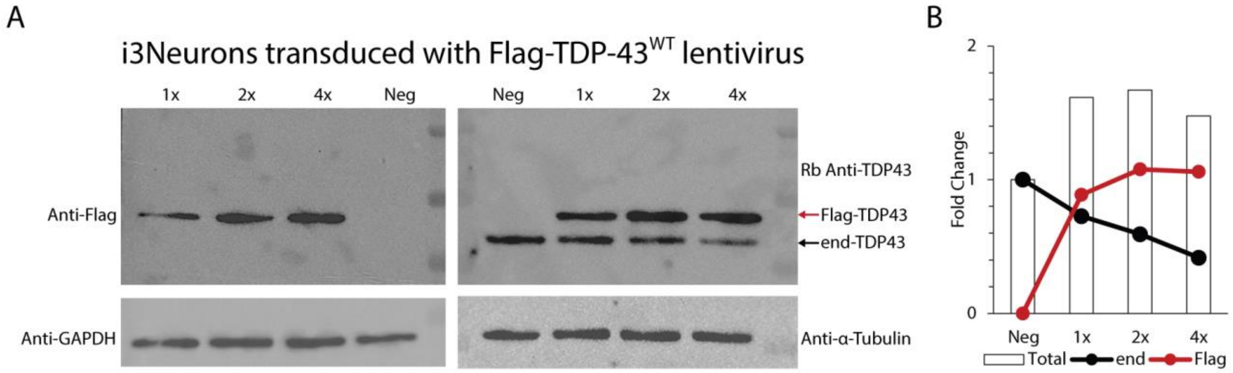

Supplementary Figure 2. i3Neuron transduction with human TDP-43. i3Neurons were transduced with lentivirus containing N-terminal Flag-tagged wild type TDP-43 at 1MOI, 2MOI, and 4MOI, respectively. (**A**) Consistent increase of exogenous TDP-43 (left) is seen accompanied with endogenous TDP-43 autoregulation (right). (**B**) Quantification of the right immunoblot, shows ~1.6 fold increase of total TDP-43 (endogenous TDP43 + transduced Flag-TDP43^{WT}).

A

Mouse

Gene	Control_1	Control_2	G298S_1	G298S_2	TDP43WT_1	TDP43WT_2
Arhgap44	89	78	31	28	13	8
Ddi2	100	96	52	48	64	42
Psme3	90	92	51	46	45	39
Dgkq	100	100	63	55	55	49
Mrpl45	99	99	62	61	49	49
Psmd14	100	100	58	62	53	60
Hjurp	96	95	49	44	75	64
Cops4	90	85	54	56	52	49
Tiam1	71	49	30	21	15	44
Creld1	100	100	72	66	70	61
Inpp4a	67	77	45	41	37	38
Shank1	59	57	27	27	31	27
Pakap	66	61	28	27	36	43
Arhgef11	94	76	71	70	40	43
Osbpl6	64	81	36	51	61	33
Pik3cb	93	100	72	80	71	54
Pdzd4	78	82	59	43	51	62
Dst	37	28	4	8	9	3
Herc2	100	91	76	80	64	58
Golga4	40	33	14	10	11	7
Stk24	56	50	42	23	21	21
Ube3c	100	97	75	76	75	64
C530008M17Rik	64	57	39	33	31	36
Tub	100	80	66	72	56	71
Atp2b1	67	65	41	46	45	38

C

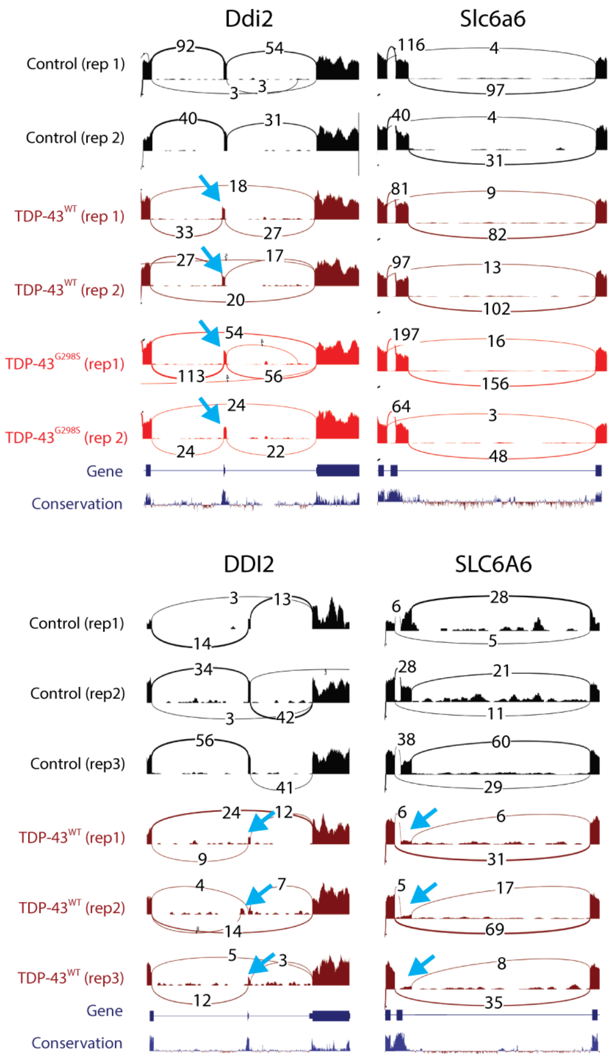

B

Human

Gene Name	Control(rep1)	Control(rep2)	Control(rep3)	TDP43WT(rep1)	TDP43WT(rep2)	TDP43WT(rep3)	Exon Type
XPNPEP1	100	99	100	24	23	26	skiptic
VARS2	98	98	99	20	14	19	skiptic
SLC35A5	100	100	100	32	40	26	skiptic
SESN3	100	100	97	53	25	47	skiptic
SBF1	81	81	76	15	8	14	skiptic
RCHY1	94	91	93	24	17	13	skiptic
RABGGTB	98	89	96	10	12	23	skiptic
PTS	91	86	92	16	11	17	skiptic
PLXNB1	95	97	97	27	21	21	skiptic
NUP93	100	99	100	32	28	34	skiptic
NUP88	100	93	91	35	36	25	skiptic
NRCAM	91	86	93	32	24	27	skiptic
NIFK	90	97	98	22	44	52	skiptic
MYBBP1A	91	86	95	14	14	10	skiptic
HYOU1	100	100	100	18	16	21	skiptic
GART	90	94	93	15	10	18	skiptic
FBXO22	95	89	89	25	28	26	skiptic
ELP2	96	96	97	27	29	41	skiptic
CSDE1	98	96	97	10	8	11	skiptic
CLASP2	94	92	96	28	27	31	skiptic
CERT1	70	73	72	17	10	20	skiptic
ZNF767P	82	88	85	21	20	8	skiptic
ZMYND11	78	64	63	2	6	12	skiptic
WSCD1	100	100	100	45	28	34	skiptic
WDR41	88	89	93	4	4	19	skiptic
TRIM16	100	100	100	-1	-1	-1	skiptic
TP53BP2	75	80	80	24	9	35	skiptic
TNR	100	95	95	24	32	31	skiptic
TMEM263	71	59	52	0	14	0	skiptic
TESK1	100	94	99	34	26	45	skiptic
STC2	100	100	-1	23	29	-1	skiptic
SLC17A5	100	86	80	11	11	27	skiptic
SCN9A	100	100	99	33	32	55	skiptic
RNF114	97	94	95	33	17	31	skiptic
RHOT2	100	97	91	50	30	38	skiptic
NIPAL3	59	86	91	2	0	6	skiptic
NICN1	85	83	94	29	25	31	skiptic
HIRA	100	99	100	45	41	45	skiptic
HCFC2	100	88	100	31	17	18	skiptic
HARS2	98	97	92	27	22	32	skiptic
FRAS1	100	100	100	29	38	30	skiptic
FBRS	100	98	98	34	25	39	skiptic
FAT1	88	91	79	11	11	20	skiptic
FAM102A	100	100	95	-1	-1	-1	skiptic
ERMAD	100	100	100	27	31	47	skiptic
DDI2	86	96	100	29	13	60	skiptic
COQ5	100	99	99	27	30	19	skiptic
CANX	99	100	99	46	37	49	skiptic
BMPR1A	100	100	100	20	19	26	skiptic
ATP9B	100	96	100	-1	44	13	skiptic
AMT	100	95	89	11	5	29	skiptic
WRAP73	100	100	100	38	29	38	skiptic
TLCD3A	100	92	95	38	29	28	skiptic
RNASET2	100	71	89	22	50	20	skiptic
ELP1	88	88	98	21	31	56	skiptic
CSPP1	100	94	100	22	17	38	skiptic
CCDC126	100	79	100	26	36	44	skiptic
BRI3BP	100	100	100	42	20	60	skiptic
FANCG	83	57	88	14	14	13	skiptic
TSPAN11	100	94	97	19	16	20	skiptic
NHLRC3	-1	100	100	-1	-1	-1	skiptic
NEBL	-1	100	100	40	-1	33	skiptic
INTS10	84	91	87	26	17	25	skiptic
DPY19L1P1	-1	100	100	20	-1	40	skiptic
TTBK2	-1	100	100	25	33	57	skiptic
NQO2	100	100	100	9	10	44	skiptic
ADAM23	83	63	64	7	2	23	skiptic
SUN1	100	96	95	0	4	0	skiptic
KCNMA1	74	59	66	-1	0	7	skiptic
ITGB1	100	100	100	7	5	14	mutually exclusive
SEC61A2	100	100	99	41	39	37	exon inc
PPA1	100	96	99	29	29	37	exon inc
DRGX	93	87	100	42	29	45	exon inc
LINC00680-GUSBP4	100	92	69	4	0	0	exon inc
HECW1	59	73	75	24	8	9	exon inc
SPRK2	86	65	84	21	13	29	exon inc
LGI4	88	85	97	27	20	50	exon inc
FAM66C	73	53	76	0	22	-1	alternative
SCN3A	100	99	100	51	29	31	alternative
ENSG00000256591	100	100	100	40	40	42	3' retention
ACTR8	-1	91	71	0	2	0	3' retention

Supplementary Figure 3. Differences in Exon Skipping Targets between Mouse and Human. Tables in panel A and B indicate percent spliced-in (PSI) values of skiptic exons across mouse and human samples. Analysis of RNA-seq datasets obtained from transgenic lines that overexpressed either TDP-43^{G298S} or TDP-43^{WT} and control littermates are shown, comparing our list of skipped exons with previously reported skiptic events in mouse (*1*), we found that 4 out of 25 were shared (*Creld1*, *Herc2*, *Psme3*, *Ube3c*, Supplementary Table 1, Tab-1,2) (**A**). RNA-seq data from human i3Neurons revealed that the set of repressed exons observed in mice differed from those observed in humans. Most splicing changes identified in this article correspond to skiptic exons (69/81, Supplementary Table 1, Tab2-3) (**B**). Under TDP-43 overexpression in both mouse and human samples our datasets the Ddi2 gene exhibits exon skipping. Prior reports (1) showed skiptic exons in PLOD1 and SLC6A6, our analysis confirms exon skipping for SLC6A6 in human i3N cells (**C**).

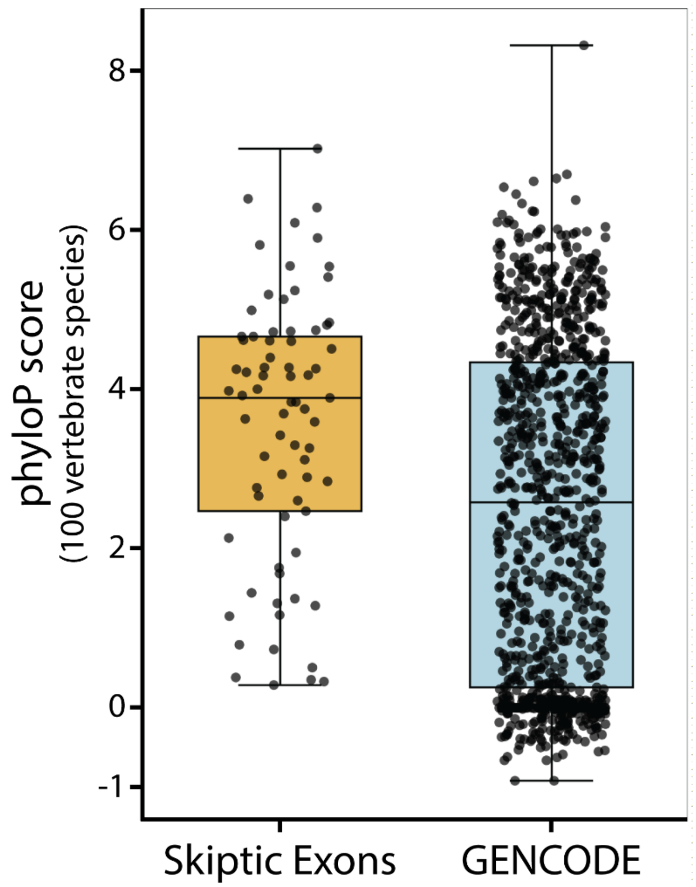

Supplementary Figure 4. Comparison of conservation scores between skiptic exons and GENCODE exons. The phyloP conservation scores, calculated across 100 vertebrate species, are presented for skiptic exons and a random sample of 1000 GENCODE v45 exons (*2*). Both box plots illustrate the mean and interquartile range, with individual data points representing individual exon scores. Skiptic exons have comparable levels of evolutionary conservation to GENCODE, with skiptic exons exhibiting a tendency toward higher conservation.

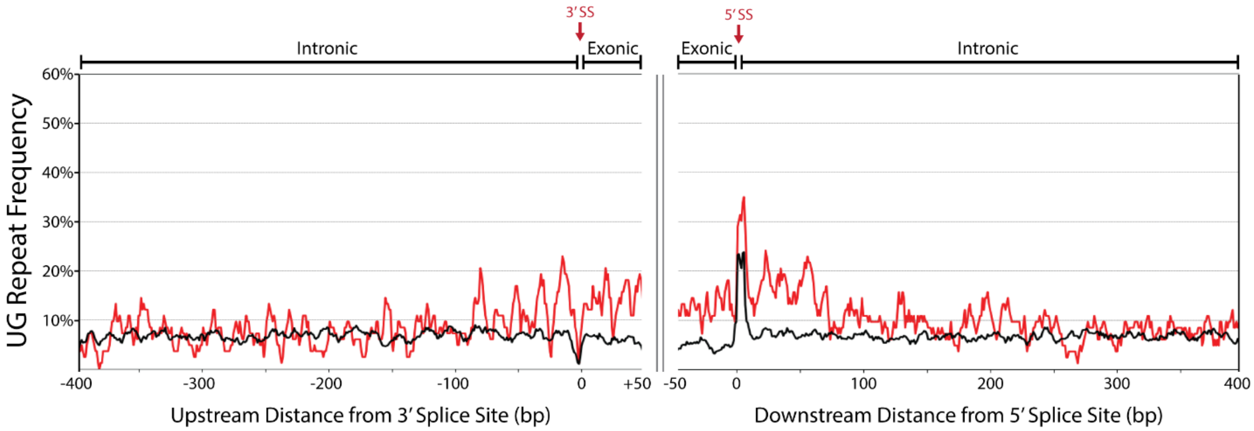

Supplementary Figure 5. UG/GU repeat frequency near skiptic exons. This plot represents the frequency of UG/UG dinucleotide repeats that are adjacent to human i3Neuron skiptic exons within a +/- 400bp window. A small 5-10% increase in UG repeat frequency can be detected within a 75bp window surrounding skiptic exons. By contrast, UG repeat frequency in cryptic exons ranges from 30-40% near the 5' splice site (*3*).

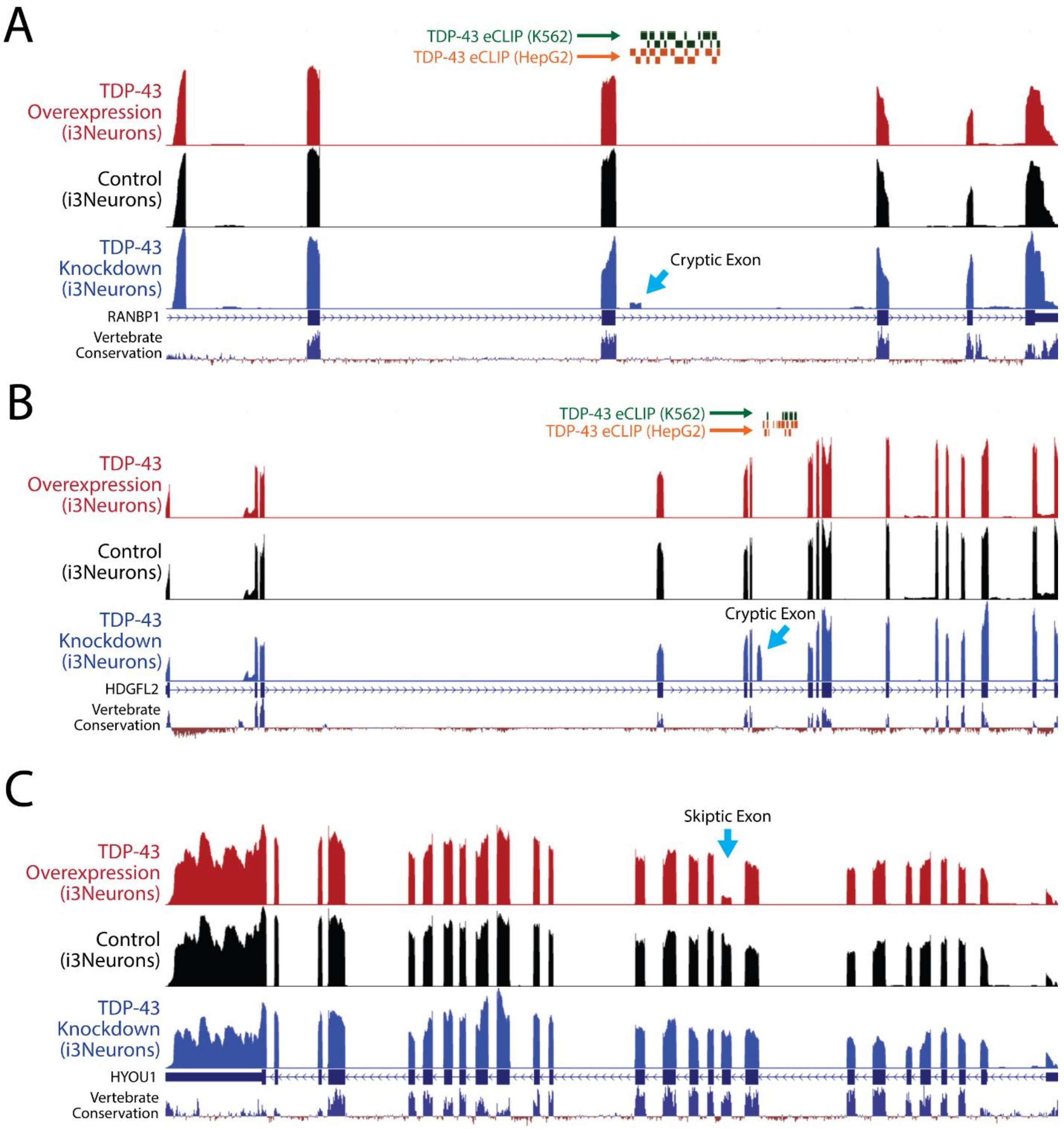
A
TDP-43 eCLIP (K562)
TDP-43 eCLIP (HepG2)
TDP-43 Overexpression (i3Neurons)
Control (i3Neurons)
TDP-43 Knockdown (i3Neurons)
Cryptic Exon
RANBP1
Vertebrate Conservation
B
TDP-43 eCLIP (K562)
TDP-43 eCLIP (HepG2)
TDP-43 Overexpression (i3Neurons)
Control (i3Neurons)
TDP-43 Knockdown (i3Neurons)
Cryptic Exon
HDGFL2
Vertebrate Conservation
C
Skiptic Exon
TDP-43 Overexpression (i3Neurons)
Control (i3Neurons)
TDP-43 Knockdown (i3Neurons)
HYOU1
Vertebrate Conservation

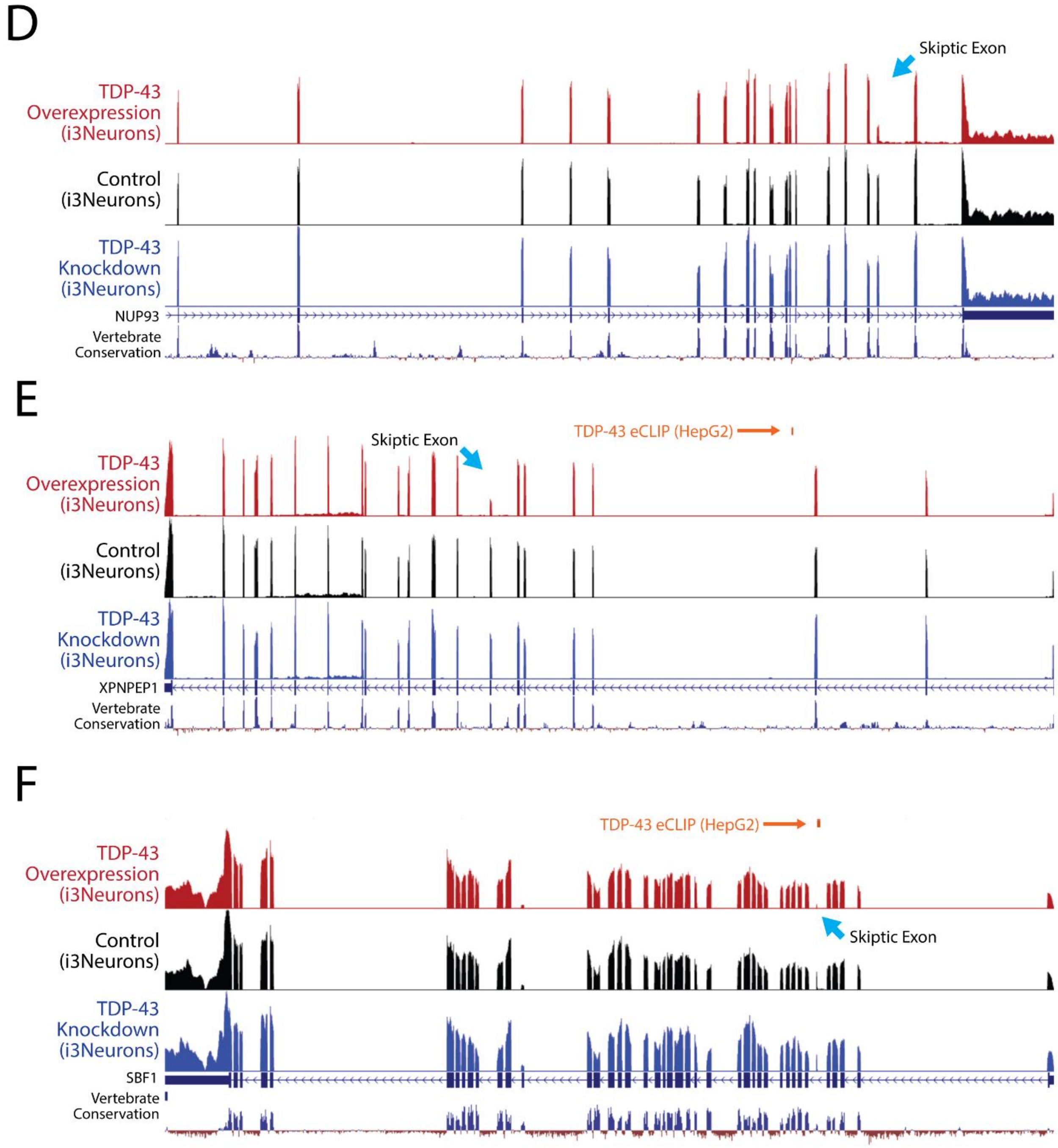
D
Skiptic Exon
TDP-43 Overexpression (i3Neurons)
Control (i3Neurons)
TDP-43 Knockdown (i3Neurons)
NUP93
Vertebrate Conservation
E
TDP-43 eCLIP (HepG2)
Skiptic Exon
TDP-43 Overexpression (i3Neurons)
Control (i3Neurons)
TDP-43 Knockdown (i3Neurons)
XPNPEP1
Vertebrate Conservation
F
TDP-43 eCLIP (HepG2)
TDP-43 Overexpression (i3Neurons)
Skiptic Exon
Control (i3Neurons)
TDP-43 Knockdown (i3Neurons)
SBF1
Vertebrate Conservation

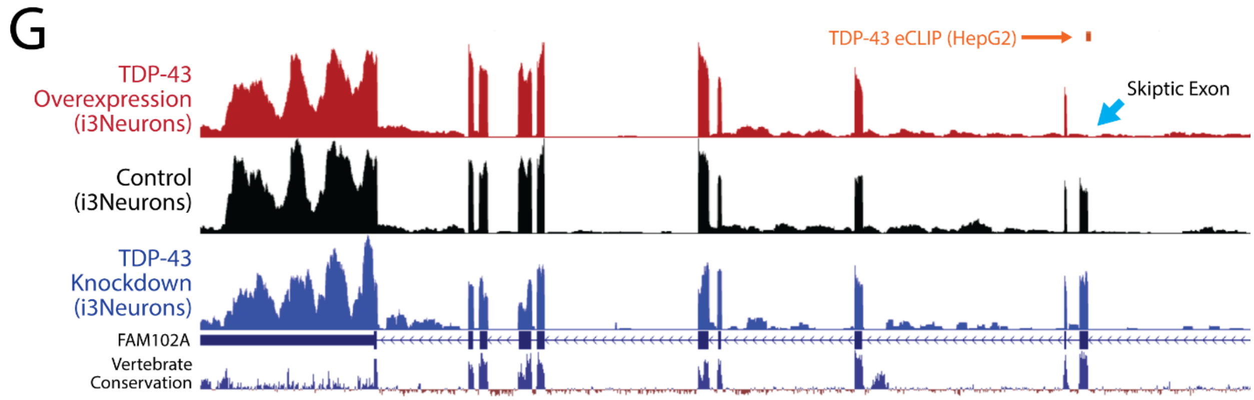

Supplementary Figure 6. TDP-43 eCLIP data from HepG2 and K562 cells. TDP-43 eCLIP datasets were generated by the ENCODE consortium (*4*) in HepG2 and K562 cells. Large TDP-43 eCLIP footprints are detected adjacent to TDP-43 cryptic exons in RANBP1 (**A**) and HDGFL2 (**B**). By contrast, most skiptic exons do not have any adjacent eCLIP footprints for either HepG2 or K562, despite significant exon skipping; representative skiptic events in HYOU1 (**C**), NUP93 (**D**), and XPNPEP1 (**E**). A minority of skiptic exons have single eCLIP footprints adjacent to the skiptic exon, for example SBF1 (**F**) and FAM102A (**G**). TDP-43 eCLIP data supports the hypothesis that TDP-43 binds strongly to sites adjacent to cryptic exons and binds weakly to sites adjacent to skiptic exons.

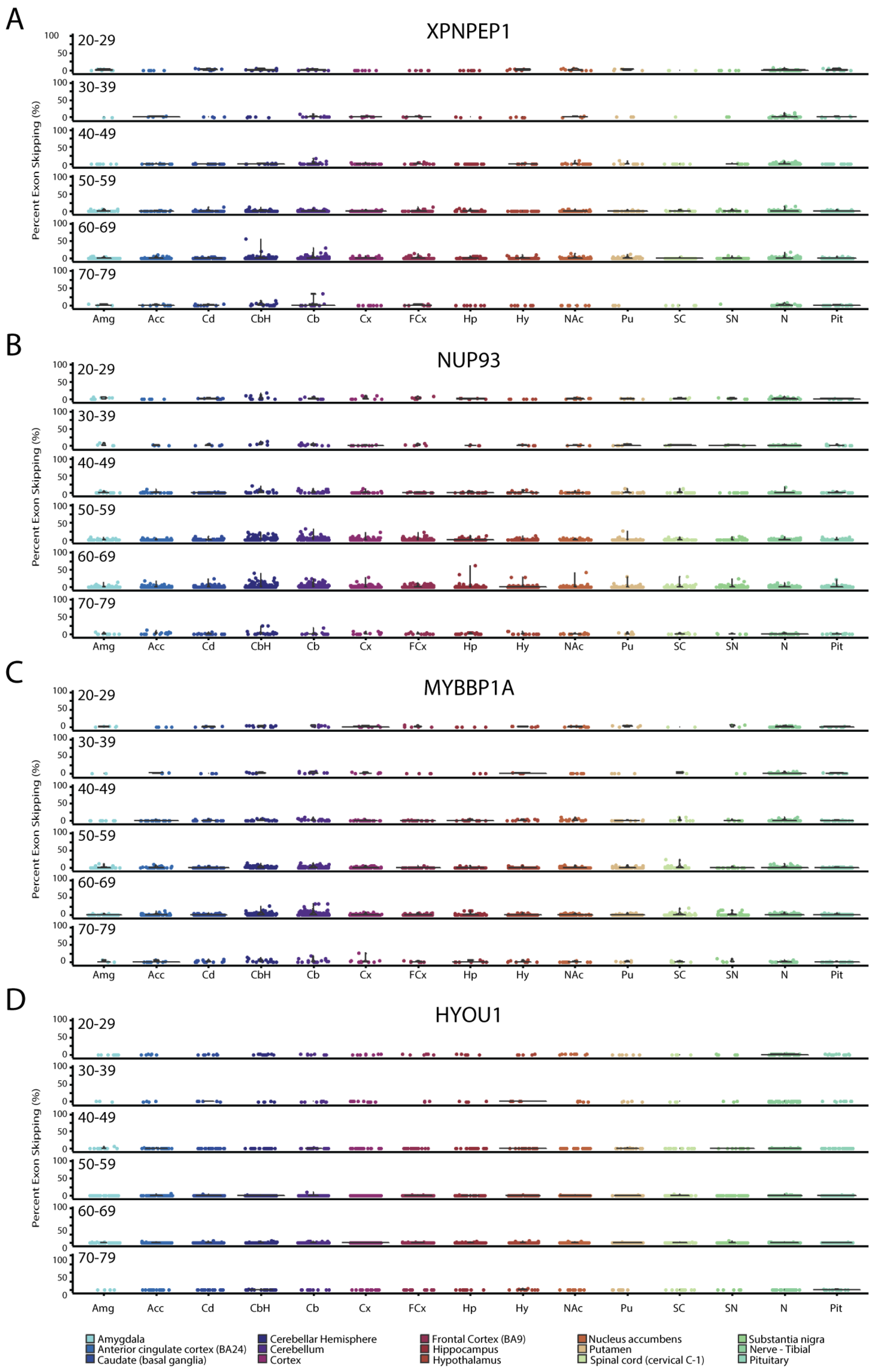
A
XPNPEP1
B
NUP93
C
MYBBP1A
D
HYOU1
Percent Exon Skipping (%)
20-29
30-39
40-49
50-59
60-69
70-79
100
50
0
Amg
Acc
Cd
CbH
Cb
Cx
FCx
Hp
Hy
NAc
Pu
SC
SN
N
Pit
Amygdala
Anterior cingulate cortex (BA24)
Caudate (basal ganglia)
Cerebellar Hemisphere
Cerebellum
Cortex
Frontal Cortex (BA9)
Hippocampus
Hypothalamus
Nucleus accumbens
Putamen
Spinal cord (cervical C-1)
Substantia nigra
Nerve - Tibial
Pituitary

Supplementary Figure 7. Exons skipping events are found in human brain samples from publicly available archives.. Graph show the percentage of exon skipping for four genes (XPNPEP1, NUP93, MYBBP1A and HYOU1) from the Genotype-Tissue Expression (GTEx) Portal at ages ranging from 20-29, 30-39, 40-49, 50-59, 60-69, and 70-79. Skipping events were found in most of the different brain areas analyzed, with a higher frequency in the cerebellum and cortex.

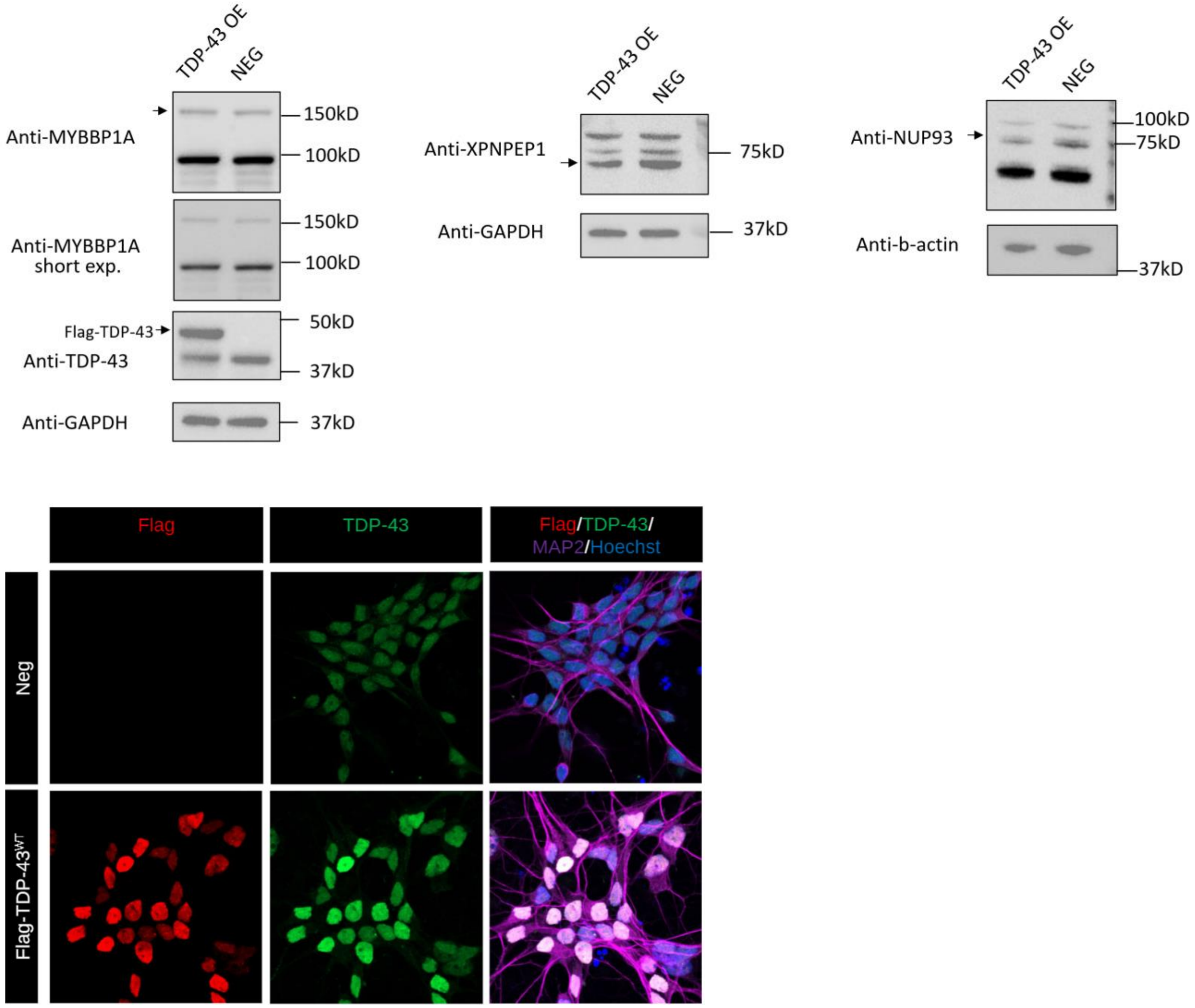

Supplementary Figure 8. Immunoblot analysis of proteins with inframe skiptic exons. (Top) Immunoblots for *MYBBP1A*, *XPNPEP1*, and *NUP9,* while *MYBBP1A* is unaffected by the skiptic exon induced by TDP-43 overexpression (TDP-43 OE), protein levels of *XPNPEP1* and *NUP93* are modestly reduced upon TDP-43 overexpression. Nevertheless, it is difficult to determine whether changes in protein levels are a direct result of skiptic exons or downstream consequences of TDP-43 overexpression. (Bottom) Lentiviral overexpression of FLAG-tagged TDP-43 (red) in i3Neurons does not lead to cytoplasmic aggregation and is localized to the nucleus like total TDP-43 (green). Cytoplasm compartment can be inferred by MAP2 (magenta) and nuclear compartment can be identified using Hoechst stain (blue).

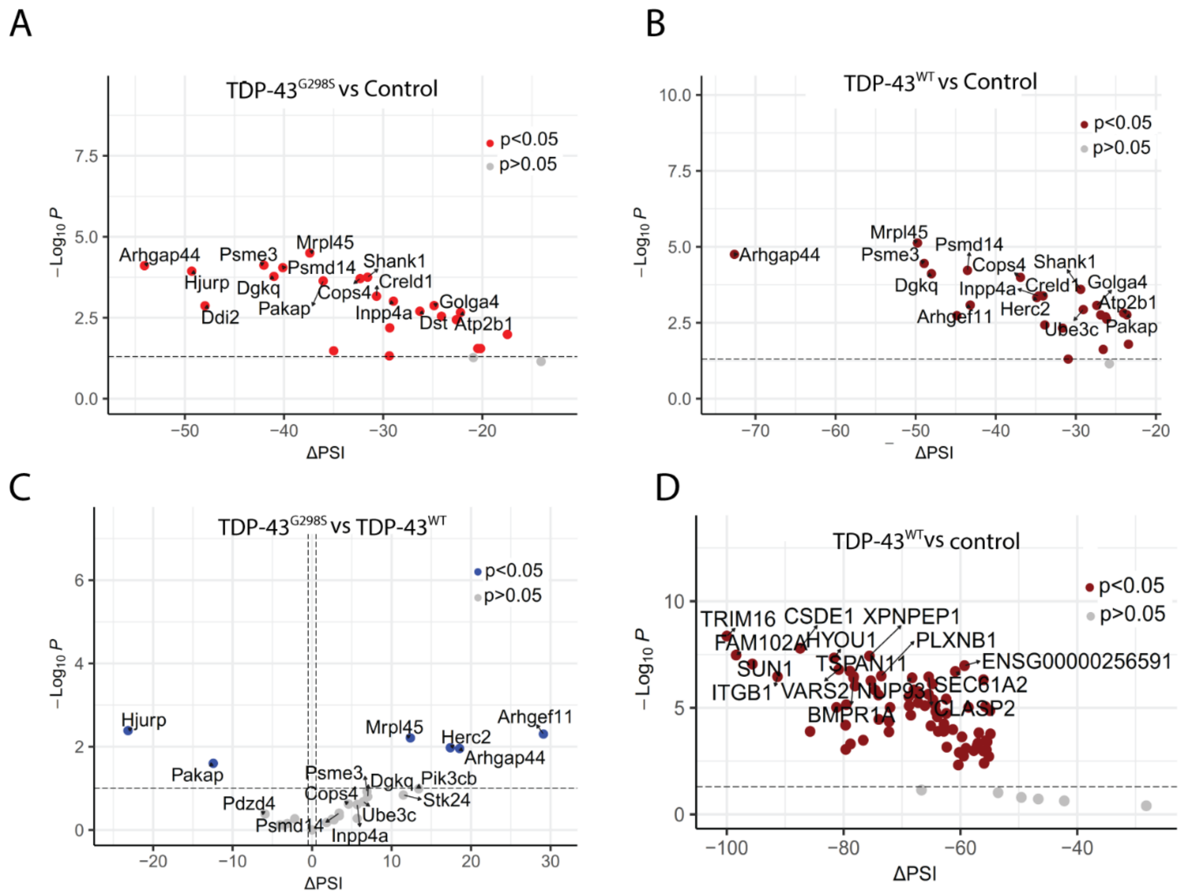

Supplementary Figure 9. Differences in PSI values in transgenic mouse. Our manual analysis showed similar splicing changes in TDP-43^{WT} and TDP-43^{G298S} compared to the control mouse, we used *limma* to evaluate statistical changes between PSI value across the different groups. While most skipping events are conserved, three out of the 25 genes (*Osbpl6*, *Argef11* and *Tub*) appear not to be skipped in both TDP-43^{WT} and TDP-43^{G298S} groups (p-values included in Supplementary Table 1, Tab 1) (**A, B**). When we compared PSI values between TDP-43 overexpressing lines, differences were found in 6 genes; 4 of them, *Arhgef11*, *Herc2*, *Mrpl45*, and *Arhgap44*, were more repressed in TDP-43^{WT} compared to TDP-43^{G298S}, and 2 of them, *Hjurp* and *Pakap* were more repressed in TDP-43^{G298S} compared to TDP-43^{WT}. Since there is not a clear trend for transgenic lines to exclusively be a stronger splicing repressor, we cannot attribute a gain of function to the G298S mutation especially considering that in both cases TDP-43 is increased (**C**). Using the same approach, we compared PSI values in the transduced i3Neurons with TDP-43 or not and we found that 5 genes do not show a significant splicing change (*STC2*, *NHLRC3*, *NEBL*, *DPY19L1P1*, *TTBK2*, *IL17RB*), however visual examination and manual curation indicates that those events appear to be present when inspected by sashimi plots (p-values included in Supplementary Table 1, Tab-3) (**D**).

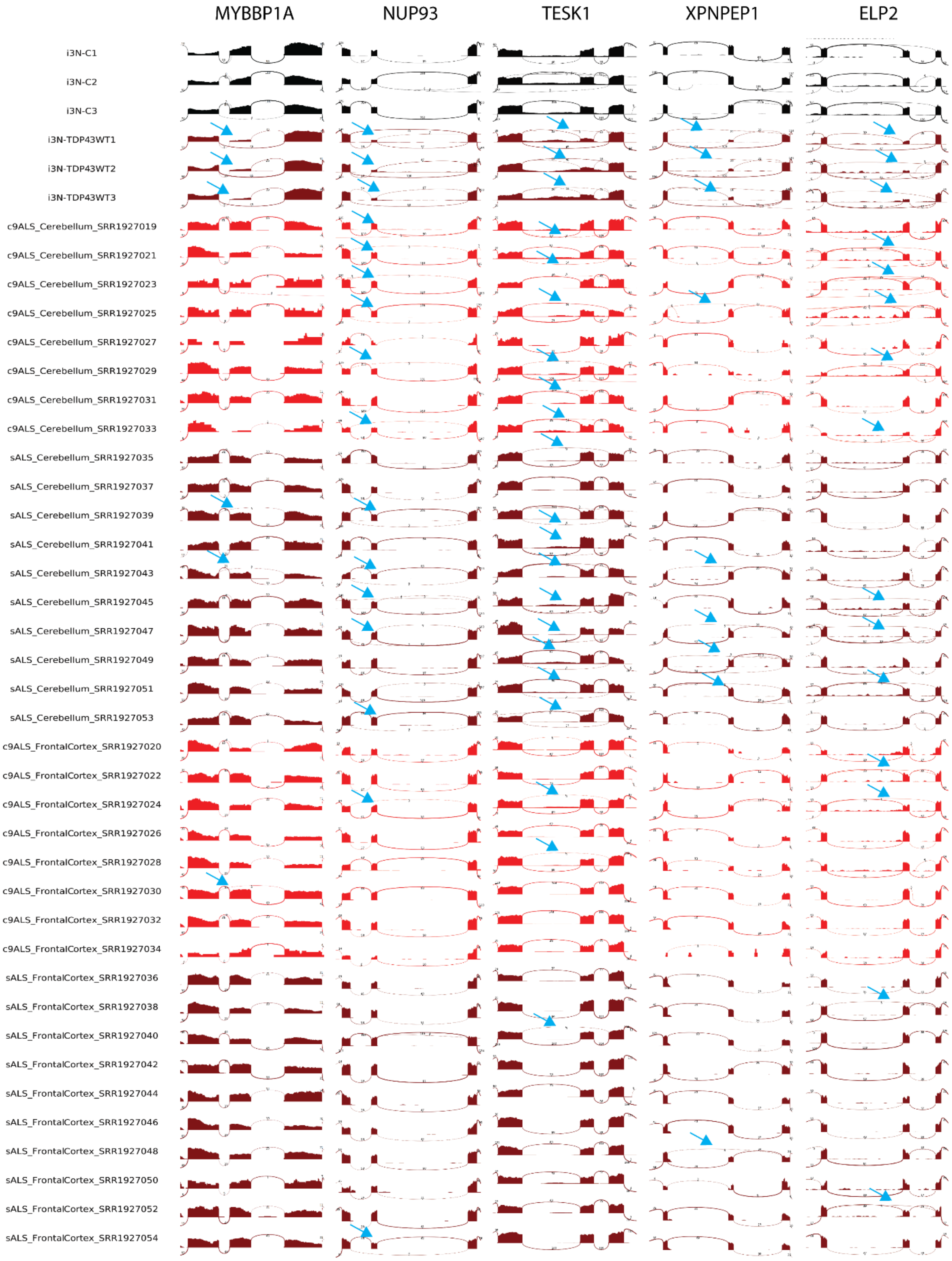

MYBBP1A
NUP93
TESK1
XPNPEP1
ELP2
i3N-C1
i3N-C2
i3N-C3
i3N-TDP43WT1
i3N-TDP43WT2
i3N-TDP43WT3
c9ALS_Cerebellum_SRR1927019
c9ALS_Cerebellum_SRR1927021
c9ALS_Cerebellum_SRR1927023
c9ALS_Cerebellum_SRR1927025
c9ALS_Cerebellum_SRR1927027
c9ALS_Cerebellum_SRR1927029
c9ALS_Cerebellum_SRR1927031
c9ALS_Cerebellum_SRR1927033
sALS_Cerebellum_SRR1927035
sALS_Cerebellum_SRR1927037
sALS_Cerebellum_SRR1927039
sALS_Cerebellum_SRR1927041
sALS_Cerebellum_SRR1927043
sALS_Cerebellum_SRR1927045
sALS_Cerebellum_SRR1927047
sALS_Cerebellum_SRR1927049
sALS_Cerebellum_SRR1927051
sALS_Cerebellum_SRR1927053
c9ALS_FrontalCortex_SRR1927020
c9ALS_FrontalCortex_SRR1927022
c9ALS_FrontalCortex_SRR1927024
c9ALS_FrontalCortex_SRR1927026
c9ALS_FrontalCortex_SRR1927028
c9ALS_FrontalCortex_SRR1927030
c9ALS_FrontalCortex_SRR1927032
c9ALS_FrontalCortex_SRR1927034
sALS_FrontalCortex_SRR1927036
sALS_FrontalCortex_SRR1927038
sALS_FrontalCortex_SRR1927040
sALS_FrontalCortex_SRR1927042
sALS_FrontalCortex_SRR1927044
sALS_FrontalCortex_SRR1927046
sALS_FrontalCortex_SRR1927048
sALS_FrontalCortex_SRR1927050
sALS_FrontalCortex_SRR1927052
sALS_FrontalCortex_SRR1927054

Supplementary Figure 10. Representative data in sashimi plot showing exon skipping events in human ALS datasets. We examined human datasets from six independent research groups, focusing on ALS patients with TDP-43 mutations (Q331K, M337V, K263E, Q343R, and G376D), as well as C9ORF72 cases, and data from sporadic ALS patients, alongside a recent study employing colony morphology neural stem cells (iCoMoNSCs). Sashimi plots for the explored exons are provided in supplementary data files (Supplementary Data File 3-7). Our analysis of exon skipping for the genes BMPR1A, CANX, COQ5, ELP2, HYOU1, MYBBP1A, NUP93, SCN9A, SESN3, SLC35A5, TESK1, VARS2, WSCD1, and XPNPEP1 revealed occurrences in both C9ORF72 and sporadic ALS cases. Notably, MYBBP1A, NUP93, TESK1, and XPNPEP1 exhibited particularly enriched exon skipping events in the cerebellum, consistent with our findings from GTEx data included in Figure 3 (Supplementary Data File 3). Blue arrows indicate tracks where skiptic events are observed for the data from both C9ORF72 and sporadic patients. Furthermore, we observed exon skipping events in the ELP2 and SLC6A6 genes associated with M337V mutations, though limited to samples from a single study (Supplementary Data File 4). Skiptic events from differentiated human iCoMoNSCs were also detected in a variety of genes including BMPR1A (5.05%), ELP2 (9.02%), HYOU1 (1.66%), MYPBBP1A (8.37%), NUP93 (4.67%), VARS2 (3.01%) upon induction with of TDP-43 with DOX (Supplementary Data File 5), although as mentioned in the limitations of our study implications of these skiptic events in the disease context need to be study. In contrast, exon skipping events are absent or diminished in loss-of-function models of iPSCs, NPTX2 organoids, and the loss-of-function mutation in the RRM2mut as reported by Fratta et al. (Coordinates and genes explored in this analysis are included in Supplementary Table 1, Tab-4).

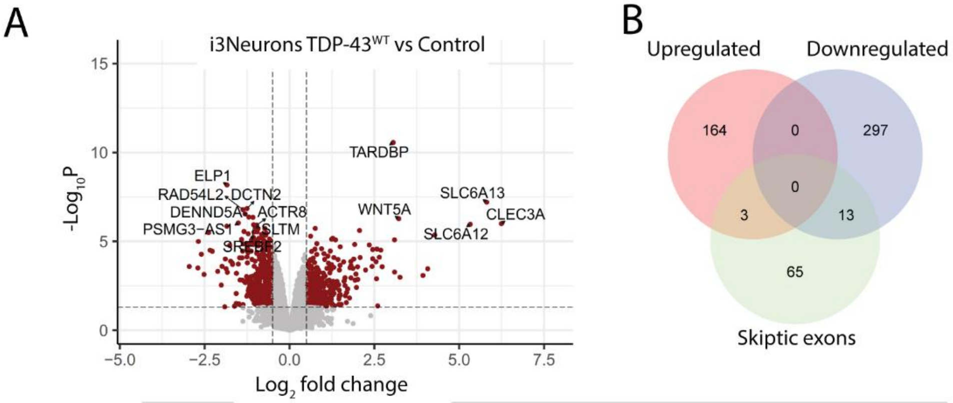

C

Gene	Control(rep1)	Control(rep2)	Control(rep3)	TDP43WT(rep1)	TDP43WT(rep2)	TDP43WT(rep3)	Control(rep1)	Control(rep2)	Control(rep3)	TDP43WT(rep1)	TDP43WT(rep2)	TDP43WT(rep3)	CorrelationCoef	P_Value
RNF114	5.551443	5.67431	5.619727	4.830276	4.764727	4.813614	97.14	93.75	95.12	33.33	16.67	30.56	0.988216	0.000207
CSDE1	9.481697	9.477659	9.447942	9.252131	9.163892	9.198622	98.12	96.17	97.42	9.94	8.41	11.07	0.979876	0.000603
PLXNB1	7.272659	7.413306	7.204437	6.351863	6.518521	6.284163	94.87	97.03	96.95	26.67	20.93	20.59	0.977362	0.000763
HARS2	5.376356	5.322462	5.190577	4.688448	4.799258	4.805673	97.50	97.10	92.22	27.03	21.57	31.71	0.974717	0.000951
COQ5	4.857026	4.890672	4.82362	4.444104	4.329527	4.445755	100.00	99.01	99.24	27.45	30.19	18.52	0.966642	0.001651
ITGB1	7.152799	7.0418	7.178646	6.808241	6.716702	6.749169	100.00	100.00	100.00	6.56	5.04	14.44	0.96288	0.002041
NUP88	4.894647	5.010147	5.175164	3.802327	3.817272	3.896956	100.00	92.86	90.59	34.78	36.36	25.00	0.962649	0.002067
ELP2	5.228042	5.296938	5.273976	4.812451	4.960508	5.022176	96.30	95.89	97.06	26.92	28.57	41.07	0.960145	0.002351
TESK1	6.308988	6.412267	6.231638	5.397787	5.249841	5.14685	100.00	94.23	98.61	34.00	25.58	44.83	0.953793	0.003153
ELP1	7.602069	7.534835	7.613927	5.68294	5.691358	5.859727	87.50	88.03	97.50	20.83	31.25	55.56	0.951778	0.003432
FANCG	4.372361	4.274228	4.494921	3.802327	4.024846	4.025598	83.33	56.76	88.00	14.29	14.29	12.50	0.938084	0.005632
SCN3A	8.35947	8.385196	8.299613	7.637631	7.87013	7.390414	100.00	99.47	100.00	50.77	29.41	30.77	0.90552	0.012968
NICN1	5.734783	5.54042	5.417125	5.068556	4.947096	4.781583	84.85	82.86	93.55	29.03	25.35	31.03	0.896829	0.015417
LGI4	3.706504	3.930223	3.726154	2.887796	2.993502	2.843658	87.50	85.00	97.30	26.67	20.00	50.00	0.892525	0.016706
RABGGTB	5.216204	5.244494	5.191599	4.987473	5.095148	5.117211	98.44	89.40	95.60	10.19	11.57	23.08	0.88772	0.018203
PTS	4.608026	4.596418	4.831507	4.41782	4.228619	4.096747	91.01	86.24	92.00	15.57	10.87	16.79	0.878743	0.021163
VARS2	6.487018	6.489221	6.229152	5.807398	6.089491	5.768705	98.18	98.40	99.22	20.00	13.85	19.44	0.867909	0.02502
SEC61A2	6.17781	6.047742	6.332349	5.774401	5.790166	5.928381	100.00	100.00	99.29	40.74	39.19	36.73	0.867845	0.025044
SUN1	7.243797	7.252981	7.302748	7.36587	7.603258	7.53712	100.00	96.30	94.74	0.00	4.17	0.00	-0.84192	0.03551
FBXO22	4.774676	4.899178	4.675201	5.287592	5.298933	4.978103	95.24	89.32	88.55	25.49	27.66	25.53	-0.84616	0.033678
RNASET2	2.163241	2.138555	2.215404	2.407467	2.425218	2.679539	100.00	70.59	89.47	22.22	50.00	20.00	-0.85458	0.030182
GART	6.068487	6.045445	6.122294	6.191307	6.160109	6.186633	90.00	93.94	93.48	14.81	10.20	17.54	-0.88568	0.018857
TSPAN11	5.042746	4.894081	4.561245	6.135947	6.033483	5.60447	100.00	94.44	97.30	19.05	15.91	20.00	-0.93033	0.007112
MYBBP1A	5.152411	5.205244	5.159584	5.276258	5.356589	5.387535	91.30	85.71	94.74	14.29	13.79	9.52	-0.93334	0.006517
SLC17A5	4.40922	4.315488	4.372321	4.707862	4.68387	4.566594	100.00	85.71	79.55	10.53	11.11	26.92	-0.94258	0.00485
INTS10	5.631613	5.682218	5.759265	5.961929	5.964793	5.989248	84.21	91.30	86.59	26.14	17.24	24.64	-0.95482	0.003015
SLC35A5	5.692688	5.785855	5.792643	6.160814	6.166736	6.107016	100.00	100.00	100.00	32.47	39.51	26.32	-0.96598	0.001716
HIRA	6.794216	6.843188	6.769394	7.285373	7.23119	7.173579	100.00	98.74	100.00	44.78	40.65	45.16	-0.98459	0.000354
RHOT2	6.06354	6.252151	5.995228	5.937468	5.950461	5.827408	100.00	96.50	91.25	50.00	29.73	37.50	0.757473	0.081096
ZNF767P	4.845546	4.83501	4.633703	4.484063	4.706788	4.435499	82.35	87.50	85.37	20.51	20.00	8.33	0.75586	0.082131
WRAP73	3.780717	3.844731	3.792307	3.299638	3.394491	3.788315	100.00	100.00	100.00	37.50	28.57	37.50	0.736342	0.095109
WDR41	5.759463	5.921481	5.8338	5.654457	5.531096	5.161443	88.46	89.23	92.55	3.85	3.60	19.12	0.703689	0.118692
ZMYND11	7.044839	7.159064	7.106265	7.044658	6.967879	6.978397	77.59	63.51	63.30	2.36	6.25	11.93	0.691892	0.127771
HECW1	6.72176	6.687384	6.637269	6.576402	6.60707	6.287968	58.82	73.47	75.00	24.32	8.16	8.57	0.679638	0.137508
NRCAM	8.02186	8.072492	7.899288	7.874301	8.001981	7.72779	91.35	86.27	93.27	32.32	23.89	27.36	0.517127	0.293455
NUP93	6.275157	6.193189	6.63201	5.790424	5.947578	6.583336	100.00	99.16	99.64	32.18	27.68	34.13	0.450319	0.370181
CERT1	5.664653	5.759001	5.718022	5.613868	5.737718	5.621158	70.00	72.86	71.88	16.67	10.45	20.37	0.44624	0.37507
WSCD1	5.349514	5.391616	5.636332	5.306818	5.274596	5.476927	100.00	100.00	100.00	45.45	28.21	34.48	0.431813	0.392538
PPA1	6.105856	5.922316	6.171284	6.004746	5.982744	6.045404	100.00	96.43	99.25	28.68	28.57	36.54	0.381863	0.455047
CLASP2	9.227765	9.339165	9.225946	9.23446	9.281625	9.061542	93.58	92.34	96.43	28.22	26.76	31.09	0.380666	0.456582
DDI2	2.235391	2.638686	2.929688	2.442656	2.44716	2.801686	85.71	96.43	100.00	28.57	12.50	60.00	0.377967	0.460047
BRI3BP	1.223614	1.306562	1.664202	0.925598	1.29213	1.565027	100.00	100.00	100.00	41.67	20.00	60.00	0.374079	0.465054
NIFK	3.637172	3.636653	3.952771	3.619646	3.757061	3.733557	89.66	96.97	98.48	22.22	44.44	51.85	0.316399	0.541239
TLCD3A	3.968834	4.063343	4.140134	3.906821	4.024846	4.070476	100.00	92.00	95.24	37.50	29.03	27.78	0.284381	0.584928
CSPP1	4.434984	4.35807	4.216408	4.170905	4.483448	4.074888	100.00	93.75	100.00	22.22	17.39	37.50	0.193829	0.712897
TNR	5.948407	5.700827	6.535522	5.75936	5.864408	6.312461	100.00	94.87	94.69	24.14	32.14	31.03	0.147903	0.779764
FBRS	6.824745	6.819184	6.523806	6.86122	6.720648	6.520032	100.00	98.40	97.64	33.87	25.20	38.71	0.051345	0.923049
XPNPEP1	6.104251	6.138195	6.187764	6.082102	6.072828	6.26884	100.00	99.34	100.00	23.81	23.15	25.69	0.034328	0.948529
HYOU1	7.600362	7.449376	7.85934	7.537428	7.490564	7.867854	100.00	100.00	100.00	18.13	16.28	20.69	0.034025	0.948983
SCN9A	7.500196	8.026643	7.150286	7.553949	7.827372	6.847853	100.00	100.00	99.11	32.69	32.35	54.84	0.015925	0.976114
DRGX	3.828152	4.026546	3.770591	3.846789	3.949981	3.688203	93.33	86.96	100.00	41.67	28.57	45.45	0.00335	0.994975
FRAS1	6.25505	6.024603	6.488492	6.246282	6.268062	6.265949	100.00	100.00	100.00	28.57	37.50	30.00	-0.01215	0.981774
SESN3	6.808084	6.686402	6.873337	6.820555	6.699094	6.945045	100.00	100.00	97.14	53.33	25.00	47.37	-0.01683	0.974763
SBF1	8.408083	8.426681	8.258275	8.428373	8.408801	8.292119	81.43	80.70	75.76	14.89	7.80	13.64	-0.0567	0.915035
BMPR1A	5.242703	5.184515	4.953374	5.335184	5.142986	4.937494	100.00	100.00	100.00	20.00	18.87	26.32	-0.07762	0.883806
CCDC126	2.623793	2.614094	3.139075	2.929738	2.802453	2.914294	100.00	78.57	100.00	26.32	36.36	44.44	-0.12446	0.814278
TMEM263	3.093776	3.304002	3.383989	3.179622	3.354414	3.308251	71.43	58.82	51.61	0.00	13.64	0.00	-0.20849	0.691795
RCHY1	5.9591	6.173688	5.797349	6.220324	6.099051	5.881298	93.75	91.46	92.50	23.73	17.28	12.93	-0.24616	0.638213
AMT	4.030969	4.026546	4.075185	4.191738	4.186845	3.814934	100.00	95.24	88.68	11.11	5.26	28.57	-0.25271	0.628999
HCFC2	4.538429	4.81896	4.947337	4.895224	5.04926	4.663517	100.00	87.50	100.00	31.25	16.67	17.65	-0.30097	0.562182
NIPAL3	6.157836	6.130991	5.506052	6.45312	6.243194	5.599886	59.26	86.36	91.38	1.52	0.00	5.56	-0.36341	0.478885
CANX	8.477907	8.427857	8.845004	8.748782	8.748458	8.991802	98.57	99.53	98.93	46.13	36.94	49.17	-0.58146	0.226107
ADAM23	5.79968	5.822989	5.772982	5.841855	5.857256	5.786299	83.33	62.71	63.95	7.41	2.00	23.40	-0.62742	0.182362
NQO2	3.637172	4.102176	4.177744	4.426634	4.323608	4.492676	100.00	100.00	100.00	9.09	10.00	44.44	-0.71156	0.112797
FAT1	7.21806	6.964875	6.664018	7.696214	7.737023	7.108899	87.50	91.30	79.31	11.11	10.87	20.00	-0.74686	0.088011
TP53BP2	5.565506	5.448362	5.30263	5.704846	5.708492	5.448308	75.00	80.00	79.66	23.68	9.30	34.78	-0.74723	0.087766

Supplementary Figure 11. Gene expression analysis in human iPSC neurons. We performed an RNA-seq expression analysis using Voom and *limma* packages. Top expressed or downregulated genes are not included in our exon skipping or splice change list (**A**). We found that 16 genes of our described splicing changes events change their expression and most of them remain unchanged (65/81), gene list are included in Supplementary Table 1, Tab-5,6) (**B**). We also correlated expression levels with PSI values from the described splicing changes list and 42% (28/66) of our described gene list presented correlation, though most of them did not (**C**). In Yellow CPM values are included, blue scale shows PSI, (Pearson correlation coefficients and corresponding p-values are shown).

Supplementary File References:

1. P. Fratta, P. Sivakumar, J. Humphrey, K. Lo, T. Ricketts, H. Oliveira, J. M. Brito-Armas, B. Kalmar, A. Ule, Y. Yu, N. Birsa, C. Bodo, T. Collins, A. E. Conicella, A. M. Maza, A. Marrero-Gagliardi, M. Stewart, J. Mianne, S. Corrochano, W. Emmett, G. Codner, M. Groves, R. Fukumura, Y. Gondo, M. Lythgoe, E. Pauws, E. Peskett, P. Stanier, L. Teboul, M. Hallegger, A. Calvo, A. Chiò, A. M. Isaacs, N. L. Fawzi, E. Wang, D. E. Housman, F. Baralle, L. Greensmith, E. Buratti, V. Plagnol, E. M. Fisher, A. Acevedo-Arozena, Mice with endogenous TDP-43 mutations exhibit gain of splicing function and characteristics of amyotrophic lateral sclerosis. *EMBO J.* **37**, e98684 (2018).

2. A. Frankish, M. Diekhans, I. Jungreis, J. Lagarde, J. E. Loveland, J. M. Mudge, C. Sisu, J. C. Wright, J. Armstrong, I. Barnes, A. Berry, A. Bignell, C. Boix, S. Carbonell Sala, F. Cunningham, T. Di Domenico, S. Donaldson, I. T. Fiddes, C. García Girón, J. M. Gonzalez, T. Grego, M. Hardy, T. Hourlier, K. L. Howe, T. Hunt, O. G. Izuogu, R. Johnson, F. J. Martin, L. Martínez, S. Mohanan, P. Muir, F. C. P. Navarro, A. Parker, B. Pei, F. Pozo, F. C. Riera, M. Ruffier, B. M. Schmitt, E. Stapleton, M.-M. Suner, I. Sycheva, B. Uszczynska-Ratajczak, M. Y. Wolf, J. Xu, Y. T. Yang, A. Yates, D. Zerbino, Y. Zhang, J. S. Choudhary, M. Gerstein, R. Guigó, T. J. P. Hubbard, M. Kellis, B. Paten, M. L. Tress, P. Flicek, GENCODE 2021. *Nucleic Acids Res.* **49**, gkaa1087- (2020).

3. S. P. McClory, K. W. Lynch, J. P. Ling, HnRNP L represses cryptic exons. *Rna* **24**, 761–768 (2018).

4. F. Abascal, R. Acosta, N. J. Addleman, J. Adrian, V. Afzal, R. Ai, B. Aken, J. A. Akiyama, O. A. Jammal, H. Amrhein, S. M. Anderson, G. R. Andrews, I. Antoshechkin, K. G. Ardlie, J. Armstrong, M. Astley, B. Banerjee, A. A. Barkal, I. H. A. Barnes, I. Barozzi, D. Barrell, G. Barson, D. Bates, U. K. Baymuradov, C. Bazile, M. A. Beer, S. Beik, M. A. Bender, R. Bennett, L. P. B. Bouvrette, B. E. Bernstein, A. Berry, A. Bhaskar, A. Bignell, S. M. Blue, D. M. Bodine, C. Boix, N. Boley, T. Borrman, B. Borsari, A. P. Boyle, L. A. Brandsmeier, A. Breschi, E. H. Bresnick, J. A. Brooks, M. Buckley, C. B. Burge, R. Byron, E. Cahill, L. Cai, L. Cao, M. Carty, R. G. Castanon, A. Castillo, H. Chaib, E. T. Chan, D. R. Chee, S. Chee, H. Chen, H. Chen, J.-Y. Chen, S. Chen, J. M. Cherry, S. B. Chhetri, J. S. Choudhary, J. Chrast, D. Chung, D. Clarke, N. A. L. Cody, C. J. Coppola, J. Coursen, A. M. D'Ippolito, S. Dalton, C. Danyko, C. Davidson, J. Davila-Velderrain, C. A. Davis, J. Dekker, A. Deran, G. DeSalvo, G. Despacio-Reyes, C. N. Dewey, D. E. Dickel, M. Diegel, M. Diekhans, V. Dileep, B. Ding, S. Djebali, A. Dobin, D. Dominguez, S. Donaldson, J. Drenkow, T. R. Dreszer, Y. Drier, M. O. Duff, D. Dunn, C. Eastman, J. R. Ecker, M. D. Edwards, N. El-Ali, S. I. Elhajjajy, K. Elkins, A. Emili, C. B. Epstein, R. C. Evans, I. Ezkurdia, K. Fan, P. J. Farnham, N. P. Farrell, E. A. Feingold, A.-M. Ferreira, K. Fisher-Aylor, S. Fitzgerald, P. Flicek, C. S. Foo, K. Fortier, A. Frankish, P. Freese, S. Fu, X.-D. Fu, Y. Fu, Y. Fukuda-Yuzawa, M. Fulciniti, A. P. W. Funnell, I. Gabdank, T. Galeev, M. Gao, C. G. Giron, T. H. Garvin, C. A. Gelboin-Burkhart, G. Georgolopoulos, M. B. Gerstein, B. M. Giardine, D. K. Gifford, D. M. Gilbert, D. A. Gilchrist, S. Gillespie, T. R. Gingeras, P. Gong, A. Gonzalez, J. M. Gonzalez, P. Good, A. Goren, D. U. Gorkin, B. R. Graveley, M. Gray, J. F. Greenblatt, E. Griffiths, M. T. Groudine, F. Grubert, M. Gu, R. Guigó, H. Guo, Y. Guo, Y. Guo, G. Gursoy, M. Gutierrez-Arcelus, J. Halow, R. C. Hardison, M. Hardy, M. Hariharan, A. Harmanci, A. Harrington, J. L. Harrow, T. B. Hashimoto, R. D. Hasz, M. Hatan, E. Haugen, J. E. Hayes, P. He, Y. He, N. Heidari, D. Hendrickson, E. F. Heuston, J. A. Hilton, B. C. Hitz, A. Hochman, C. Holgren, L. Hou, S. Hou, Y.-H. E. Hsiao, S. Hsu, H. Huang, T. J. Hubbard, J. Huey, T. R. Hughes, T. Hunt, S. Ibarrientos, R. Issner, M. Iwata, O. Izuogu, T. Jaakkola, N.

Jameel, C. Jansen, L. Jiang, P. Jiang, A. Johnson, R. Johnson, I. Jungreis, M. Kadaba, M. Kasowski, M. Kasparian, M. Kato, R. Kaul, T. Kawli, M. Kay, J. C. Keen, S. Keles, C. A. Keller, D. Kelley, M. Kellis, P. Kheradpour, D. S. Kim, A. Kirilusha, R. J. Klein, B. Knoechel, S. Kuan, M. J. Kulik, S. Kumar, A. Kundaje, T. Kutyavin, J. Lagarde, B. R. Lajoie, N. J. Lambert, J. Lazar, A. Y. Lee, D. Lee, E. Lee, J. W. Lee, K. Lee, C. S. Leslie, S. Levy, B. Li, H. Li, N. Li, S. Li, X. Li, Y. I. Li, Y. Li, Y. Li, Y. Li, J. Lian, M. W. Libbrecht, S. Lin, Y. Lin, D. Liu, J. Liu, P. Liu, T. Liu, X. S. Liu, Y. Liu, Y. Liu, M. Long, S. Lou, J. Loveland, A. Lu, Y. Lu, E. Lécuyer, L. Ma, M. Mackiewicz, B. J. Mannion, M. Mannstadt, D. Manthravadi, G. K. Marinov, F. J. Martin, E. Mattei, K. McCue, M. McEown, G. McVicker, S. K. Meadows, A. Meissner, E. M. Mendenhall, C. L. Messer, W. Meuleman, C. Meyer, S. Miller, M. G. Milton, T. Mishra, D. E. Moore, H. M. Moore, J. E. Moore, S. H. Moore, J. Moran, A. Mortazavi, J. M. Mudge, N. Munshi, R. Murad, R. M. Myers, V. Nandakumar, P. Nandi, A. M. Narasimha, A. K. Narayanan, H. Naughton, F. C. P. Navarro, P. Navas, J. Nazarovs, J. Nelson, S. Neph, F. J. Neri, J. R. Nery, A. R. Nesmith, J. S. Newberry, K. M. Newberry, V. Ngo, R. Nguyen, T. B. Nguyen, T. Nguyen, A. Nishida, W. S. Noble, C. S. Novak, E. M. Novoa, B. Nuñez, C. W. O'Donnell, S. Olson, K. C. Onate, E. Otterman, H. Ozadam, M. Pagan, T. Palden, X. Pan, Y. Park, E. C. Partridge, B. Paten, F. Pauli-Behn, M. J. Pazin, B. Pei, L. A. Pennacchio, A. R. Perez, E. H. Perry, D. D. Pervouchine, N. N. Phalke, Q. Pham, D. H. Phanstiel, I. Plajzer-Frick, G. A. Pratt, H. E. Pratt, S. Preissl, J. K. Pritchard, Y. Pritykin, M. J. Purcaro, Q. Qin, G. Quinones-Valdez, I. Rabano, E. Radovani, A. Raj, N. Rajagopal, O. Ram, L. Ramirez, R. N. Ramirez, D. Rausch, S. Raychaudhuri, J. Raymond, R. Razavi, T. E. Reddy, T. M. Reimonn, B. Ren, A. Reymond, A. Reynolds, S. K. Rhie, J. Rinn, M. Rivera, J. C. Rivera-Mulia, B. S. Roberts, J. M. Rodriguez, J. Rozowsky, R. Ryan, E. Rynes, D. N. Salins, R. Sandstrom, T. Sasaki, S. Sathe, D. Savic, A. Scavelli, J. Scheiman, C. Schlaffner, J. A. Schloss, F. W. Schmitges, L. H. See, A. Sethi, M. Setty, A. Shafer, S. Shan, E. Sharon, Q. Shen, Y. Shen, R. I. Sherwood, M. Shi, S. Shin, N. Shoresh, K. Siebenthall, C. Sisu, T. Slifer, C. A. Sloan, A. Smith, V. Snetkova, M. P. Snyder, D. V. Spacek, S. Srinivasan, R. Srivas, G. Stamatoyannopoulos, J. A. Stamatoyannopoulos, R. Stanton, D. Steffan, S. Stehling-Sun, J. S. Strattan, A. Su, B. Sundararaman, M.-M. Suner, T. Syed, M. Szynkarek, F. Y. Tanaka, D. Tenen, M. Teng, J. A. Thomas, D. Toffey, M. L. Tress, D. E. Trout, G. Trynka, J. Tsuji, S. A. Upchurch, O. Ursu, B. Uszczynska-Ratajczak, M. C. Uziel, A. Valencia, B. V. Biber, A. G. van der Velde, E. L. V. Nostrand, Y. Vaydylevich, J. Vazquez, A. Victorsen, J. Vielmetter, J. Vierstra, A. Visel, A. Vlasova, C. M. Vockley, S. Volpi, S. Vong, H. Wang, M. Wang, Q. Wang, R. Wang, T. Wang, W. Wang, X. Wang, Y. Wang, N. K. Watson, X. Wei, Z. Wei, H. Weisser, S. M. Weissman, R. Welch, R. E. Welikson, Z. Weng, H.-J. Westra, J. W. Whitaker, C. White, K. P. White, A. Wildberg, B. A. Williams, D. Wine, H. N. Witt, B. Wold, M. Wolf, J. Wright, R. Xiao, X. Xiao, J. Xu, J. Xu, K.-K. Yan, Y. Yan, H. Yang, X. Yang, Y.-W. Yang, G. G. Yardımcı, B. A. Yee, G. W. Yeo, T. Young, T. Yu, F. Yue, C. Zaleski, C. Zang, H. Zeng, W. Zeng, D. R. Zerbino, J. Zhai, L. Zhan, Y. Zhan, B. Zhang, J. Zhang, J. Zhang, K. Zhang, L. Zhang, P. Zhang, Q. Zhang, X.-O. Zhang, Y. Zhang, Z. Zhang, Y. Zhao, Y. Zheng, G. Zhong, X.-Q. Zhou, Y. Zhu, J. Zimmerman, J. E. Moore, M. J. Purcaro, H. E. Pratt, C. B. Epstein, N. Shoresh, J. Adrian, T. Kawli, C. A. Davis, A. Dobin, R. Kaul, J. Halow, E. L. V. Nostrand, P. Freese, D. U. Gorkin, Y. Shen, Y. He, M. Mackiewicz, F. Pauli-Behn, B. A. Williams, A. Mortazavi, C. A. Keller, X.-O. Zhang, S. I. Elhajjajy, J. Huey, D. E. Dickel, V. Snetkova, X. Wei, X. Wang, J. C. Rivera-Mulia, J. Rozowsky, J. Zhang, S. B. Chhetri, J. Zhang, A. Victorsen, K. P. White, A. Visel, G. W. Yeo, C. B. Burge, E. Lécuyer, D. M. Gilbert, J. Dekker, J. Rinn, E. M. Mendenhall, J. R. Ecker, M. Kellis, R. J. Klein, W. S. Noble, A. Kundaje, R. Guigó, P. J. Farnham, J. M. Cherry, R. M. Myers, B. Ren, B. R. Graveley, M. B. Gerstein, L. A. Pennacchio, M. P. Snyder, B. E. Bernstein, B. Wold, R. C. Hardison, T. R. Gingeras, J. A. Stamatoyannopoulos, Z. Weng, Expanded encyclopaedias of DNA elements in the human and mouse genomes. *Nature* **583**, 699–710 (2020).
