# Supplementary material for: Elevated nuclear TDP-43 induces constitutive exon skipping
Source: Mol Neurodegener. 2024 Jun 9;19:45. doi: 10.1186/s13024-024-00732-w (PMC11163724; doi:10.1186/s13024-024-00732-w)
Supplement: Supplementary file 3 — Supplementary Material 3 [file 13024_2024_732_MOESM3_ESM.pdf]

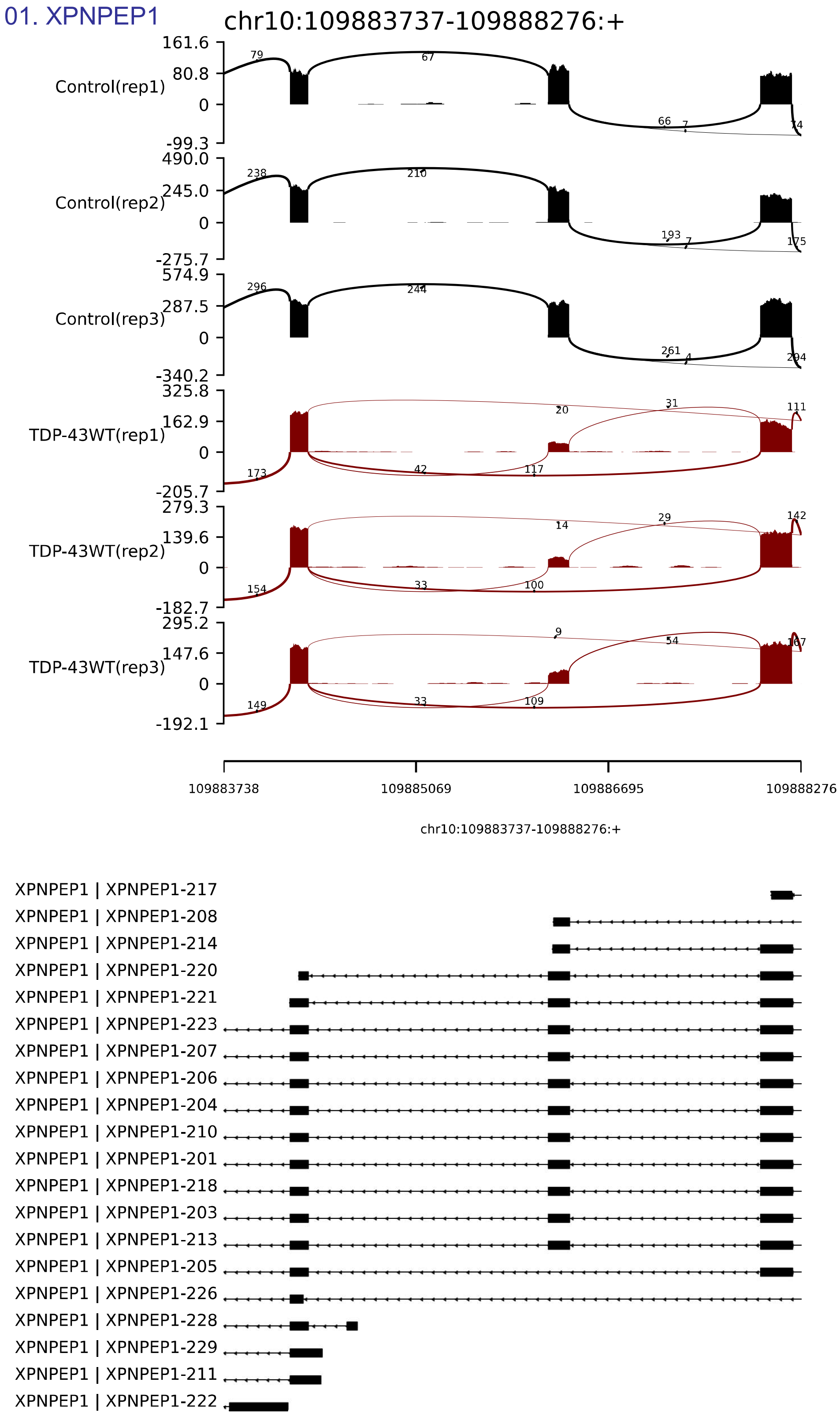
01. XPNPEP1
chr10:109883737-109888276:+
Control(rep1)
Control(rep2)
Control(rep3)
TDP-43WT(rep1)
TDP-43WT(rep2)
TDP-43WT(rep3)
109883738
109885069
109886695
109888276
chr10:109883737-109888276:+
XPNPEP1 | XPNPEP1-217
XPNPEP1 | XPNPEP1-208
XPNPEP1 | XPNPEP1-214
XPNPEP1 | XPNPEP1-220
XPNPEP1 | XPNPEP1-221
XPNPEP1 | XPNPEP1-223
XPNPEP1 | XPNPEP1-207
XPNPEP1 | XPNPEP1-206
XPNPEP1 | XPNPEP1-204
XPNPEP1 | XPNPEP1-210
XPNPEP1 | XPNPEP1-201
XPNPEP1 | XPNPEP1-218
XPNPEP1 | XPNPEP1-203
XPNPEP1 | XPNPEP1-213
XPNPEP1 | XPNPEP1-205
XPNPEP1 | XPNPEP1-226
XPNPEP1 | XPNPEP1-228
XPNPEP1 | XPNPEP1-229
XPNPEP1 | XPNPEP1-211
XPNPEP1 | XPNPEP1-222

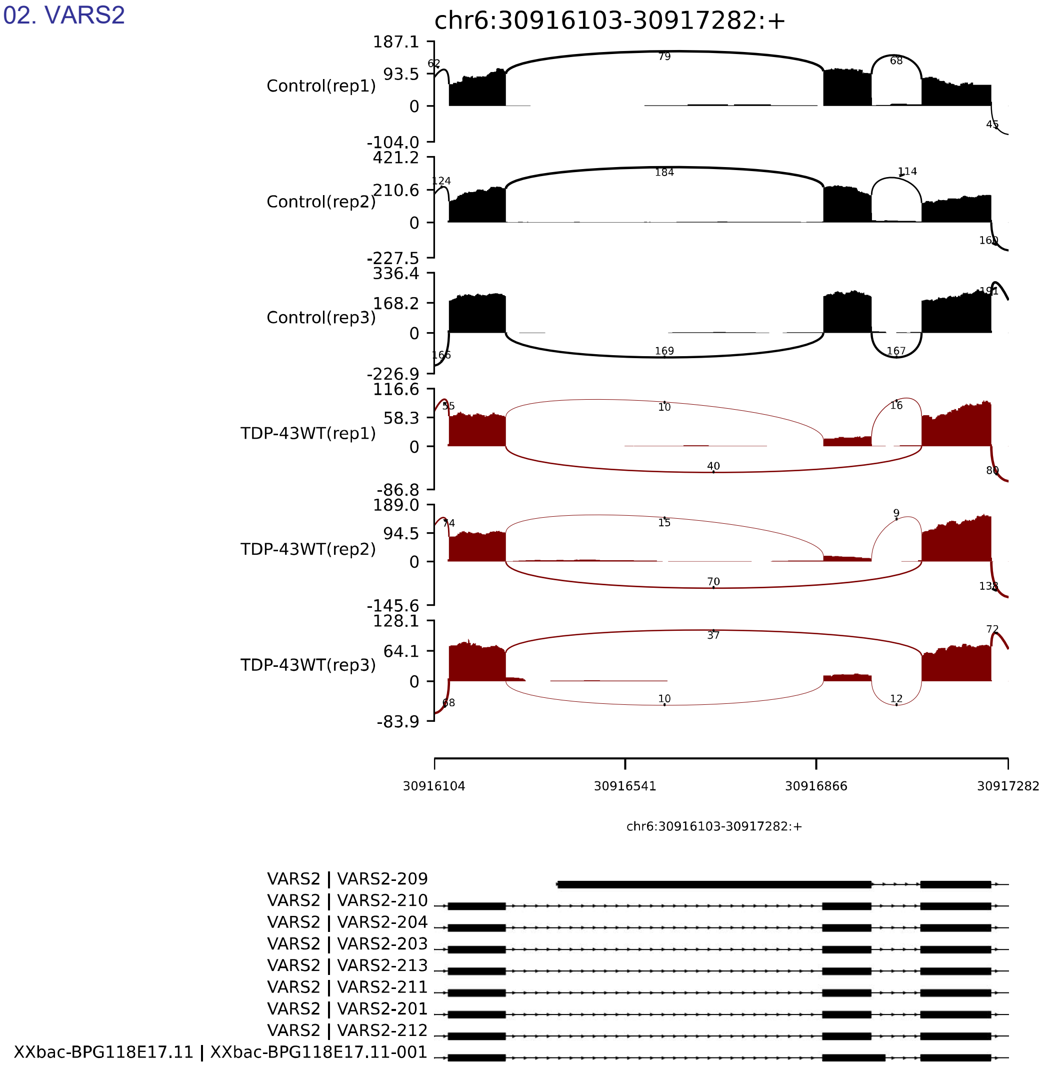
02. VARS2
chr6:30916103-30917282:+
Control(rep1)
Control(rep2)
Control(rep3)
TDP-43WT(rep1)
TDP-43WT(rep2)
TDP-43WT(rep3)
30916104
30916541
30916866
30917282
chr6:30916103-30917282:+
VARS2 | VARS2-209
VARS2 | VARS2-210
VARS2 | VARS2-204
VARS2 | VARS2-203
VARS2 | VARS2-213
VARS2 | VARS2-211
VARS2 | VARS2-201
VARS2 | VARS2-212
XXbac-BPG118E17.11 | XXbac-BPG118E17.11-001

03. SLC35A5

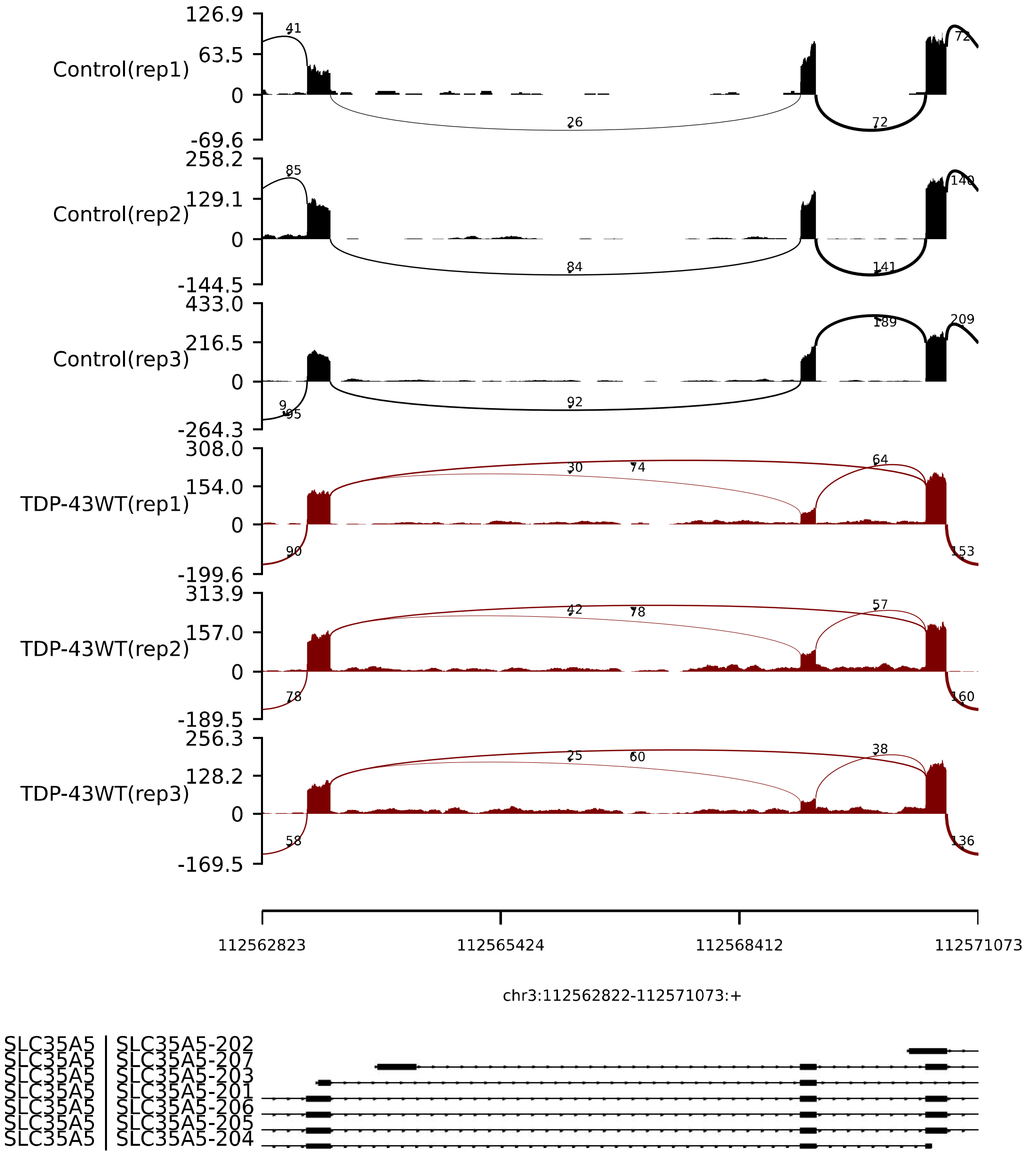

04. SESN3

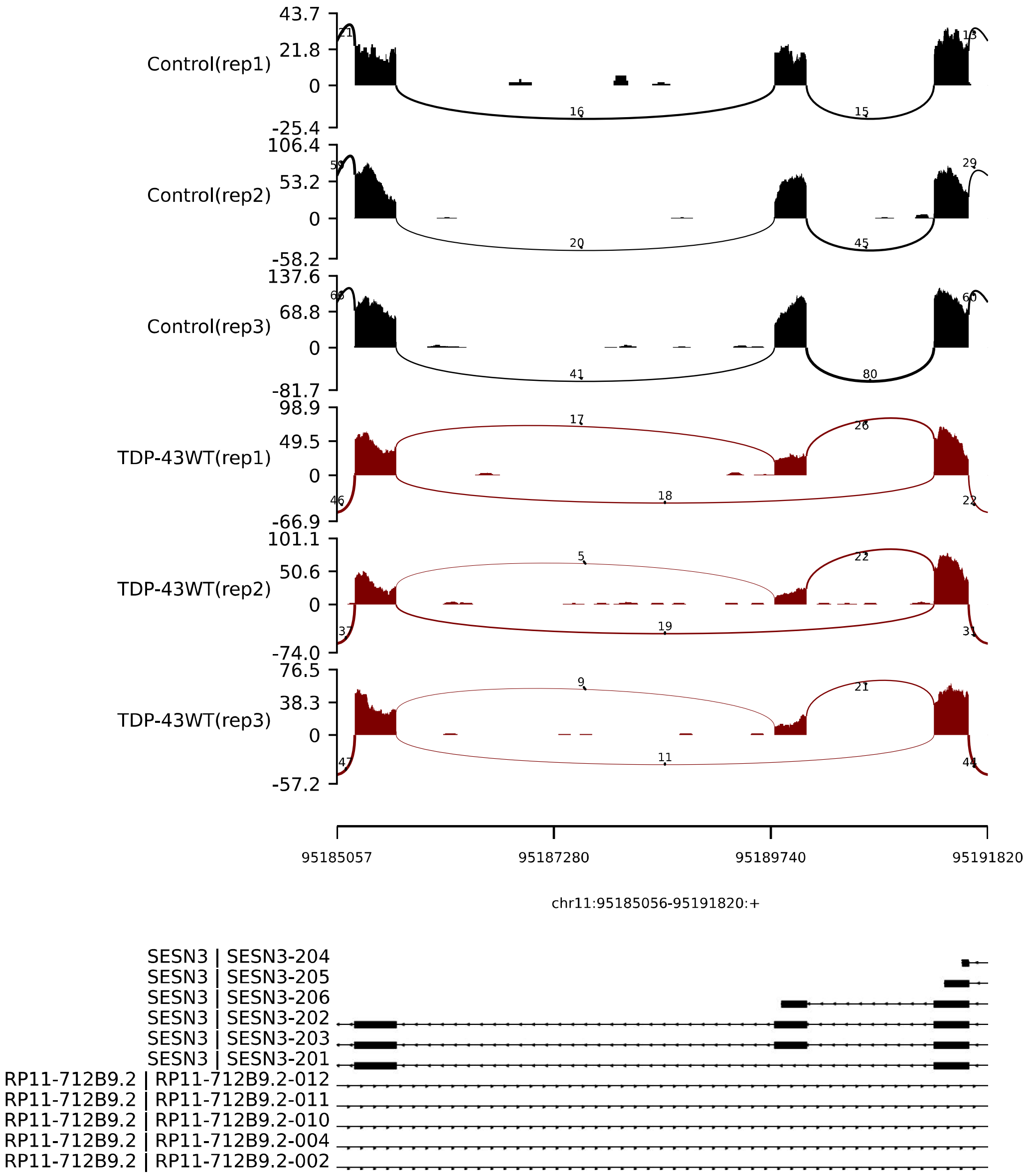

05. SEC61A2

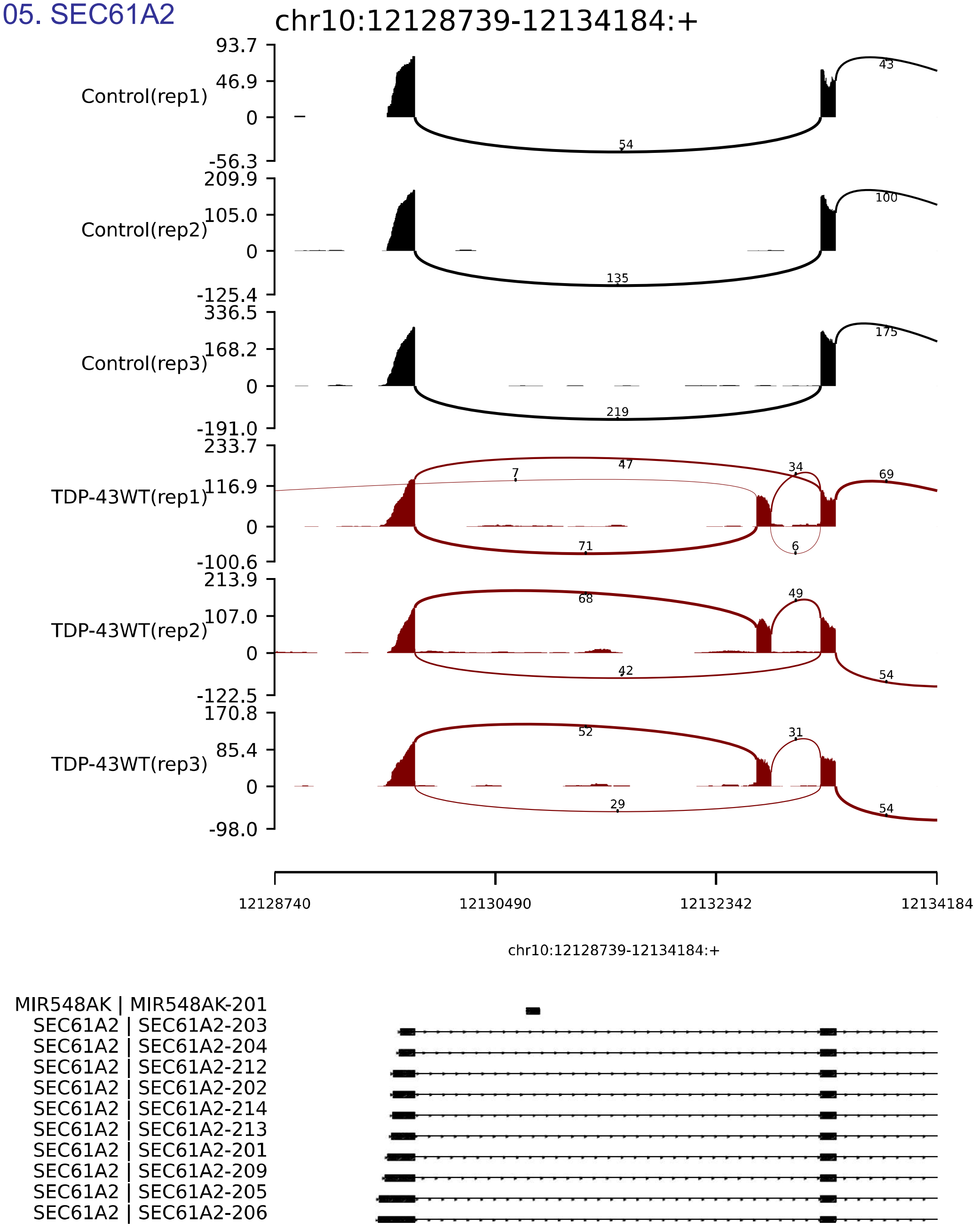

06. SBF1

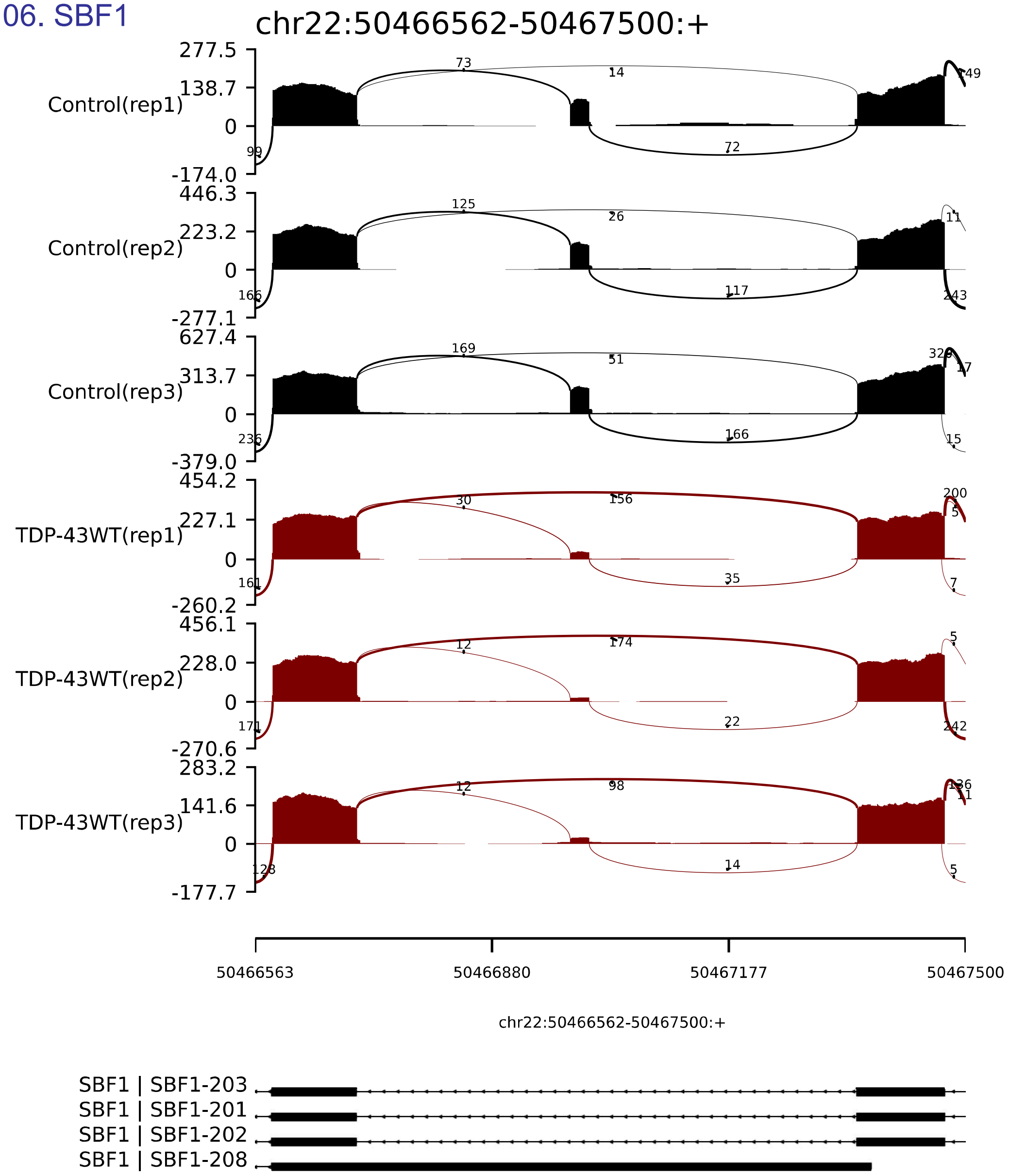

07. RCHY1

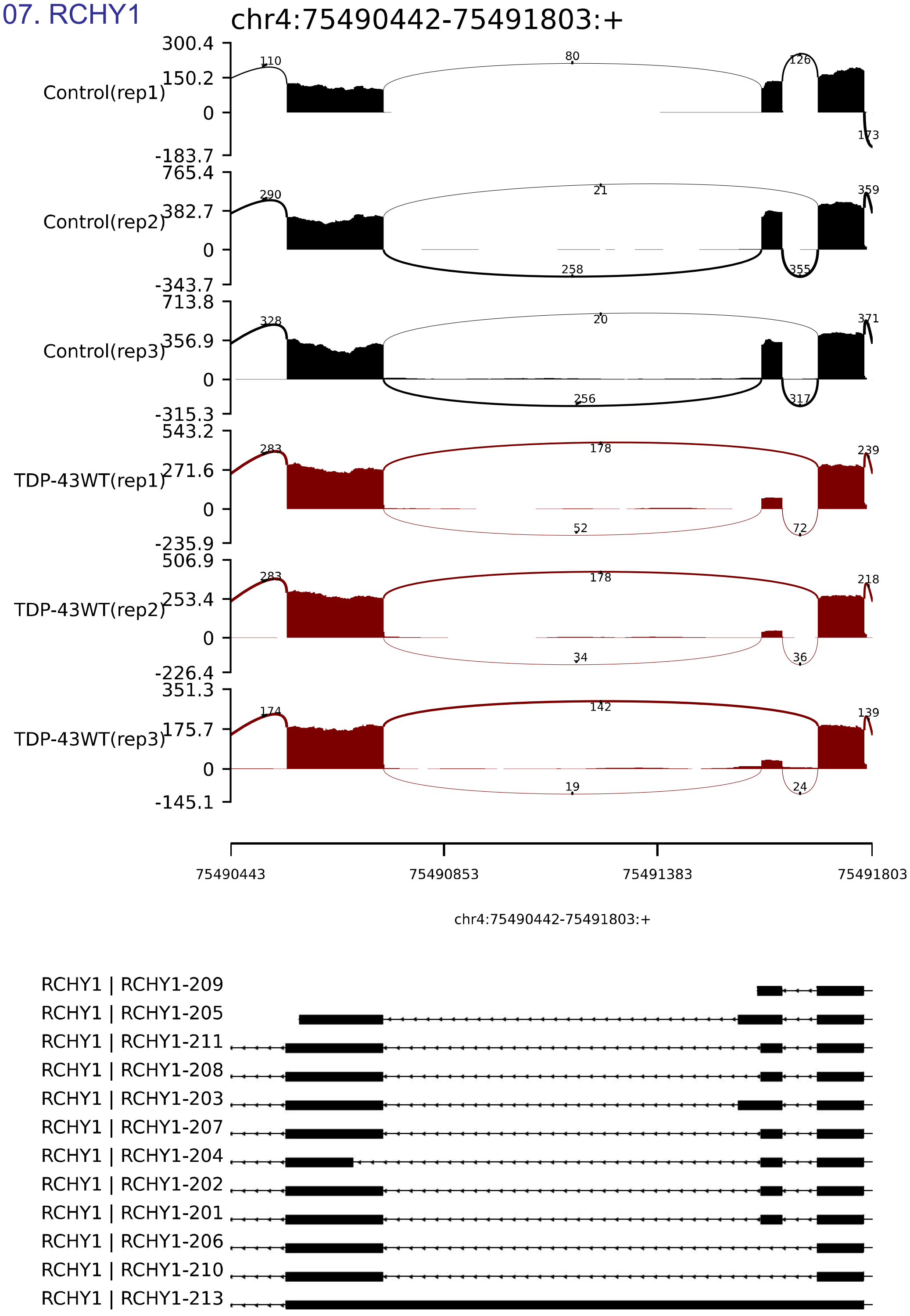

08. RABGGTB

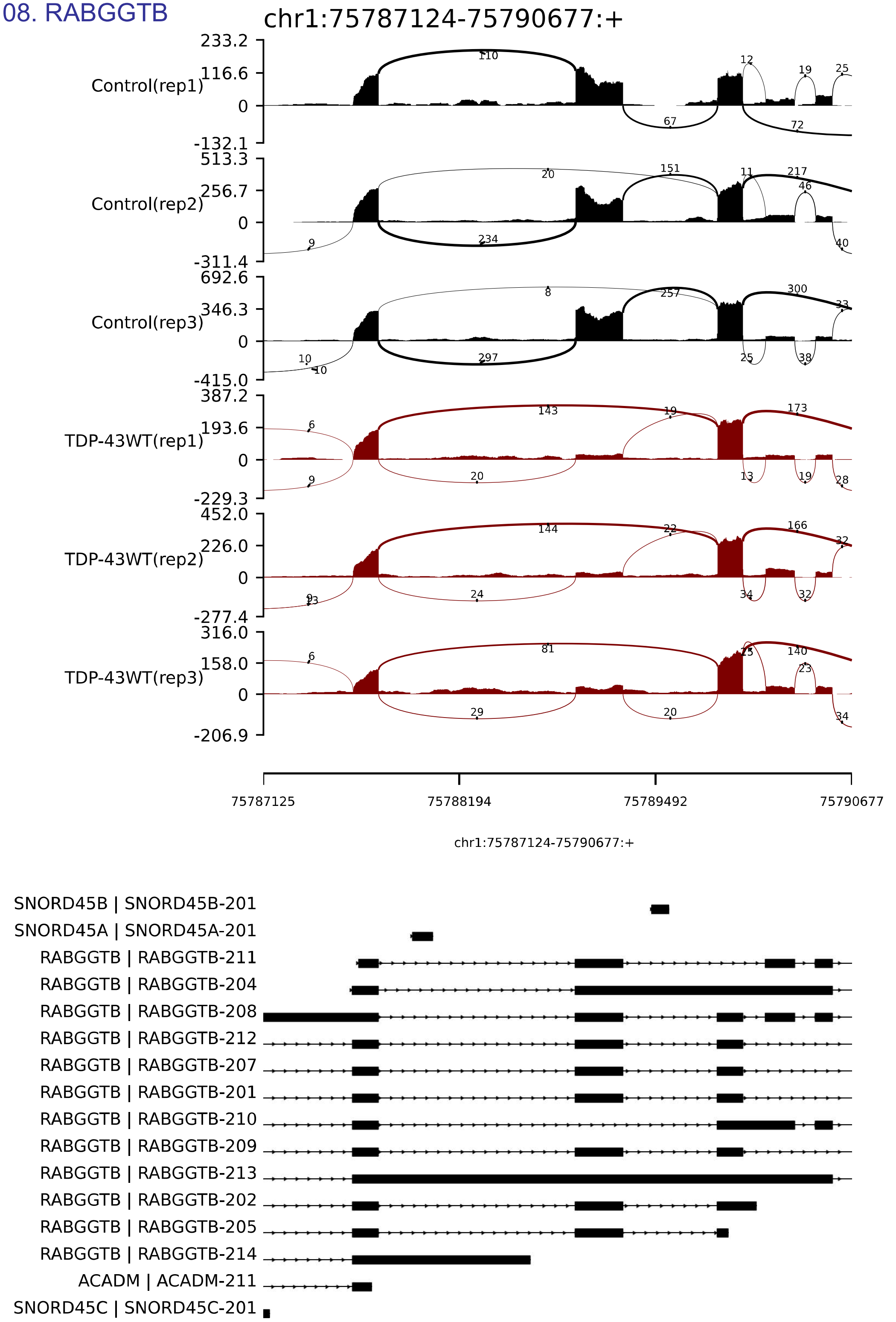

09. PTS

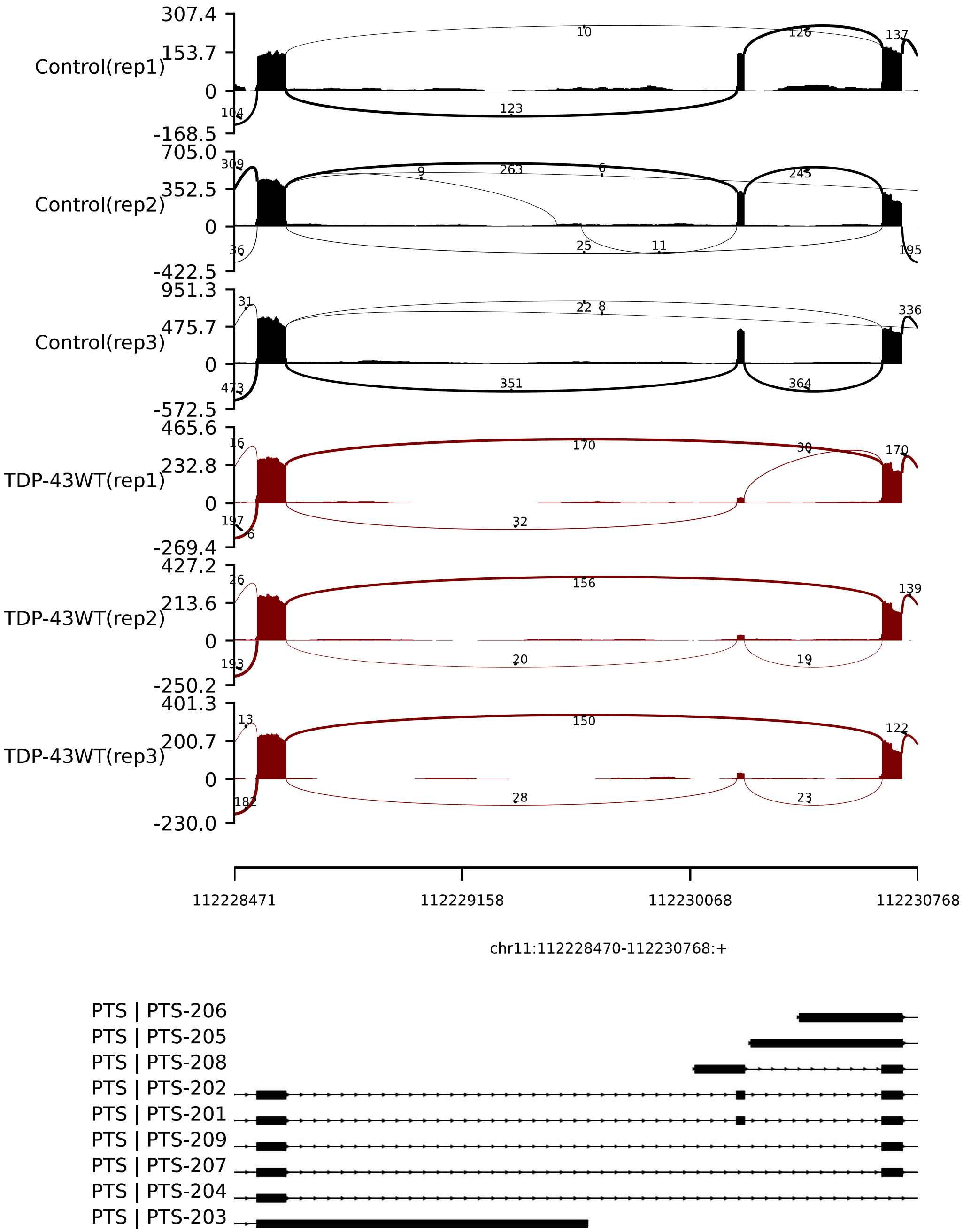

10. PLXNB1

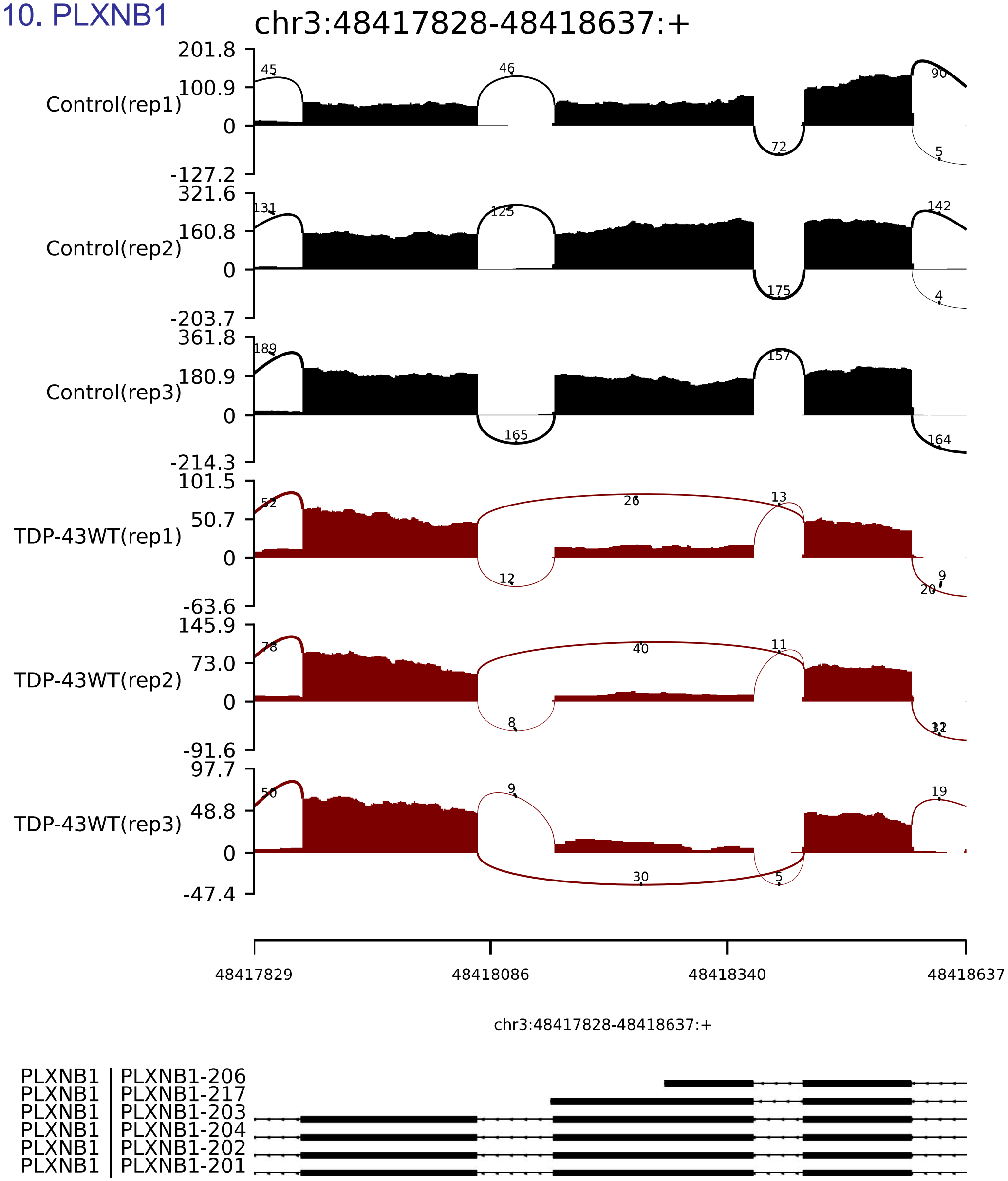

11. NUP93

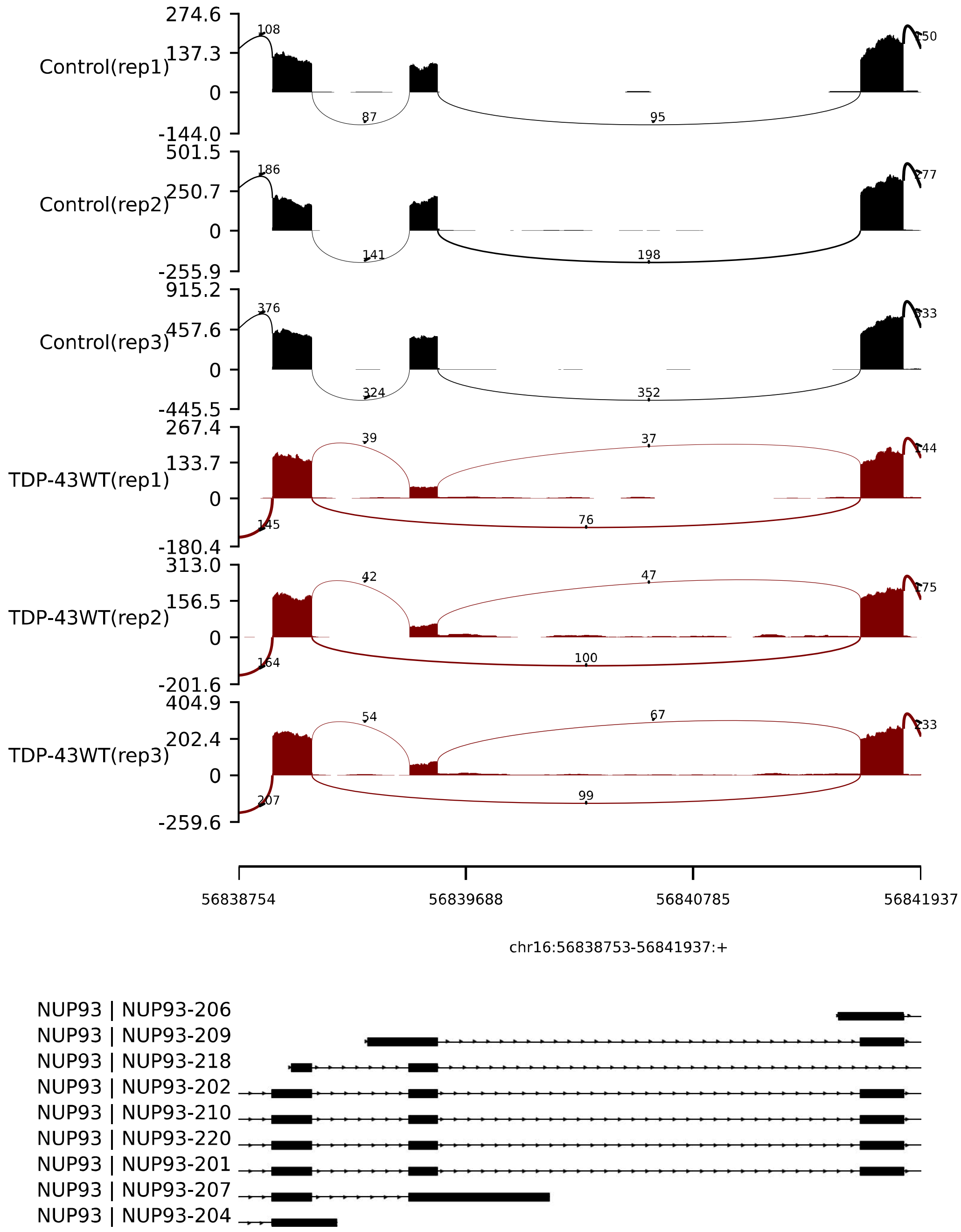

12. NUP88

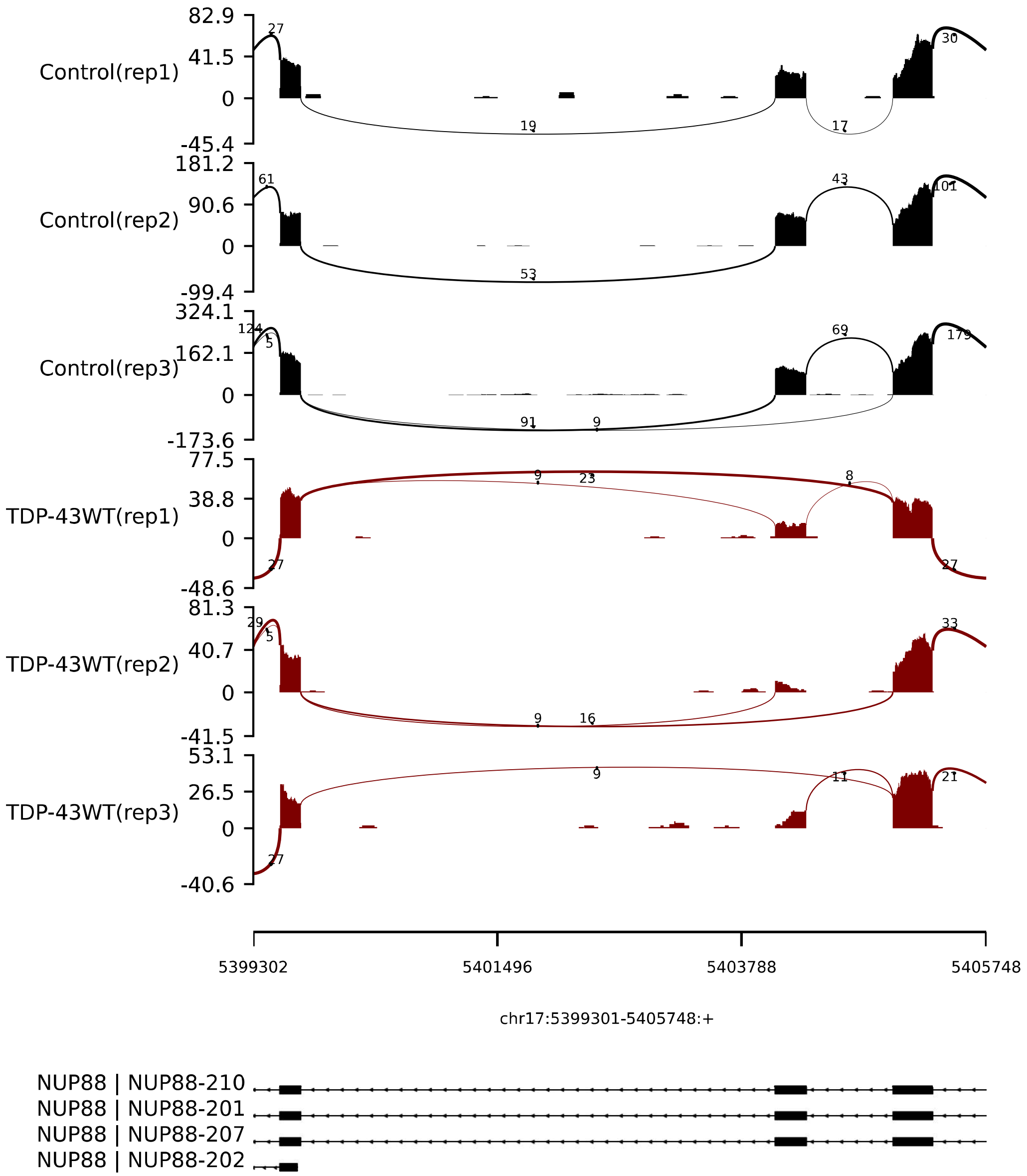

13. NRCAM

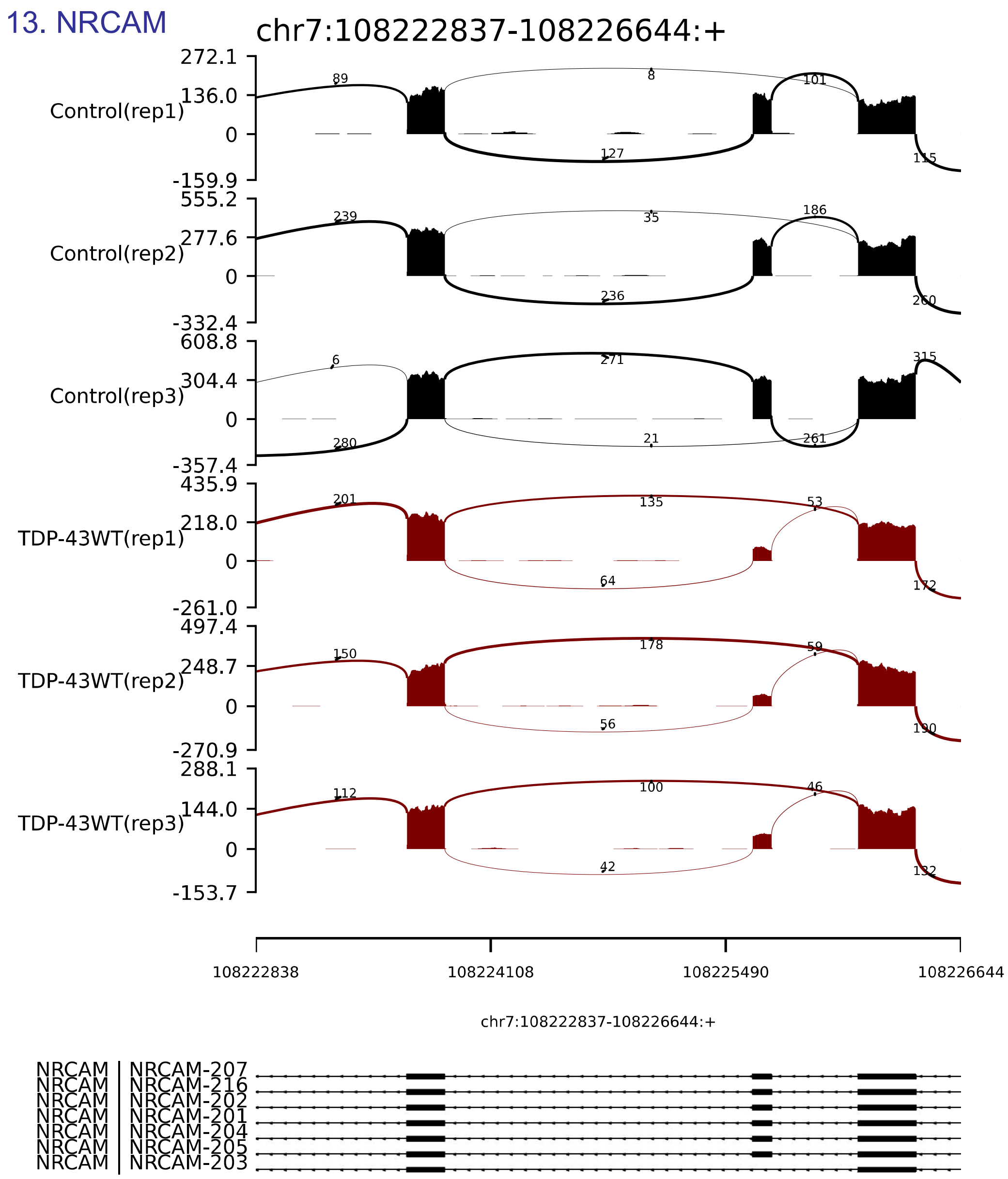

14. NIFK

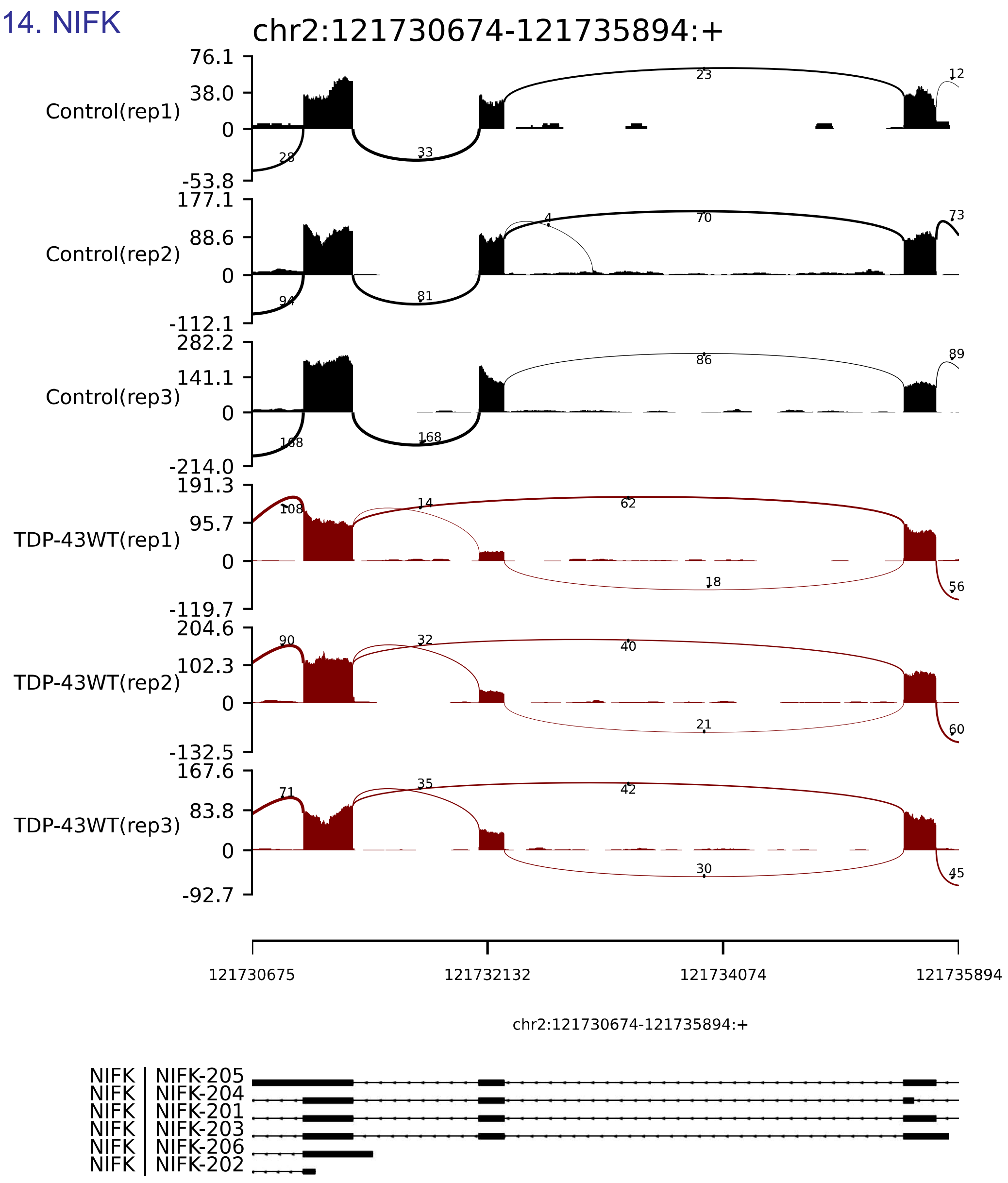

15. MYBBP1A

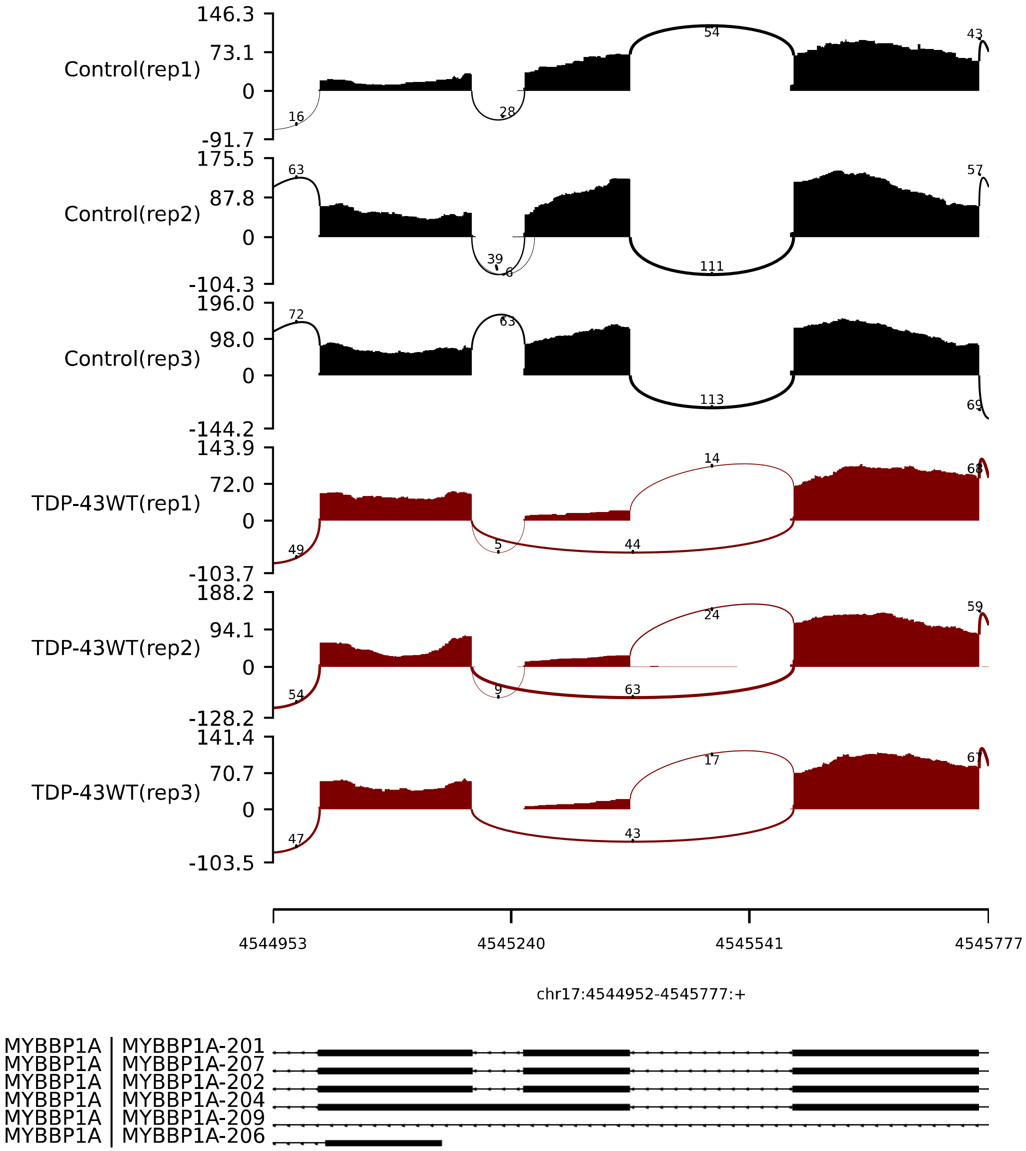

16. HYOU1

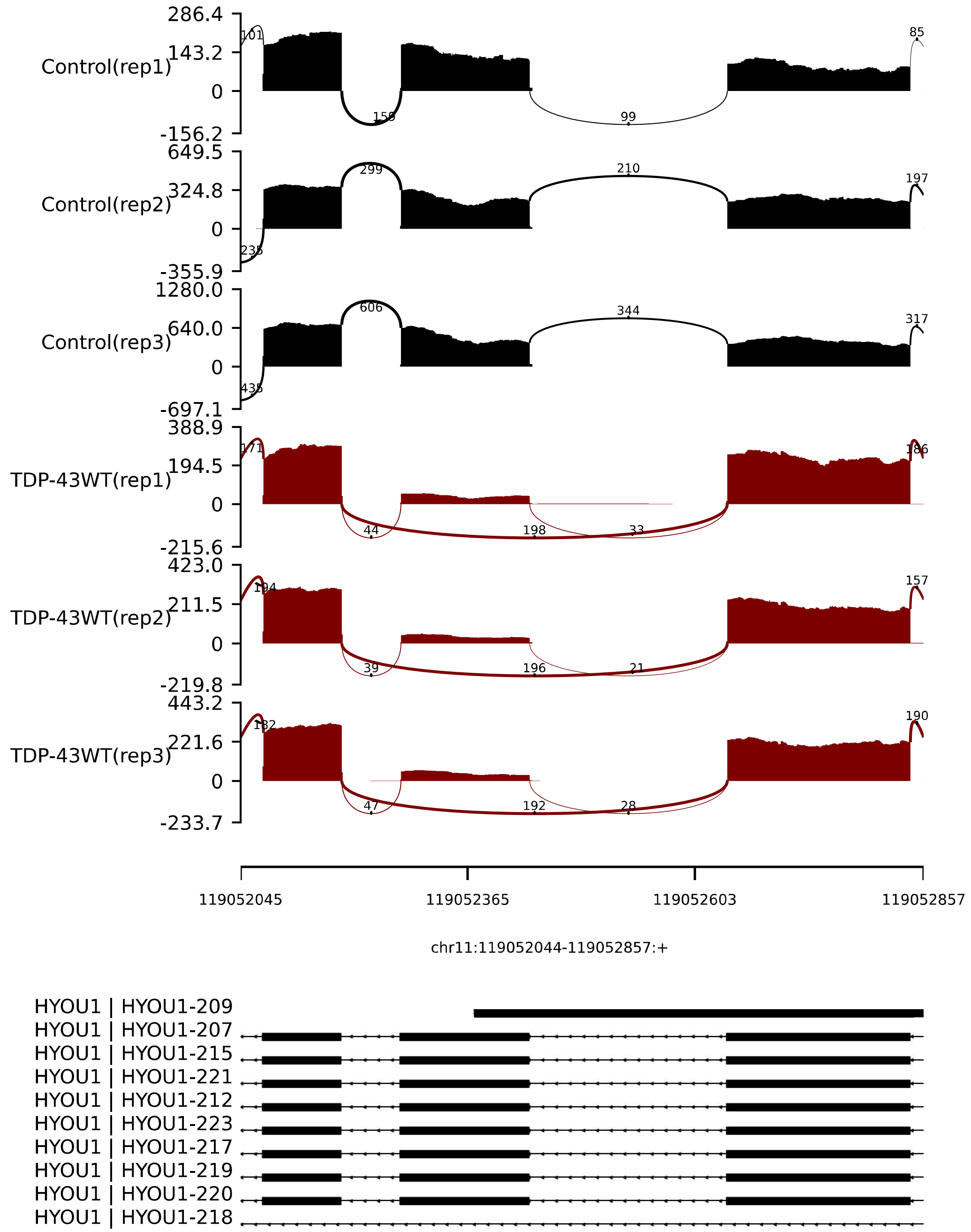

17. GART

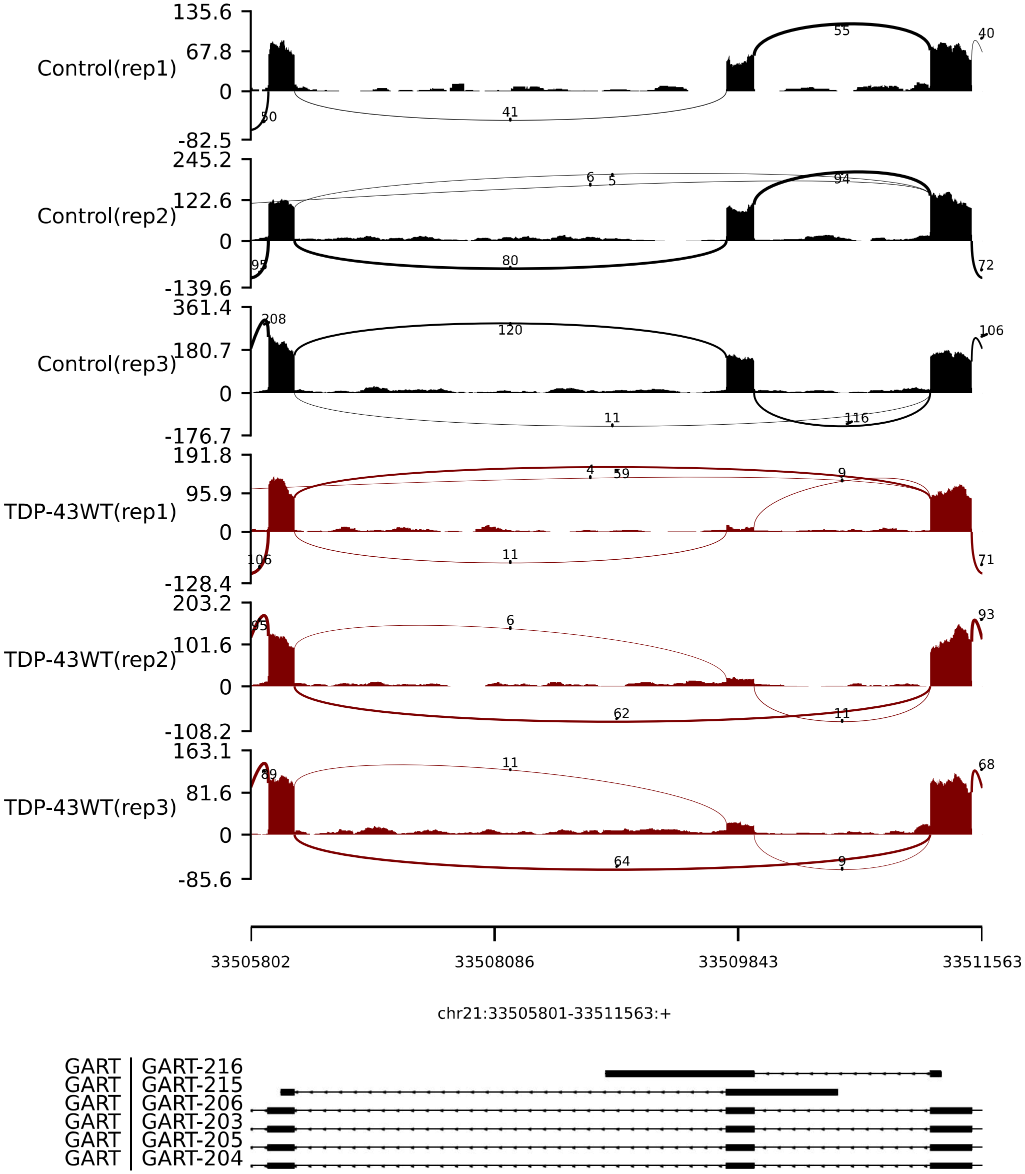

18. FBXO22

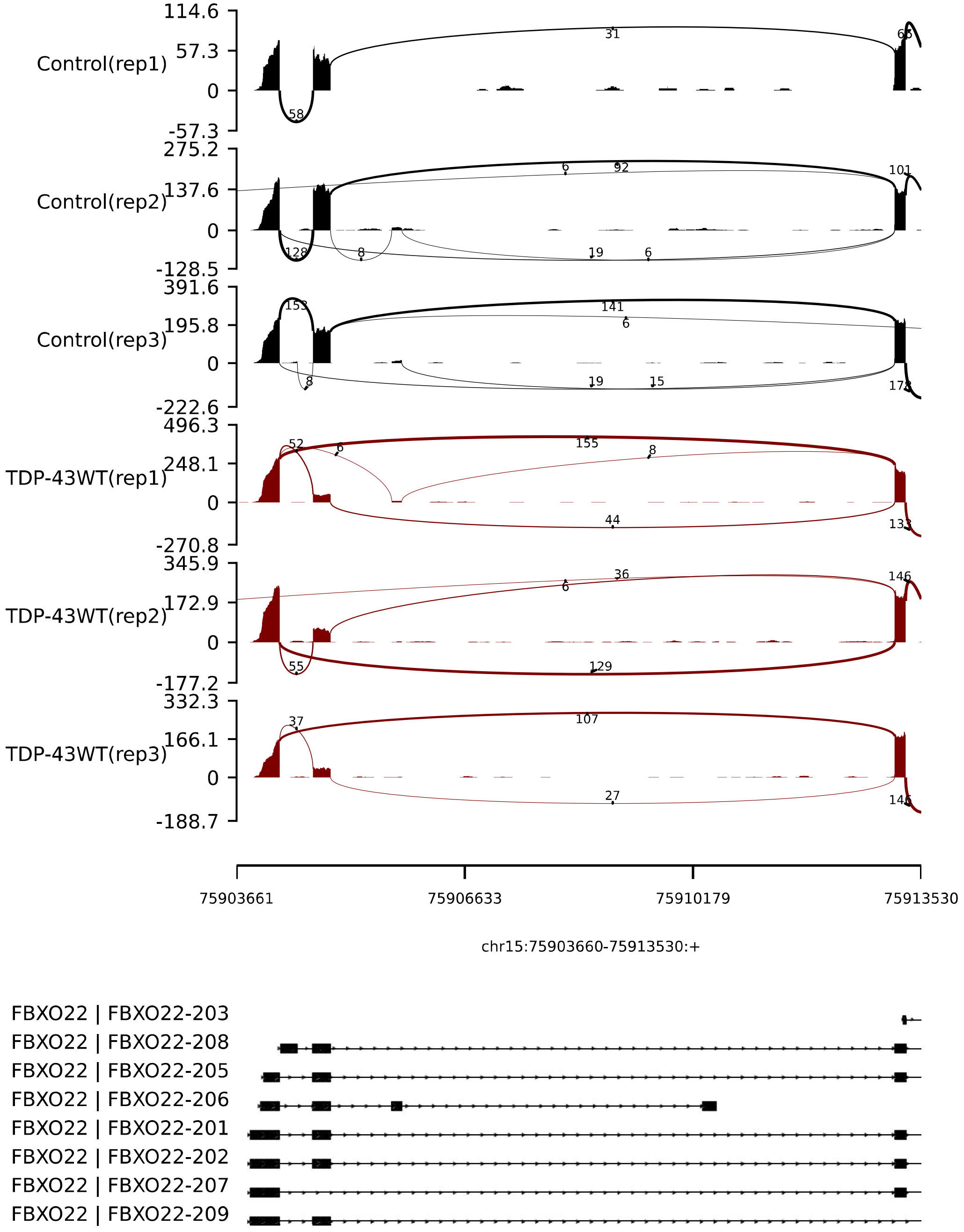

19. ELP2 chr18:36166598-36171437:+

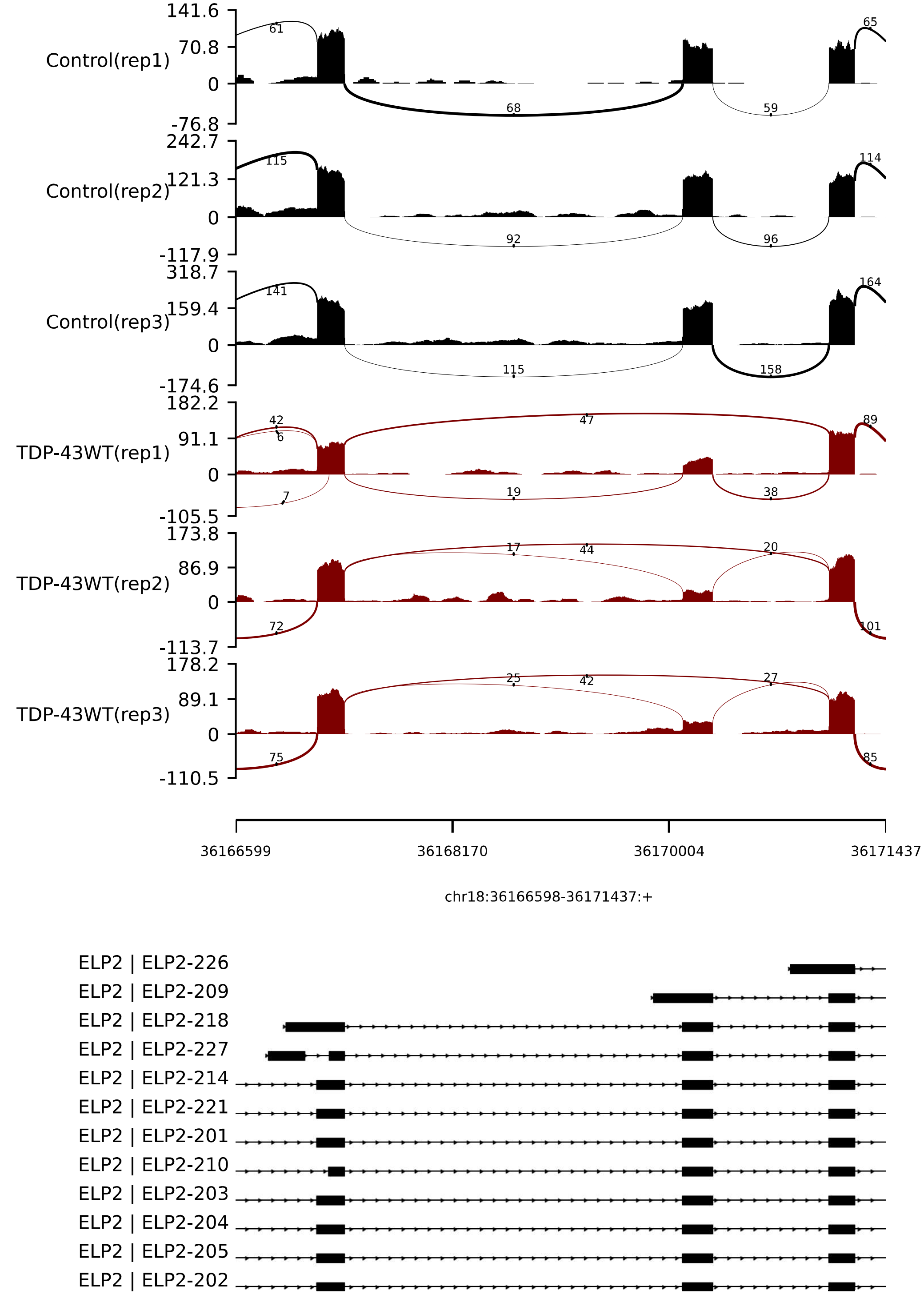

20. CSDE1 chr1:114729970-114733097:+

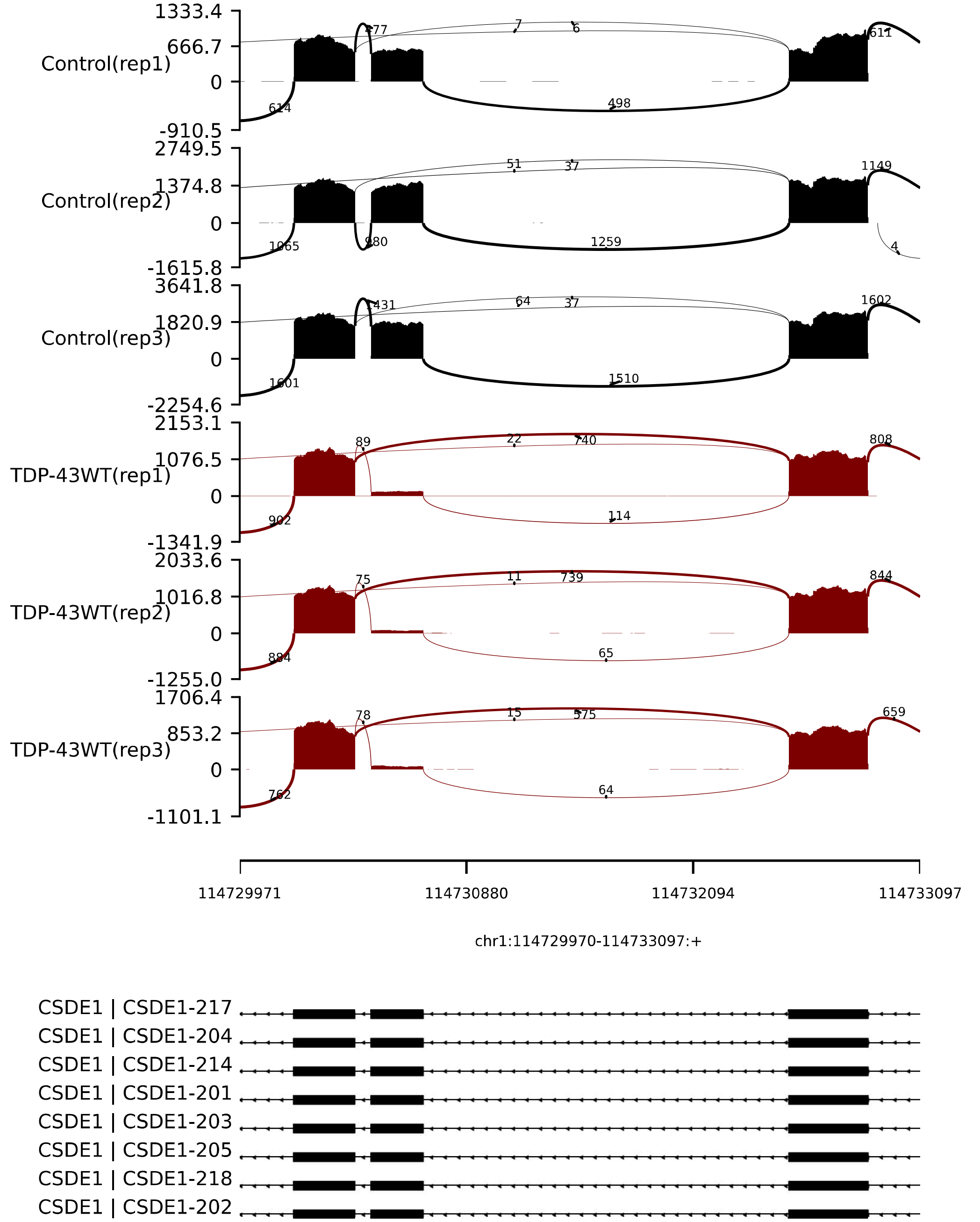

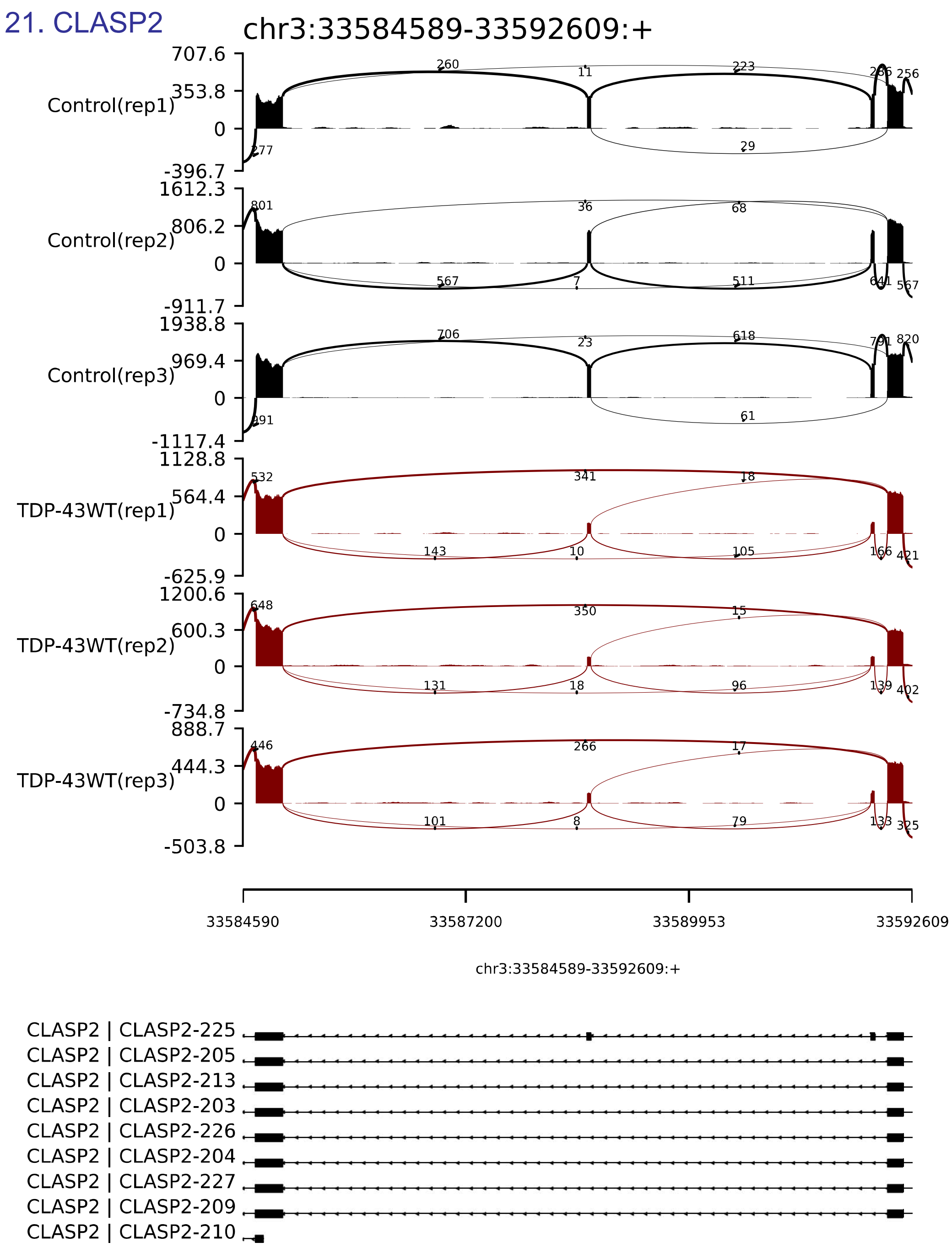
21. CLASP2
chr3:33584589-33592609:+
Control(rep1)
Control(rep2)
Control(rep3)
TDP-43WT(rep1)
TDP-43WT(rep2)
TDP-43WT(rep3)
33584590
33587200
33589953
33592609
chr3:33584589-33592609:+
CLASP2 | CLASP2-225
CLASP2 | CLASP2-205
CLASP2 | CLASP2-213
CLASP2 | CLASP2-203
CLASP2 | CLASP2-226
CLASP2 | CLASP2-204
CLASP2 | CLASP2-227
CLASP2 | CLASP2-209
CLASP2 | CLASP2-210

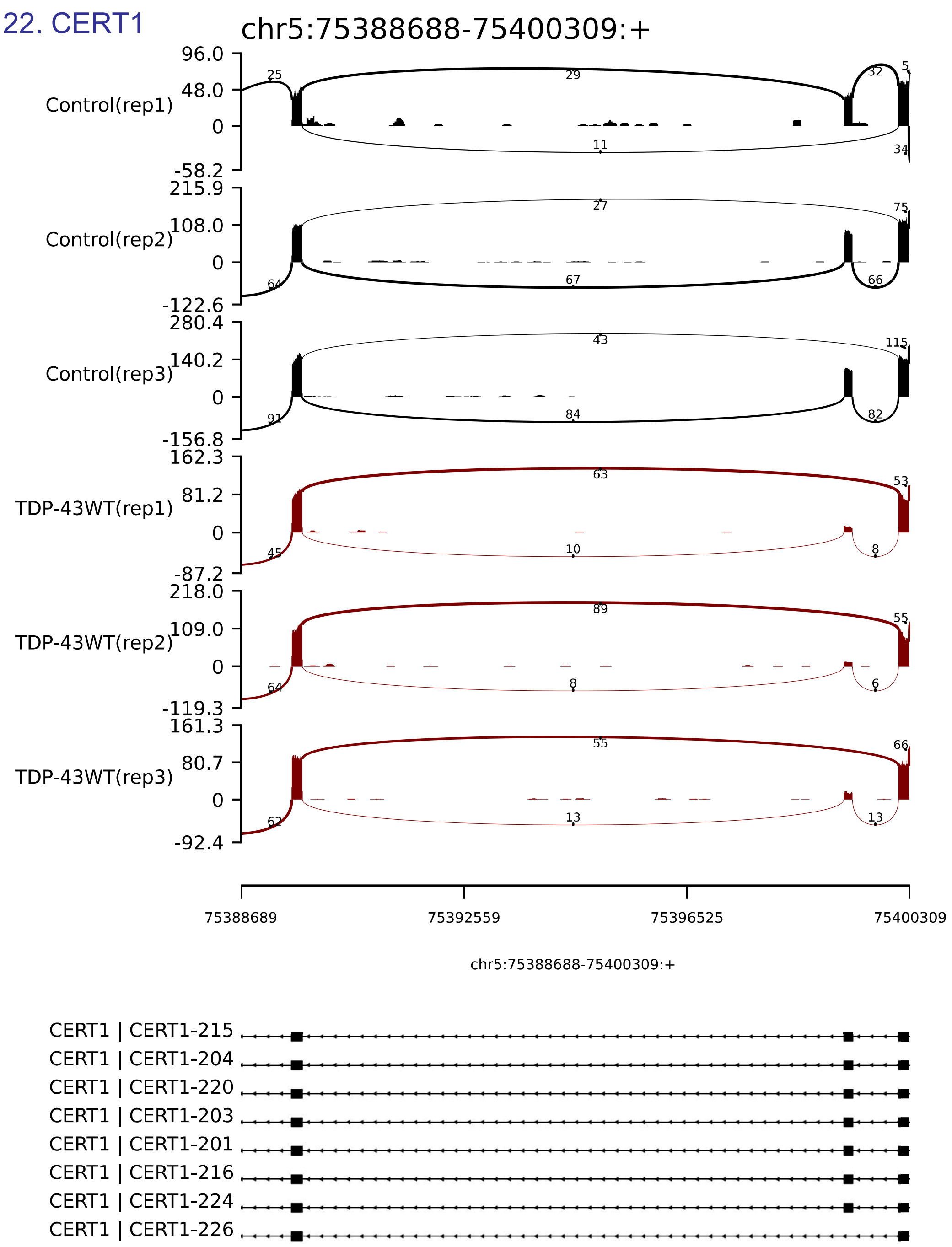
22. CERT1
chr5:75388688-75400309:+
Control(rep1)
Control(rep2)
Control(rep3)
TDP-43WT(rep1)
TDP-43WT(rep2)
TDP-43WT(rep3)
75388689
75392559
75396525
75400309
chr5:75388688-75400309:+
CERT1 | CERT1-215
CERT1 | CERT1-204
CERT1 | CERT1-220
CERT1 | CERT1-203
CERT1 | CERT1-201
CERT1 | CERT1-216
CERT1 | CERT1-224
CERT1 | CERT1-226

23. ZNF767P

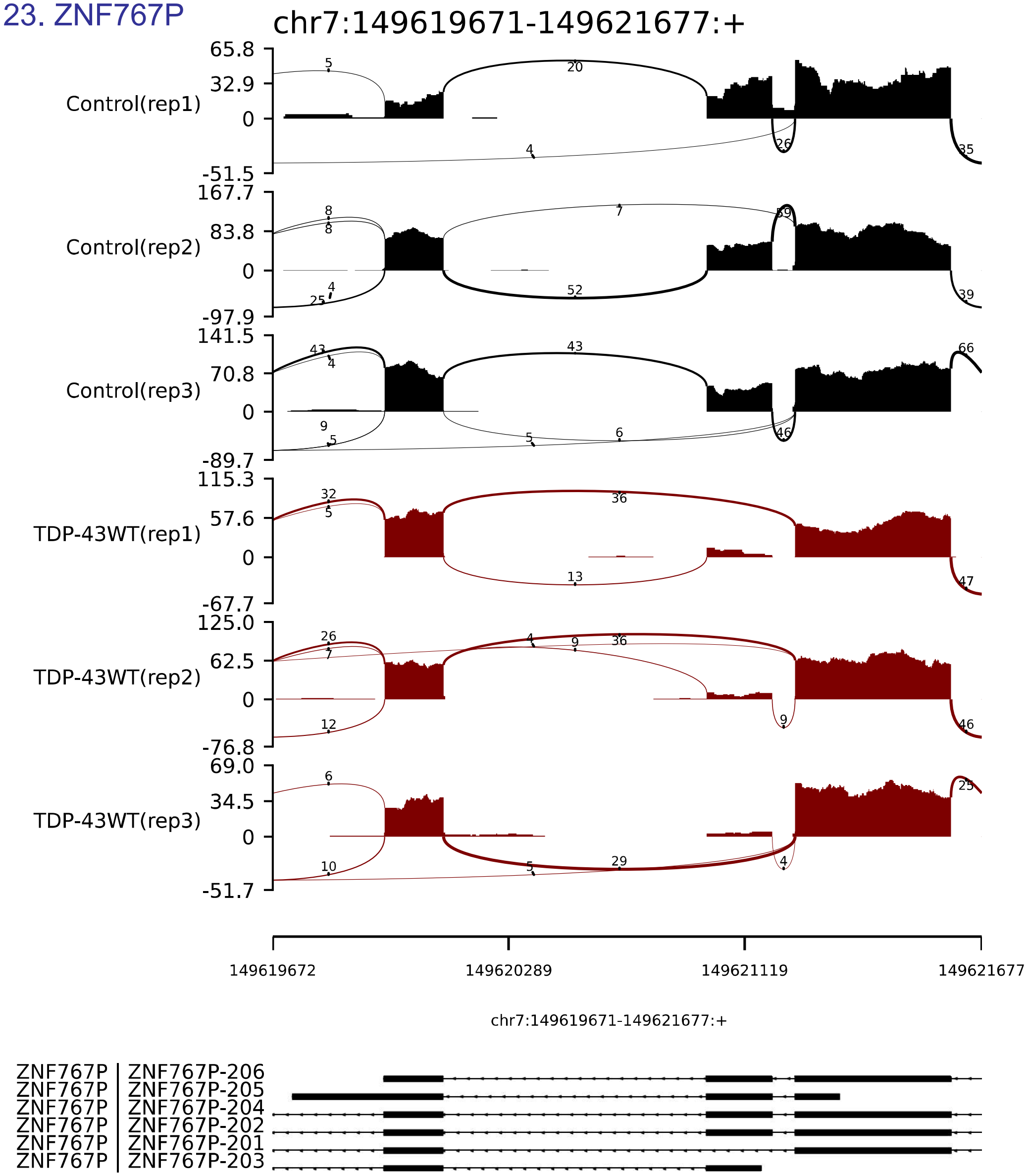

24. ZMYND11

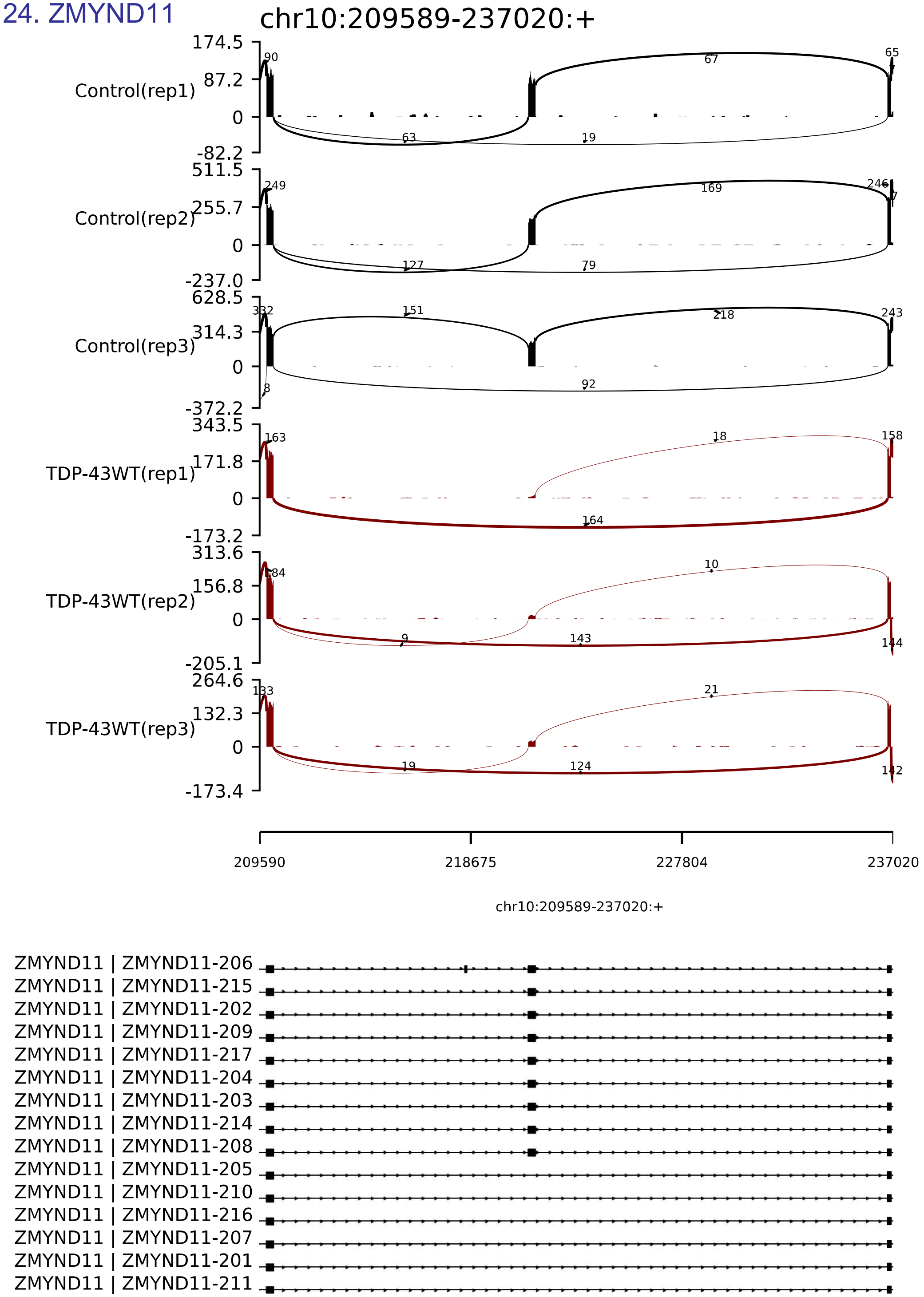

25. WSCD1

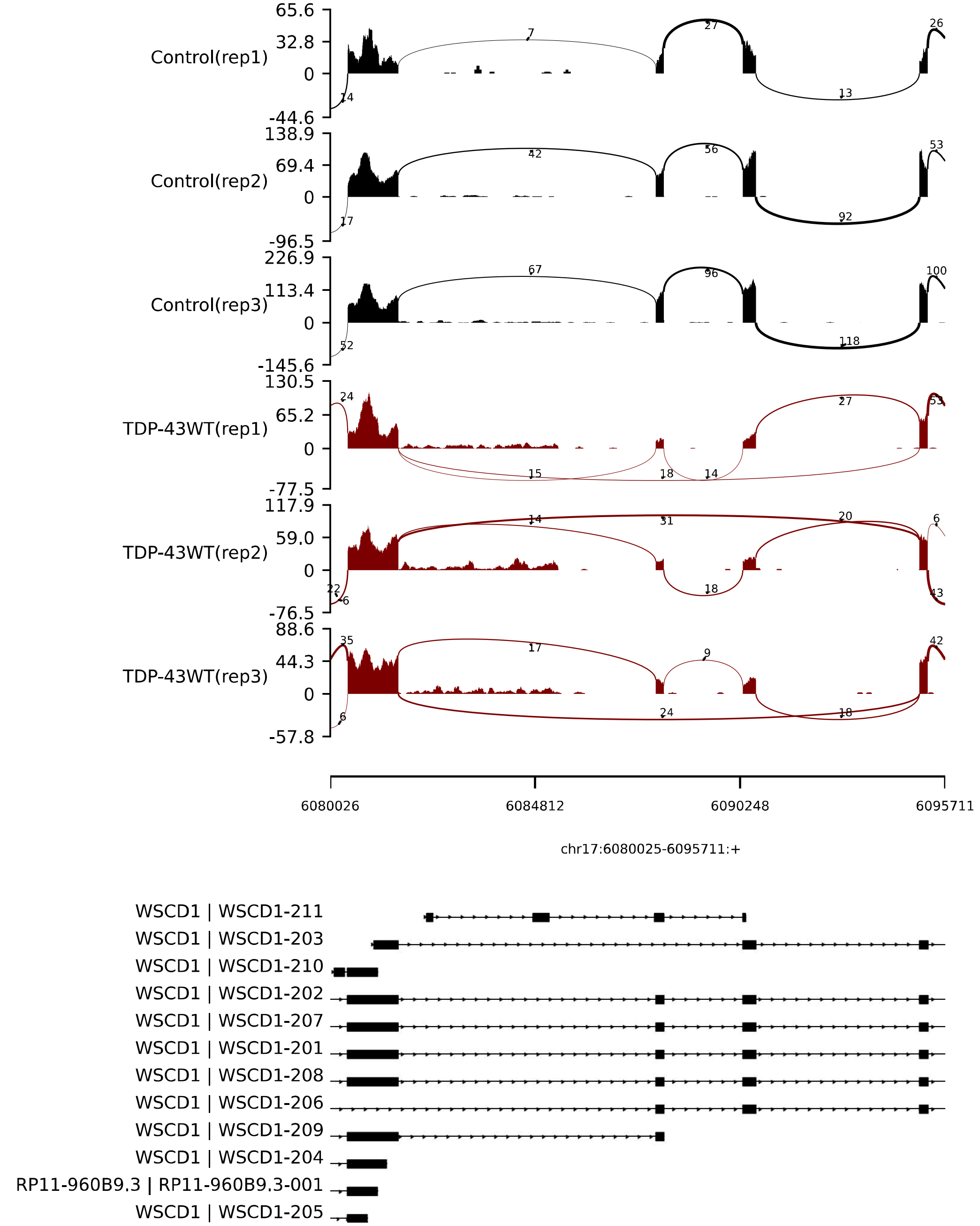

26. WDR41

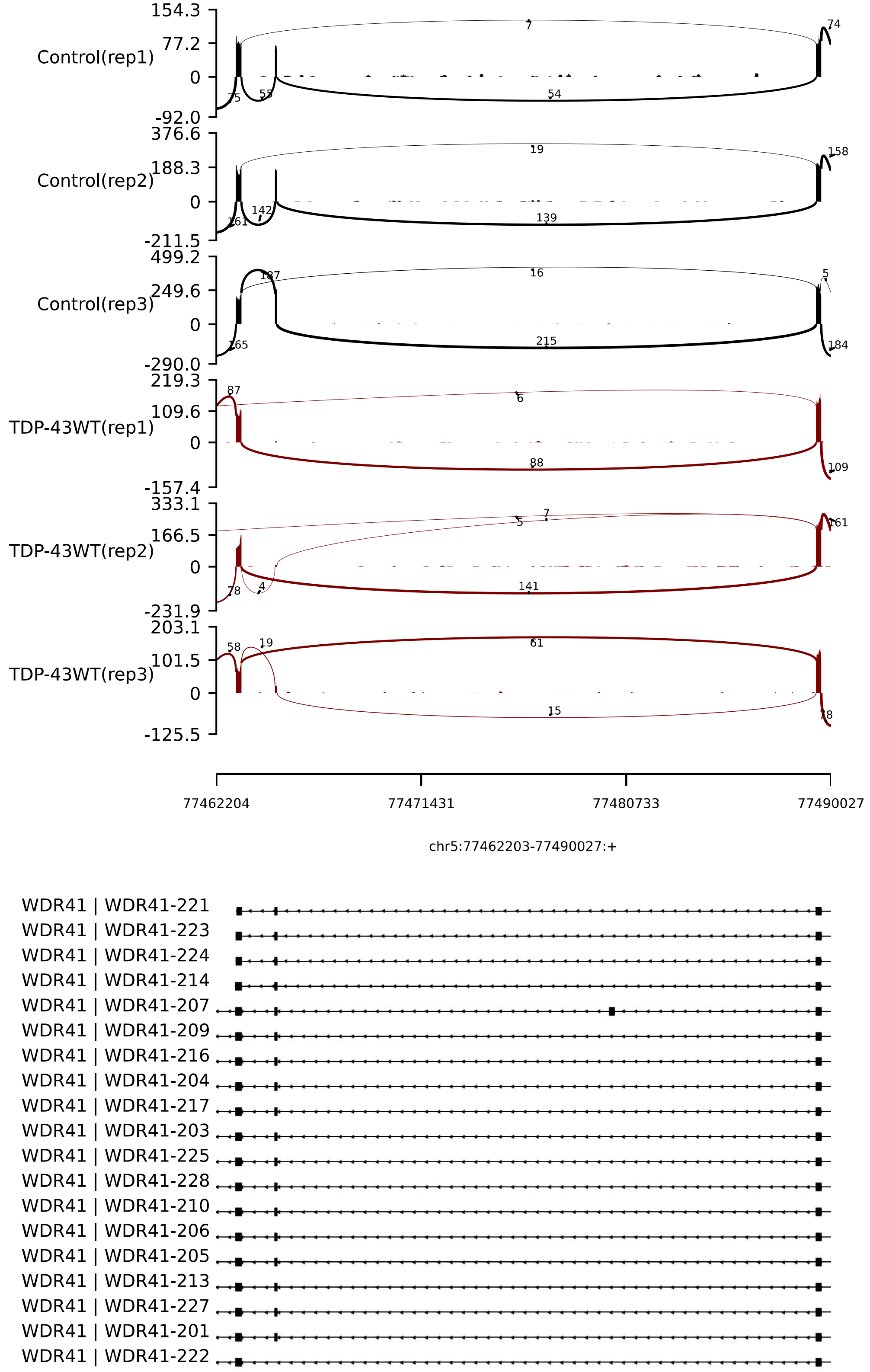

27. TRIM16

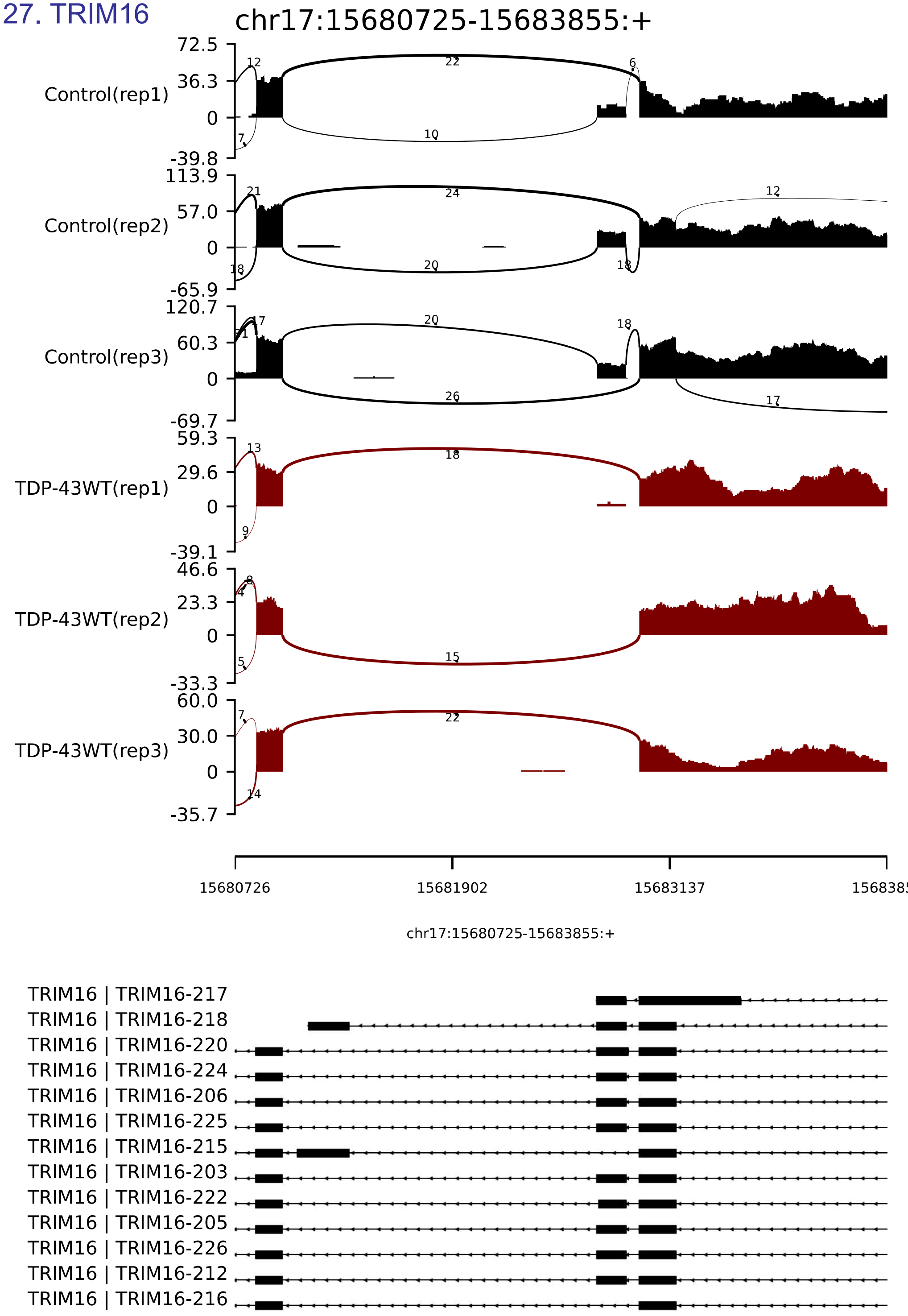

28. TP53BP2

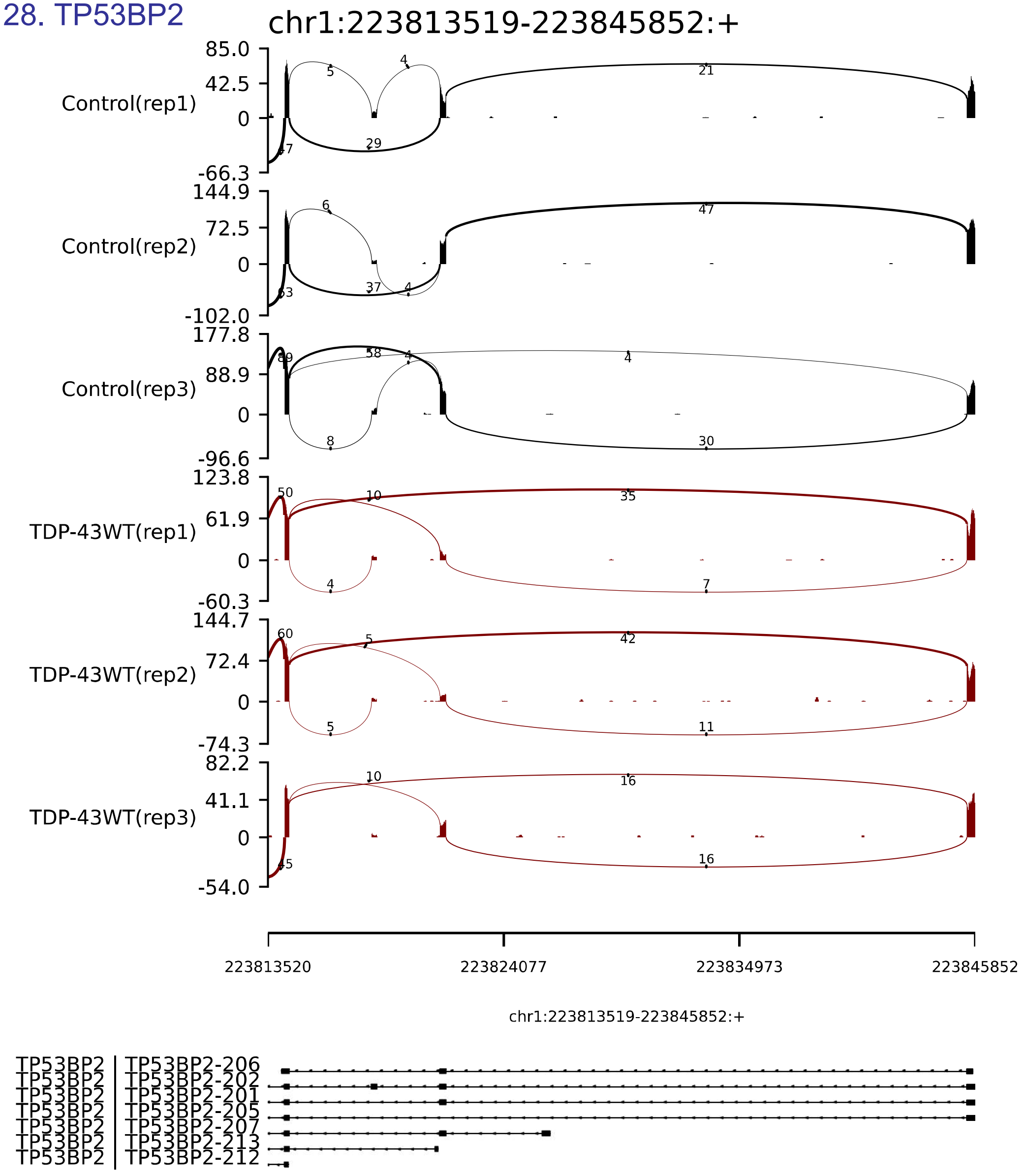

29. TNR

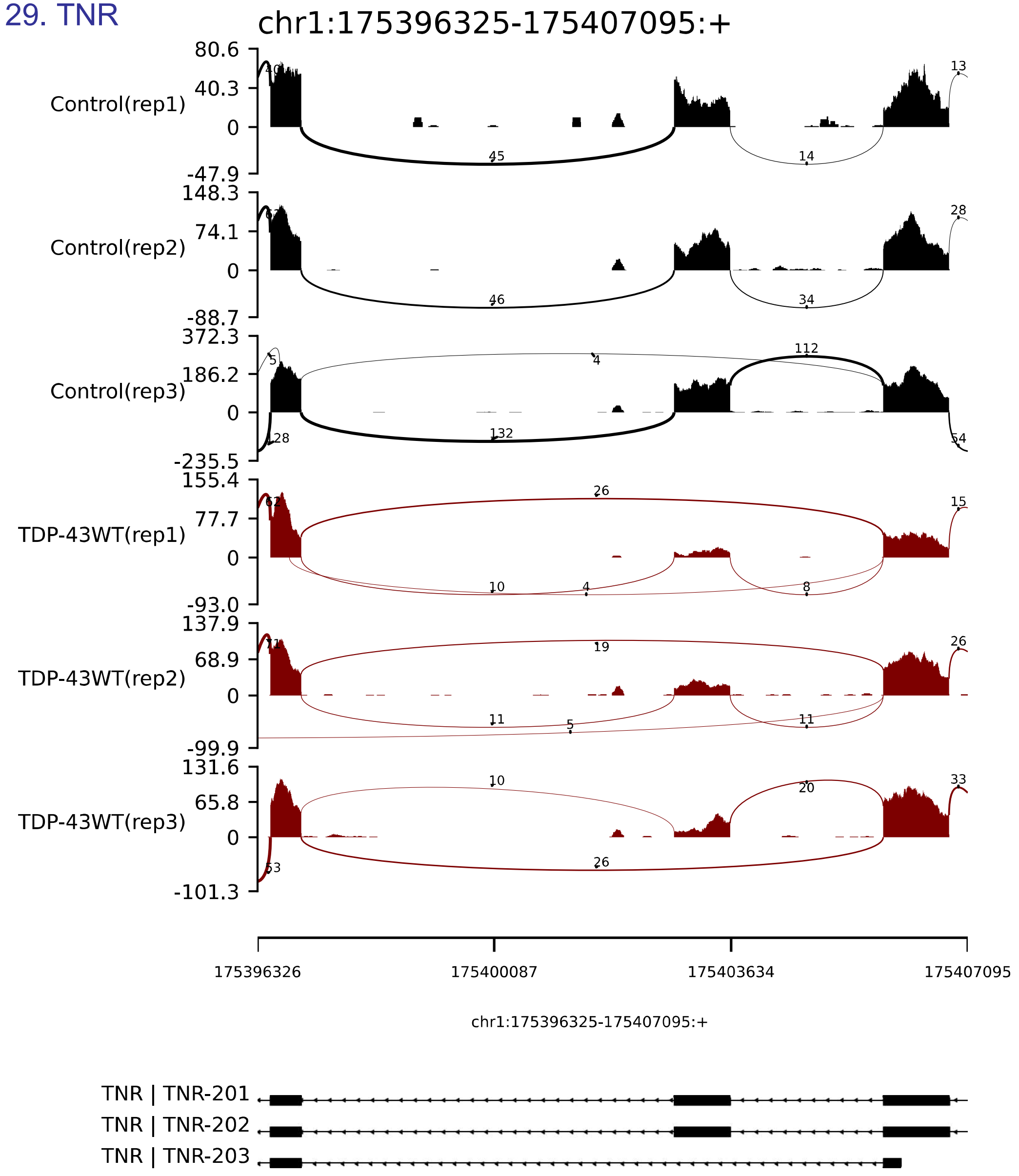

30. TMEM263

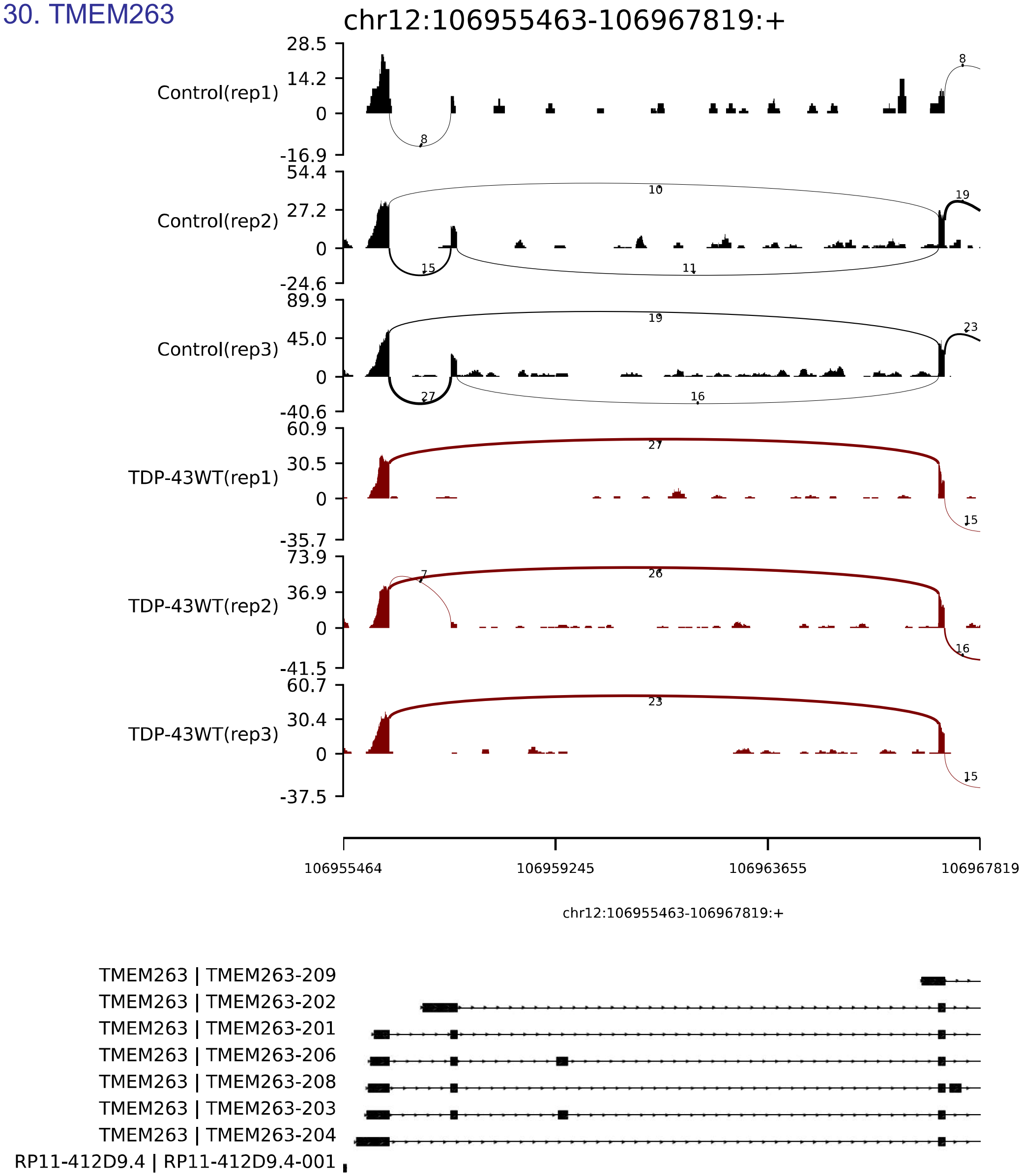

31. TESK1

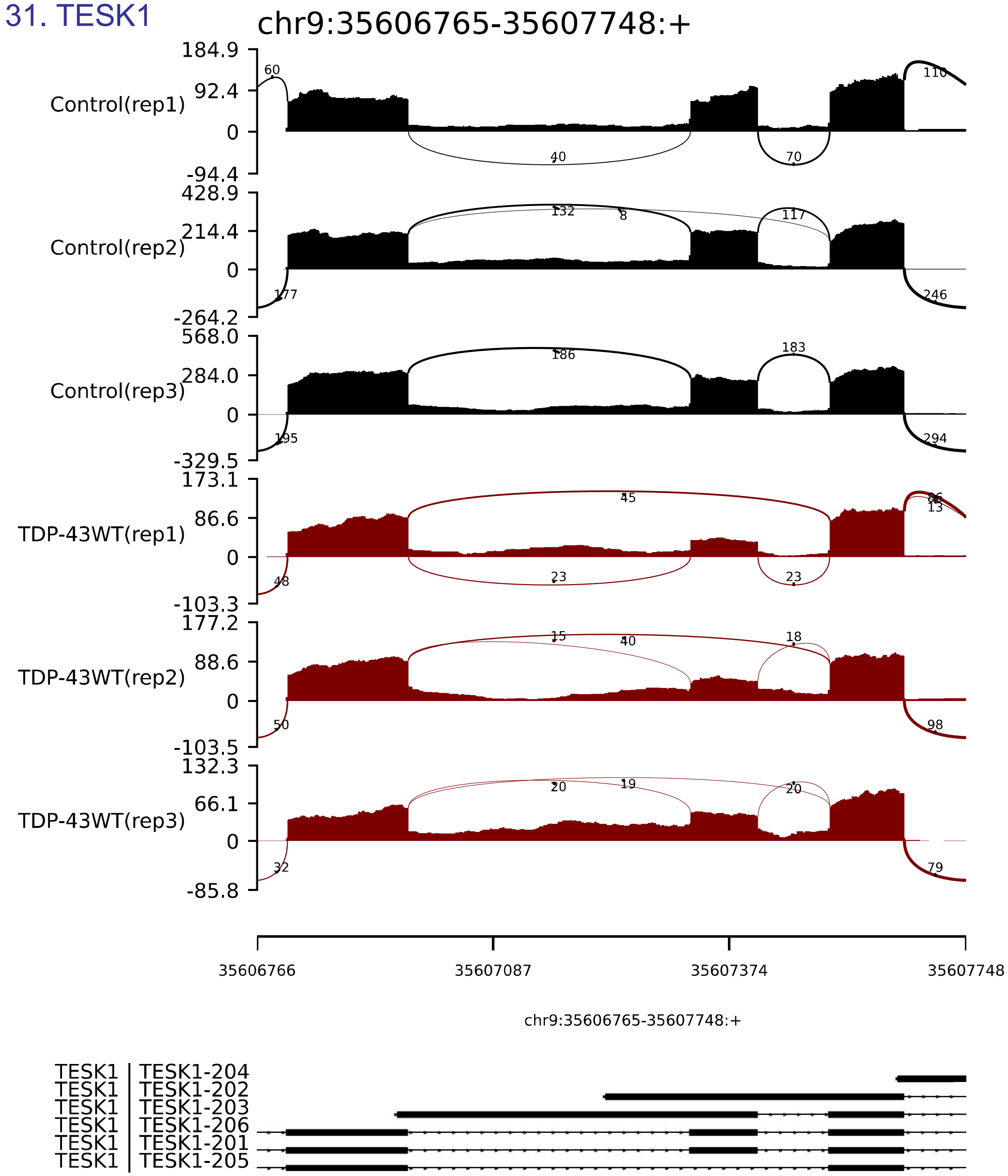

32. STC2

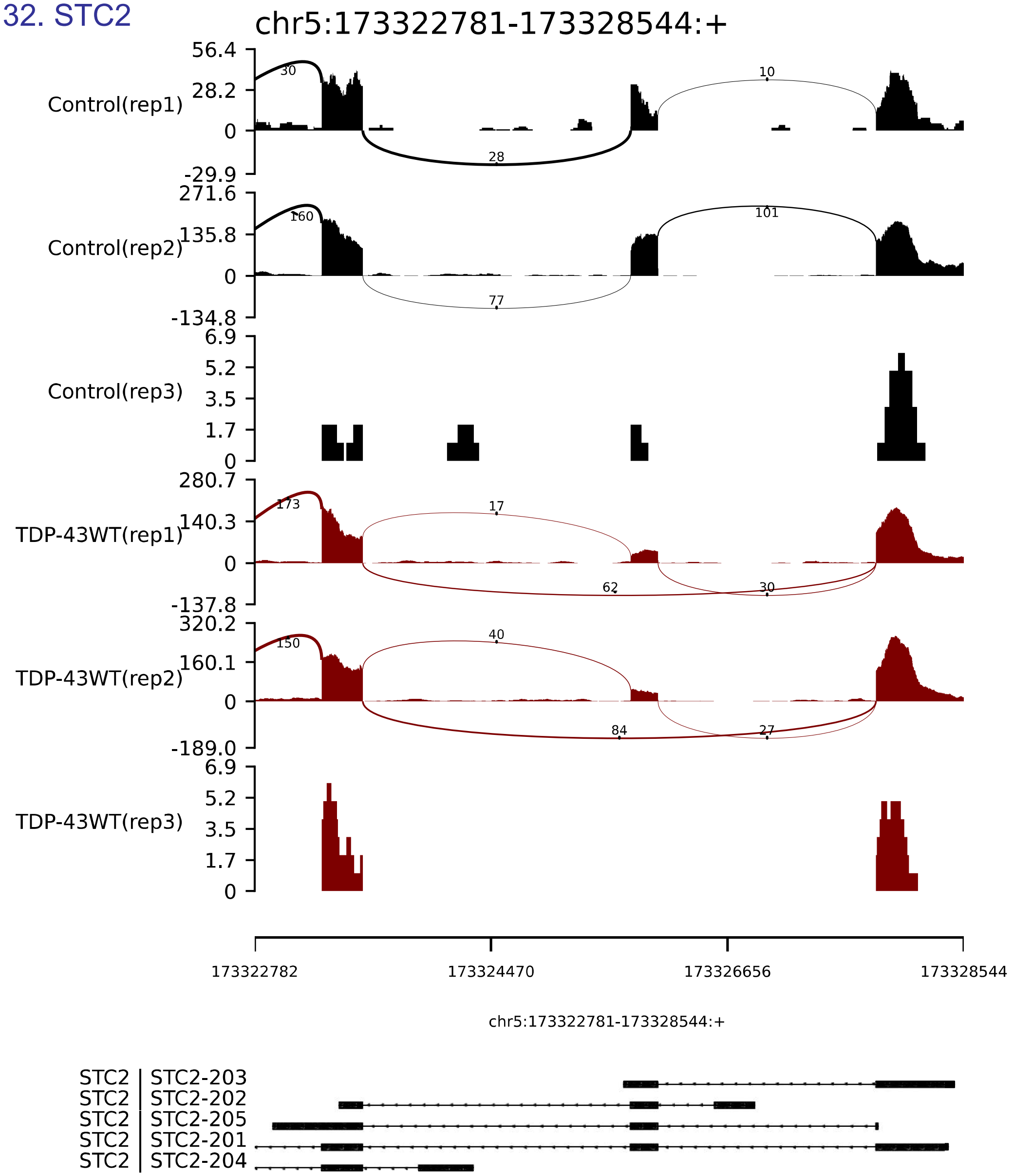

33. SLC17A5

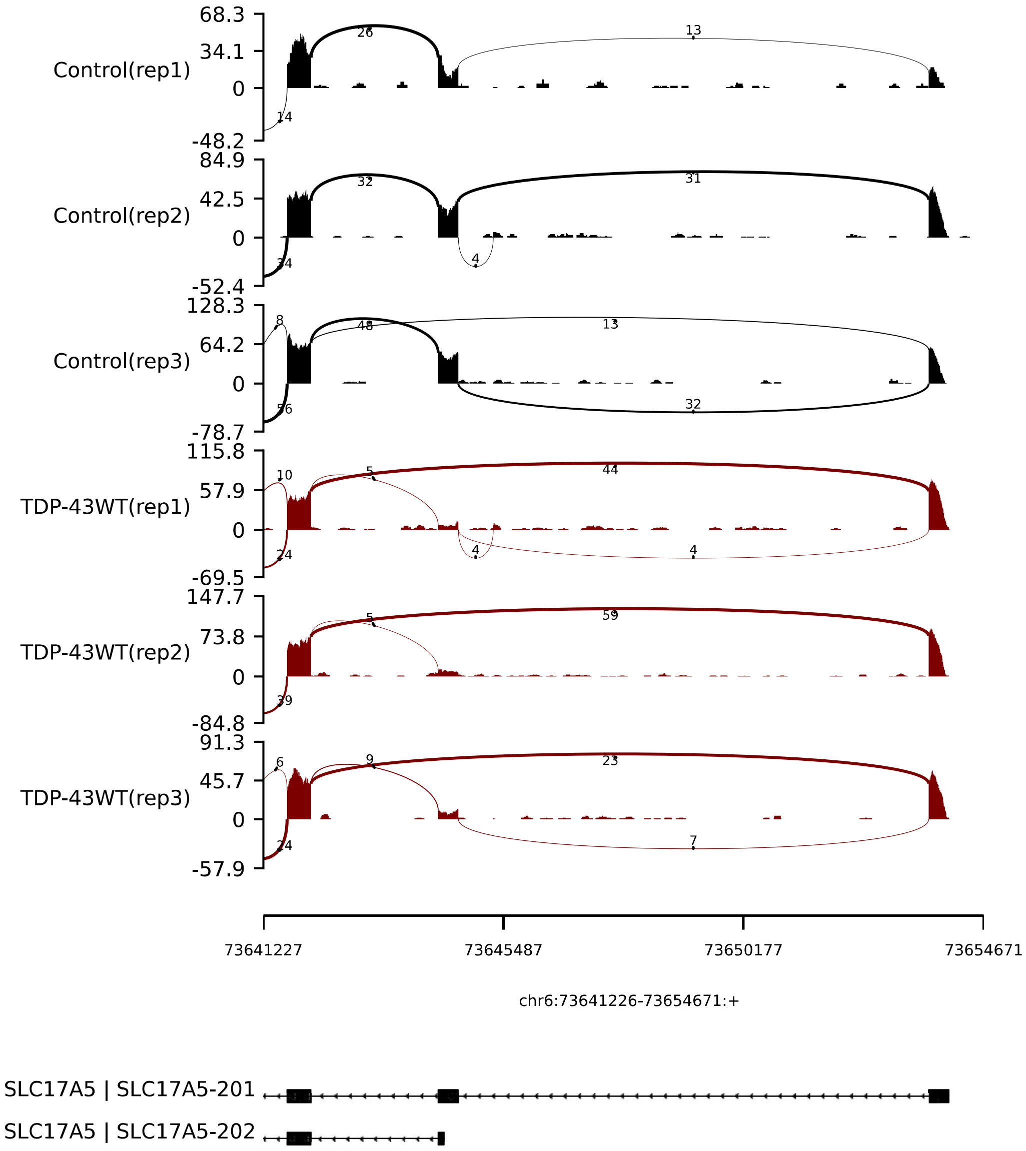

34. SCN9A

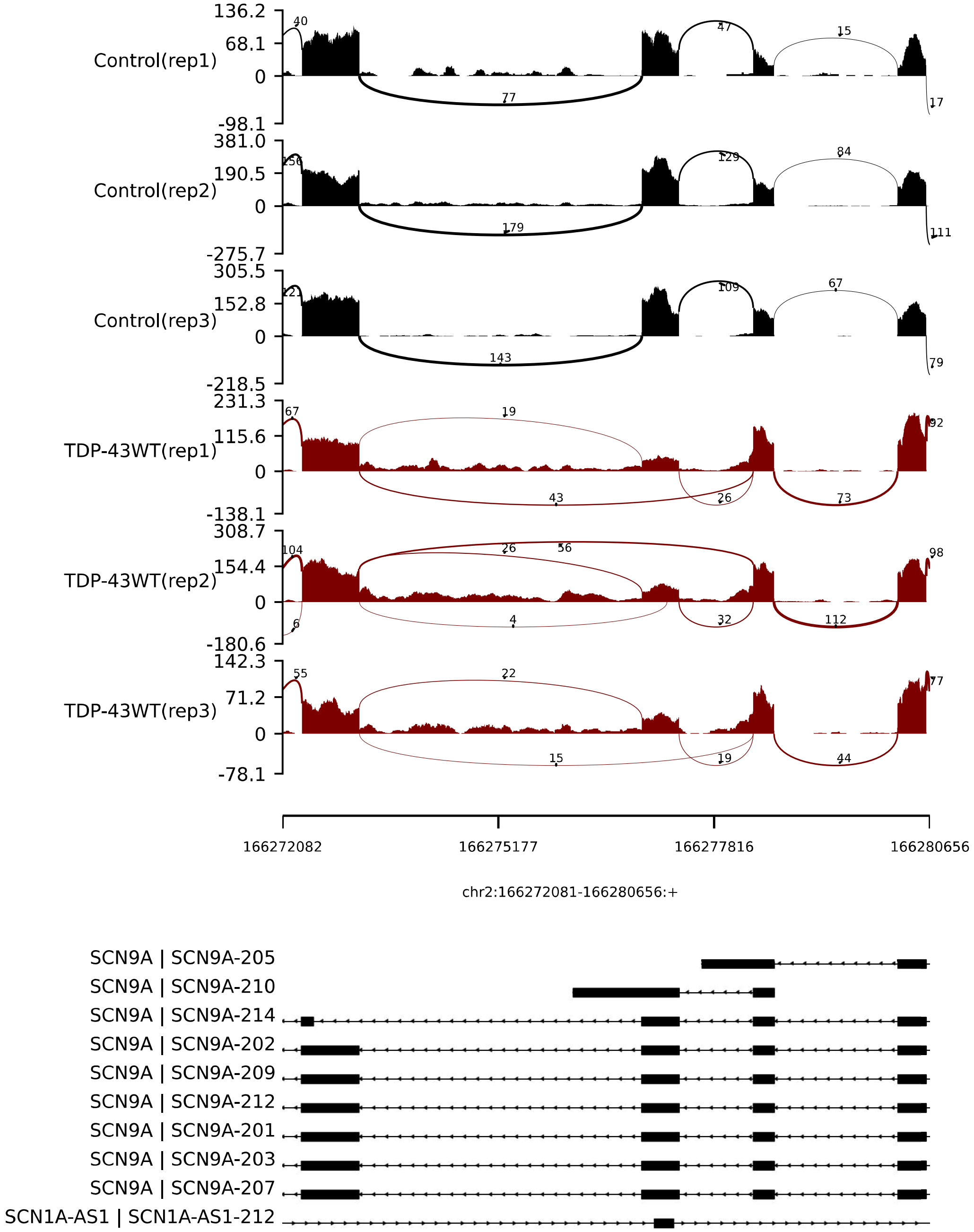

35. RNF114

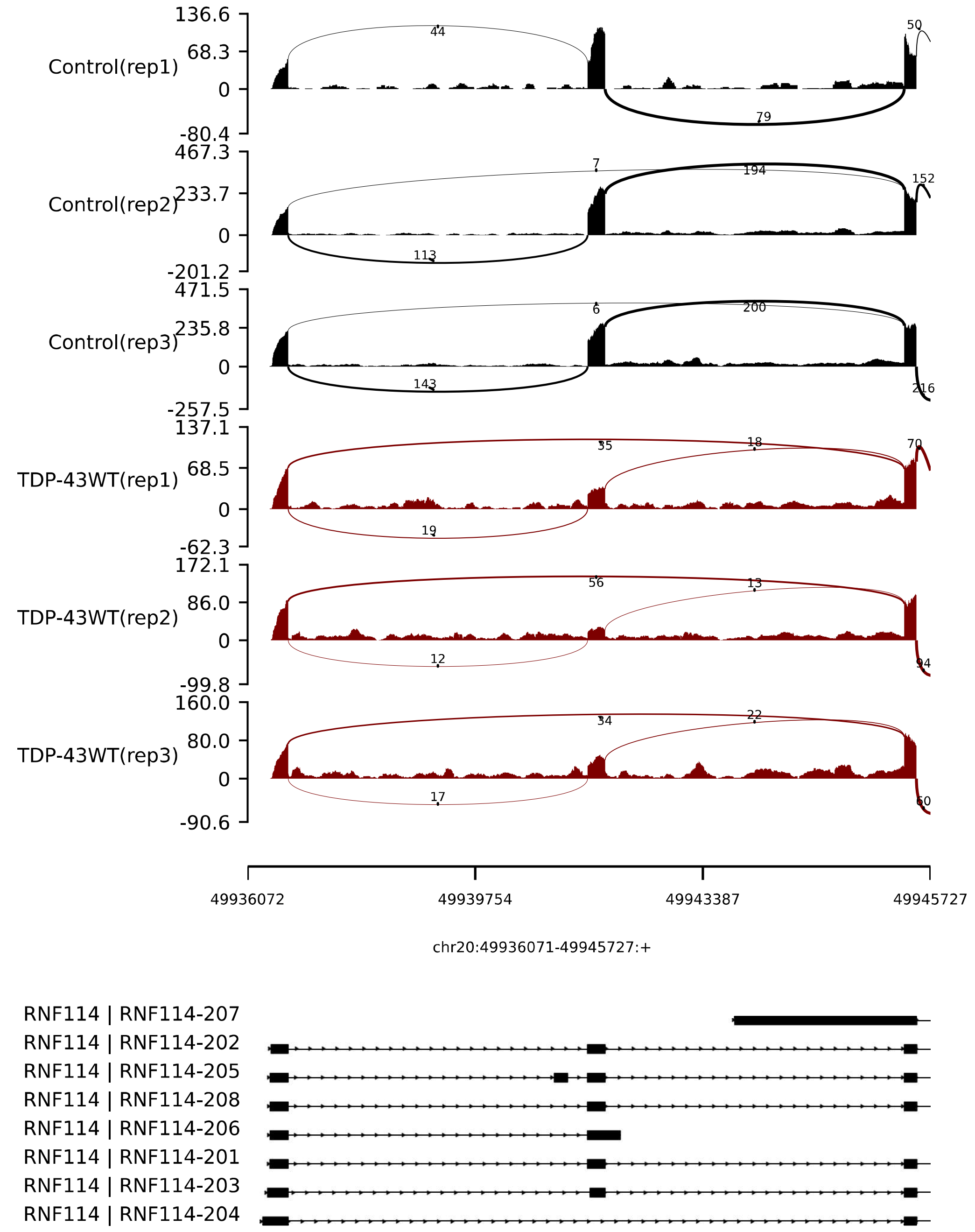

36. RHOT2

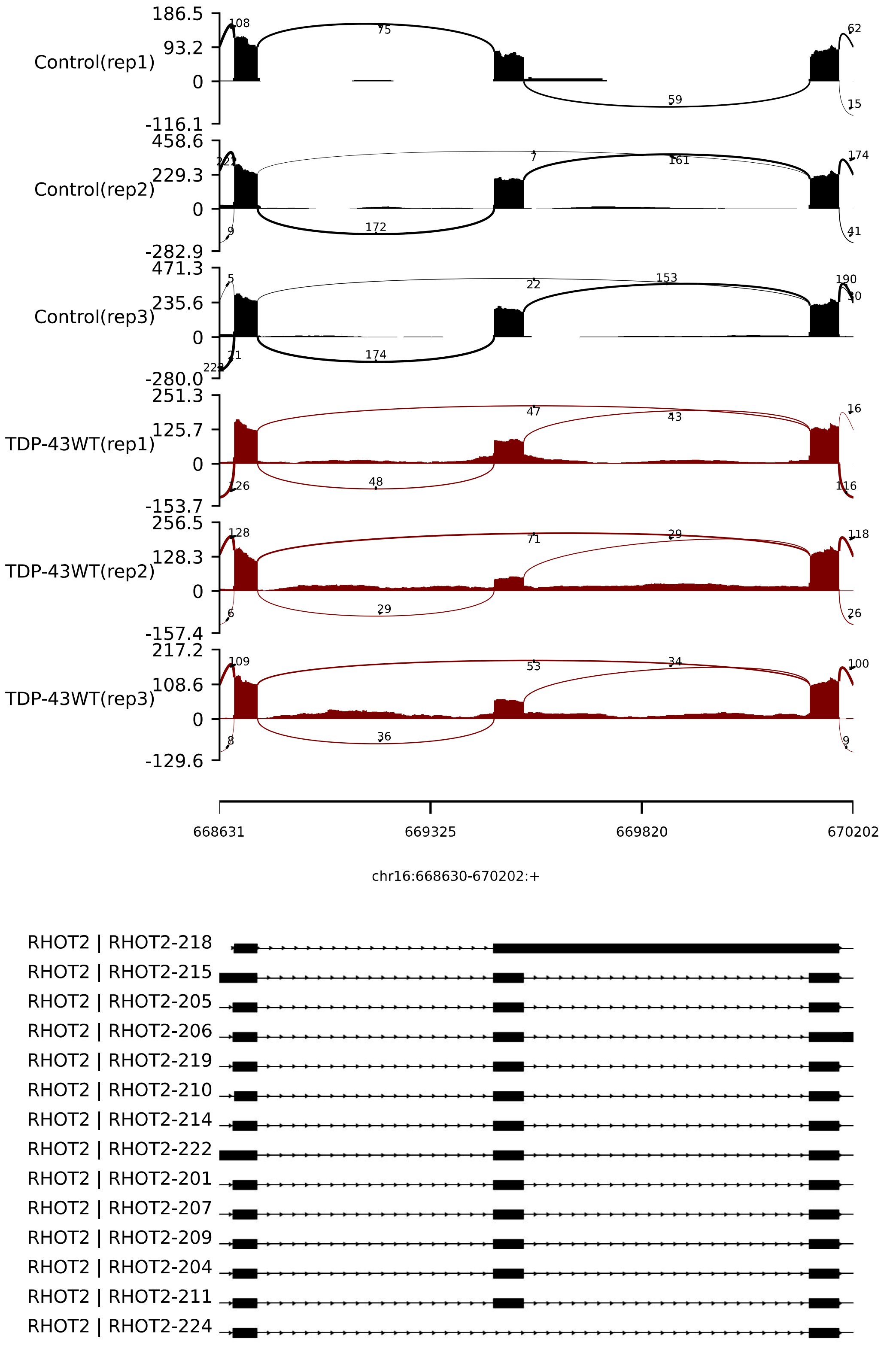

37. PPA1

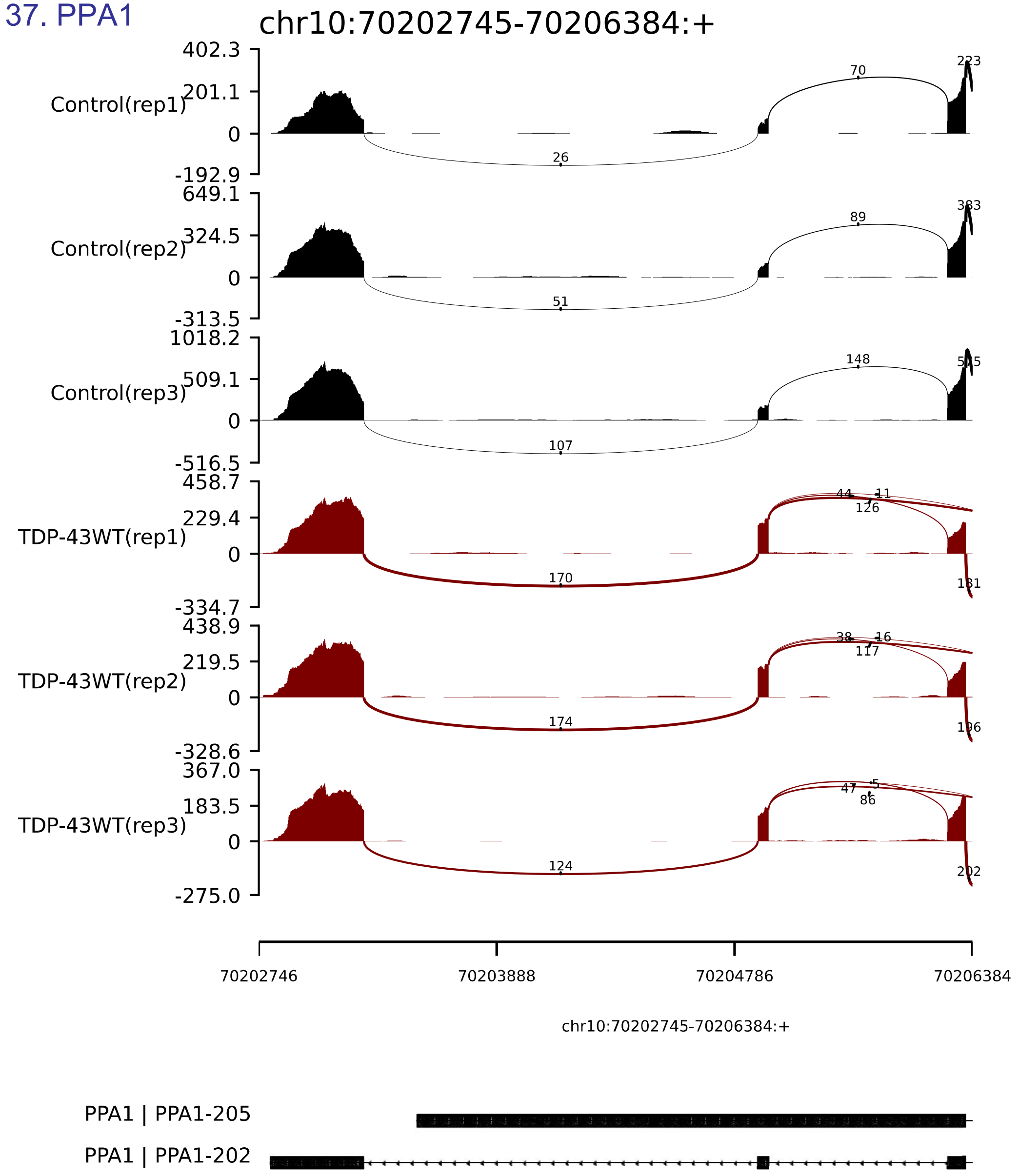

38. NIPAL3

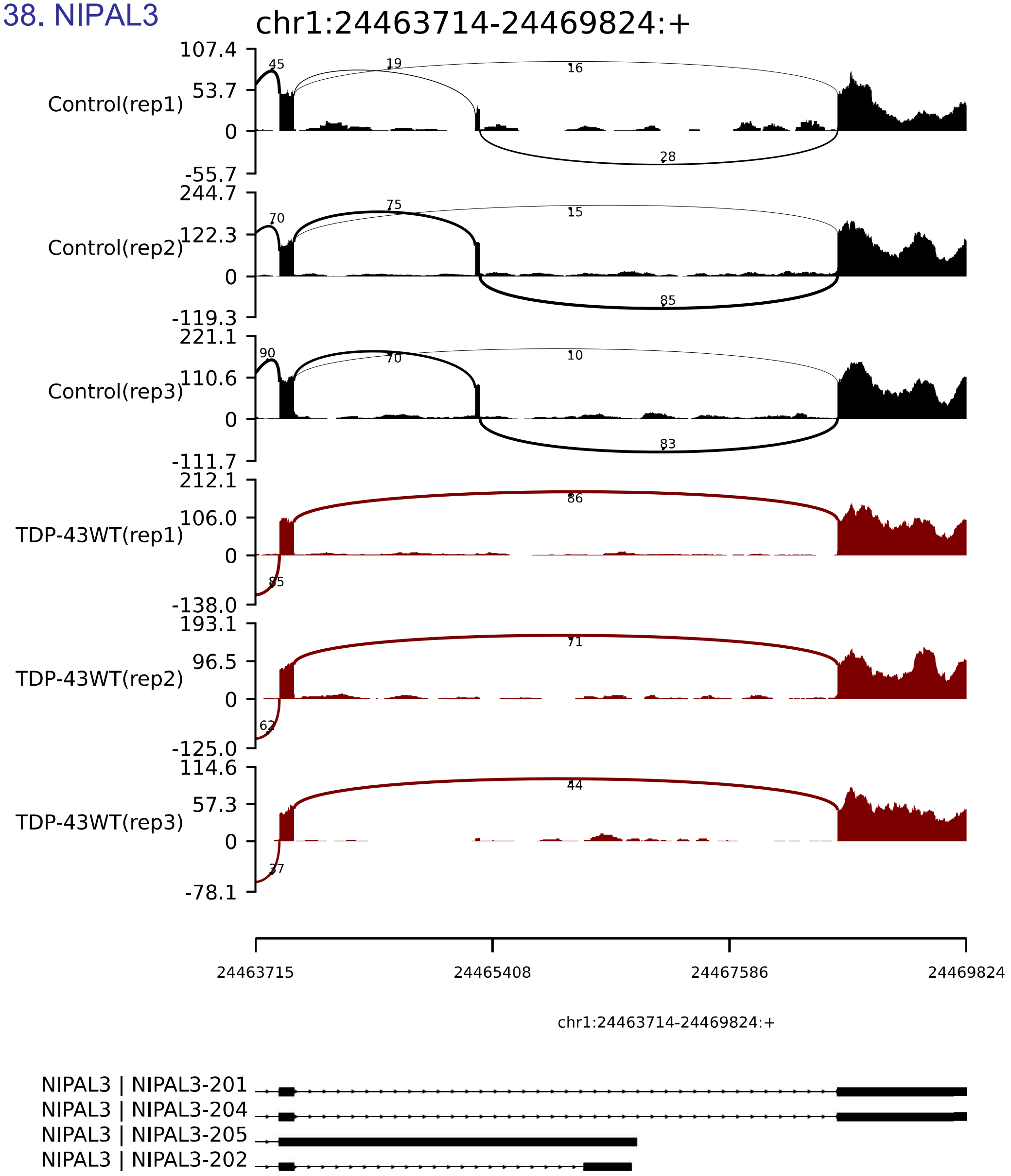

39. NICN1

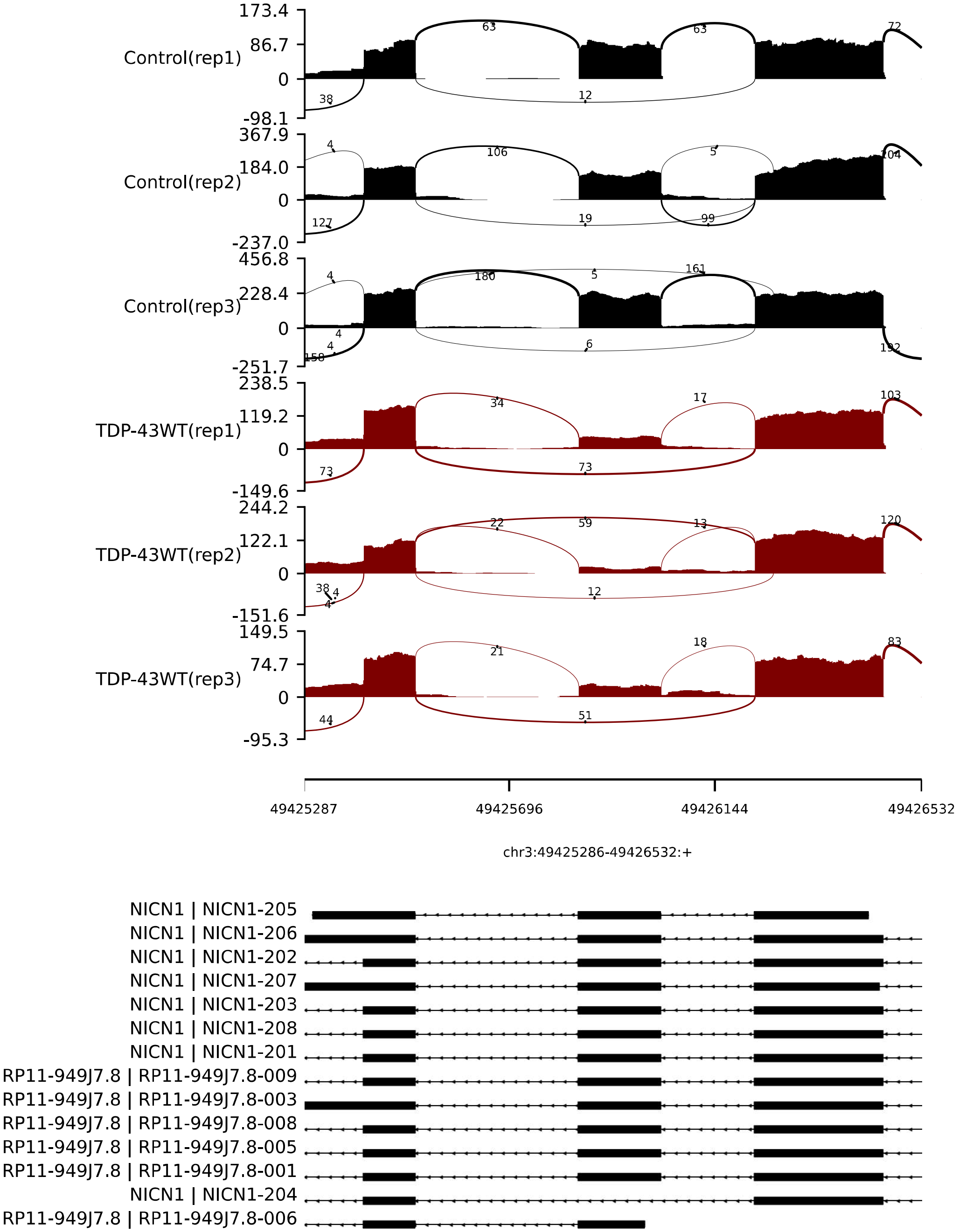

40. HIRA

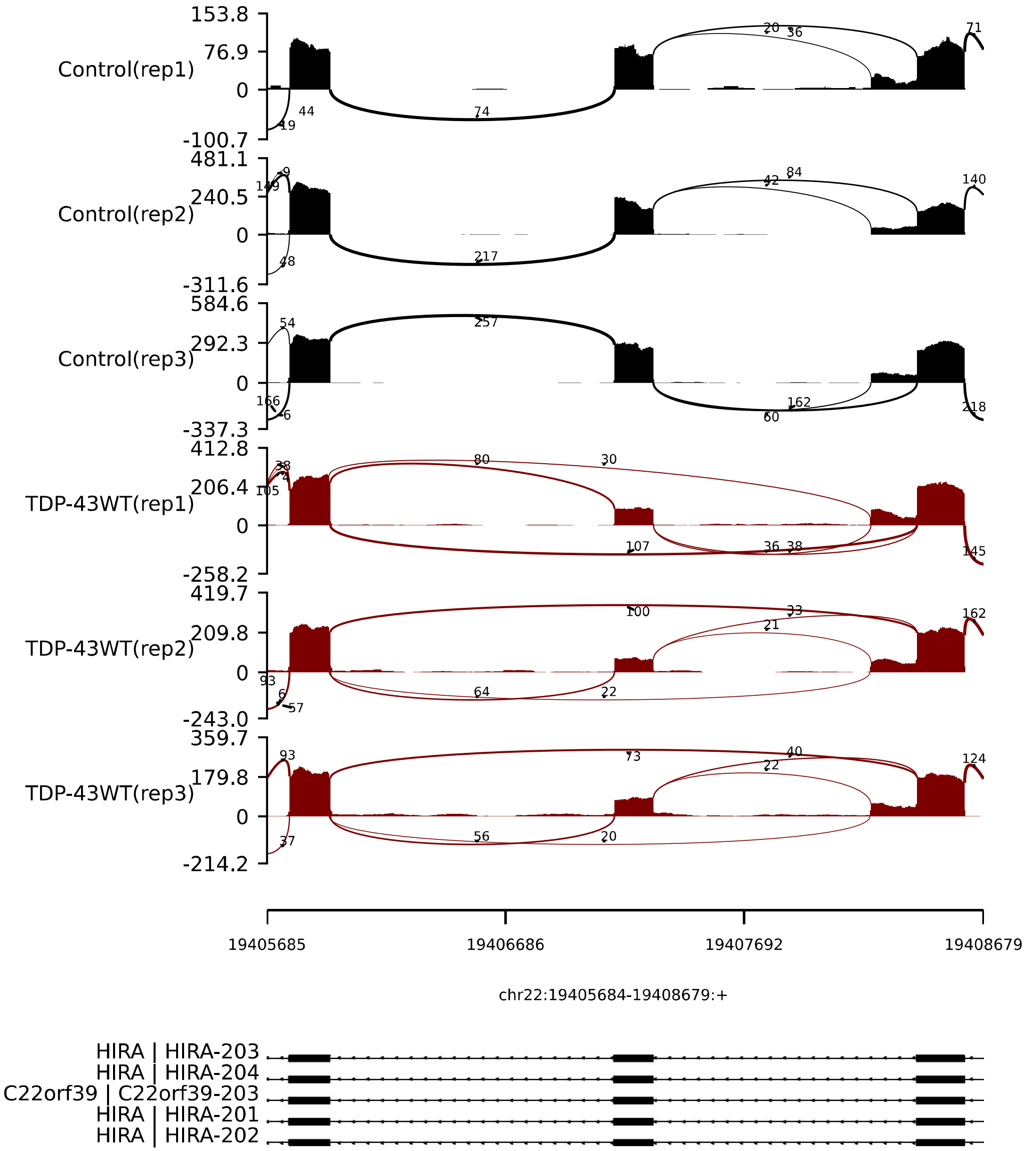

41. HCFC2

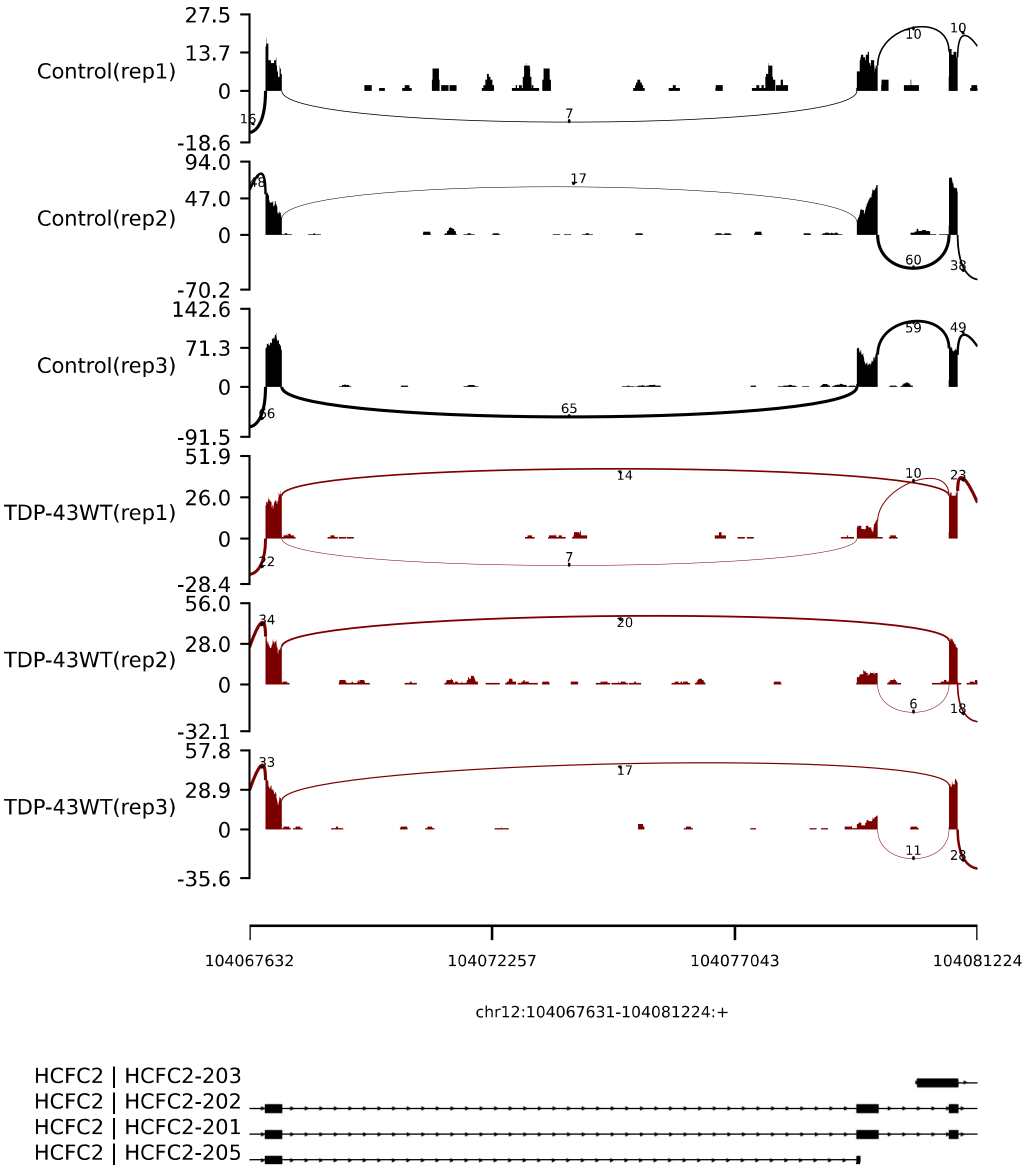

42. HARS2

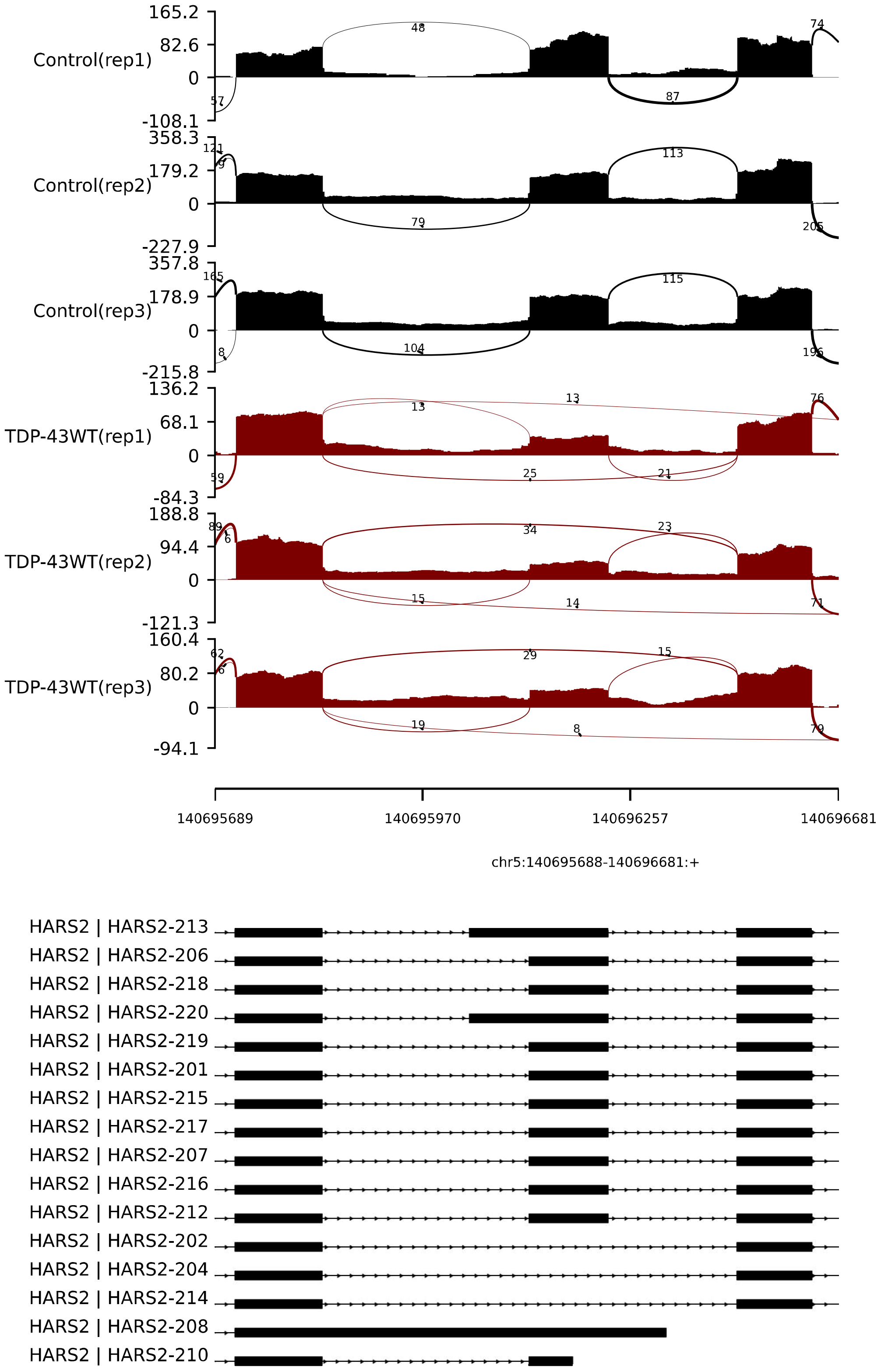

43. FRAS1

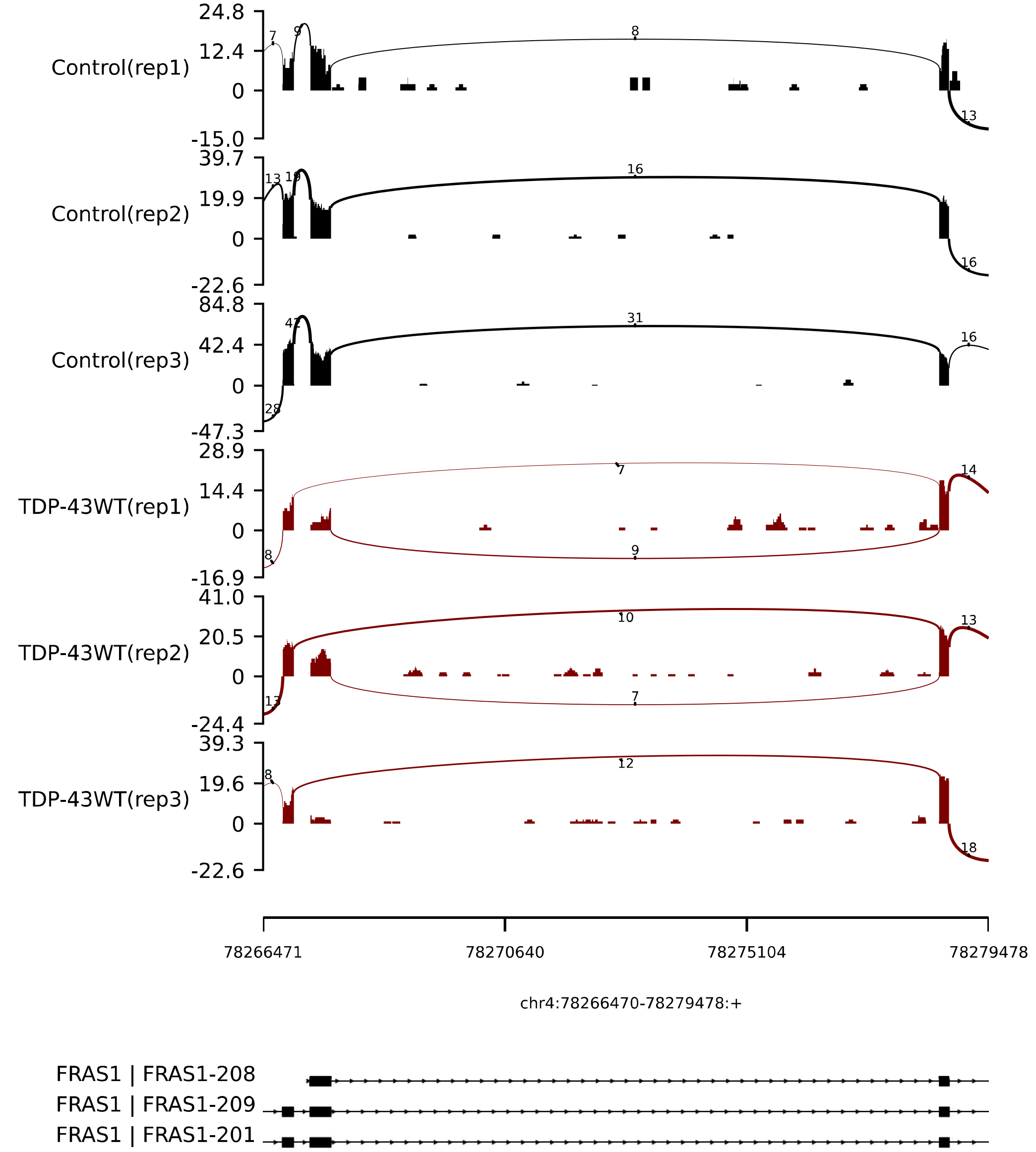

44. FBRS

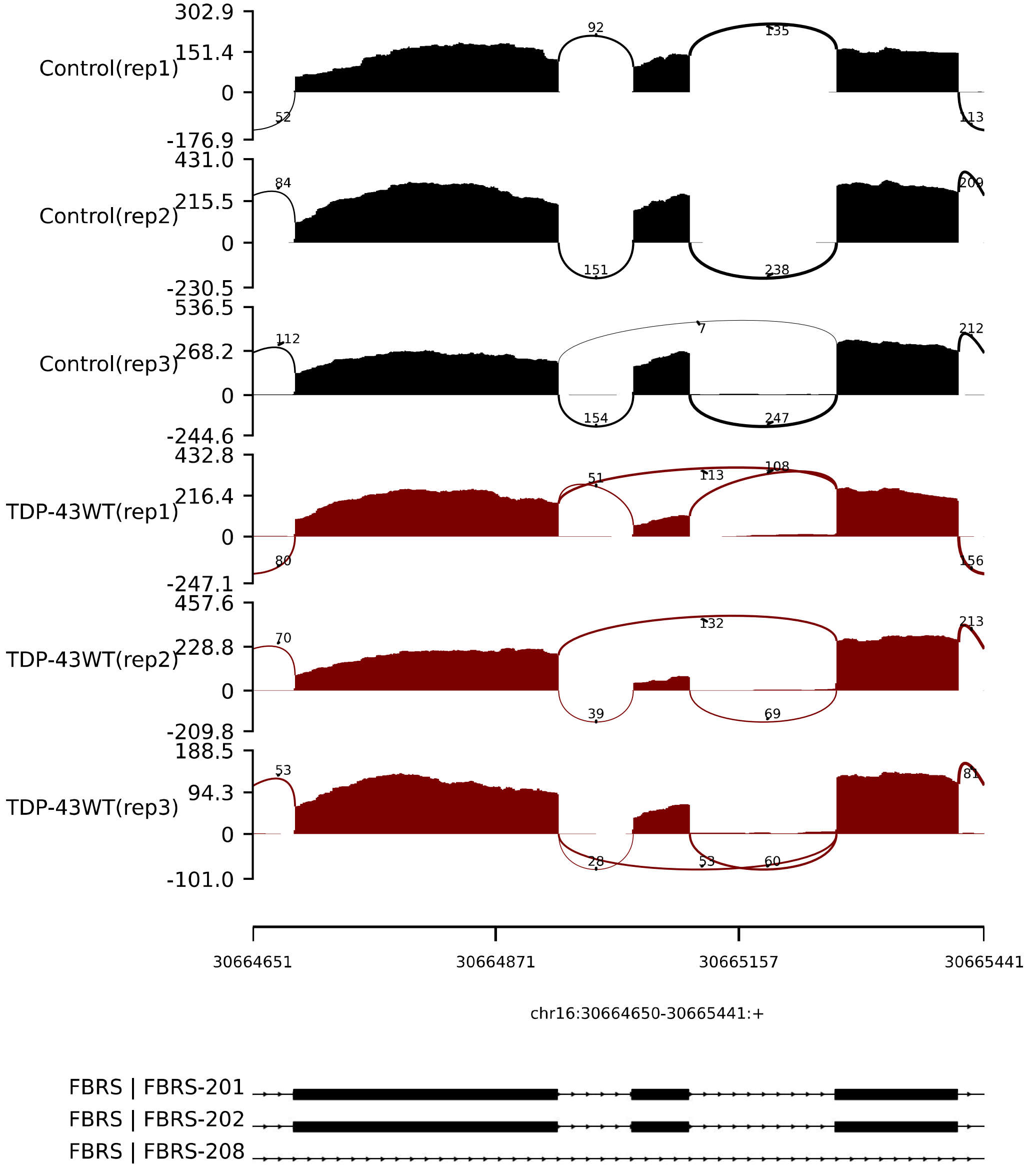

45. FAT1

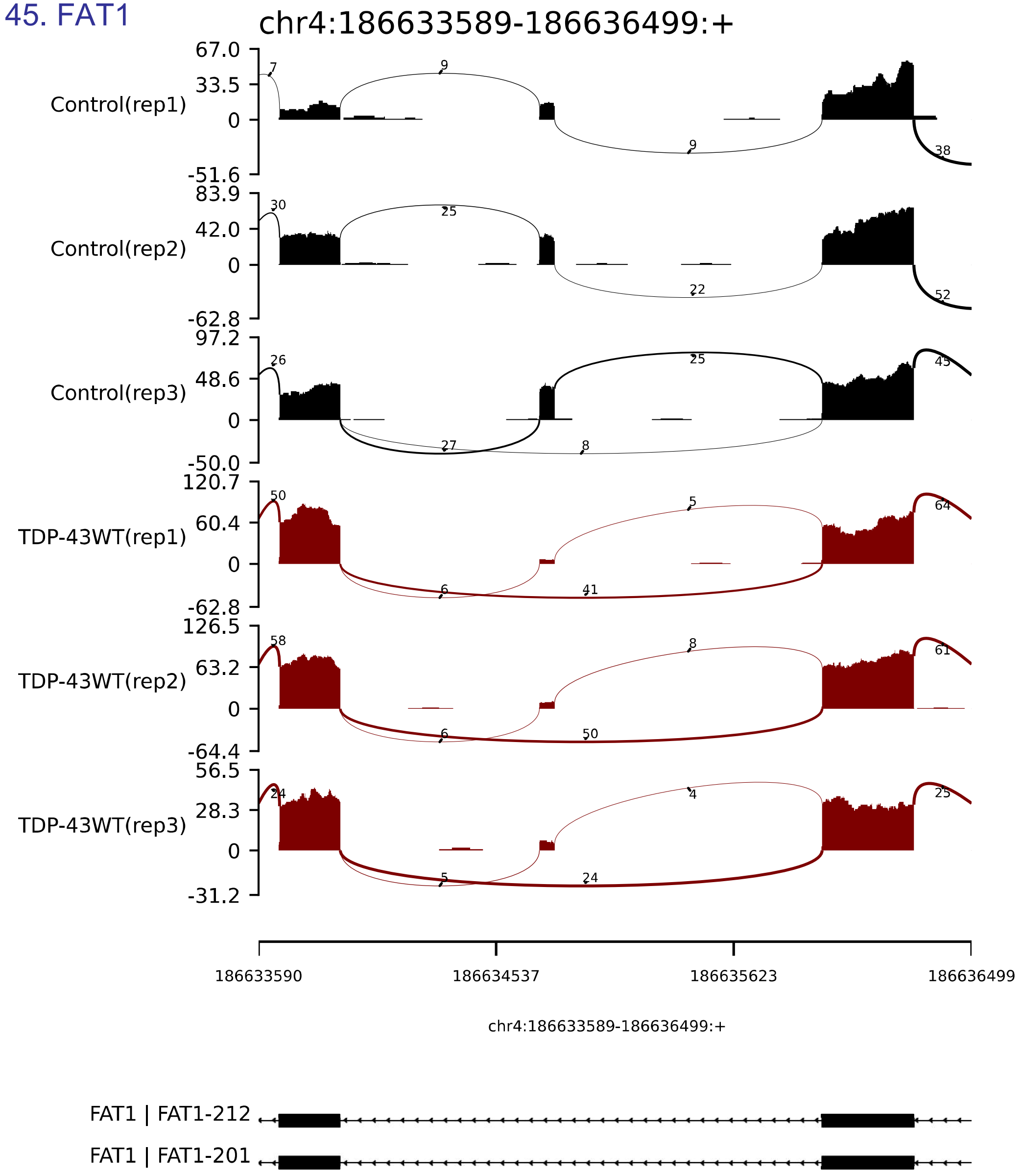

46. FAM66C

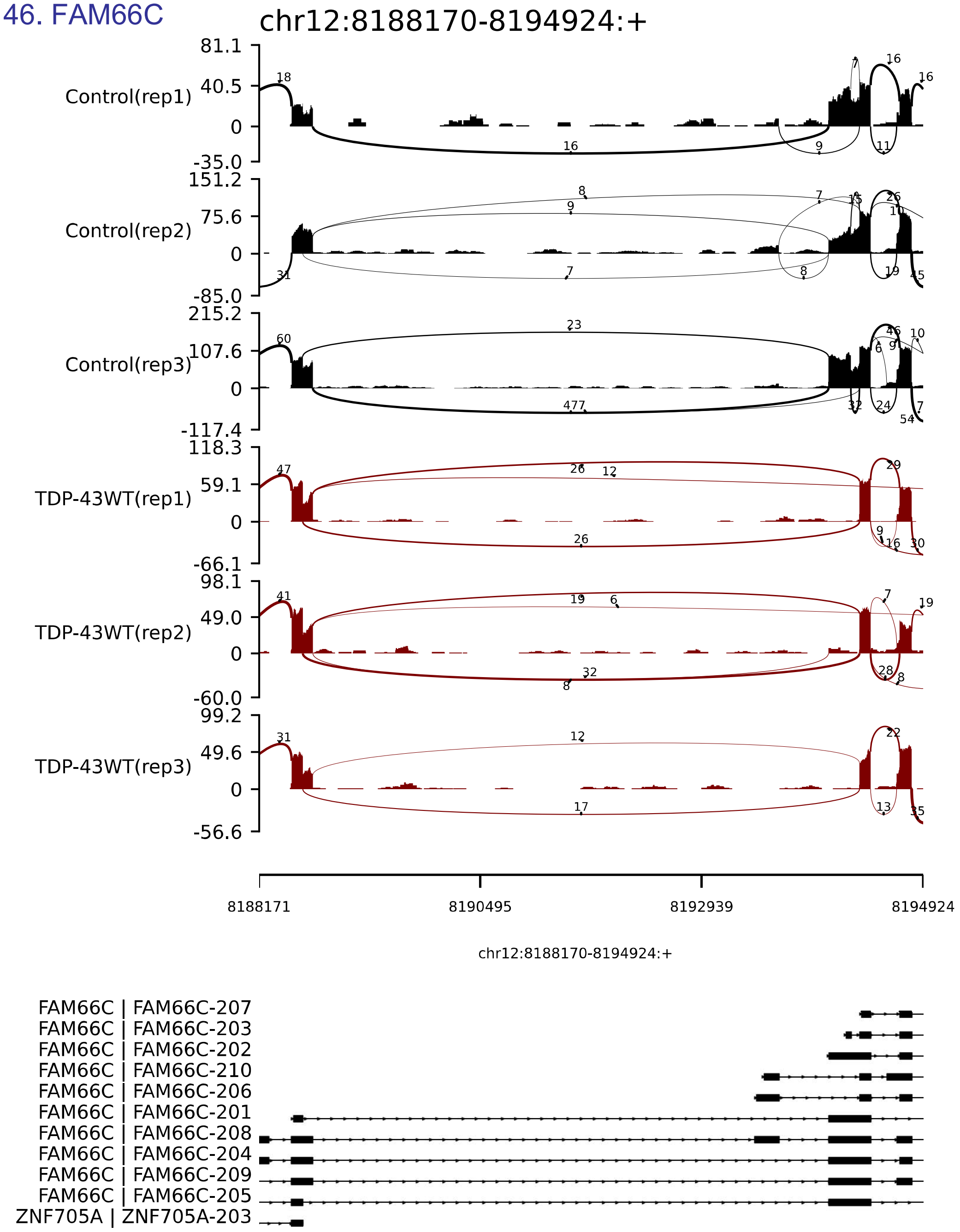

47. FAM102A

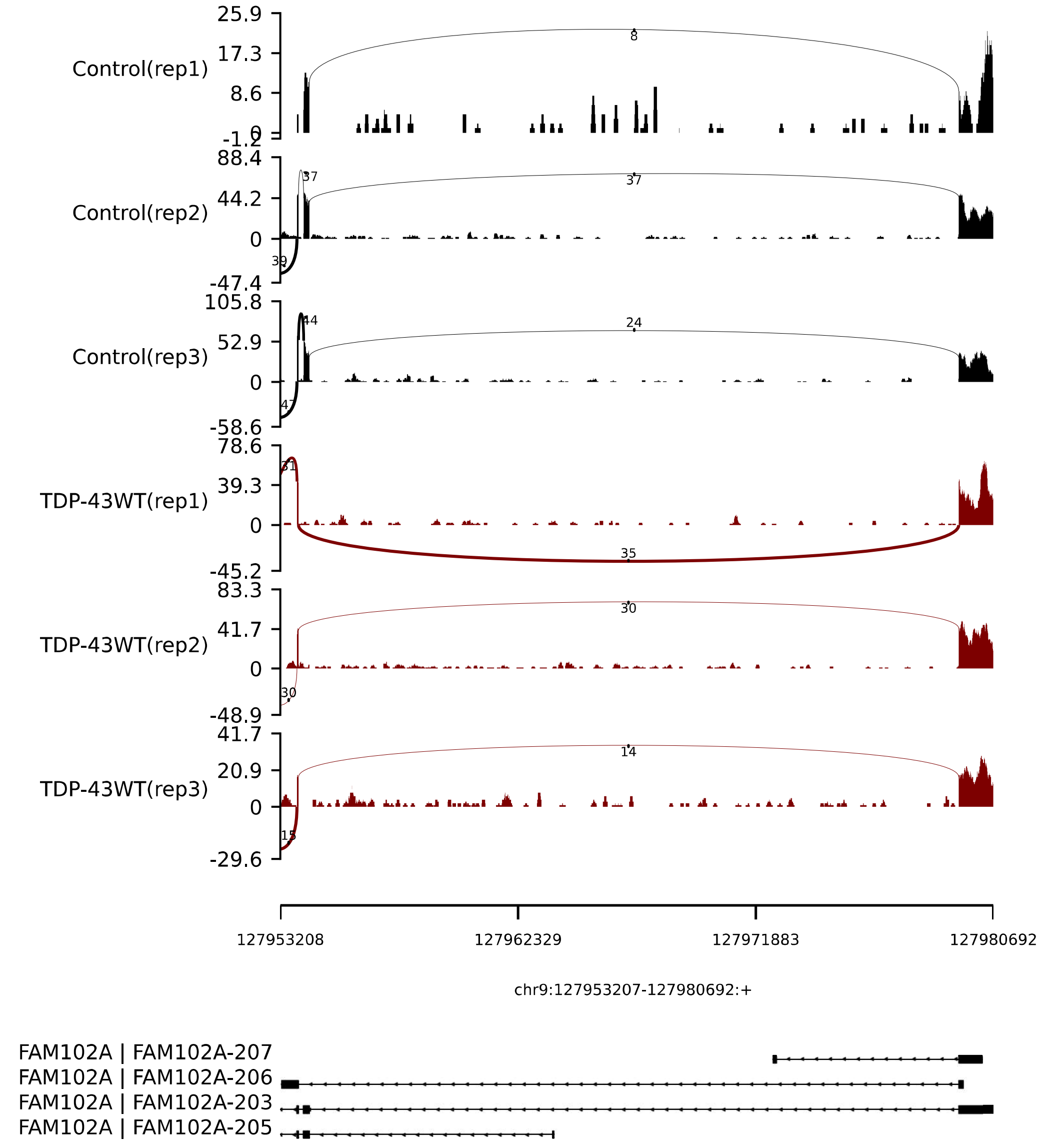

48. ERMAD

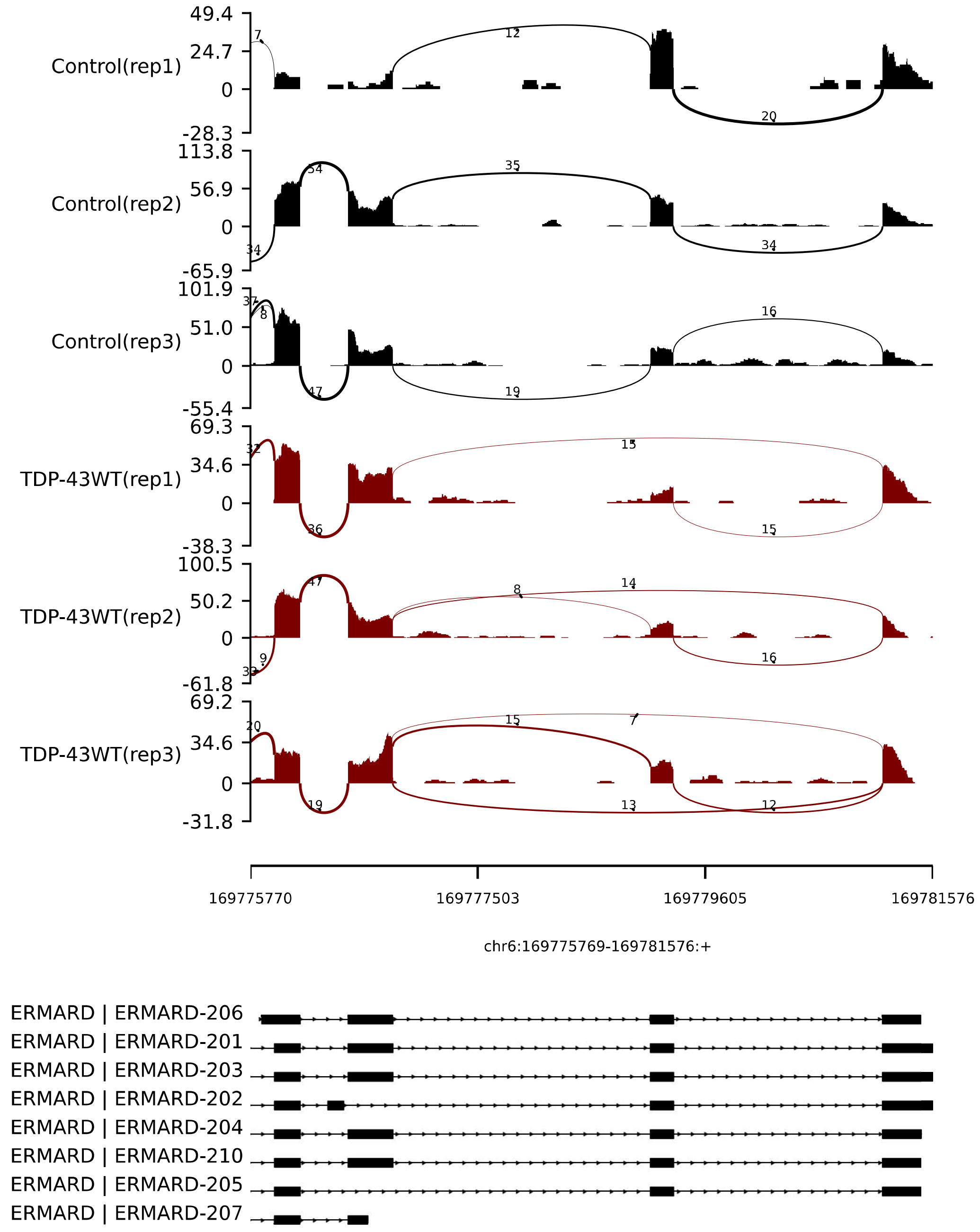

49. DRGX

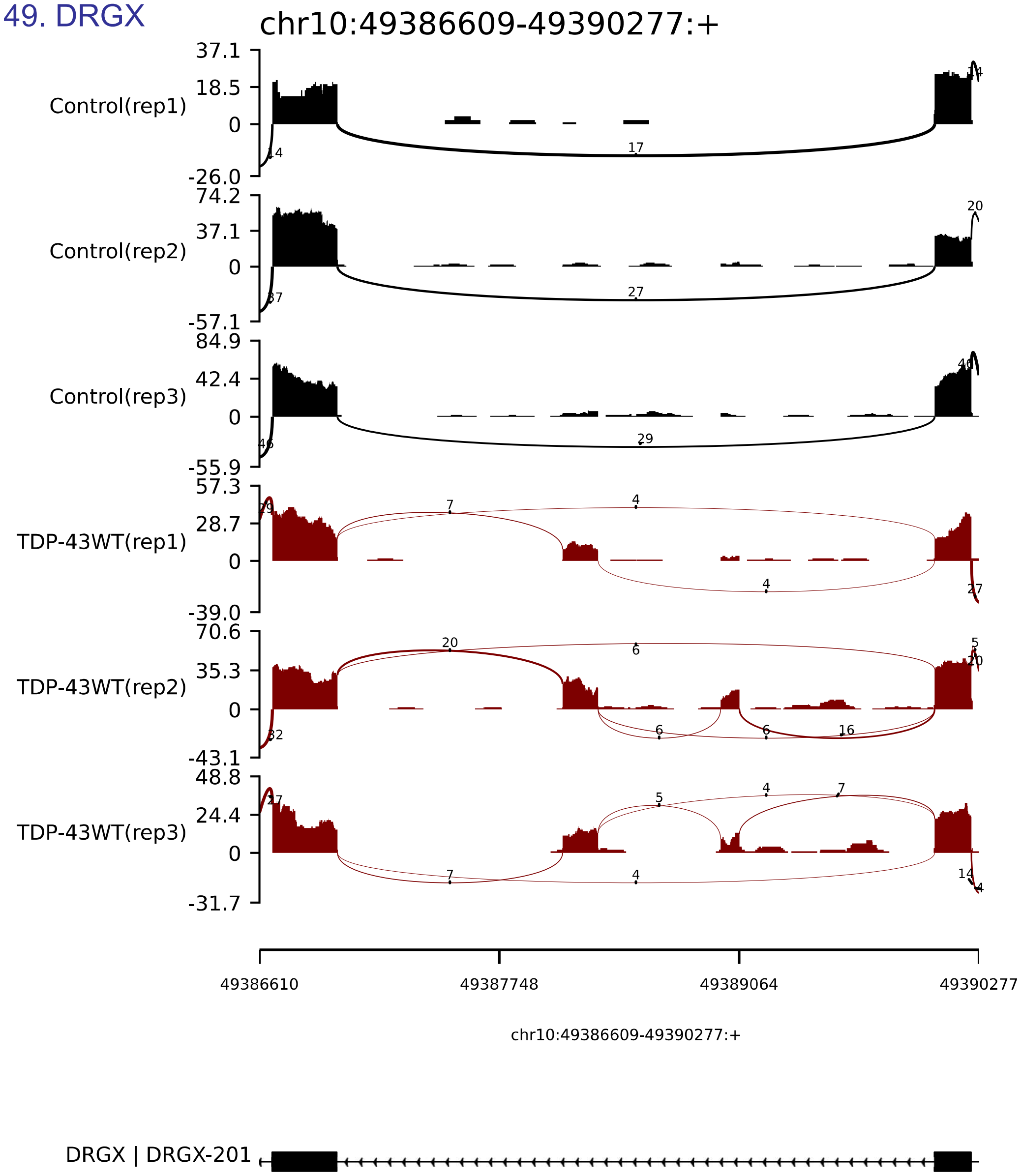

50. DDI2

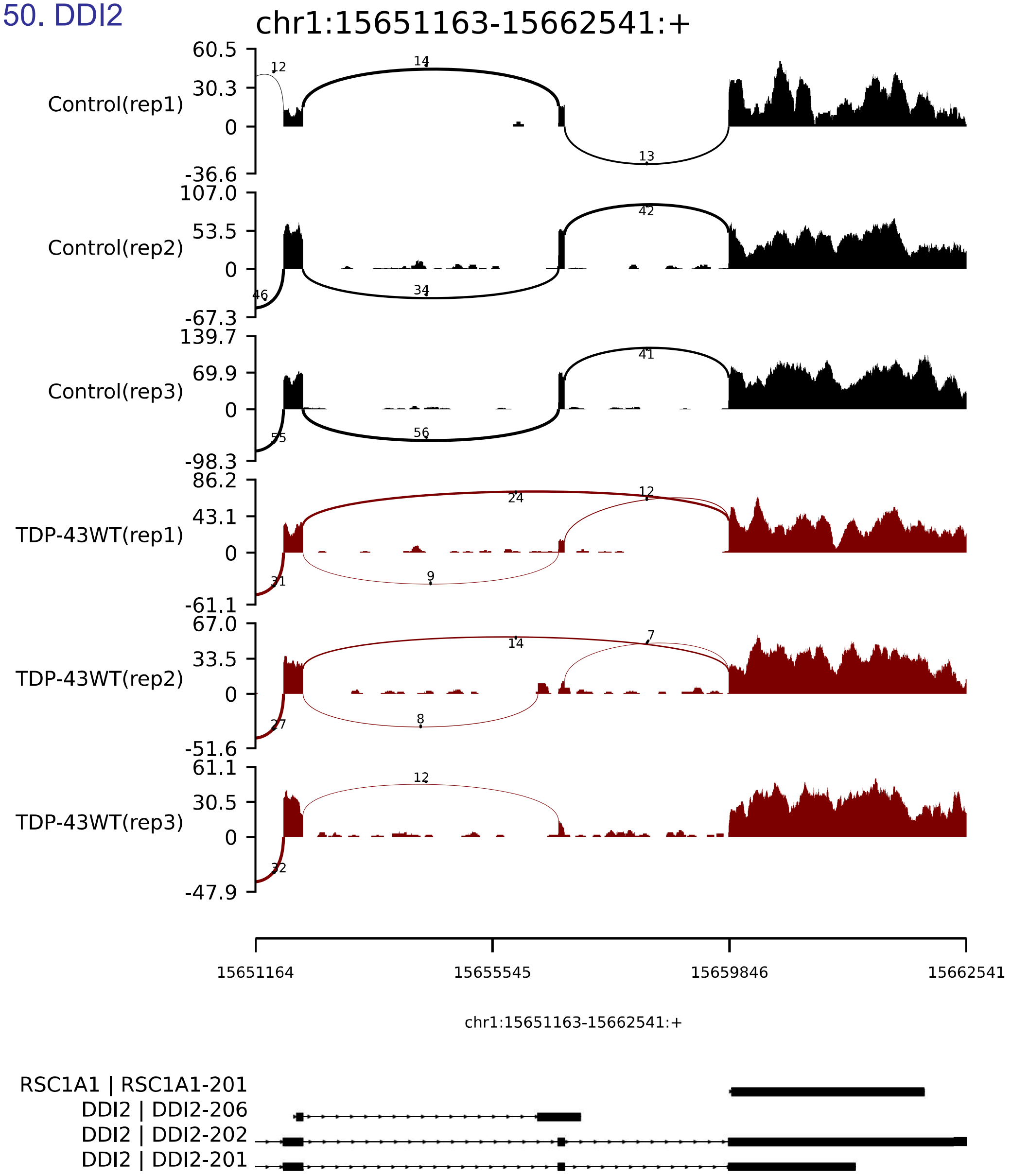

51. COQ5

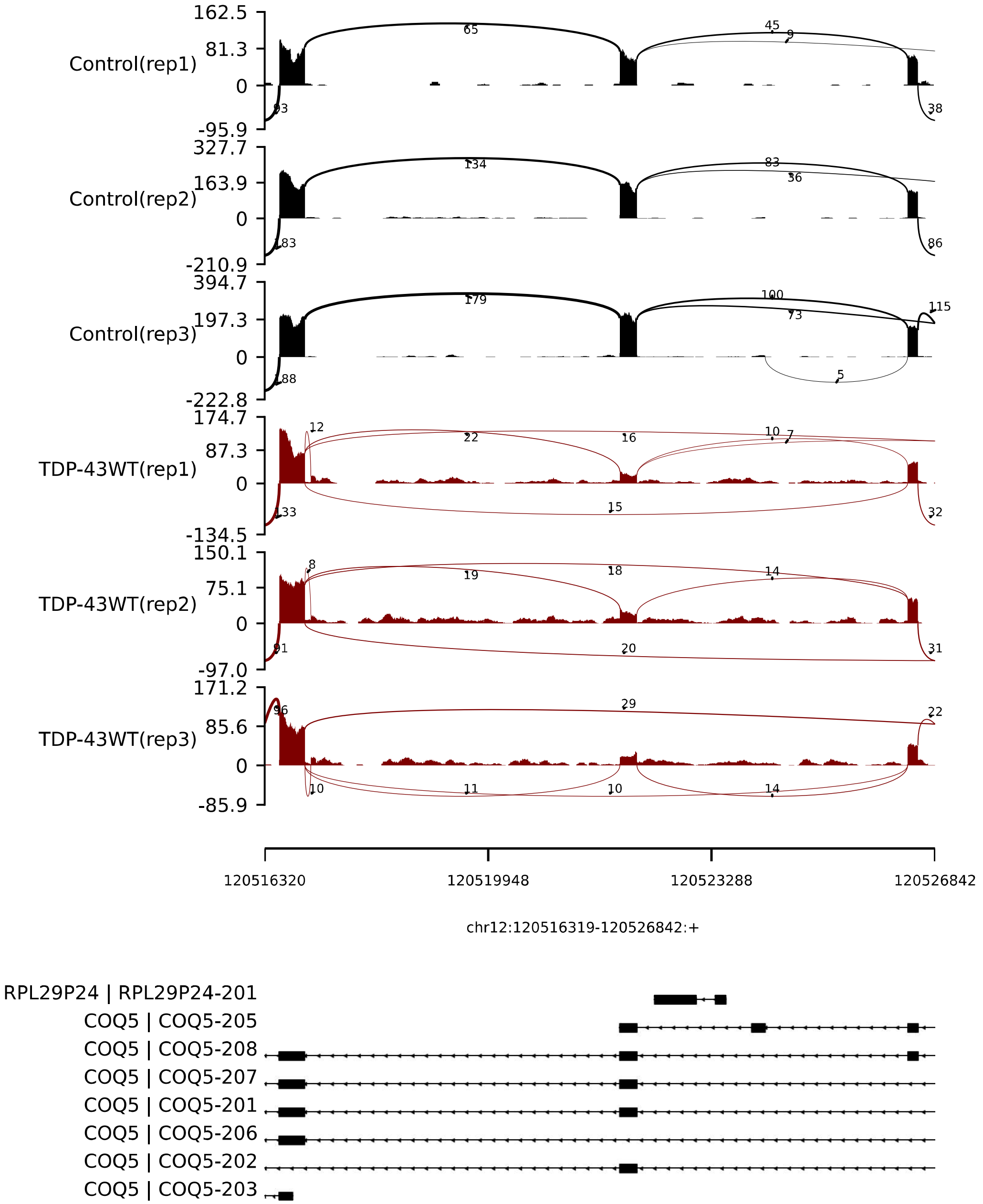

52. CANX

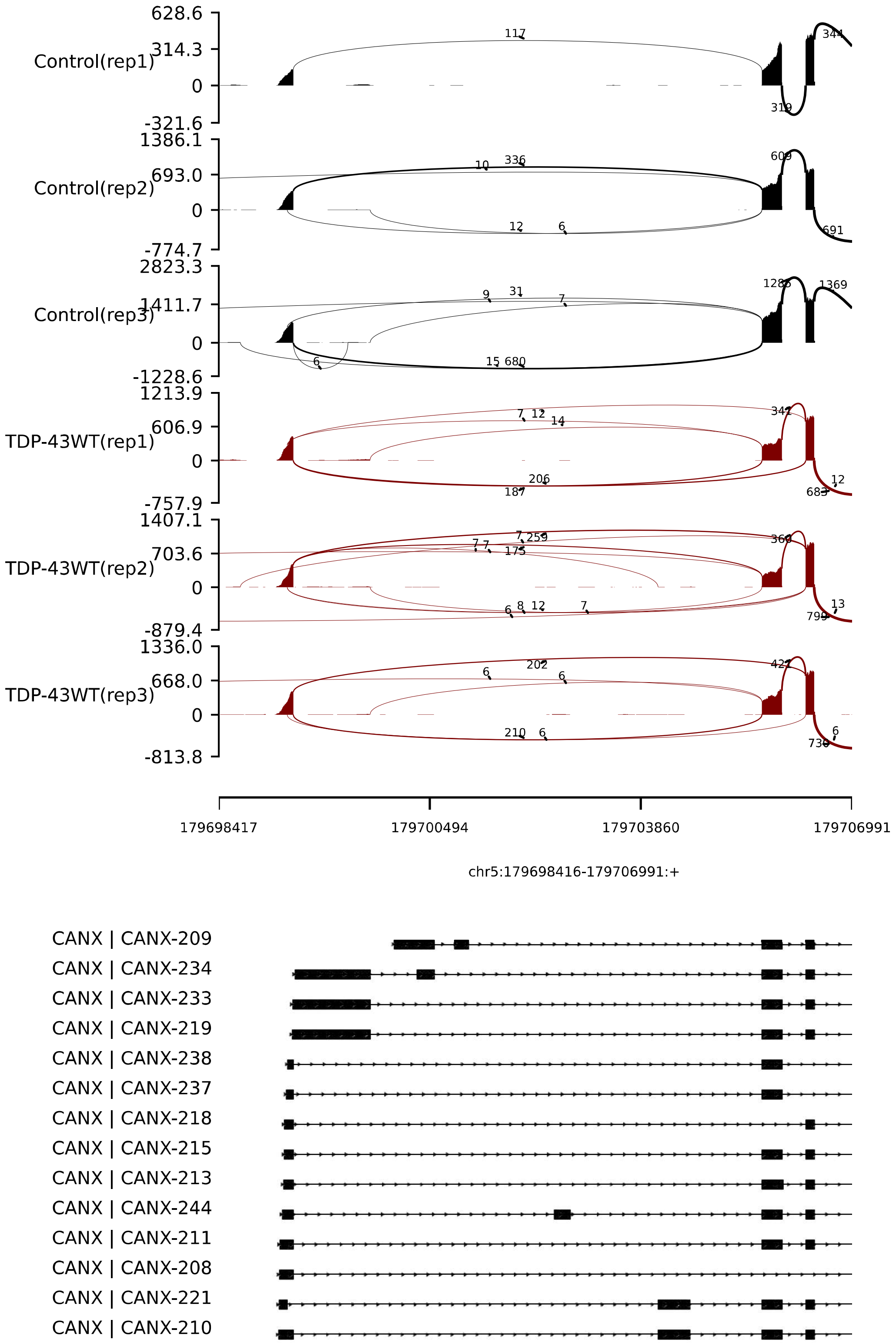

53. BMPR1A

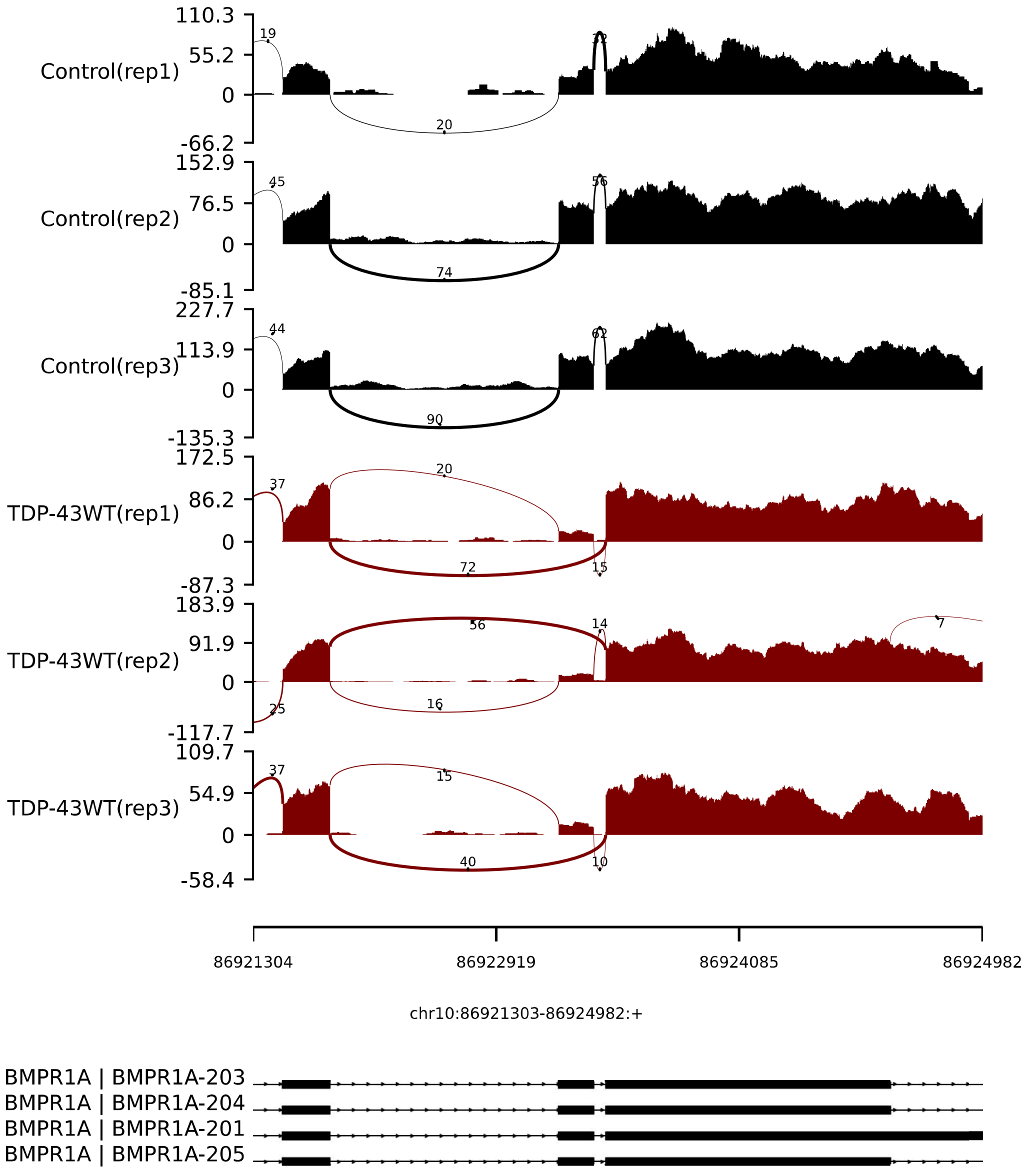

54. ATP9B

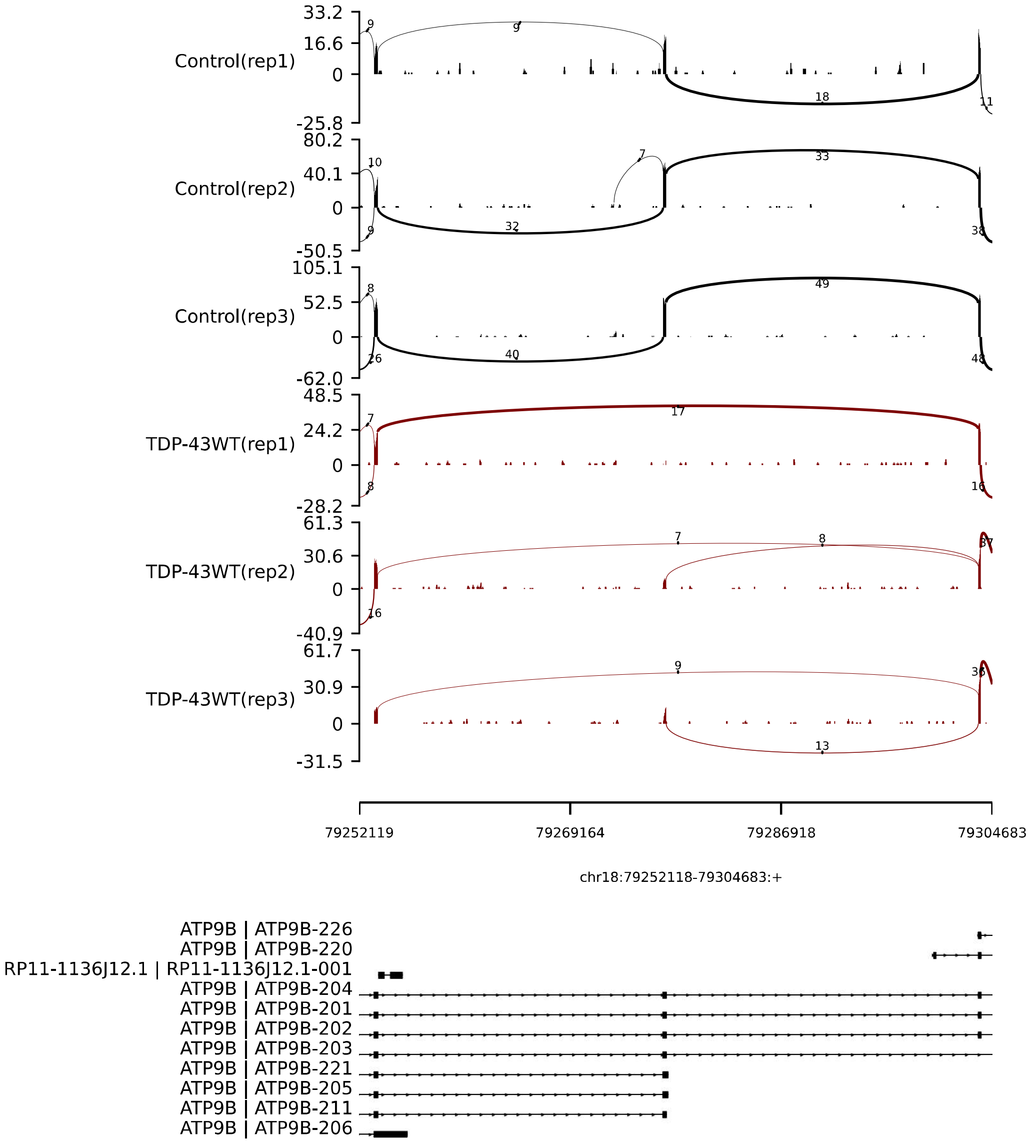

55. AMT

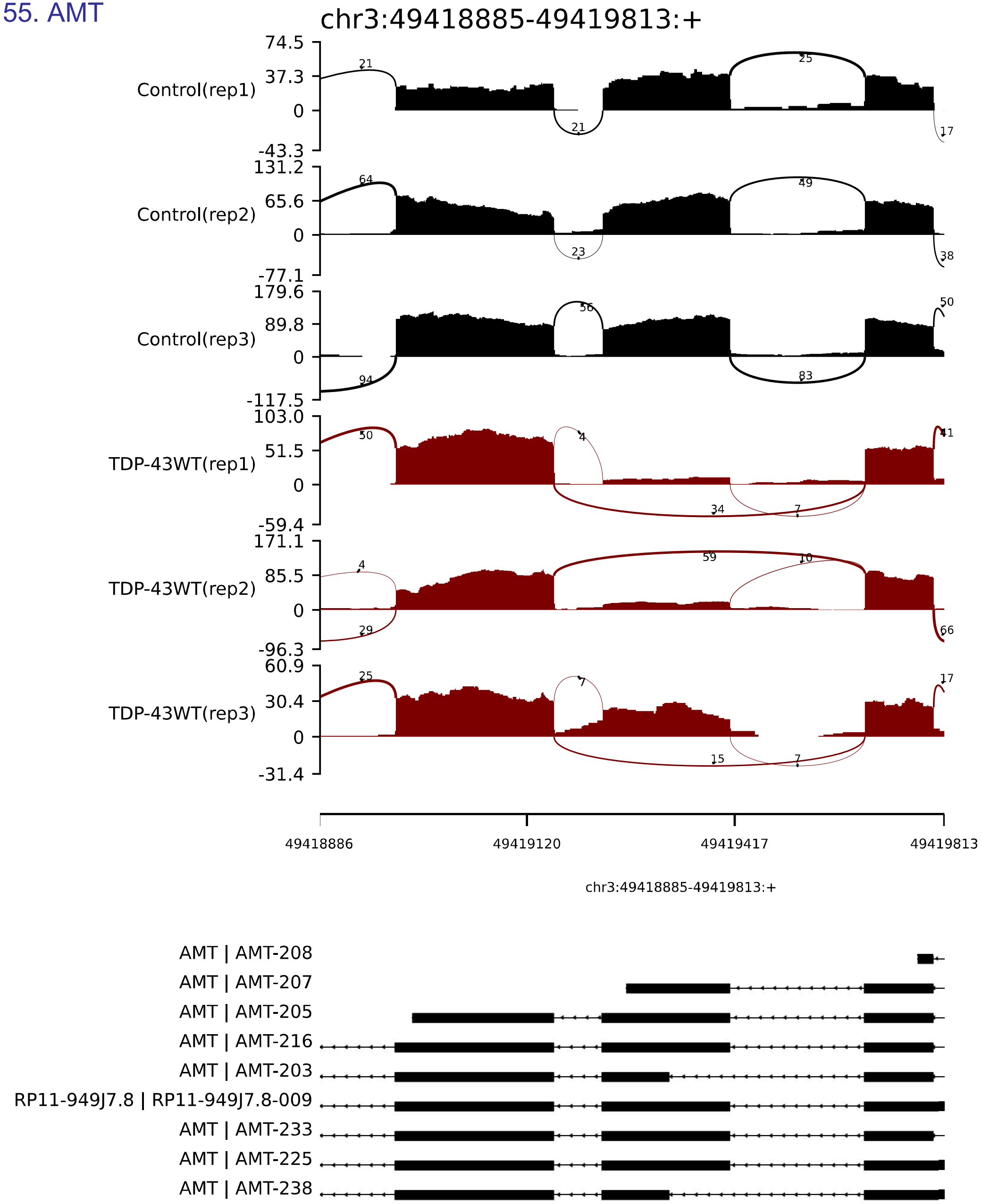

56. WRAP73

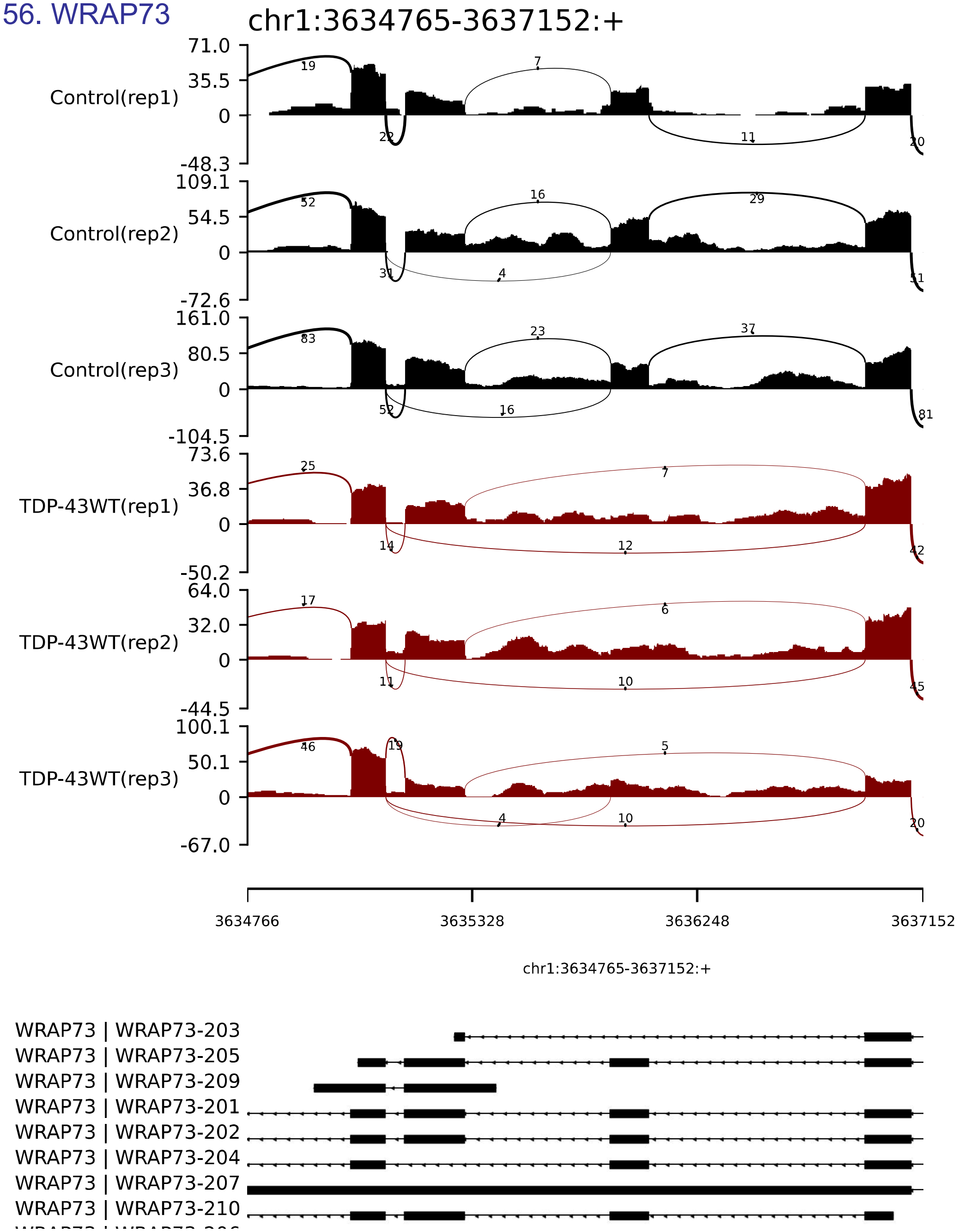

57. TLCD3A

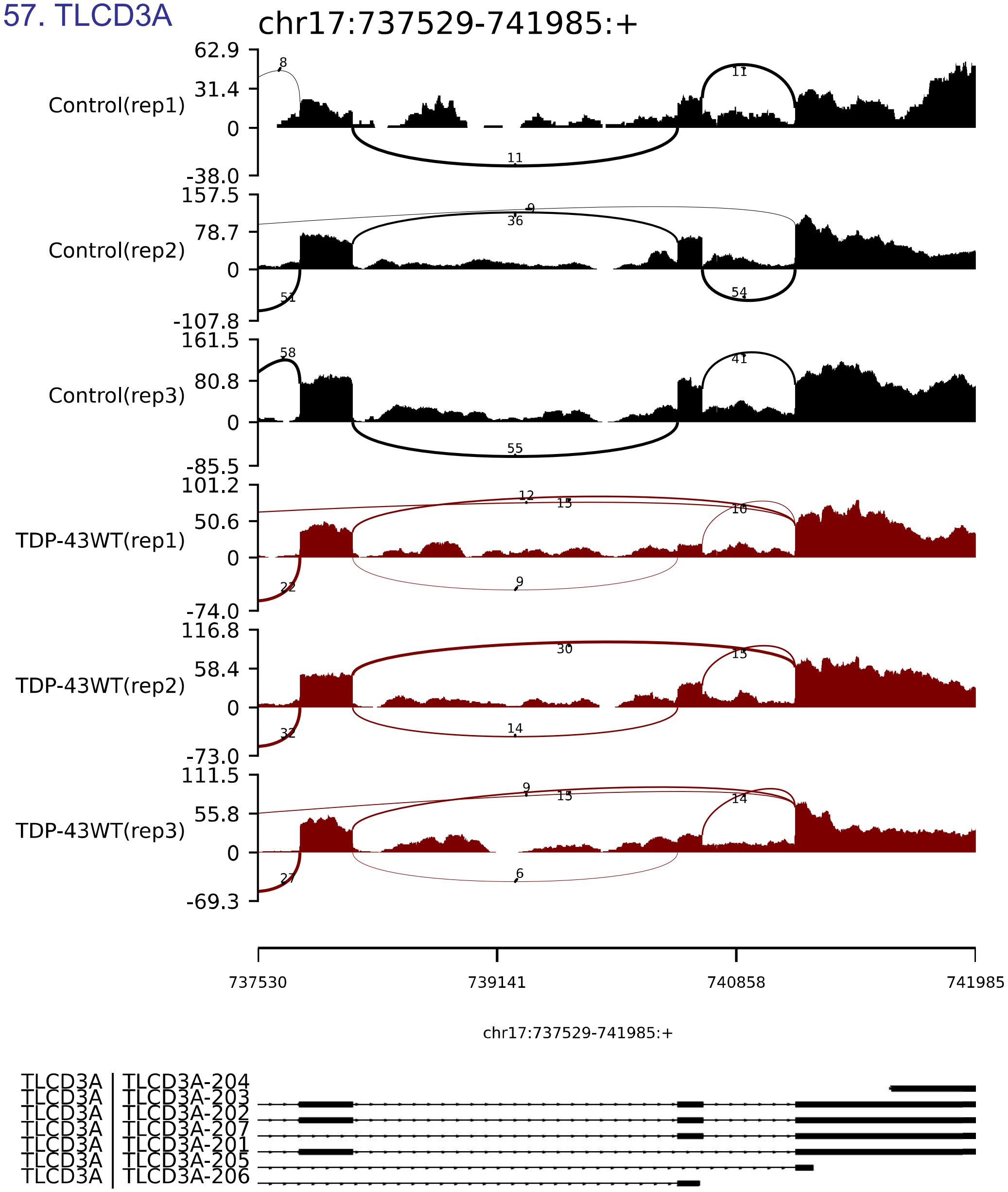

58. SCN3A

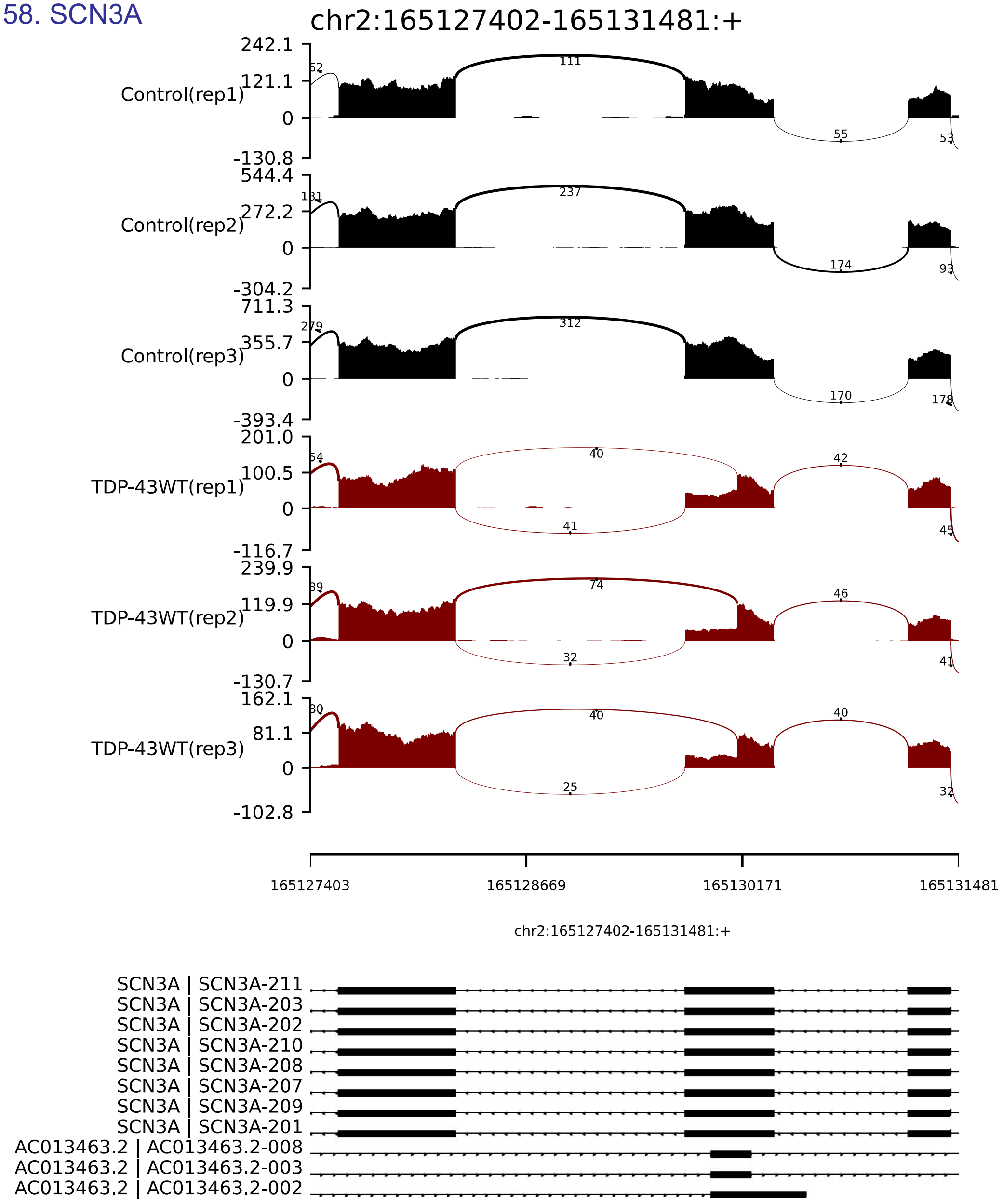

59. RNASET2

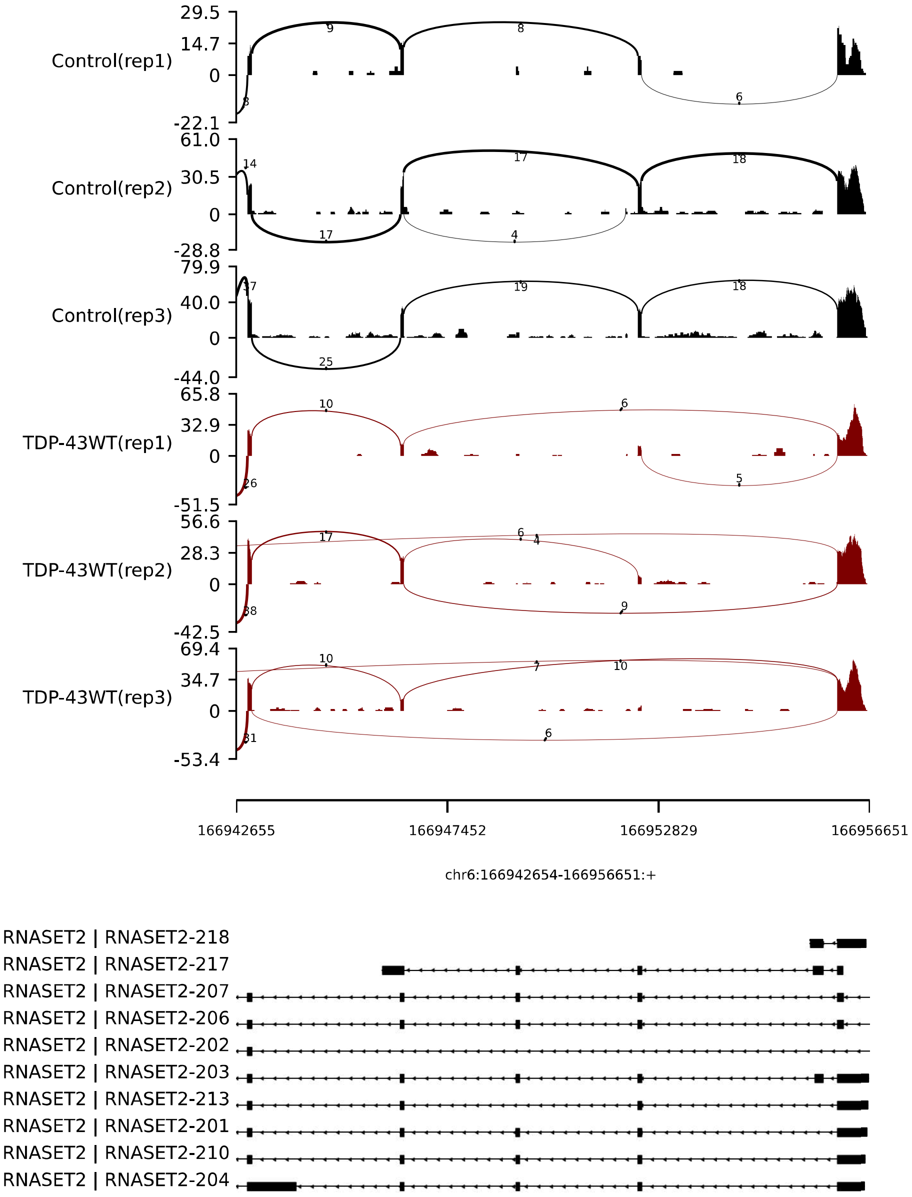

60. LINC00680-GUSBP4

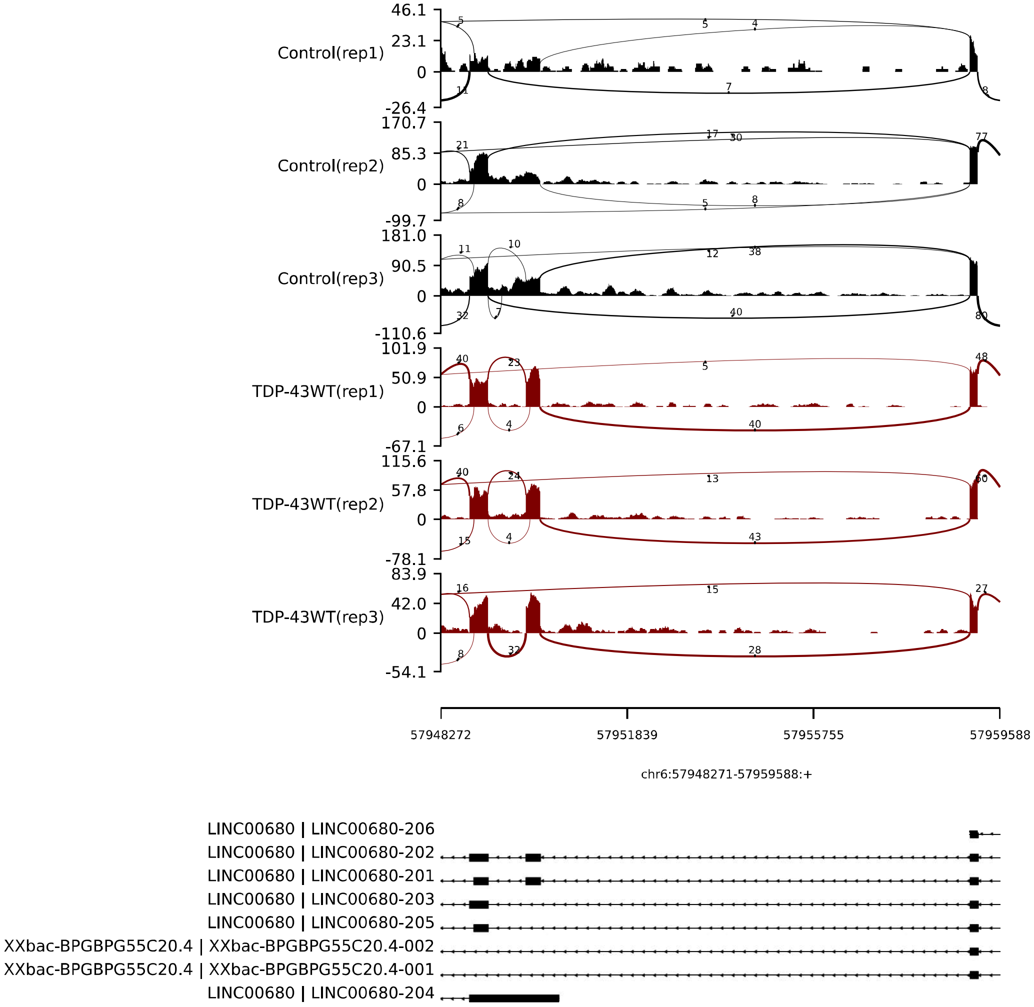

61. ITGB1

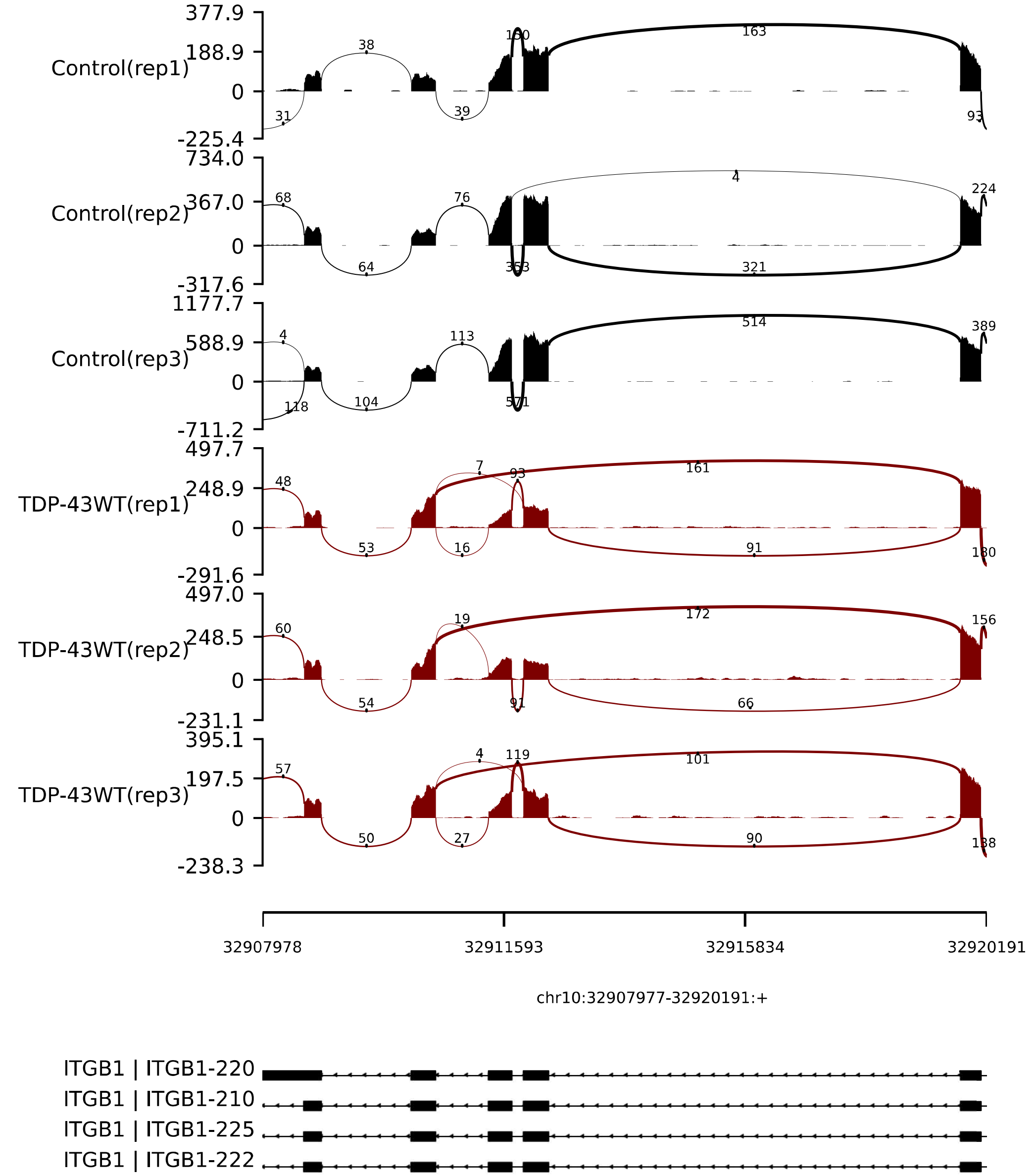

62. HECW1

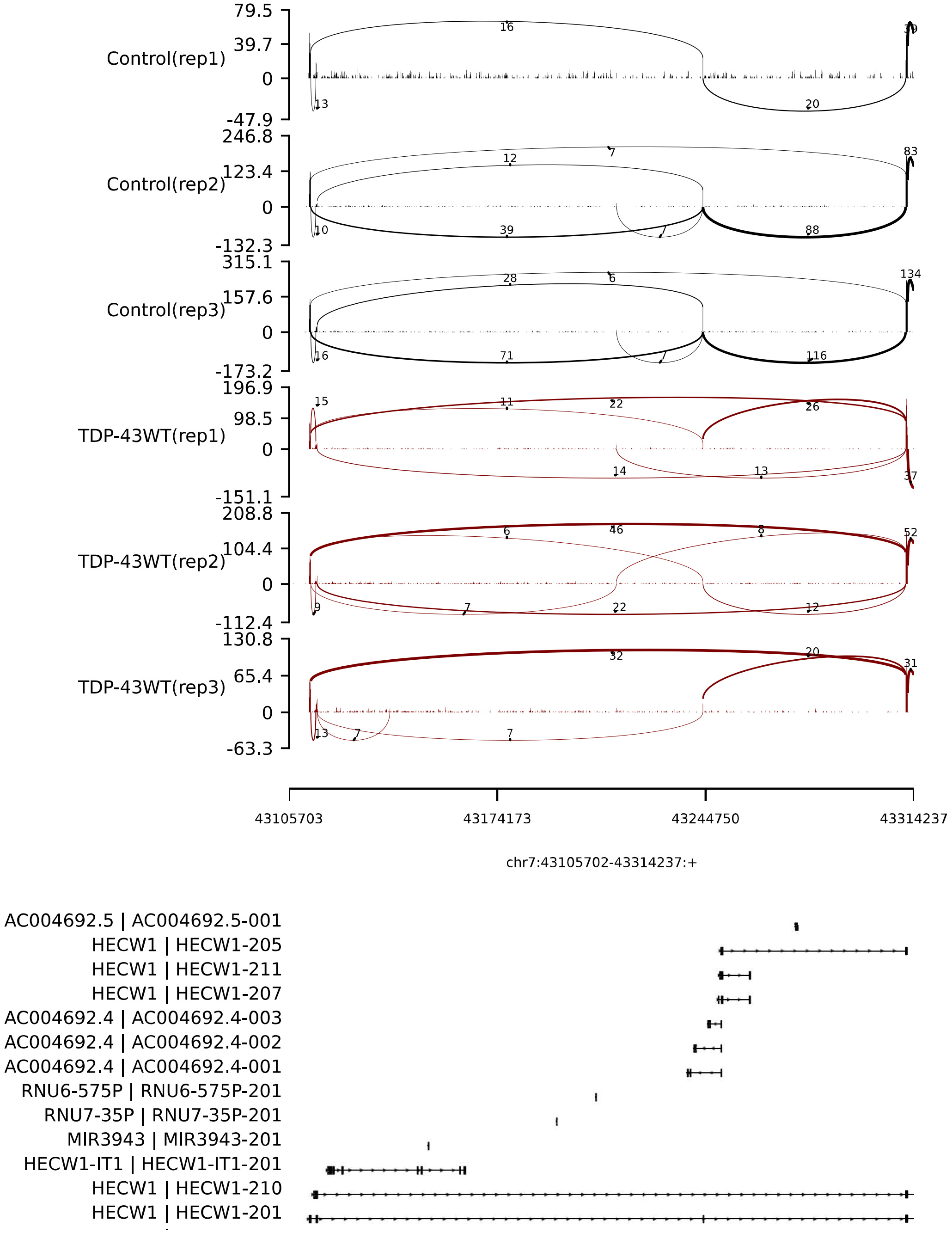

63. ENSG00000256591

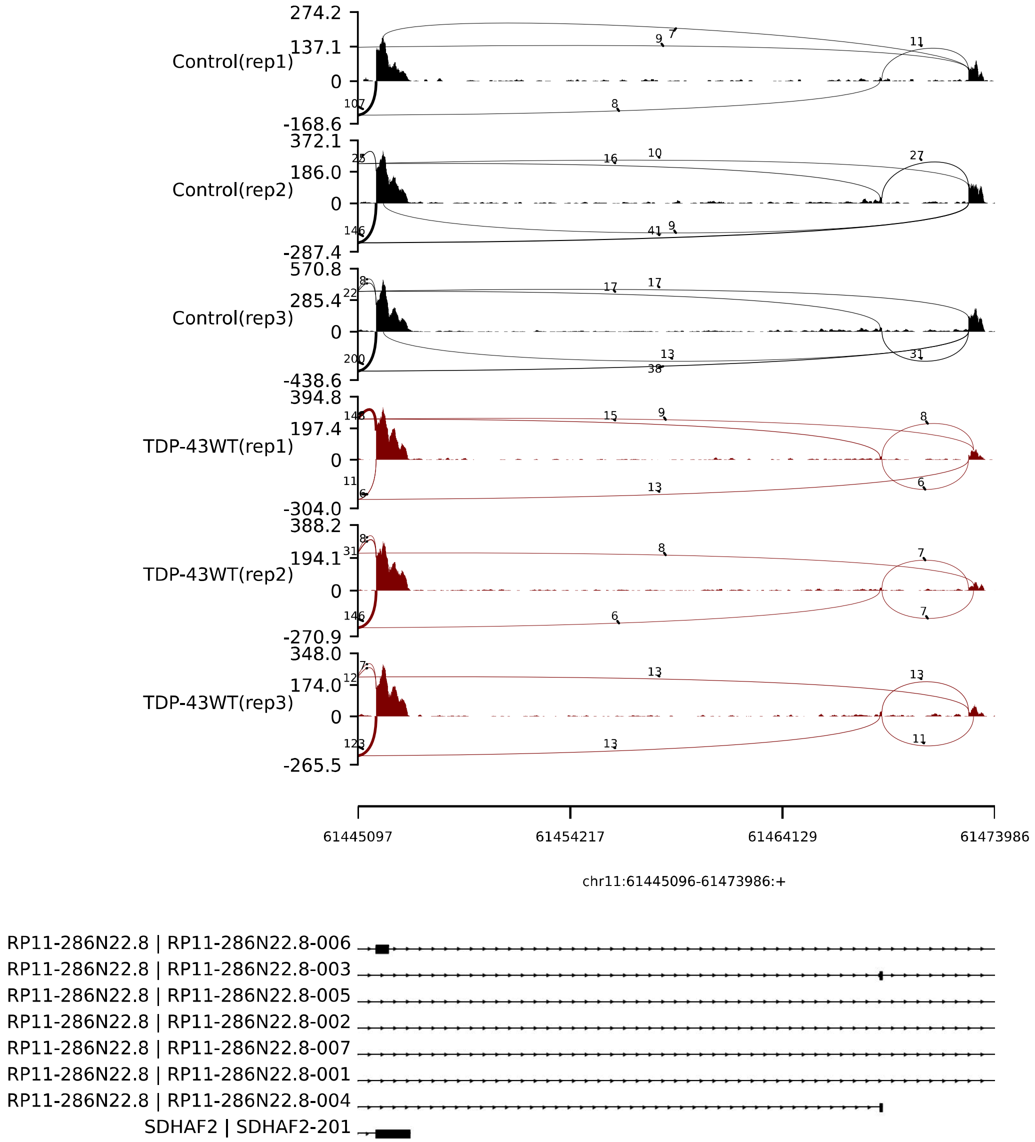

64. ELP1

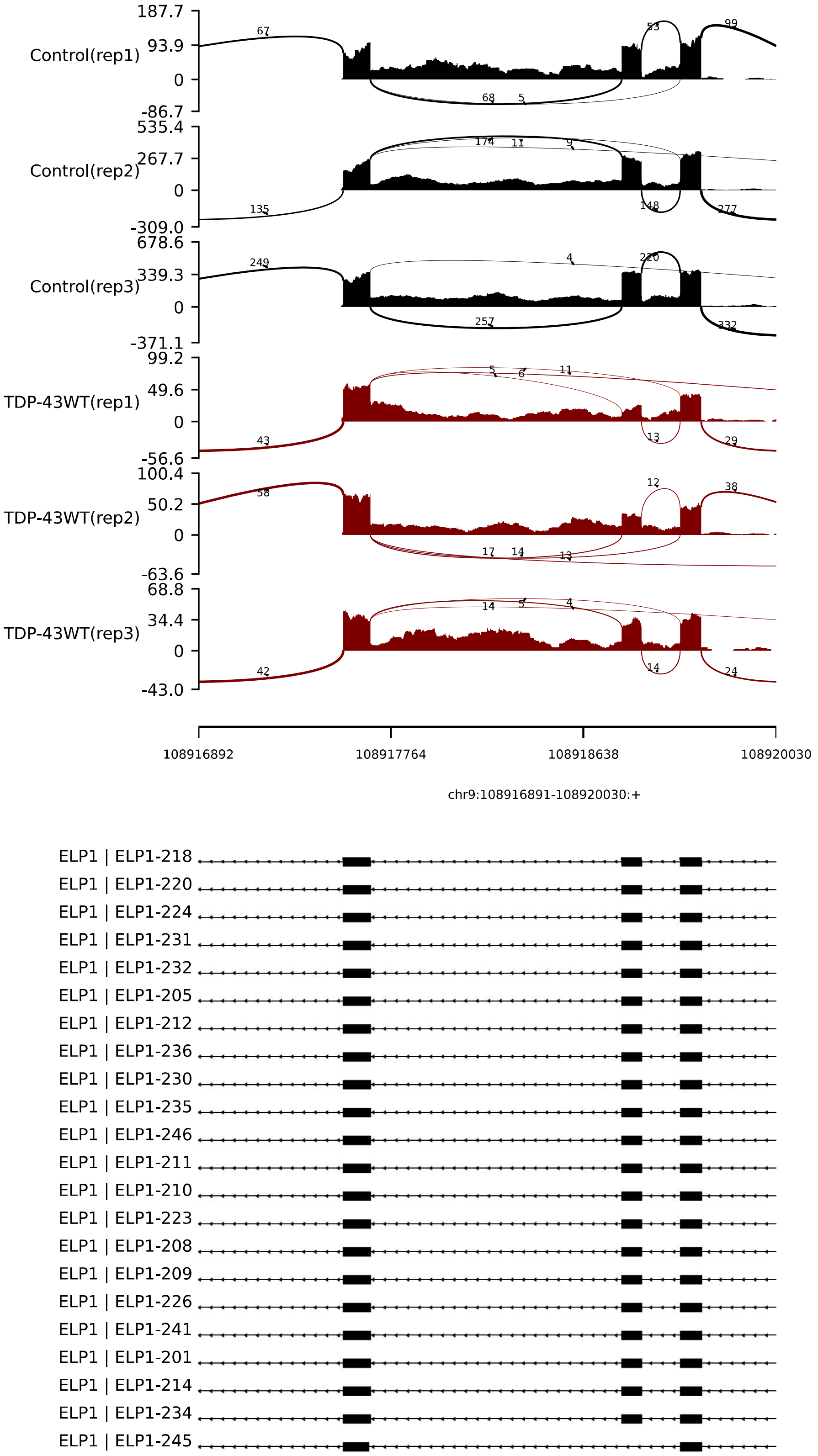

65. CSPP1

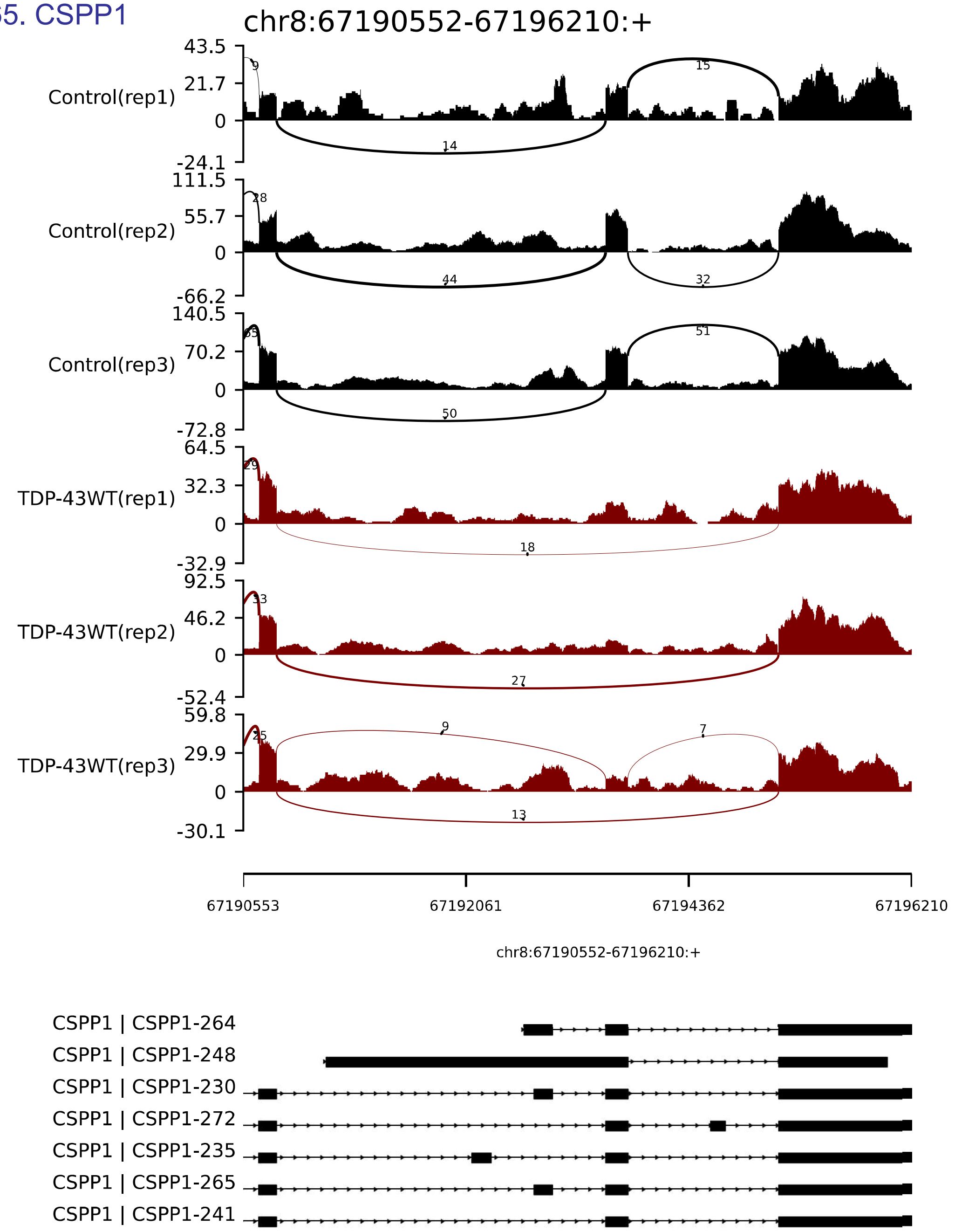

66. CCDC126

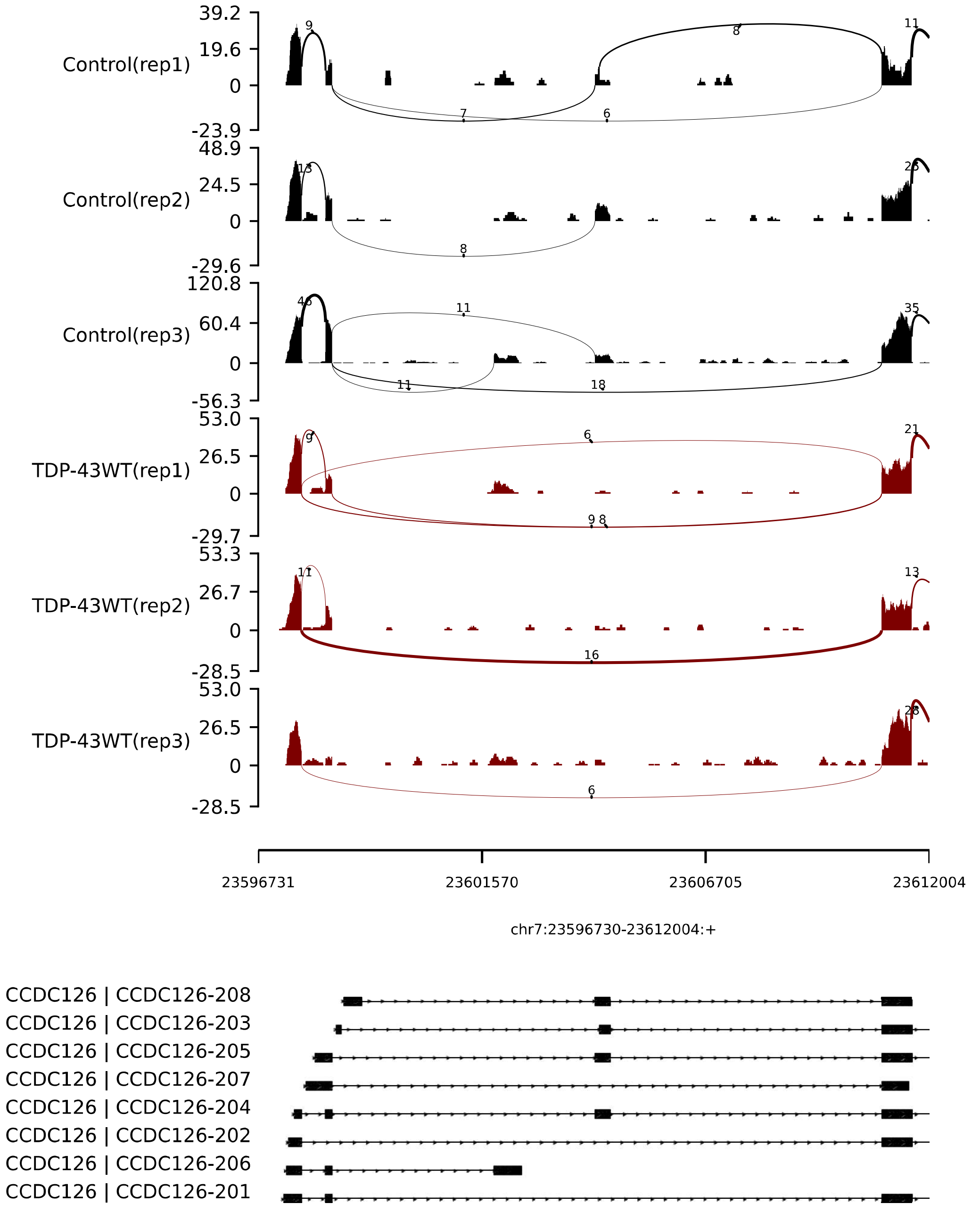

67. BRI3BP

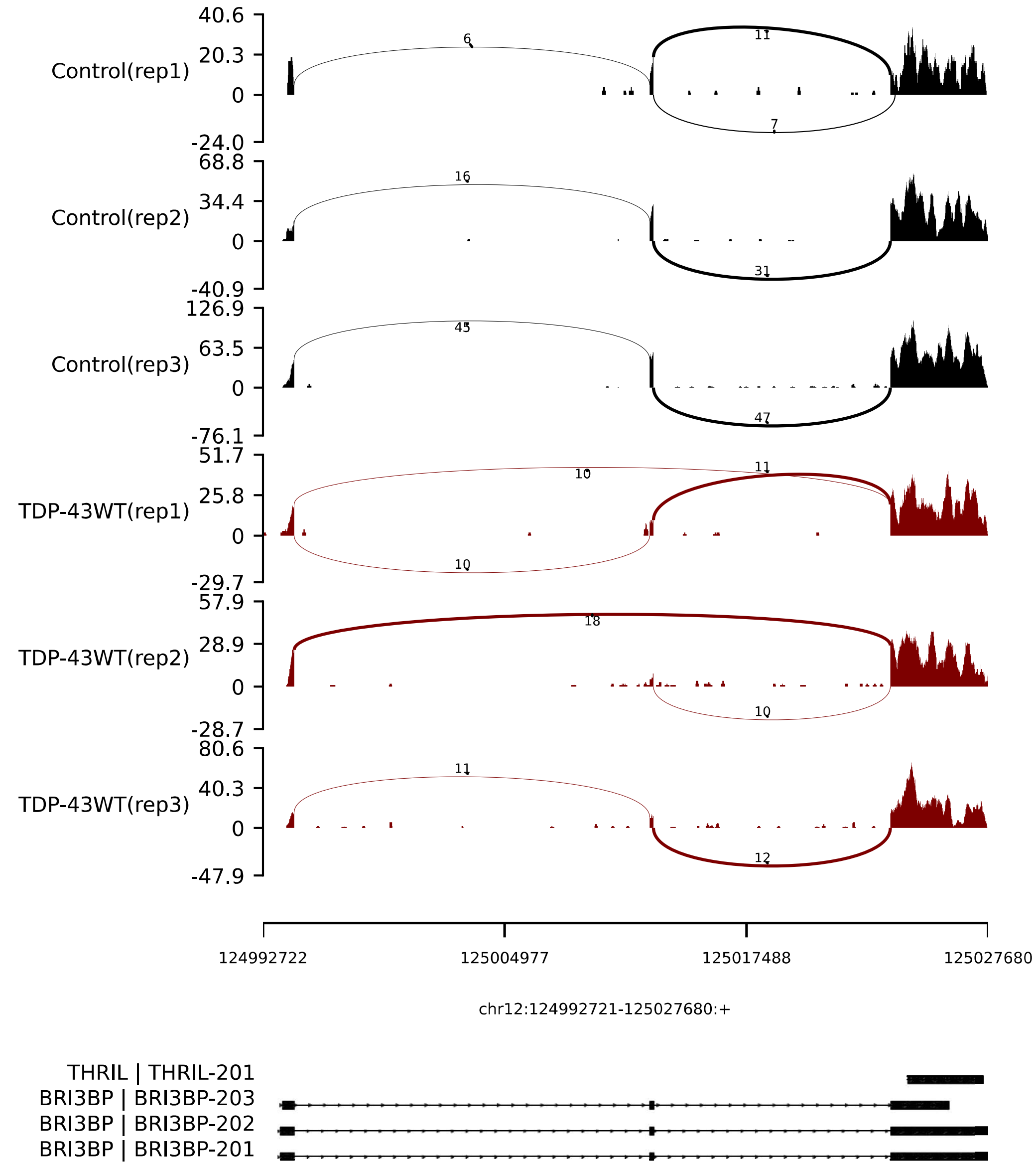

68. FANCG

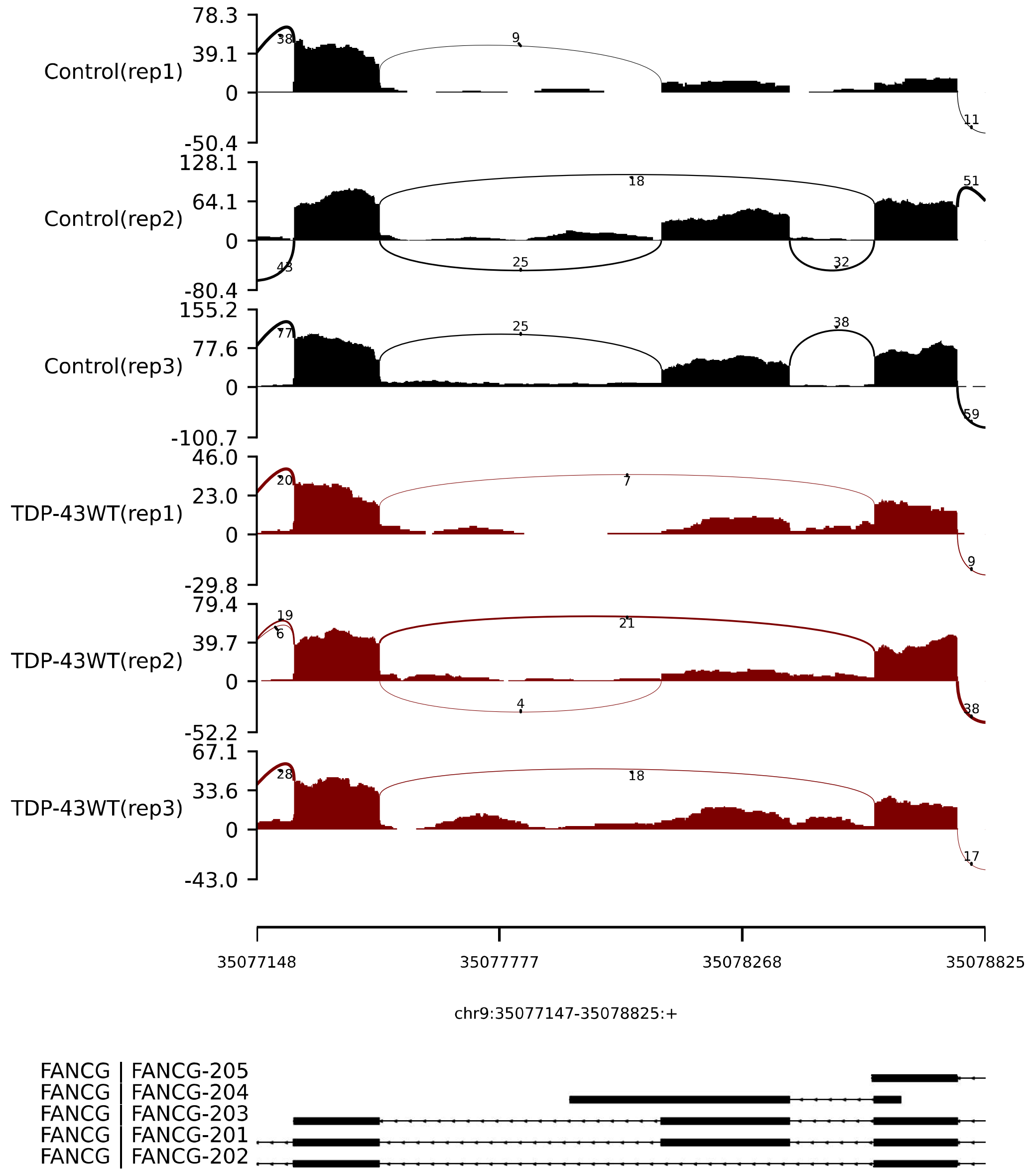

69. TSPAN11

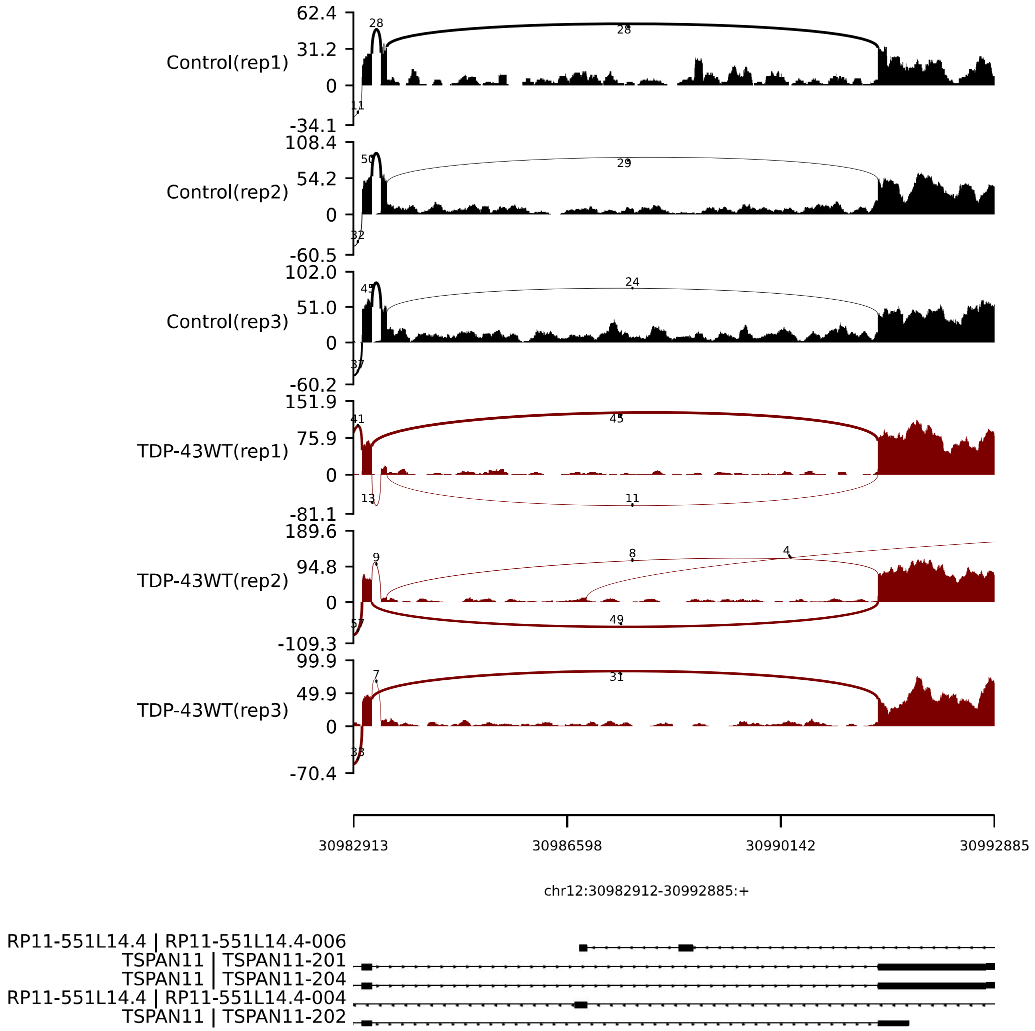

70. NHLRC3

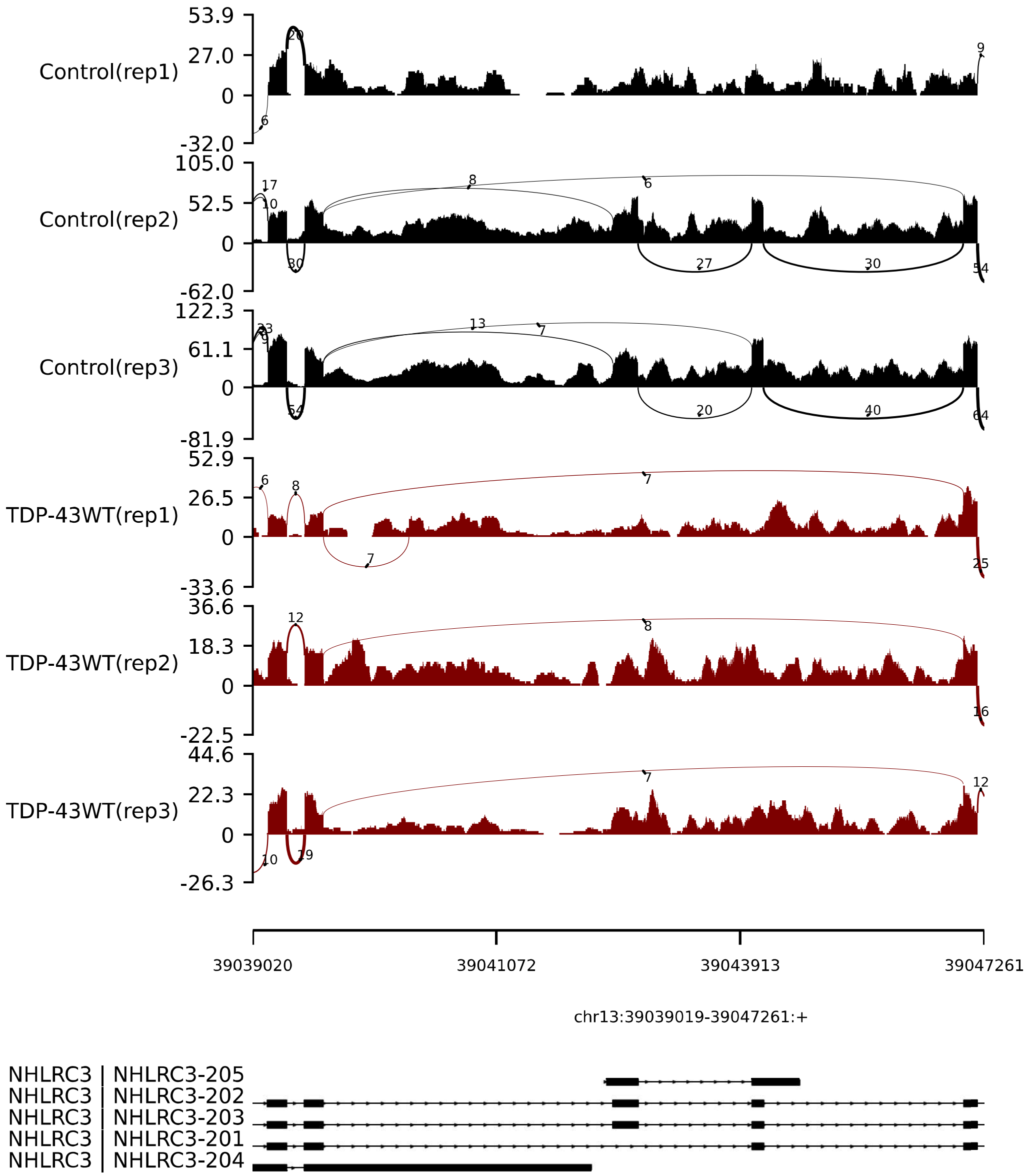

71. NEBL

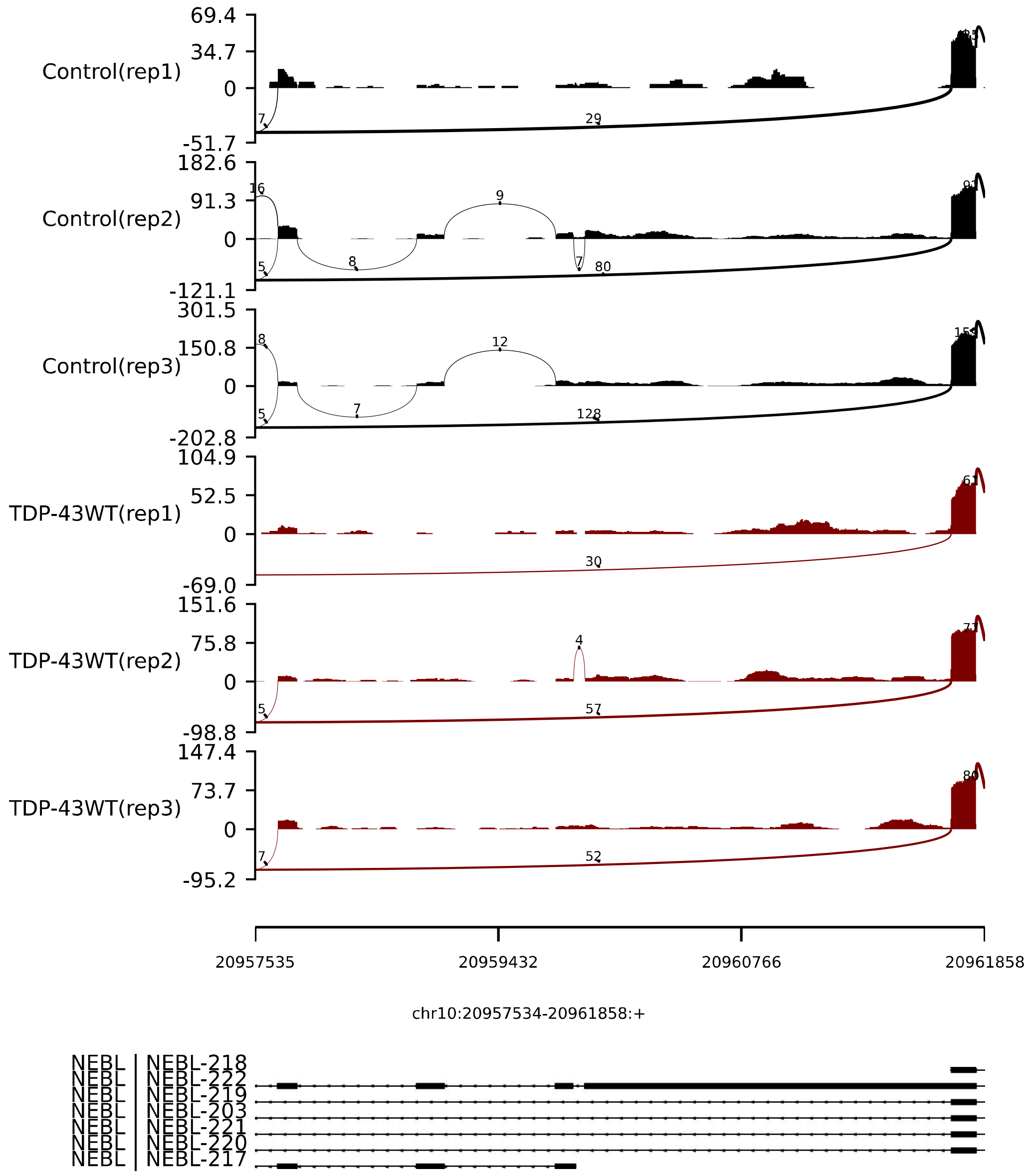

72. INTS10

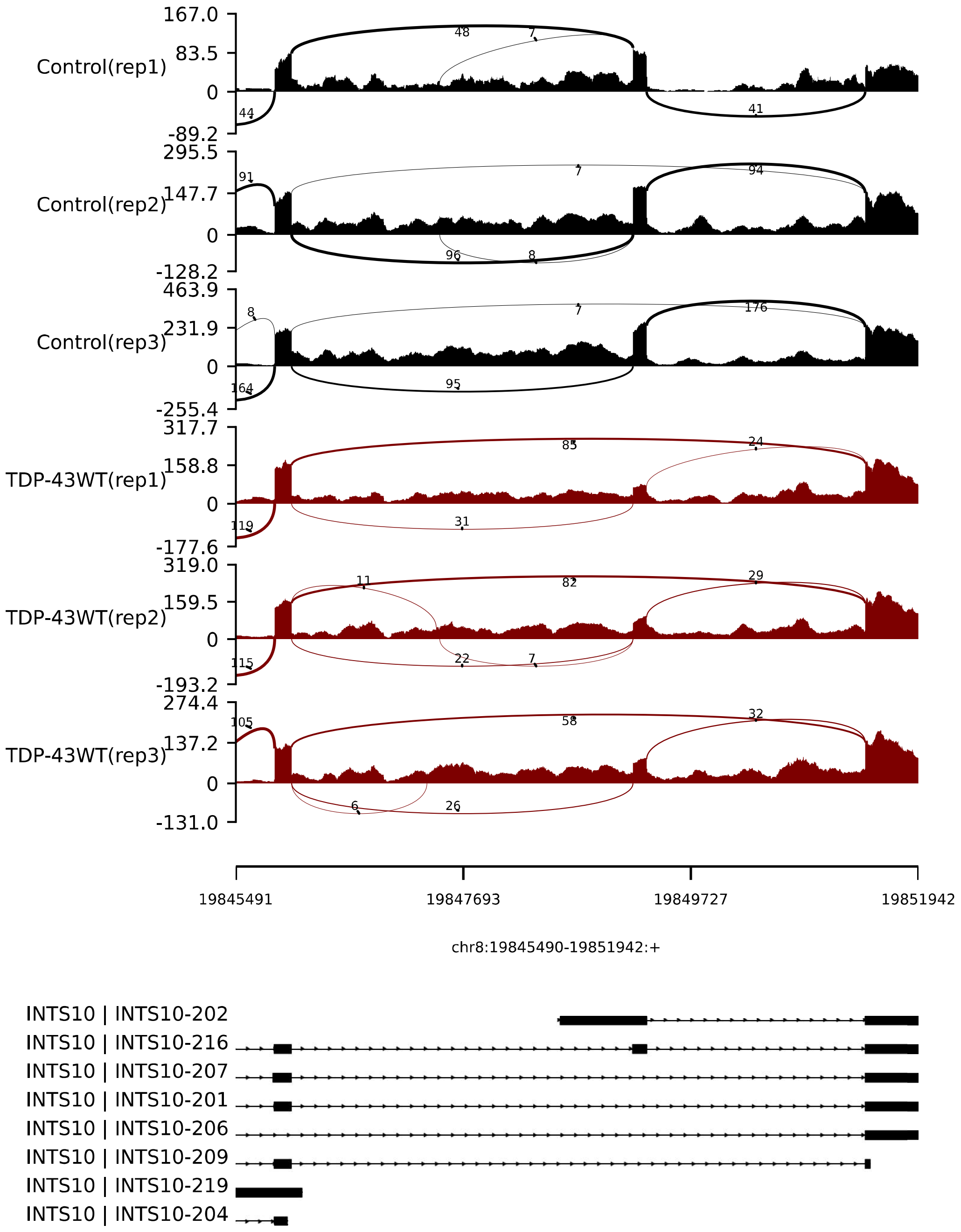

73. DPY19L1P1

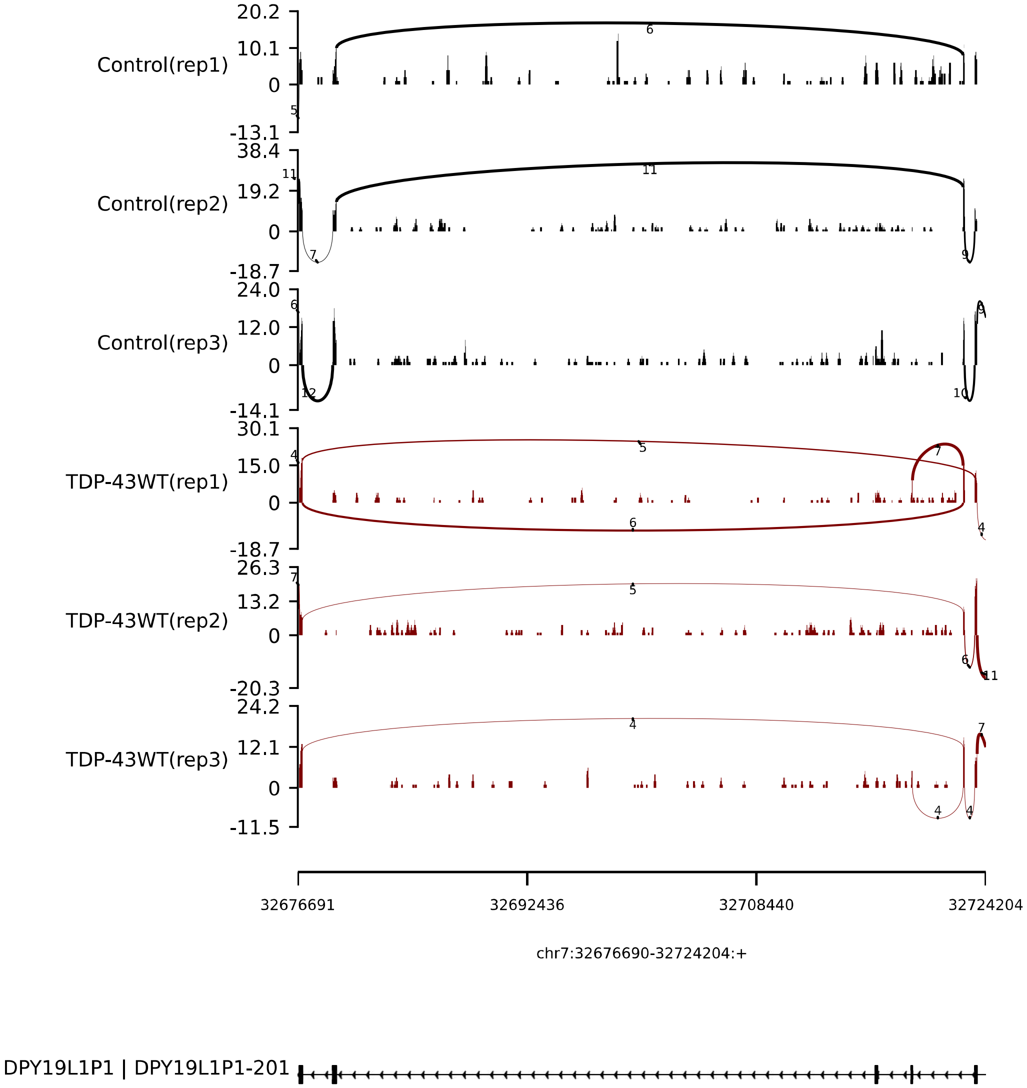

74. TTBK2

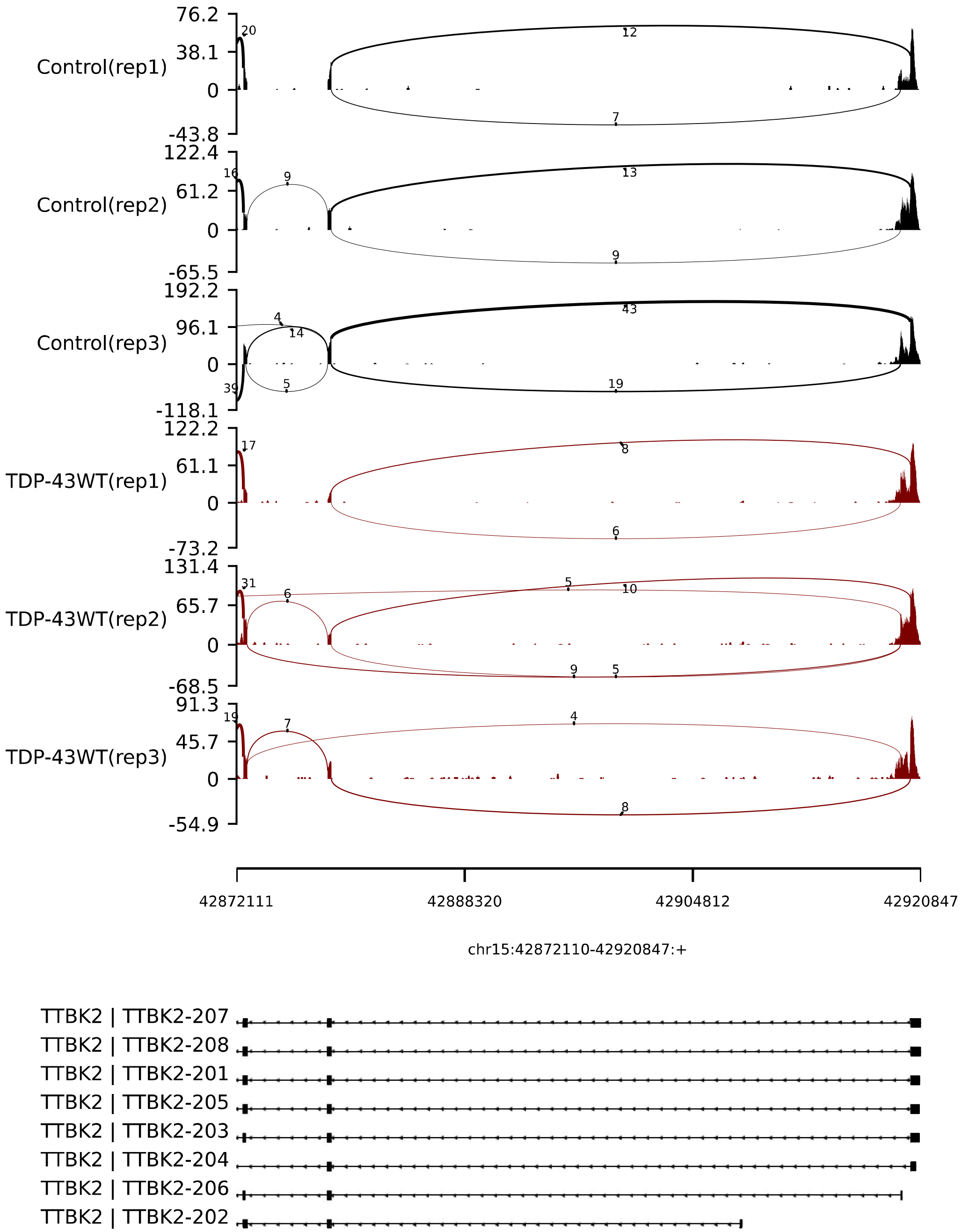

75. SPRK2

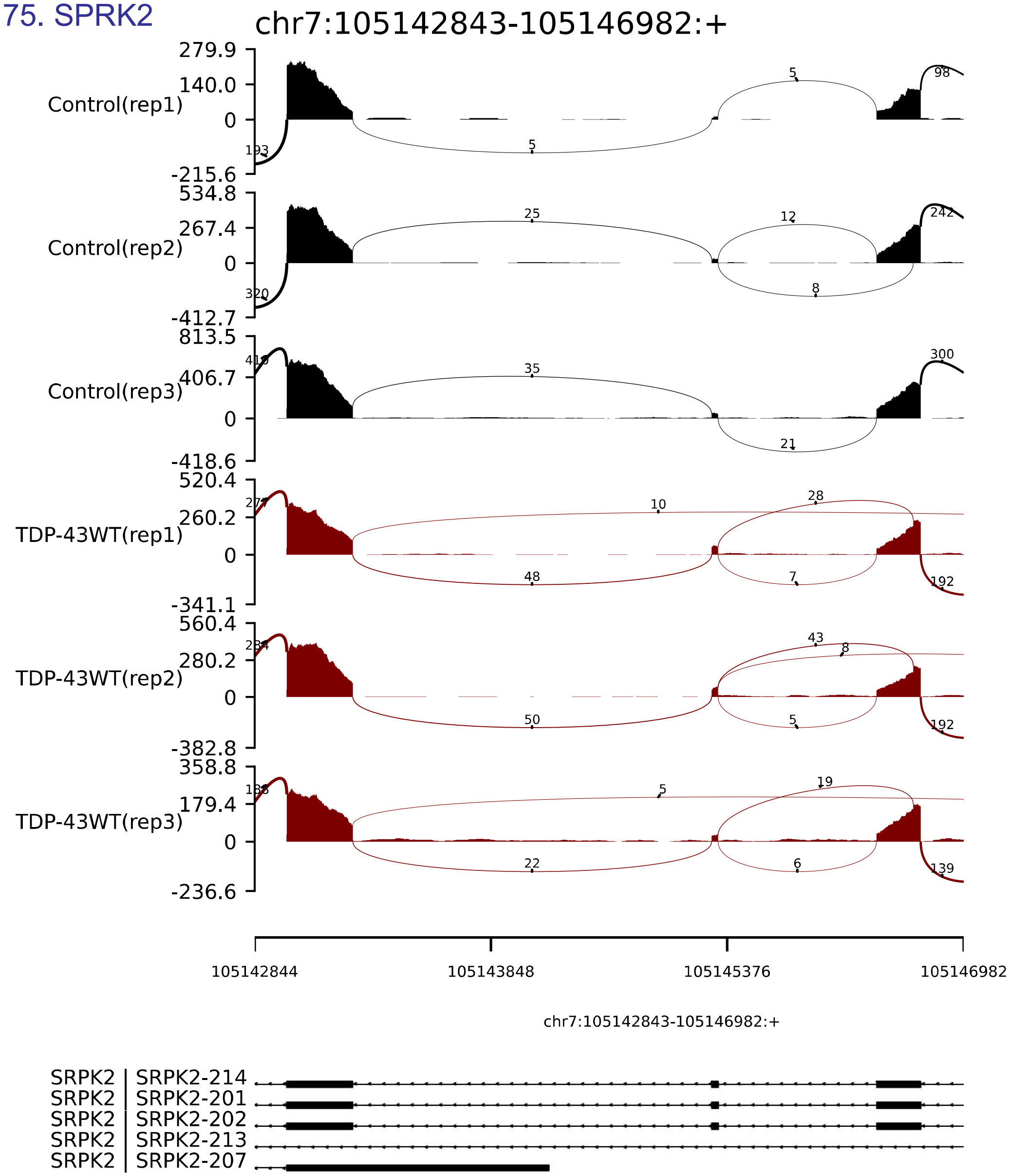

76. NQO2

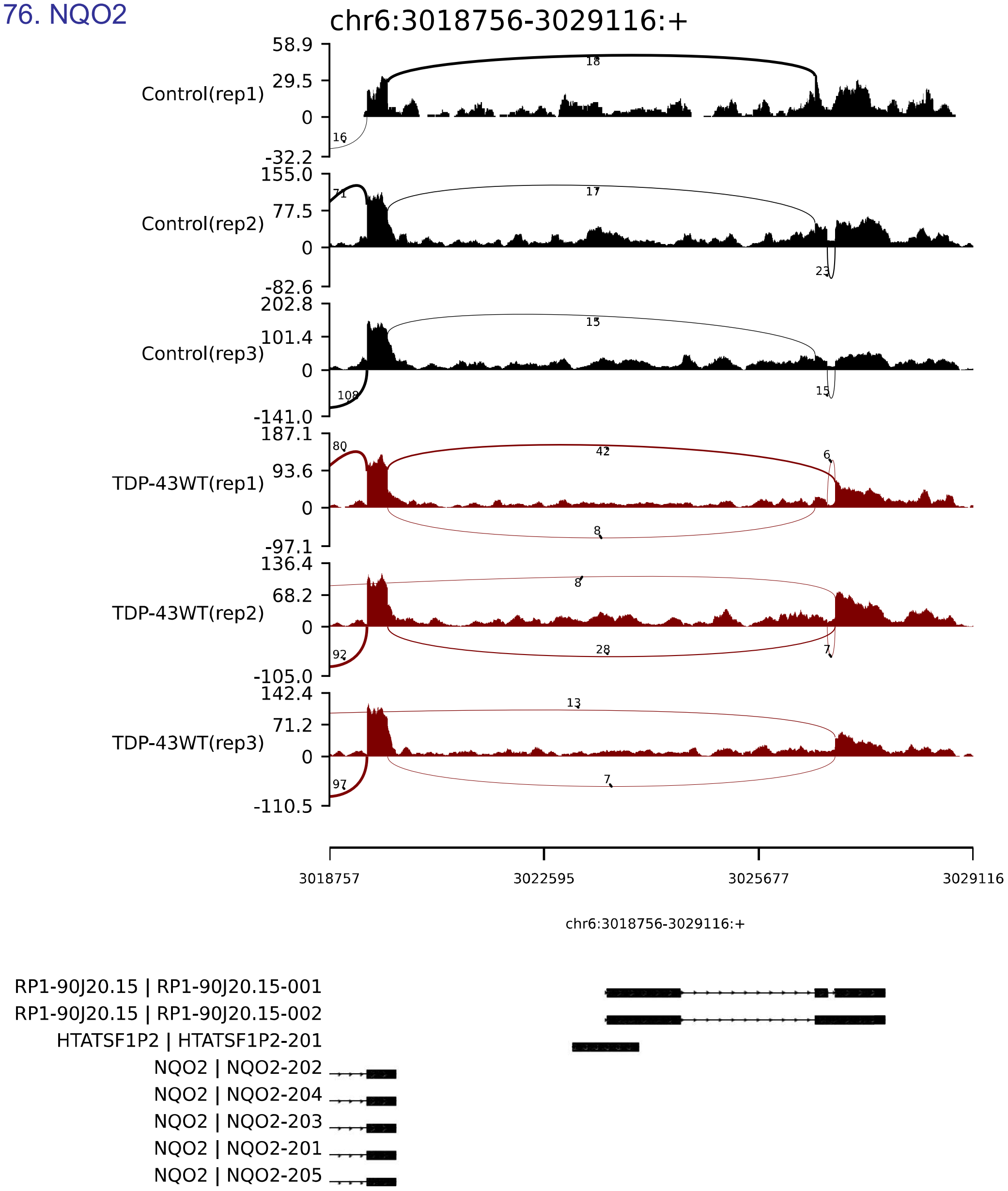

77. LGI4

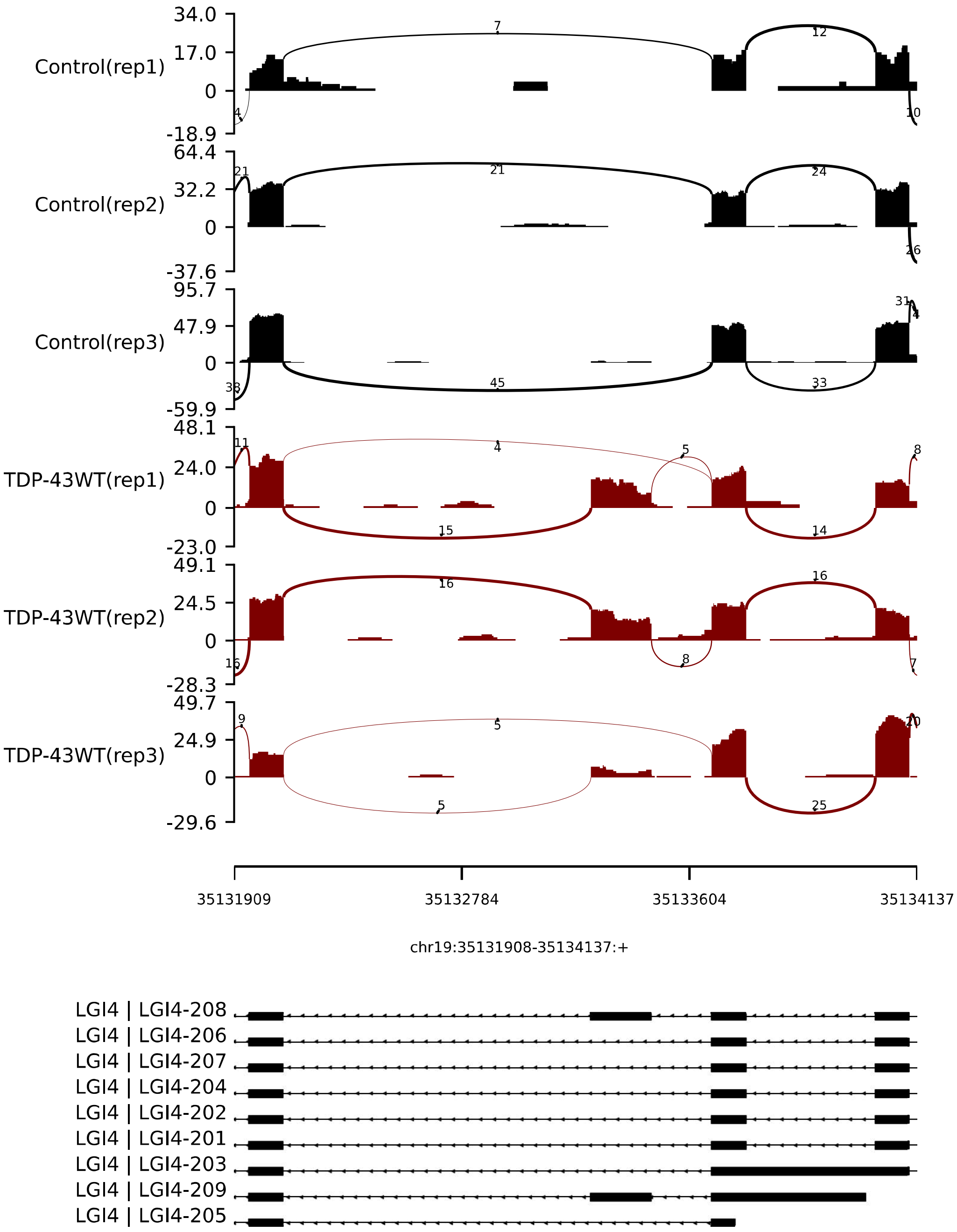

78. KCNMA1

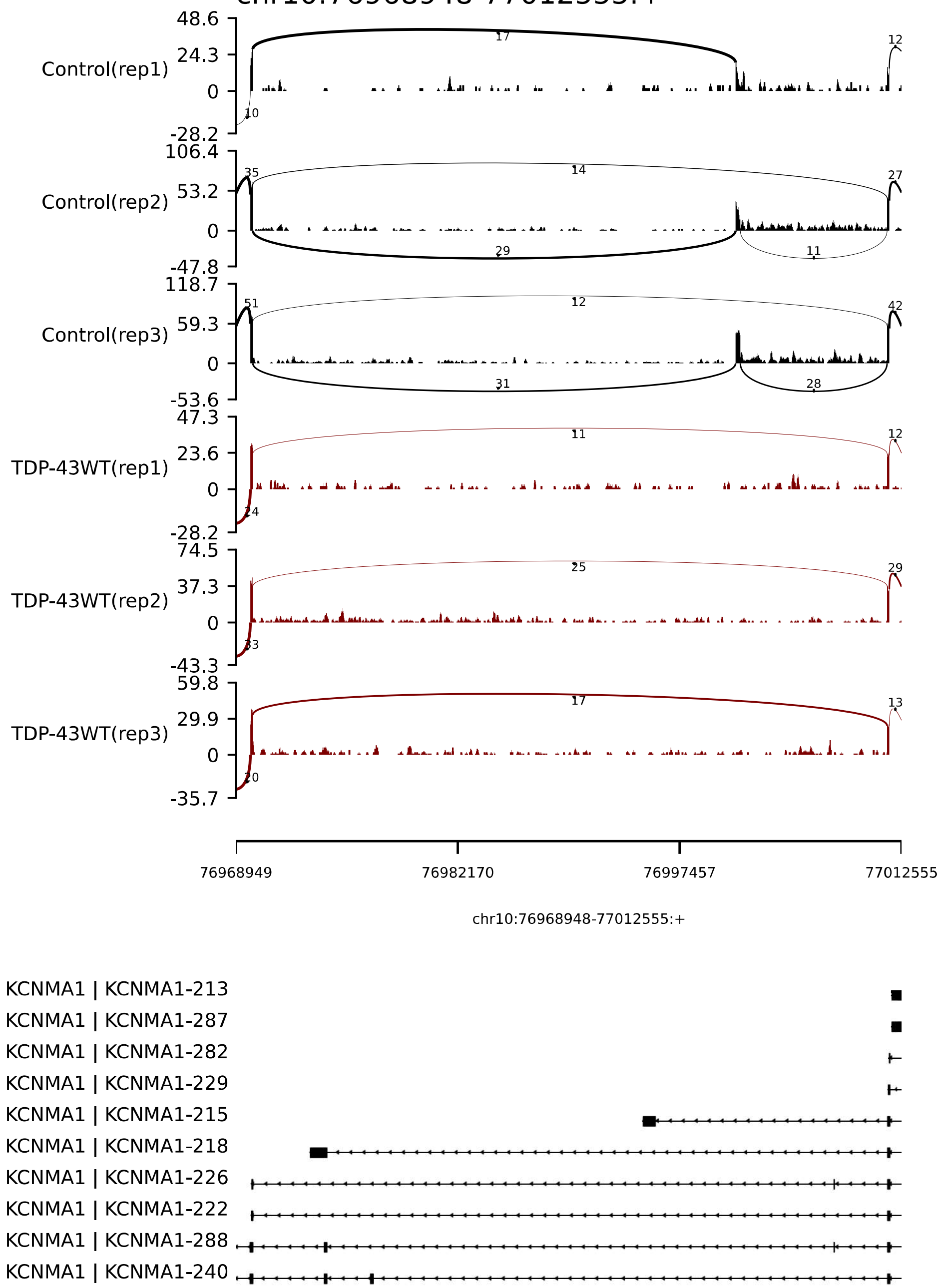

79. ADAM23

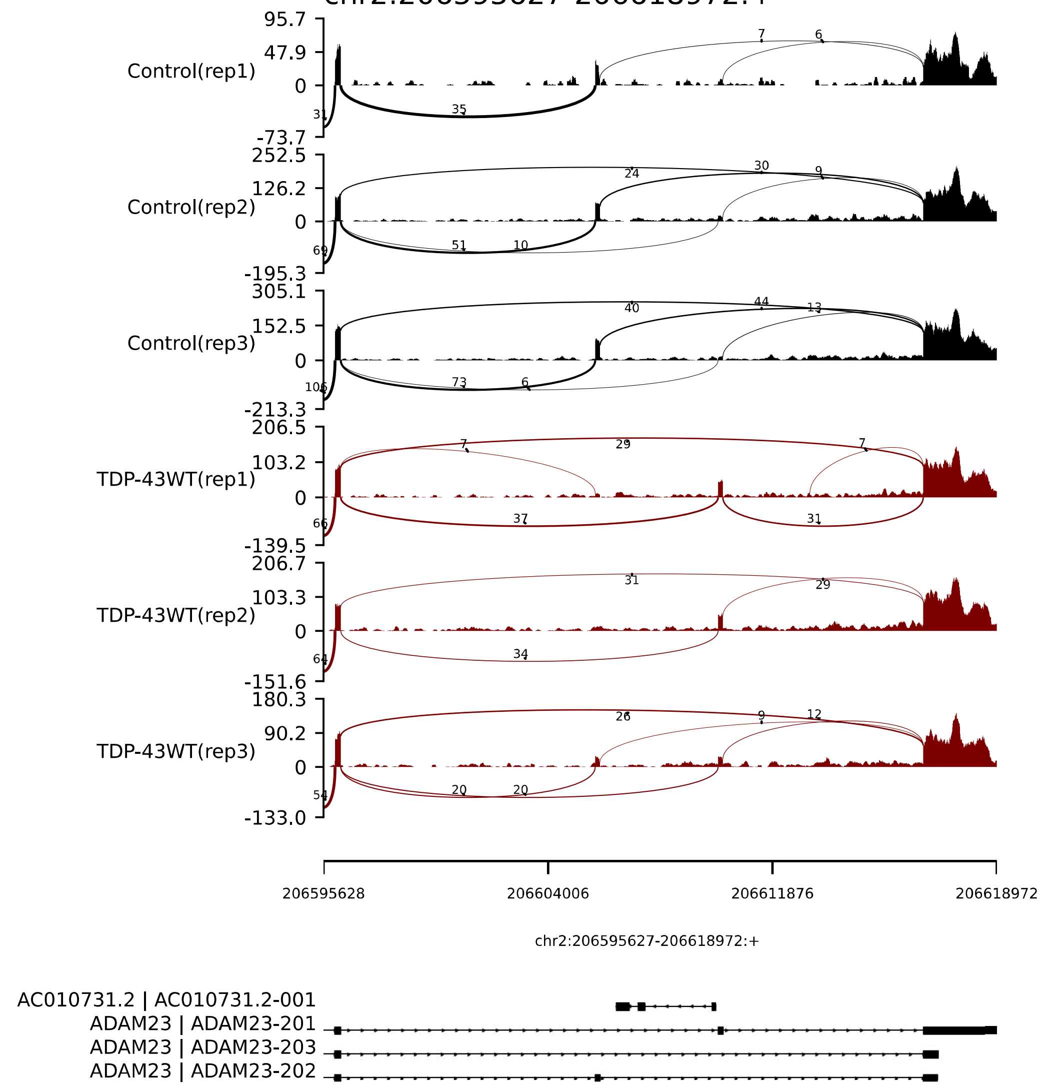

80. SUN1

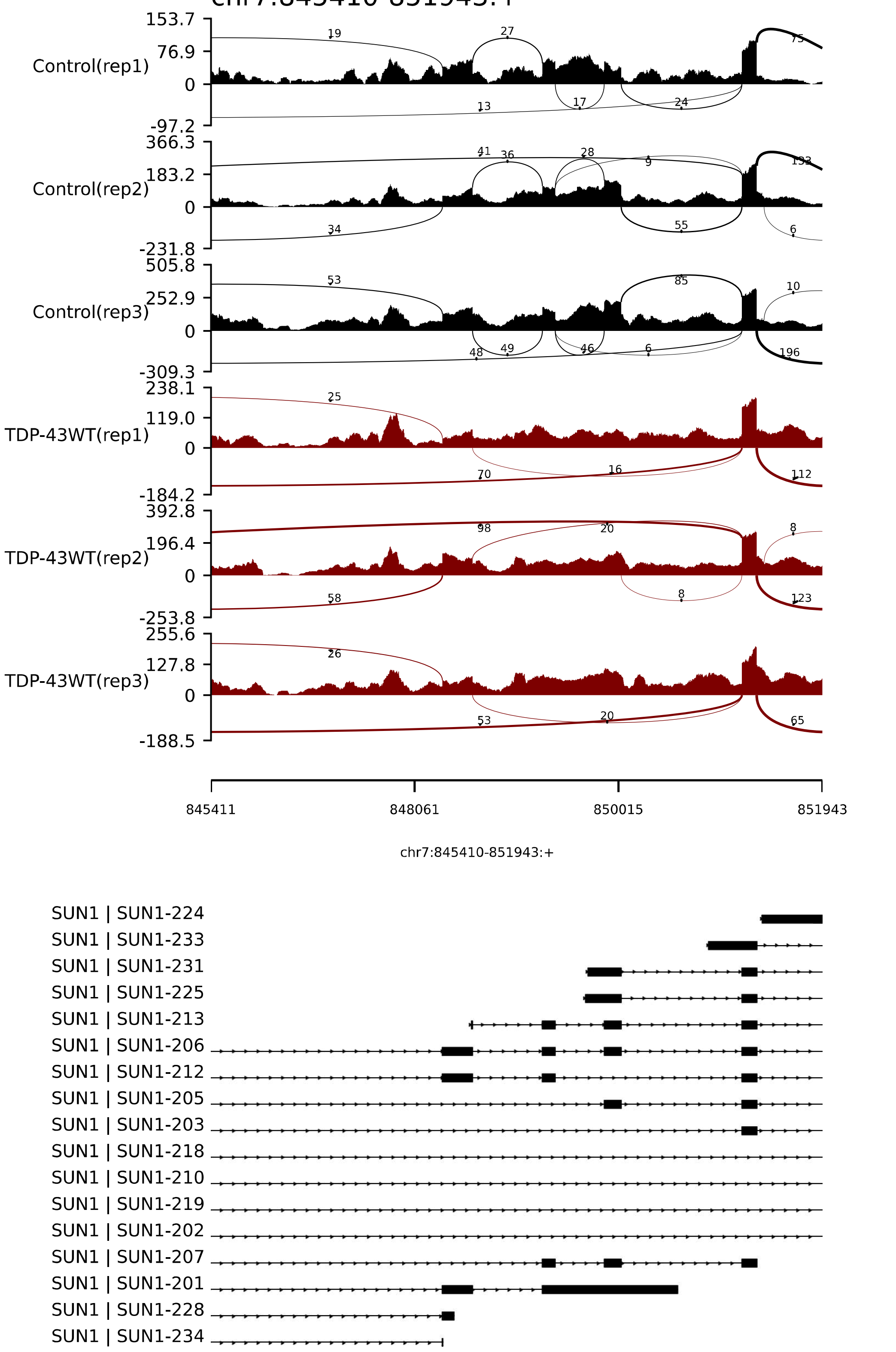

81. IL17RB

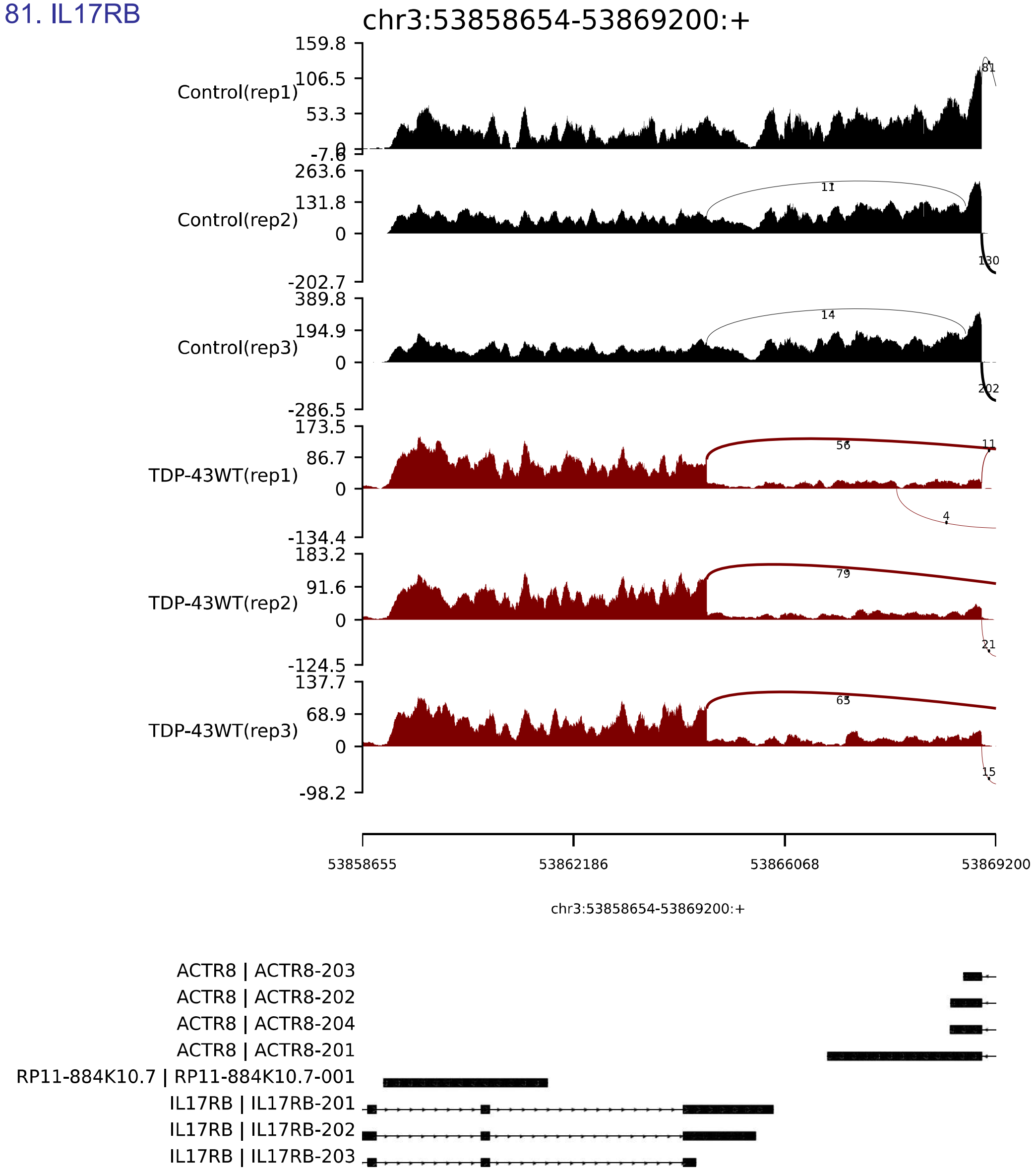

chr3:53858654-53869200:+
Control(rep1)
Control(rep2)
Control(rep3)
TDP-43WT(rep1)
TDP-43WT(rep2)
TDP-43WT(rep3)
53858655
53862186
53866068
53869200
chr3:53858654-53869200:+
ACTR8 | ACTR8-203
ACTR8 | ACTR8-202
ACTR8 | ACTR8-204
ACTR8 | ACTR8-201
RP11-884K10.7 | RP11-884K10.7-001
IL17RB | IL17RB-201
IL17RB | IL17RB-202
IL17RB | IL17RB-203
