# Supplementary material for: Elevated nuclear TDP-43 induces constitutive exon skipping
Source: Mol Neurodegener. 2024 Jun 9;19:45. doi: 10.1186/s13024-024-00732-w (PMC11163724; doi:10.1186/s13024-024-00732-w)

1. Arhgap44

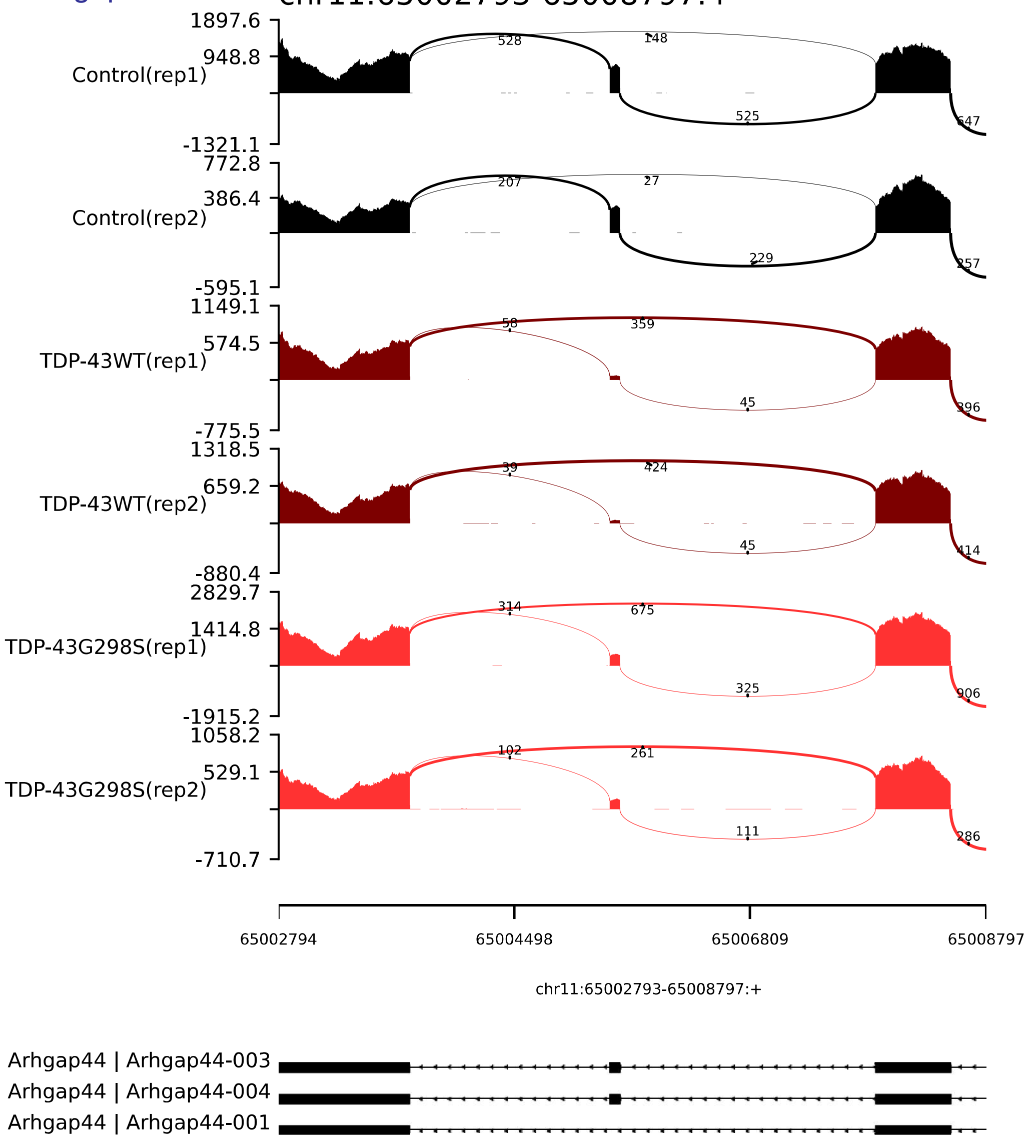

2. Ddi2

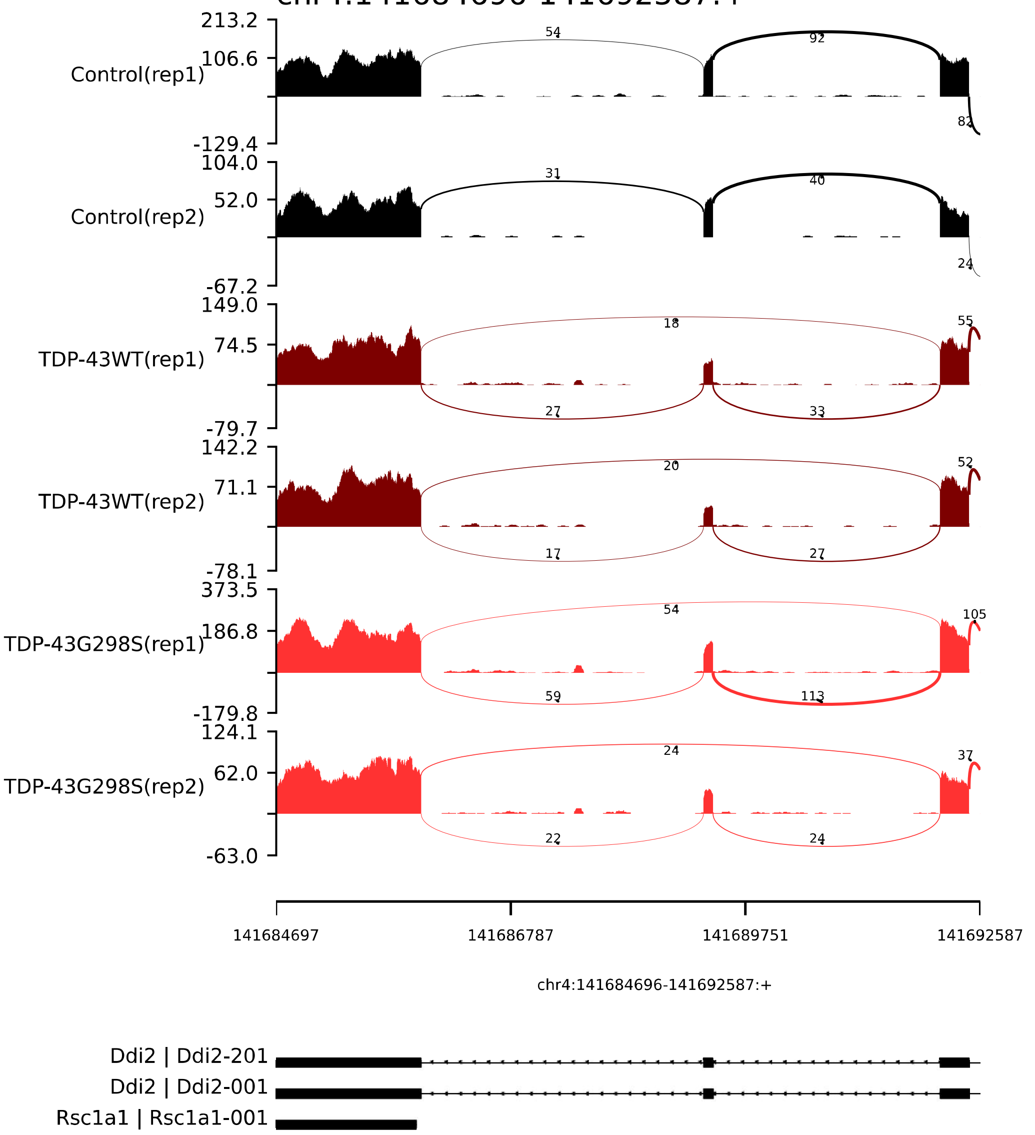

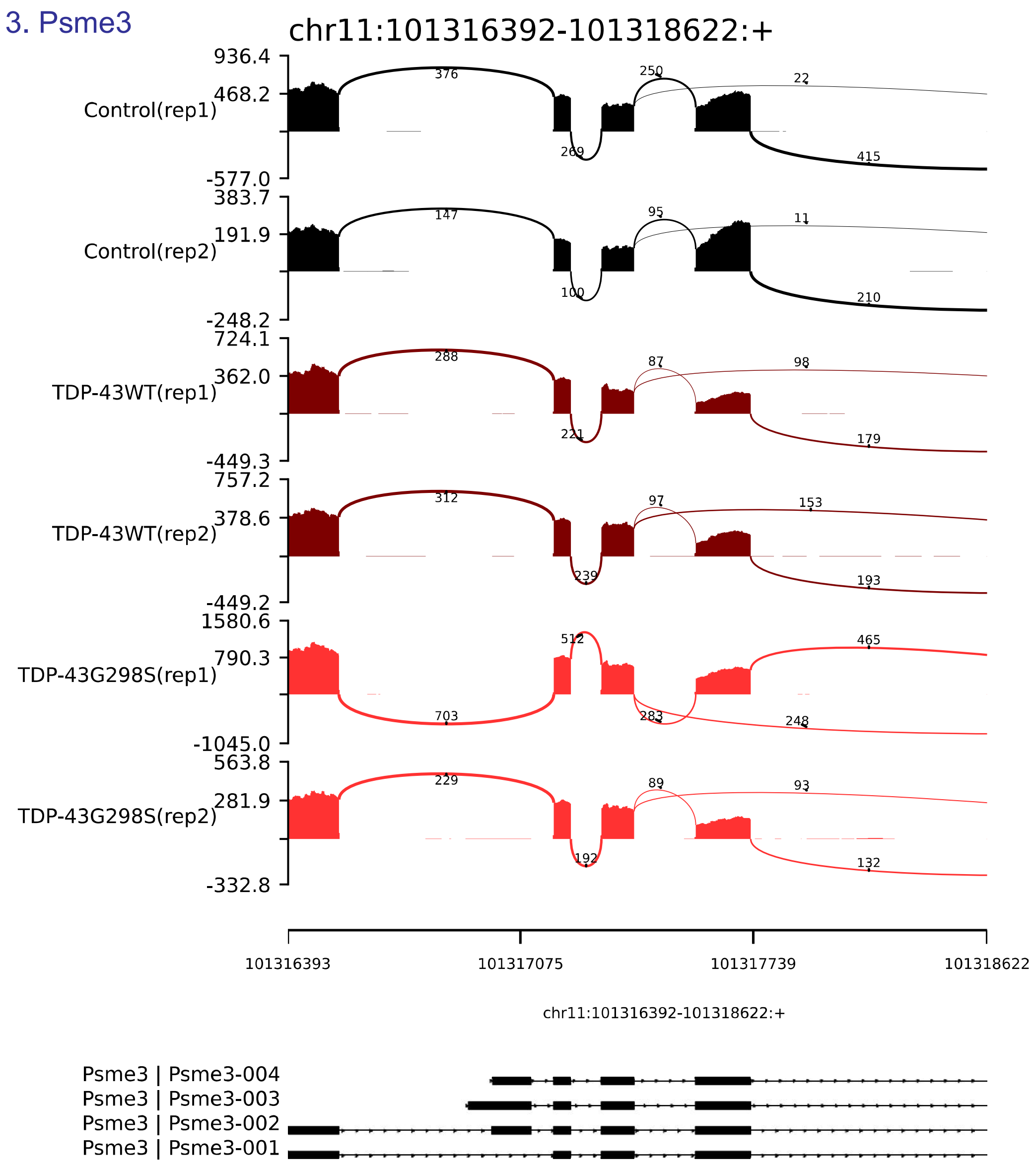
3. Psme3
chr11:101316392-101318622:+
936.4
468.2
-577.0
Control(rep1)
383.7
191.9
-248.2
Control(rep2)
724.1
362.0
-449.3
TDP-43WT(rep1)
757.2
378.6
-449.2
TDP-43WT(rep2)
1580.6
790.3
-1045.0
TDP-43G298S(rep1)
563.8
281.9
-332.8
TDP-43G298S(rep2)
101316393
101317075
101317739
101318622
chr11:101316392-101318622:+
Psme3 | Psme3-004
Psme3 | Psme3-003
Psme3 | Psme3-002
Psme3 | Psme3-001

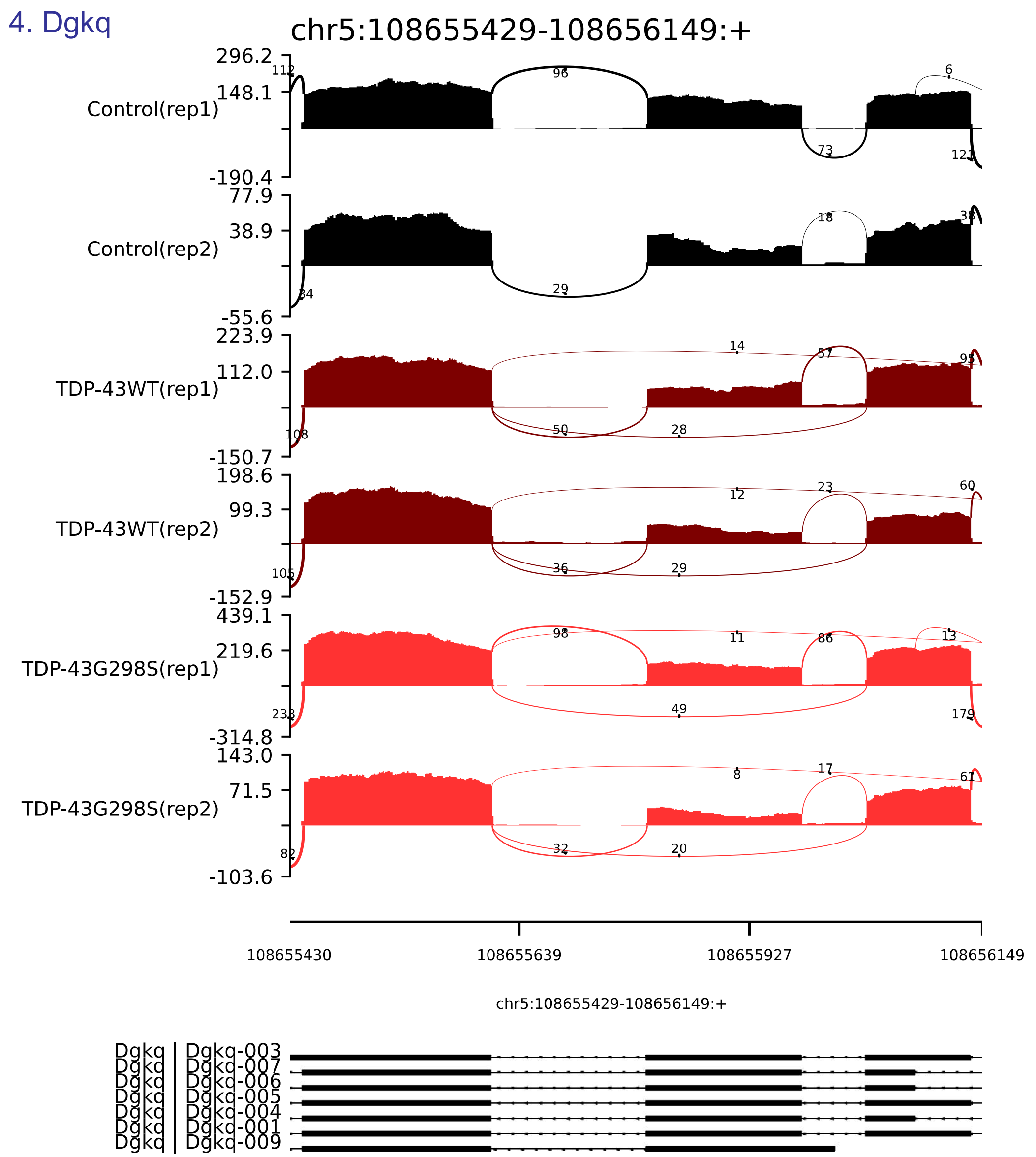
4. Dgkq
chr5:108655429-108656149:+
296.2
148.1
-190.4
Control(rep1)
77.9
38.9
-55.6
Control(rep2)
223.9
112.0
-150.7
TDP-43WT(rep1)
198.6
99.3
-152.9
TDP-43WT(rep2)
439.1
219.6
-314.8
TDP-43G298S(rep1)
143.0
71.5
-103.6
TDP-43G298S(rep2)
108655430
108655639
108655927
108656149
chr5:108655429-108656149:+
Dgkq | Dgkq-003
Dgkq | Dgkq-007
Dgkq | Dgkq-006
Dgkq | Dgkq-005
Dgkq | Dgkq-004
Dgkq | Dgkq-001
Dgkq | Dgkq-009

5. Mrpl45

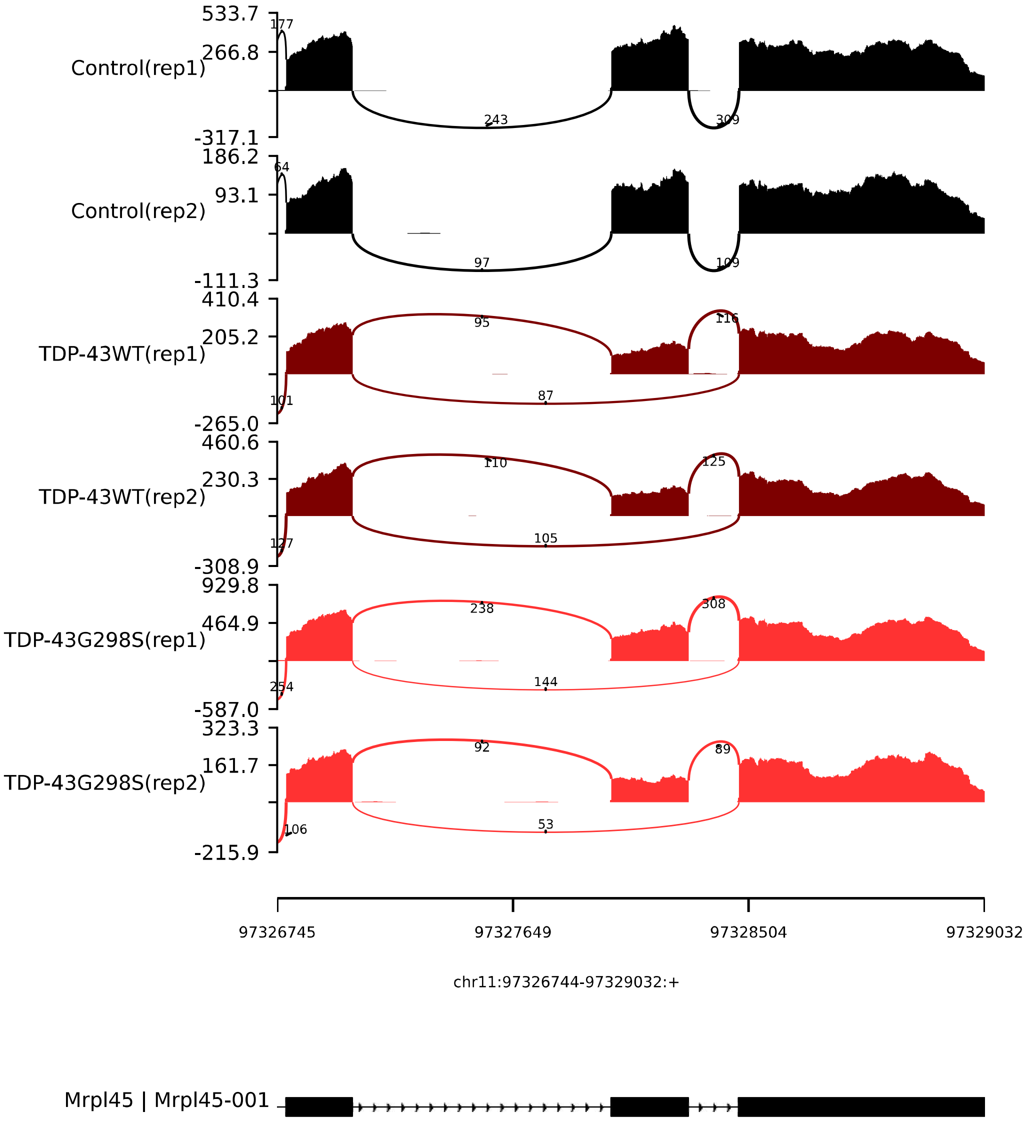

6. Psmd14

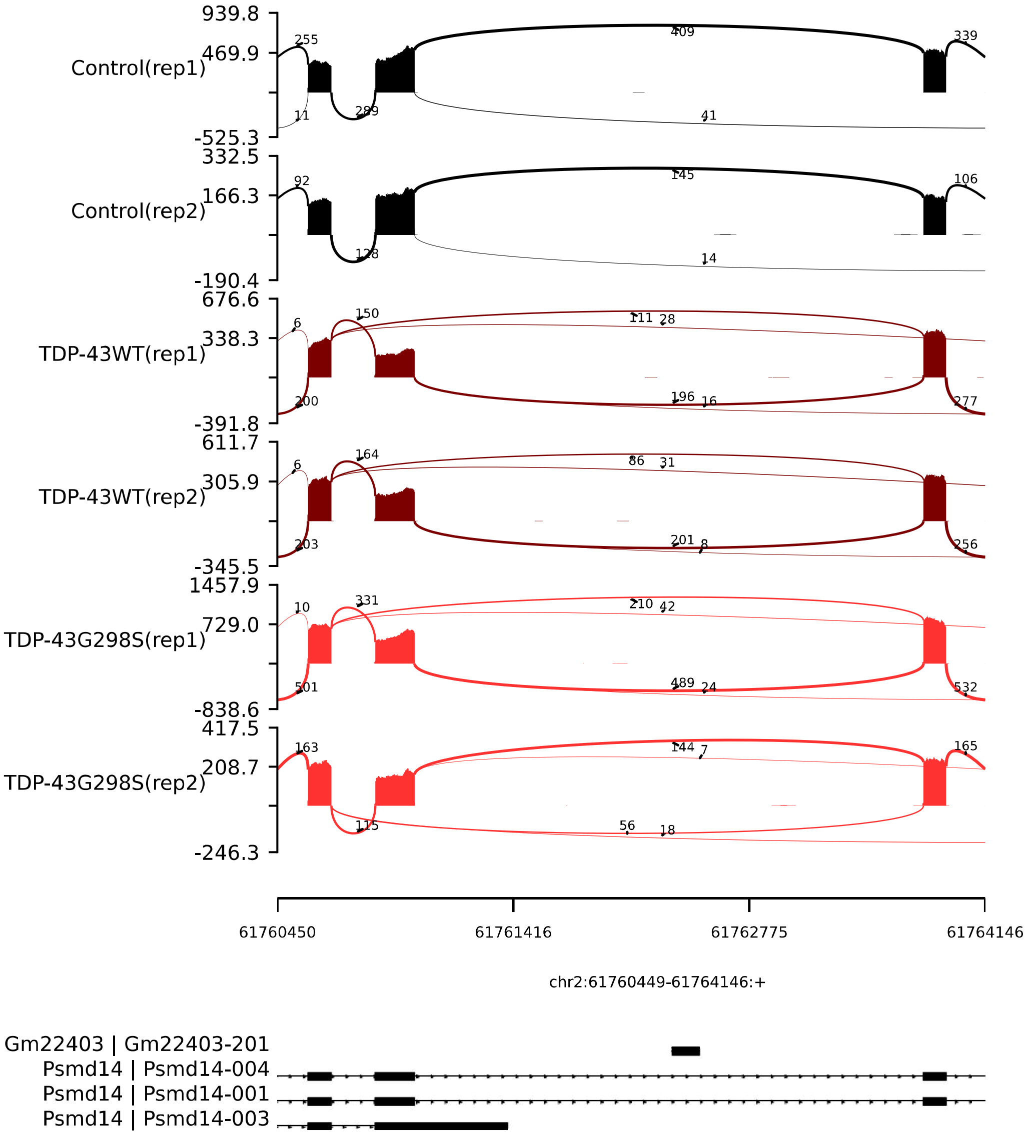

7. Hjurp

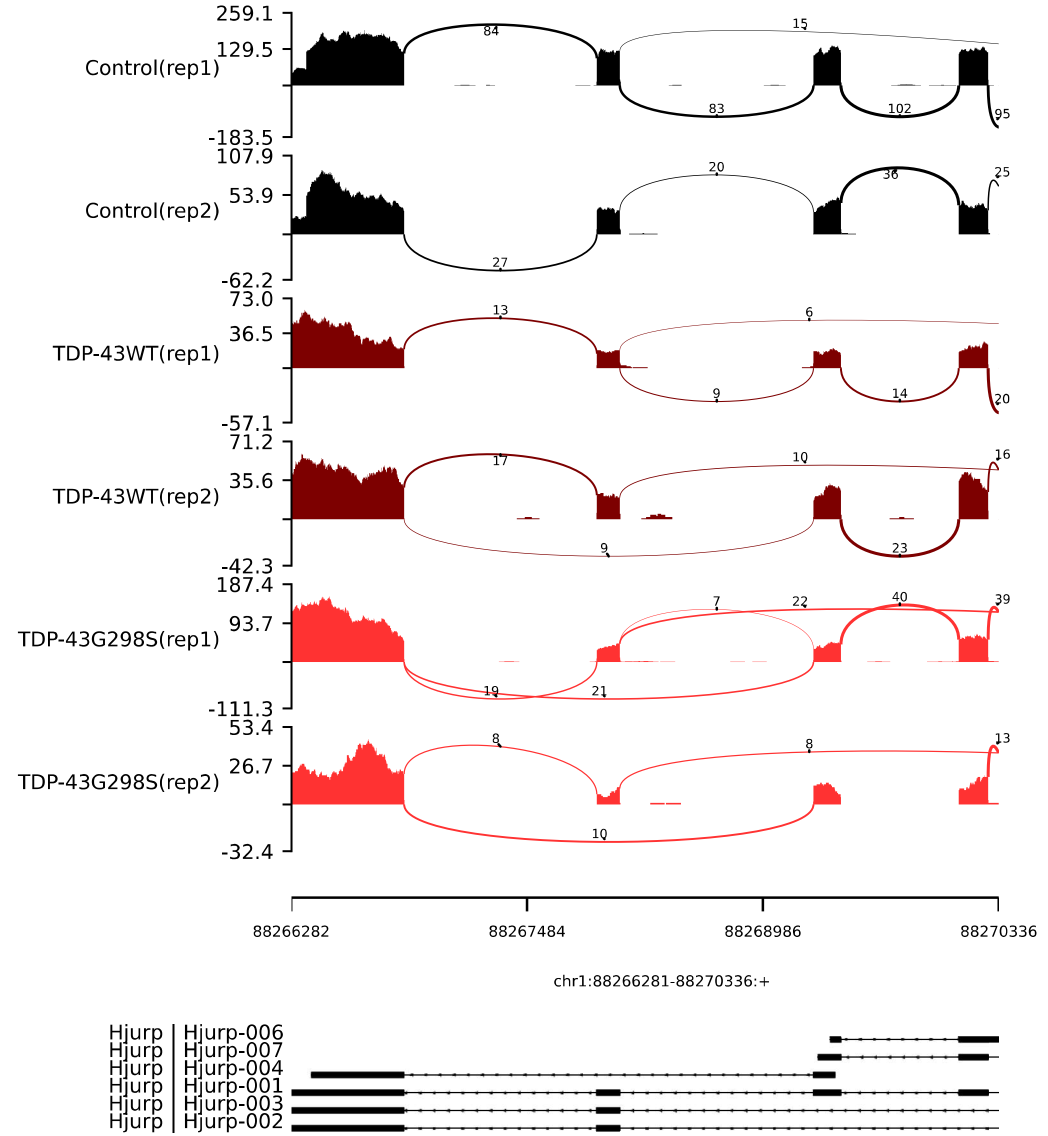

8. Cops4

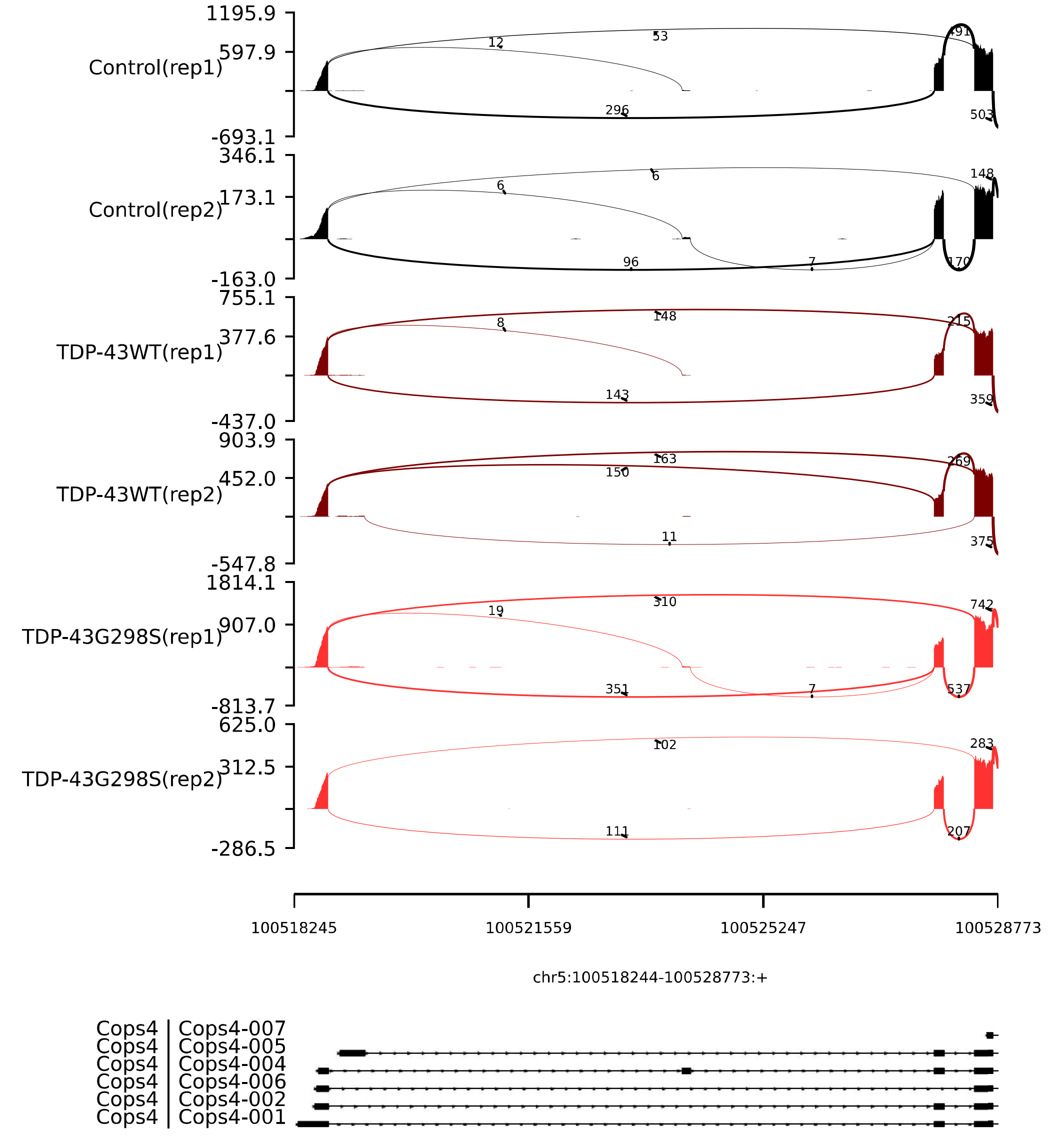

9. Tiam1

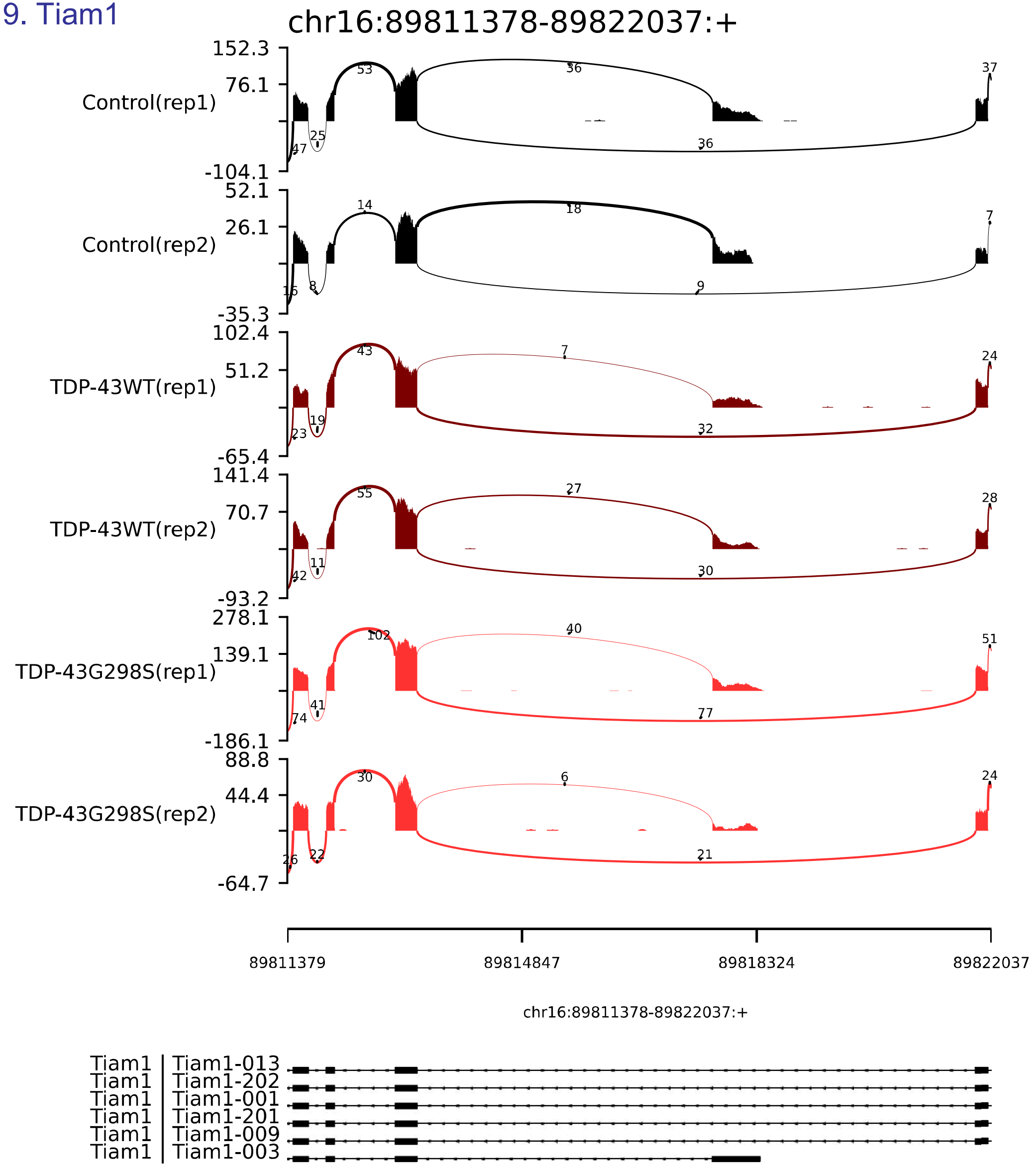

10. Creld1

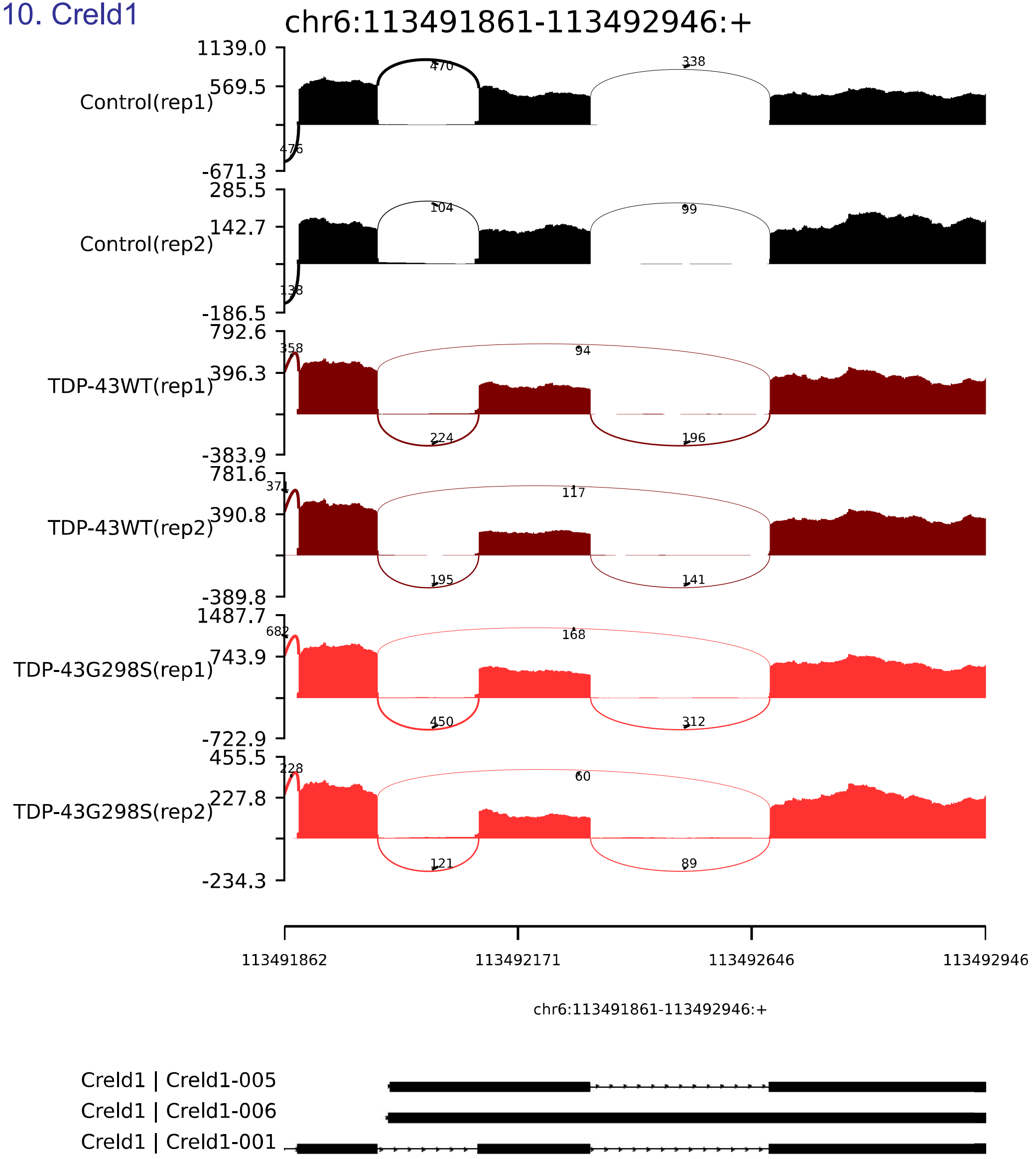

11. Inpp4a

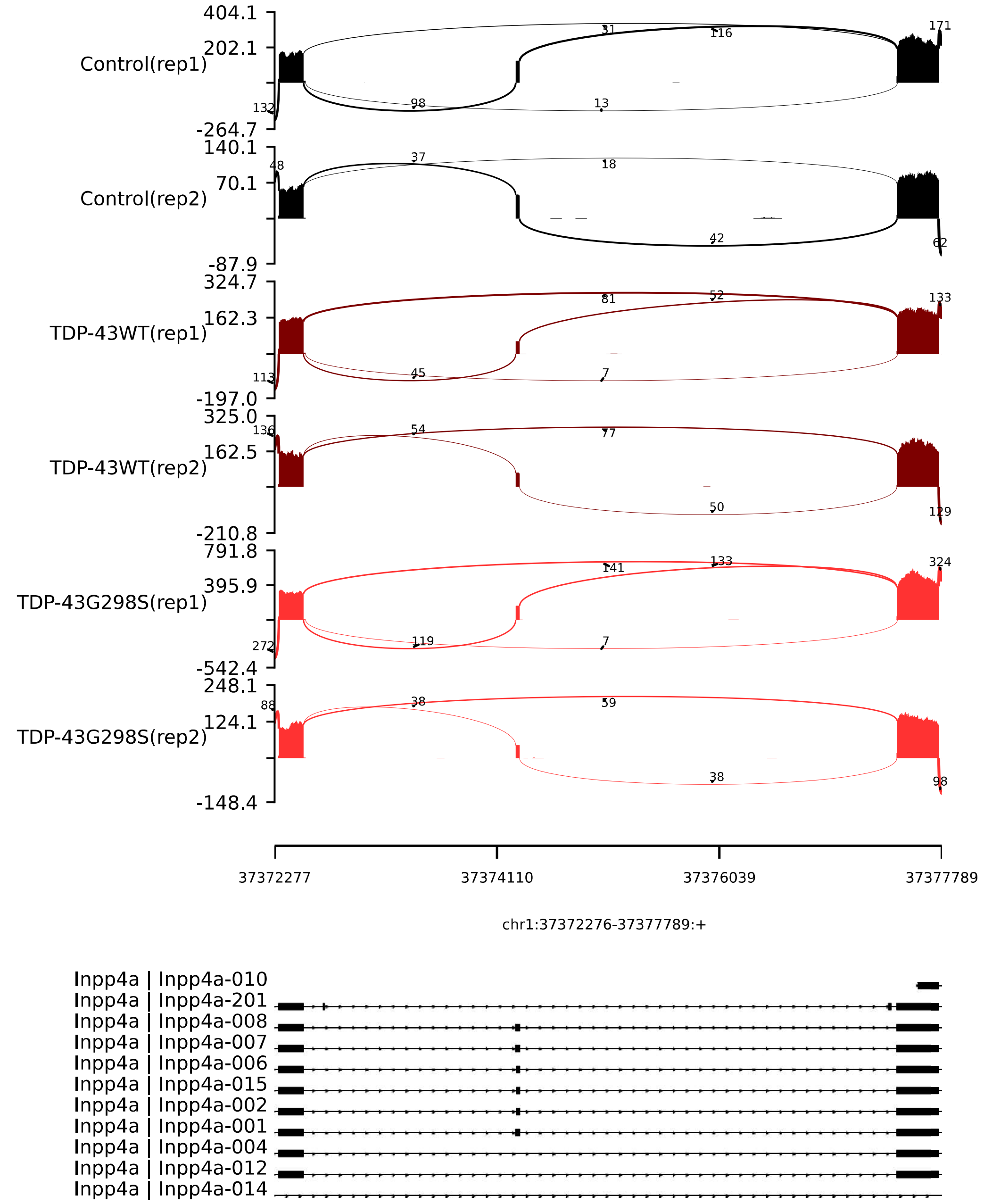

12. Shank1

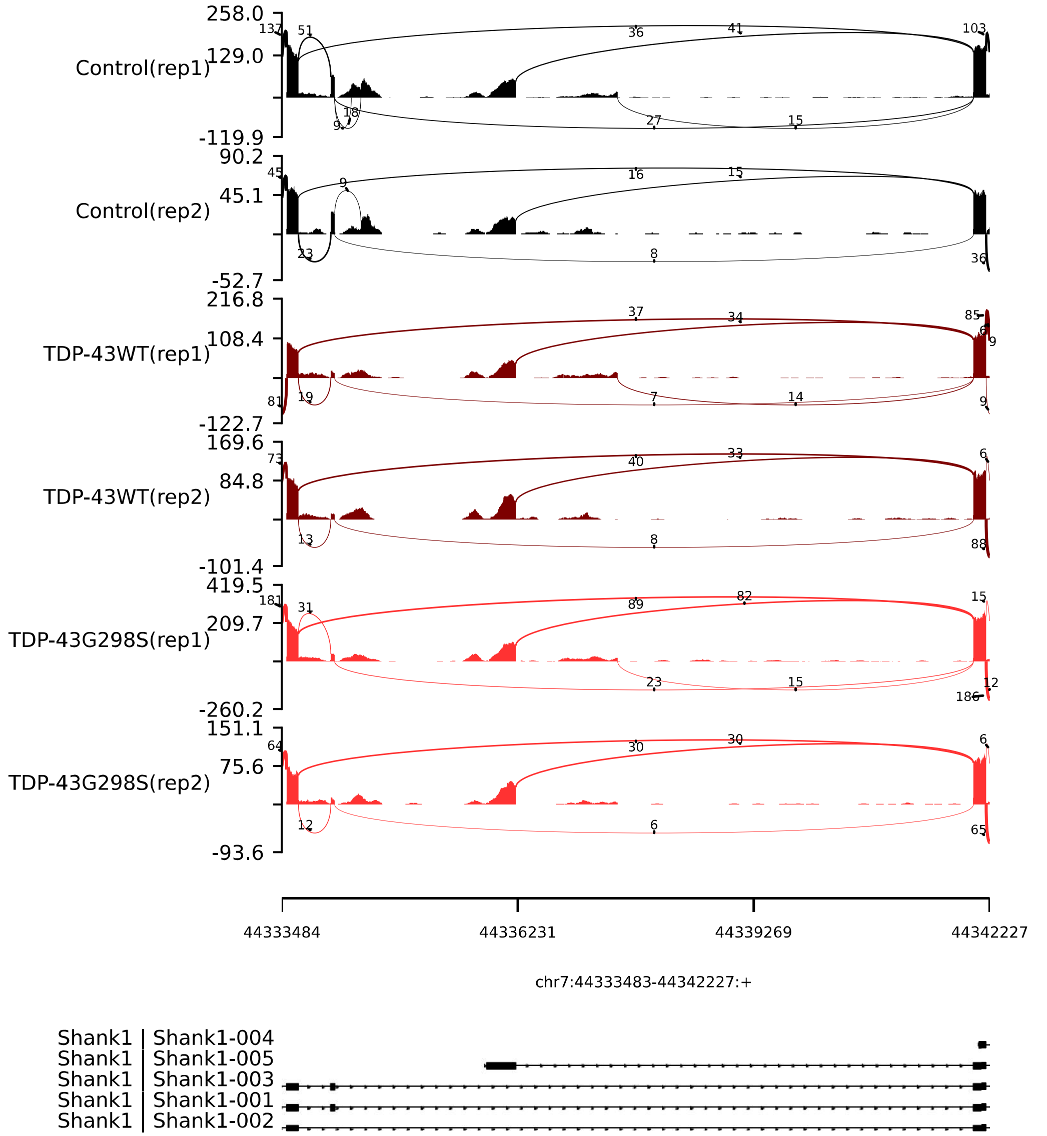

13. Pakap

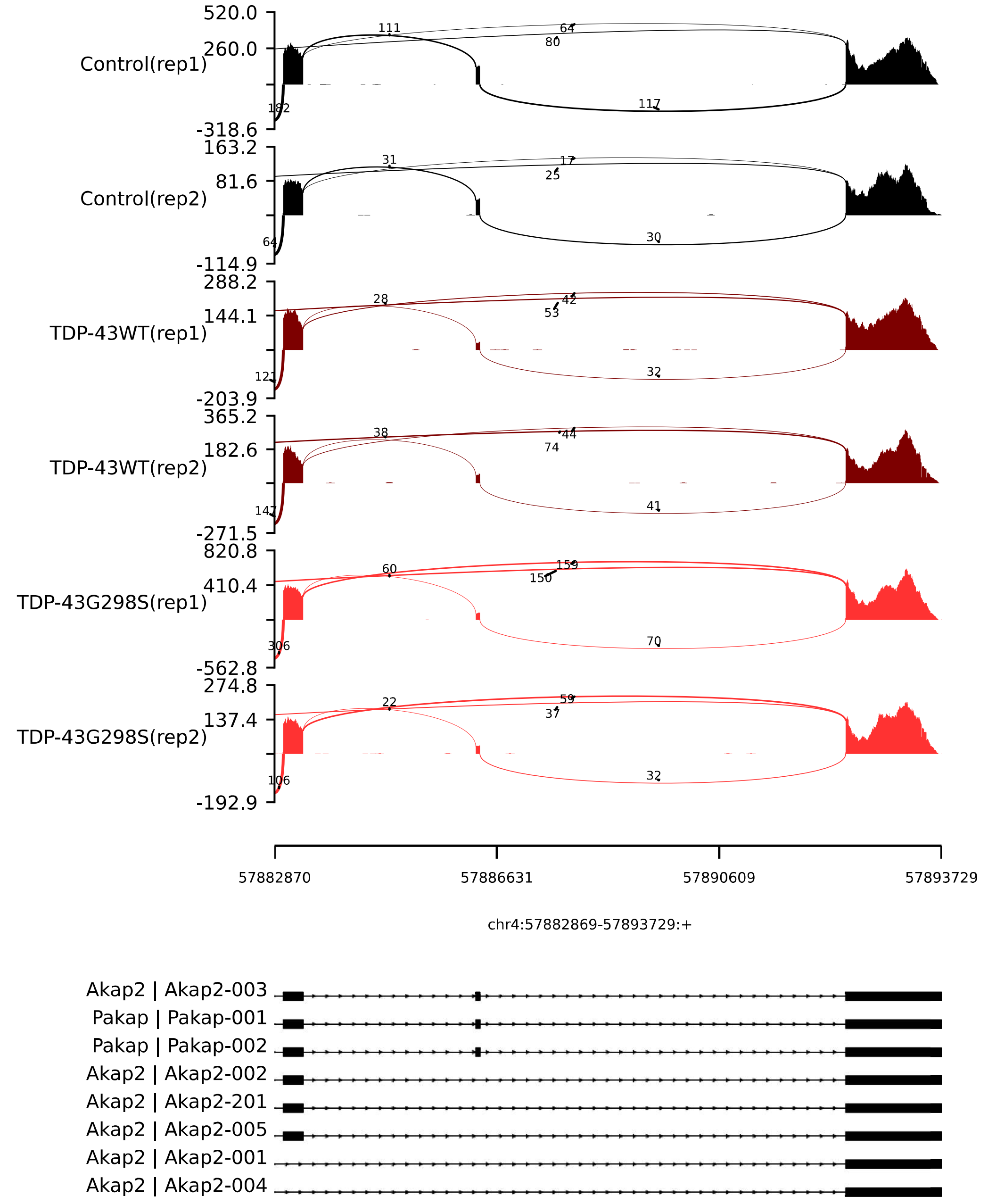

14. Arhgef11

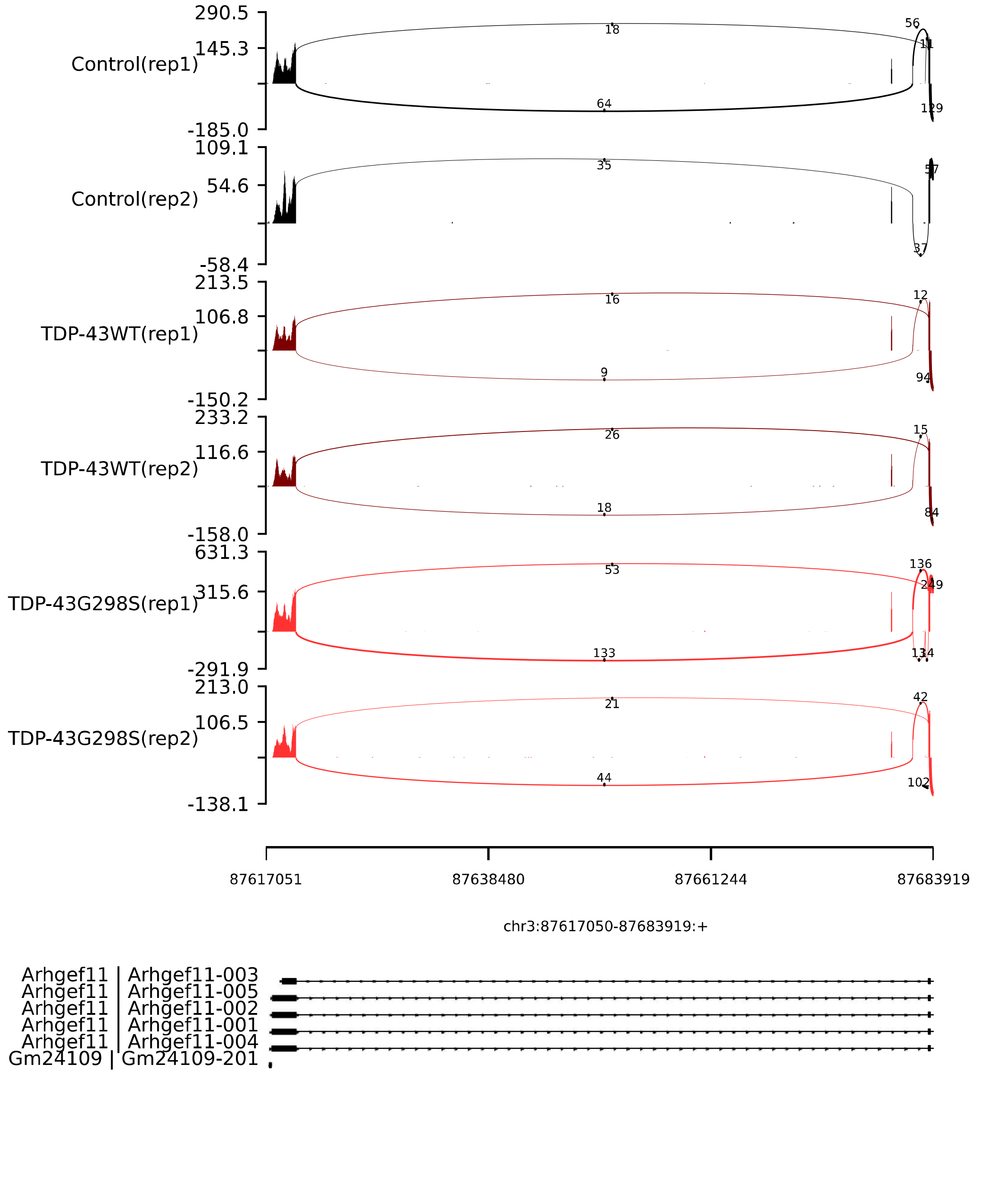

15. Osbpl6

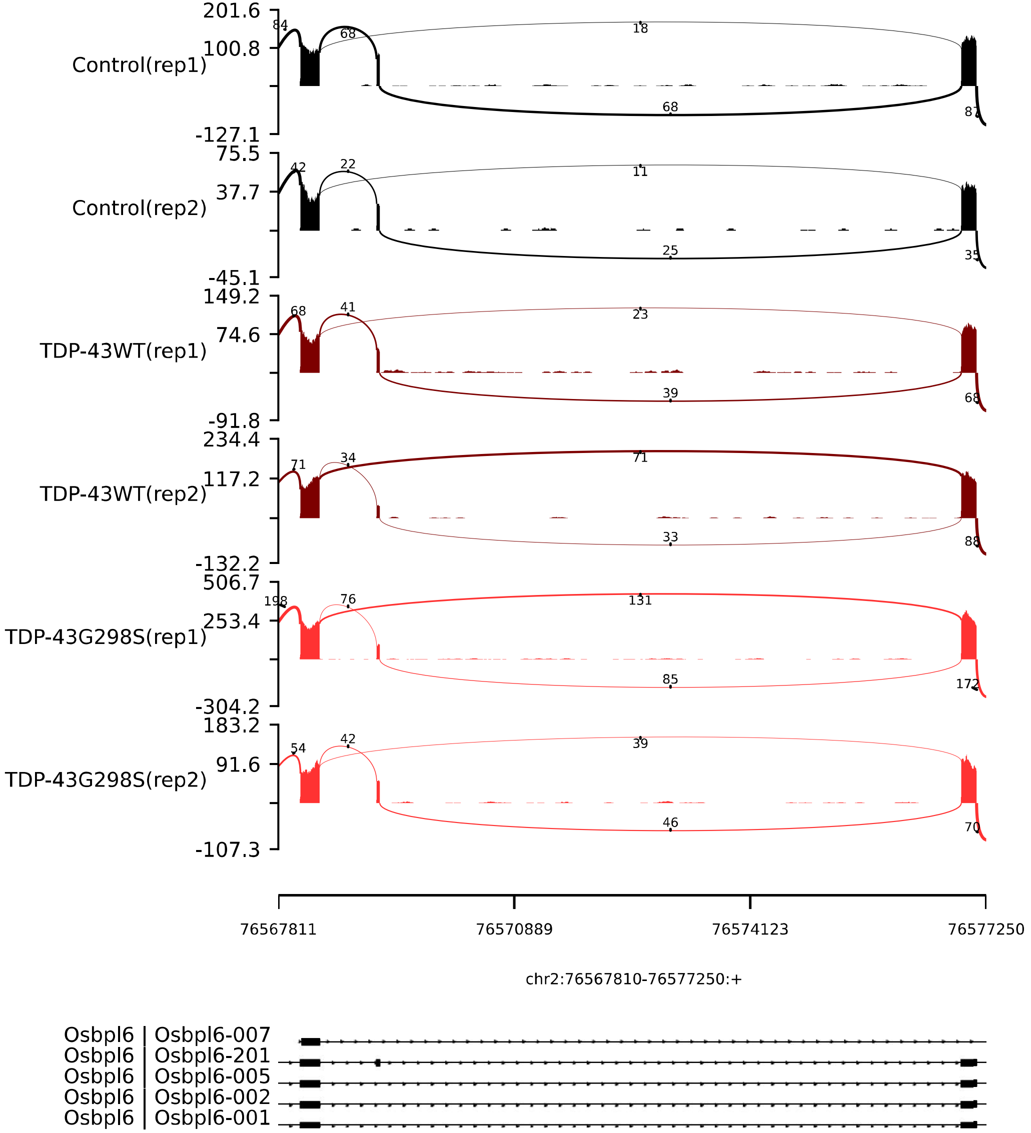

16. Pik3cb

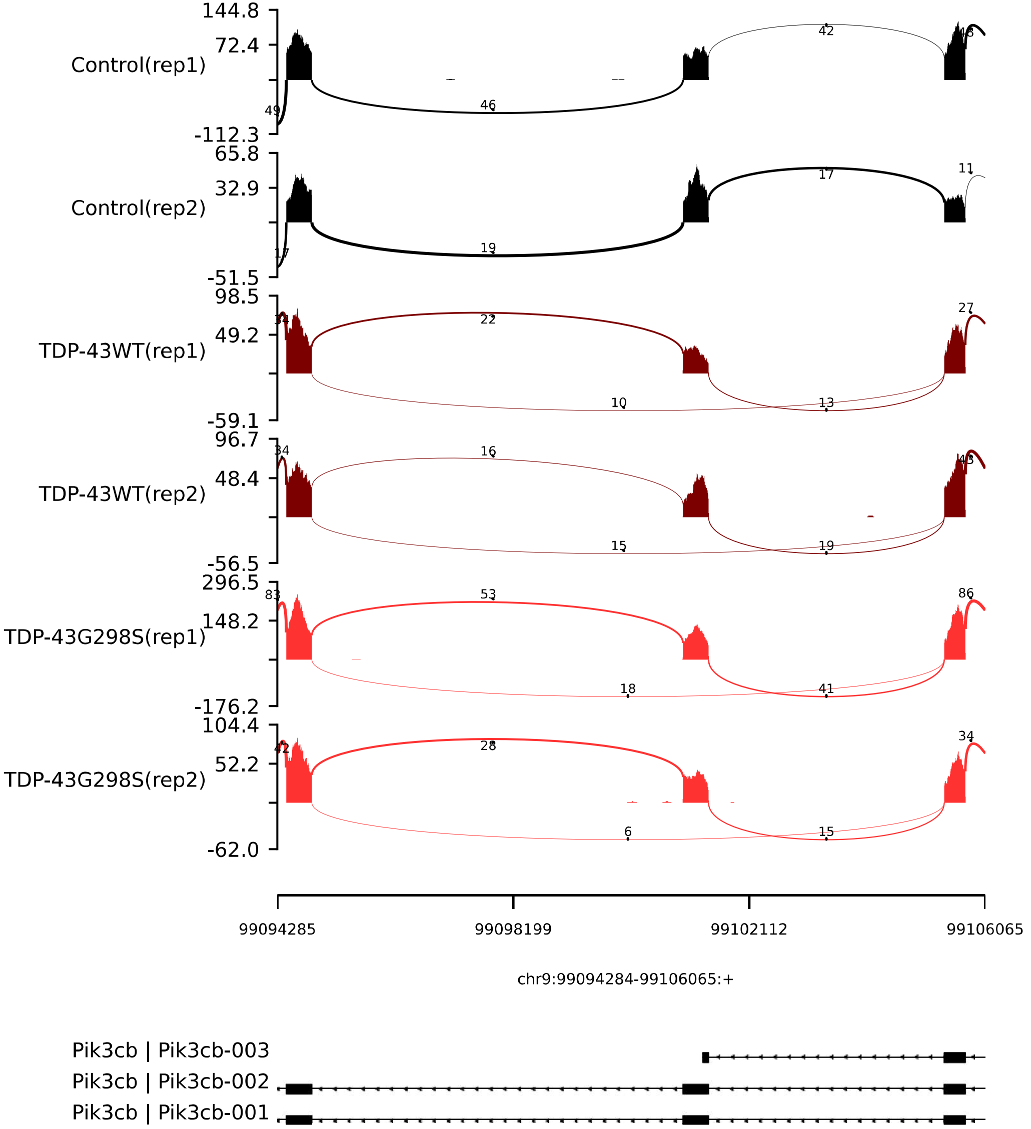

17. Pdzd4

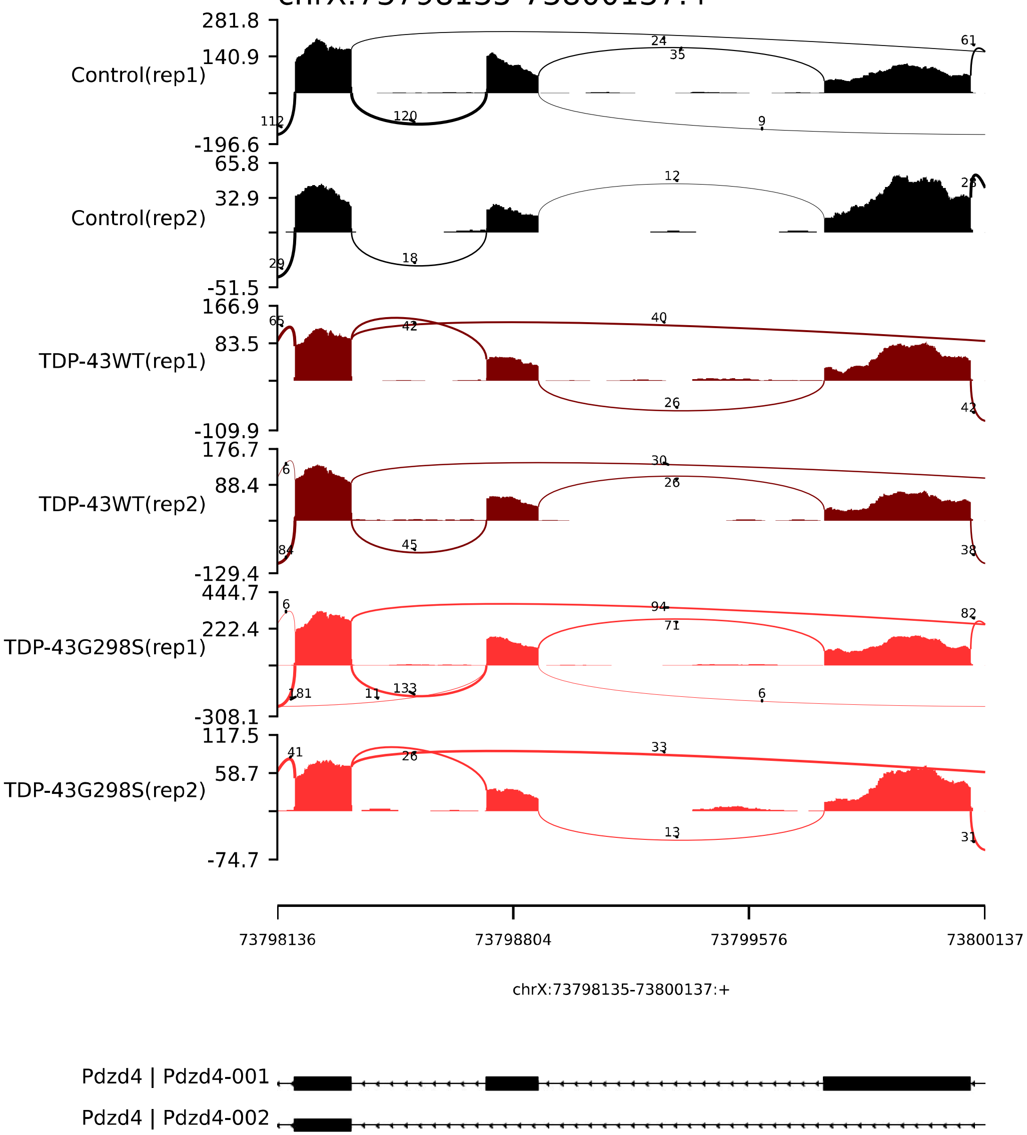

18. Dst

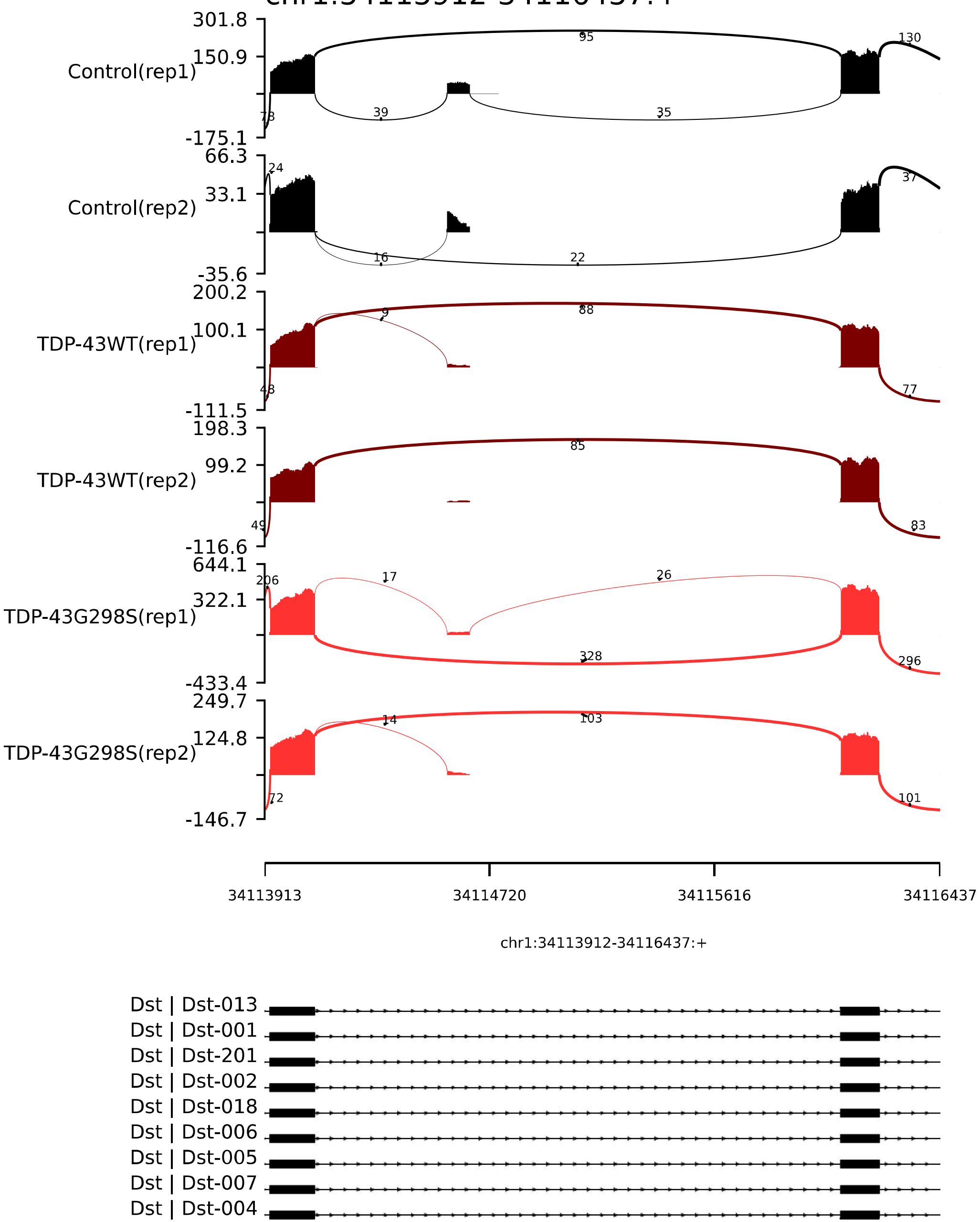

19. Herc2

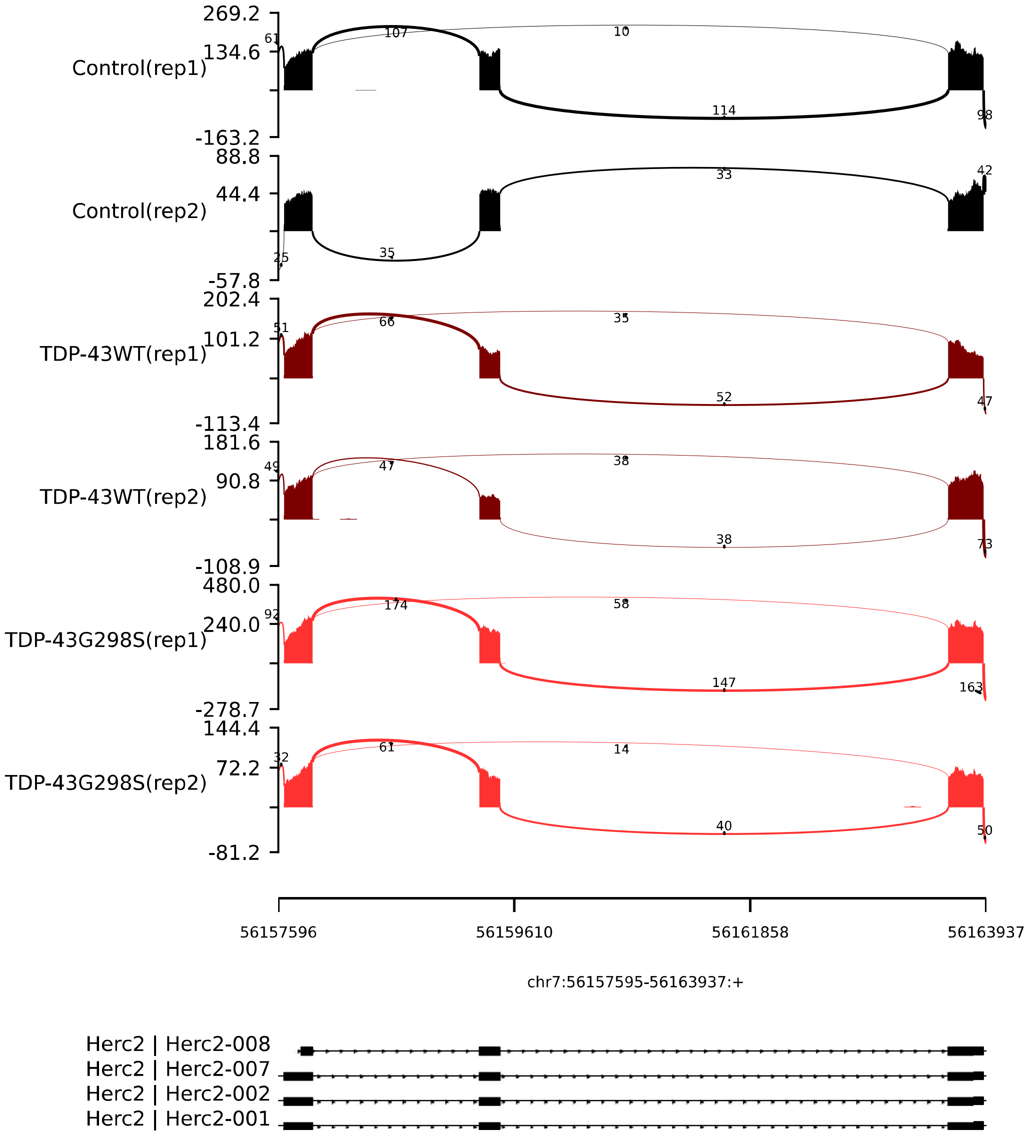

20. Golga4

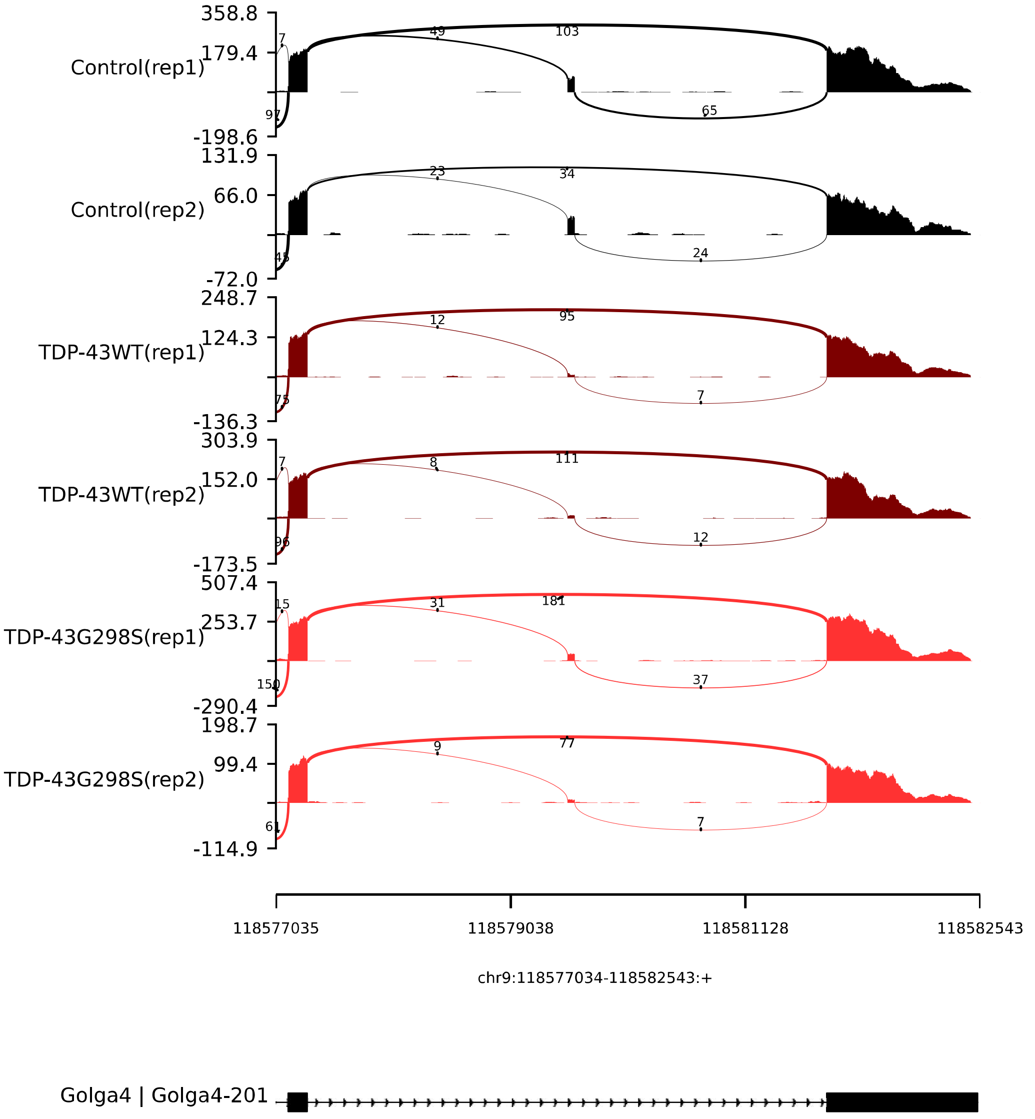

21. Stk24

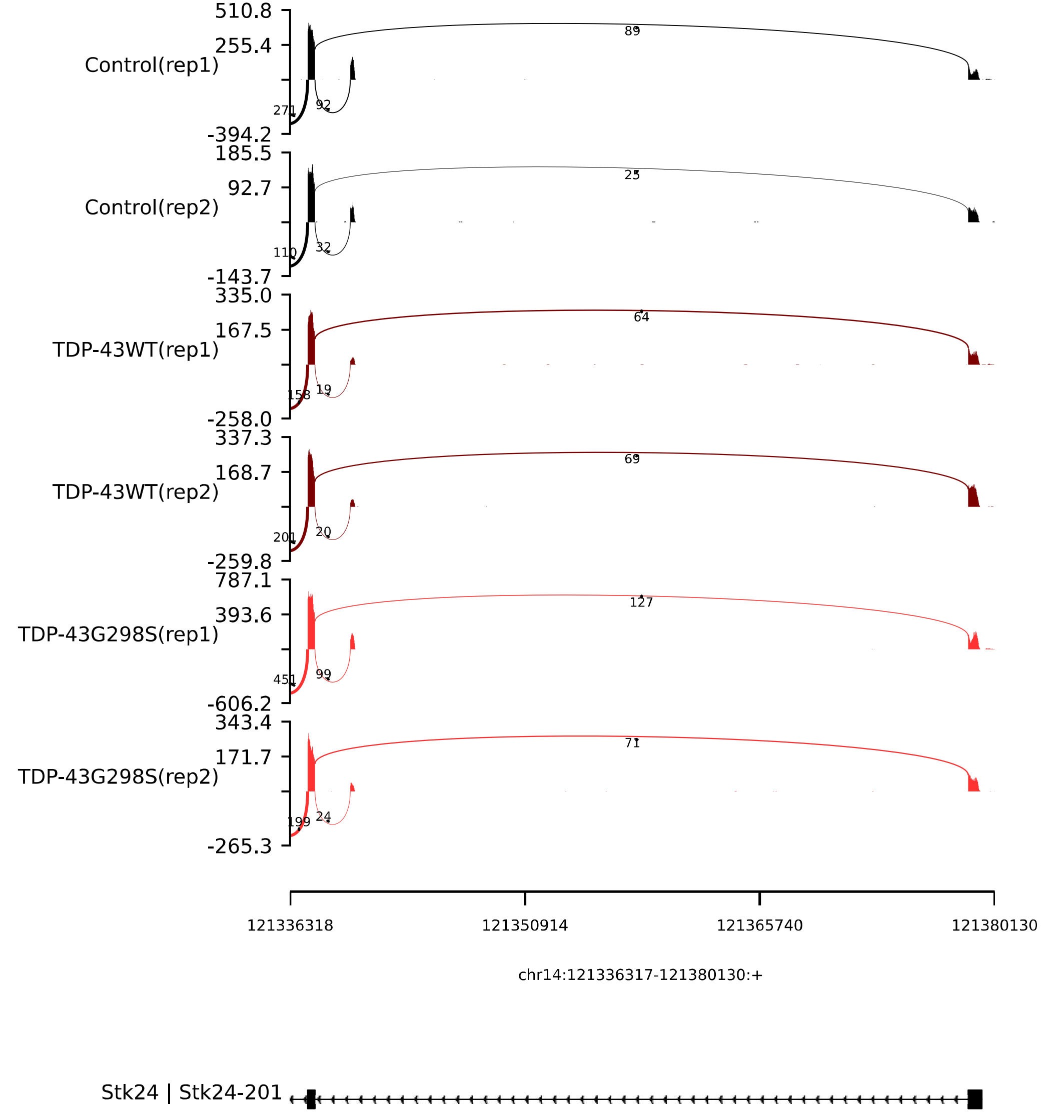

22. Ube3c

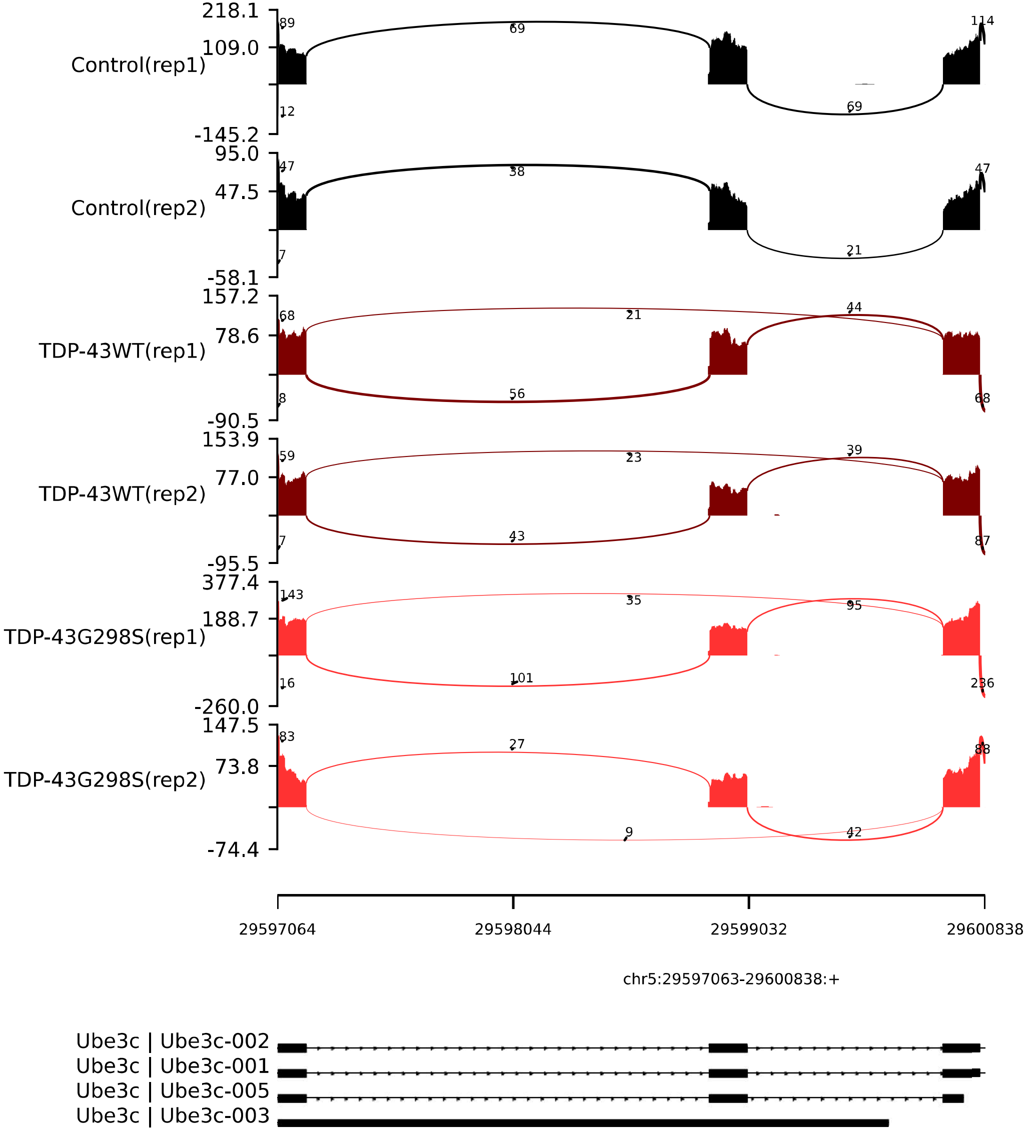

23. C530008M17Rik

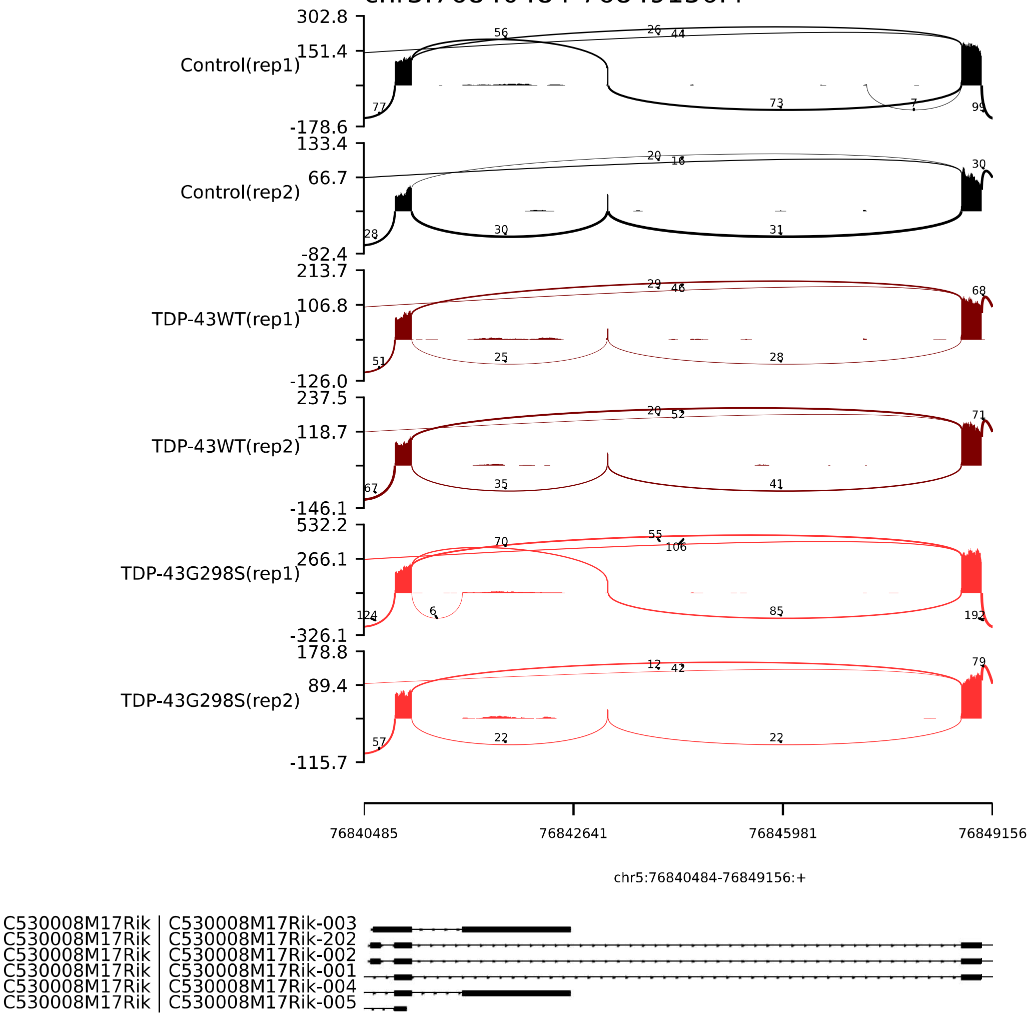

24. Tub

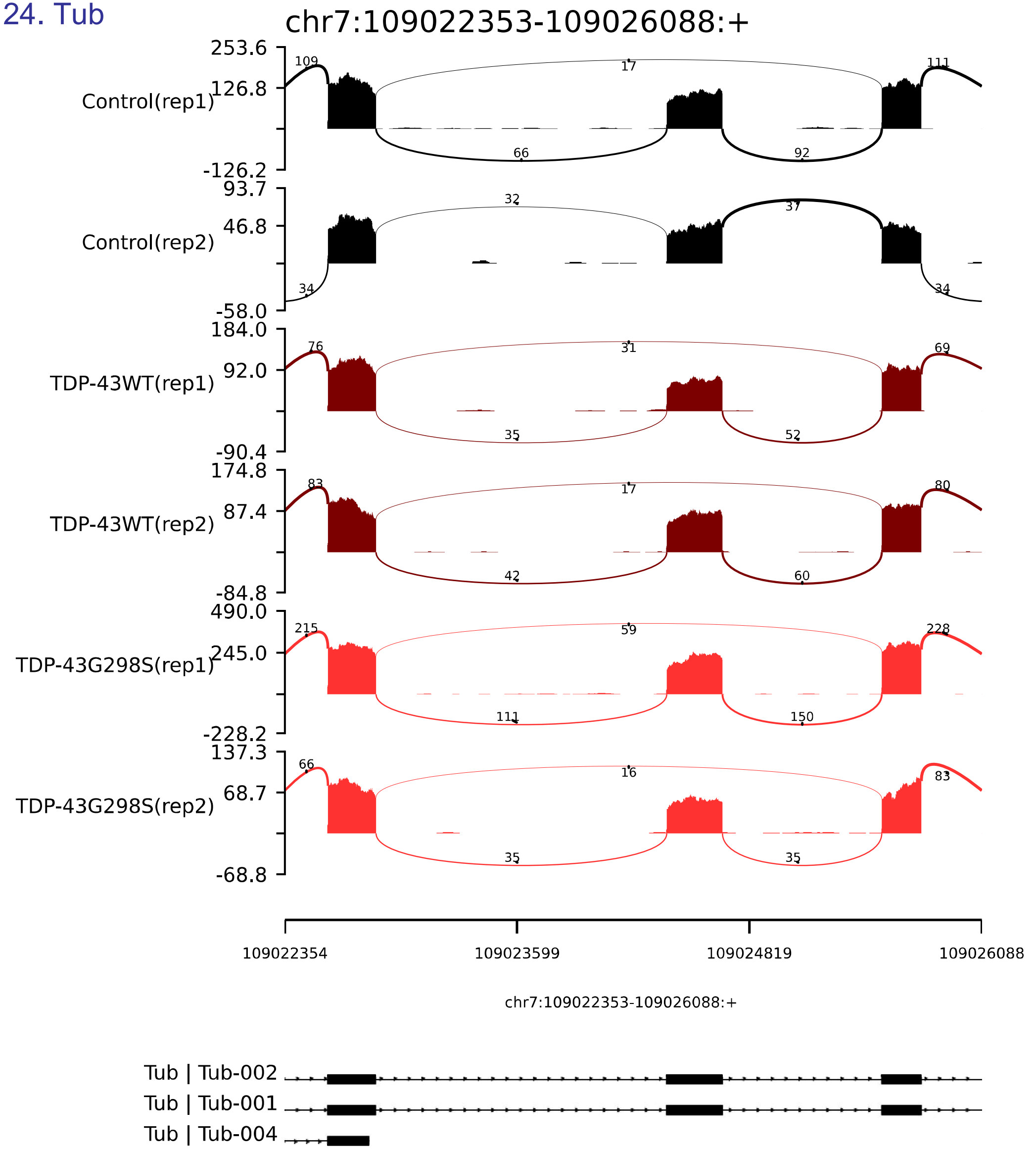

Supplement: Supplementary file 4 — Supplementary Material 4 [file 13024_2024_732_MOESM4_ESM.pdf]
