# Supplementary material for: Elevated nuclear TDP-43 induces constitutive exon skipping
Source: Mol Neurodegener. 2024 Jun 9;19:45. doi: 10.1186/s13024-024-00732-w (PMC11163724; doi:10.1186/s13024-024-00732-w)
Supplement: Supplementary file 5 — Supplementary Material 5 [file 13024_2024_732_MOESM5_ESM.pdf]

Gene	Coordinates	Splicing Type	Data
BMPR1A	chr10:86921303-86924982	skiptic exon	Carmen-Orozco, et al. 2023
CANX	chr5:179698416-179706991	skiptic exon	Carmen-Orozco, et al. 2023
COQ5	chr12:120516319-120526842	skiptic exon	Carmen-Orozco, et al. 2023
ELP2	chr18:36166598-36171437	skiptic exon	Carmen-Orozco, et al. 2023
HYOU1	chr11:119052044-119052857	skiptic exon	Carmen-Orozco, et al. 2023
MYBBP1A	chr17:4544952-4545777	skiptic exon	Carmen-Orozco, et al. 2023
NUP93	chr16:56838753-56841937	skiptic exon	Carmen-Orozco, et al. 2023
SCN9A	chr2:166272081-166280656	skiptic exon	Carmen-Orozco, et al. 2023
SESN3	chr11:95185056-95191820	skiptic exon	Carmen-Orozco, et al. 2023
SLC35A5	chr3:112562822-112571073	skiptic exon	Carmen-Orozco, et al. 2023
TESK1	chr9:35606765-35607748	skiptic exon	Carmen-Orozco, et al. 2023
VARS2	chr6:30916103-30917282	skiptic exon	Carmen-Orozco, et al. 2023
WSCD1	chr17:6080025-6095711	skiptic exon	Carmen-Orozco, et al. 2023
XPNPEP1	chr10:109883737-109888276	skiptic exon	Carmen-Orozco, et al. 2023
DDI2	chr1:15651731-15662471	skiptic exon	Carmen-Orozco, et al. 2023
PLOD1	chr1:11965458-11967116	skiptic exon	Fratta, et al. 2018
SLC6A6	chr3:14467812-14472314	skiptic exon	Fratta, et al. 2018
ACTL6B	chr7:100649782-100655263	cryptic exon	Irwin, et al. 2024
AGRN	chr1:1044046-1045563	cryptic exon	Irwin, et al. 2025
EPB41L4A	chr5:112265962-112275610	cryptic exon	Irwin, et al. 2026
HDGFL2	chr19:4491492-4494096	cryptic exon	Irwin, et al. 2027
SLC24A3	chr20:19681638-19684461	cryptic exon	Irwin, et al. 2028
STMN2	chr8:79610163-79637892	cryptic exon	Klim, et al. 2019, Melamed et al. 2019
UNC13A	chr19:17641397-17642959	cryptic exon	Rosa Ma, et al. 2022
ATG4B	chr2:241667964-241672277	cryptic exon	Ling, et al. 2015
GPSM2	chr1:108892000-108899964	cryptic exon	Ling, et al. 2015
PFKP	chr10:3081102-3102438	cryptic exon	Ling, et al. 2015

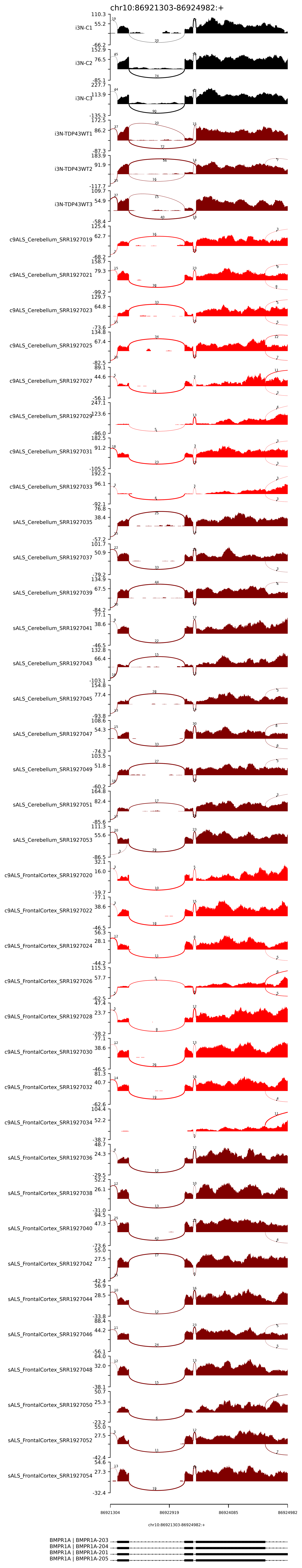

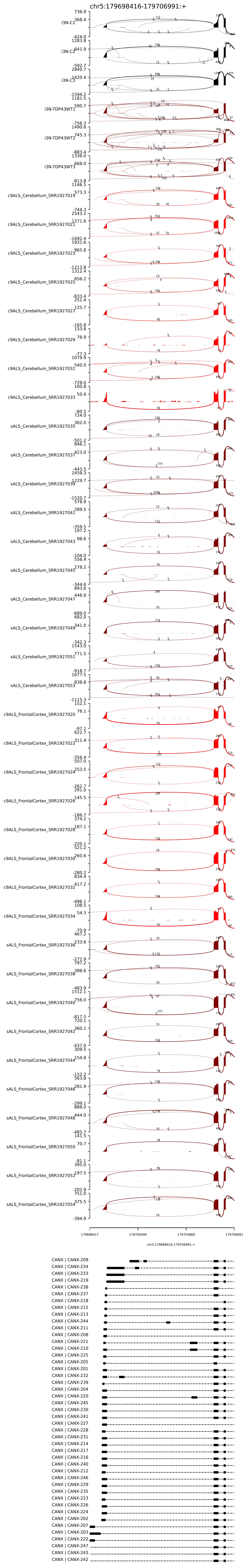
chr5:179698416-179706991:+
i3N-C1
i3N-C2
i3N-C3
i3N-TDP43WT1
i3N-TDP43WT2
i3N-TDP43WT3
c9ALS_Cerebellum_SRR1927019
c9ALS_Cerebellum_SRR1927021
c9ALS_Cerebellum_SRR1927023
c9ALS_Cerebellum_SRR1927025
c9ALS_Cerebellum_SRR1927027
c9ALS_Cerebellum_SRR1927029
c9ALS_Cerebellum_SRR1927031
c9ALS_Cerebellum_SRR1927033
sALS_Cerebellum_SRR1927035
sALS_Cerebellum_SRR1927037
sALS_Cerebellum_SRR1927039
sALS_Cerebellum_SRR1927041
sALS_Cerebellum_SRR1927043
sALS_Cerebellum_SRR1927045
sALS_Cerebellum_SRR1927047
sALS_Cerebellum_SRR1927049
sALS_Cerebellum_SRR1927051
sALS_Cerebellum_SRR1927053
c9ALS_FrontalCortex_SRR1927020
c9ALS_FrontalCortex_SRR1927022
c9ALS_FrontalCortex_SRR1927024
c9ALS_FrontalCortex_SRR1927026
c9ALS_FrontalCortex_SRR1927028
c9ALS_FrontalCortex_SRR1927030
c9ALS_FrontalCortex_SRR1927032
c9ALS_FrontalCortex_SRR1927034
sALS_FrontalCortex_SRR1927036
sALS_FrontalCortex_SRR1927038
sALS_FrontalCortex_SRR1927040
sALS_FrontalCortex_SRR1927042
sALS_FrontalCortex_SRR1927044
sALS_FrontalCortex_SRR1927046
sALS_FrontalCortex_SRR1927048
sALS_FrontalCortex_SRR1927050
sALS_FrontalCortex_SRR1927052
sALS_FrontalCortex_SRR1927054
179698417
179700494
179703860
179706991
chr5:179698416-179706991:+
CANX | CANX-209
CANX | CANX-234
CANX | CANX-233
CANX | CANX-219
CANX | CANX-238
CANX | CANX-237
CANX | CANX-218
CANX | CANX-215
CANX | CANX-213
CANX | CANX-244
CANX | CANX-211
CANX | CANX-208
CANX | CANX-221
CANX | CANX-210
CANX | CANX-225
CANX | CANX-205
CANX | CANX-201
CANX | CANX-232
CANX | CANX-239
CANX | CANX-204
CANX | CANX-220
CANX | CANX-245
CANX | CANX-230
CANX | CANX-241
CANX | CANX-227
CANX | CANX-228
CANX | CANX-231
CANX | CANX-214
CANX | CANX-217
CANX | CANX-216
CANX | CANX-240
CANX | CANX-212
CANX | CANX-246
CANX | CANX-229
CANX | CANX-235
CANX | CANX-223
CANX | CANX-226
CANX | CANX-224
CANX | CANX-202
CANX | CANX-207
CANX | CANX-203
CANX | CANX-222
CANX | CANX-247
CANX | CANX-243
CANX | CANX-242

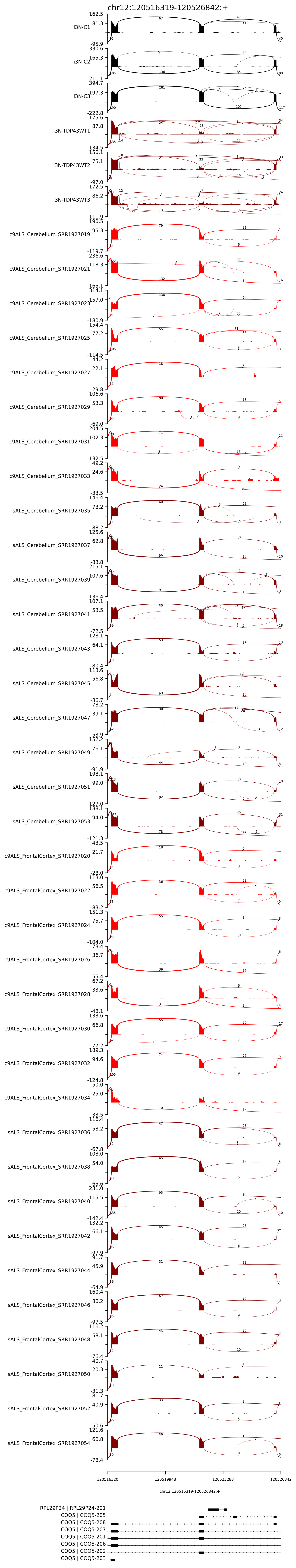
chr12:120516319-120526842:+
i3N-C1
i3N-C2
i3N-C3
i3N-TDP43WT1
i3N-TDP43WT2
i3N-TDP43WT3
c9ALS_Cerebellum_SRR1927019
c9ALS_Cerebellum_SRR1927021
c9ALS_Cerebellum_SRR1927023
c9ALS_Cerebellum_SRR1927025
c9ALS_Cerebellum_SRR1927027
c9ALS_Cerebellum_SRR1927029
c9ALS_Cerebellum_SRR1927031
c9ALS_Cerebellum_SRR1927033
sALS_Cerebellum_SRR1927035
sALS_Cerebellum_SRR1927037
sALS_Cerebellum_SRR1927039
sALS_Cerebellum_SRR1927041
sALS_Cerebellum_SRR1927043
sALS_Cerebellum_SRR1927045
sALS_Cerebellum_SRR1927047
sALS_Cerebellum_SRR1927049
sALS_Cerebellum_SRR1927051
sALS_Cerebellum_SRR1927053
c9ALS_FrontalCortex_SRR1927020
c9ALS_FrontalCortex_SRR1927022
c9ALS_FrontalCortex_SRR1927024
c9ALS_FrontalCortex_SRR1927026
c9ALS_FrontalCortex_SRR1927028
c9ALS_FrontalCortex_SRR1927030
c9ALS_FrontalCortex_SRR1927032
c9ALS_FrontalCortex_SRR1927034
sALS_FrontalCortex_SRR1927036
sALS_FrontalCortex_SRR1927038
sALS_FrontalCortex_SRR1927040
sALS_FrontalCortex_SRR1927042
sALS_FrontalCortex_SRR1927044
sALS_FrontalCortex_SRR1927046
sALS_FrontalCortex_SRR1927048
sALS_FrontalCortex_SRR1927050
sALS_FrontalCortex_SRR1927052
sALS_FrontalCortex_SRR1927054
120516320
120519948
120523288
120526842
chr12:120516319-120526842:+
RPL29P24 | RPL29P24-201
COQ5 | COQ5-205
COQ5 | COQ5-208
COQ5 | COQ5-207
COQ5 | COQ5-201
COQ5 | COQ5-206
COQ5 | COQ5-202
COQ5 | COQ5-203

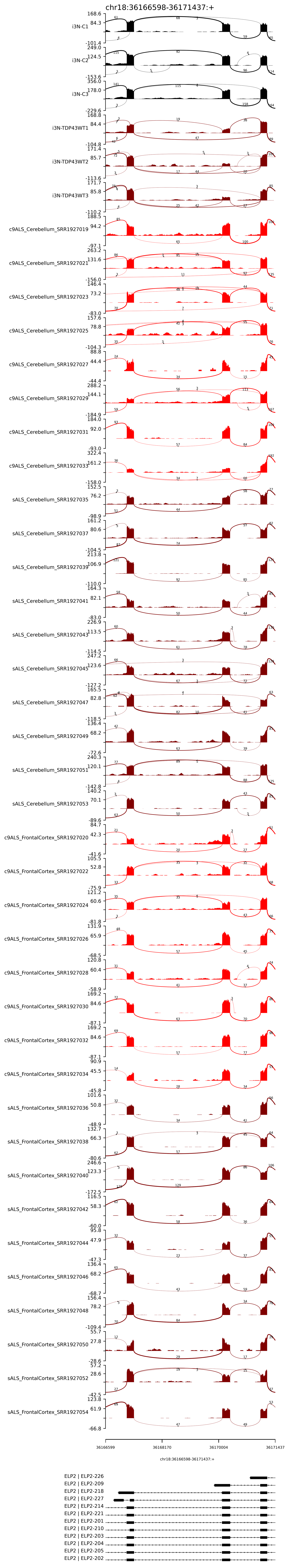
chr18:36166598-36171437:+
i3N-C1
i3N-C2
i3N-C3
i3N-TDP43WT1
i3N-TDP43WT2
i3N-TDP43WT3
c9ALS_Cerebellum_SRR1927019
c9ALS_Cerebellum_SRR1927021
c9ALS_Cerebellum_SRR1927023
c9ALS_Cerebellum_SRR1927025
c9ALS_Cerebellum_SRR1927027
c9ALS_Cerebellum_SRR1927029
c9ALS_Cerebellum_SRR1927031
c9ALS_Cerebellum_SRR1927033
sALS_Cerebellum_SRR1927035
sALS_Cerebellum_SRR1927037
sALS_Cerebellum_SRR1927039
sALS_Cerebellum_SRR1927041
sALS_Cerebellum_SRR1927043
sALS_Cerebellum_SRR1927045
sALS_Cerebellum_SRR1927047
sALS_Cerebellum_SRR1927049
sALS_Cerebellum_SRR1927051
sALS_Cerebellum_SRR1927053
c9ALS_FrontalCortex_SRR1927020
c9ALS_FrontalCortex_SRR1927022
c9ALS_FrontalCortex_SRR1927024
c9ALS_FrontalCortex_SRR1927026
c9ALS_FrontalCortex_SRR1927028
c9ALS_FrontalCortex_SRR1927030
c9ALS_FrontalCortex_SRR1927032
c9ALS_FrontalCortex_SRR1927034
sALS_FrontalCortex_SRR1927036
sALS_FrontalCortex_SRR1927038
sALS_FrontalCortex_SRR1927040
sALS_FrontalCortex_SRR1927042
sALS_FrontalCortex_SRR1927044
sALS_FrontalCortex_SRR1927046
sALS_FrontalCortex_SRR1927048
sALS_FrontalCortex_SRR1927050
sALS_FrontalCortex_SRR1927052
sALS_FrontalCortex_SRR1927054
36166599
36168170
36170004
36171437
chr18:36166598-36171437:+
ELP2 | ELP2-226
ELP2 | ELP2-209
ELP2 | ELP2-218
ELP2 | ELP2-227
ELP2 | ELP2-214
ELP2 | ELP2-221
ELP2 | ELP2-201
ELP2 | ELP2-210
ELP2 | ELP2-203
ELP2 | ELP2-204
ELP2 | ELP2-205
ELP2 | ELP2-202

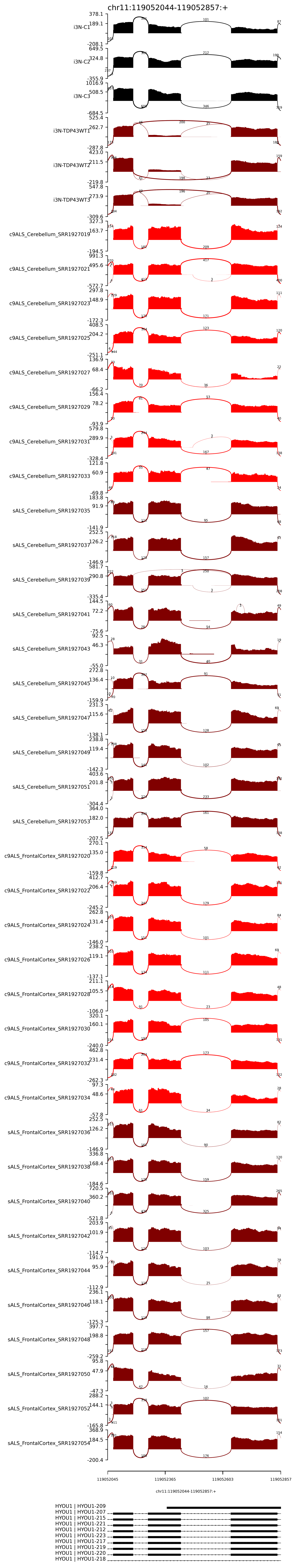

chr11:119052044-119052857:+
i3N-C1
i3N-C2
i3N-C3
i3N-TDP43WT1
i3N-TDP43WT2
i3N-TDP43WT3
c9ALS_Cerebellum_SRR1927019
c9ALS_Cerebellum_SRR1927021
c9ALS_Cerebellum_SRR1927023
c9ALS_Cerebellum_SRR1927025
c9ALS_Cerebellum_SRR1927027
c9ALS_Cerebellum_SRR1927029
c9ALS_Cerebellum_SRR1927031
c9ALS_Cerebellum_SRR1927033
sALS_Cerebellum_SRR1927035
sALS_Cerebellum_SRR1927037
sALS_Cerebellum_SRR1927039
sALS_Cerebellum_SRR1927041
sALS_Cerebellum_SRR1927043
sALS_Cerebellum_SRR1927045
sALS_Cerebellum_SRR1927047
sALS_Cerebellum_SRR1927049
sALS_Cerebellum_SRR1927051
sALS_Cerebellum_SRR1927053
c9ALS_FrontalCortex_SRR1927020
c9ALS_FrontalCortex_SRR1927022
c9ALS_FrontalCortex_SRR1927024
c9ALS_FrontalCortex_SRR1927026
c9ALS_FrontalCortex_SRR1927028
c9ALS_FrontalCortex_SRR1927030
c9ALS_FrontalCortex_SRR1927032
c9ALS_FrontalCortex_SRR1927034
sALS_FrontalCortex_SRR1927036
sALS_FrontalCortex_SRR1927038
sALS_FrontalCortex_SRR1927040
sALS_FrontalCortex_SRR1927042
sALS_FrontalCortex_SRR1927044
sALS_FrontalCortex_SRR1927046
sALS_FrontalCortex_SRR1927048
sALS_FrontalCortex_SRR1927050
sALS_FrontalCortex_SRR1927052
sALS_FrontalCortex_SRR1927054
119052045
119052365
119052603
119052857
chr11:119052044-119052857:+
HYOU1 | HYOU1-209
HYOU1 | HYOU1-207
HYOU1 | HYOU1-215
HYOU1 | HYOU1-221
HYOU1 | HYOU1-212
HYOU1 | HYOU1-223
HYOU1 | HYOU1-217
HYOU1 | HYOU1-219
HYOU1 | HYOU1-220
HYOU1 | HYOU1-218

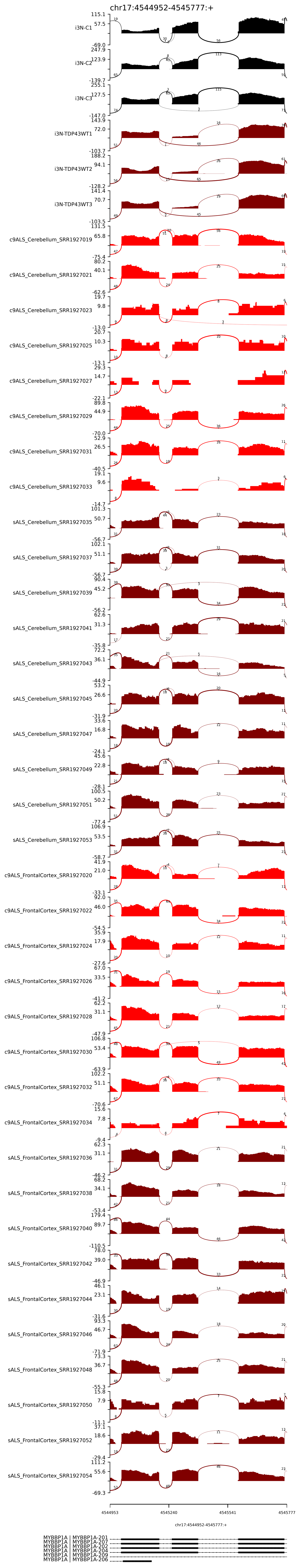
chr17:4544952-4545777:+
i3N-C1
i3N-C2
i3N-C3
i3N-TDP43WT1
i3N-TDP43WT2
i3N-TDP43WT3
c9ALS_Cerebellum_SRR1927019
c9ALS_Cerebellum_SRR1927021
c9ALS_Cerebellum_SRR1927023
c9ALS_Cerebellum_SRR1927025
c9ALS_Cerebellum_SRR1927027
c9ALS_Cerebellum_SRR1927029
c9ALS_Cerebellum_SRR1927031
c9ALS_Cerebellum_SRR1927033
sALS_Cerebellum_SRR1927035
sALS_Cerebellum_SRR1927037
sALS_Cerebellum_SRR1927039
sALS_Cerebellum_SRR1927041
sALS_Cerebellum_SRR1927043
sALS_Cerebellum_SRR1927045
sALS_Cerebellum_SRR1927047
sALS_Cerebellum_SRR1927049
sALS_Cerebellum_SRR1927051
sALS_Cerebellum_SRR1927053
c9ALS_FrontalCortex_SRR1927020
c9ALS_FrontalCortex_SRR1927022
c9ALS_FrontalCortex_SRR1927024
c9ALS_FrontalCortex_SRR1927026
c9ALS_FrontalCortex_SRR1927028
c9ALS_FrontalCortex_SRR1927030
c9ALS_FrontalCortex_SRR1927032
c9ALS_FrontalCortex_SRR1927034
sALS_FrontalCortex_SRR1927036
sALS_FrontalCortex_SRR1927038
sALS_FrontalCortex_SRR1927040
sALS_FrontalCortex_SRR1927042
sALS_FrontalCortex_SRR1927044
sALS_FrontalCortex_SRR1927046
sALS_FrontalCortex_SRR1927048
sALS_FrontalCortex_SRR1927050
sALS_FrontalCortex_SRR1927052
sALS_FrontalCortex_SRR1927054
4544953
4545240
4545541
4545777
chr17:4544952-4545777:+
MYBBP1A | MYBBP1A-201
MYBBP1A | MYBBP1A-207
MYBBP1A | MYBBP1A-202
MYBBP1A | MYBBP1A-204
MYBBP1A | MYBBP1A-209
MYBBP1A | MYBBP1A-206

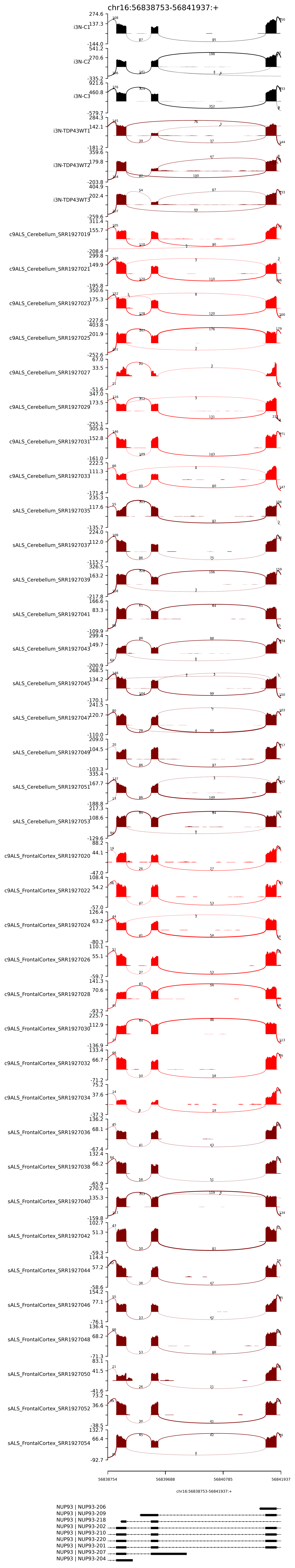

chr16:56838753-56841937:+
i3N-C1
i3N-C2
i3N-C3
i3N-TDP43WT1
i3N-TDP43WT2
i3N-TDP43WT3
c9ALS_Cerebellum_SRR1927019
c9ALS_Cerebellum_SRR1927021
c9ALS_Cerebellum_SRR1927023
c9ALS_Cerebellum_SRR1927025
c9ALS_Cerebellum_SRR1927027
c9ALS_Cerebellum_SRR1927029
c9ALS_Cerebellum_SRR1927031
c9ALS_Cerebellum_SRR1927033
sALS_Cerebellum_SRR1927035
sALS_Cerebellum_SRR1927037
sALS_Cerebellum_SRR1927039
sALS_Cerebellum_SRR1927041
sALS_Cerebellum_SRR1927043
sALS_Cerebellum_SRR1927045
sALS_Cerebellum_SRR1927047
sALS_Cerebellum_SRR1927049
sALS_Cerebellum_SRR1927051
sALS_Cerebellum_SRR1927053
c9ALS_FrontalCortex_SRR1927020
c9ALS_FrontalCortex_SRR1927022
c9ALS_FrontalCortex_SRR1927024
c9ALS_FrontalCortex_SRR1927026
c9ALS_FrontalCortex_SRR1927028
c9ALS_FrontalCortex_SRR1927030
c9ALS_FrontalCortex_SRR1927032
c9ALS_FrontalCortex_SRR1927034
sALS_FrontalCortex_SRR1927036
sALS_FrontalCortex_SRR1927038
sALS_FrontalCortex_SRR1927040
sALS_FrontalCortex_SRR1927042
sALS_FrontalCortex_SRR1927044
sALS_FrontalCortex_SRR1927046
sALS_FrontalCortex_SRR1927048
sALS_FrontalCortex_SRR1927050
sALS_FrontalCortex_SRR1927052
sALS_FrontalCortex_SRR1927054
56838754
56839688
56840785
56841937
chr16:56838753-56841937:+
NUP93 | NUP93-206
NUP93 | NUP93-209
NUP93 | NUP93-218
NUP93 | NUP93-202
NUP93 | NUP93-210
NUP93 | NUP93-220
NUP93 | NUP93-201
NUP93 | NUP93-207
NUP93 | NUP93-204

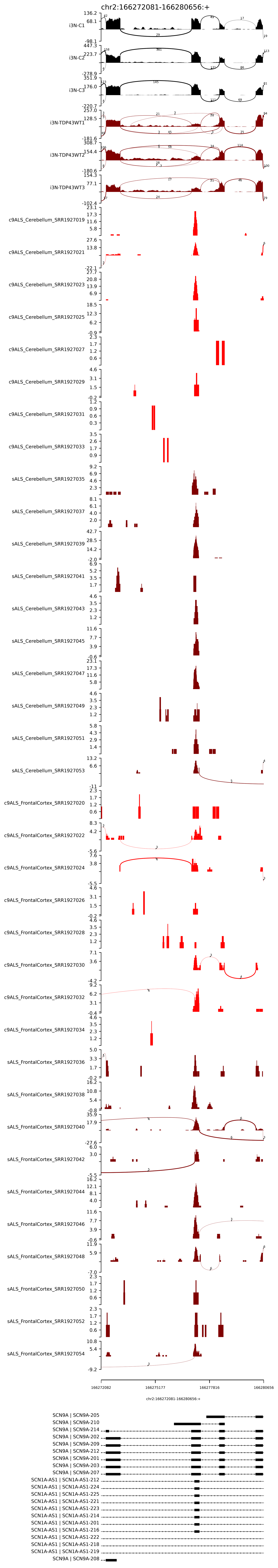

chr2:166272081-166280656:+
i3N-C1
i3N-C2
i3N-C3
i3N-TDP43WT1
i3N-TDP43WT2
i3N-TDP43WT3
c9ALS_Cerebellum_SRR1927019
c9ALS_Cerebellum_SRR1927021
c9ALS_Cerebellum_SRR1927023
c9ALS_Cerebellum_SRR1927025
c9ALS_Cerebellum_SRR1927027
c9ALS_Cerebellum_SRR1927029
c9ALS_Cerebellum_SRR1927031
c9ALS_Cerebellum_SRR1927033
sALS_Cerebellum_SRR1927035
sALS_Cerebellum_SRR1927037
sALS_Cerebellum_SRR1927039
sALS_Cerebellum_SRR1927041
sALS_Cerebellum_SRR1927043
sALS_Cerebellum_SRR1927045
sALS_Cerebellum_SRR1927047
sALS_Cerebellum_SRR1927049
sALS_Cerebellum_SRR1927051
sALS_Cerebellum_SRR1927053
c9ALS_FrontalCortex_SRR1927020
c9ALS_FrontalCortex_SRR1927022
c9ALS_FrontalCortex_SRR1927024
c9ALS_FrontalCortex_SRR1927026
c9ALS_FrontalCortex_SRR1927028
c9ALS_FrontalCortex_SRR1927030
c9ALS_FrontalCortex_SRR1927032
c9ALS_FrontalCortex_SRR1927034
sALS_FrontalCortex_SRR1927036
sALS_FrontalCortex_SRR1927038
sALS_FrontalCortex_SRR1927040
sALS_FrontalCortex_SRR1927042
sALS_FrontalCortex_SRR1927044
sALS_FrontalCortex_SRR1927046
sALS_FrontalCortex_SRR1927048
sALS_FrontalCortex_SRR1927050
sALS_FrontalCortex_SRR1927052
sALS_FrontalCortex_SRR1927054
166272082
166275177
166277816
166280656
chr2:166272081-166280656:+
SCN9A | SCN9A-205
SCN9A | SCN9A-210
SCN9A | SCN9A-214
SCN9A | SCN9A-202
SCN9A | SCN9A-209
SCN9A | SCN9A-212
SCN9A | SCN9A-201
SCN9A | SCN9A-203
SCN9A | SCN9A-207
SCN1A-AS1 | SCN1A-AS1-212
SCN1A-AS1 | SCN1A-AS1-224
SCN1A-AS1 | SCN1A-AS1-225
SCN1A-AS1 | SCN1A-AS1-221
SCN1A-AS1 | SCN1A-AS1-223
SCN1A-AS1 | SCN1A-AS1-214
SCN1A-AS1 | SCN1A-AS1-201
SCN1A-AS1 | SCN1A-AS1-216
SCN1A-AS1 | SCN1A-AS1-222
SCN1A-AS1 | SCN1A-AS1-218
SCN1A-AS1 | SCN1A-AS1-219
SCN9A | SCN9A-208

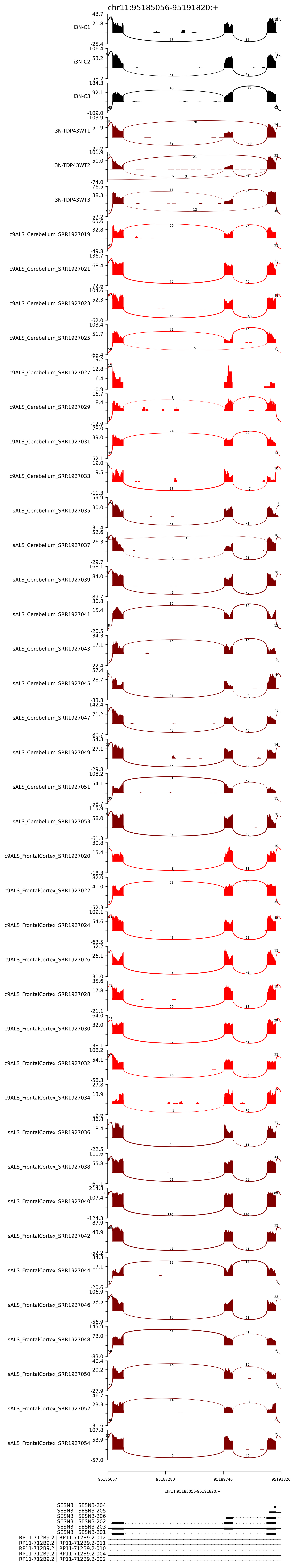
chr11:95185056-95191820:+
i3N-C1
i3N-C2
i3N-C3
i3N-TDP43WT1
i3N-TDP43WT2
i3N-TDP43WT3
c9ALS_Cerebellum_SRR1927019
c9ALS_Cerebellum_SRR1927021
c9ALS_Cerebellum_SRR1927023
c9ALS_Cerebellum_SRR1927025
c9ALS_Cerebellum_SRR1927027
c9ALS_Cerebellum_SRR1927029
c9ALS_Cerebellum_SRR1927031
c9ALS_Cerebellum_SRR1927033
sALS_Cerebellum_SRR1927035
sALS_Cerebellum_SRR1927037
sALS_Cerebellum_SRR1927039
sALS_Cerebellum_SRR1927041
sALS_Cerebellum_SRR1927043
sALS_Cerebellum_SRR1927045
sALS_Cerebellum_SRR1927047
sALS_Cerebellum_SRR1927049
sALS_Cerebellum_SRR1927051
sALS_Cerebellum_SRR1927053
c9ALS_FrontalCortex_SRR1927020
c9ALS_FrontalCortex_SRR1927022
c9ALS_FrontalCortex_SRR1927024
c9ALS_FrontalCortex_SRR1927026
c9ALS_FrontalCortex_SRR1927028
c9ALS_FrontalCortex_SRR1927030
c9ALS_FrontalCortex_SRR1927032
c9ALS_FrontalCortex_SRR1927034
sALS_FrontalCortex_SRR1927036
sALS_FrontalCortex_SRR1927038
sALS_FrontalCortex_SRR1927040
sALS_FrontalCortex_SRR1927042
sALS_FrontalCortex_SRR1927044
sALS_FrontalCortex_SRR1927046
sALS_FrontalCortex_SRR1927048
sALS_FrontalCortex_SRR1927050
sALS_FrontalCortex_SRR1927052
sALS_FrontalCortex_SRR1927054
95185057
95187280
95189740
95191820
chr11:95185056-95191820:+
SESN3 | SESN3-204
SESN3 | SESN3-205
SESN3 | SESN3-206
SESN3 | SESN3-202
SESN3 | SESN3-203
SESN3 | SESN3-201
RP11-712B9.2 | RP11-712B9.2-012
RP11-712B9.2 | RP11-712B9.2-011
RP11-712B9.2 | RP11-712B9.2-010
RP11-712B9.2 | RP11-712B9.2-004
RP11-712B9.2 | RP11-712B9.2-002

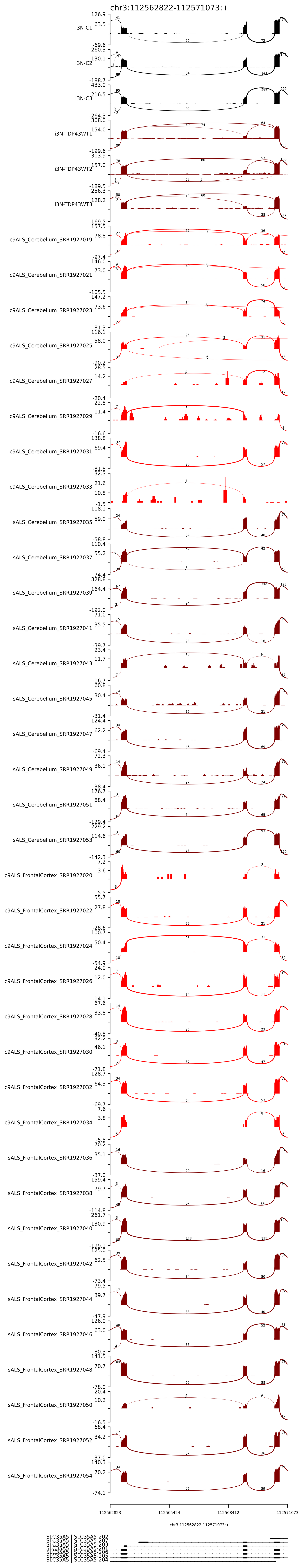
chr3:112562822-112571073:+
i3N-C1
i3N-C2
i3N-C3
i3N-TDP43WT1
i3N-TDP43WT2
i3N-TDP43WT3
c9ALS_Cerebellum_SRR1927019
c9ALS_Cerebellum_SRR1927021
c9ALS_Cerebellum_SRR1927023
c9ALS_Cerebellum_SRR1927025
c9ALS_Cerebellum_SRR1927027
c9ALS_Cerebellum_SRR1927029
c9ALS_Cerebellum_SRR1927031
c9ALS_Cerebellum_SRR1927033
sALS_Cerebellum_SRR1927035
sALS_Cerebellum_SRR1927037
sALS_Cerebellum_SRR1927039
sALS_Cerebellum_SRR1927041
sALS_Cerebellum_SRR1927043
sALS_Cerebellum_SRR1927045
sALS_Cerebellum_SRR1927047
sALS_Cerebellum_SRR1927049
sALS_Cerebellum_SRR1927051
sALS_Cerebellum_SRR1927053
c9ALS_FrontalCortex_SRR1927020
c9ALS_FrontalCortex_SRR1927022
c9ALS_FrontalCortex_SRR1927024
c9ALS_FrontalCortex_SRR1927026
c9ALS_FrontalCortex_SRR1927028
c9ALS_FrontalCortex_SRR1927030
c9ALS_FrontalCortex_SRR1927032
c9ALS_FrontalCortex_SRR1927034
sALS_FrontalCortex_SRR1927036
sALS_FrontalCortex_SRR1927038
sALS_FrontalCortex_SRR1927040
sALS_FrontalCortex_SRR1927042
sALS_FrontalCortex_SRR1927044
sALS_FrontalCortex_SRR1927046
sALS_FrontalCortex_SRR1927048
sALS_FrontalCortex_SRR1927050
sALS_FrontalCortex_SRR1927052
sALS_FrontalCortex_SRR1927054
112562823
112565424
112568412
112571073
chr3:112562822-112571073:+
SLC35A5 | SLC35A5-202
SLC35A5 | SLC35A5-207
SLC35A5 | SLC35A5-203
SLC35A5 | SLC35A5-201
SLC35A5 | SLC35A5-206
SLC35A5 | SLC35A5-205
SLC35A5 | SLC35A5-204

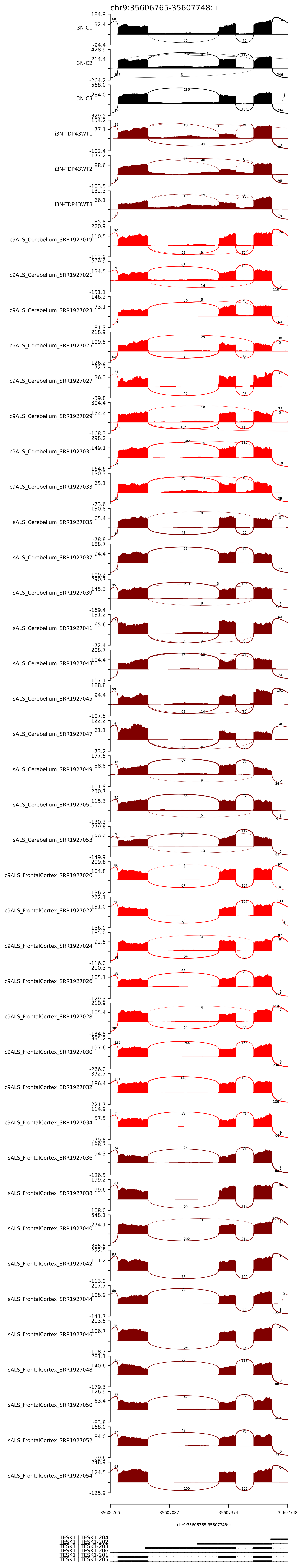
chr9:35606765-35607748:+
i3N-C1
i3N-C2
i3N-C3
i3N-TDP43WT1
i3N-TDP43WT2
i3N-TDP43WT3
c9ALS_Cerebellum_SRR1927019
c9ALS_Cerebellum_SRR1927021
c9ALS_Cerebellum_SRR1927023
c9ALS_Cerebellum_SRR1927025
c9ALS_Cerebellum_SRR1927027
c9ALS_Cerebellum_SRR1927029
c9ALS_Cerebellum_SRR1927031
c9ALS_Cerebellum_SRR1927033
sALS_Cerebellum_SRR1927035
sALS_Cerebellum_SRR1927037
sALS_Cerebellum_SRR1927039
sALS_Cerebellum_SRR1927041
sALS_Cerebellum_SRR1927043
sALS_Cerebellum_SRR1927045
sALS_Cerebellum_SRR1927047
sALS_Cerebellum_SRR1927049
sALS_Cerebellum_SRR1927051
sALS_Cerebellum_SRR1927053
c9ALS_FrontalCortex_SRR1927020
c9ALS_FrontalCortex_SRR1927022
c9ALS_FrontalCortex_SRR1927024
c9ALS_FrontalCortex_SRR1927026
c9ALS_FrontalCortex_SRR1927028
c9ALS_FrontalCortex_SRR1927030
c9ALS_FrontalCortex_SRR1927032
c9ALS_FrontalCortex_SRR1927034
sALS_FrontalCortex_SRR1927036
sALS_FrontalCortex_SRR1927038
sALS_FrontalCortex_SRR1927040
sALS_FrontalCortex_SRR1927042
sALS_FrontalCortex_SRR1927044
sALS_FrontalCortex_SRR1927046
sALS_FrontalCortex_SRR1927048
sALS_FrontalCortex_SRR1927050
sALS_FrontalCortex_SRR1927052
sALS_FrontalCortex_SRR1927054
35606766
35607087
35607374
35607748
chr9:35606765-35607748:+
TESK1 | TESK1-204
TESK1 | TESK1-202
TESK1 | TESK1-203
TESK1 | TESK1-206
TESK1 | TESK1-201
TESK1 | TESK1-205

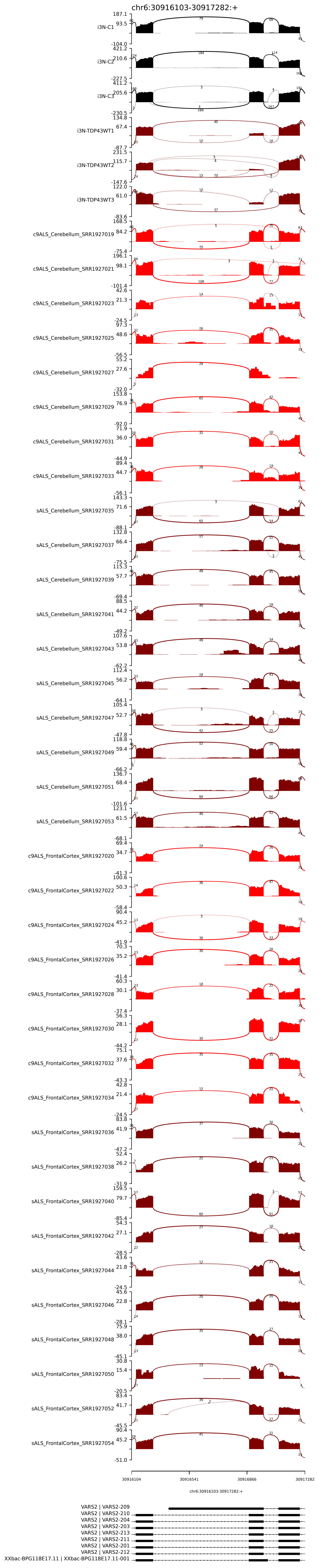
chr6:30916103-30917282:+
i3N-C1
i3N-C2
i3N-C3
i3N-TDP43WT1
i3N-TDP43WT2
i3N-TDP43WT3
c9ALS_Cerebellum_SRR1927019
c9ALS_Cerebellum_SRR1927021
c9ALS_Cerebellum_SRR1927023
c9ALS_Cerebellum_SRR1927025
c9ALS_Cerebellum_SRR1927027
c9ALS_Cerebellum_SRR1927029
c9ALS_Cerebellum_SRR1927031
c9ALS_Cerebellum_SRR1927033
sALS_Cerebellum_SRR1927035
sALS_Cerebellum_SRR1927037
sALS_Cerebellum_SRR1927039
sALS_Cerebellum_SRR1927041
sALS_Cerebellum_SRR1927043
sALS_Cerebellum_SRR1927045
sALS_Cerebellum_SRR1927047
sALS_Cerebellum_SRR1927049
sALS_Cerebellum_SRR1927051
sALS_Cerebellum_SRR1927053
c9ALS_FrontalCortex_SRR1927020
c9ALS_FrontalCortex_SRR1927022
c9ALS_FrontalCortex_SRR1927024
c9ALS_FrontalCortex_SRR1927026
c9ALS_FrontalCortex_SRR1927028
c9ALS_FrontalCortex_SRR1927030
c9ALS_FrontalCortex_SRR1927032
c9ALS_FrontalCortex_SRR1927034
sALS_FrontalCortex_SRR1927036
sALS_FrontalCortex_SRR1927038
sALS_FrontalCortex_SRR1927040
sALS_FrontalCortex_SRR1927042
sALS_FrontalCortex_SRR1927044
sALS_FrontalCortex_SRR1927046
sALS_FrontalCortex_SRR1927048
sALS_FrontalCortex_SRR1927050
sALS_FrontalCortex_SRR1927052
sALS_FrontalCortex_SRR1927054
30916104
30916541
30916866
30917282
chr6:30916103-30917282:+
VARS2 | VARS2-209
VARS2 | VARS2-210
VARS2 | VARS2-204
VARS2 | VARS2-203
VARS2 | VARS2-213
VARS2 | VARS2-211
VARS2 | VARS2-201
VARS2 | VARS2-212
XXbac-BPG118E17.11 | XXbac-BPG118E17.11-001

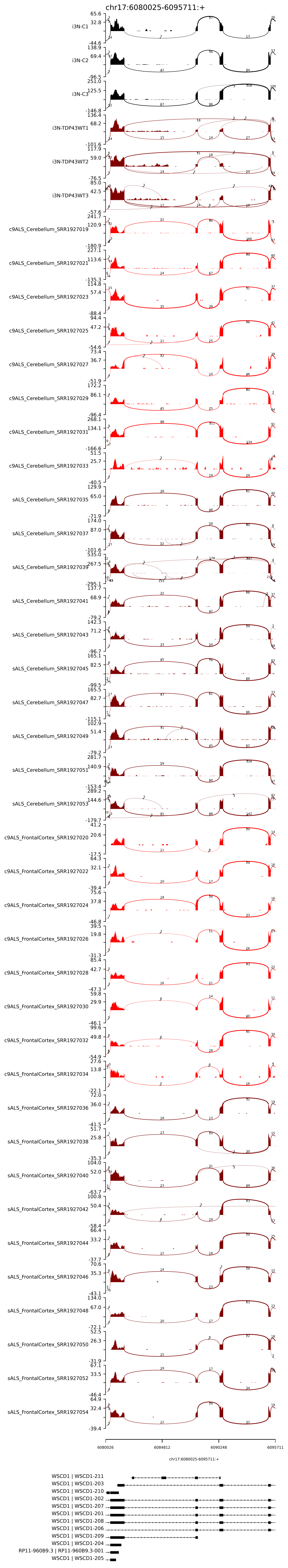
chr17:6080025-6095711:+
i3N-C1
i3N-C2
i3N-C3
i3N-TDP43WT1
i3N-TDP43WT2
i3N-TDP43WT3
c9ALS_Cerebellum_SRR1927019
c9ALS_Cerebellum_SRR1927021
c9ALS_Cerebellum_SRR1927023
c9ALS_Cerebellum_SRR1927025
c9ALS_Cerebellum_SRR1927027
c9ALS_Cerebellum_SRR1927029
c9ALS_Cerebellum_SRR1927031
c9ALS_Cerebellum_SRR1927033
sALS_Cerebellum_SRR1927035
sALS_Cerebellum_SRR1927037
sALS_Cerebellum_SRR1927039
sALS_Cerebellum_SRR1927041
sALS_Cerebellum_SRR1927043
sALS_Cerebellum_SRR1927045
sALS_Cerebellum_SRR1927047
sALS_Cerebellum_SRR1927049
sALS_Cerebellum_SRR1927051
sALS_Cerebellum_SRR1927053
c9ALS_FrontalCortex_SRR1927020
c9ALS_FrontalCortex_SRR1927022
c9ALS_FrontalCortex_SRR1927024
c9ALS_FrontalCortex_SRR1927026
c9ALS_FrontalCortex_SRR1927028
c9ALS_FrontalCortex_SRR1927030
c9ALS_FrontalCortex_SRR1927032
c9ALS_FrontalCortex_SRR1927034
sALS_FrontalCortex_SRR1927036
sALS_FrontalCortex_SRR1927038
sALS_FrontalCortex_SRR1927040
sALS_FrontalCortex_SRR1927042
sALS_FrontalCortex_SRR1927044
sALS_FrontalCortex_SRR1927046
sALS_FrontalCortex_SRR1927048
sALS_FrontalCortex_SRR1927050
sALS_FrontalCortex_SRR1927052
sALS_FrontalCortex_SRR1927054
6080026
6084812
6090248
6095711
chr17:6080025-6095711:+
WSCD1 | WSCD1-211
WSCD1 | WSCD1-203
WSCD1 | WSCD1-210
WSCD1 | WSCD1-202
WSCD1 | WSCD1-207
WSCD1 | WSCD1-201
WSCD1 | WSCD1-208
WSCD1 | WSCD1-206
WSCD1 | WSCD1-209
WSCD1 | WSCD1-204
RP11-960B9.3 | RP11-960B9.3-001
WSCD1 | WSCD1-205

chr10:109883737-109888276:+

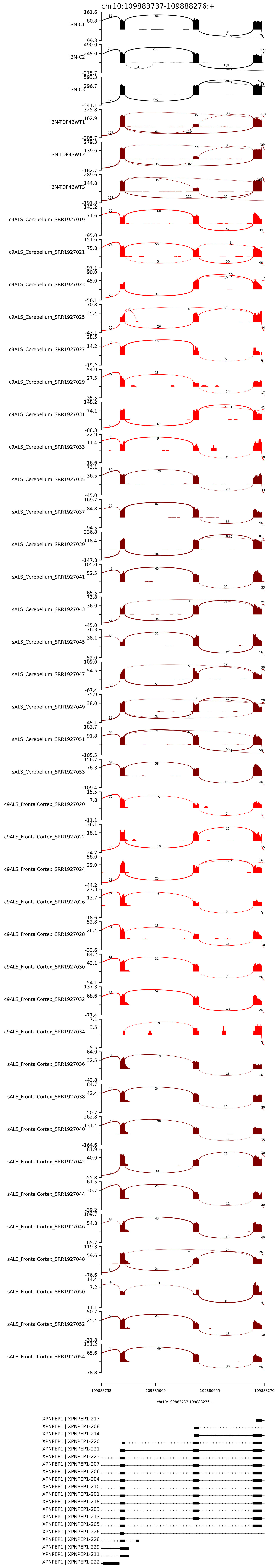

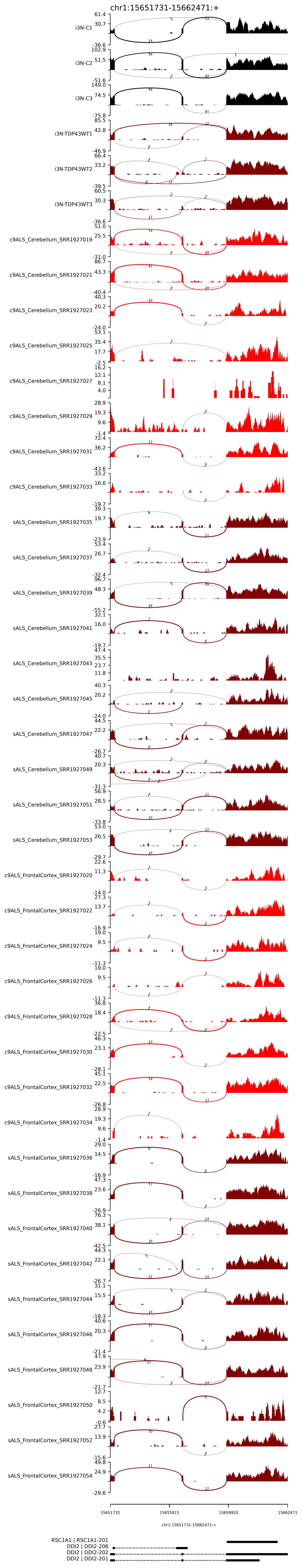
chr1:15651731-15662471:+
i3N-C1
i3N-C2
i3N-C3
i3N-TDP43WT1
i3N-TDP43WT2
i3N-TDP43WT3
c9ALS_Cerebellum_SRR1927019
c9ALS_Cerebellum_SRR1927021
c9ALS_Cerebellum_SRR1927023
c9ALS_Cerebellum_SRR1927025
c9ALS_Cerebellum_SRR1927027
c9ALS_Cerebellum_SRR1927029
c9ALS_Cerebellum_SRR1927031
c9ALS_Cerebellum_SRR1927033
sALS_Cerebellum_SRR1927035
sALS_Cerebellum_SRR1927037
sALS_Cerebellum_SRR1927039
sALS_Cerebellum_SRR1927041
sALS_Cerebellum_SRR1927043
sALS_Cerebellum_SRR1927045
sALS_Cerebellum_SRR1927047
sALS_Cerebellum_SRR1927049
sALS_Cerebellum_SRR1927051
sALS_Cerebellum_SRR1927053
c9ALS_FrontalCortex_SRR1927020
c9ALS_FrontalCortex_SRR1927022
c9ALS_FrontalCortex_SRR1927024
c9ALS_FrontalCortex_SRR1927026
c9ALS_FrontalCortex_SRR1927028
c9ALS_FrontalCortex_SRR1927030
c9ALS_FrontalCortex_SRR1927032
c9ALS_FrontalCortex_SRR1927034
sALS_FrontalCortex_SRR1927036
sALS_FrontalCortex_SRR1927038
sALS_FrontalCortex_SRR1927040
sALS_FrontalCortex_SRR1927042
sALS_FrontalCortex_SRR1927044
sALS_FrontalCortex_SRR1927046
sALS_FrontalCortex_SRR1927048
sALS_FrontalCortex_SRR1927050
sALS_FrontalCortex_SRR1927052
sALS_FrontalCortex_SRR1927054
15651732
15655915
15659920
15662471
chr1:15651731-15662471:+
RSC1A1 | RSC1A1-201
DDI2 | DDI2-206
DDI2 | DDI2-202
DDI2 | DDI2-201

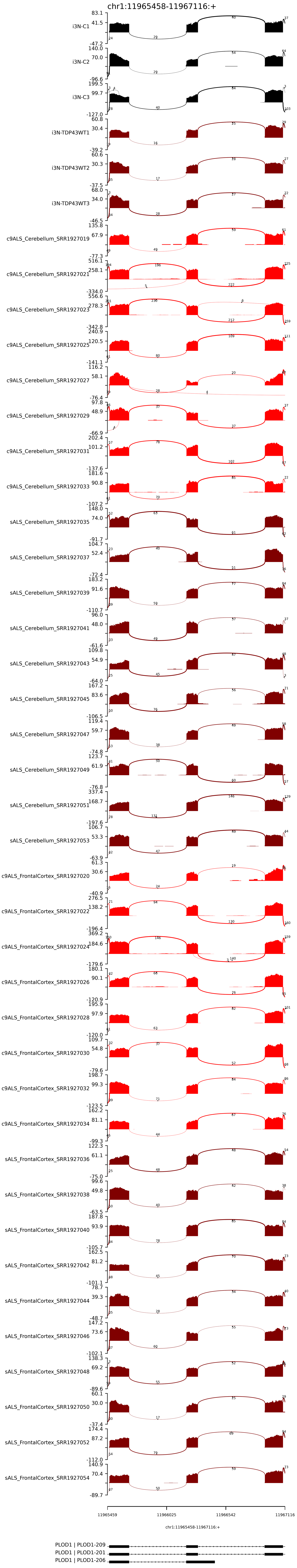

chr1:11965458-11967116:+
i3N-C1
i3N-C2
i3N-C3
i3N-TDP43WT1
i3N-TDP43WT2
i3N-TDP43WT3
c9ALS_Cerebellum_SRR1927019
c9ALS_Cerebellum_SRR1927021
c9ALS_Cerebellum_SRR1927023
c9ALS_Cerebellum_SRR1927025
c9ALS_Cerebellum_SRR1927027
c9ALS_Cerebellum_SRR1927029
c9ALS_Cerebellum_SRR1927031
c9ALS_Cerebellum_SRR1927033
sALS_Cerebellum_SRR1927035
sALS_Cerebellum_SRR1927037
sALS_Cerebellum_SRR1927039
sALS_Cerebellum_SRR1927041
sALS_Cerebellum_SRR1927043
sALS_Cerebellum_SRR1927045
sALS_Cerebellum_SRR1927047
sALS_Cerebellum_SRR1927049
sALS_Cerebellum_SRR1927051
sALS_Cerebellum_SRR1927053
c9ALS_FrontalCortex_SRR1927020
c9ALS_FrontalCortex_SRR1927022
c9ALS_FrontalCortex_SRR1927024
c9ALS_FrontalCortex_SRR1927026
c9ALS_FrontalCortex_SRR1927028
c9ALS_FrontalCortex_SRR1927030
c9ALS_FrontalCortex_SRR1927032
c9ALS_FrontalCortex_SRR1927034
sALS_FrontalCortex_SRR1927036
sALS_FrontalCortex_SRR1927038
sALS_FrontalCortex_SRR1927040
sALS_FrontalCortex_SRR1927042
sALS_FrontalCortex_SRR1927044
sALS_FrontalCortex_SRR1927046
sALS_FrontalCortex_SRR1927048
sALS_FrontalCortex_SRR1927050
sALS_FrontalCortex_SRR1927052
sALS_FrontalCortex_SRR1927054
11965459
11966025
11966542
11967116
chr1:11965458-11967116:+
PLOD1 | PLOD1-209
PLOD1 | PLOD1-201
PLOD1 | PLOD1-206

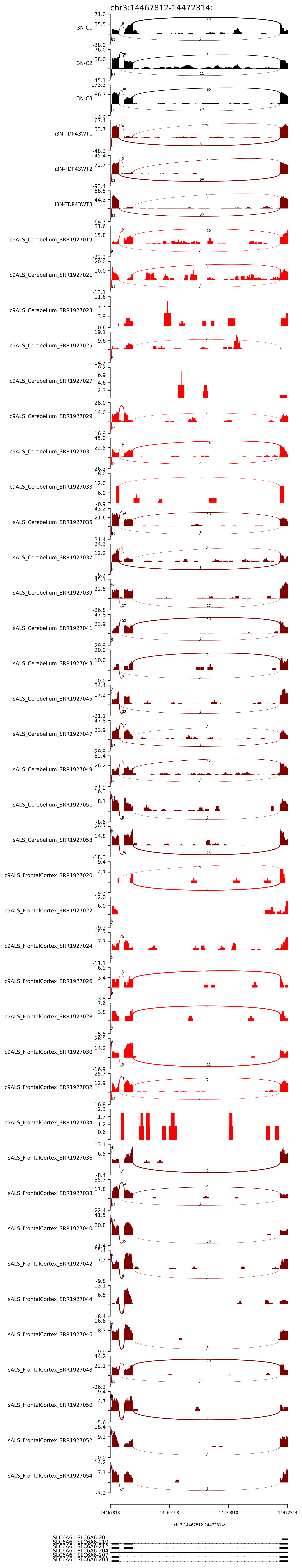
chr3:14467812-14472314:+
i3N-C1
i3N-C2
i3N-C3
i3N-TDP43WT1
i3N-TDP43WT2
i3N-TDP43WT3
c9ALS_Cerebellum_SRR1927019
c9ALS_Cerebellum_SRR1927021
c9ALS_Cerebellum_SRR1927023
c9ALS_Cerebellum_SRR1927025
c9ALS_Cerebellum_SRR1927027
c9ALS_Cerebellum_SRR1927029
c9ALS_Cerebellum_SRR1927031
c9ALS_Cerebellum_SRR1927033
sALS_Cerebellum_SRR1927035
sALS_Cerebellum_SRR1927037
sALS_Cerebellum_SRR1927039
sALS_Cerebellum_SRR1927041
sALS_Cerebellum_SRR1927043
sALS_Cerebellum_SRR1927045
sALS_Cerebellum_SRR1927047
sALS_Cerebellum_SRR1927049
sALS_Cerebellum_SRR1927051
sALS_Cerebellum_SRR1927053
c9ALS_FrontalCortex_SRR1927020
c9ALS_FrontalCortex_SRR1927022
c9ALS_FrontalCortex_SRR1927024
c9ALS_FrontalCortex_SRR1927026
c9ALS_FrontalCortex_SRR1927028
c9ALS_FrontalCortex_SRR1927030
c9ALS_FrontalCortex_SRR1927032
c9ALS_FrontalCortex_SRR1927034
sALS_FrontalCortex_SRR1927036
sALS_FrontalCortex_SRR1927038
sALS_FrontalCortex_SRR1927040
sALS_FrontalCortex_SRR1927042
sALS_FrontalCortex_SRR1927044
sALS_FrontalCortex_SRR1927046
sALS_FrontalCortex_SRR1927048
sALS_FrontalCortex_SRR1927050
sALS_FrontalCortex_SRR1927052
sALS_FrontalCortex_SRR1927054
14467813
14469198
14470810
14472314
chr3:14467812-14472314:+
SLC6A6 | SLC6A6-201
SLC6A6 | SLC6A6-210
SLC6A6 | SLC6A6-212
SLC6A6 | SLC6A6-204
SLC6A6 | SLC6A6-207
SLC6A6 | SLC6A6-203

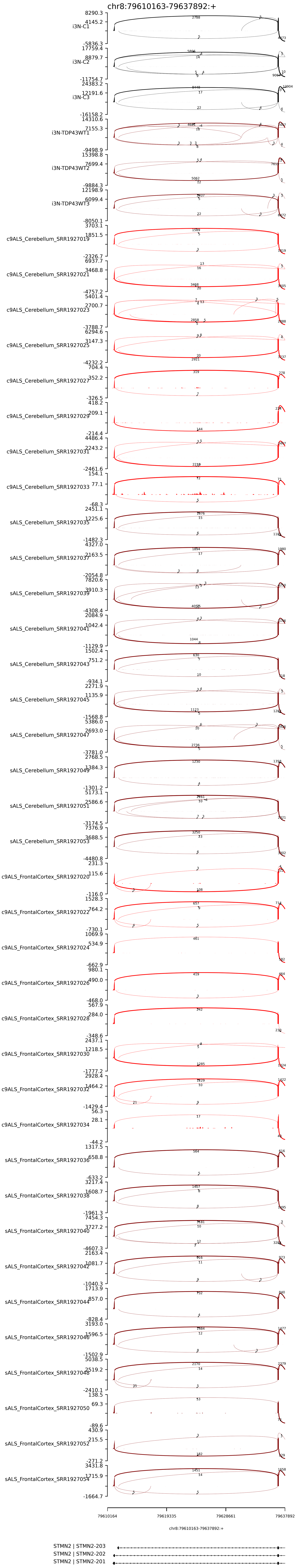

chr8:79610163-79637892:+
i3N-C1
i3N-C2
i3N-C3
i3N-TDP43WT1
i3N-TDP43WT2
i3N-TDP43WT3
c9ALS_Cerebellum_SRR1927019
c9ALS_Cerebellum_SRR1927021
c9ALS_Cerebellum_SRR1927023
c9ALS_Cerebellum_SRR1927025
c9ALS_Cerebellum_SRR1927027
c9ALS_Cerebellum_SRR1927029
c9ALS_Cerebellum_SRR1927031
c9ALS_Cerebellum_SRR1927033
sALS_Cerebellum_SRR1927035
sALS_Cerebellum_SRR1927037
sALS_Cerebellum_SRR1927039
sALS_Cerebellum_SRR1927041
sALS_Cerebellum_SRR1927043
sALS_Cerebellum_SRR1927045
sALS_Cerebellum_SRR1927047
sALS_Cerebellum_SRR1927049
sALS_Cerebellum_SRR1927051
sALS_Cerebellum_SRR1927053
c9ALS_FrontalCortex_SRR1927020
c9ALS_FrontalCortex_SRR1927022
c9ALS_FrontalCortex_SRR1927024
c9ALS_FrontalCortex_SRR1927026
c9ALS_FrontalCortex_SRR1927028
c9ALS_FrontalCortex_SRR1927030
c9ALS_FrontalCortex_SRR1927032
c9ALS_FrontalCortex_SRR1927034
sALS_FrontalCortex_SRR1927036
sALS_FrontalCortex_SRR1927038
sALS_FrontalCortex_SRR1927040
sALS_FrontalCortex_SRR1927042
sALS_FrontalCortex_SRR1927044
sALS_FrontalCortex_SRR1927046
sALS_FrontalCortex_SRR1927048
sALS_FrontalCortex_SRR1927050
sALS_FrontalCortex_SRR1927052
sALS_FrontalCortex_SRR1927054
79610164
79619335
79628661
79637892
chr8:79610163-79637892:+
STMN2 | STMN2-203
STMN2 | STMN2-202
STMN2 | STMN2-201

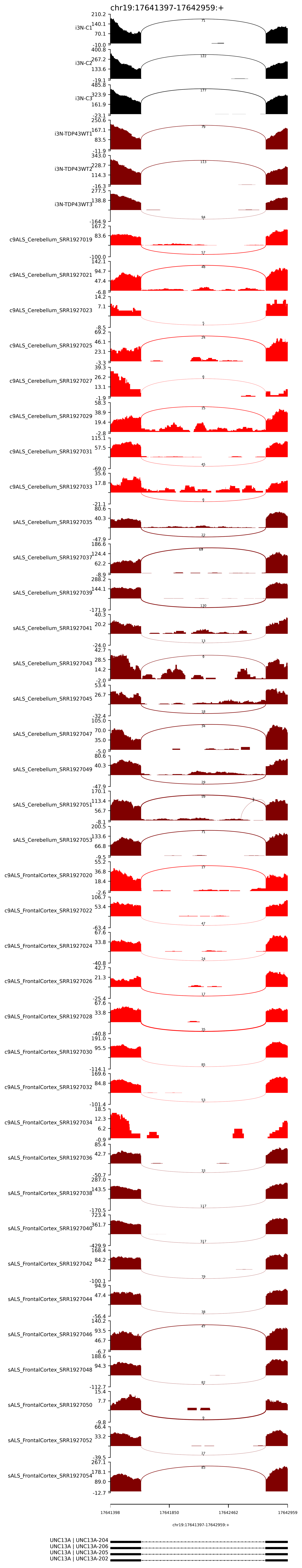
chr19:17641397-17642959:+
i3N-C1
i3N-C2
i3N-C3
i3N-TDP43WT1
i3N-TDP43WT2
i3N-TDP43WT3
c9ALS_Cerebellum_SRR1927019
c9ALS_Cerebellum_SRR1927021
c9ALS_Cerebellum_SRR1927023
c9ALS_Cerebellum_SRR1927025
c9ALS_Cerebellum_SRR1927027
c9ALS_Cerebellum_SRR1927029
c9ALS_Cerebellum_SRR1927031
c9ALS_Cerebellum_SRR1927033
sALS_Cerebellum_SRR1927035
sALS_Cerebellum_SRR1927037
sALS_Cerebellum_SRR1927039
sALS_Cerebellum_SRR1927041
sALS_Cerebellum_SRR1927043
sALS_Cerebellum_SRR1927045
sALS_Cerebellum_SRR1927047
sALS_Cerebellum_SRR1927049
sALS_Cerebellum_SRR1927051
sALS_Cerebellum_SRR1927053
c9ALS_FrontalCortex_SRR1927020
c9ALS_FrontalCortex_SRR1927022
c9ALS_FrontalCortex_SRR1927024
c9ALS_FrontalCortex_SRR1927026
c9ALS_FrontalCortex_SRR1927028
c9ALS_FrontalCortex_SRR1927030
c9ALS_FrontalCortex_SRR1927032
c9ALS_FrontalCortex_SRR1927034
sALS_FrontalCortex_SRR1927036
sALS_FrontalCortex_SRR1927038
sALS_FrontalCortex_SRR1927040
sALS_FrontalCortex_SRR1927042
sALS_FrontalCortex_SRR1927044
sALS_FrontalCortex_SRR1927046
sALS_FrontalCortex_SRR1927048
sALS_FrontalCortex_SRR1927050
sALS_FrontalCortex_SRR1927052
sALS_FrontalCortex_SRR1927054
17641398
17641850
17642462
17642959
chr19:17641397-17642959:+
UNC13A | UNC13A-204
UNC13A | UNC13A-206
UNC13A | UNC13A-205
UNC13A | UNC13A-202

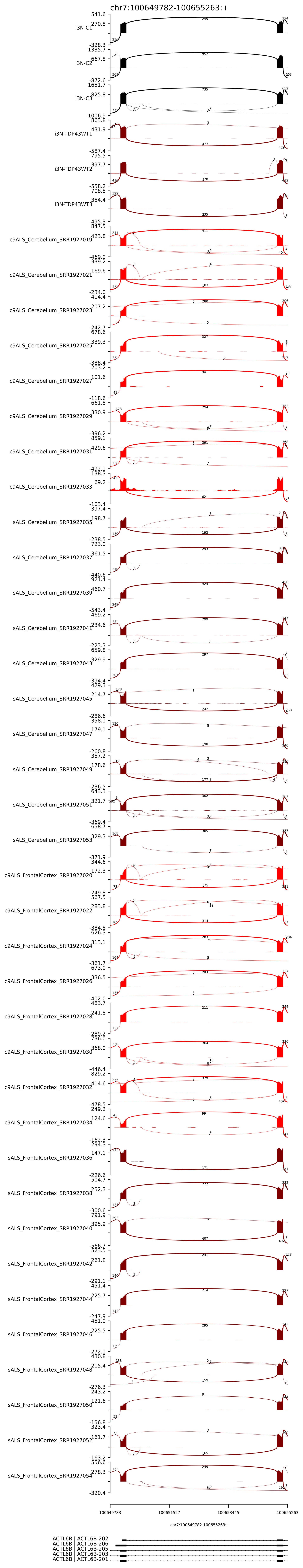

chr7:100649782-100655263:+
i3N-C1
i3N-C2
i3N-C3
i3N-TDP43WT1
i3N-TDP43WT2
i3N-TDP43WT3
c9ALS_Cerebellum_SRR1927019
c9ALS_Cerebellum_SRR1927021
c9ALS_Cerebellum_SRR1927023
c9ALS_Cerebellum_SRR1927025
c9ALS_Cerebellum_SRR1927027
c9ALS_Cerebellum_SRR1927029
c9ALS_Cerebellum_SRR1927031
c9ALS_Cerebellum_SRR1927033
sALS_Cerebellum_SRR1927035
sALS_Cerebellum_SRR1927037
sALS_Cerebellum_SRR1927039
sALS_Cerebellum_SRR1927041
sALS_Cerebellum_SRR1927043
sALS_Cerebellum_SRR1927045
sALS_Cerebellum_SRR1927047
sALS_Cerebellum_SRR1927049
sALS_Cerebellum_SRR1927051
sALS_Cerebellum_SRR1927053
c9ALS_FrontalCortex_SRR1927020
c9ALS_FrontalCortex_SRR1927022
c9ALS_FrontalCortex_SRR1927024
c9ALS_FrontalCortex_SRR1927026
c9ALS_FrontalCortex_SRR1927028
c9ALS_FrontalCortex_SRR1927030
c9ALS_FrontalCortex_SRR1927032
c9ALS_FrontalCortex_SRR1927034
sALS_FrontalCortex_SRR1927036
sALS_FrontalCortex_SRR1927038
sALS_FrontalCortex_SRR1927040
sALS_FrontalCortex_SRR1927042
sALS_FrontalCortex_SRR1927044
sALS_FrontalCortex_SRR1927046
sALS_FrontalCortex_SRR1927048
sALS_FrontalCortex_SRR1927050
sALS_FrontalCortex_SRR1927052
sALS_FrontalCortex_SRR1927054
100649783
100651527
100653445
100655263
chr7:100649782-100655263:+
ACTL6B | ACTL6B-202
ACTL6B | ACTL6B-206
ACTL6B | ACTL6B-205
ACTL6B | ACTL6B-203
ACTL6B | ACTL6B-201

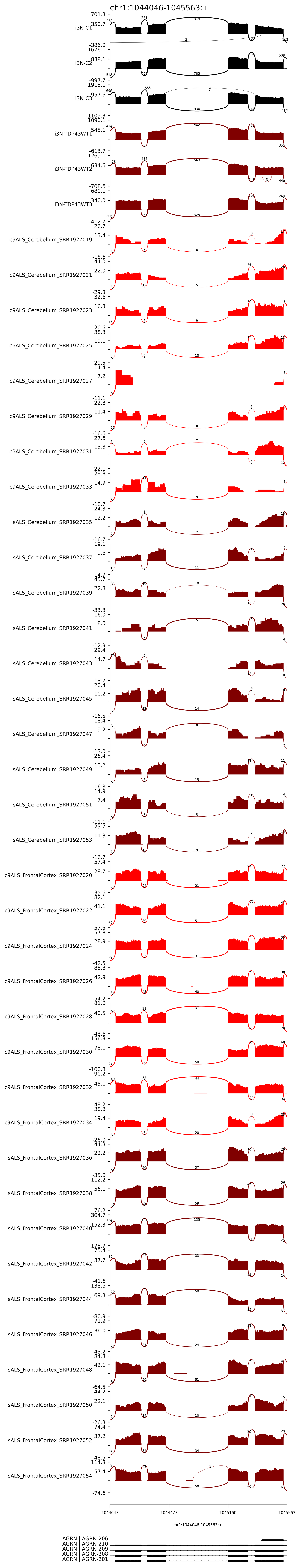

chr1:1044046-1045563:+
i3N-C1
i3N-C2
i3N-C3
i3N-TDP43WT1
i3N-TDP43WT2
i3N-TDP43WT3
c9ALS_Cerebellum_SRR1927019
c9ALS_Cerebellum_SRR1927021
c9ALS_Cerebellum_SRR1927023
c9ALS_Cerebellum_SRR1927025
c9ALS_Cerebellum_SRR1927027
c9ALS_Cerebellum_SRR1927029
c9ALS_Cerebellum_SRR1927031
c9ALS_Cerebellum_SRR1927033
sALS_Cerebellum_SRR1927035
sALS_Cerebellum_SRR1927037
sALS_Cerebellum_SRR1927039
sALS_Cerebellum_SRR1927041
sALS_Cerebellum_SRR1927043
sALS_Cerebellum_SRR1927045
sALS_Cerebellum_SRR1927047
sALS_Cerebellum_SRR1927049
sALS_Cerebellum_SRR1927051
sALS_Cerebellum_SRR1927053
c9ALS_FrontalCortex_SRR1927020
c9ALS_FrontalCortex_SRR1927022
c9ALS_FrontalCortex_SRR1927024
c9ALS_FrontalCortex_SRR1927026
c9ALS_FrontalCortex_SRR1927028
c9ALS_FrontalCortex_SRR1927030
c9ALS_FrontalCortex_SRR1927032
c9ALS_FrontalCortex_SRR1927034
sALS_FrontalCortex_SRR1927036
sALS_FrontalCortex_SRR1927038
sALS_FrontalCortex_SRR1927040
sALS_FrontalCortex_SRR1927042
sALS_FrontalCortex_SRR1927044
sALS_FrontalCortex_SRR1927046
sALS_FrontalCortex_SRR1927048
sALS_FrontalCortex_SRR1927050
sALS_FrontalCortex_SRR1927052
sALS_FrontalCortex_SRR1927054
1044047
1044477
1045160
1045563
chr1:1044046-1045563:+
AGRN AGRN-206
AGRN AGRN-210
AGRN AGRN-209
AGRN AGRN-208
AGRN AGRN-201

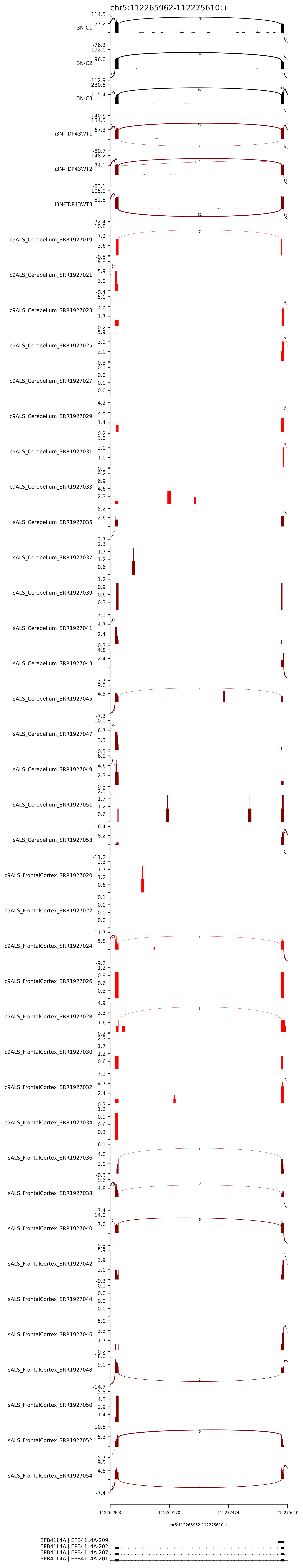
chr5:112265962-112275610:+
i3N-C1
i3N-C2
i3N-C3
i3N-TDP43WT1
i3N-TDP43WT2
i3N-TDP43WT3
c9ALS_Cerebellum_SRR1927019
c9ALS_Cerebellum_SRR1927021
c9ALS_Cerebellum_SRR1927023
c9ALS_Cerebellum_SRR1927025
c9ALS_Cerebellum_SRR1927027
c9ALS_Cerebellum_SRR1927029
c9ALS_Cerebellum_SRR1927031
c9ALS_Cerebellum_SRR1927033
sALS_Cerebellum_SRR1927035
sALS_Cerebellum_SRR1927037
sALS_Cerebellum_SRR1927039
sALS_Cerebellum_SRR1927041
sALS_Cerebellum_SRR1927043
sALS_Cerebellum_SRR1927045
sALS_Cerebellum_SRR1927047
sALS_Cerebellum_SRR1927049
sALS_Cerebellum_SRR1927051
sALS_Cerebellum_SRR1927053
c9ALS_FrontalCortex_SRR1927020
c9ALS_FrontalCortex_SRR1927022
c9ALS_FrontalCortex_SRR1927024
c9ALS_FrontalCortex_SRR1927026
c9ALS_FrontalCortex_SRR1927028
c9ALS_FrontalCortex_SRR1927030
c9ALS_FrontalCortex_SRR1927032
c9ALS_FrontalCortex_SRR1927034
sALS_FrontalCortex_SRR1927036
sALS_FrontalCortex_SRR1927038
sALS_FrontalCortex_SRR1927040
sALS_FrontalCortex_SRR1927042
sALS_FrontalCortex_SRR1927044
sALS_FrontalCortex_SRR1927046
sALS_FrontalCortex_SRR1927048
sALS_FrontalCortex_SRR1927050
sALS_FrontalCortex_SRR1927052
sALS_FrontalCortex_SRR1927054
112265963
112269170
112272474
112275610
chr5:112265962-112275610:+
EPB41L4A | EPB41L4A-209
EPB41L4A | EPB41L4A-202
EPB41L4A | EPB41L4A-207
EPB41L4A | EPB41L4A-201

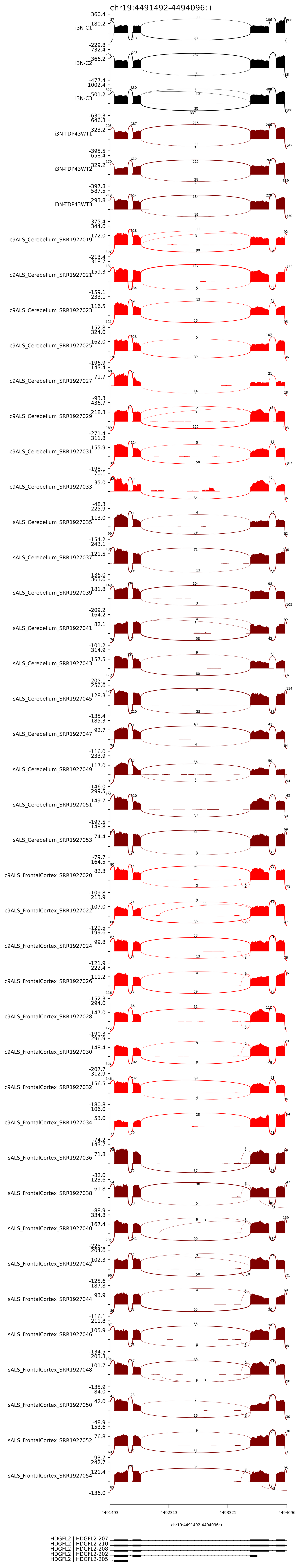

chr19:4491492-4494096:+
i3N-C1
i3N-C2
i3N-C3
i3N-TDP43WT1
i3N-TDP43WT2
i3N-TDP43WT3
c9ALS_Cerebellum_SRR1927019
c9ALS_Cerebellum_SRR1927021
c9ALS_Cerebellum_SRR1927023
c9ALS_Cerebellum_SRR1927025
c9ALS_Cerebellum_SRR1927027
c9ALS_Cerebellum_SRR1927029
c9ALS_Cerebellum_SRR1927031
c9ALS_Cerebellum_SRR1927033
sALS_Cerebellum_SRR1927035
sALS_Cerebellum_SRR1927037
sALS_Cerebellum_SRR1927039
sALS_Cerebellum_SRR1927041
sALS_Cerebellum_SRR1927043
sALS_Cerebellum_SRR1927045
sALS_Cerebellum_SRR1927047
sALS_Cerebellum_SRR1927049
sALS_Cerebellum_SRR1927051
sALS_Cerebellum_SRR1927053
c9ALS_FrontalCortex_SRR1927020
c9ALS_FrontalCortex_SRR1927022
c9ALS_FrontalCortex_SRR1927024
c9ALS_FrontalCortex_SRR1927026
c9ALS_FrontalCortex_SRR1927028
c9ALS_FrontalCortex_SRR1927030
c9ALS_FrontalCortex_SRR1927032
c9ALS_FrontalCortex_SRR1927034
sALS_FrontalCortex_SRR1927036
sALS_FrontalCortex_SRR1927038
sALS_FrontalCortex_SRR1927040
sALS_FrontalCortex_SRR1927042
sALS_FrontalCortex_SRR1927044
sALS_FrontalCortex_SRR1927046
sALS_FrontalCortex_SRR1927048
sALS_FrontalCortex_SRR1927050
sALS_FrontalCortex_SRR1927052
sALS_FrontalCortex_SRR1927054
4491493
4492313
4493321
4494096
chr19:4491492-4494096:+
HDGFL2 | HDGFL2-207
HDGFL2 | HDGFL2-210
HDGFL2 | HDGFL2-208
HDGFL2 | HDGFL2-202
HDGFL2 | HDGFL2-205

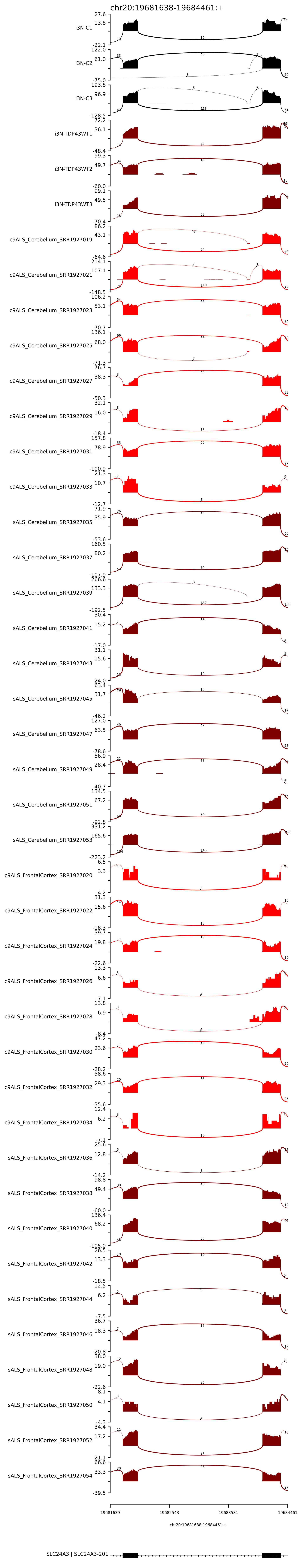
chr20:19681638-19684461:+
i3N-C1
i3N-C2
i3N-C3
i3N-TDP43WT1
i3N-TDP43WT2
i3N-TDP43WT3
c9ALS_Cerebellum_SRR1927019
c9ALS_Cerebellum_SRR1927021
c9ALS_Cerebellum_SRR1927023
c9ALS_Cerebellum_SRR1927025
c9ALS_Cerebellum_SRR1927027
c9ALS_Cerebellum_SRR1927029
c9ALS_Cerebellum_SRR1927031
c9ALS_Cerebellum_SRR1927033
sALS_Cerebellum_SRR1927035
sALS_Cerebellum_SRR1927037
sALS_Cerebellum_SRR1927039
sALS_Cerebellum_SRR1927041
sALS_Cerebellum_SRR1927043
sALS_Cerebellum_SRR1927045
sALS_Cerebellum_SRR1927047
sALS_Cerebellum_SRR1927049
sALS_Cerebellum_SRR1927051
sALS_Cerebellum_SRR1927053
c9ALS_FrontalCortex_SRR1927020
c9ALS_FrontalCortex_SRR1927022
c9ALS_FrontalCortex_SRR1927024
c9ALS_FrontalCortex_SRR1927026
c9ALS_FrontalCortex_SRR1927028
c9ALS_FrontalCortex_SRR1927030
c9ALS_FrontalCortex_SRR1927032
c9ALS_FrontalCortex_SRR1927034
sALS_FrontalCortex_SRR1927036
sALS_FrontalCortex_SRR1927038
sALS_FrontalCortex_SRR1927040
sALS_FrontalCortex_SRR1927042
sALS_FrontalCortex_SRR1927044
sALS_FrontalCortex_SRR1927046
sALS_FrontalCortex_SRR1927048
sALS_FrontalCortex_SRR1927050
sALS_FrontalCortex_SRR1927052
sALS_FrontalCortex_SRR1927054
19681639
19682543
19683581
19684461
chr20:19681638-19684461:+
SLC24A3 | SLC24A3-201

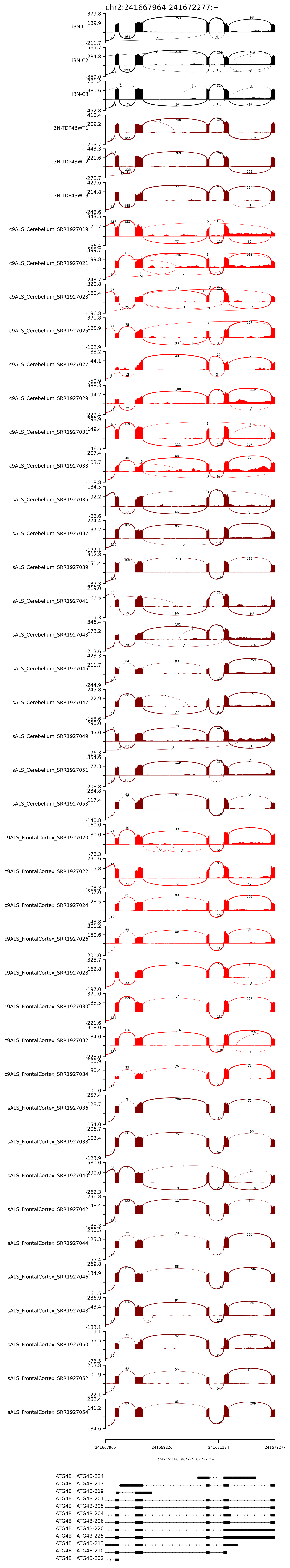

chr2:241667964-241672277:+
i3N-C1
i3N-C2
i3N-C3
i3N-TDP43WT1
i3N-TDP43WT2
i3N-TDP43WT3
c9ALS_Cerebellum_SRR1927019
c9ALS_Cerebellum_SRR1927021
c9ALS_Cerebellum_SRR1927023
c9ALS_Cerebellum_SRR1927025
c9ALS_Cerebellum_SRR1927027
c9ALS_Cerebellum_SRR1927029
c9ALS_Cerebellum_SRR1927031
c9ALS_Cerebellum_SRR1927033
sALS_Cerebellum_SRR1927035
sALS_Cerebellum_SRR1927037
sALS_Cerebellum_SRR1927039
sALS_Cerebellum_SRR1927041
sALS_Cerebellum_SRR1927043
sALS_Cerebellum_SRR1927045
sALS_Cerebellum_SRR1927047
sALS_Cerebellum_SRR1927049
sALS_Cerebellum_SRR1927051
sALS_Cerebellum_SRR1927053
c9ALS_FrontalCortex_SRR1927020
c9ALS_FrontalCortex_SRR1927022
c9ALS_FrontalCortex_SRR1927024
c9ALS_FrontalCortex_SRR1927026
c9ALS_FrontalCortex_SRR1927028
c9ALS_FrontalCortex_SRR1927030
c9ALS_FrontalCortex_SRR1927032
c9ALS_FrontalCortex_SRR1927034
sALS_FrontalCortex_SRR1927036
sALS_FrontalCortex_SRR1927038
sALS_FrontalCortex_SRR1927040
sALS_FrontalCortex_SRR1927042
sALS_FrontalCortex_SRR1927044
sALS_FrontalCortex_SRR1927046
sALS_FrontalCortex_SRR1927048
sALS_FrontalCortex_SRR1927050
sALS_FrontalCortex_SRR1927052
sALS_FrontalCortex_SRR1927054
241667965
241669226
241671124
241672277
chr2:241667964-241672277:+
ATG4B | ATG4B-224
ATG4B | ATG4B-217
ATG4B | ATG4B-219
ATG4B | ATG4B-201
ATG4B | ATG4B-205
ATG4B | ATG4B-204
ATG4B | ATG4B-206
ATG4B | ATG4B-220
ATG4B | ATG4B-225
ATG4B | ATG4B-213
ATG4B | ATG4B-210
ATG4B | ATG4B-202

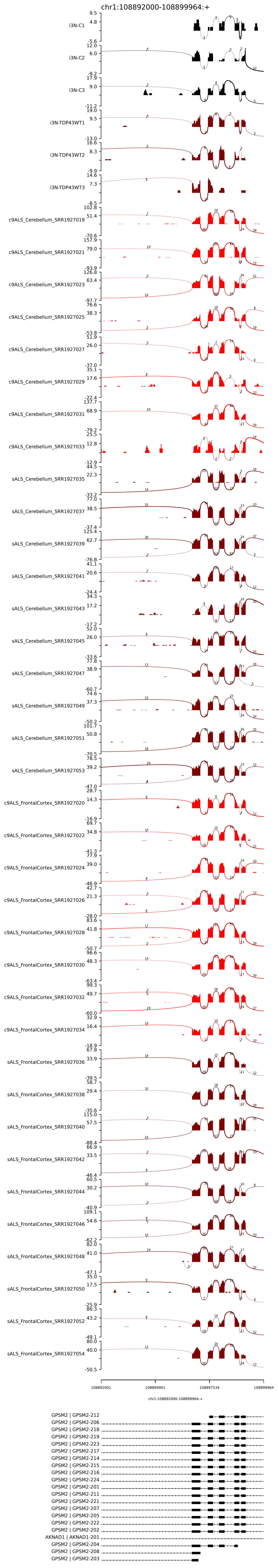

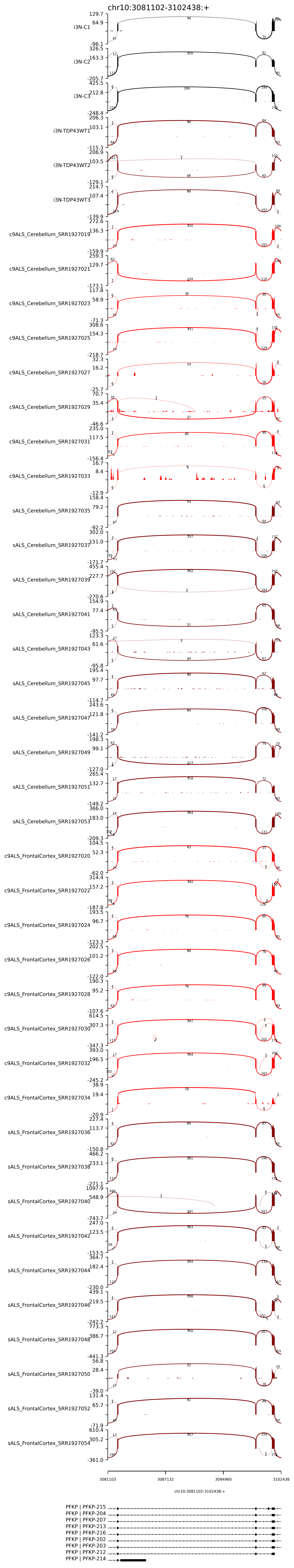
chr10:3081102-3102438:+
i3N-C1
i3N-C2
i3N-C3
i3N-TDP43WT1
i3N-TDP43WT2
i3N-TDP43WT3
c9ALS_Cerebellum_SRR1927019
c9ALS_Cerebellum_SRR1927021
c9ALS_Cerebellum_SRR1927023
c9ALS_Cerebellum_SRR1927025
c9ALS_Cerebellum_SRR1927027
c9ALS_Cerebellum_SRR1927029
c9ALS_Cerebellum_SRR1927031
c9ALS_Cerebellum_SRR1927033
sALS_Cerebellum_SRR1927035
sALS_Cerebellum_SRR1927037
sALS_Cerebellum_SRR1927039
sALS_Cerebellum_SRR1927041
sALS_Cerebellum_SRR1927043
sALS_Cerebellum_SRR1927045
sALS_Cerebellum_SRR1927047
sALS_Cerebellum_SRR1927049
sALS_Cerebellum_SRR1927051
sALS_Cerebellum_SRR1927053
c9ALS_FrontalCortex_SRR1927020
c9ALS_FrontalCortex_SRR1927022
c9ALS_FrontalCortex_SRR1927024
c9ALS_FrontalCortex_SRR1927026
c9ALS_FrontalCortex_SRR1927028
c9ALS_FrontalCortex_SRR1927030
c9ALS_FrontalCortex_SRR1927032
c9ALS_FrontalCortex_SRR1927034
sALS_FrontalCortex_SRR1927036
sALS_FrontalCortex_SRR1927038
sALS_FrontalCortex_SRR1927040
sALS_FrontalCortex_SRR1927042
sALS_FrontalCortex_SRR1927044
sALS_FrontalCortex_SRR1927046
sALS_FrontalCortex_SRR1927048
sALS_FrontalCortex_SRR1927050
sALS_FrontalCortex_SRR1927052
sALS_FrontalCortex_SRR1927054
3081103
3087132
3094960
3102438
chr10:3081102-3102438:+
PFKP | PFKP-215
PFKP | PFKP-204
PFKP | PFKP-207
PFKP | PFKP-213
PFKP | PFKP-216
PFKP | PFKP-202
PFKP | PFKP-203
PFKP | PFKP-212
PFKP | PFKP-214
