# Supplementary material for: Elevated nuclear TDP-43 induces constitutive exon skipping
Source: Mol Neurodegener. 2024 Jun 9;19:45. doi: 10.1186/s13024-024-00732-w (PMC11163724; doi:10.1186/s13024-024-00732-w)
Supplement: Supplementary file 6 — Supplementary Material 6 [file 13024_2024_732_MOESM6_ESM.pdf]

Gene	Coordinates	Splicing Type	Data
BMPR1A	chr10:86921303-86924982	skiptic exon	Carmen-Orozco, et al. 2023
CANX	chr5:179698416-179706991	skiptic exon	Carmen-Orozco, et al. 2023
COQ5	chr12:120516319-120526842	skiptic exon	Carmen-Orozco, et al. 2023
ELP2	chr18:36166598-36171437	skiptic exon	Carmen-Orozco, et al. 2023
HYOU1	chr11:119052044-119052857	skiptic exon	Carmen-Orozco, et al. 2023
MYBBP1A	chr17:4544952-4545777	skiptic exon	Carmen-Orozco, et al. 2023
NUP93	chr16:56838753-56841937	skiptic exon	Carmen-Orozco, et al. 2023
SCN9A	chr2:166272081-166280656	skiptic exon	Carmen-Orozco, et al. 2023
SESN3	chr11:95185056-95191820	skiptic exon	Carmen-Orozco, et al. 2023
SLC35A5	chr3:112562822-112571073	skiptic exon	Carmen-Orozco, et al. 2023
TESK1	chr9:35606765-35607748	skiptic exon	Carmen-Orozco, et al. 2023
VARS2	chr6:30916103-30917282	skiptic exon	Carmen-Orozco, et al. 2023
WSCD1	chr17:6080025-6095711	skiptic exon	Carmen-Orozco, et al. 2023
XPNPEP1	chr10:109883737-109888276	skiptic exon	Carmen-Orozco, et al. 2023
DDI2	chr1:15651731-15662471	skiptic exon	Carmen-Orozco, et al. 2023
PLOD1	chr1:11965458-11967116	skiptic exon	Fratta, et al. 2018
SLC6A6	chr3:14467812-14472314	skiptic exon	Fratta, et al. 2018
ACTL6B	chr7:100649782-100655263	cryptic exon	Irwin, et al. 2024
AGRN	chr1:1044046-1045563	cryptic exon	Irwin, et al. 2025
EPB41L4A	chr5:112265962-112275610	cryptic exon	Irwin, et al. 2026
HDGFL2	chr19:4491492-4494096	cryptic exon	Irwin, et al. 2027
SLC24A3	chr20:19681638-19684461	cryptic exon	Irwin, et al. 2028
STMN2	chr8:79610163-79637892	cryptic exon	Klim, et al. 2019, Melamed et al. 2019
UNC13A	chr19:17641397-17642959	cryptic exon	Rosa Ma, et al. 2022
ATG4B	chr2:241667964-241672277	cryptic exon	Ling, et al. 2015
GPSM2	chr1:108892000-108899964	cryptic exon	Ling, et al. 2015
PFKP	chr10:3081102-3102438	cryptic exon	Ling, et al. 2015

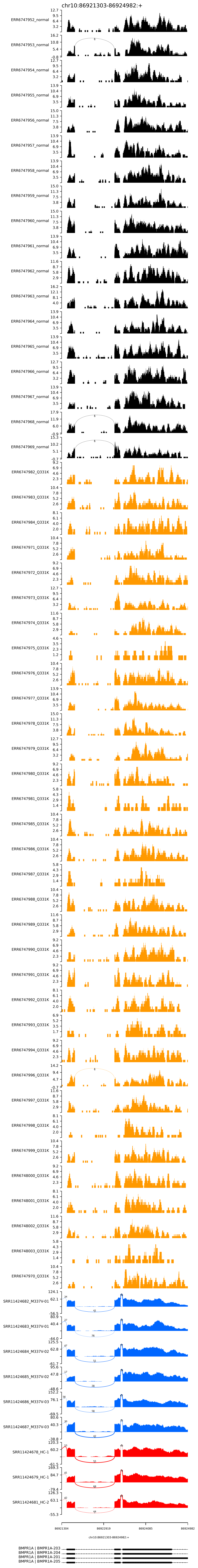

chr10:86921303-86924982:+
ERR6747952_normal
ERR6747953_normal
ERR6747954_normal
ERR6747955_normal
ERR6747956_normal
ERR6747957_normal
ERR6747958_normal
ERR6747959_normal
ERR6747960_normal
ERR6747961_normal
ERR6747962_normal
ERR6747963_normal
ERR6747964_normal
ERR6747965_normal
ERR6747966_normal
ERR6747967_normal
ERR6747968_normal
ERR6747969_normal
ERR6747982_Q331K
ERR6747983_Q331K
ERR6747984_Q331K
ERR6747971_Q331K
ERR6747972_Q331K
ERR6747973_Q331K
ERR6747974_Q331K
ERR6747975_Q331K
ERR6747976_Q331K
ERR6747977_Q331K
ERR6747978_Q331K
ERR6747979_Q331K
ERR6747980_Q331K
ERR6747981_Q331K
ERR6747985_Q331K
ERR6747986_Q331K
ERR6747987_Q331K
ERR6747988_Q331K
ERR6747989_Q331K
ERR6747990_Q331K
ERR6747991_Q331K
ERR6747992_Q331K
ERR6747993_Q331K
ERR6747994_Q331K
ERR6747996_Q331K
ERR6747997_Q331K
ERR6747998_Q331K
ERR6747999_Q331K
ERR6748000_Q331K
ERR6748001_Q331K
ERR6748002_Q331K
ERR6748003_Q331K
ERR6747970_Q331K
SRR11424682_M337V-01
SRR11424683_M337V-01
SRR11424684_M337V-02
SRR11424685_M337V-02
SRR11424686_M337V-03
SRR11424687_M337V-03
SRR11424678_HC-1
SRR11424679_HC-1
SRR11424681_HC-2
86921304
86922919
86924085
86924982
chr10:86921303-86924982:+
BMPR1A | BMPR1A-203
BMPR1A | BMPR1A-204
BMPR1A | BMPR1A-201
BMPR1A | BMPR1A-205

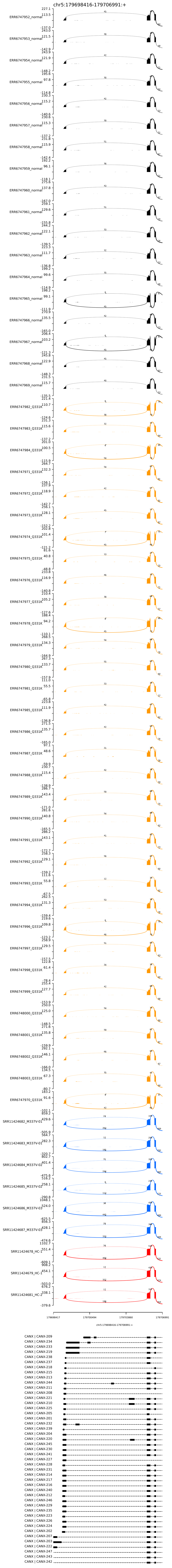

chr5:179698416-179706991:+

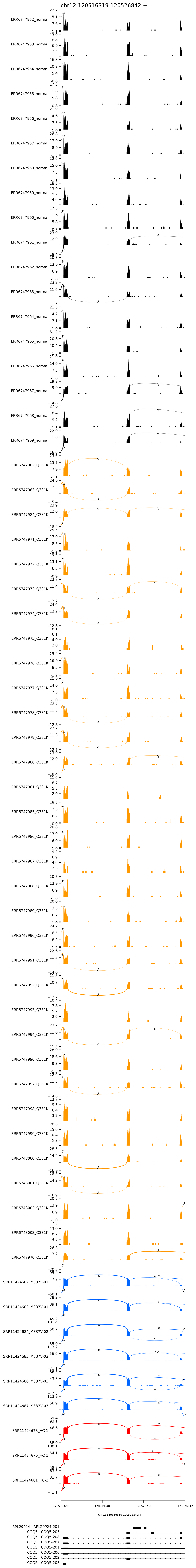
chr12:120516319-120526842:+
ERR6747952_normal
ERR6747953_normal
ERR6747954_normal
ERR6747955_normal
ERR6747956_normal
ERR6747957_normal
ERR6747958_normal
ERR6747959_normal
ERR6747960_normal
ERR6747961_normal
ERR6747962_normal
ERR6747963_normal
ERR6747964_normal
ERR6747965_normal
ERR6747966_normal
ERR6747967_normal
ERR6747968_normal
ERR6747969_normal
ERR6747982_Q331K
ERR6747983_Q331K
ERR6747984_Q331K
ERR6747971_Q331K
ERR6747972_Q331K
ERR6747973_Q331K
ERR6747974_Q331K
ERR6747975_Q331K
ERR6747976_Q331K
ERR6747977_Q331K
ERR6747978_Q331K
ERR6747979_Q331K
ERR6747980_Q331K
ERR6747981_Q331K
ERR6747985_Q331K
ERR6747986_Q331K
ERR6747987_Q331K
ERR6747988_Q331K
ERR6747989_Q331K
ERR6747990_Q331K
ERR6747991_Q331K
ERR6747992_Q331K
ERR6747993_Q331K
ERR6747994_Q331K
ERR6747996_Q331K
ERR6747997_Q331K
ERR6747998_Q331K
ERR6747999_Q331K
ERR6748000_Q331K
ERR6748001_Q331K
ERR6748002_Q331K
ERR6748003_Q331K
ERR6747970_Q331K
SRR11424682_M337V-01
SRR11424683_M337V-01
SRR11424684_M337V-02
SRR11424685_M337V-02
SRR11424686_M337V-03
SRR11424687_M337V-03
SRR11424678_HC-1
SRR11424679_HC-1
SRR11424681_HC-2
120516320
120519948
120523288
120526842
chr12:120516319-120526842:+
RPL29P24 | RPL29P24-201
COQ5 | COQ5-205
COQ5 | COQ5-208
COQ5 | COQ5-207
COQ5 | COQ5-201
COQ5 | COQ5-206
COQ5 | COQ5-202
COQ5 | COQ5-203

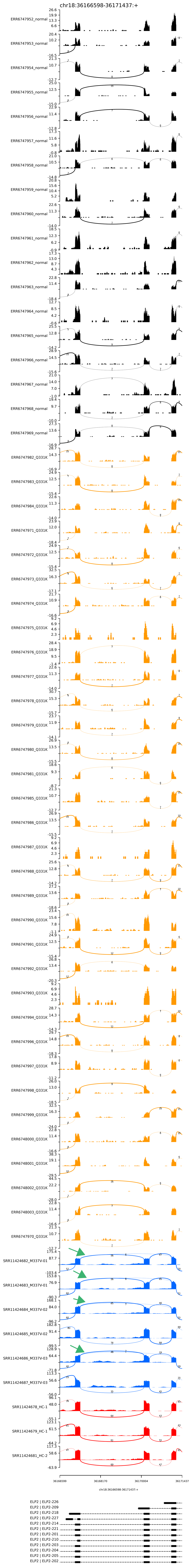
chr18:36166598-36171437:+
ERR6747952_normal
ERR6747953_normal
ERR6747954_normal
ERR6747955_normal
ERR6747956_normal
ERR6747957_normal
ERR6747958_normal
ERR6747959_normal
ERR6747960_normal
ERR6747961_normal
ERR6747962_normal
ERR6747963_normal
ERR6747964_normal
ERR6747965_normal
ERR6747966_normal
ERR6747967_normal
ERR6747968_normal
ERR6747969_normal
ERR6747982_Q331K
ERR6747983_Q331K
ERR6747984_Q331K
ERR6747971_Q331K
ERR6747972_Q331K
ERR6747973_Q331K
ERR6747974_Q331K
ERR6747975_Q331K
ERR6747976_Q331K
ERR6747977_Q331K
ERR6747978_Q331K
ERR6747979_Q331K
ERR6747980_Q331K
ERR6747981_Q331K
ERR6747985_Q331K
ERR6747986_Q331K
ERR6747987_Q331K
ERR6747988_Q331K
ERR6747989_Q331K
ERR6747990_Q331K
ERR6747991_Q331K
ERR6747992_Q331K
ERR6747993_Q331K
ERR6747994_Q331K
ERR6747996_Q331K
ERR6747997_Q331K
ERR6747998_Q331K
ERR6747999_Q331K
ERR6748000_Q331K
ERR6748001_Q331K
ERR6748002_Q331K
ERR6748003_Q331K
ERR6747970_Q331K
SRR11424682_M337V-01
SRR11424683_M337V-01
SRR11424684_M337V-02
SRR11424685_M337V-02
SRR11424686_M337V-03
SRR11424687_M337V-03
SRR11424678_HC-1
SRR11424679_HC-1
SRR11424681_HC-2
36166599
36168170
36170004
36171437
chr18:36166598-36171437:+
ELP2 | ELP2-226
ELP2 | ELP2-209
ELP2 | ELP2-218
ELP2 | ELP2-227
ELP2 | ELP2-214
ELP2 | ELP2-221
ELP2 | ELP2-201
ELP2 | ELP2-210
ELP2 | ELP2-203
ELP2 | ELP2-204
ELP2 | ELP2-205
ELP2 | ELP2-202

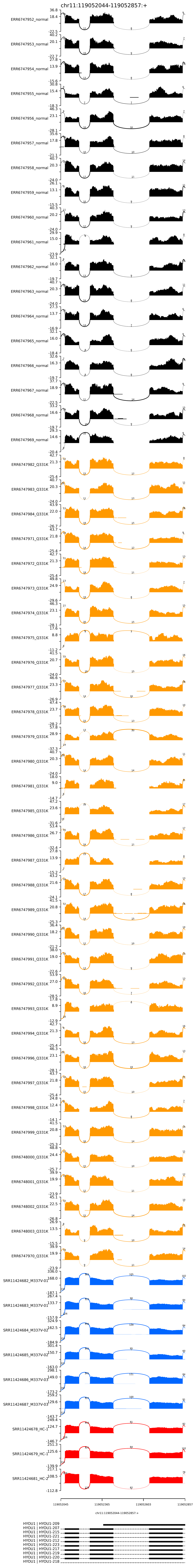
chr11:119052044-119052857:+
ERR6747952_normal
ERR6747953_normal
ERR6747954_normal
ERR6747955_normal
ERR6747956_normal
ERR6747957_normal
ERR6747958_normal
ERR6747959_normal
ERR6747960_normal
ERR6747961_normal
ERR6747962_normal
ERR6747963_normal
ERR6747964_normal
ERR6747965_normal
ERR6747966_normal
ERR6747967_normal
ERR6747968_normal
ERR6747969_normal
ERR6747982_Q331K
ERR6747983_Q331K
ERR6747984_Q331K
ERR6747971_Q331K
ERR6747972_Q331K
ERR6747973_Q331K
ERR6747974_Q331K
ERR6747975_Q331K
ERR6747976_Q331K
ERR6747977_Q331K
ERR6747978_Q331K
ERR6747979_Q331K
ERR6747980_Q331K
ERR6747981_Q331K
ERR6747985_Q331K
ERR6747986_Q331K
ERR6747987_Q331K
ERR6747988_Q331K
ERR6747989_Q331K
ERR6747990_Q331K
ERR6747991_Q331K
ERR6747992_Q331K
ERR6747993_Q331K
ERR6747994_Q331K
ERR6747996_Q331K
ERR6747997_Q331K
ERR6747998_Q331K
ERR6747999_Q331K
ERR6748000_Q331K
ERR6748001_Q331K
ERR6748002_Q331K
ERR6748003_Q331K
ERR6747970_Q331K
SRR11424682_M337V-01
SRR11424683_M337V-01
SRR11424684_M337V-02
SRR11424685_M337V-02
SRR11424686_M337V-03
SRR11424687_M337V-03
SRR11424678_HC-1
SRR11424679_HC-1
SRR11424681_HC-2
119052045
119052365
119052603
119052857
chr11:119052044-119052857:+
HYOU1 | HYOU1-209
HYOU1 | HYOU1-207
HYOU1 | HYOU1-215
HYOU1 | HYOU1-221
HYOU1 | HYOU1-212
HYOU1 | HYOU1-223
HYOU1 | HYOU1-217
HYOU1 | HYOU1-219
HYOU1 | HYOU1-218

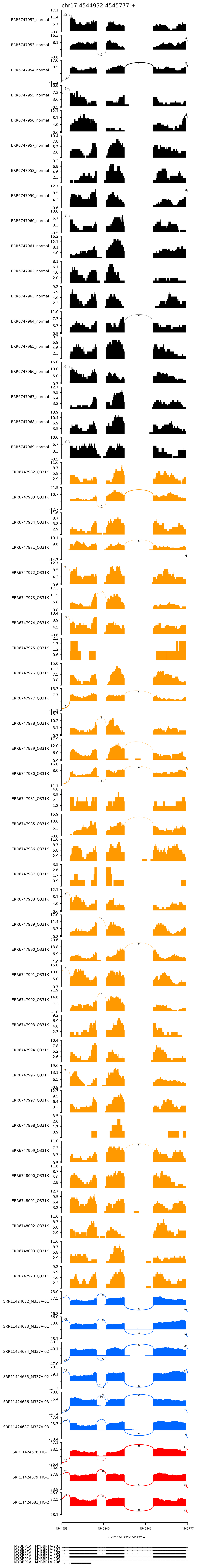
chr17:4544952-4545777:+
ERR6747952_normal
ERR6747953_normal
ERR6747954_normal
ERR6747955_normal
ERR6747956_normal
ERR6747957_normal
ERR6747958_normal
ERR6747959_normal
ERR6747960_normal
ERR6747961_normal
ERR6747962_normal
ERR6747963_normal
ERR6747964_normal
ERR6747965_normal
ERR6747966_normal
ERR6747967_normal
ERR6747968_normal
ERR6747969_normal
ERR6747982_Q331K
ERR6747983_Q331K
ERR6747984_Q331K
ERR6747971_Q331K
ERR6747972_Q331K
ERR6747973_Q331K
ERR6747974_Q331K
ERR6747975_Q331K
ERR6747976_Q331K
ERR6747977_Q331K
ERR6747978_Q331K
ERR6747979_Q331K
ERR6747980_Q331K
ERR6747981_Q331K
ERR6747985_Q331K
ERR6747986_Q331K
ERR6747987_Q331K
ERR6747988_Q331K
ERR6747989_Q331K
ERR6747990_Q331K
ERR6747991_Q331K
ERR6747992_Q331K
ERR6747993_Q331K
ERR6747994_Q331K
ERR6747996_Q331K
ERR6747997_Q331K
ERR6747998_Q331K
ERR6747999_Q331K
ERR6748000_Q331K
ERR6748001_Q331K
ERR6748002_Q331K
ERR6748003_Q331K
ERR6747970_Q331K
SRR11424682_M337V-01
SRR11424683_M337V-01
SRR11424684_M337V-02
SRR11424685_M337V-02
SRR11424686_M337V-03
SRR11424687_M337V-03
SRR11424678_HC-1
SRR11424679_HC-1
SRR11424681_HC-2
4544953
4545240
4545541
4545777
chr17:4544952-4545777:+

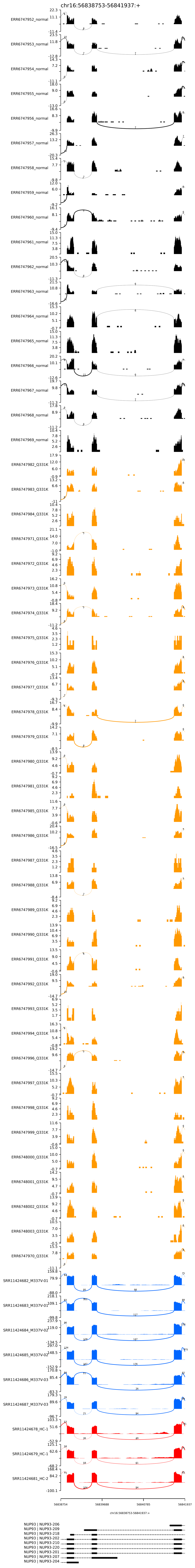
chr16:56838753-56841937:+
ERR6747952_normal
ERR6747953_normal
ERR6747954_normal
ERR6747955_normal
ERR6747956_normal
ERR6747957_normal
ERR6747958_normal
ERR6747959_normal
ERR6747960_normal
ERR6747961_normal
ERR6747962_normal
ERR6747963_normal
ERR6747964_normal
ERR6747965_normal
ERR6747966_normal
ERR6747967_normal
ERR6747968_normal
ERR6747969_normal
ERR6747982_Q331K
ERR6747983_Q331K
ERR6747984_Q331K
ERR6747971_Q331K
ERR6747972_Q331K
ERR6747973_Q331K
ERR6747974_Q331K
ERR6747975_Q331K
ERR6747976_Q331K
ERR6747977_Q331K
ERR6747978_Q331K
ERR6747979_Q331K
ERR6747980_Q331K
ERR6747981_Q331K
ERR6747985_Q331K
ERR6747986_Q331K
ERR6747987_Q331K
ERR6747988_Q331K
ERR6747989_Q331K
ERR6747990_Q331K
ERR6747991_Q331K
ERR6747992_Q331K
ERR6747993_Q331K
ERR6747994_Q331K
ERR6747996_Q331K
ERR6747997_Q331K
ERR6747998_Q331K
ERR6747999_Q331K
ERR6748000_Q331K
ERR6748001_Q331K
ERR6748002_Q331K
ERR6748003_Q331K
ERR6747970_Q331K
SRR11424682_M337V-01
SRR11424683_M337V-01
SRR11424684_M337V-02
SRR11424685_M337V-02
SRR11424686_M337V-03
SRR11424687_M337V-03
SRR11424678_HC-1
SRR11424679_HC-1
SRR11424681_HC-2
56838754
56839688
56840785
56841937
chr16:56838753-56841937:+
NUP93 | NUP93-206
NUP93 | NUP93-209
NUP93 | NUP93-218
NUP93 | NUP93-202
NUP93 | NUP93-210
NUP93 | NUP93-220
NUP93 | NUP93-203
NUP93 | NUP93-201
NUP93 | NUP93-211
NUP93 | NUP93-204

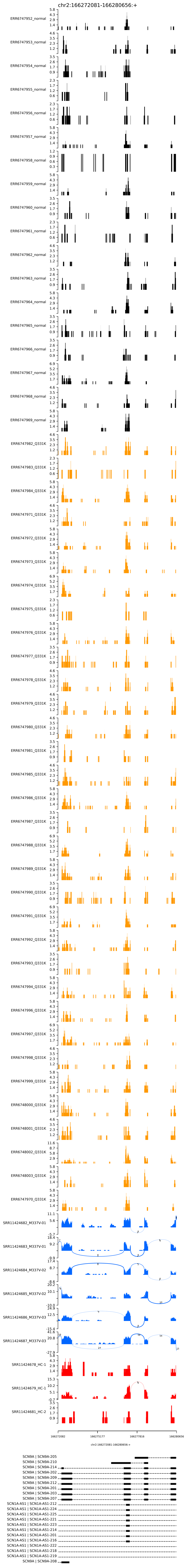

chr2:166272081-166280656:+
ERR6747952_normal
ERR6747953_normal
ERR6747954_normal
ERR6747955_normal
ERR6747956_normal
ERR6747957_normal
ERR6747958_normal
ERR6747959_normal
ERR6747960_normal
ERR6747961_normal
ERR6747962_normal
ERR6747963_normal
ERR6747964_normal
ERR6747965_normal
ERR6747966_normal
ERR6747967_normal
ERR6747968_normal
ERR6747969_normal
ERR6747982_Q331K
ERR6747983_Q331K
ERR6747984_Q331K
ERR6747971_Q331K
ERR6747972_Q331K
ERR6747973_Q331K
ERR6747974_Q331K
ERR6747975_Q331K
ERR6747976_Q331K
ERR6747977_Q331K
ERR6747978_Q331K
ERR6747979_Q331K
ERR6747980_Q331K
ERR6747981_Q331K
ERR6747985_Q331K
ERR6747986_Q331K
ERR6747987_Q331K
ERR6747988_Q331K
ERR6747989_Q331K
ERR6747990_Q331K
ERR6747991_Q331K
ERR6747992_Q331K
ERR6747993_Q331K
ERR6747994_Q331K
ERR6747996_Q331K
ERR6747997_Q331K
ERR6747998_Q331K
ERR6747999_Q331K
ERR6748000_Q331K
ERR6748001_Q331K
ERR6748002_Q331K
ERR6748003_Q331K
ERR6747970_Q331K
SRR11424682_M337V-01
SRR11424683_M337V-01
SRR11424684_M337V-02
SRR11424685_M337V-02
SRR11424686_M337V-03
SRR11424687_M337V-03
SRR11424678_HC-1
SRR11424679_HC-1
SRR11424681_HC-2
166272082
166275177
166277816
166280656
chr2:166272081-166280656:+
SCN9A | SCN9A-205
SCN9A | SCN9A-210
SCN9A | SCN9A-214
SCN9A | SCN9A-202
SCN9A | SCN9A-209
SCN9A | SCN9A-212
SCN9A | SCN9A-201
SCN9A | SCN9A-203
SCN9A | SCN9A-207
SCN1A-AS1 | SCN1A-AS1-212
SCN1A-AS1 | SCN1A-AS1-224
SCN1A-AS1 | SCN1A-AS1-225
SCN1A-AS1 | SCN1A-AS1-221
SCN1A-AS1 | SCN1A-AS1-220
SCN1A-AS1 | SCN1A-AS1-214
SCN1A-AS1 | SCN1A-AS1-201
SCN1A-AS1 | SCN1A-AS1-216
SCN1A-AS1 | SCN1A-AS1-222
SCN1A-AS1 | SCN1A-AS1-218
SCN1A-AS1 | SCN1A-AS1-219
SCN9A | SCN9A-208

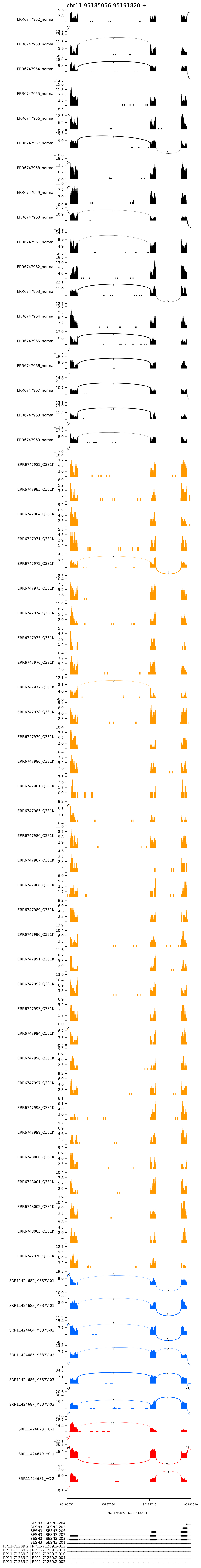
chr11:95185056-95191820:+
ERR6747952_normal
ERR6747953_normal
ERR6747954_normal
ERR6747955_normal
ERR6747956_normal
ERR6747957_normal
ERR6747958_normal
ERR6747959_normal
ERR6747960_normal
ERR6747961_normal
ERR6747962_normal
ERR6747963_normal
ERR6747964_normal
ERR6747965_normal
ERR6747966_normal
ERR6747967_normal
ERR6747968_normal
ERR6747969_normal
ERR6747982_Q331K
ERR6747983_Q331K
ERR6747984_Q331K
ERR6747971_Q331K
ERR6747972_Q331K
ERR6747973_Q331K
ERR6747974_Q331K
ERR6747975_Q331K
ERR6747976_Q331K
ERR6747977_Q331K
ERR6747978_Q331K
ERR6747979_Q331K
ERR6747980_Q331K
ERR6747981_Q331K
ERR6747985_Q331K
ERR6747986_Q331K
ERR6747987_Q331K
ERR6747988_Q331K
ERR6747989_Q331K
ERR6747990_Q331K
ERR6747991_Q331K
ERR6747992_Q331K
ERR6747993_Q331K
ERR6747994_Q331K
ERR6747996_Q331K
ERR6747997_Q331K
ERR6747998_Q331K
ERR6747999_Q331K
ERR6748000_Q331K
ERR6748001_Q331K
ERR6748002_Q331K
ERR6748003_Q331K
ERR6747970_Q331K
SRR11424682_M337V-01
SRR11424683_M337V-01
SRR11424684_M337V-02
SRR11424685_M337V-02
SRR11424686_M337V-03
SRR11424687_M337V-03
SRR11424678_HC-1
SRR11424679_HC-1
SRR11424681_HC-2
95185057
95187280
95189740
95191820
chr11:95185056-95191820:+

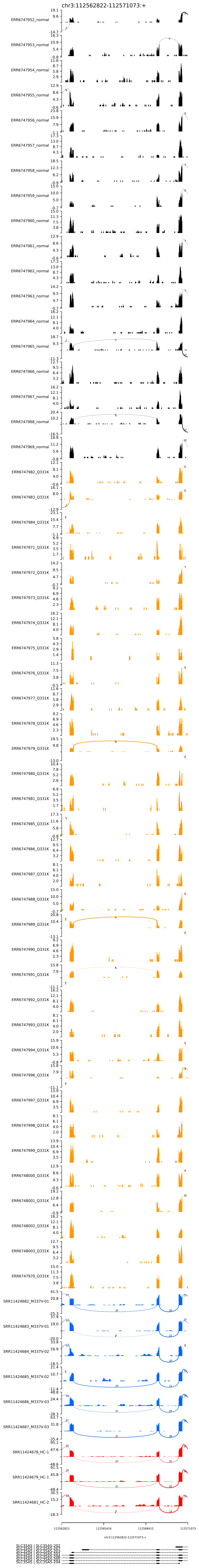
chr3:112562822-112571073:+
ERR6747952_normal
ERR6747953_normal
ERR6747954_normal
ERR6747955_normal
ERR6747956_normal
ERR6747957_normal
ERR6747958_normal
ERR6747959_normal
ERR6747960_normal
ERR6747961_normal
ERR6747962_normal
ERR6747963_normal
ERR6747964_normal
ERR6747965_normal
ERR6747966_normal
ERR6747967_normal
ERR6747968_normal
ERR6747969_normal
ERR6747982_Q331K
ERR6747983_Q331K
ERR6747984_Q331K
ERR6747971_Q331K
ERR6747972_Q331K
ERR6747973_Q331K
ERR6747974_Q331K
ERR6747975_Q331K
ERR6747976_Q331K
ERR6747977_Q331K
ERR6747978_Q331K
ERR6747979_Q331K
ERR6747980_Q331K
ERR6747981_Q331K
ERR6747985_Q331K
ERR6747986_Q331K
ERR6747987_Q331K
ERR6747988_Q331K
ERR6747989_Q331K
ERR6747990_Q331K
ERR6747991_Q331K
ERR6747992_Q331K
ERR6747993_Q331K
ERR6747994_Q331K
ERR6747996_Q331K
ERR6747997_Q331K
ERR6747998_Q331K
ERR6747999_Q331K
ERR6748000_Q331K
ERR6748001_Q331K
ERR6748002_Q331K
ERR6748003_Q331K
ERR6747970_Q331K
SRR11424682_M337V-01
SRR11424683_M337V-01
SRR11424684_M337V-02
SRR11424685_M337V-02
SRR11424686_M337V-03
SRR11424687_M337V-03
SRR11424678_HC-1
SRR11424679_HC-1
SRR11424680_HC-2
SRR11424681_HC-2
112562823
112565424
112568412
112571073
chr3:112562822-112571073:+

chr9:35606765-35607748:+

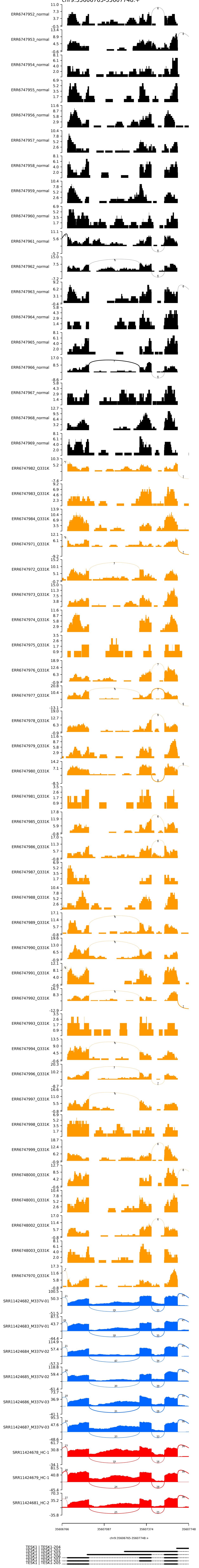

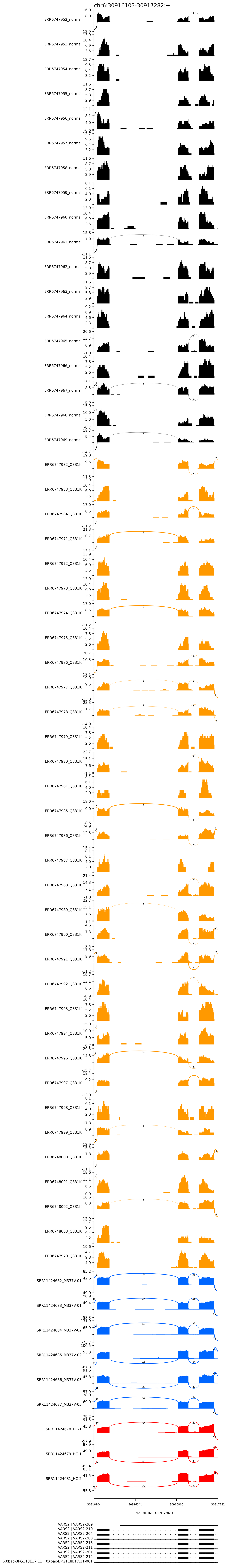
chr6:30916103-30917282:+
ERR6747952_normal
ERR6747953_normal
ERR6747954_normal
ERR6747955_normal
ERR6747956_normal
ERR6747957_normal
ERR6747958_normal
ERR6747959_normal
ERR6747960_normal
ERR6747961_normal
ERR6747962_normal
ERR6747963_normal
ERR6747964_normal
ERR6747965_normal
ERR6747966_normal
ERR6747967_normal
ERR6747968_normal
ERR6747969_normal
ERR6747982_Q331K
ERR6747983_Q331K
ERR6747984_Q331K
ERR6747971_Q331K
ERR6747972_Q331K
ERR6747973_Q331K
ERR6747974_Q331K
ERR6747975_Q331K
ERR6747976_Q331K
ERR6747977_Q331K
ERR6747978_Q331K
ERR6747979_Q331K
ERR6747980_Q331K
ERR6747981_Q331K
ERR6747985_Q331K
ERR6747986_Q331K
ERR6747987_Q331K
ERR6747988_Q331K
ERR6747989_Q331K
ERR6747990_Q331K
ERR6747991_Q331K
ERR6747992_Q331K
ERR6747993_Q331K
ERR6747994_Q331K
ERR6747996_Q331K
ERR6747997_Q331K
ERR6747998_Q331K
ERR6747999_Q331K
ERR6748000_Q331K
ERR6748001_Q331K
ERR6748002_Q331K
ERR6748003_Q331K
ERR6747970_Q331K
SRR11424682_M337V-01
SRR11424683_M337V-01
SRR11424684_M337V-02
SRR11424685_M337V-02
SRR11424686_M337V-03
SRR11424687_M337V-03
SRR11424678_HC-1
SRR11424679_HC-1
SRR11424681_HC-2
30916104
30916541
30916866
30917282
chr6:30916103-30917282:+
VARS2 | VARS2-209
VARS2 | VARS2-210
VARS2 | VARS2-204
VARS2 | VARS2-203
VARS2 | VARS2-213
VARS2 | VARS2-211
VARS2 | VARS2-201
VARS2 | VARS2-212
XXbac-BPG118E17.11 | XXbac-BPG118E17.11-001

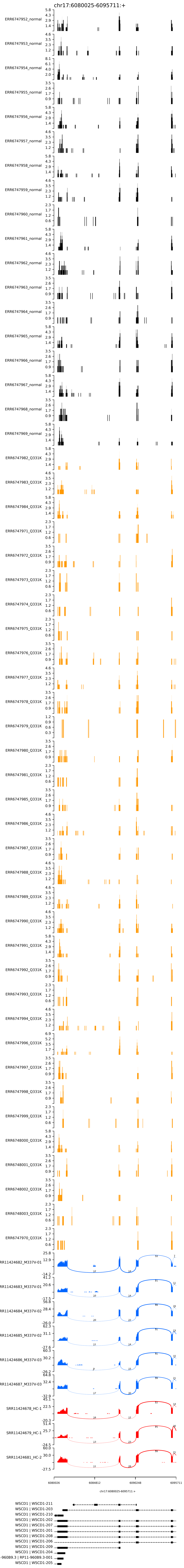
chr17:6080025-6095711:+
ERR6747952_normal
ERR6747953_normal
ERR6747954_normal
ERR6747955_normal
ERR6747956_normal
ERR6747957_normal
ERR6747958_normal
ERR6747959_normal
ERR6747960_normal
ERR6747961_normal
ERR6747962_normal
ERR6747963_normal
ERR6747964_normal
ERR6747965_normal
ERR6747966_normal
ERR6747967_normal
ERR6747968_normal
ERR6747969_normal
ERR6747982_Q331K
ERR6747983_Q331K
ERR6747984_Q331K
ERR6747971_Q331K
ERR6747972_Q331K
ERR6747973_Q331K
ERR6747974_Q331K
ERR6747975_Q331K
ERR6747976_Q331K
ERR6747977_Q331K
ERR6747978_Q331K
ERR6747979_Q331K
ERR6747980_Q331K
ERR6747981_Q331K
ERR6747985_Q331K
ERR6747986_Q331K
ERR6747987_Q331K
ERR6747988_Q331K
ERR6747989_Q331K
ERR6747990_Q331K
ERR6747991_Q331K
ERR6747992_Q331K
ERR6747993_Q331K
ERR6747994_Q331K
ERR6747996_Q331K
ERR6747997_Q331K
ERR6747998_Q331K
ERR6747999_Q331K
ERR6748000_Q331K
ERR6748001_Q331K
ERR6748002_Q331K
ERR6748003_Q331K
ERR6747970_Q331K
SRR11424682_M337V-01
SRR11424683_M337V-01
SRR11424684_M337V-02
SRR11424685_M337V-02
SRR11424686_M337V-03
SRR11424687_M337V-03
SRR11424678_HC-1
SRR11424679_HC-1
SRR11424681_HC-2
6080026
6084812
6090268
6095711
chr17:6080025-6095711:+
WSCD1 | WSCD1-211
WSCD1 | WSCD1-203
WSCD1 | WSCD1-210
WSCD1 | WSCD1-202
WSCD1 | WSCD1-207
WSCD1 | WSCD1-201
WSCD1 | WSCD1-208
WSCD1 | WSCD1-206
WSCD1 | WSCD1-209
WSCD1 | WSCD1-204
RP11-960B9.3 | RP11-960B9.3-001
WSCD1 | WSCD1-205

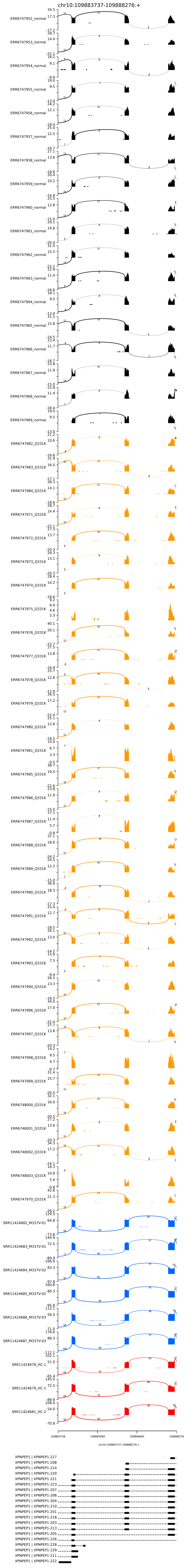

chr10:109883737-109888276:+
ERR6747952_normal
ERR6747953_normal
ERR6747954_normal
ERR6747955_normal
ERR6747956_normal
ERR6747957_normal
ERR6747958_normal
ERR6747959_normal
ERR6747960_normal
ERR6747961_normal
ERR6747962_normal
ERR6747963_normal
ERR6747964_normal
ERR6747965_normal
ERR6747966_normal
ERR6747967_normal
ERR6747968_normal
ERR6747969_normal
ERR6747982_Q331K
ERR6747983_Q331K
ERR6747984_Q331K
ERR6747971_Q331K
ERR6747972_Q331K
ERR6747973_Q331K
ERR6747974_Q331K
ERR6747975_Q331K
ERR6747976_Q331K
ERR6747977_Q331K
ERR6747978_Q331K
ERR6747979_Q331K
ERR6747980_Q331K
ERR6747981_Q331K
ERR6747985_Q331K
ERR6747986_Q331K
ERR6747987_Q331K
ERR6747988_Q331K
ERR6747989_Q331K
ERR6747990_Q331K
ERR6747991_Q331K
ERR6747992_Q331K
ERR6747993_Q331K
ERR6747994_Q331K
ERR6747996_Q331K
ERR6747997_Q331K
ERR6747998_Q331K
ERR6747999_Q331K
ERR6748000_Q331K
ERR6748001_Q331K
ERR6748002_Q331K
ERR6748003_Q331K
ERR6747970_Q331K
SRR11424682_M337V-02
SRR11424683_M337V-01
SRR11424684_M337V-02
SRR11424685_M337V-02
SRR11424686_M337V-03
SRR11424687_M337V-03
SRR11424678_HC-1
SRR11424679_HC-1
SRR11424681_HC-2
109883738
109885069
109886695
109888276
chr10:109883737-109888276:+
XPNPEP1 | XPNPEP1-217
XPNPEP1 | XPNPEP1-208
XPNPEP1 | XPNPEP1-214
XPNPEP1 | XPNPEP1-220
XPNPEP1 | XPNPEP1-221
XPNPEP1 | XPNPEP1-223
XPNPEP1 | XPNPEP1-207
XPNPEP1 | XPNPEP1-206
XPNPEP1 | XPNPEP1-204
XPNPEP1 | XPNPEP1-210
XPNPEP1 | XPNPEP1-201
XPNPEP1 | XPNPEP1-218
XPNPEP1 | XPNPEP1-203
XPNPEP1 | XPNPEP1-213
XPNPEP1 | XPNPEP1-205
XPNPEP1 | XPNPEP1-226
XPNPEP1 | XPNPEP1-228
XPNPEP1 | XPNPEP1-229
XPNPEP1 | XPNPEP1-211
XPNPEP1 | XPNPEP1-222

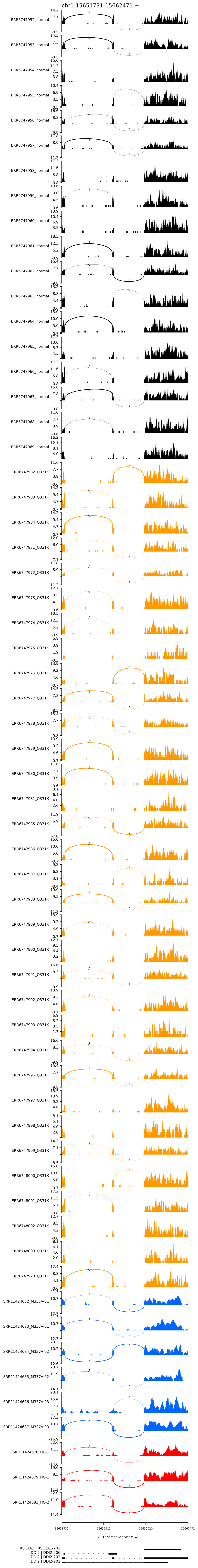
chr1:15651731-15662471:+
ERR6747952_normal
ERR6747953_normal
ERR6747954_normal
ERR6747955_normal
ERR6747956_normal
ERR6747957_normal
ERR6747958_normal
ERR6747959_normal
ERR6747960_normal
ERR6747961_normal
ERR6747962_normal
ERR6747963_normal
ERR6747964_normal
ERR6747965_normal
ERR6747966_normal
ERR6747967_normal
ERR6747968_normal
ERR6747969_normal
ERR6747982_Q331K
ERR6747983_Q331K
ERR6747984_Q331K
ERR6747971_Q331K
ERR6747972_Q331K
ERR6747973_Q331K
ERR6747974_Q331K
ERR6747975_Q331K
ERR6747976_Q331K
ERR6747977_Q331K
ERR6747978_Q331K
ERR6747979_Q331K
ERR6747980_Q331K
ERR6747981_Q331K
ERR6747985_Q331K
ERR6747986_Q331K
ERR6747987_Q331K
ERR6747988_Q331K
ERR6747989_Q331K
ERR6747990_Q331K
ERR6747991_Q331K
ERR6747992_Q331K
ERR6747993_Q331K
ERR6747994_Q331K
ERR6747996_Q331K
ERR6747997_Q331K
ERR6747998_Q331K
ERR6747999_Q331K
ERR6748000_Q331K
ERR6748001_Q331K
ERR6748002_Q331K
ERR6748003_Q331K
ERR6747970_Q331K
SRR11424682_M337V-01
SRR11424683_M337V-01
SRR11424684_M337V-02
SRR11424685_M337V-02
SRR11424686_M337V-03
SRR11424687_M337V-03
SRR11424678_HC-1
SRR11424679_HC-1
SRR11424681_HC-2
15651732
15655915
15659920
15662471
chr1:15651731-15662471:+
RSC1A1 | RSC1A1-201
DDI2 | DDI2-206
DDI2 | DDI2-202
DDI2 | DDI2-201

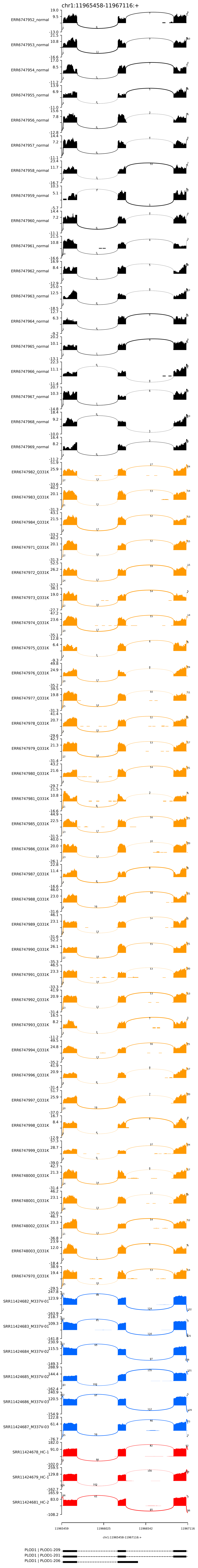
chr1:11965458-11967116:+
ERR6747952_normal
ERR6747953_normal
ERR6747954_normal
ERR6747955_normal
ERR6747956_normal
ERR6747957_normal
ERR6747958_normal
ERR6747959_normal
ERR6747960_normal
ERR6747961_normal
ERR6747962_normal
ERR6747963_normal
ERR6747964_normal
ERR6747965_normal
ERR6747966_normal
ERR6747967_normal
ERR6747968_normal
ERR6747969_normal
ERR6747982_Q331K
ERR6747983_Q331K
ERR6747984_Q331K
ERR6747971_Q331K
ERR6747972_Q331K
ERR6747973_Q331K
ERR6747974_Q331K
ERR6747975_Q331K
ERR6747976_Q331K
ERR6747977_Q331K
ERR6747978_Q331K
ERR6747979_Q331K
ERR6747980_Q331K
ERR6747981_Q331K
ERR6747985_Q331K
ERR6747986_Q331K
ERR6747987_Q331K
ERR6747988_Q331K
ERR6747989_Q331K
ERR6747990_Q331K
ERR6747991_Q331K
ERR6747992_Q331K
ERR6747993_Q331K
ERR6747994_Q331K
ERR6747996_Q331K
ERR6747997_Q331K
ERR6747998_Q331K
ERR6747999_Q331K
ERR6748000_Q331K
ERR6748001_Q331K
ERR6748002_Q331K
ERR6748003_Q331K
ERR6747970_Q331K
SRR11424682_M337V-01
SRR11424683_M337V-01
SRR11424684_M337V-02
SRR11424685_M337V-02
SRR11424686_M337V-03
SRR11424687_M337V-03
SRR11424678_HC-1
SRR11424679_HC-1
SRR11424681_HC-2
11965459
11966025
11966542
11967116
chr1:11965458-11967116:+
PLOD1 | PLOD1-209
PLOD1 | PLOD1-201
PLOD1 | PLOD1-206

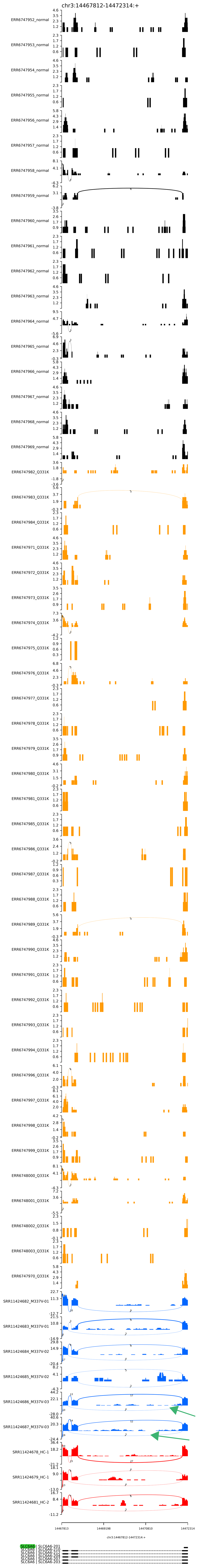
chr3:14467812-14472314:+
ERR6747952_normal
ERR6747953_normal
ERR6747954_normal
ERR6747955_normal
ERR6747956_normal
ERR6747957_normal
ERR6747958_normal
ERR6747959_normal
ERR6747960_normal
ERR6747961_normal
ERR6747962_normal
ERR6747963_normal
ERR6747964_normal
ERR6747965_normal
ERR6747966_normal
ERR6747967_normal
ERR6747968_normal
ERR6747969_normal
ERR6747982_Q331K
ERR6747983_Q331K
ERR6747984_Q331K
ERR6747971_Q331K
ERR6747972_Q331K
ERR6747973_Q331K
ERR6747974_Q331K
ERR6747975_Q331K
ERR6747976_Q331K
ERR6747977_Q331K
ERR6747978_Q331K
ERR6747979_Q331K
ERR6747980_Q331K
ERR6747981_Q331K
ERR6747985_Q331K
ERR6747986_Q331K
ERR6747987_Q331K
ERR6747988_Q331K
ERR6747989_Q331K
ERR6747990_Q331K
ERR6747991_Q331K
ERR6747992_Q331K
ERR6747993_Q331K
ERR6747994_Q331K
ERR6747996_Q331K
ERR6747997_Q331K
ERR6747998_Q331K
ERR6747999_Q331K
ERR6748000_Q331K
ERR6748001_Q331K
ERR6748002_Q331K
ERR6748003_Q331K
ERR6747970_Q331K
SRR11424682_M337V-01
SRR11424683_M337V-01
SRR11424684_M337V-02
SRR11424685_M337V-02
SRR11424686_M337V-03
SRR11424687_M337V-03
SRR11424678_HC-1
SRR11424679_HC-1
SRR11424681_HC-2
14467813
14469198
14470810
14472314
chr3:14467812-14472314:+
SLC6A6 | SLC6A6-201
SLC6A6 | SLC6A6-210
SLC6A6 | SLC6A6-212
SLC6A6 | SLC6A6-204
SLC6A6 | SLC6A6-207
SLC6A6 | SLC6A6-203

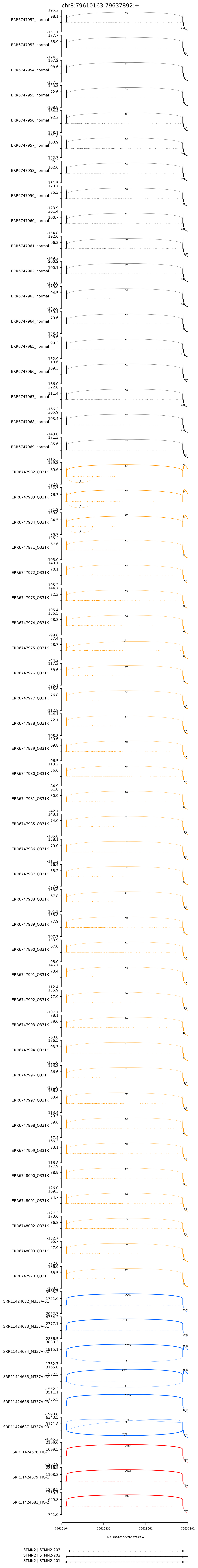
chr8:79610163-79637892:+
ERR6747952_normal
ERR6747953_normal
ERR6747954_normal
ERR6747955_normal
ERR6747956_normal
ERR6747957_normal
ERR6747958_normal
ERR6747959_normal
ERR6747960_normal
ERR6747961_normal
ERR6747962_normal
ERR6747963_normal
ERR6747964_normal
ERR6747965_normal
ERR6747966_normal
ERR6747967_normal
ERR6747968_normal
ERR6747969_normal
ERR6747982_Q331K
ERR6747983_Q331K
ERR6747984_Q331K
ERR6747971_Q331K
ERR6747972_Q331K
ERR6747973_Q331K
ERR6747974_Q331K
ERR6747975_Q331K
ERR6747976_Q331K
ERR6747977_Q331K
ERR6747978_Q331K
ERR6747979_Q331K
ERR6747980_Q331K
ERR6747981_Q331K
ERR6747985_Q331K
ERR6747986_Q331K
ERR6747987_Q331K
ERR6747988_Q331K
ERR6747989_Q331K
ERR6747990_Q331K
ERR6747991_Q331K
ERR6747992_Q331K
ERR6747993_Q331K
ERR6747994_Q331K
ERR6747996_Q331K
ERR6747997_Q331K
ERR6747998_Q331K
ERR6747999_Q331K
ERR6748000_Q331K
ERR6748001_Q331K
ERR6748002_Q331K
ERR6748003_Q331K
ERR6747970_Q331K
SRR11424682_M337V-01
SRR11424683_M337V-01
SRR11424684_M337V-02
SRR11424685_M337V-02
SRR11424686_M337V-03
SRR11424687_M337V-03
SRR11424678_HC-1
SRR11424679_HC-1
SRR11424681_HC-2
79610164
79619335
79628661
79637892
chr8:79610163-79637892:+
STMN2 | STMN2-203
STMN2 | STMN2-202
STMN2 | STMN2-201

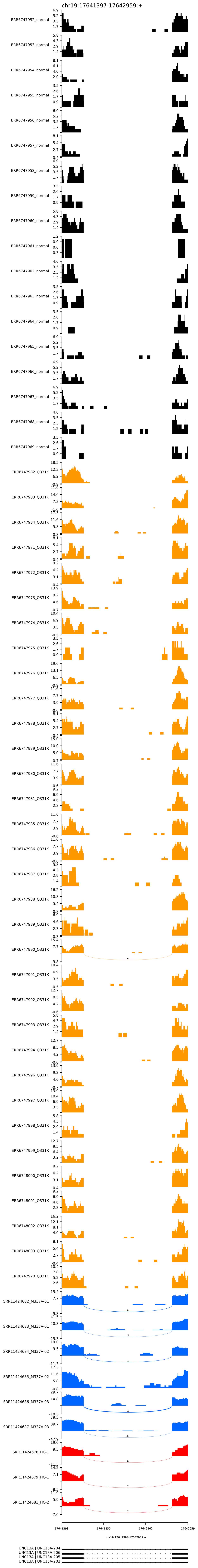

chr19:17641397-17642959:+
ERR6747952_normal
ERR6747953_normal
ERR6747954_normal
ERR6747955_normal
ERR6747956_normal
ERR6747957_normal
ERR6747958_normal
ERR6747959_normal
ERR6747960_normal
ERR6747961_normal
ERR6747962_normal
ERR6747963_normal
ERR6747964_normal
ERR6747965_normal
ERR6747966_normal
ERR6747967_normal
ERR6747968_normal
ERR6747969_normal
ERR6747982_Q331K
ERR6747983_Q331K
ERR6747984_Q331K
ERR6747971_Q331K
ERR6747972_Q331K
ERR6747973_Q331K
ERR6747974_Q331K
ERR6747975_Q331K
ERR6747976_Q331K
ERR6747977_Q331K
ERR6747978_Q331K
ERR6747979_Q331K
ERR6747980_Q331K
ERR6747981_Q331K
ERR6747985_Q331K
ERR6747986_Q331K
ERR6747987_Q331K
ERR6747988_Q331K
ERR6747989_Q331K
ERR6747990_Q331K
ERR6747991_Q331K
ERR6747992_Q331K
ERR6747993_Q331K
ERR6747994_Q331K
ERR6747996_Q331K
ERR6747997_Q331K
ERR6747998_Q331K
ERR6747999_Q331K
ERR6748000_Q331K
ERR6748001_Q331K
ERR6748002_Q331K
ERR6748003_Q331K
ERR6747970_Q331K
SRR11424682_M337V-01
SRR11424683_M337V-01
SRR11424684_M337V-02
SRR11424685_M337V-02
SRR11424686_M337V-03
SRR11424687_M337V-03
SRR11424678_HC-1
SRR11424679_HC-1
SRR11424681_HC-2
17641398
17641850
17642462
17642959
chr19:17641397-17642959:+
UNC13A | UNC13A-204
UNC13A | UNC13A-206
UNC13A | UNC13A-205
UNC13A | UNC13A-202

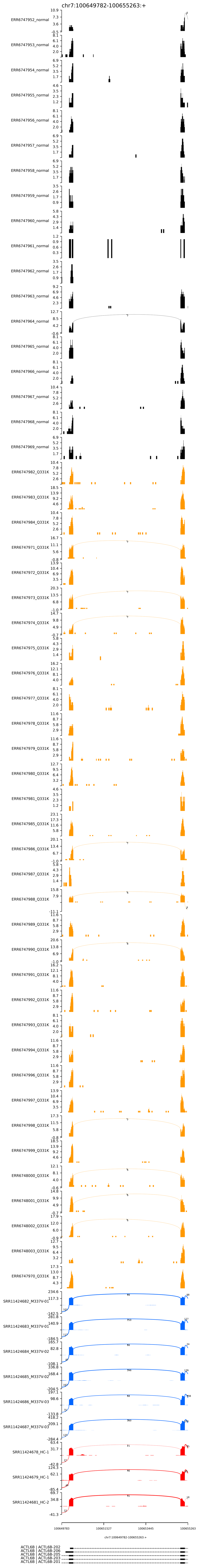
chr7:100649782-100655263:+
ERR6747952_normal
ERR6747953_normal
ERR6747954_normal
ERR6747955_normal
ERR6747956_normal
ERR6747957_normal
ERR6747958_normal
ERR6747959_normal
ERR6747960_normal
ERR6747961_normal
ERR6747962_normal
ERR6747963_normal
ERR6747964_normal
ERR6747965_normal
ERR6747966_normal
ERR6747967_normal
ERR6747968_normal
ERR6747969_normal
ERR6747982_Q331K
ERR6747983_Q331K
ERR6747984_Q331K
ERR6747971_Q331K
ERR6747972_Q331K
ERR6747973_Q331K
ERR6747974_Q331K
ERR6747975_Q331K
ERR6747976_Q331K
ERR6747977_Q331K
ERR6747978_Q331K
ERR6747979_Q331K
ERR6747980_Q331K
ERR6747981_Q331K
ERR6747985_Q331K
ERR6747986_Q331K
ERR6747987_Q331K
ERR6747988_Q331K
ERR6747989_Q331K
ERR6747990_Q331K
ERR6747991_Q331K
ERR6747992_Q331K
ERR6747993_Q331K
ERR6747994_Q331K
ERR6747996_Q331K
ERR6747997_Q331K
ERR6747998_Q331K
ERR6747999_Q331K
ERR6748000_Q331K
ERR6748001_Q331K
ERR6748002_Q331K
ERR6748003_Q331K
ERR6747970_Q331K
SRR11424682_M337V-01
SRR11424683_M337V-01
SRR11424684_M337V-01
SRR11424685_M337V-02
SRR11424686_M337V-03
SRR11424687_M337V-03
SRR11424678_HC-1
SRR11424679_HC-1
SRR11424681_HC-2
100649783
100651527
100653445
100655263
chr7:100649782-100655263:+
ACTL6B | ACTL6B-202
ACTL6B | ACTL6B-206
ACTL6B | ACTL6B-205
ACTL6B | ACTL6B-203
ACTL6B | ACTL6B-201

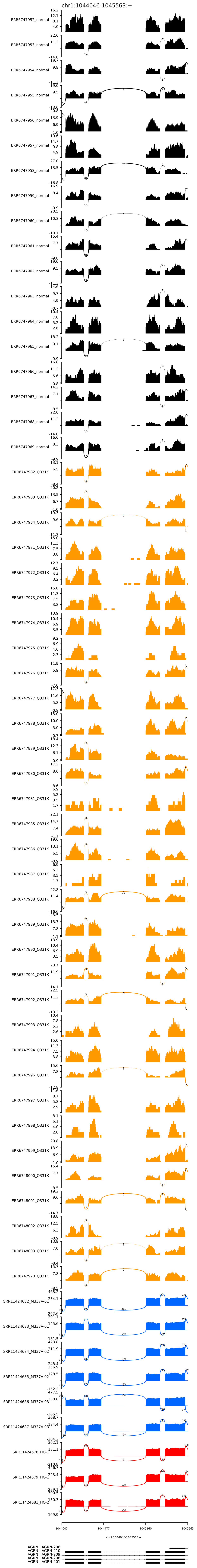

chr1:1044046-1045563:+
1044047
1044477
1045160
1045563
chr1:1044046-1045563:+
AGRN | AGRN-206
AGRN | AGRN-210
AGRN | AGRN-209
AGRN | AGRN-208
AGRN | AGRN-201

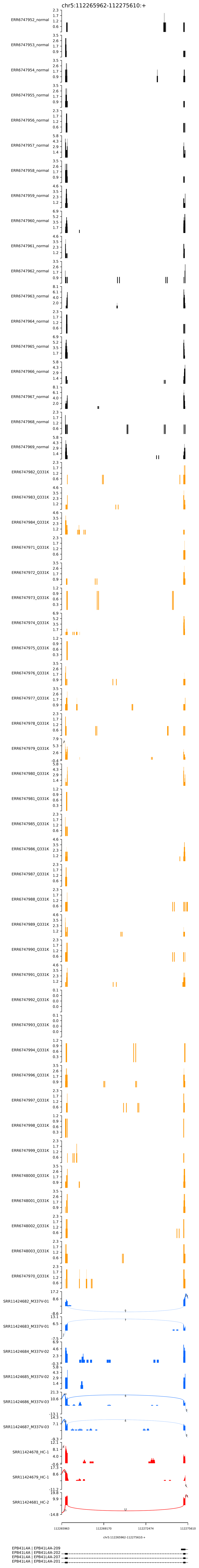
chr5:112265962-112275610:+
ERR6747952_normal
ERR6747953_normal
ERR6747954_normal
ERR6747955_normal
ERR6747956_normal
ERR6747957_normal
ERR6747958_normal
ERR6747959_normal
ERR6747960_normal
ERR6747961_normal
ERR6747962_normal
ERR6747963_normal
ERR6747964_normal
ERR6747965_normal
ERR6747966_normal
ERR6747967_normal
ERR6747968_normal
ERR6747969_normal
ERR6747982_Q331K
ERR6747983_Q331K
ERR6747984_Q331K
ERR6747971_Q331K
ERR6747972_Q331K
ERR6747973_Q331K
ERR6747974_Q331K
ERR6747975_Q331K
ERR6747976_Q331K
ERR6747977_Q331K
ERR6747978_Q331K
ERR6747979_Q331K
ERR6747980_Q331K
ERR6747981_Q331K
ERR6747985_Q331K
ERR6747986_Q331K
ERR6747987_Q331K
ERR6747988_Q331K
ERR6747989_Q331K
ERR6747990_Q331K
ERR6747991_Q331K
ERR6747992_Q331K
ERR6747993_Q331K
ERR6747994_Q331K
ERR6747996_Q331K
ERR6747997_Q331K
ERR6747998_Q331K
ERR6747999_Q331K
ERR6748000_Q331K
ERR6748001_Q331K
ERR6748002_Q331K
ERR6748003_Q331K
ERR6747970_Q331K
SRR11424682_M337V-01
SRR11424683_M337V-01
SRR11424684_M337V-02
SRR11424685_M337V-02
SRR11424686_M337V-03
SRR11424687_M337V-03
SRR11424678_HC-1
SRR11424679_HC-1
SRR11424681_HC-2
112265963
112269170
112272474
112275610
chr5:112265962-112275610:+
EP841L4A | EP841L4A-209
EP841L4A | EP841L4A-202
EP841L4A | EP841L4A-207
EP841L4A | EP841L4A-201

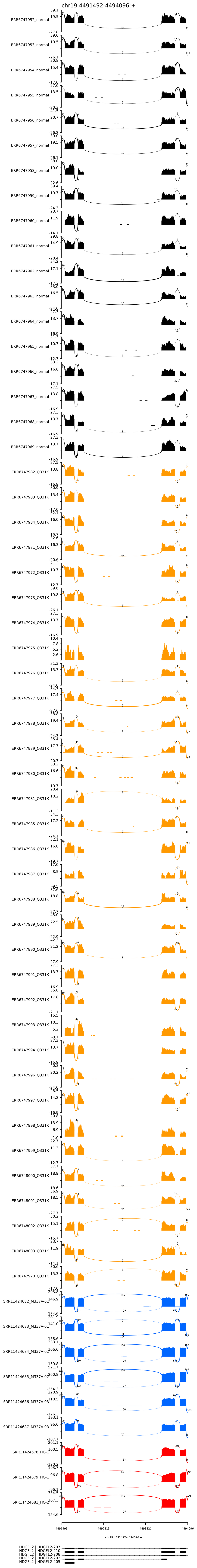
chr19:4491492-4494096:+

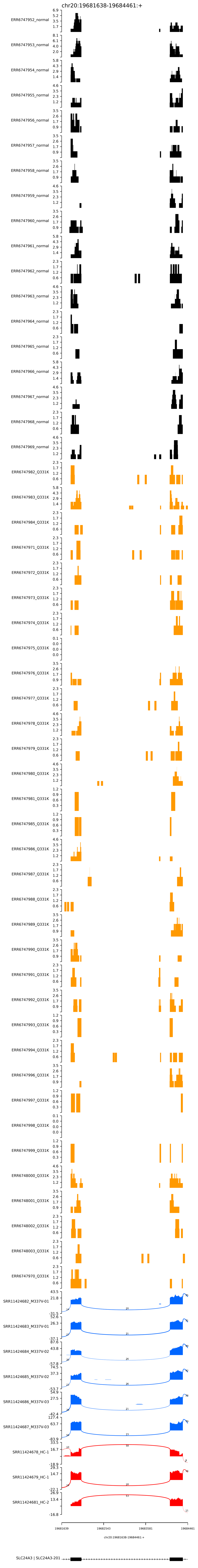
chr20:19681638-19684461:+
ERR6747952_normal
ERR6747953_normal
ERR6747954_normal
ERR6747955_normal
ERR6747956_normal
ERR6747957_normal
ERR6747958_normal
ERR6747959_normal
ERR6747960_normal
ERR6747961_normal
ERR6747962_normal
ERR6747963_normal
ERR6747964_normal
ERR6747965_normal
ERR6747966_normal
ERR6747967_normal
ERR6747968_normal
ERR6747969_normal
ERR6747982_Q331K
ERR6747983_Q331K
ERR6747984_Q331K
ERR6747971_Q331K
ERR6747972_Q331K
ERR6747973_Q331K
ERR6747974_Q331K
ERR6747975_Q331K
ERR6747976_Q331K
ERR6747977_Q331K
ERR6747978_Q331K
ERR6747979_Q331K
ERR6747980_Q331K
ERR6747981_Q331K
ERR6747985_Q331K
ERR6747986_Q331K
ERR6747987_Q331K
ERR6747988_Q331K
ERR6747989_Q331K
ERR6747990_Q331K
ERR6747991_Q331K
ERR6747992_Q331K
ERR6747993_Q331K
ERR6747994_Q331K
ERR6747996_Q331K
ERR6747997_Q331K
ERR6747998_Q331K
ERR6747999_Q331K
ERR6748000_Q331K
ERR6748001_Q331K
ERR6748002_Q331K
ERR6748003_Q331K
ERR6747970_Q331K
SRR11424682_M337V-01
SRR11424683_M337V-01
SRR11424684_M337V-02
SRR11424685_M337V-02
SRR11424686_M337V-03
SRR11424687_M337V-03
SRR11424678_HC-1
SRR11424679_HC-1
SRR11424681_HC-2
19681639
19682543
19683581
19684461
chr20:19681638-19684461:+
SLC24A3 | SLC24A3-201

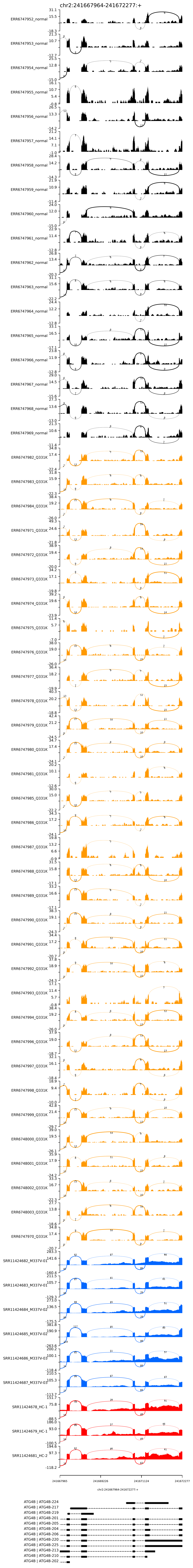
chr2:241667964-241672277:+
ERR6747952_normal
ERR6747953_normal
ERR6747954_normal
ERR6747955_normal
ERR6747956_normal
ERR6747957_normal
ERR6747958_normal
ERR6747959_normal
ERR6747960_normal
ERR6747961_normal
ERR6747962_normal
ERR6747963_normal
ERR6747964_normal
ERR6747965_normal
ERR6747966_normal
ERR6747967_normal
ERR6747968_normal
ERR6747969_normal
ERR6747982_Q331K
ERR6747983_Q331K
ERR6747984_Q331K
ERR6747971_Q331K
ERR6747972_Q331K
ERR6747973_Q331K
ERR6747974_Q331K
ERR6747975_Q331K
ERR6747976_Q331K
ERR6747977_Q331K
ERR6747978_Q331K
ERR6747979_Q331K
ERR6747980_Q331K
ERR6747981_Q331K
ERR6747985_Q331K
ERR6747986_Q331K
ERR6747987_Q331K
ERR6747988_Q331K
ERR6747989_Q331K
ERR6747990_Q331K
ERR6747991_Q331K
ERR6747992_Q331K
ERR6747993_Q331K
ERR6747994_Q331K
ERR6747996_Q331K
ERR6747997_Q331K
ERR6747998_Q331K
ERR6747999_Q331K
ERR6748000_Q331K
ERR6748001_Q331K
ERR6748002_Q331K
ERR6748003_Q331K
ERR6747970_Q331K
SRR11424682_M337V-01
SRR11424683_M337V-01
SRR11424684_M337V-02
SRR11424685_M337V-02
SRR11424686_M337V-03
SRR11424687_M337V-03
SRR11424678_HC-1
SRR11424679_HC-1
SRR11424681_HC-2
241667965
241669226
241671124
241672277
chr2:241667964-241672277:+
ATG4B | ATG4B-224
ATG4B | ATG4B-217
ATG4B | ATG4B-219
ATG4B | ATG4B-201
ATG4B | ATG4B-205
ATG4B | ATG4B-204
ATG4B | ATG4B-206
ATG4B | ATG4B-220
ATG4B | ATG4B-225
ATG4B | ATG4B-213
ATG4B | ATG4B-210
ATG4B | ATG4B-202

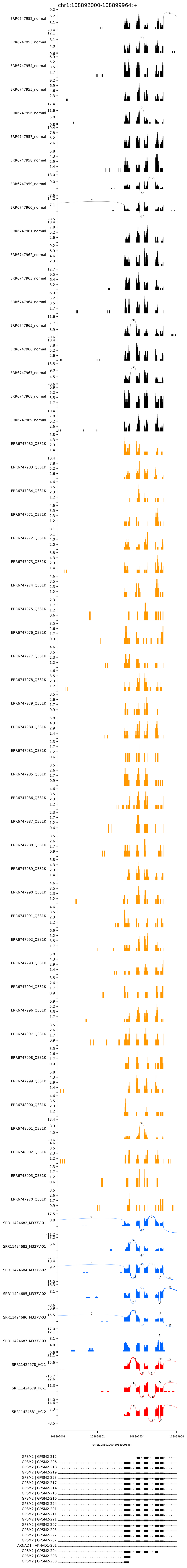
chr1:108892000-108899964:+
ERR6747952_normal
ERR6747953_normal
ERR6747954_normal
ERR6747955_normal
ERR6747956_normal
ERR6747957_normal
ERR6747958_normal
ERR6747959_normal
ERR6747960_normal
ERR6747961_normal
ERR6747962_normal
ERR6747963_normal
ERR6747964_normal
ERR6747965_normal
ERR6747966_normal
ERR6747967_normal
ERR6747968_normal
ERR6747969_normal
ERR6747982_Q331K
ERR6747983_Q331K
ERR6747984_Q331K
ERR6747971_Q331K
ERR6747972_Q331K
ERR6747973_Q331K
ERR6747974_Q331K
ERR6747975_Q331K
ERR6747976_Q331K
ERR6747977_Q331K
ERR6747978_Q331K
ERR6747979_Q331K
ERR6747980_Q331K
ERR6747981_Q331K
ERR6747985_Q331K
ERR6747986_Q331K
ERR6747987_Q331K
ERR6747988_Q331K
ERR6747989_Q331K
ERR6747990_Q331K
ERR6747991_Q331K
ERR6747992_Q331K
ERR6747993_Q331K
ERR6747994_Q331K
ERR6747996_Q331K
ERR6747997_Q331K
ERR6747998_Q331K
ERR6747999_Q331K
ERR6748000_Q331K
ERR6748001_Q331K
ERR6748002_Q331K
ERR6748003_Q331K
ERR6747970_Q331K
SRR11424682_M337V-01
SRR11424683_M337V-01
SRR11424684_M337V-02
SRR11424685_M337V-02
SRR11424686_M337V-03
SRR11424687_M337V-03
SRR11424678_HC-1
SRR11424679_HC-1
SRR11424681_HC-2
108892001
108894901
108897534
108899964
chr1:108892000-108899964:+
GPSM2 | GPSM2-212
GPSM2 | GPSM2-206
GPSM2 | GPSM2-218
GPSM2 | GPSM2-219
GPSM2 | GPSM2-223
GPSM2 | GPSM2-217
GPSM2 | GPSM2-214
GPSM2 | GPSM2-215
GPSM2 | GPSM2-216
GPSM2 | GPSM2-224
GPSM2 | GPSM2-201
GPSM2 | GPSM2-211
GPSM2 | GPSM2-221
GPSM2 | GPSM2-207
GPSM2 | GPSM2-205
GPSM2 | GPSM2-222
GPSM2 | GPSM2-202
AKNAD1 | AKNAD1-201
GPSM2 | GPSM2-204
GPSM2 | GPSM2-208
GPSM2 | GPSM2-203

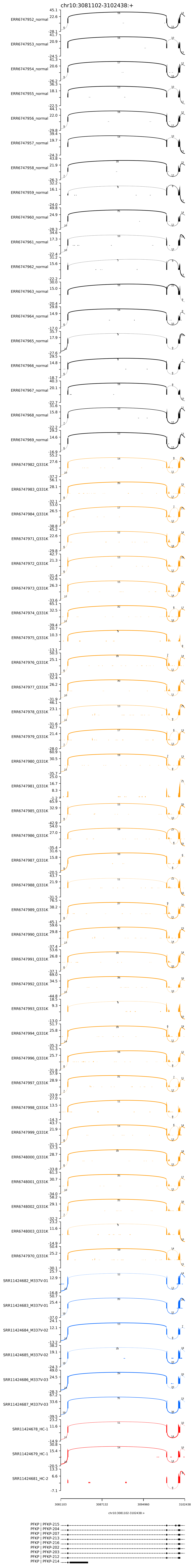
chr10:3081102-3102438:+
ERR6747952_normal
ERR6747953_normal
ERR6747954_normal
ERR6747955_normal
ERR6747956_normal
ERR6747957_normal
ERR6747958_normal
ERR6747959_normal
ERR6747960_normal
ERR6747961_normal
ERR6747962_normal
ERR6747963_normal
ERR6747964_normal
ERR6747965_normal
ERR6747966_normal
ERR6747967_normal
ERR6747968_normal
ERR6747969_normal
ERR6747982_Q331K
ERR6747983_Q331K
ERR6747984_Q331K
ERR6747971_Q331K
ERR6747972_Q331K
ERR6747973_Q331K
ERR6747974_Q331K
ERR6747975_Q331K
ERR6747976_Q331K
ERR6747977_Q331K
ERR6747978_Q331K
ERR6747979_Q331K
ERR6747980_Q331K
ERR6747981_Q331K
ERR6747985_Q331K
ERR6747986_Q331K
ERR6747987_Q331K
ERR6747988_Q331K
ERR6747989_Q331K
ERR6747990_Q331K
ERR6747991_Q331K
ERR6747992_Q331K
ERR6747993_Q331K
ERR6747994_Q331K
ERR6747996_Q331K
ERR6747997_Q331K
ERR6747998_Q331K
ERR6747999_Q331K
ERR6748000_Q331K
ERR6748001_Q331K
ERR6748002_Q331K
ERR6748003_Q331K
ERR6747970_Q331K
SRR11424682_M337V-01
SRR11424683_M337V-01
SRR11424684_M337V-02
SRR11424685_M337V-02
SRR11424686_M337V-03
SRR11424687_M337V-03
SRR11424678_HC-1
SRR11424679_HC-1
SRR11424681_HC-2
3081103
3087132
3094960
3102438
chr10:3081102-3102438:+
PFKP | PFKP-215
PFKP | PFKP-204
PFKP | PFKP-207
PFKP | PFKP-213
PFKP | PFKP-216
PFKP | PFKP-202
PFKP | PFKP-203
PFKP | PFKP-212
PFKP | PFKP-214
