# Supplementary material for: Elevated nuclear TDP-43 induces constitutive exon skipping
Source: Mol Neurodegener. 2024 Jun 9;19:45. doi: 10.1186/s13024-024-00732-w (PMC11163724; doi:10.1186/s13024-024-00732-w)
Supplement: Supplementary file 7 — Supplementary Material 7 [file 13024_2024_732_MOESM7_ESM.pdf]

| Gene | Coordinates | Splicing Type | Data |
|---|---|---|---|
| BMPR1A | chr10:86921303-86924982 | skiptic exon | Carmen-Orozco, et al. 2023 |
| CANX | chr5:179698416-179706991 | skiptic exon | Carmen-Orozco, et al. 2023 |
| COQ5 | chr12:120516319-120526842 | skiptic exon | Carmen-Orozco, et al. 2023 |
| ELP2 | chr18:36166598-36171437 | skiptic exon | Carmen-Orozco, et al. 2023 |
| HYOU1 | chr11:119052044-119052857 | skiptic exon | Carmen-Orozco, et al. 2023 |
| MYBBP1A | chr17:4544952-4545777 | skiptic exon | Carmen-Orozco, et al. 2023 |
| NUP93 | chr16:56838753-56841937 | skiptic exon | Carmen-Orozco, et al. 2023 |
| SCN9A | chr2:166272081-166280656 | skiptic exon | Carmen-Orozco, et al. 2023 |
| SESN3 | chr11:95185056-95191820 | skiptic exon | Carmen-Orozco, et al. 2023 |
| SLC35A5 | chr3:112562822-112571073 | skiptic exon | Carmen-Orozco, et al. 2023 |
| TESK1 | chr9:35606765-35607748 | skiptic exon | Carmen-Orozco, et al. 2023 |
| VARS2 | chr6:30916103-30917282 | skiptic exon | Carmen-Orozco, et al. 2023 |
| WSCD1 | chr17:6080025-6095711 | skiptic exon | Carmen-Orozco, et al. 2023 |
| XPNPEP1 | chr10:109883737-109888276 | skiptic exon | Carmen-Orozco, et al. 2023 |
| DDI2 | chr1:15651731-15662471 | skiptic exon | Carmen-Orozco, et al. 2023 |
| PLOD1 | chr1:11965458-11967116 | skiptic exon | Fratta, et al. 2018 |
| SLC6A6 | chr3:14467812-14472314 | skiptic exon | Fratta, et al. 2018 |
| ACTL6B | chr7:100649782-100655263 | cryptic exon | Irwin, et al. 2024 |
| AGRN | chr1:1044046-1045563 | cryptic exon | Irwin, et al. 2025 |
| EPB41L4A | chr5:112265962-112275610 | cryptic exon | Irwin, et al. 2026 |
| HDGFL2 | chr19:4491492-4494096 | cryptic exon | Irwin, et al. 2027 |
| SLC24A3 | chr20:19681638-19684461 | cryptic exon | Irwin, et al. 2028 |
| STMN2 | chr8:79610163-79637892 | cryptic exon | Klim, et al. 2019, Melamed et al. 2019 |
| UNC13A | chr19:17641397-17642959 | cryptic exon | Rosa Ma, et al. 2022 |
| ATG4B | chr2:241667964-241672277 | cryptic exon | Ling, et al. 2015 |
| GPSM2 | chr1:108892000-108899964 | cryptic exon | Ling, et al. 2015 |
| PFKP | chr10:3081102-3102438 | cryptic exon | Ling, et al. 2015 |

| | TDP-43-HA OFF (Skiptic PSI) | TDP-43-HA (Skiptic PSI) |
|---|---|---|
| BMPR1A | 0.00% | 5.91% |
| BMPR1A | 0.00% | 4.66% |
| BMPR1A | 0.00% | 4.57% |
| ELP2 | 0.44% | 11.59% |
| ELP2 | 0.23% | 6.24% |
| ELP2 | 0.26% | 9.23% |
| HYOU1 | 0.27% | 0.13% |
| HYOU1 | 0.17% | 2.31% |
| HYOU1 | 0.06% | 2.55% |
| MYPBBP1A | 0.87% | 8.31% |
| MYPBBP1A | 0.00% | 7.60% |
| MYPBBP1A | 0.00% | 9.21% |
| NUP93 | 2.23% | 6.08% |
| NUP93 | 2.40% | 4.82% |
| NUP93 | 1.86% | 3.10% |
| VARS2 | 0.58% | 1.50% |
| VARS2 | 0.00% | 1.92% |
| VARS2 | 0.00% | 5.60% |

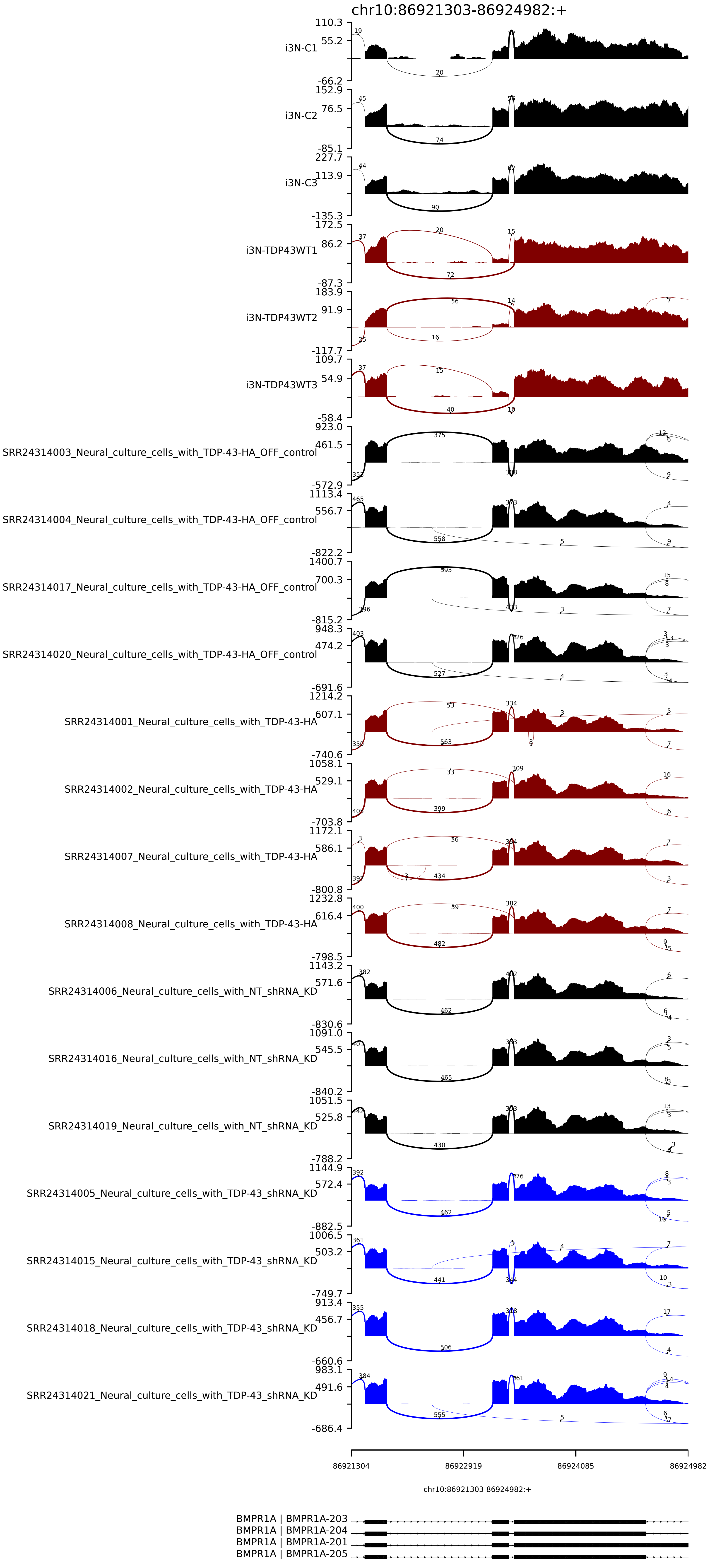
chr10:86921303-86924982:+
i3N-C1
i3N-C2
i3N-C3
i3N-TDP43WT1
i3N-TDP43WT2
i3N-TDP43WT3
SRR24314003_Neural_culture_cells_with_TDP-43-HA_OFF_control
SRR24314004_Neural_culture_cells_with_TDP-43-HA_OFF_control
SRR24314017_Neural_culture_cells_with_TDP-43-HA_OFF_control
SRR24314020_Neural_culture_cells_with_TDP-43-HA_OFF_control
SRR24314001_Neural_culture_cells_with_TDP-43-HA
SRR24314002_Neural_culture_cells_with_TDP-43-HA
SRR24314007_Neural_culture_cells_with_TDP-43-HA
SRR24314008_Neural_culture_cells_with_TDP-43-HA
SRR24314006_Neural_culture_cells_with_NT_shRNA_KD
SRR24314016_Neural_culture_cells_with_NT_shRNA_KD
SRR24314019_Neural_culture_cells_with_NT_shRNA_KD
SRR24314005_Neural_culture_cells_with_TDP-43_shRNA_KD
SRR24314015_Neural_culture_cells_with_TDP-43_shRNA_KD
SRR24314018_Neural_culture_cells_with_TDP-43_shRNA_KD
SRR24314021_Neural_culture_cells_with_TDP-43_shRNA_KD
86921304
86922919
86924085
86924982
chr10:86921303-86924982:+
BMPR1A | BMPR1A-203
BMPR1A | BMPR1A-204
BMPR1A | BMPR1A-201
BMPR1A | BMPR1A-205

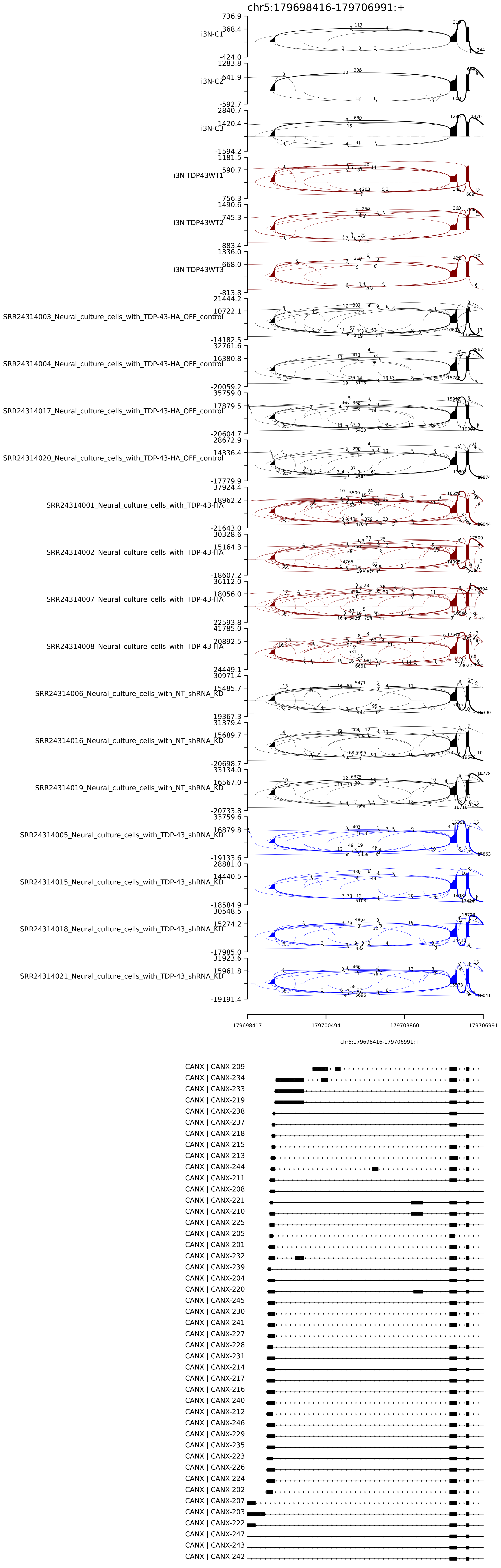
chr5:179698416-179706991:+
i3N-C1
i3N-C2
i3N-C3
i3N-TDP43WT1
i3N-TDP43WT2
i3N-TDP43WT3
SRR24314003_Neural_culture_cells_with_TDP-43-HA_OFF_control
SRR24314004_Neural_culture_cells_with_TDP-43-HA_OFF_control
SRR24314017_Neural_culture_cells_with_TDP-43-HA_OFF_control
SRR24314020_Neural_culture_cells_with_TDP-43-HA_OFF_control
SRR24314001_Neural_culture_cells_with_TDP-43-HA
SRR24314002_Neural_culture_cells_with_TDP-43-HA
SRR24314007_Neural_culture_cells_with_TDP-43-HA
SRR24314008_Neural_culture_cells_with_TDP-43-HA
SRR24314006_Neural_culture_cells_with_NT_shRNA_KD
SRR24314016_Neural_culture_cells_with_NT_shRNA_KD
SRR24314019_Neural_culture_cells_with_NT_shRNA_KD
SRR24314005_Neural_culture_cells_with_TDP-43_shRNA_KD
SRR24314015_Neural_culture_cells_with_TDP-43_shRNA_KD
SRR24314018_Neural_culture_cells_with_TDP-43_shRNA_KD
SRR24314021_Neural_culture_cells_with_TDP-43_shRNA_KD
179698417
179700494
179703860
179706991
chr5:179698416-179706991:+
CANX | CANX-209
CANX | CANX-234
CANX | CANX-233
CANX | CANX-219
CANX | CANX-238
CANX | CANX-237
CANX | CANX-218
CANX | CANX-215
CANX | CANX-213
CANX | CANX-244
CANX | CANX-211
CANX | CANX-208
CANX | CANX-221
CANX | CANX-210
CANX | CANX-225
CANX | CANX-205
CANX | CANX-201
CANX | CANX-232
CANX | CANX-239
CANX | CANX-204
CANX | CANX-220
CANX | CANX-245
CANX | CANX-230
CANX | CANX-241
CANX | CANX-227
CANX | CANX-228
CANX | CANX-231
CANX | CANX-214
CANX | CANX-217
CANX | CANX-216
CANX | CANX-240
CANX | CANX-212
CANX | CANX-246
CANX | CANX-229
CANX | CANX-235
CANX | CANX-223
CANX | CANX-226
CANX | CANX-224
CANX | CANX-202
CANX | CANX-207
CANX | CANX-203
CANX | CANX-222
CANX | CANX-247
CANX | CANX-243
CANX | CANX-242

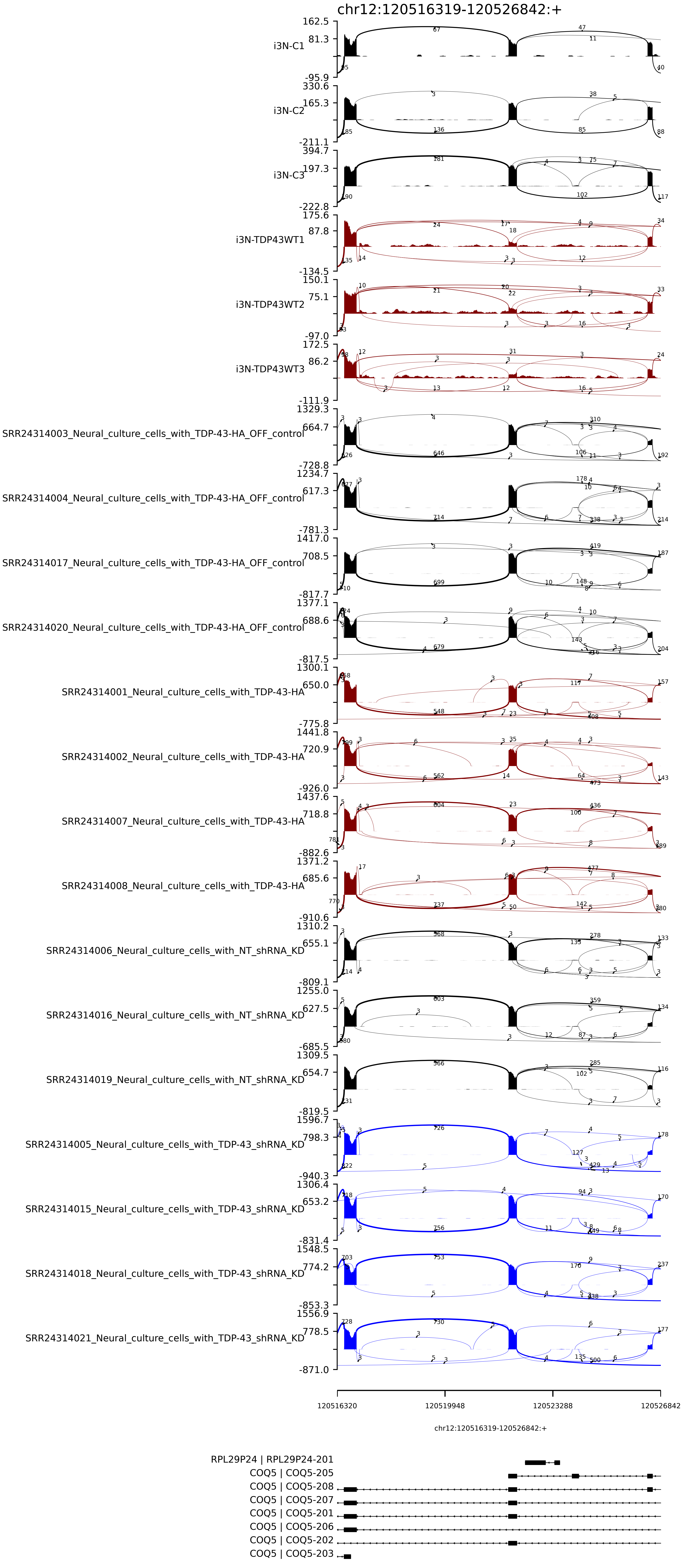

chr12:120516319-120526842:+
i3N-C1
i3N-C2
i3N-C3
i3N-TDP43WT1
i3N-TDP43WT2
i3N-TDP43WT3
SRR24314003_Neural_culture_cells_with_TDP-43-HA_OFF_control
SRR24314004_Neural_culture_cells_with_TDP-43-HA_OFF_control
SRR24314017_Neural_culture_cells_with_TDP-43-HA_OFF_control
SRR24314020_Neural_culture_cells_with_TDP-43-HA_OFF_control
SRR24314001_Neural_culture_cells_with_TDP-43-HA
SRR24314002_Neural_culture_cells_with_TDP-43-HA
SRR24314007_Neural_culture_cells_with_TDP-43-HA
SRR24314008_Neural_culture_cells_with_TDP-43-HA
SRR24314006_Neural_culture_cells_with_NT_shRNA_KD
SRR24314016_Neural_culture_cells_with_NT_shRNA_KD
SRR24314019_Neural_culture_cells_with_NT_shRNA_KD
SRR24314005_Neural_culture_cells_with_TDP-43_shRNA_KD
SRR24314015_Neural_culture_cells_with_TDP-43_shRNA_KD
SRR24314018_Neural_culture_cells_with_TDP-43_shRNA_KD
SRR24314021_Neural_culture_cells_with_TDP-43_shRNA_KD
120516320
120519948
120523288
120526842
chr12:120516319-120526842:+
RPL29P24 | RPL29P24-201
COQ5 | COQ5-205
COQ5 | COQ5-208
COQ5 | COQ5-207
COQ5 | COQ5-201
COQ5 | COQ5-206
COQ5 | COQ5-202
COQ5 | COQ5-203

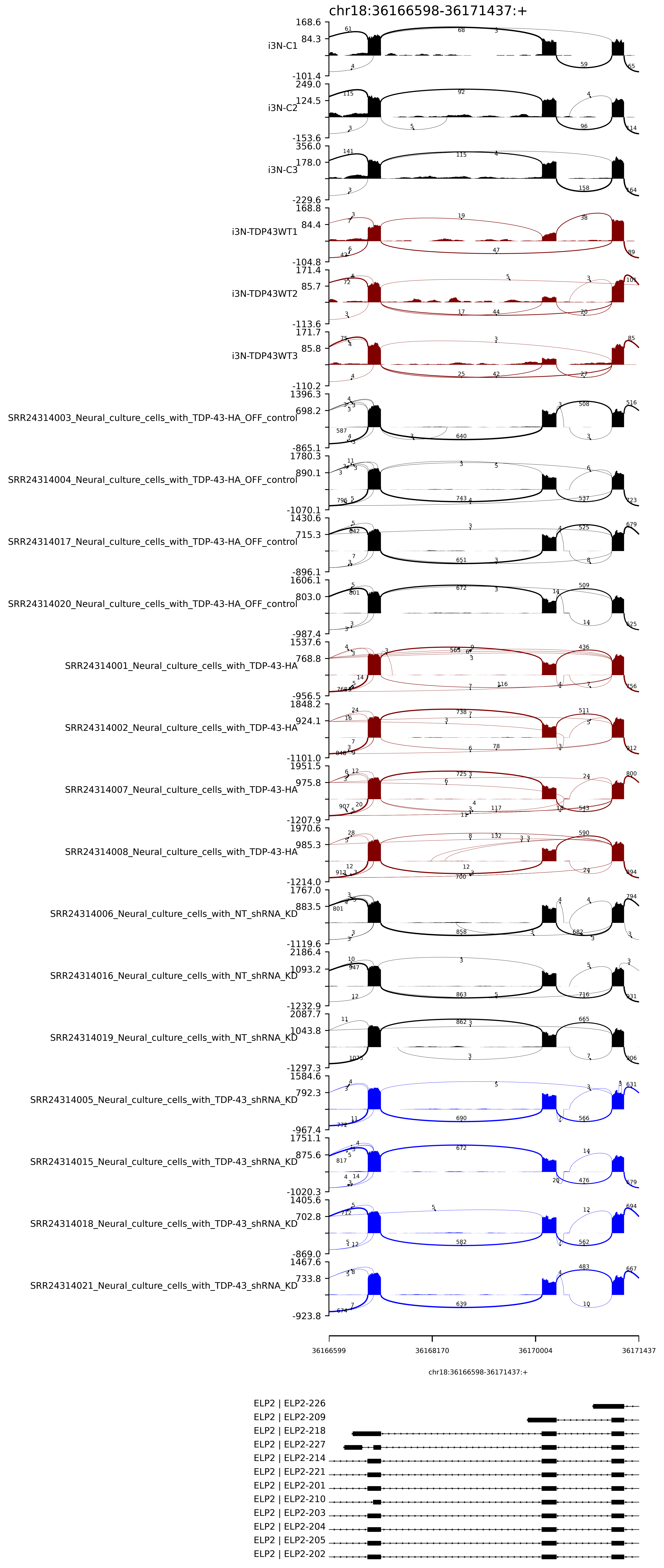
chr18:36166598-36171437:+
i3N-C1
i3N-C2
i3N-C3
i3N-TDP43WT1
i3N-TDP43WT2
i3N-TDP43WT3
SRR24314003_Neural_culture_cells_with_TDP-43-HA_OFF_control
SRR24314004_Neural_culture_cells_with_TDP-43-HA_OFF_control
SRR24314017_Neural_culture_cells_with_TDP-43-HA_OFF_control
SRR24314020_Neural_culture_cells_with_TDP-43-HA_OFF_control
SRR24314001_Neural_culture_cells_with_TDP-43-HA
SRR24314002_Neural_culture_cells_with_TDP-43-HA
SRR24314007_Neural_culture_cells_with_TDP-43-HA
SRR24314008_Neural_culture_cells_with_TDP-43-HA
SRR24314006_Neural_culture_cells_with_NT_shRNA_KD
SRR24314016_Neural_culture_cells_with_NT_shRNA_KD
SRR24314019_Neural_culture_cells_with_NT_shRNA_KD
SRR24314005_Neural_culture_cells_with_TDP-43_shRNA_KD
SRR24314015_Neural_culture_cells_with_TDP-43_shRNA_KD
SRR24314018_Neural_culture_cells_with_TDP-43_shRNA_KD
SRR24314021_Neural_culture_cells_with_TDP-43_shRNA_KD
36166599
36168170
36170004
36171437
chr18:36166598-36171437:+
ELP2 | ELP2-226
ELP2 | ELP2-209
ELP2 | ELP2-218
ELP2 | ELP2-227
ELP2 | ELP2-214
ELP2 | ELP2-221
ELP2 | ELP2-201
ELP2 | ELP2-210
ELP2 | ELP2-203
ELP2 | ELP2-204
ELP2 | ELP2-205
ELP2 | ELP2-202

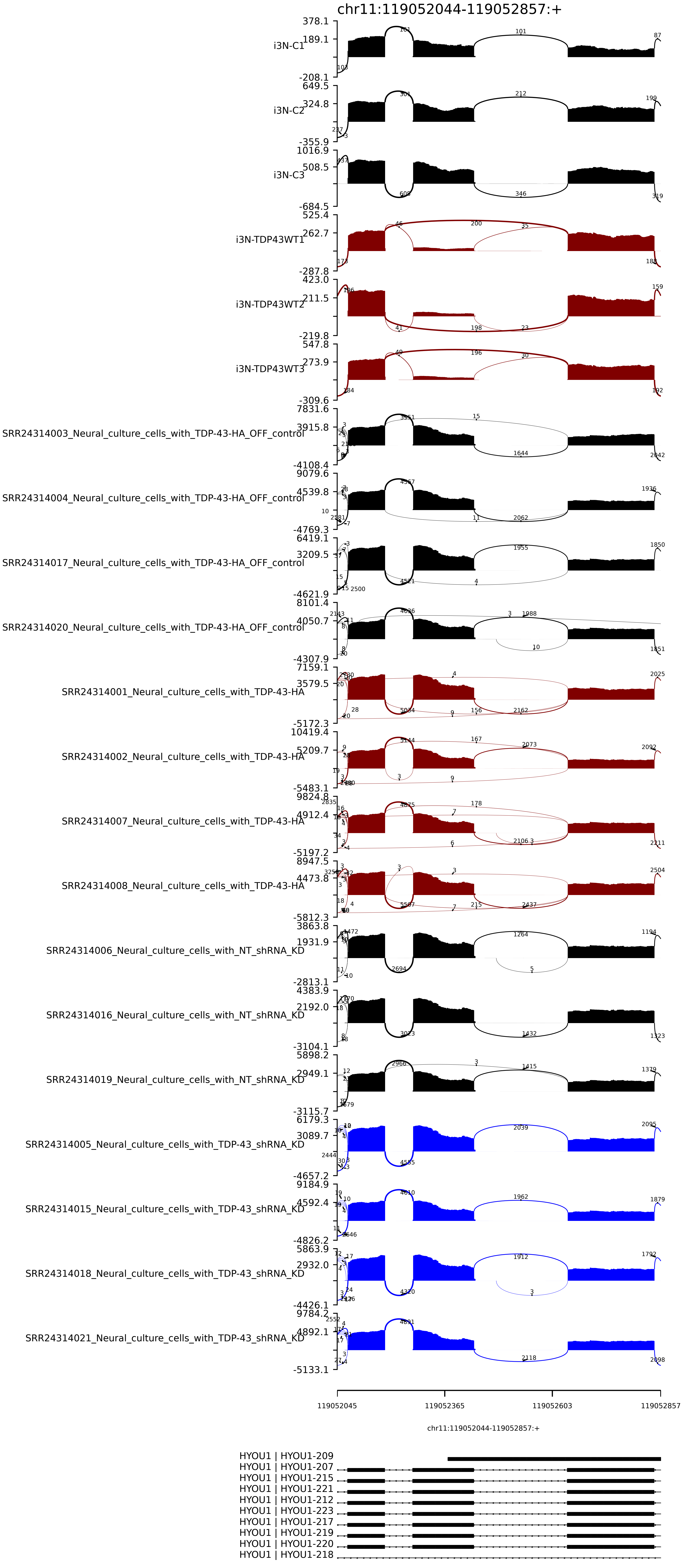
chr11:119052044-119052857:+
i3N-C1
i3N-C2
i3N-C3
i3N-TDP43WT1
i3N-TDP43WT2
i3N-TDP43WT3
SRR24314003_Neural_culture_cells_with_TDP-43-HA_OFF_control
SRR24314004_Neural_culture_cells_with_TDP-43-HA_OFF_control
SRR24314017_Neural_culture_cells_with_TDP-43-HA_OFF_control
SRR24314020_Neural_culture_cells_with_TDP-43-HA_OFF_control
SRR24314001_Neural_culture_cells_with_TDP-43-HA
SRR24314002_Neural_culture_cells_with_TDP-43-HA
SRR24314007_Neural_culture_cells_with_TDP-43-HA
SRR24314008_Neural_culture_cells_with_TDP-43-HA
SRR24314006_Neural_culture_cells_with_NT_shRNA_KD
SRR24314016_Neural_culture_cells_with_NT_shRNA_KD
SRR24314019_Neural_culture_cells_with_NT_shRNA_KD
SRR24314005_Neural_culture_cells_with_TDP-43_shRNA_KD
SRR24314015_Neural_culture_cells_with_TDP-43_shRNA_KD
SRR24314018_Neural_culture_cells_with_TDP-43_shRNA_KD
SRR24314021_Neural_culture_cells_with_TDP-43_shRNA_KD
119052045
119052365
119052603
119052857
chr11:119052044-119052857:+
HYOU1 | HYOU1-209
HYOU1 | HYOU1-207
HYOU1 | HYOU1-215
HYOU1 | HYOU1-221
HYOU1 | HYOU1-212
HYOU1 | HYOU1-223
HYOU1 | HYOU1-217
HYOU1 | HYOU1-219
HYOU1 | HYOU1-220
HYOU1 | HYOU1-218

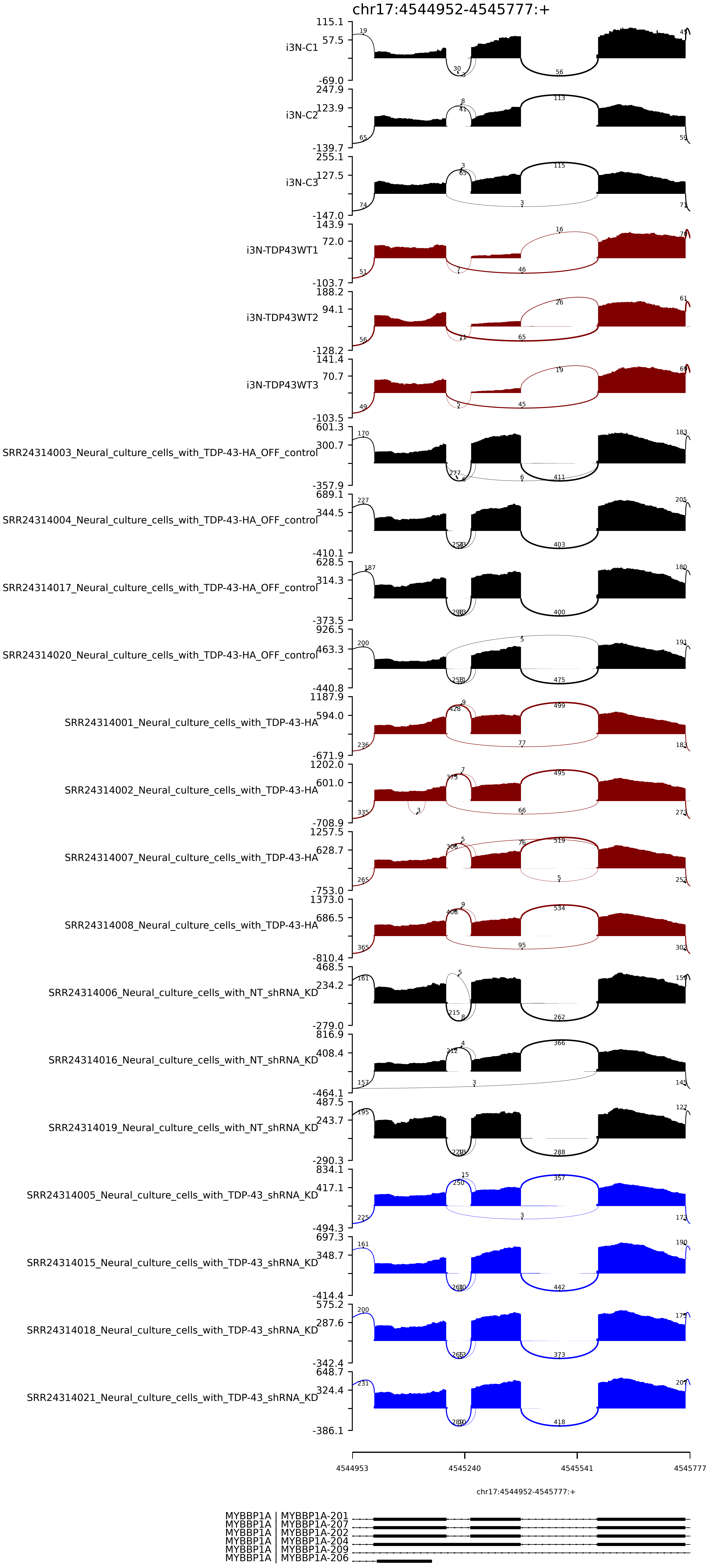

chr17:4544952-4545777:+
i3N-C1
i3N-C2
i3N-C3
i3N-TDP43WT1
i3N-TDP43WT2
i3N-TDP43WT3
SRR24314003_Neural_culture_cells_with_TDP-43-HA_OFF_control
SRR24314004_Neural_culture_cells_with_TDP-43-HA_OFF_control
SRR24314017_Neural_culture_cells_with_TDP-43-HA_OFF_control
SRR24314020_Neural_culture_cells_with_TDP-43-HA_OFF_control
SRR24314001_Neural_culture_cells_with_TDP-43-HA
SRR24314002_Neural_culture_cells_with_TDP-43-HA
SRR24314007_Neural_culture_cells_with_TDP-43-HA
SRR24314008_Neural_culture_cells_with_TDP-43-HA
SRR24314006_Neural_culture_cells_with_NT_shRNA_KD
SRR24314016_Neural_culture_cells_with_NT_shRNA_KD
SRR24314019_Neural_culture_cells_with_NT_shRNA_KD
SRR24314005_Neural_culture_cells_with_TDP-43_shRNA_KD
SRR24314015_Neural_culture_cells_with_TDP-43_shRNA_KD
SRR24314018_Neural_culture_cells_with_TDP-43_shRNA_KD
SRR24314021_Neural_culture_cells_with_TDP-43_shRNA_KD
4544953
4545240
4545541
4545777
chr17:4544952-4545777:+
MYBBP1A | MYBBP1A-201
MYBBP1A | MYBBP1A-207
MYBBP1A | MYBBP1A-202
MYBBP1A | MYBBP1A-204
MYBBP1A | MYBBP1A-209
MYBBP1A | MYBBP1A-206

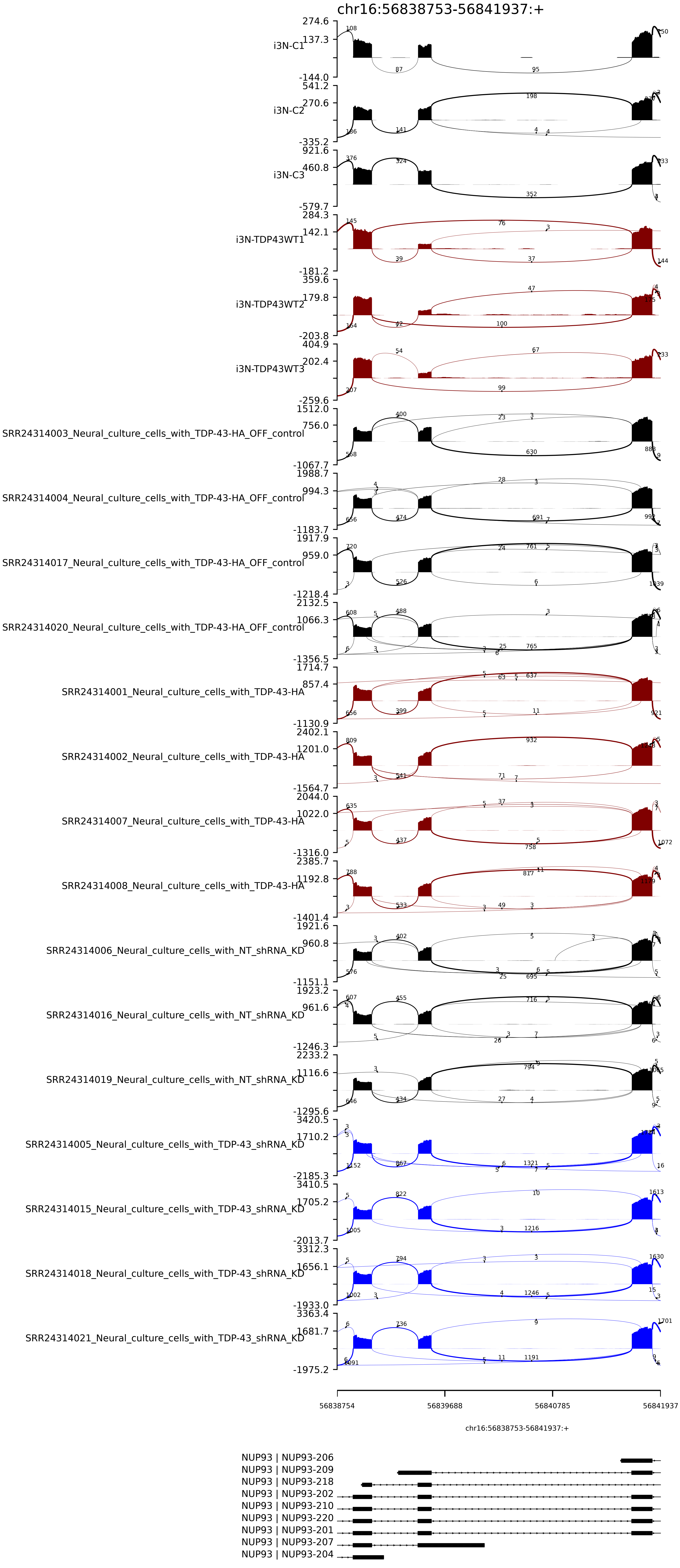

chr16:56838753-56841937:+
i3N-C1
i3N-C2
i3N-C3
i3N-TDP43WT1
i3N-TDP43WT2
i3N-TDP43WT3
SRR24314003_Neural_culture_cells_with_TDP-43-HA_OFF_control
SRR24314004_Neural_culture_cells_with_TDP-43-HA_OFF_control
SRR24314017_Neural_culture_cells_with_TDP-43-HA_OFF_control
SRR24314020_Neural_culture_cells_with_TDP-43-HA_OFF_control
SRR24314001_Neural_culture_cells_with_TDP-43-HA
SRR24314002_Neural_culture_cells_with_TDP-43-HA
SRR24314007_Neural_culture_cells_with_TDP-43-HA
SRR24314008_Neural_culture_cells_with_TDP-43-HA
SRR24314006_Neural_culture_cells_with_NT_shRNA_KD
SRR24314016_Neural_culture_cells_with_NT_shRNA_KD
SRR24314019_Neural_culture_cells_with_NT_shRNA_KD
SRR24314005_Neural_culture_cells_with_TDP-43_shRNA_KD
SRR24314015_Neural_culture_cells_with_TDP-43_shRNA_KD
SRR24314018_Neural_culture_cells_with_TDP-43_shRNA_KD
SRR24314021_Neural_culture_cells_with_TDP-43_shRNA_KD
56838754
56839688
56840785
56841937
chr16:56838753-56841937:+
NUP93 | NUP93-206
NUP93 | NUP93-209
NUP93 | NUP93-218
NUP93 | NUP93-202
NUP93 | NUP93-210
NUP93 | NUP93-220
NUP93 | NUP93-201
NUP93 | NUP93-207
NUP93 | NUP93-204

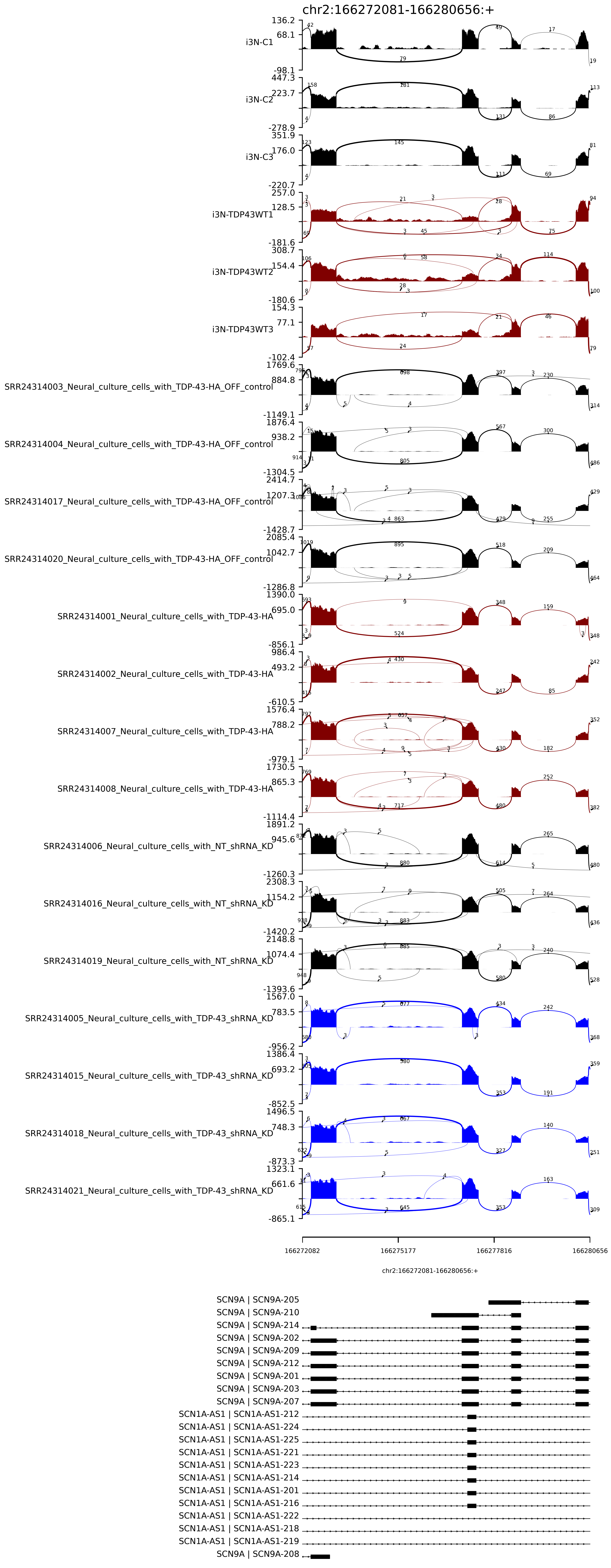
chr2:166272081-166280656:+
i3N-C1
i3N-C2
i3N-C3
i3N-TDP43WT1
i3N-TDP43WT2
i3N-TDP43WT3
SRR24314003_Neural_culture_cells_with_TDP-43-HA_OFF_control
SRR24314004_Neural_culture_cells_with_TDP-43-HA_OFF_control
SRR24314017_Neural_culture_cells_with_TDP-43-HA_OFF_control
SRR24314020_Neural_culture_cells_with_TDP-43-HA_OFF_control
SRR24314001_Neural_culture_cells_with_TDP-43-HA
SRR24314002_Neural_culture_cells_with_TDP-43-HA
SRR24314007_Neural_culture_cells_with_TDP-43-HA
SRR24314008_Neural_culture_cells_with_TDP-43-HA
SRR24314006_Neural_culture_cells_with_NT_shRNA_KD
SRR24314016_Neural_culture_cells_with_NT_shRNA_KD
SRR24314019_Neural_culture_cells_with_NT_shRNA_KD
SRR24314005_Neural_culture_cells_with_TDP-43_shRNA_KD
SRR24314015_Neural_culture_cells_with_TDP-43_shRNA_KD
SRR24314018_Neural_culture_cells_with_TDP-43_shRNA_KD
SRR24314021_Neural_culture_cells_with_TDP-43_shRNA_KD
166272082
166275177
166277816
166280656
chr2:166272081-166280656:+
SCN9A | SCN9A-205
SCN9A | SCN9A-210
SCN9A | SCN9A-214
SCN9A | SCN9A-202
SCN9A | SCN9A-209
SCN9A | SCN9A-212
SCN9A | SCN9A-201
SCN9A | SCN9A-203
SCN9A | SCN9A-207
SCN1A-AS1 | SCN1A-AS1-212
SCN1A-AS1 | SCN1A-AS1-224
SCN1A-AS1 | SCN1A-AS1-225
SCN1A-AS1 | SCN1A-AS1-221
SCN1A-AS1 | SCN1A-AS1-223
SCN1A-AS1 | SCN1A-AS1-214
SCN1A-AS1 | SCN1A-AS1-201
SCN1A-AS1 | SCN1A-AS1-216
SCN1A-AS1 | SCN1A-AS1-222
SCN1A-AS1 | SCN1A-AS1-218
SCN1A-AS1 | SCN1A-AS1-219
SCN9A | SCN9A-208

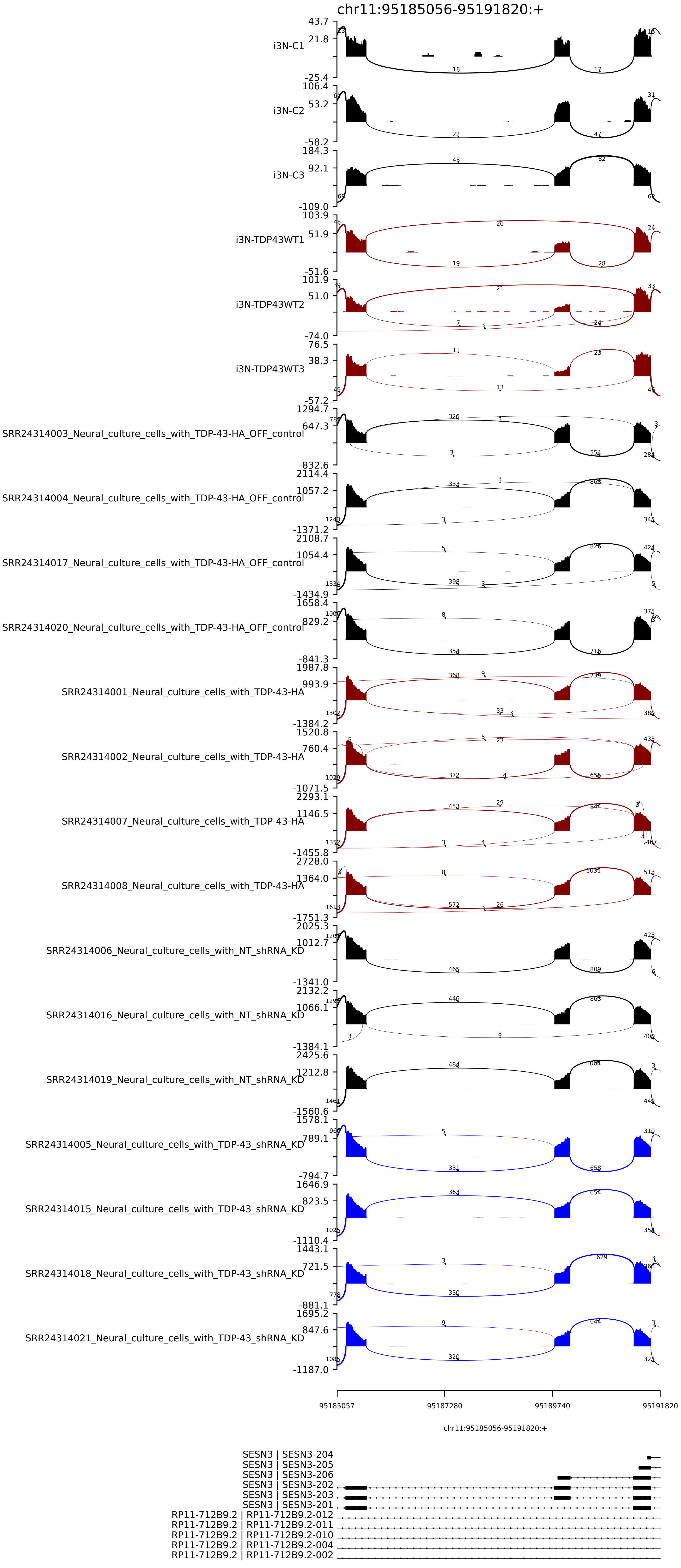
chr11:95185056-95191820:+
i3N-C1
i3N-C2
i3N-C3
i3N-TDP43WT1
i3N-TDP43WT2
i3N-TDP43WT3
SRR24314003_Neural_culture_cells_with_TDP-43-HA_OFF_control
SRR24314004_Neural_culture_cells_with_TDP-43-HA_OFF_control
SRR24314017_Neural_culture_cells_with_TDP-43-HA_OFF_control
SRR24314020_Neural_culture_cells_with_TDP-43-HA_OFF_control
SRR24314001_Neural_culture_cells_with_TDP-43-HA
SRR24314002_Neural_culture_cells_with_TDP-43-HA
SRR24314007_Neural_culture_cells_with_TDP-43-HA
SRR24314008_Neural_culture_cells_with_TDP-43-HA
SRR24314006_Neural_culture_cells_with_NT_shRNA_KD
SRR24314016_Neural_culture_cells_with_NT_shRNA_KD
SRR24314019_Neural_culture_cells_with_NT_shRNA_KD
SRR24314005_Neural_culture_cells_with_TDP-43_shRNA_KD
SRR24314015_Neural_culture_cells_with_TDP-43_shRNA_KD
SRR24314018_Neural_culture_cells_with_TDP-43_shRNA_KD
SRR24314021_Neural_culture_cells_with_TDP-43_shRNA_KD
95185057
95187280
95189740
95191820
chr11:95185056-95191820:+
SESN3 | SESN3-204
SESN3 | SESN3-205
SESN3 | SESN3-206
SESN3 | SESN3-202
SESN3 | SESN3-203
SESN3 | SESN3-201
RP11-712B9.2 | RP11-712B9.2-012
RP11-712B9.2 | RP11-712B9.2-011
RP11-712B9.2 | RP11-712B9.2-010
RP11-712B9.2 | RP11-712B9.2-004
RP11-712B9.2 | RP11-712B9.2-002

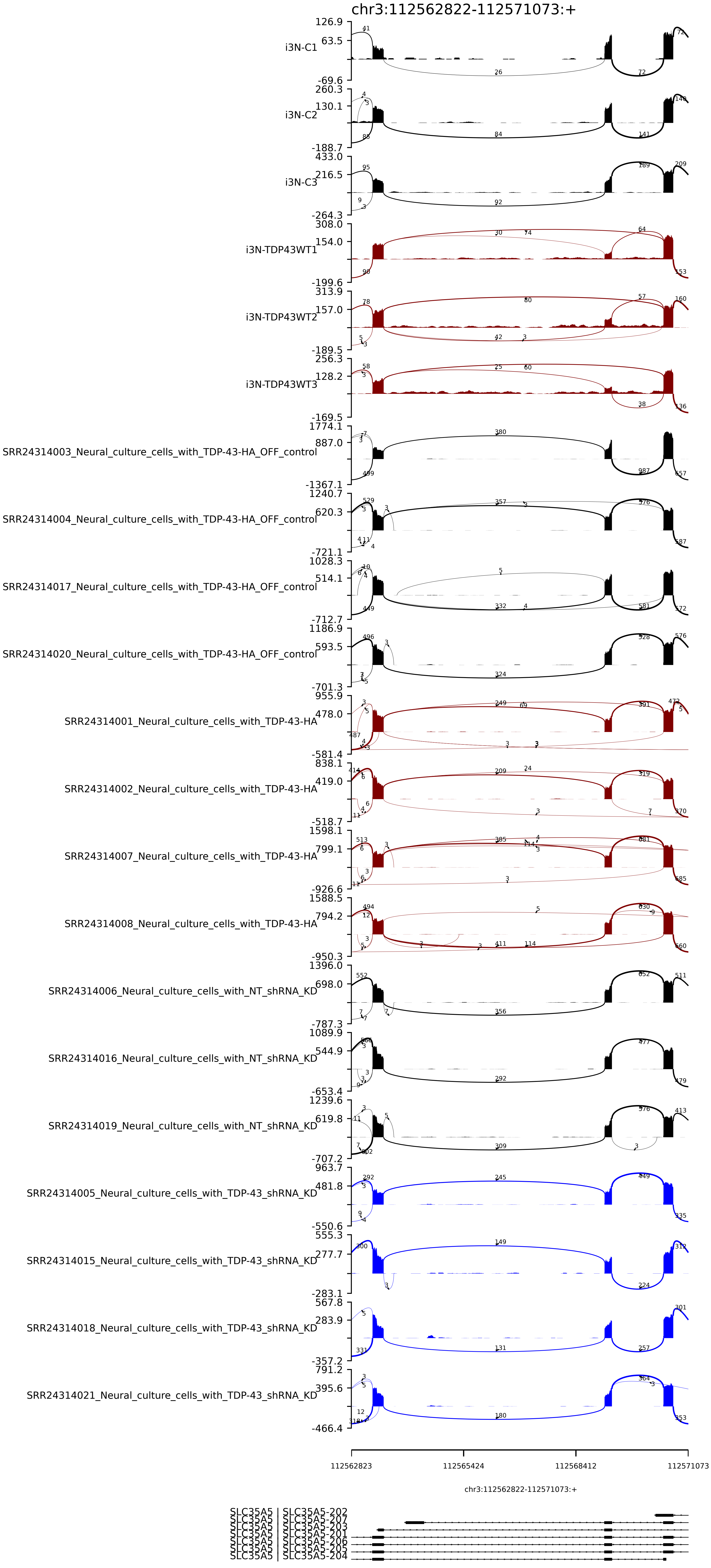

chr3:112562822-112571073:+
i3N-C1
i3N-C2
i3N-C3
i3N-TDP43WT1
i3N-TDP43WT2
i3N-TDP43WT3
SRR24314003_Neural_culture_cells_with_TDP-43-HA_OFF_control
SRR24314004_Neural_culture_cells_with_TDP-43-HA_OFF_control
SRR24314017_Neural_culture_cells_with_TDP-43-HA_OFF_control
SRR24314020_Neural_culture_cells_with_TDP-43-HA_OFF_control
SRR24314001_Neural_culture_cells_with_TDP-43-HA
SRR24314002_Neural_culture_cells_with_TDP-43-HA
SRR24314007_Neural_culture_cells_with_TDP-43-HA
SRR24314008_Neural_culture_cells_with_TDP-43-HA
SRR24314006_Neural_culture_cells_with_NT_shRNA_KD
SRR24314016_Neural_culture_cells_with_NT_shRNA_KD
SRR24314019_Neural_culture_cells_with_NT_shRNA_KD
SRR24314005_Neural_culture_cells_with_TDP-43_shRNA_KD
SRR24314015_Neural_culture_cells_with_TDP-43_shRNA_KD
SRR24314018_Neural_culture_cells_with_TDP-43_shRNA_KD
SRR24314021_Neural_culture_cells_with_TDP-43_shRNA_KD
112562823
112565424
112568412
112571073
chr3:112562822-112571073:+
SLC35A5 | SLC35A5-202
SLC35A5 | SLC35A5-207
SLC35A5 | SLC35A5-203
SLC35A5 | SLC35A5-201
SLC35A5 | SLC35A5-206
SLC35A5 | SLC35A5-205
SLC35A5 | SLC35A5-204

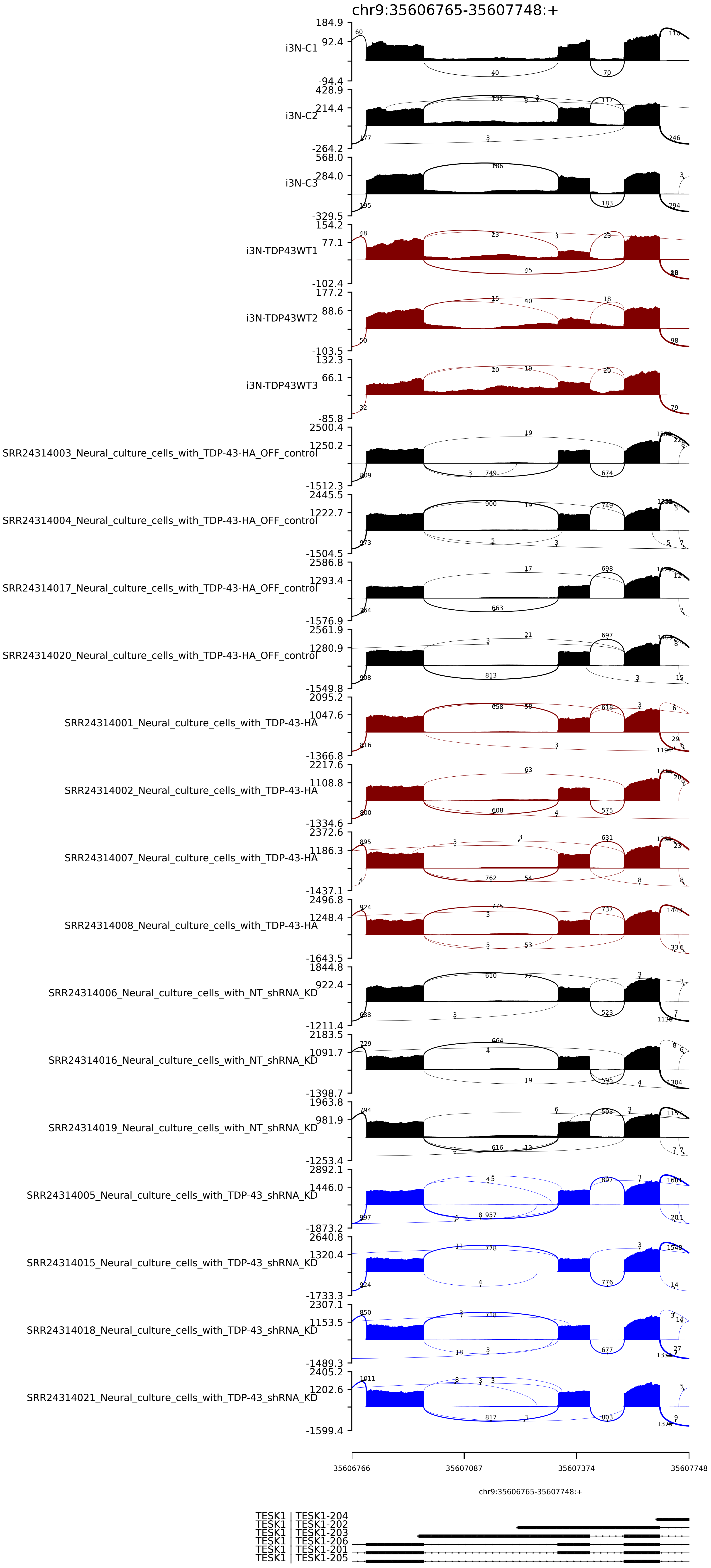
chr9:35606765-35607748:+
i3N-C1
i3N-C2
i3N-C3
i3N-TDP43WT1
i3N-TDP43WT2
i3N-TDP43WT3
SRR24314003_Neural_culture_cells_with_TDP-43-HA_OFF_control
SRR24314004_Neural_culture_cells_with_TDP-43-HA_OFF_control
SRR24314017_Neural_culture_cells_with_TDP-43-HA_OFF_control
SRR24314020_Neural_culture_cells_with_TDP-43-HA_OFF_control
SRR24314001_Neural_culture_cells_with_TDP-43-HA
SRR24314002_Neural_culture_cells_with_TDP-43-HA
SRR24314007_Neural_culture_cells_with_TDP-43-HA
SRR24314008_Neural_culture_cells_with_TDP-43-HA
SRR24314006_Neural_culture_cells_with_NT_shRNA_KD
SRR24314016_Neural_culture_cells_with_NT_shRNA_KD
SRR24314019_Neural_culture_cells_with_NT_shRNA_KD
SRR24314005_Neural_culture_cells_with_TDP-43_shRNA_KD
SRR24314015_Neural_culture_cells_with_TDP-43_shRNA_KD
SRR24314018_Neural_culture_cells_with_TDP-43_shRNA_KD
SRR24314021_Neural_culture_cells_with_TDP-43_shRNA_KD
35606766
35607087
35607374
35607748
chr9:35606765-35607748:+
TESK1 | TESK1-204
TESK1 | TESK1-202
TESK1 | TESK1-203
TESK1 | TESK1-206
TESK1 | TESK1-201
TESK1 | TESK1-205

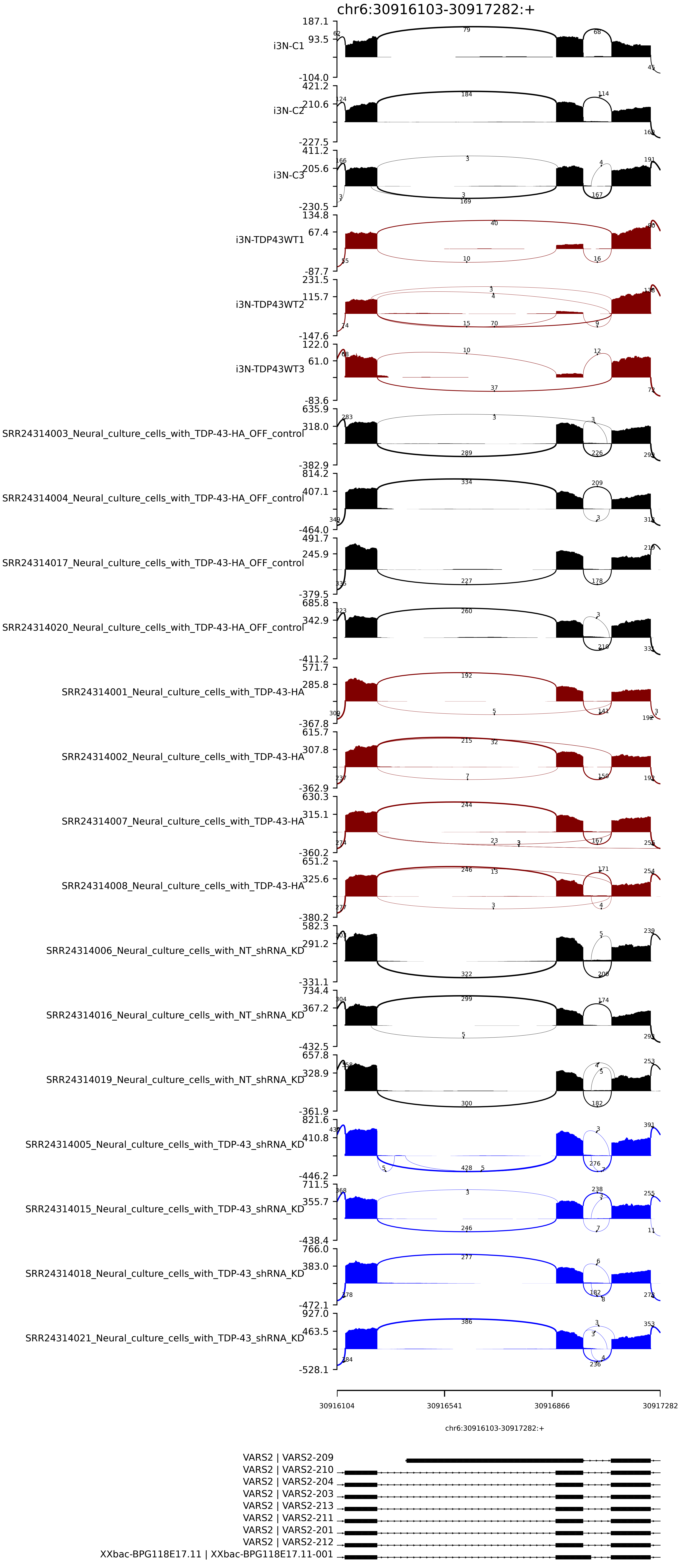
chr6:30916103-30917282:+
i3N-C1
187.1
93.5
-104.0
i3N-C2
421.2
210.6
-227.5
i3N-C3
411.2
205.6
-230.5
i3N-TDP43WT1
134.8
67.4
-87.7
i3N-TDP43WT2
231.5
115.7
-147.6
i3N-TDP43WT3
122.0
61.0
-83.6
SRR24314003_Neural_culture_cells_with_TDP-43-HA_OFF_control
635.9
318.0
-382.9
SRR24314004_Neural_culture_cells_with_TDP-43-HA_OFF_control
814.2
407.1
-464.0
SRR24314017_Neural_culture_cells_with_TDP-43-HA_OFF_control
491.7
245.9
-379.5
SRR24314020_Neural_culture_cells_with_TDP-43-HA_OFF_control
685.8
342.9
-411.2
SRR24314001_Neural_culture_cells_with_TDP-43-HA
571.7
285.8
-367.8
SRR24314002_Neural_culture_cells_with_TDP-43-HA
615.7
307.8
-362.9
SRR24314007_Neural_culture_cells_with_TDP-43-HA
630.3
315.1
-360.2
SRR24314008_Neural_culture_cells_with_TDP-43-HA
651.2
325.6
-380.2
SRR24314006_Neural_culture_cells_with_NT_shRNA_KD
582.3
291.2
-331.1
SRR24314016_Neural_culture_cells_with_NT_shRNA_KD
734.4
367.2
-432.5
SRR24314019_Neural_culture_cells_with_NT_shRNA_KD
657.8
328.9
-361.9
SRR24314005_Neural_culture_cells_with_TDP-43_shRNA_KD
821.6
410.8
-446.2
SRR24314015_Neural_culture_cells_with_TDP-43_shRNA_KD
711.5
355.7
-438.4
SRR24314018_Neural_culture_cells_with_TDP-43_shRNA_KD
766.0
383.0
-472.1
SRR24314021_Neural_culture_cells_with_TDP-43_shRNA_KD
927.0
463.5
-528.1
30916104
30916541
30916866
30917282
chr6:30916103-30917282:+
VARS2 | VARS2-209
VARS2 | VARS2-210
VARS2 | VARS2-204
VARS2 | VARS2-203
VARS2 | VARS2-213
VARS2 | VARS2-211
VARS2 | VARS2-201
VARS2 | VARS2-212
XXbac-BPG118E17.11 | XXbac-BPG118E17.11-001

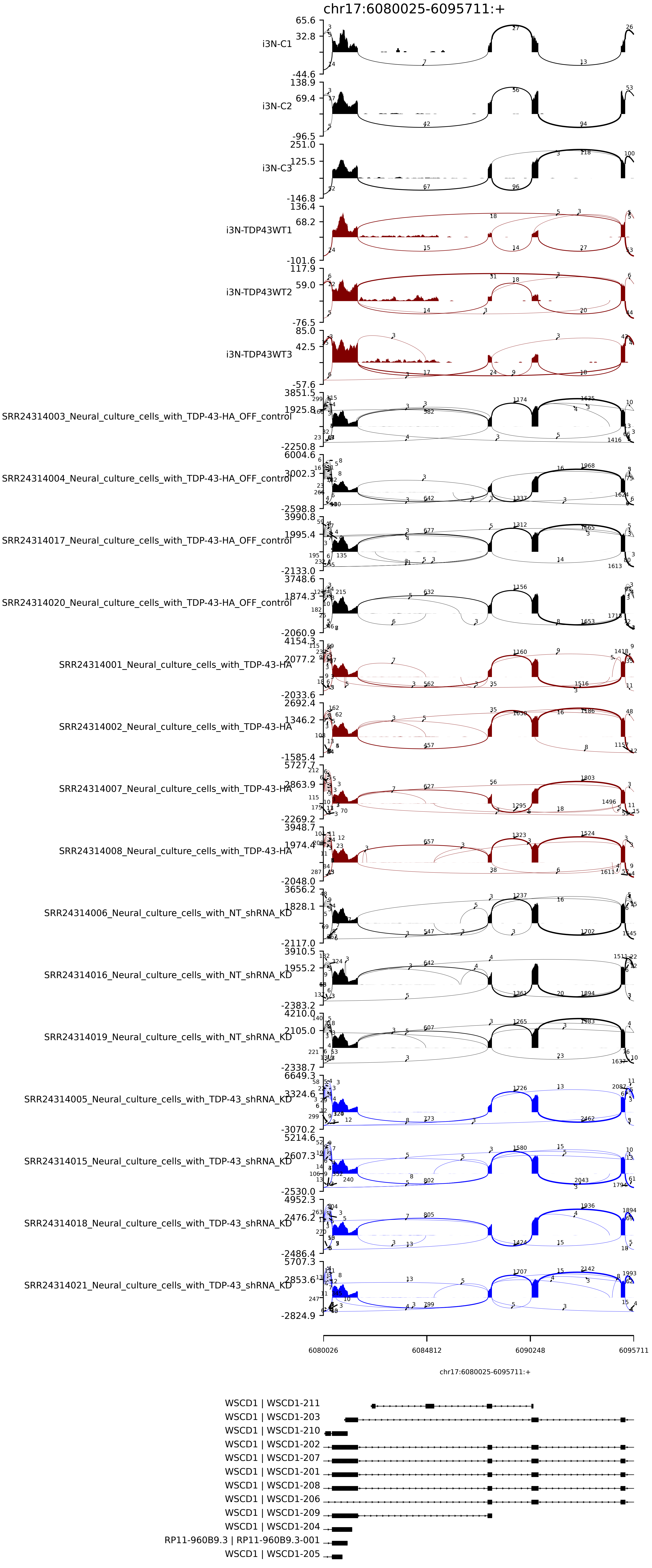
chr17:6080025-6095711:+
i3N-C1
i3N-C2
i3N-C3
i3N-TDP43WT1
i3N-TDP43WT2
i3N-TDP43WT3
SRR24314003_Neural_culture_cells_with_TDP-43-HA_OFF_control
SRR24314004_Neural_culture_cells_with_TDP-43-HA_OFF_control
SRR24314017_Neural_culture_cells_with_TDP-43-HA_OFF_control
SRR24314020_Neural_culture_cells_with_TDP-43-HA_OFF_control
SRR24314001_Neural_culture_cells_with_TDP-43-HA
SRR24314002_Neural_culture_cells_with_TDP-43-HA
SRR24314007_Neural_culture_cells_with_TDP-43-HA
SRR24314008_Neural_culture_cells_with_TDP-43-HA
SRR24314006_Neural_culture_cells_with_NT_shRNA_KD
SRR24314016_Neural_culture_cells_with_NT_shRNA_KD
SRR24314019_Neural_culture_cells_with_NT_shRNA_KD
SRR24314005_Neural_culture_cells_with_TDP-43_shRNA_KD
SRR24314015_Neural_culture_cells_with_TDP-43_shRNA_KD
SRR24314018_Neural_culture_cells_with_TDP-43_shRNA_KD
SRR24314021_Neural_culture_cells_with_TDP-43_shRNA_KD
6080026
6084812
6090248
6095711
chr17:6080025-6095711:+
WSCD1 | WSCD1-211
WSCD1 | WSCD1-203
WSCD1 | WSCD1-210
WSCD1 | WSCD1-202
WSCD1 | WSCD1-207
WSCD1 | WSCD1-201
WSCD1 | WSCD1-208
WSCD1 | WSCD1-206
WSCD1 | WSCD1-209
WSCD1 | WSCD1-204
RP11-960B9.3 | RP11-960B9.3-001
WSCD1 | WSCD1-205

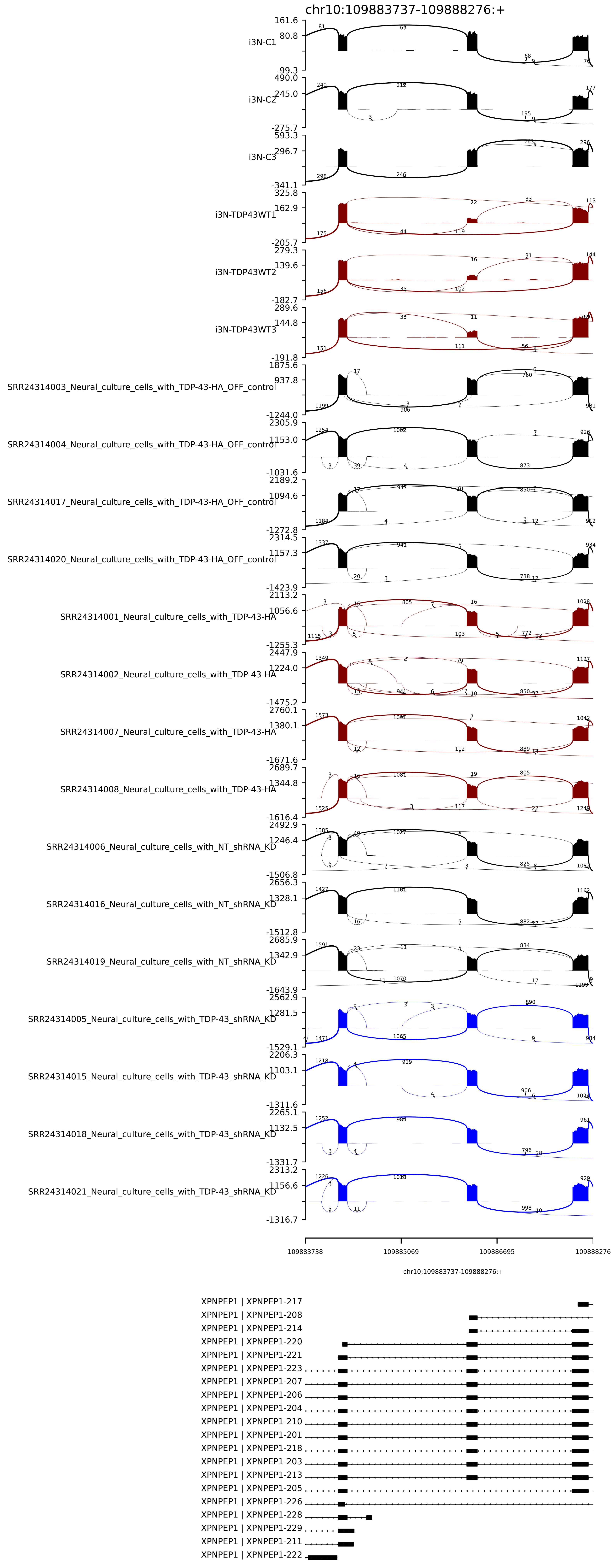
chr10:109883737-109888276:+
i3N-C1
i3N-C2
i3N-C3
i3N-TDP43WT1
i3N-TDP43WT2
i3N-TDP43WT3
SRR24314003_Neural_culture_cells_with_TDP-43-HA_OFF_control
SRR24314004_Neural_culture_cells_with_TDP-43-HA_OFF_control
SRR24314017_Neural_culture_cells_with_TDP-43-HA_OFF_control
SRR24314020_Neural_culture_cells_with_TDP-43-HA_OFF_control
SRR24314001_Neural_culture_cells_with_TDP-43-HA
SRR24314002_Neural_culture_cells_with_TDP-43-HA
SRR24314007_Neural_culture_cells_with_TDP-43-HA
SRR24314008_Neural_culture_cells_with_TDP-43-HA
SRR24314006_Neural_culture_cells_with_NT_shRNA_KD
SRR24314016_Neural_culture_cells_with_NT_shRNA_KD
SRR24314019_Neural_culture_cells_with_NT_shRNA_KD
SRR24314005_Neural_culture_cells_with_TDP-43_shRNA_KD
SRR24314015_Neural_culture_cells_with_TDP-43_shRNA_KD
SRR24314018_Neural_culture_cells_with_TDP-43_shRNA_KD
SRR24314021_Neural_culture_cells_with_TDP-43_shRNA_KD
109883738
109885069
109886695
109888276
chr10:109883737-109888276:+
XPNPEP1 | XPNPEP1-217
XPNPEP1 | XPNPEP1-208
XPNPEP1 | XPNPEP1-214
XPNPEP1 | XPNPEP1-220
XPNPEP1 | XPNPEP1-221
XPNPEP1 | XPNPEP1-223
XPNPEP1 | XPNPEP1-207
XPNPEP1 | XPNPEP1-206
XPNPEP1 | XPNPEP1-204
XPNPEP1 | XPNPEP1-210
XPNPEP1 | XPNPEP1-201
XPNPEP1 | XPNPEP1-218
XPNPEP1 | XPNPEP1-203
XPNPEP1 | XPNPEP1-213
XPNPEP1 | XPNPEP1-205
XPNPEP1 | XPNPEP1-226
XPNPEP1 | XPNPEP1-228
XPNPEP1 | XPNPEP1-229
XPNPEP1 | XPNPEP1-211
XPNPEP1 | XPNPEP1-222

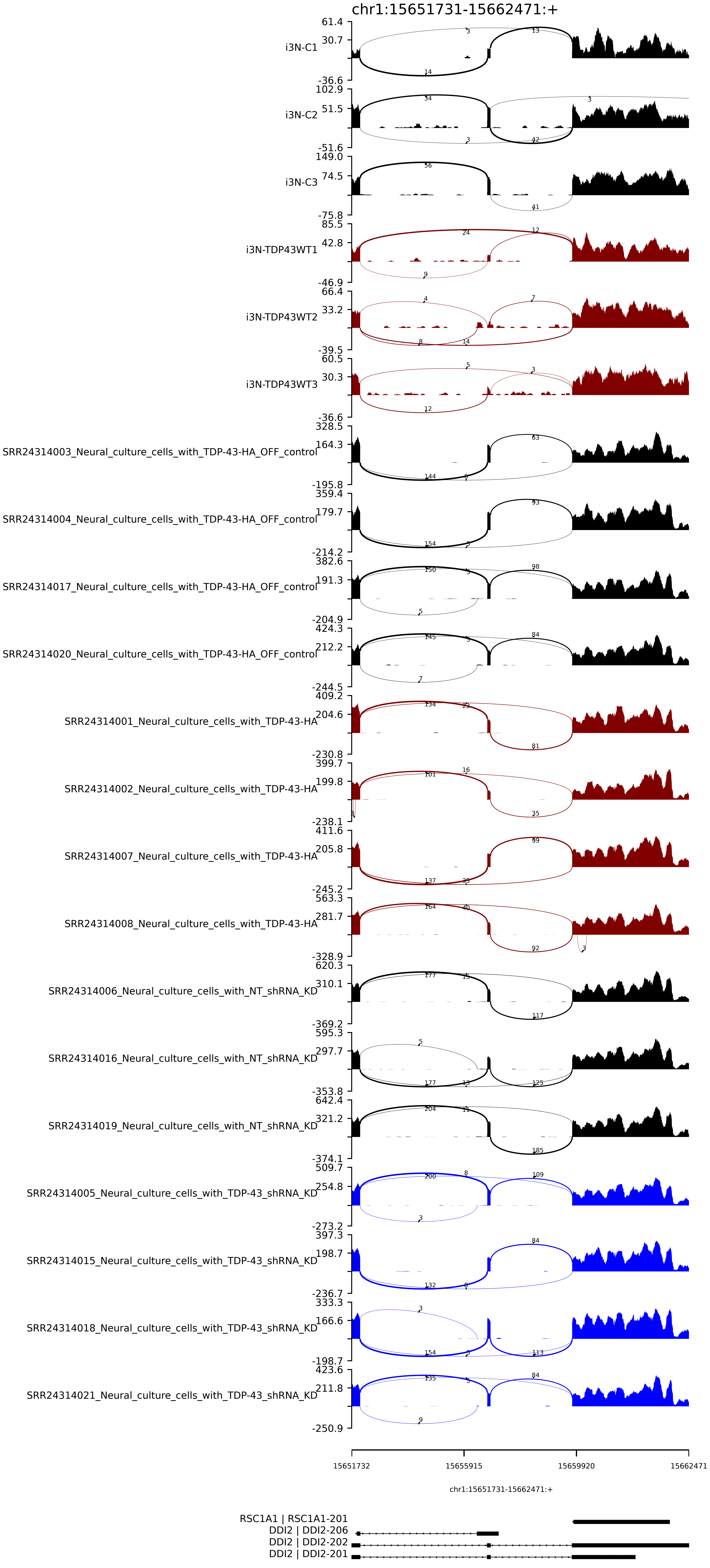
chr1:15651731-15662471:+
i3N-C1
i3N-C2
i3N-C3
i3N-TDP43WT1
i3N-TDP43WT2
i3N-TDP43WT3
SRR24314003_Neural_culture_cells_with_TDP-43-HA_OFF_control
SRR24314004_Neural_culture_cells_with_TDP-43-HA_OFF_control
SRR24314017_Neural_culture_cells_with_TDP-43-HA_OFF_control
SRR24314020_Neural_culture_cells_with_TDP-43-HA_OFF_control
SRR24314001_Neural_culture_cells_with_TDP-43-HA
SRR24314002_Neural_culture_cells_with_TDP-43-HA
SRR24314007_Neural_culture_cells_with_TDP-43-HA
SRR24314008_Neural_culture_cells_with_TDP-43-HA
SRR24314006_Neural_culture_cells_with_NT_shRNA_KD
SRR24314016_Neural_culture_cells_with_NT_shRNA_KD
SRR24314019_Neural_culture_cells_with_NT_shRNA_KD
SRR24314005_Neural_culture_cells_with_TDP-43_shRNA_KD
SRR24314015_Neural_culture_cells_with_TDP-43_shRNA_KD
SRR24314018_Neural_culture_cells_with_TDP-43_shRNA_KD
SRR24314021_Neural_culture_cells_with_TDP-43_shRNA_KD
15651732
15655915
15659920
15662471
chr1:15651731-15662471:+
RSC1A1 | RSC1A1-201
DDI2 | DDI2-206
DDI2 | DDI2-202
DDI2 | DDI2-201

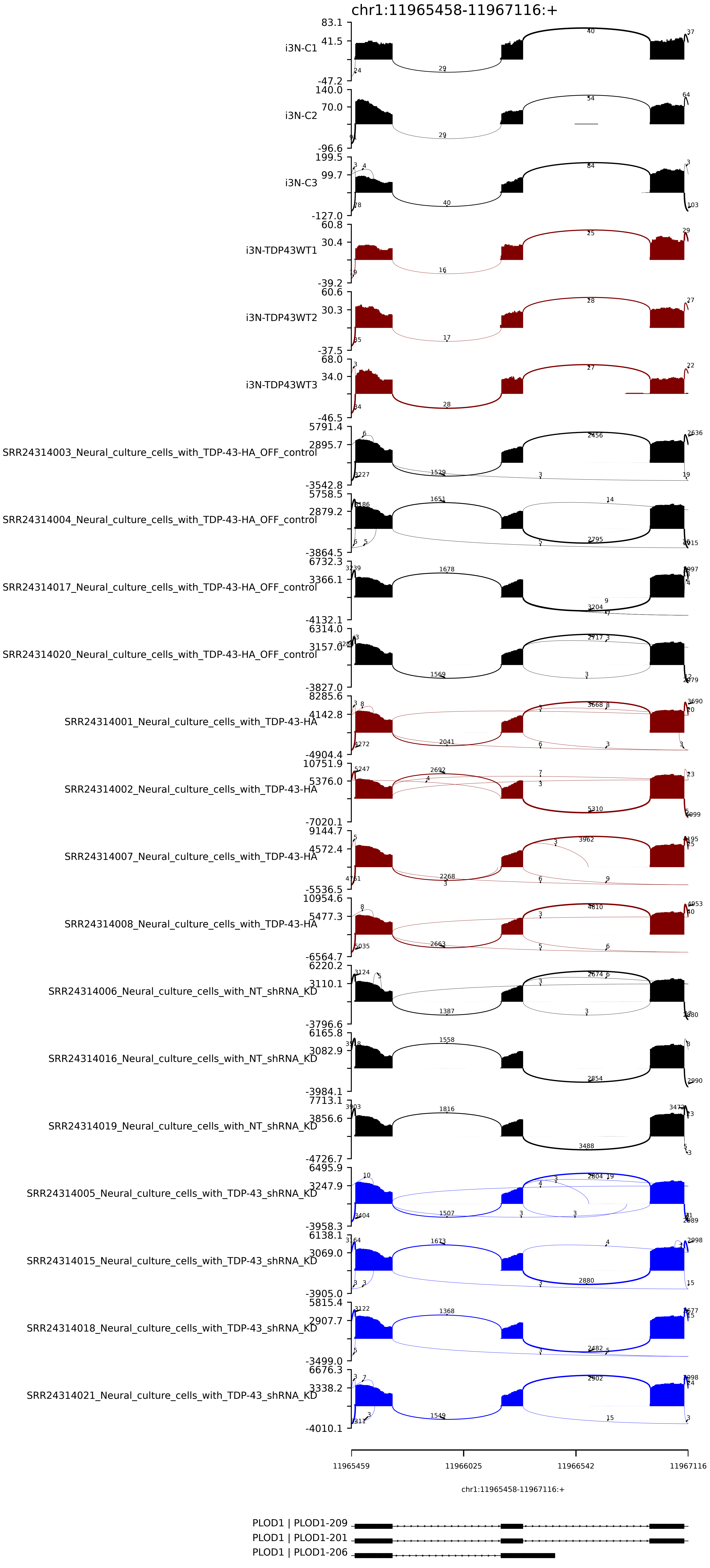
chr1:11965458-11967116:+
i3N-C1
i3N-C2
i3N-C3
i3N-TDP43WT1
i3N-TDP43WT2
i3N-TDP43WT3
SRR24314003_Neural_culture_cells_with_TDP-43-HA_OFF_control
SRR24314004_Neural_culture_cells_with_TDP-43-HA_OFF_control
SRR24314017_Neural_culture_cells_with_TDP-43-HA_OFF_control
SRR24314020_Neural_culture_cells_with_TDP-43-HA_OFF_control
SRR24314001_Neural_culture_cells_with_TDP-43-HA
SRR24314002_Neural_culture_cells_with_TDP-43-HA
SRR24314007_Neural_culture_cells_with_TDP-43-HA
SRR24314008_Neural_culture_cells_with_TDP-43-HA
SRR24314006_Neural_culture_cells_with_NT_shRNA_KD
SRR24314016_Neural_culture_cells_with_NT_shRNA_KD
SRR24314019_Neural_culture_cells_with_NT_shRNA_KD
SRR24314005_Neural_culture_cells_with_TDP-43_shRNA_KD
SRR24314015_Neural_culture_cells_with_TDP-43_shRNA_KD
SRR24314018_Neural_culture_cells_with_TDP-43_shRNA_KD
SRR24314021_Neural_culture_cells_with_TDP-43_shRNA_KD
11965459
11966025
11966542
11967116
chr1:11965458-11967116:+
PLOD1 | PLOD1-209
PLOD1 | PLOD1-201
PLOD1 | PLOD1-206

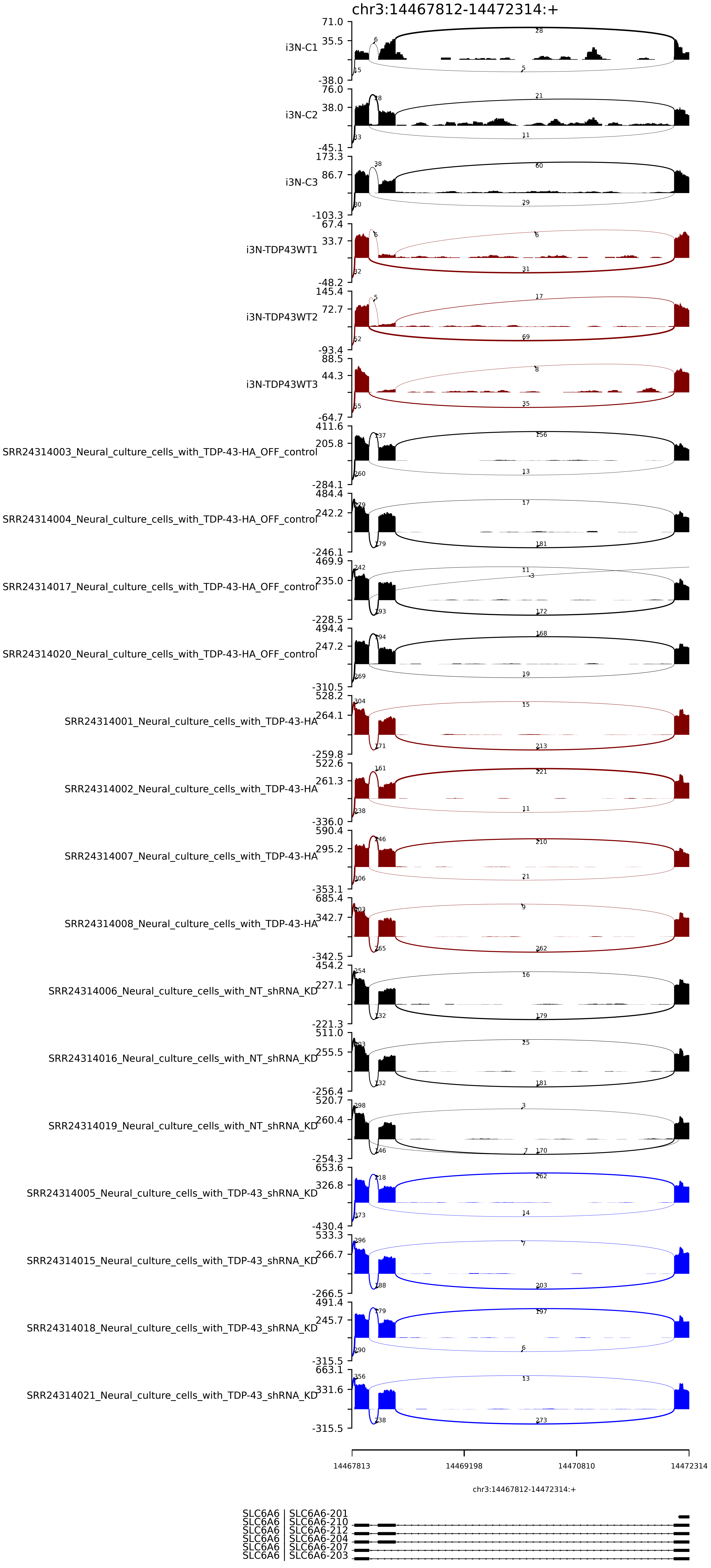
chr3:14467812-14472314:+
i3N-C1
i3N-C2
i3N-C3
i3N-TDP43WT1
i3N-TDP43WT2
i3N-TDP43WT3
SRR24314003_Neural_culture_cells_with_TDP-43-HA_OFF_control
SRR24314004_Neural_culture_cells_with_TDP-43-HA_OFF_control
SRR24314017_Neural_culture_cells_with_TDP-43-HA_OFF_control
SRR24314020_Neural_culture_cells_with_TDP-43-HA_OFF_control
SRR24314001_Neural_culture_cells_with_TDP-43-HA
SRR24314002_Neural_culture_cells_with_TDP-43-HA
SRR24314007_Neural_culture_cells_with_TDP-43-HA
SRR24314008_Neural_culture_cells_with_TDP-43-HA
SRR24314006_Neural_culture_cells_with_NT_shRNA_KD
SRR24314016_Neural_culture_cells_with_NT_shRNA_KD
SRR24314019_Neural_culture_cells_with_NT_shRNA_KD
SRR24314005_Neural_culture_cells_with_TDP-43_shRNA_KD
SRR24314015_Neural_culture_cells_with_TDP-43_shRNA_KD
SRR24314018_Neural_culture_cells_with_TDP-43_shRNA_KD
SRR24314021_Neural_culture_cells_with_TDP-43_shRNA_KD
14467813
14469198
14470810
14472314
chr3:14467812-14472314:+
SLC6A6 | SLC6A6-201
SLC6A6 | SLC6A6-210
SLC6A6 | SLC6A6-212
SLC6A6 | SLC6A6-204
SLC6A6 | SLC6A6-207
SLC6A6 | SLC6A6-203

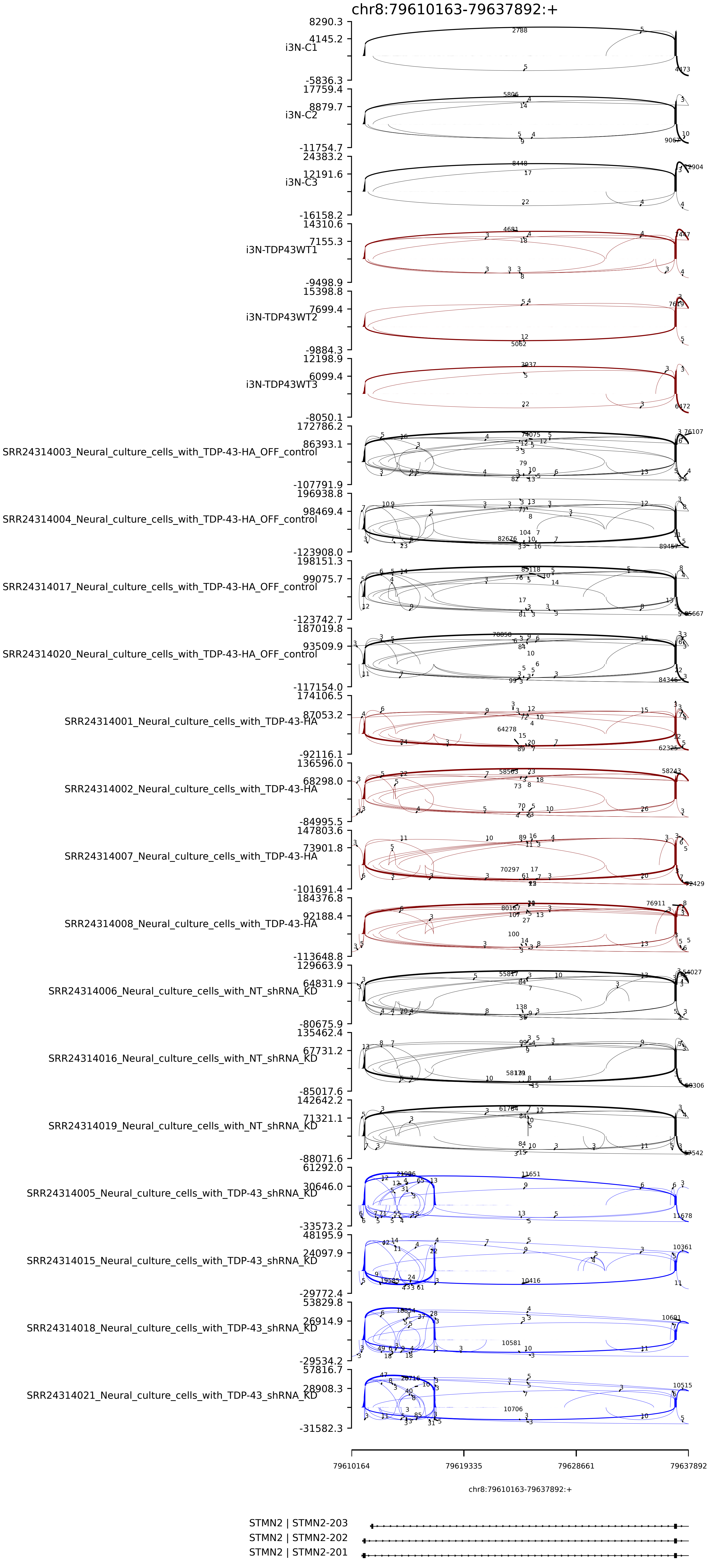
chr8:79610163-79637892:+
i3N-C1
i3N-C2
i3N-C3
i3N-TDP43WT1
i3N-TDP43WT2
i3N-TDP43WT3
SRR24314003_Neural_culture_cells_with_TDP-43-HA_OFF_control
SRR24314004_Neural_culture_cells_with_TDP-43-HA_OFF_control
SRR24314017_Neural_culture_cells_with_TDP-43-HA_OFF_control
SRR24314020_Neural_culture_cells_with_TDP-43-HA_OFF_control
SRR24314001_Neural_culture_cells_with_TDP-43-HA
SRR24314002_Neural_culture_cells_with_TDP-43-HA
SRR24314007_Neural_culture_cells_with_TDP-43-HA
SRR24314008_Neural_culture_cells_with_TDP-43-HA
SRR24314006_Neural_culture_cells_with_NT_shRNA_KD
SRR24314016_Neural_culture_cells_with_NT_shRNA_KD
SRR24314019_Neural_culture_cells_with_NT_shRNA_KD
SRR24314005_Neural_culture_cells_with_TDP-43_shRNA_KD
SRR24314015_Neural_culture_cells_with_TDP-43_shRNA_KD
SRR24314018_Neural_culture_cells_with_TDP-43_shRNA_KD
SRR24314021_Neural_culture_cells_with_TDP-43_shRNA_KD
79610164
79619335
79628661
79637892
chr8:79610163-79637892:+
STMN2 | STMN2-203
STMN2 | STMN2-202
STMN2 | STMN2-201

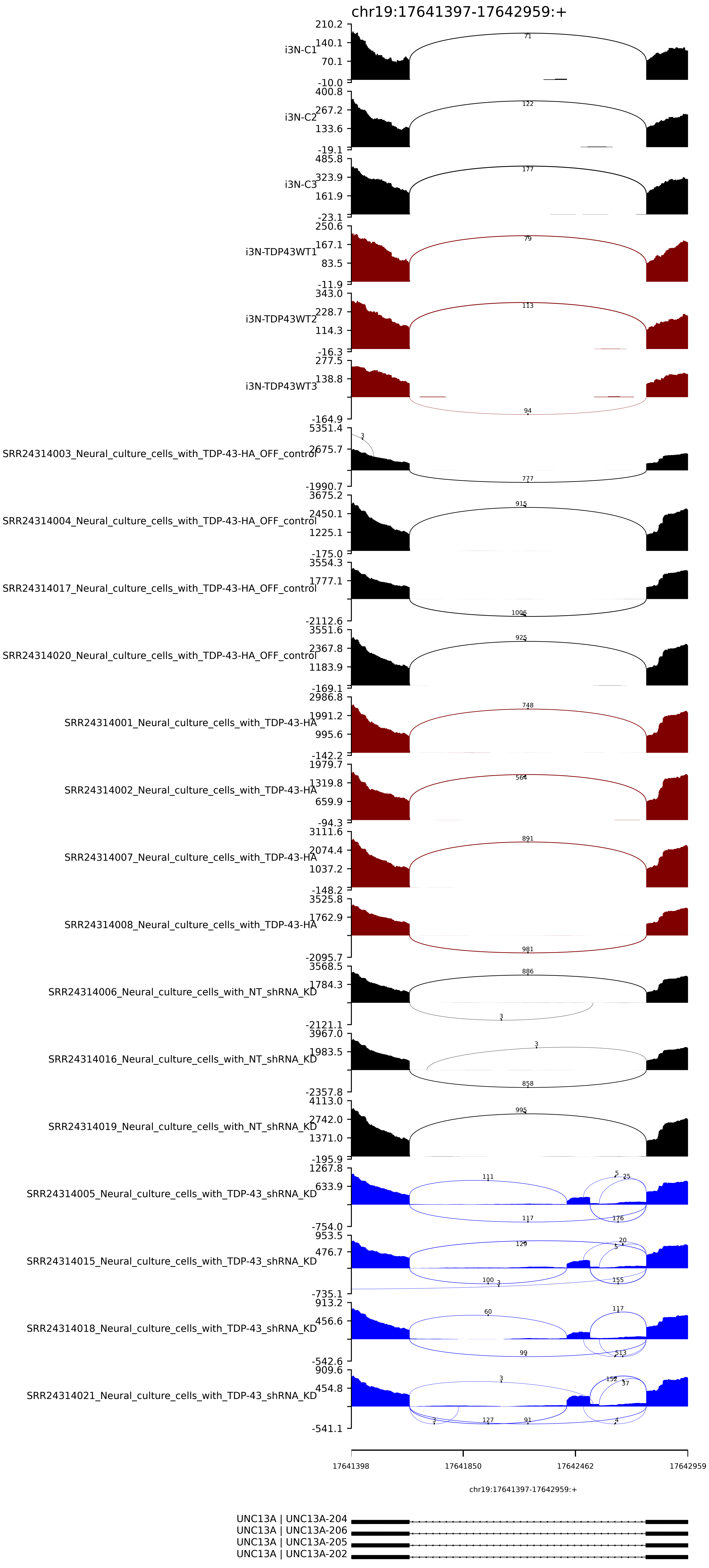
chr19:17641397-17642959:+
i3N-C1
i3N-C2
i3N-C3
i3N-TDP43WT1
i3N-TDP43WT2
i3N-TDP43WT3
SRR24314003_Neural_culture_cells_with_TDP-43-HA_OFF_control
SRR24314004_Neural_culture_cells_with_TDP-43-HA_OFF_control
SRR24314017_Neural_culture_cells_with_TDP-43-HA_OFF_control
SRR24314020_Neural_culture_cells_with_TDP-43-HA_OFF_control
SRR24314001_Neural_culture_cells_with_TDP-43-HA
SRR24314002_Neural_culture_cells_with_TDP-43-HA
SRR24314007_Neural_culture_cells_with_TDP-43-HA
SRR24314008_Neural_culture_cells_with_TDP-43-HA
SRR24314006_Neural_culture_cells_with_NT_shRNA_KD
SRR24314016_Neural_culture_cells_with_NT_shRNA_KD
SRR24314019_Neural_culture_cells_with_NT_shRNA_KD
SRR24314005_Neural_culture_cells_with_TDP-43_shRNA_KD
SRR24314015_Neural_culture_cells_with_TDP-43_shRNA_KD
SRR24314018_Neural_culture_cells_with_TDP-43_shRNA_KD
SRR24314021_Neural_culture_cells_with_TDP-43_shRNA_KD
17641398
17641850
17642462
17642959
chr19:17641397-17642959:+
UNC13A | UNC13A-204
UNC13A | UNC13A-206
UNC13A | UNC13A-205
UNC13A | UNC13A-202

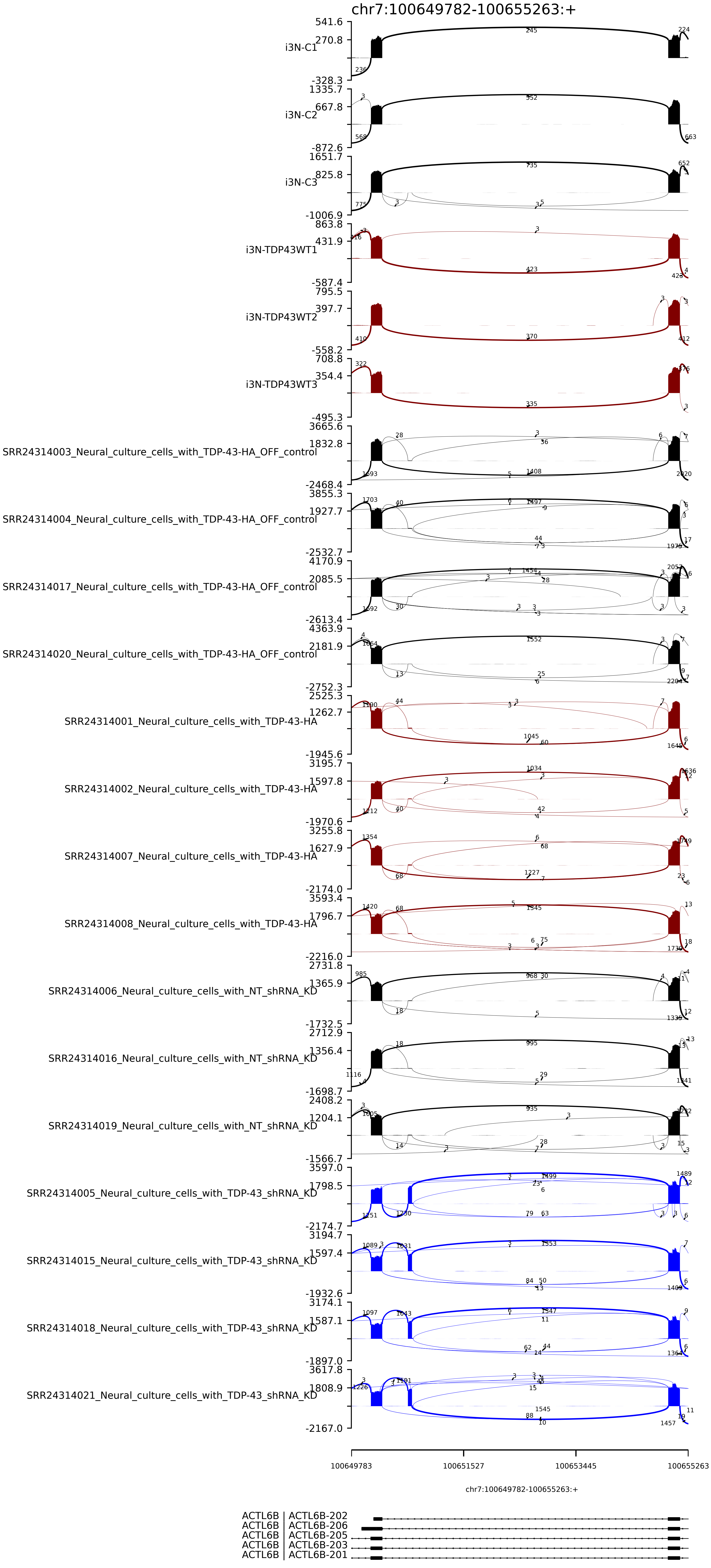
chr7:100649782-100655263:+
i3N-C1
i3N-C2
i3N-C3
i3N-TDP43WT1
i3N-TDP43WT2
i3N-TDP43WT3
SRR24314003_Neural_culture_cells_with_TDP-43-HA_OFF_control
SRR24314004_Neural_culture_cells_with_TDP-43-HA_OFF_control
SRR24314017_Neural_culture_cells_with_TDP-43-HA_OFF_control
SRR24314020_Neural_culture_cells_with_TDP-43-HA_OFF_control
SRR24314001_Neural_culture_cells_with_TDP-43-HA
SRR24314002_Neural_culture_cells_with_TDP-43-HA
SRR24314007_Neural_culture_cells_with_TDP-43-HA
SRR24314008_Neural_culture_cells_with_TDP-43-HA
SRR24314006_Neural_culture_cells_with_NT_shRNA_KD
SRR24314016_Neural_culture_cells_with_NT_shRNA_KD
SRR24314019_Neural_culture_cells_with_NT_shRNA_KD
SRR24314005_Neural_culture_cells_with_TDP-43_shRNA_KD
SRR24314015_Neural_culture_cells_with_TDP-43_shRNA_KD
SRR24314018_Neural_culture_cells_with_TDP-43_shRNA_KD
SRR24314021_Neural_culture_cells_with_TDP-43_shRNA_KD
100649783
100651527
100653445
100655263
chr7:100649782-100655263:+
ACTL6B | ACTL6B-202
ACTL6B | ACTL6B-206
ACTL6B | ACTL6B-205
ACTL6B | ACTL6B-203
ACTL6B | ACTL6B-201

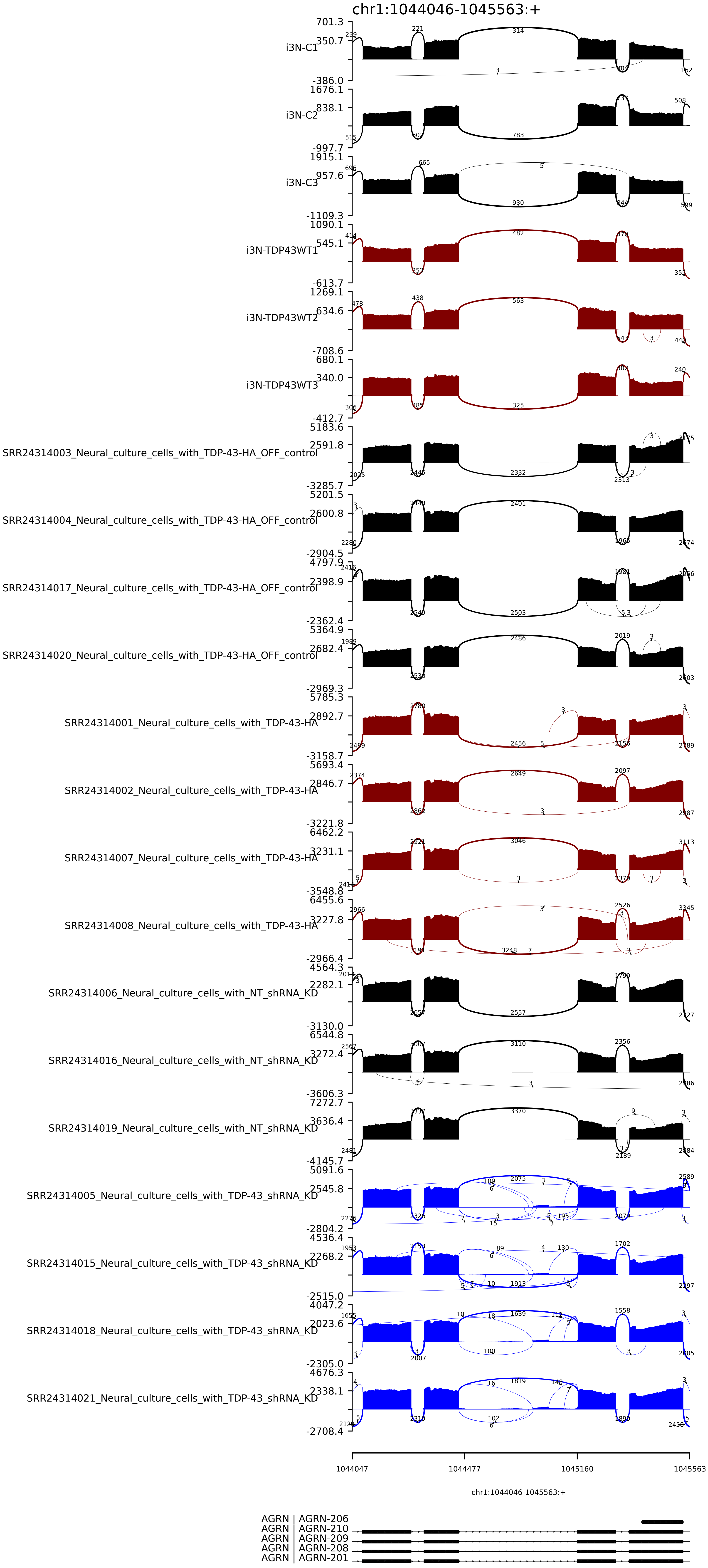

chr1:1044046-1045563:+
i3N-C1
i3N-C2
i3N-C3
i3N-TDP43WT1
i3N-TDP43WT2
i3N-TDP43WT3
SRR24314003_Neural_culture_cells_with_TDP-43-HA_OFF_control
SRR24314004_Neural_culture_cells_with_TDP-43-HA_OFF_control
SRR24314017_Neural_culture_cells_with_TDP-43-HA_OFF_control
SRR24314020_Neural_culture_cells_with_TDP-43-HA_OFF_control
SRR24314001_Neural_culture_cells_with_TDP-43-HA
SRR24314002_Neural_culture_cells_with_TDP-43-HA
SRR24314007_Neural_culture_cells_with_TDP-43-HA
SRR24314008_Neural_culture_cells_with_TDP-43-HA
SRR24314006_Neural_culture_cells_with_NT_shRNA_KD
SRR24314016_Neural_culture_cells_with_NT_shRNA_KD
SRR24314019_Neural_culture_cells_with_NT_shRNA_KD
SRR24314005_Neural_culture_cells_with_TDP-43_shRNA_KD
SRR24314015_Neural_culture_cells_with_TDP-43_shRNA_KD
SRR24314018_Neural_culture_cells_with_TDP-43_shRNA_KD
SRR24314021_Neural_culture_cells_with_TDP-43_shRNA_KD
1044047
1044477
1045160
1045563
chr1:1044046-1045563:+
AGRN | AGRN-206
AGRN | AGRN-210
AGRN | AGRN-209
AGRN | AGRN-208
AGRN | AGRN-201

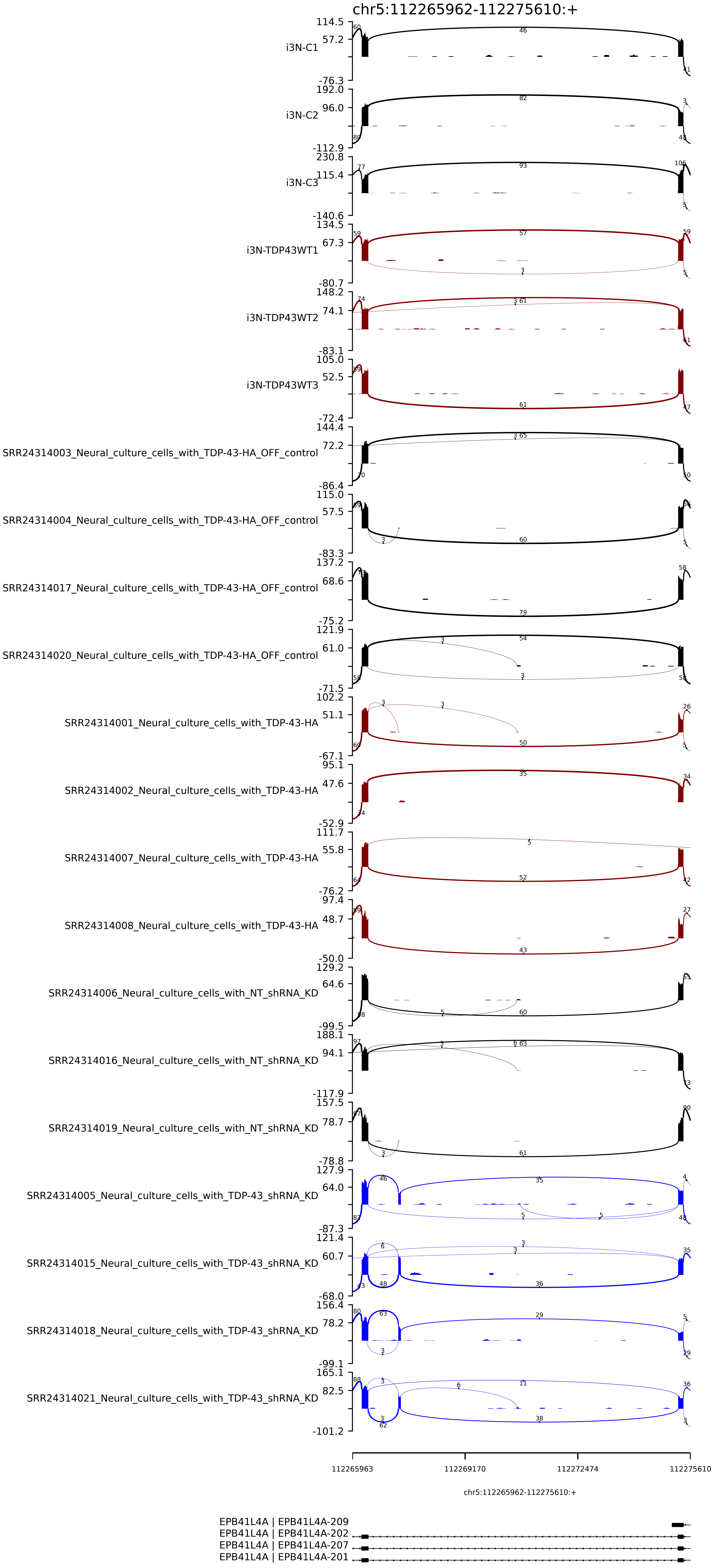

chr5:112265962-112275610:+
i3N-C1
i3N-C2
i3N-C3
i3N-TDP43WT1
i3N-TDP43WT2
i3N-TDP43WT3
SRR24314003_Neural_culture_cells_with_TDP-43-HA_OFF_control
SRR24314004_Neural_culture_cells_with_TDP-43-HA_OFF_control
SRR24314017_Neural_culture_cells_with_TDP-43-HA_OFF_control
SRR24314020_Neural_culture_cells_with_TDP-43-HA_OFF_control
SRR24314001_Neural_culture_cells_with_TDP-43-HA
SRR24314002_Neural_culture_cells_with_TDP-43-HA
SRR24314007_Neural_culture_cells_with_TDP-43-HA
SRR24314008_Neural_culture_cells_with_TDP-43-HA
SRR24314006_Neural_culture_cells_with_NT_shRNA_KD
SRR24314016_Neural_culture_cells_with_NT_shRNA_KD
SRR24314019_Neural_culture_cells_with_NT_shRNA_KD
SRR24314005_Neural_culture_cells_with_TDP-43_shRNA_KD
SRR24314015_Neural_culture_cells_with_TDP-43_shRNA_KD
SRR24314018_Neural_culture_cells_with_TDP-43_shRNA_KD
SRR24314021_Neural_culture_cells_with_TDP-43_shRNA_KD
112265963
112269170
112272474
112275610
chr5:112265962-112275610:+
EPB41L4A | EPB41L4A-209
EPB41L4A | EPB41L4A-202
EPB41L4A | EPB41L4A-207
EPB41L4A | EPB41L4A-201

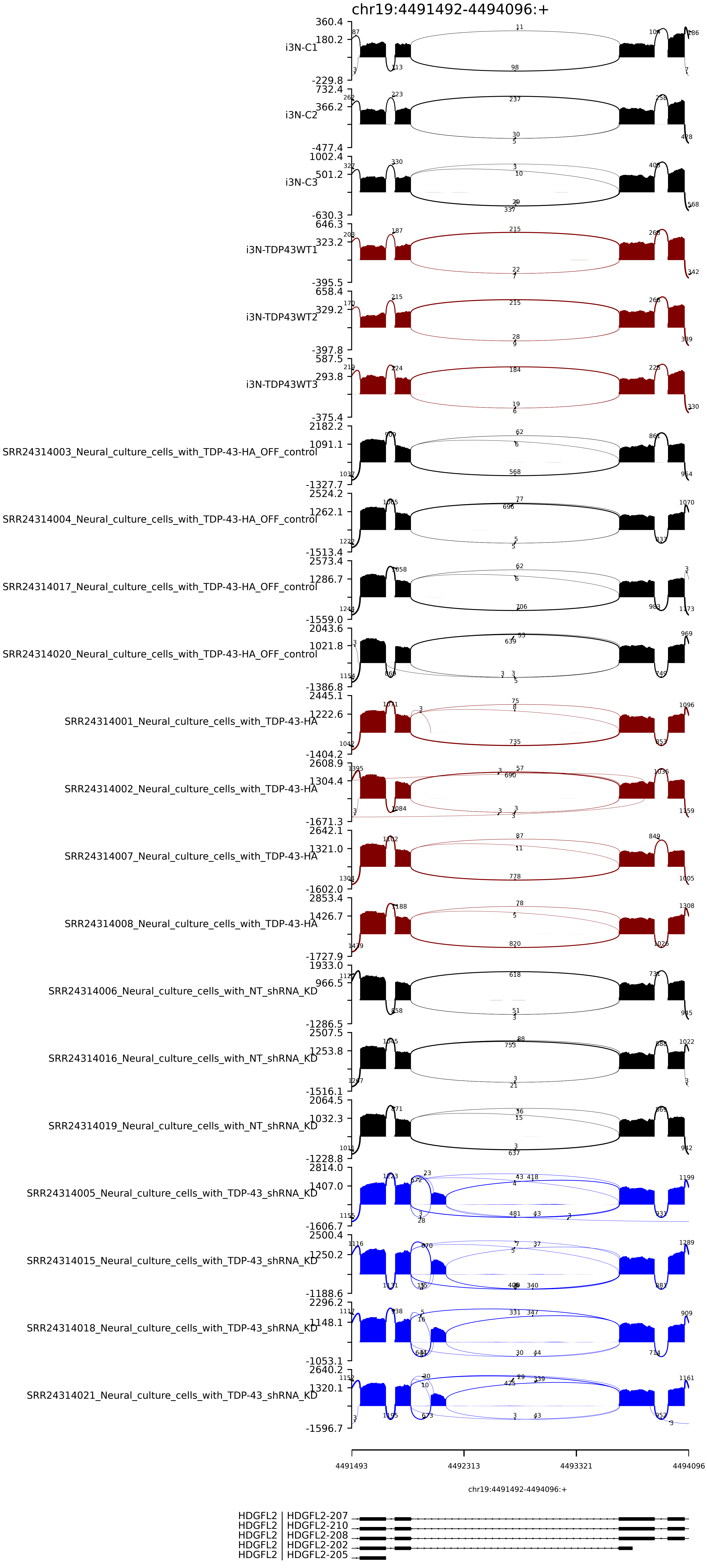
chr19:4491492-4494096:+
i3N-C1
i3N-C2
i3N-C3
i3N-TDP43WT1
i3N-TDP43WT2
i3N-TDP43WT3
SRR24314003_Neural_culture_cells_with_TDP-43-HA_OFF_control
SRR24314004_Neural_culture_cells_with_TDP-43-HA_OFF_control
SRR24314017_Neural_culture_cells_with_TDP-43-HA_OFF_control
SRR24314020_Neural_culture_cells_with_TDP-43-HA_OFF_control
SRR24314001_Neural_culture_cells_with_TDP-43-HA
SRR24314002_Neural_culture_cells_with_TDP-43-HA
SRR24314007_Neural_culture_cells_with_TDP-43-HA
SRR24314008_Neural_culture_cells_with_TDP-43-HA
SRR24314006_Neural_culture_cells_with_NT_shRNA_KD
SRR24314016_Neural_culture_cells_with_NT_shRNA_KD
SRR24314019_Neural_culture_cells_with_NT_shRNA_KD
SRR24314005_Neural_culture_cells_with_TDP-43_shRNA_KD
SRR24314015_Neural_culture_cells_with_TDP-43_shRNA_KD
SRR24314018_Neural_culture_cells_with_TDP-43_shRNA_KD
SRR24314021_Neural_culture_cells_with_TDP-43_shRNA_KD
4491493
4492313
4493321
4494096
chr19:4491492-4494096:+
HDGFL2 | HDGFL2-207
HDGFL2 | HDGFL2-210
HDGFL2 | HDGFL2-208
HDGFL2 | HDGFL2-202
HDGFL2 | HDGFL2-205

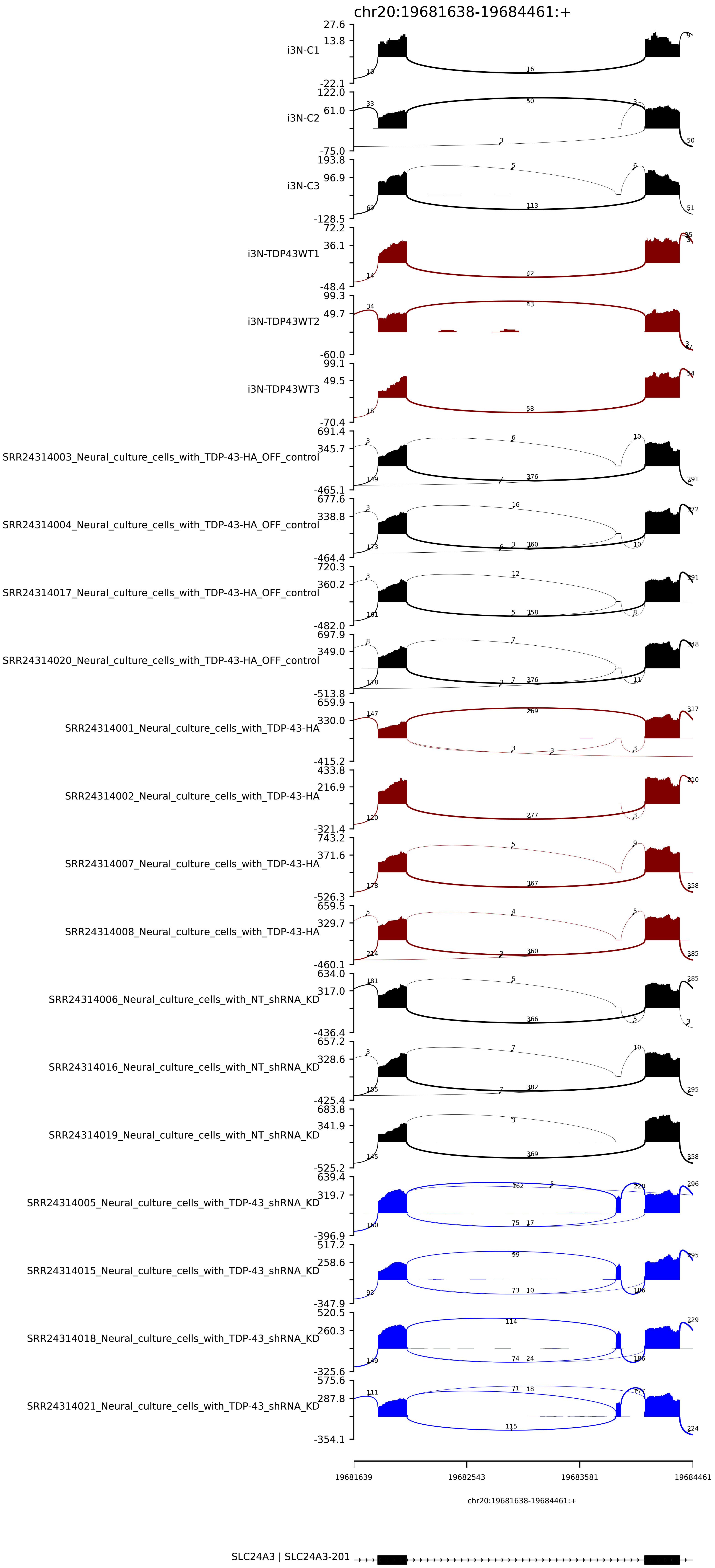
chr20:19681638-19684461:+
i3N-C1
i3N-C2
i3N-C3
i3N-TDP43WT1
i3N-TDP43WT2
i3N-TDP43WT3
SRR24314003_Neural_culture_cells_with_TDP-43-HA_OFF_control
SRR24314004_Neural_culture_cells_with_TDP-43-HA_OFF_control
SRR24314017_Neural_culture_cells_with_TDP-43-HA_OFF_control
SRR24314020_Neural_culture_cells_with_TDP-43-HA_OFF_control
SRR24314001_Neural_culture_cells_with_TDP-43-HA
SRR24314002_Neural_culture_cells_with_TDP-43-HA
SRR24314007_Neural_culture_cells_with_TDP-43-HA
SRR24314008_Neural_culture_cells_with_TDP-43-HA
SRR24314006_Neural_culture_cells_with_NT_shRNA_KD
SRR24314016_Neural_culture_cells_with_NT_shRNA_KD
SRR24314019_Neural_culture_cells_with_NT_shRNA_KD
SRR24314005_Neural_culture_cells_with_TDP-43_shRNA_KD
SRR24314015_Neural_culture_cells_with_TDP-43_shRNA_KD
SRR24314018_Neural_culture_cells_with_TDP-43_shRNA_KD
SRR24314021_Neural_culture_cells_with_TDP-43_shRNA_KD
19681639
19682543
19683581
19684461
chr20:19681638-19684461:+
SLC24A3 | SLC24A3-201

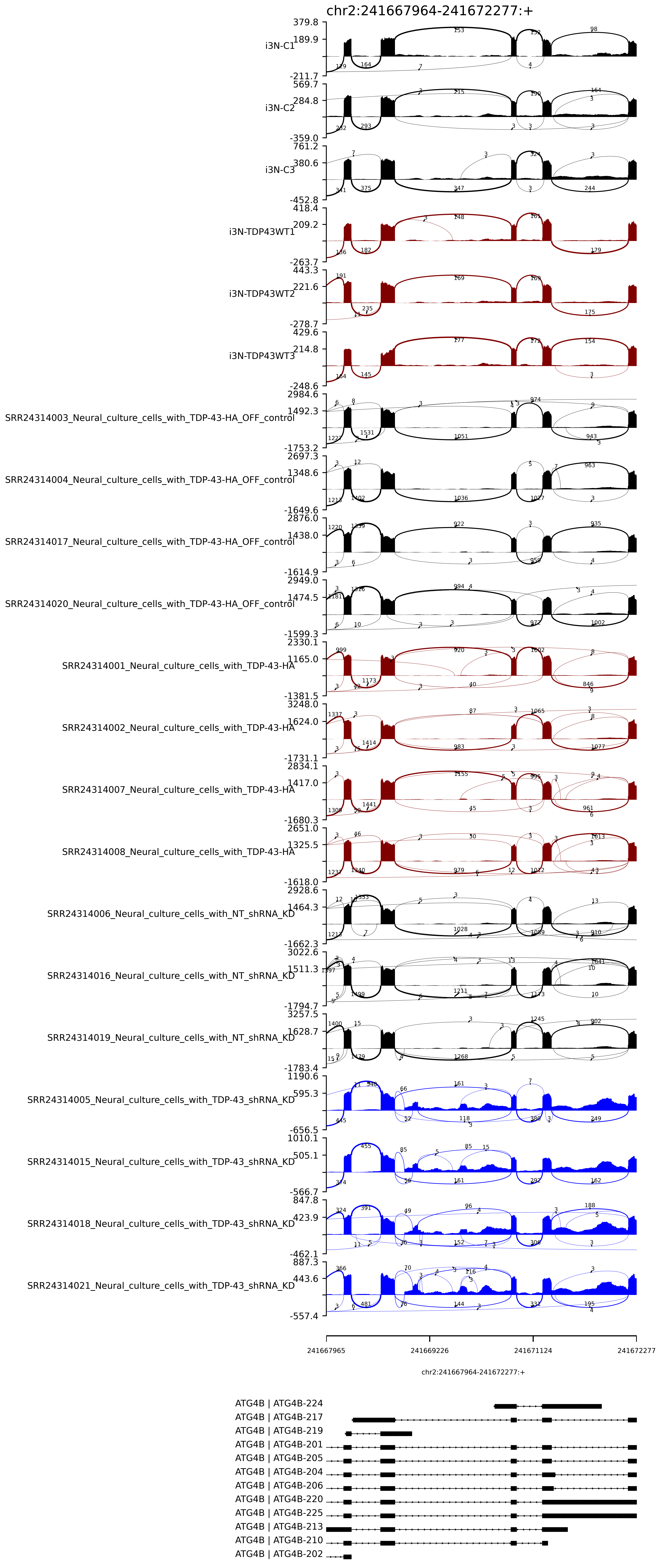

chr2:241667964-241672277:+
i3N-C1
i3N-C2
i3N-C3
i3N-TDP43WT1
i3N-TDP43WT2
i3N-TDP43WT3
SRR24314003_Neural_culture_cells_with_TDP-43-HA_OFF_control
SRR24314004_Neural_culture_cells_with_TDP-43-HA_OFF_control
SRR24314017_Neural_culture_cells_with_TDP-43-HA_OFF_control
SRR24314020_Neural_culture_cells_with_TDP-43-HA_OFF_control
SRR24314001_Neural_culture_cells_with_TDP-43-HA
SRR24314002_Neural_culture_cells_with_TDP-43-HA
SRR24314007_Neural_culture_cells_with_TDP-43-HA
SRR24314008_Neural_culture_cells_with_TDP-43-HA
SRR24314006_Neural_culture_cells_with_NT_shRNA_KD
SRR24314016_Neural_culture_cells_with_NT_shRNA_KD
SRR24314019_Neural_culture_cells_with_NT_shRNA_KD
SRR24314005_Neural_culture_cells_with_TDP-43_shRNA_KD
SRR24314015_Neural_culture_cells_with_TDP-43_shRNA_KD
SRR24314018_Neural_culture_cells_with_TDP-43_shRNA_KD
SRR24314021_Neural_culture_cells_with_TDP-43_shRNA_KD
241667965
241669226
241671124
241672277
chr2:241667964-241672277:+
ATG4B | ATG4B-224
ATG4B | ATG4B-217
ATG4B | ATG4B-219
ATG4B | ATG4B-201
ATG4B | ATG4B-205
ATG4B | ATG4B-204
ATG4B | ATG4B-206
ATG4B | ATG4B-220
ATG4B | ATG4B-225
ATG4B | ATG4B-213
ATG4B | ATG4B-210
ATG4B | ATG4B-202

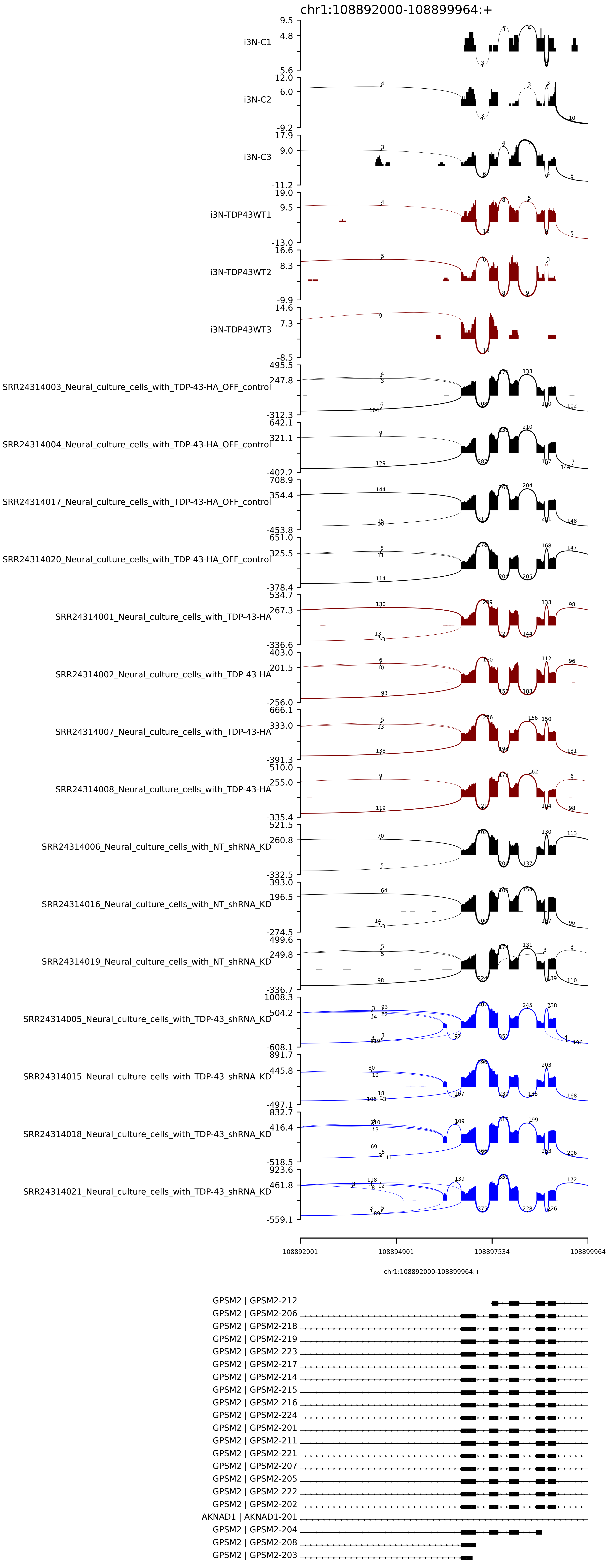
chr1:108892000-108899964:+
i3N-C1
i3N-C2
i3N-C3
i3N-TDP43WT1
i3N-TDP43WT2
i3N-TDP43WT3
SRR24314003_Neural_culture_cells_with_TDP-43-HA_OFF_control
SRR24314004_Neural_culture_cells_with_TDP-43-HA_OFF_control
SRR24314017_Neural_culture_cells_with_TDP-43-HA_OFF_control
SRR24314020_Neural_culture_cells_with_TDP-43-HA_OFF_control
SRR24314001_Neural_culture_cells_with_TDP-43-HA
SRR24314002_Neural_culture_cells_with_TDP-43-HA
SRR24314007_Neural_culture_cells_with_TDP-43-HA
SRR24314008_Neural_culture_cells_with_TDP-43-HA
SRR24314006_Neural_culture_cells_with_NT_shRNA_KD
SRR24314016_Neural_culture_cells_with_NT_shRNA_KD
SRR24314019_Neural_culture_cells_with_NT_shRNA_KD
SRR24314005_Neural_culture_cells_with_TDP-43_shRNA_KD
SRR24314015_Neural_culture_cells_with_TDP-43_shRNA_KD
SRR24314018_Neural_culture_cells_with_TDP-43_shRNA_KD
SRR24314021_Neural_culture_cells_with_TDP-43_shRNA_KD
108892001
108894901
108897534
108899964
chr1:108892000-108899964:+
GPSM2 | GPSM2-212
GPSM2 | GPSM2-206
GPSM2 | GPSM2-218
GPSM2 | GPSM2-219
GPSM2 | GPSM2-223
GPSM2 | GPSM2-217
GPSM2 | GPSM2-214
GPSM2 | GPSM2-215
GPSM2 | GPSM2-216
GPSM2 | GPSM2-224
GPSM2 | GPSM2-201
GPSM2 | GPSM2-211
GPSM2 | GPSM2-221
GPSM2 | GPSM2-207
GPSM2 | GPSM2-205
GPSM2 | GPSM2-222
GPSM2 | GPSM2-202
AKNAD1 | AKNAD1-201
GPSM2 | GPSM2-204
GPSM2 | GPSM2-208
GPSM2 | GPSM2-203

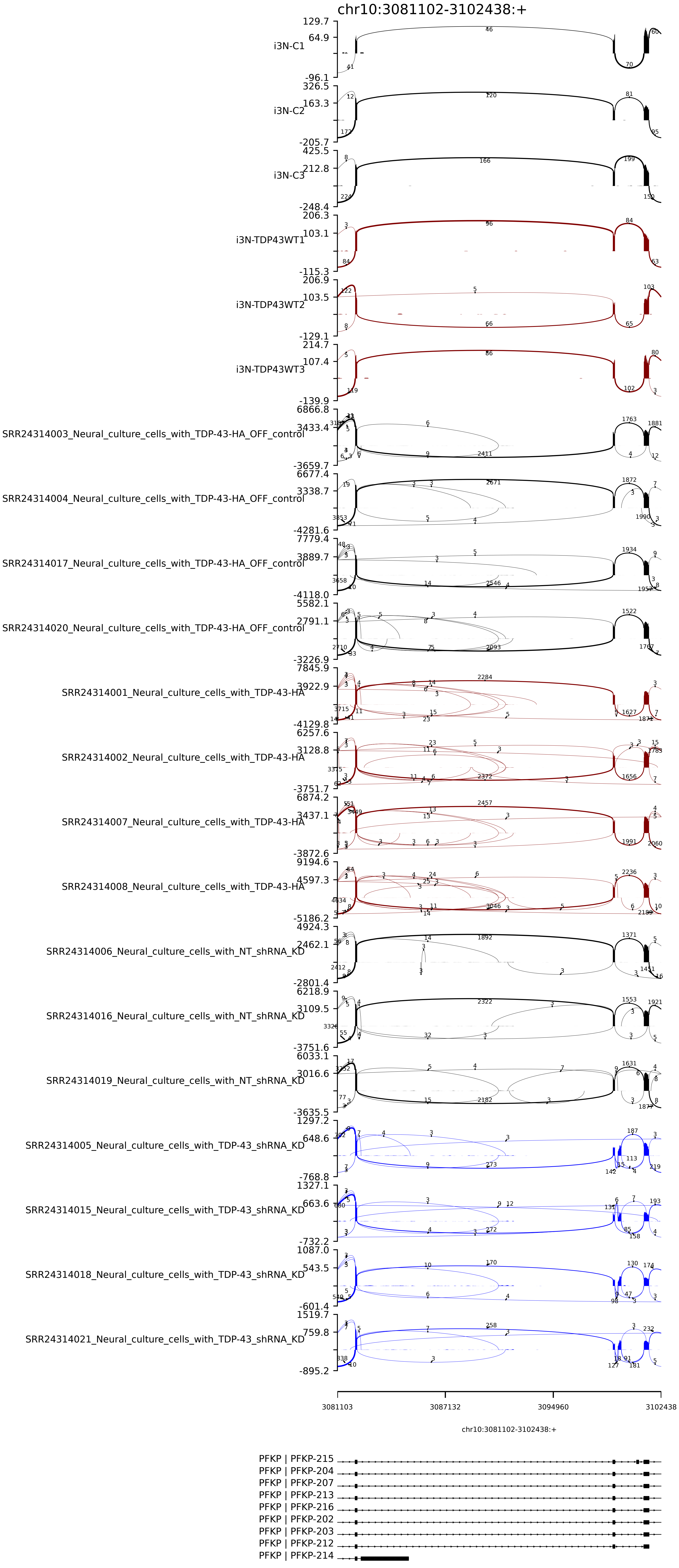
chr10:3081102-3102438:+
i3N-C1
i3N-C2
i3N-C3
i3N-TDP43WT1
i3N-TDP43WT2
i3N-TDP43WT3
SRR24314003_Neural_culture_cells_with_TDP-43-HA_OFF_control
SRR24314004_Neural_culture_cells_with_TDP-43-HA_OFF_control
SRR24314017_Neural_culture_cells_with_TDP-43-HA_OFF_control
SRR24314020_Neural_culture_cells_with_TDP-43-HA_OFF_control
SRR24314001_Neural_culture_cells_with_TDP-43-HA
SRR24314002_Neural_culture_cells_with_TDP-43-HA
SRR24314007_Neural_culture_cells_with_TDP-43-HA
SRR24314008_Neural_culture_cells_with_TDP-43-HA
SRR24314006_Neural_culture_cells_with_NT_shRNA_KD
SRR24314016_Neural_culture_cells_with_NT_shRNA_KD
SRR24314019_Neural_culture_cells_with_NT_shRNA_KD
SRR24314005_Neural_culture_cells_with_TDP-43_shRNA_KD
SRR24314015_Neural_culture_cells_with_TDP-43_shRNA_KD
SRR24314018_Neural_culture_cells_with_TDP-43_shRNA_KD
SRR24314021_Neural_culture_cells_with_TDP-43_shRNA_KD
3081103
3087132
3094960
3102438
chr10:3081102-3102438:+
PFKP | PFKP-215
PFKP | PFKP-204
PFKP | PFKP-207
PFKP | PFKP-213
PFKP | PFKP-216
PFKP | PFKP-202
PFKP | PFKP-203
PFKP | PFKP-212
PFKP | PFKP-214
