# Supplementary material for: Elevated nuclear TDP-43 induces constitutive exon skipping
Source: Mol Neurodegener. 2024 Jun 9;19:45. doi: 10.1186/s13024-024-00732-w (PMC11163724; doi:10.1186/s13024-024-00732-w)
Supplement: Supplementary file 8 — Supplementary Material 8 [file 13024_2024_732_MOESM8_ESM.pdf]

Gene	Coordinates	Splicing Type	Data
BMPR1A	chr10:86921303-86924982	skiptic exon	Carmen-Orozco, et al. 2023
CANX	chr5:179698416-179706991	skiptic exon	Carmen-Orozco, et al. 2023
COQ5	chr12:120516319-120526842	skiptic exon	Carmen-Orozco, et al. 2023
ELP2	chr18:36166598-36171437	skiptic exon	Carmen-Orozco, et al. 2023
HYOU1	chr11:119052044-119052857	skiptic exon	Carmen-Orozco, et al. 2023
MYBBP1A	chr17:4544952-4545777	skiptic exon	Carmen-Orozco, et al. 2023
NUP93	chr16:56838753-56841937	skiptic exon	Carmen-Orozco, et al. 2023
SCN9A	chr2:166272081-166280656	skiptic exon	Carmen-Orozco, et al. 2023
SESN3	chr11:95185056-95191820	skiptic exon	Carmen-Orozco, et al. 2023
SLC35A5	chr3:112562822-112571073	skiptic exon	Carmen-Orozco, et al. 2023
TESK1	chr9:35606765-35607748	skiptic exon	Carmen-Orozco, et al. 2023
VARS2	chr6:30916103-30917282	skiptic exon	Carmen-Orozco, et al. 2023
WSCD1	chr17:6080025-6095711	skiptic exon	Carmen-Orozco, et al. 2023
XPNPEP1	chr10:109883737-109888276	skiptic exon	Carmen-Orozco, et al. 2023
DDI2	chr1:15651731-15662471	skiptic exon	Carmen-Orozco, et al. 2023
PLOD1	chr1:11965458-11967116	skiptic exon	Fratta, et al. 2018
SLC6A6	chr3:14467812-14472314	skiptic exon	Fratta, et al. 2018
ACTL6B	chr7:100649782-100655263	cryptic exon	Irwin, et al. 2024
AGRN	chr1:1044046-1045563	cryptic exon	Irwin, et al. 2025
EPB41L4A	chr5:112265962-112275610	cryptic exon	Irwin, et al. 2026
HDGFL2	chr19:4491492-4494096	cryptic exon	Irwin, et al. 2027
SLC24A3	chr20:19681638-19684461	cryptic exon	Irwin, et al. 2028
STMN2	chr8:79610163-79637892	cryptic exon	Klim, et al. 2019, Melamed et al. 2019
UNC13A	chr19:17641397-17642959	cryptic exon	Rosa Ma, et al. 2022
ATG4B	chr2:241667964-241672277	cryptic exon	Ling, et al. 2015
GPSM2	chr1:108892000-108899964	cryptic exon	Ling, et al. 2015
PFKP	chr10:3081102-3102438	cryptic exon	Ling, et al. 2015

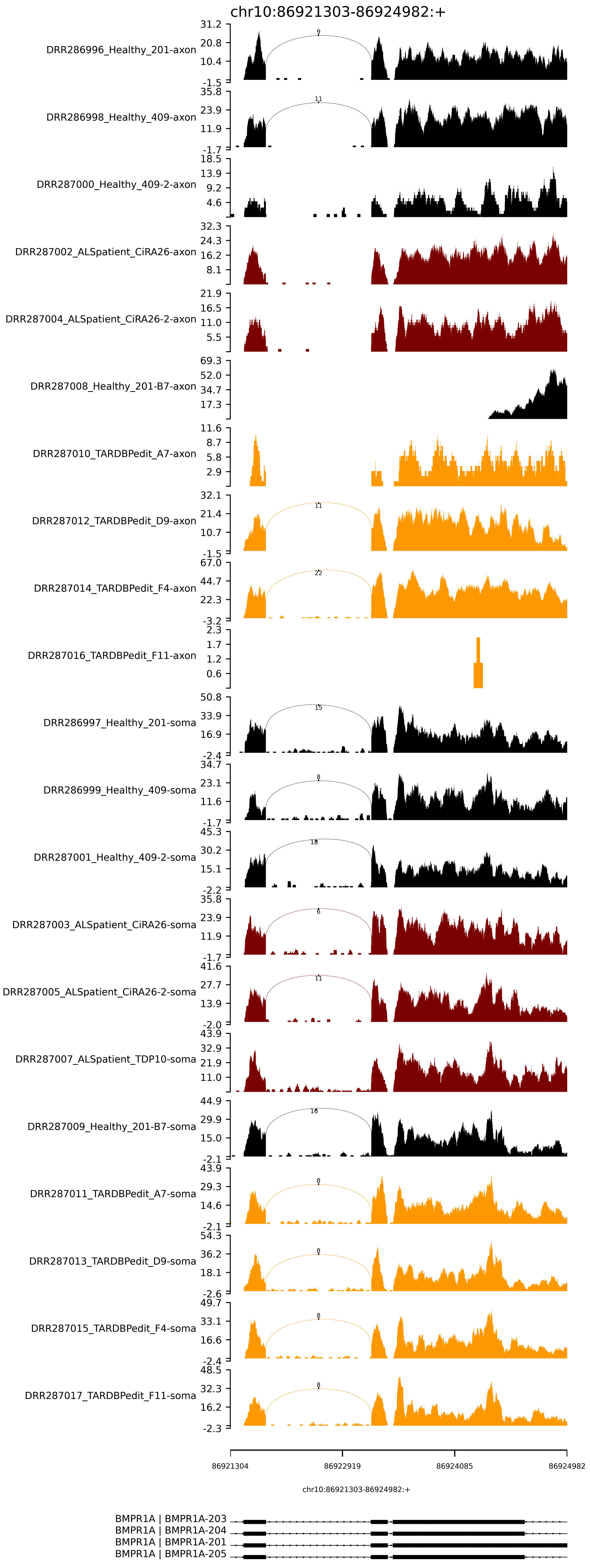
chr10:86921303-86924982:+
DRR286996_Healthy_201-axon
DRR286998_Healthy_409-axon
DRR287000_Healthy_409-2-axon
DRR287002_ALSpatient_CiRA26-axon
DRR287004_ALSpatient_CiRA26-2-axon
DRR287008_Healthy_201-B7-axon
DRR287010_TARDBPedit_A7-axon
DRR287012_TARDBPedit_D9-axon
DRR287014_TARDBPedit_F4-axon
DRR287016_TARDBPedit_F11-axon
DRR286997_Healthy_201-soma
DRR286999_Healthy_409-soma
DRR287001_Healthy_409-2-soma
DRR287003_ALSpatient_CiRA26-soma
DRR287005_ALSpatient_CiRA26-2-soma
DRR287007_ALSpatient_TDP10-soma
DRR287009_Healthy_201-B7-soma
DRR287011_TARDBPedit_A7-soma
DRR287013_TARDBPedit_D9-soma
DRR287015_TARDBPedit_F4-soma
DRR287017_TARDBPedit_F11-soma
86921304
86922919
86924085
86924982
chr10:86921303-86924982:+
BMPR1A | BMPR1A-203
BMPR1A | BMPR1A-204
BMPR1A | BMPR1A-201
BMPR1A | BMPR1A-205

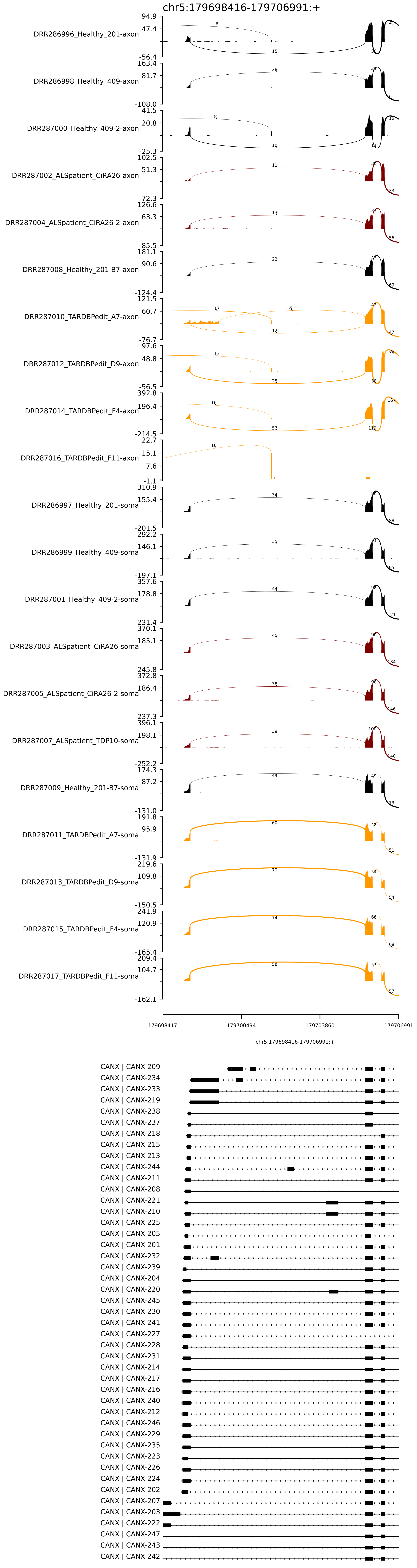
chr5:179698416-179706991:+
DRR286996_Healthy_201-axon
94.9
47.4
-56.4
DRR286998_Healthy_409-axon
163.4
81.7
-108.0
DRR287000_Healthy_409-2-axon
41.5
20.8
-25.3
DRR287002_ALSpatient_CiRA26-axon
102.5
51.3
-72.3
DRR287004_ALSpatient_CiRA26-2-axon
126.6
63.3
-85.5
DRR287008_Healthy_201-B7-axon
181.1
90.6
-124.4
DRR287010_TARDBPedit_A7-axon
121.5
60.7
-76.7
DRR287012_TARDBPedit_D9-axon
97.6
48.8
-56.5
DRR287014_TARDBPedit_F4-axon
392.8
196.4
-214.5
DRR287016_TARDBPedit_F11-axon
22.7
15.1
7.6
-1.1
DRR286997_Healthy_201-soma
310.9
155.4
-201.5
DRR286999_Healthy_409-soma
292.2
146.1
-197.1
DRR287001_Healthy_409-2-soma
357.6
178.8
-231.4
DRR287003_ALSpatient_CiRA26-soma
370.1
185.1
-245.8
DRR287005_ALSpatient_CiRA26-2-soma
372.8
186.4
-237.3
DRR287007_ALSpatient_TDP10-soma
396.1
198.1
-252.2
DRR287009_Healthy_201-B7-soma
174.3
87.2
-131.0
DRR287011_TARDBPedit_A7-soma
191.8
95.9
-131.9
DRR287013_TARDBPedit_D9-soma
219.6
109.8
-150.5
DRR287015_TARDBPedit_F4-soma
241.9
120.9
-165.4
DRR287017_TARDBPedit_F11-soma
209.4
104.7
-162.1
179698417
179700494
179703860
179706991
chr5:179698416-179706991:+
CANX | CANX-209
CANX | CANX-234
CANX | CANX-233
CANX | CANX-219
CANX | CANX-238
CANX | CANX-237
CANX | CANX-218
CANX | CANX-215
CANX | CANX-213
CANX | CANX-244
CANX | CANX-211
CANX | CANX-208
CANX | CANX-221
CANX | CANX-210
CANX | CANX-225
CANX | CANX-205
CANX | CANX-201
CANX | CANX-232
CANX | CANX-239
CANX | CANX-204
CANX | CANX-220
CANX | CANX-245
CANX | CANX-230
CANX | CANX-241
CANX | CANX-227
CANX | CANX-228
CANX | CANX-231
CANX | CANX-214
CANX | CANX-217
CANX | CANX-216
CANX | CANX-240
CANX | CANX-212
CANX | CANX-246
CANX | CANX-229
CANX | CANX-235
CANX | CANX-223
CANX | CANX-226
CANX | CANX-224
CANX | CANX-202
CANX | CANX-207
CANX | CANX-203
CANX | CANX-222
CANX | CANX-247
CANX | CANX-243
CANX | CANX-242

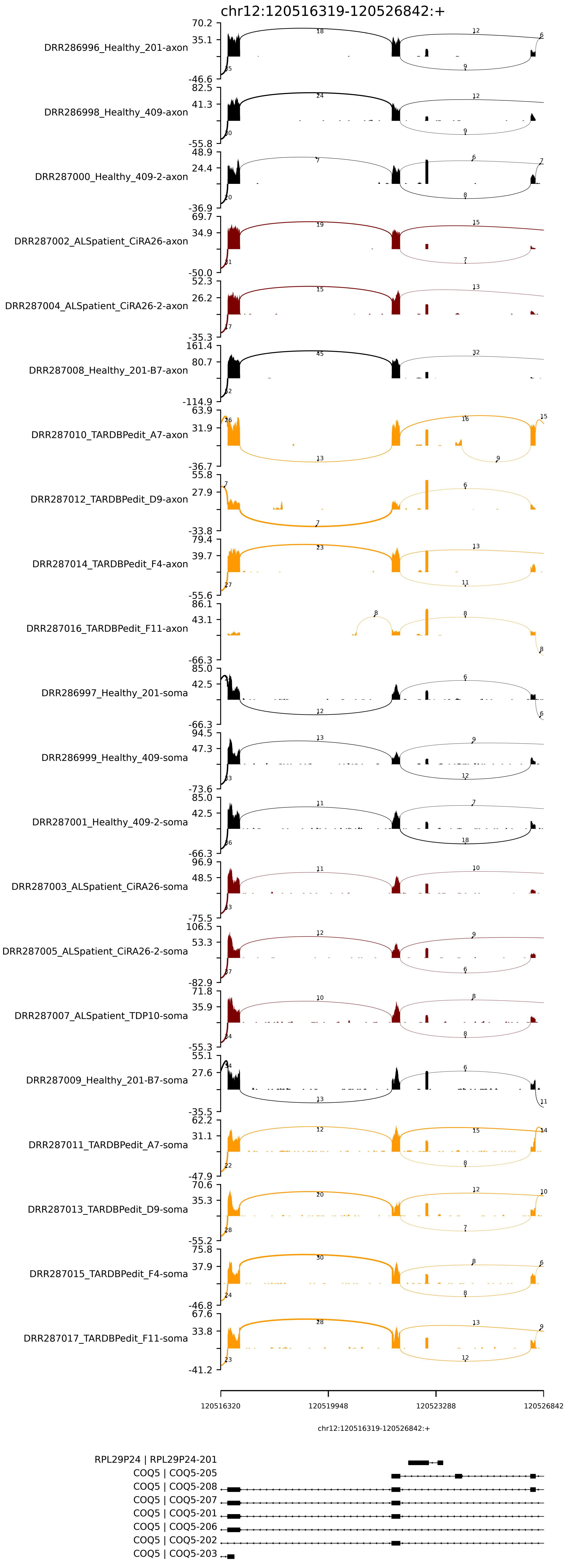
chr12:120516319-120526842:+
DRR286996_Healthy_201-axon
DRR286998_Healthy_409-axon
DRR287000_Healthy_409-2-axon
DRR287002_ALSpatient_CiRA26-axon
DRR287004_ALSpatient_CiRA26-2-axon
DRR287008_Healthy_201-B7-axon
DRR287010_TARDBPedit_A7-axon
DRR287012_TARDBPedit_D9-axon
DRR287014_TARDBPedit_F4-axon
DRR287016_TARDBPedit_F11-axon
DRR286997_Healthy_201-soma
DRR286999_Healthy_409-soma
DRR287001_Healthy_409-2-soma
DRR287003_ALSpatient_CiRA26-soma
DRR287005_ALSpatient_CiRA26-2-soma
DRR287007_ALSpatient_TDP10-soma
DRR287009_Healthy_201-B7-soma
DRR287011_TARDBPedit_A7-soma
DRR287013_TARDBPedit_D9-soma
DRR287015_TARDBPedit_F4-soma
DRR287017_TARDBPedit_F11-soma
120516320
120519948
120523288
120526842
chr12:120516319-120526842:+
RPL29P24 | RPL29P24-201
COQ5 | COQ5-205
COQ5 | COQ5-208
COQ5 | COQ5-207
COQ5 | COQ5-201
COQ5 | COQ5-206
COQ5 | COQ5-202
COQ5 | COQ5-203

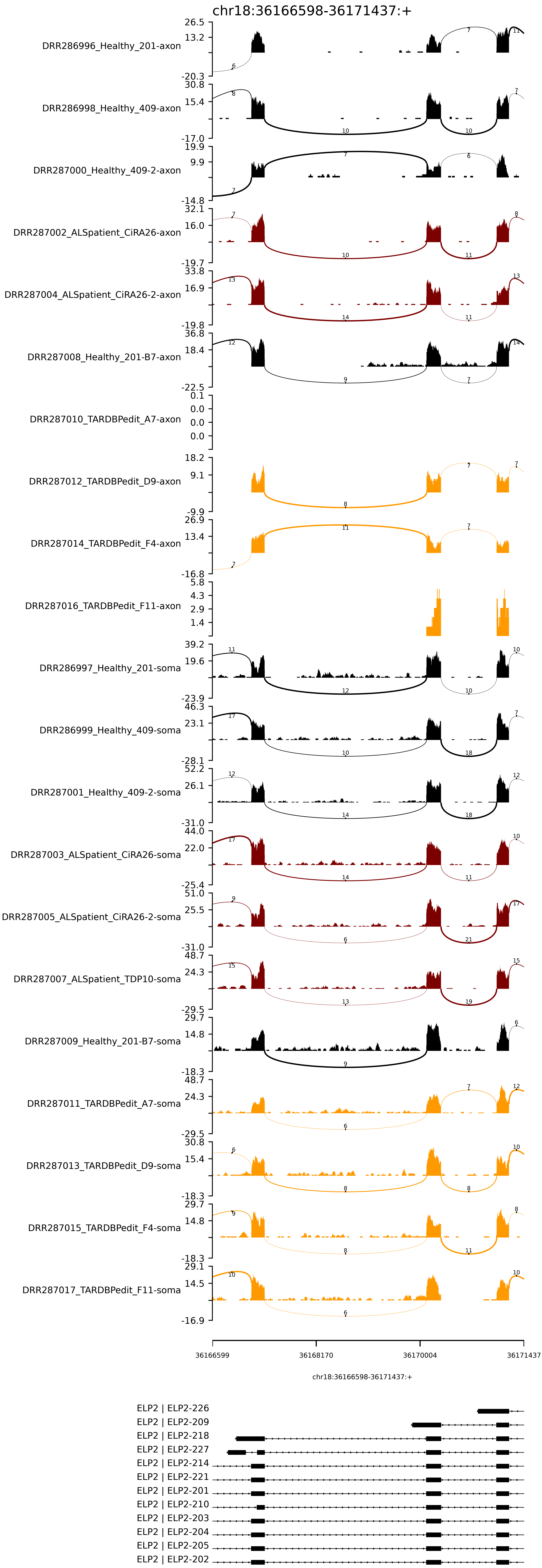
chr18:36166598-36171437:+
DRR286996_Healthy_201-axon
DRR286998_Healthy_409-axon
DRR287000_Healthy_409-2-axon
DRR287002_ALSpatient_CiRA26-axon
DRR287004_ALSpatient_CiRA26-2-axon
DRR287008_Healthy_201-B7-axon
DRR287010_TARDBPedit_A7-axon
DRR287012_TARDBPedit_D9-axon
DRR287014_TARDBPedit_F4-axon
DRR287016_TARDBPedit_F11-axon
DRR286997_Healthy_201-soma
DRR286999_Healthy_409-soma
DRR287001_Healthy_409-2-soma
DRR287003_ALSpatient_CiRA26-soma
DRR287005_ALSpatient_CiRA26-2-soma
DRR287007_ALSpatient_TDP10-soma
DRR287009_Healthy_201-B7-soma
DRR287011_TARDBPedit_A7-soma
DRR287013_TARDBPedit_D9-soma
DRR287015_TARDBPedit_F4-soma
DRR287017_TARDBPedit_F11-soma
36166599
36168170
36170004
36171437
chr18:36166598-36171437:+
ELP2 | ELP2-226
ELP2 | ELP2-209
ELP2 | ELP2-218
ELP2 | ELP2-227
ELP2 | ELP2-214
ELP2 | ELP2-221
ELP2 | ELP2-201
ELP2 | ELP2-210
ELP2 | ELP2-203
ELP2 | ELP2-204
ELP2 | ELP2-205
ELP2 | ELP2-202

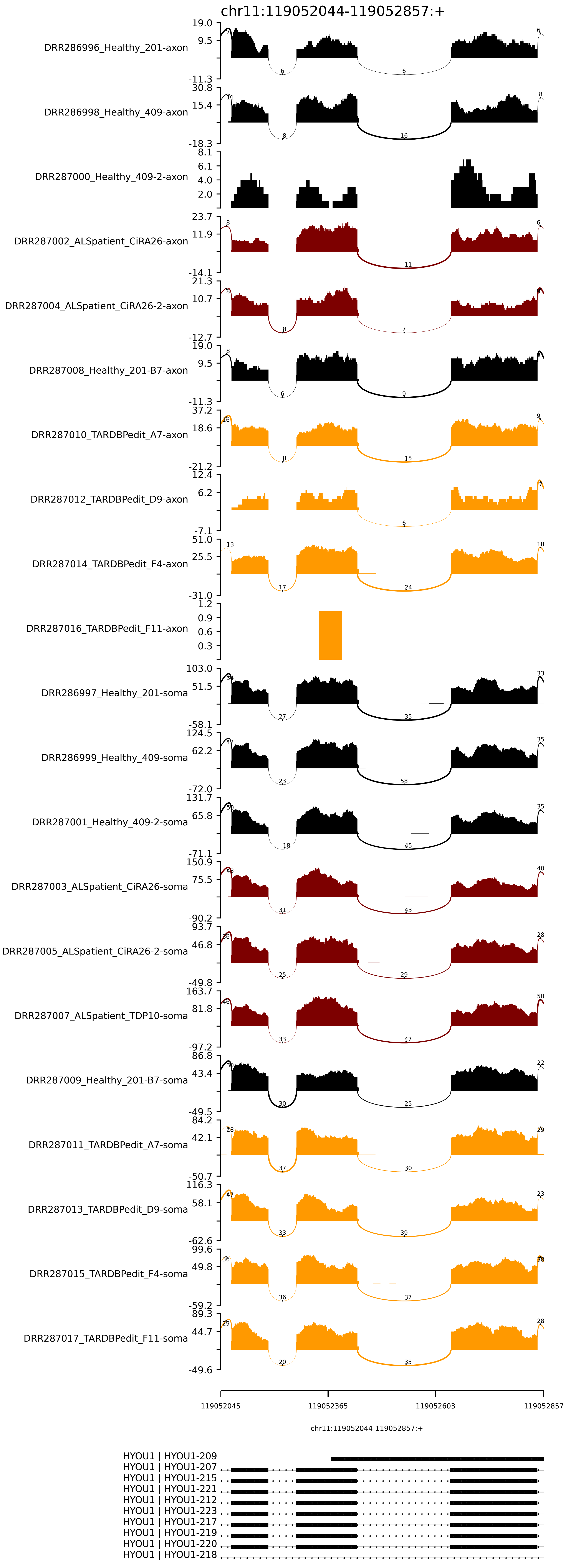
chr11:119052044-119052857:+
DRR286996_Healthy_201-axon
DRR286998_Healthy_409-axon
DRR287000_Healthy_409-2-axon
DRR287002_ALSpatient_CiRA26-axon
DRR287004_ALSpatient_CiRA26-2-axon
DRR287008_Healthy_201-B7-axon
DRR287010_TARDBPedit_A7-axon
DRR287012_TARDBPedit_D9-axon
DRR287014_TARDBPedit_F4-axon
DRR287016_TARDBPedit_F11-axon
DRR286997_Healthy_201-soma
DRR286999_Healthy_409-soma
DRR287001_Healthy_409-2-soma
DRR287003_ALSpatient_CiRA26-soma
DRR287005_ALSpatient_CiRA26-2-soma
DRR287007_ALSpatient_TDP10-soma
DRR287009_Healthy_201-B7-soma
DRR287011_TARDBPedit_A7-soma
DRR287013_TARDBPedit_D9-soma
DRR287015_TARDBPedit_F4-soma
DRR287017_TARDBPedit_F11-soma
119052045
119052365
119052603
119052857
chr11:119052044-119052857:+
HYOU1 | HYOU1-209
HYOU1 | HYOU1-207
HYOU1 | HYOU1-215
HYOU1 | HYOU1-221
HYOU1 | HYOU1-212
HYOU1 | HYOU1-223
HYOU1 | HYOU1-217
HYOU1 | HYOU1-219
HYOU1 | HYOU1-220
HYOU1 | HYOU1-218

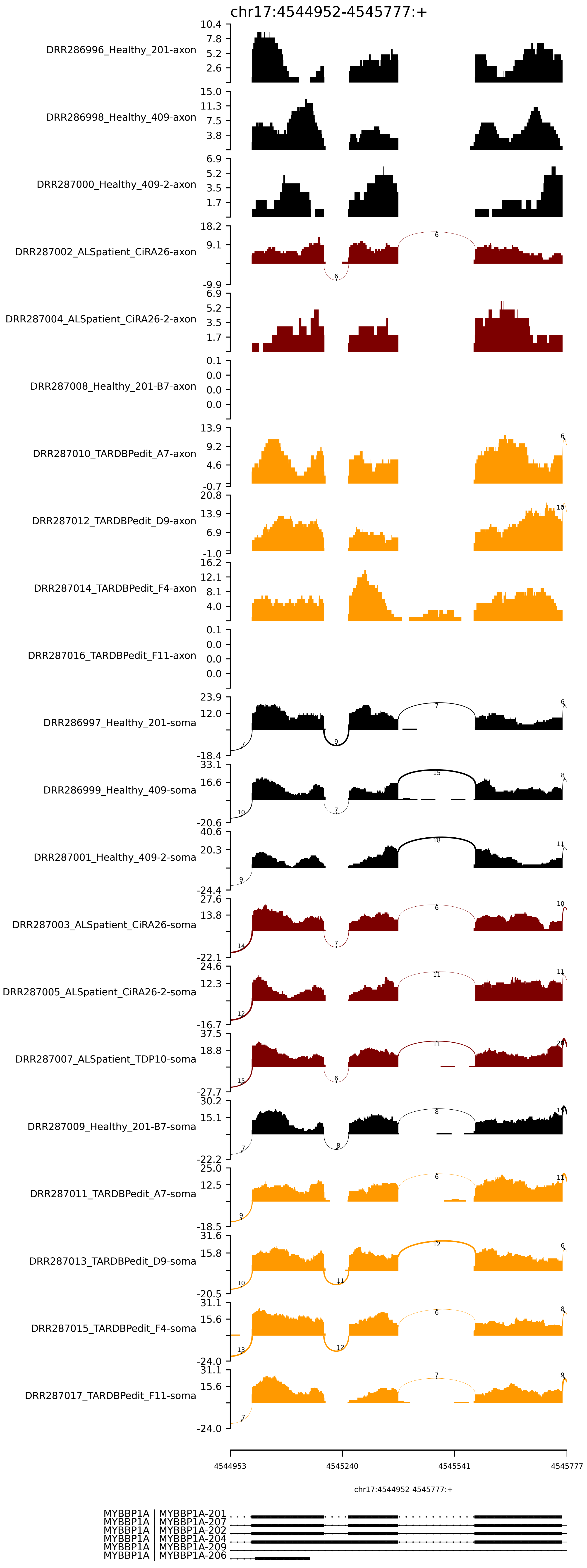
chr17:4544952-4545777:+
DRR286996_Healthy_201-axon
DRR286998_Healthy_409-axon
DRR287000_Healthy_409-2-axon
DRR287002_ALSpatient_CiRA26-axon
DRR287004_ALSpatient_CiRA26-2-axon
DRR287008_Healthy_201-B7-axon
DRR287010_TARDBPedit_A7-axon
DRR287012_TARDBPedit_D9-axon
DRR287014_TARDBPedit_F4-axon
DRR287016_TARDBPedit_F11-axon
DRR286997_Healthy_201-soma
DRR286999_Healthy_409-soma
DRR287001_Healthy_409-2-soma
DRR287003_ALSpatient_CiRA26-soma
DRR287005_ALSpatient_CiRA26-2-soma
DRR287007_ALSpatient_TDP10-soma
DRR287009_Healthy_201-B7-soma
DRR287011_TARDBPedit_A7-soma
DRR287013_TARDBPedit_D9-soma
DRR287015_TARDBPedit_F4-soma
DRR287017_TARDBPedit_F11-soma
4544953
4545240
4545541
4545777
chr17:4544952-4545777:+
MYBBP1A | MYBBP1A-201
MYBBP1A | MYBBP1A-207
MYBBP1A | MYBBP1A-202
MYBBP1A | MYBBP1A-204
MYBBP1A | MYBBP1A-209
MYBBP1A | MYBBP1A-206

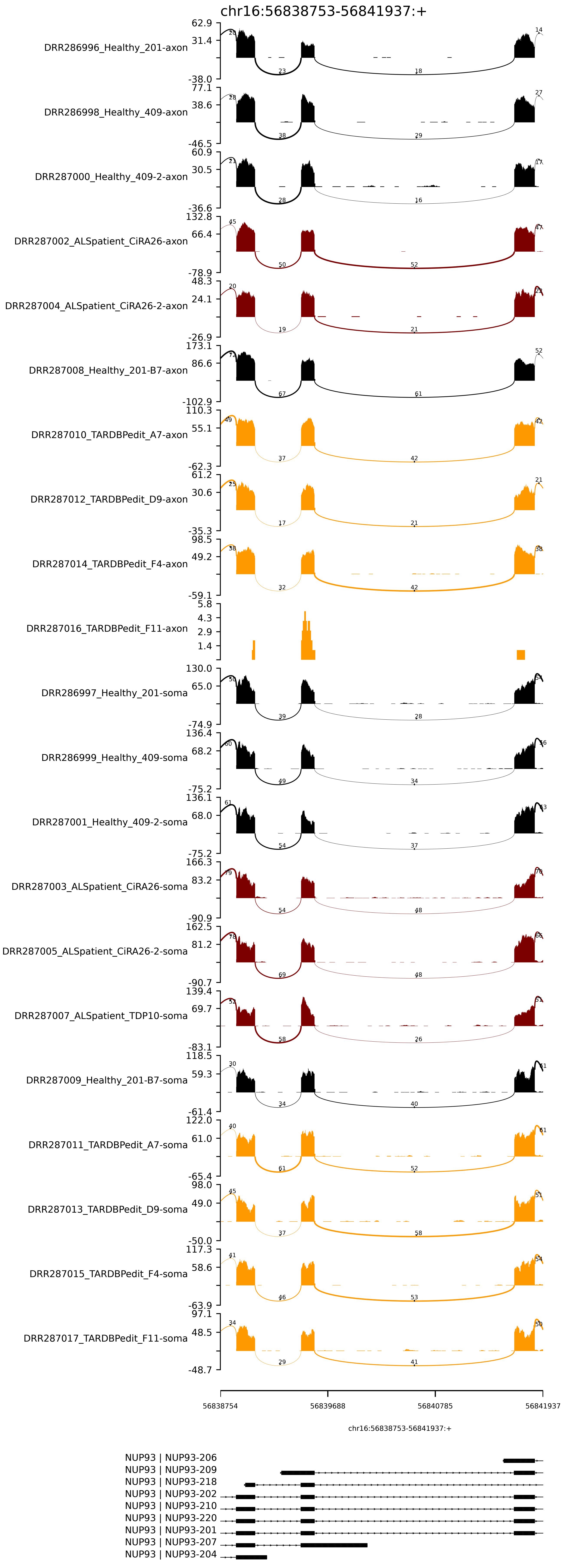
chr16:56838753-56841937:+
DRR286996_Healthy_201-axon
DRR286998_Healthy_409-axon
DRR287000_Healthy_409-2-axon
DRR287002_ALSpatient_CiRA26-axon
DRR287004_ALSpatient_CiRA26-2-axon
DRR287008_Healthy_201-B7-axon
DRR287010_TARDBPedit_A7-axon
DRR287012_TARDBPedit_D9-axon
DRR287014_TARDBPedit_F4-axon
DRR287016_TARDBPedit_F11-axon
DRR286997_Healthy_201-soma
DRR286999_Healthy_409-soma
DRR287001_Healthy_409-2-soma
DRR287003_ALSpatient_CiRA26-soma
DRR287005_ALSpatient_CiRA26-2-soma
DRR287007_ALSpatient_TDP10-soma
DRR287009_Healthy_201-B7-soma
DRR287011_TARDBPedit_A7-soma
DRR287013_TARDBPedit_D9-soma
DRR287015_TARDBPedit_F4-soma
DRR287017_TARDBPedit_F11-soma
56838754
56839688
56840785
56841937
chr16:56838753-56841937:+
NUP93 | NUP93-206
NUP93 | NUP93-209
NUP93 | NUP93-218
NUP93 | NUP93-202
NUP93 | NUP93-210
NUP93 | NUP93-220
NUP93 | NUP93-201
NUP93 | NUP93-207
NUP93 | NUP93-204

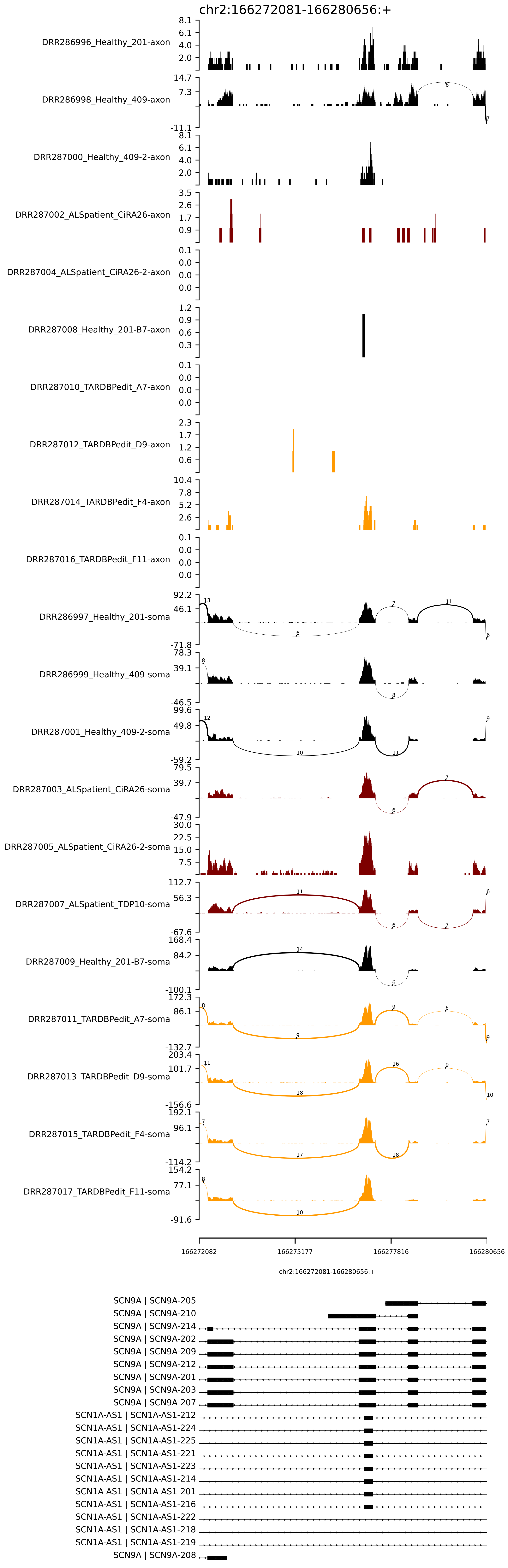
chr2:166272081-166280656:+
DRR286996_Healthy_201-axon
DRR286998_Healthy_409-axon
DRR287000_Healthy_409-2-axon
DRR287002_ALSpatient_CiRA26-axon
DRR287004_ALSpatient_CiRA26-2-axon
DRR287008_Healthy_201-B7-axon
DRR287010_TARDBPedit_A7-axon
DRR287012_TARDBPedit_D9-axon
DRR287014_TARDBPedit_F4-axon
DRR287016_TARDBPedit_F11-axon
DRR286997_Healthy_201-soma
DRR286999_Healthy_409-soma
DRR287001_Healthy_409-2-soma
DRR287003_ALSpatient_CiRA26-soma
DRR287005_ALSpatient_CiRA26-2-soma
DRR287007_ALSpatient_TDP10-soma
DRR287009_Healthy_201-B7-soma
DRR287011_TARDBPedit_A7-soma
DRR287013_TARDBPedit_D9-soma
DRR287015_TARDBPedit_F4-soma
DRR287017_TARDBPedit_F11-soma
166272082
166275177
166277816
166280656
chr2:166272081-166280656:+
SCN9A | SCN9A-205
SCN9A | SCN9A-210
SCN9A | SCN9A-214
SCN9A | SCN9A-202
SCN9A | SCN9A-209
SCN9A | SCN9A-212
SCN9A | SCN9A-201
SCN9A | SCN9A-203
SCN9A | SCN9A-207
SCN1A-AS1 | SCN1A-AS1-212
SCN1A-AS1 | SCN1A-AS1-224
SCN1A-AS1 | SCN1A-AS1-225
SCN1A-AS1 | SCN1A-AS1-221
SCN1A-AS1 | SCN1A-AS1-223
SCN1A-AS1 | SCN1A-AS1-214
SCN1A-AS1 | SCN1A-AS1-201
SCN1A-AS1 | SCN1A-AS1-216
SCN1A-AS1 | SCN1A-AS1-222
SCN1A-AS1 | SCN1A-AS1-218
SCN1A-AS1 | SCN1A-AS1-219
SCN9A | SCN9A-208

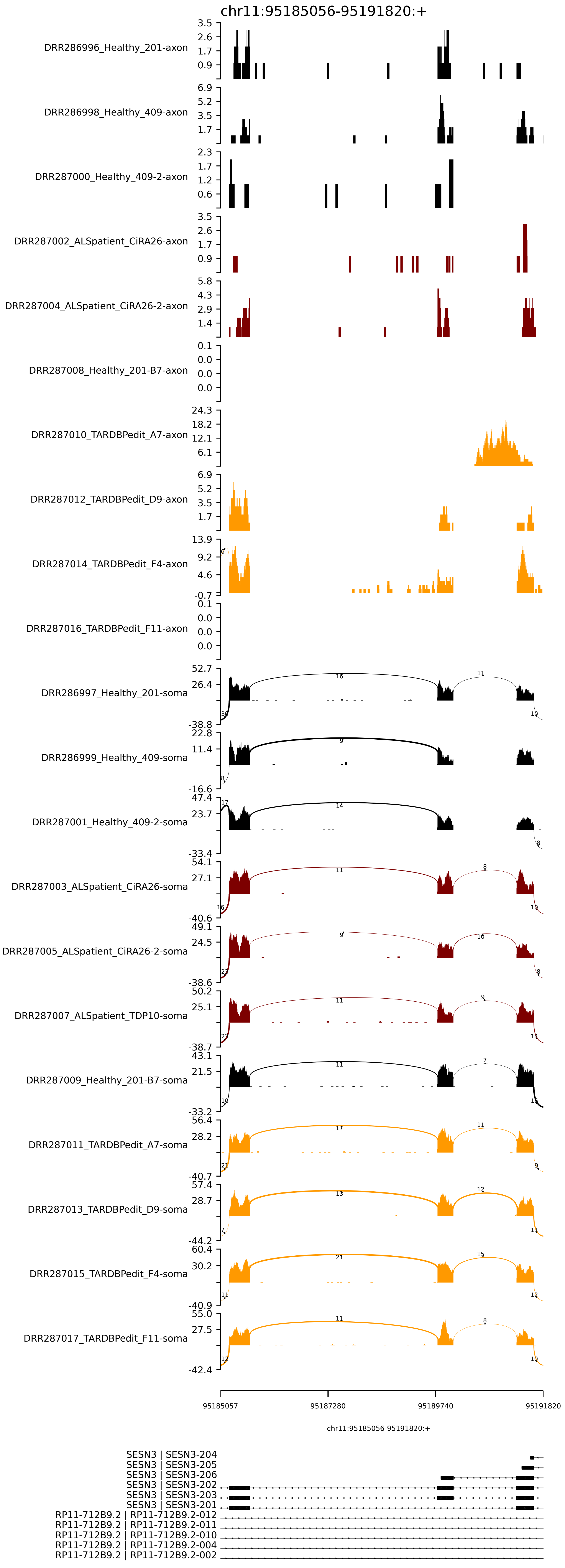

chr11:95185056-95191820:+
DRR286996_Healthy_201-axon
DRR286998_Healthy_409-axon
DRR287000_Healthy_409-2-axon
DRR287002_ALSpatient_CiRA26-axon
DRR287004_ALSpatient_CiRA26-2-axon
DRR287008_Healthy_201-B7-axon
DRR287010_TARDBPedit_A7-axon
DRR287012_TARDBPedit_D9-axon
DRR287014_TARDBPedit_F4-axon
DRR287016_TARDBPedit_F11-axon
DRR286997_Healthy_201-soma
DRR286999_Healthy_409-soma
DRR287001_Healthy_409-2-soma
DRR287003_ALSpatient_CiRA26-soma
DRR287005_ALSpatient_CiRA26-2-soma
DRR287007_ALSpatient_TDP10-soma
DRR287009_Healthy_201-B7-soma
DRR287011_TARDBPedit_A7-soma
DRR287013_TARDBPedit_D9-soma
DRR287015_TARDBPedit_F4-soma
DRR287017_TARDBPedit_F11-soma
95185057
95187280
95189740
95191820
chr11:95185056-95191820:+
SESN3 | SESN3-204
SESN3 | SESN3-205
SESN3 | SESN3-206
SESN3 | SESN3-202
SESN3 | SESN3-203
SESN3 | SESN3-201
RP11-712B9.2 | RP11-712B9.2-012
RP11-712B9.2 | RP11-712B9.2-011
RP11-712B9.2 | RP11-712B9.2-010
RP11-712B9.2 | RP11-712B9.2-004
RP11-712B9.2 | RP11-712B9.2-002

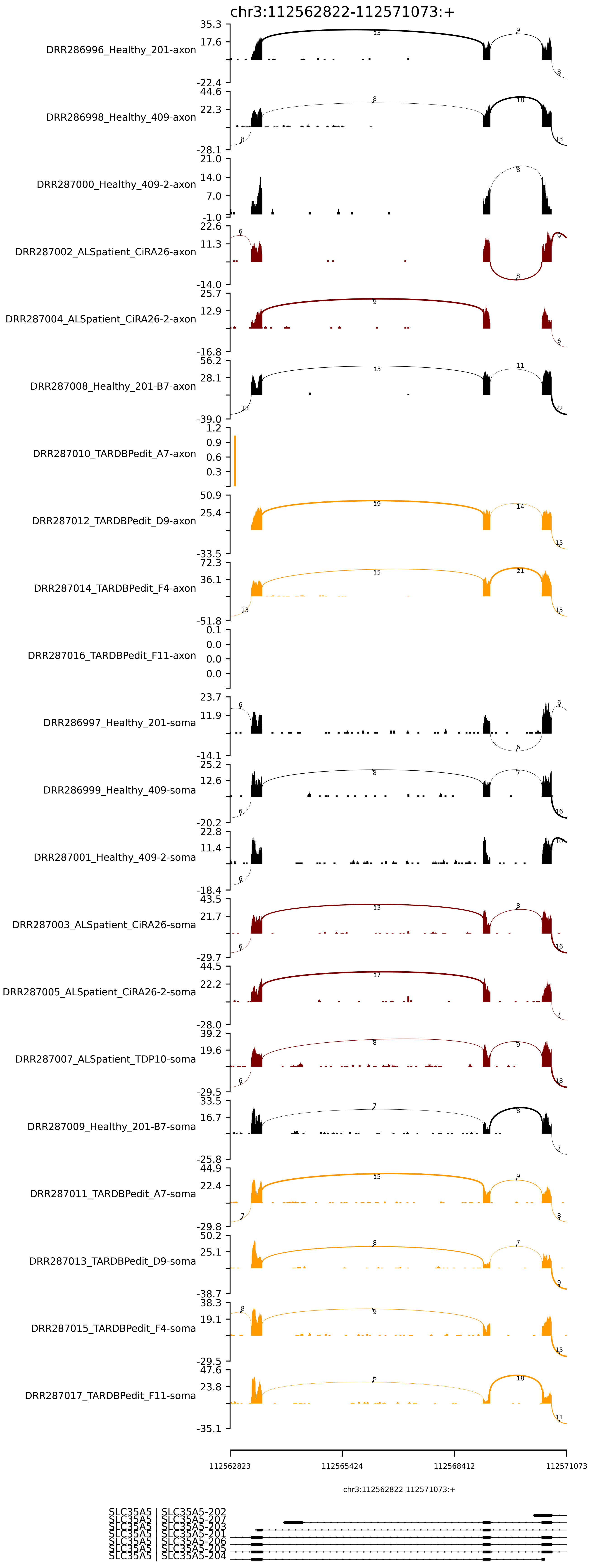
chr3:112562822-112571073:+
35.3
17.6
-22.4
DRR286996_Healthy_201-axon
44.6
22.3
-28.1
DRR286998_Healthy_409-axon
21.0
14.0
7.0
-1.0
DRR287000_Healthy_409-2-axon
22.6
11.3
-14.0
DRR287002_ALSpatient_CiRA26-axon
25.7
12.9
-16.8
DRR287004_ALSpatient_CiRA26-2-axon
56.2
28.1
-39.0
DRR287008_Healthy_201-B7-axon
1.2
0.9
0.6
0.3
DRR287010_TARDBPedit_A7-axon
50.9
25.4
-33.5
DRR287012_TARDBPedit_D9-axon
72.3
36.1
-51.8
DRR287014_TARDBPedit_F4-axon
0.1
0.0
0.0
0.0
DRR287016_TARDBPedit_F11-axon
23.7
11.9
-14.1
DRR286997_Healthy_201-soma
25.2
12.6
-20.2
DRR286999_Healthy_409-soma
22.8
11.4
-18.4
DRR287001_Healthy_409-2-soma
43.5
21.7
-29.7
DRR287003_ALSpatient_CiRA26-soma
44.5
22.2
-28.0
DRR287005_ALSpatient_CiRA26-2-soma
39.2
19.6
-29.5
DRR287007_ALSpatient_TDP10-soma
33.5
16.7
-25.8
DRR287009_Healthy_201-B7-soma
44.9
22.4
-29.8
DRR287011_TARDBPedit_A7-soma
50.2
25.1
-38.7
DRR287013_TARDBPedit_D9-soma
38.3
19.1
-29.5
DRR287015_TARDBPedit_F4-soma
47.6
23.8
-35.1
DRR287017_TARDBPedit_F11-soma
112562823
112565424
112568412
112571073
chr3:112562822-112571073:+
SLC35A5 | SLC35A5-202
SLC35A5 | SLC35A5-207
SLC35A5 | SLC35A5-203
SLC35A5 | SLC35A5-201
SLC35A5 | SLC35A5-206
SLC35A5 | SLC35A5-205
SLC35A5 | SLC35A5-204

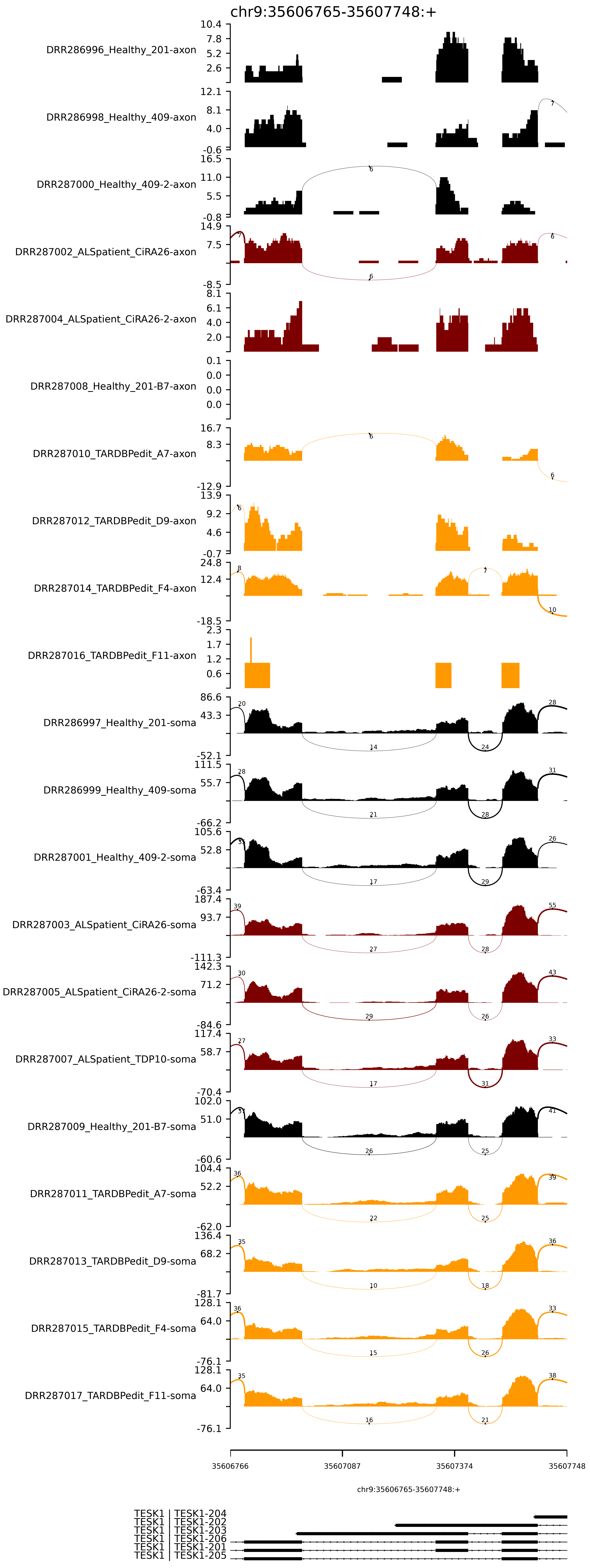
chr9:35606765-35607748:+
DRR286996_Healthy_201-axon
DRR286998_Healthy_409-axon
DRR287000_Healthy_409-2-axon
DRR287002_ALSpatient_CiRA26-axon
DRR287004_ALSpatient_CiRA26-2-axon
DRR287008_Healthy_201-B7-axon
DRR287010_TARDBPedit_A7-axon
DRR287012_TARDBPedit_D9-axon
DRR287014_TARDBPedit_F4-axon
DRR287016_TARDBPedit_F11-axon
DRR286997_Healthy_201-soma
DRR286999_Healthy_409-soma
DRR287001_Healthy_409-2-soma
DRR287003_ALSpatient_CiRA26-soma
DRR287005_ALSpatient_CiRA26-2-soma
DRR287007_ALSpatient_TDP10-soma
DRR287009_Healthy_201-B7-soma
DRR287011_TARDBPedit_A7-soma
DRR287013_TARDBPedit_D9-soma
DRR287015_TARDBPedit_F4-soma
DRR287017_TARDBPedit_F11-soma
35606766
35607087
35607374
35607748
chr9:35606765-35607748:+
TESK1 | TESK1-204
TESK1 | TESK1-202
TESK1 | TESK1-203
TESK1 | TESK1-206
TESK1 | TESK1-201
TESK1 | TESK1-205

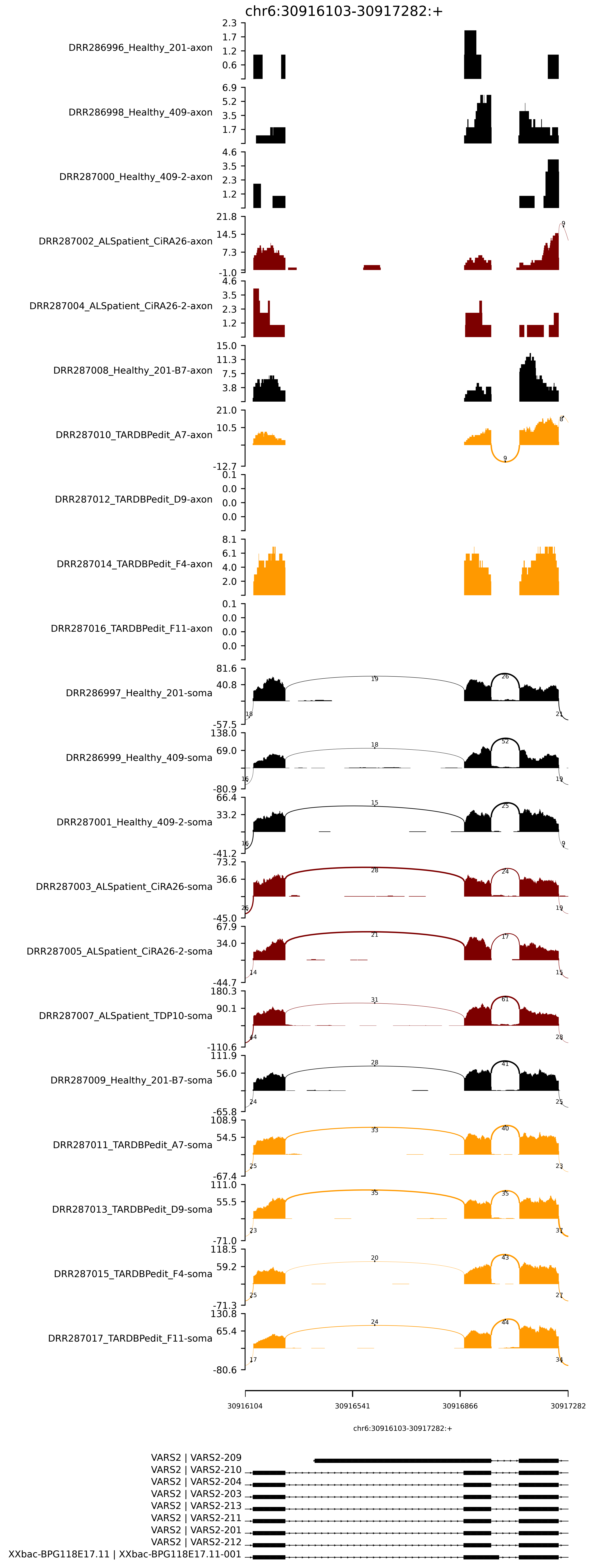
chr6:30916103-30917282:+
DRR286996_Healthy_201-axon
DRR286998_Healthy_409-axon
DRR287000_Healthy_409-2-axon
DRR287002_ALSpatient_CiRA26-axon
DRR287004_ALSpatient_CiRA26-2-axon
DRR287008_Healthy_201-B7-axon
DRR287010_TARDBPedit_A7-axon
DRR287012_TARDBPedit_D9-axon
DRR287014_TARDBPedit_F4-axon
DRR287016_TARDBPedit_F11-axon
DRR286997_Healthy_201-soma
DRR286999_Healthy_409-soma
DRR287001_Healthy_409-2-soma
DRR287003_ALSpatient_CiRA26-soma
DRR287005_ALSpatient_CiRA26-2-soma
DRR287007_ALSpatient_TDP10-soma
DRR287009_Healthy_201-B7-soma
DRR287011_TARDBPedit_A7-soma
DRR287013_TARDBPedit_D9-soma
DRR287015_TARDBPedit_F4-soma
DRR287017_TARDBPedit_F11-soma
30916104
30916541
30916866
30917282
chr6:30916103-30917282:+
VARS2 | VARS2-209
VARS2 | VARS2-210
VARS2 | VARS2-204
VARS2 | VARS2-203
VARS2 | VARS2-213
VARS2 | VARS2-211
VARS2 | VARS2-201
VARS2 | VARS2-212
XXbac-BPG118E17.11 | XXbac-BPG118E17.11-001

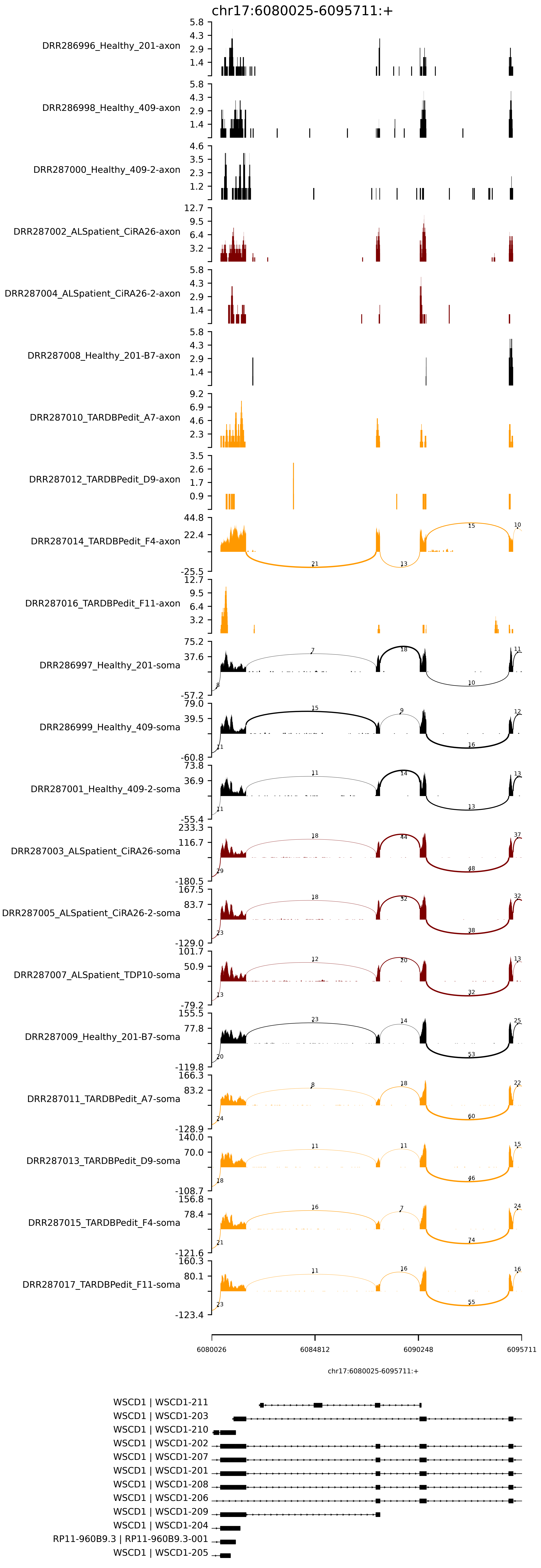

chr17:6080025-6095711:+
DRR286996_Healthy_201-axon
DRR286998_Healthy_409-axon
DRR287000_Healthy_409-2-axon
DRR287002_ALSpatient_CiRA26-axon
DRR287004_ALSpatient_CiRA26-2-axon
DRR287008_Healthy_201-B7-axon
DRR287010_TARDBPedit_A7-axon
DRR287012_TARDBPedit_D9-axon
DRR287014_TARDBPedit_F4-axon
DRR287016_TARDBPedit_F11-axon
DRR286997_Healthy_201-soma
DRR286999_Healthy_409-soma
DRR287001_Healthy_409-2-soma
DRR287003_ALSpatient_CiRA26-soma
DRR287005_ALSpatient_CiRA26-2-soma
DRR287007_ALSpatient_TDP10-soma
DRR287009_Healthy_201-B7-soma
DRR287011_TARDBPedit_A7-soma
DRR287013_TARDBPedit_D9-soma
DRR287015_TARDBPedit_F4-soma
DRR287017_TARDBPedit_F11-soma
6080026
6084812
6090248
6095711
chr17:6080025-6095711:+
WSCD1 | WSCD1-211
WSCD1 | WSCD1-203
WSCD1 | WSCD1-210
WSCD1 | WSCD1-202
WSCD1 | WSCD1-207
WSCD1 | WSCD1-201
WSCD1 | WSCD1-208
WSCD1 | WSCD1-206
WSCD1 | WSCD1-209
WSCD1 | WSCD1-204
RP11-960B9.3 | RP11-960B9.3-001
WSCD1 | WSCD1-205

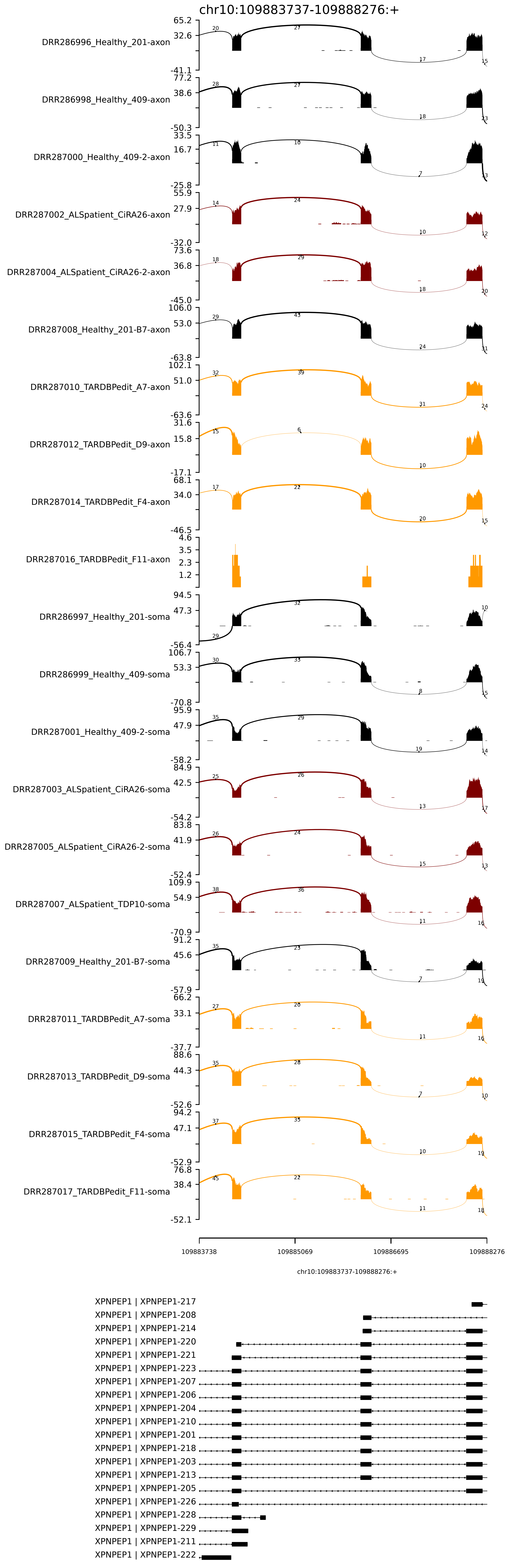

chr10:109883737-109888276:+
DRR286996_Healthy_201-axon
DRR286998_Healthy_409-axon
DRR287000_Healthy_409-2-axon
DRR287002_ALSpatient_CiRA26-axon
DRR287004_ALSpatient_CiRA26-2-axon
DRR287008_Healthy_201-B7-axon
DRR287010_TARDBPedit_A7-axon
DRR287012_TARDBPedit_D9-axon
DRR287014_TARDBPedit_F4-axon
DRR287016_TARDBPedit_F11-axon
DRR286997_Healthy_201-soma
DRR286999_Healthy_409-soma
DRR287001_Healthy_409-2-soma
DRR287003_ALSpatient_CiRA26-soma
DRR287005_ALSpatient_CiRA26-2-soma
DRR287007_ALSpatient_TDP10-soma
DRR287009_Healthy_201-B7-soma
DRR287011_TARDBPedit_A7-soma
DRR287013_TARDBPedit_D9-soma
DRR287015_TARDBPedit_F4-soma
DRR287017_TARDBPedit_F11-soma
109883738
109885069
109886695
109888276
chr10:109883737-109888276:+
XPNPEP1 | XPNPEP1-217
XPNPEP1 | XPNPEP1-208
XPNPEP1 | XPNPEP1-214
XPNPEP1 | XPNPEP1-220
XPNPEP1 | XPNPEP1-221
XPNPEP1 | XPNPEP1-223
XPNPEP1 | XPNPEP1-207
XPNPEP1 | XPNPEP1-206
XPNPEP1 | XPNPEP1-204
XPNPEP1 | XPNPEP1-210
XPNPEP1 | XPNPEP1-201
XPNPEP1 | XPNPEP1-218
XPNPEP1 | XPNPEP1-203
XPNPEP1 | XPNPEP1-213
XPNPEP1 | XPNPEP1-205
XPNPEP1 | XPNPEP1-226
XPNPEP1 | XPNPEP1-228
XPNPEP1 | XPNPEP1-229
XPNPEP1 | XPNPEP1-211
XPNPEP1 | XPNPEP1-222

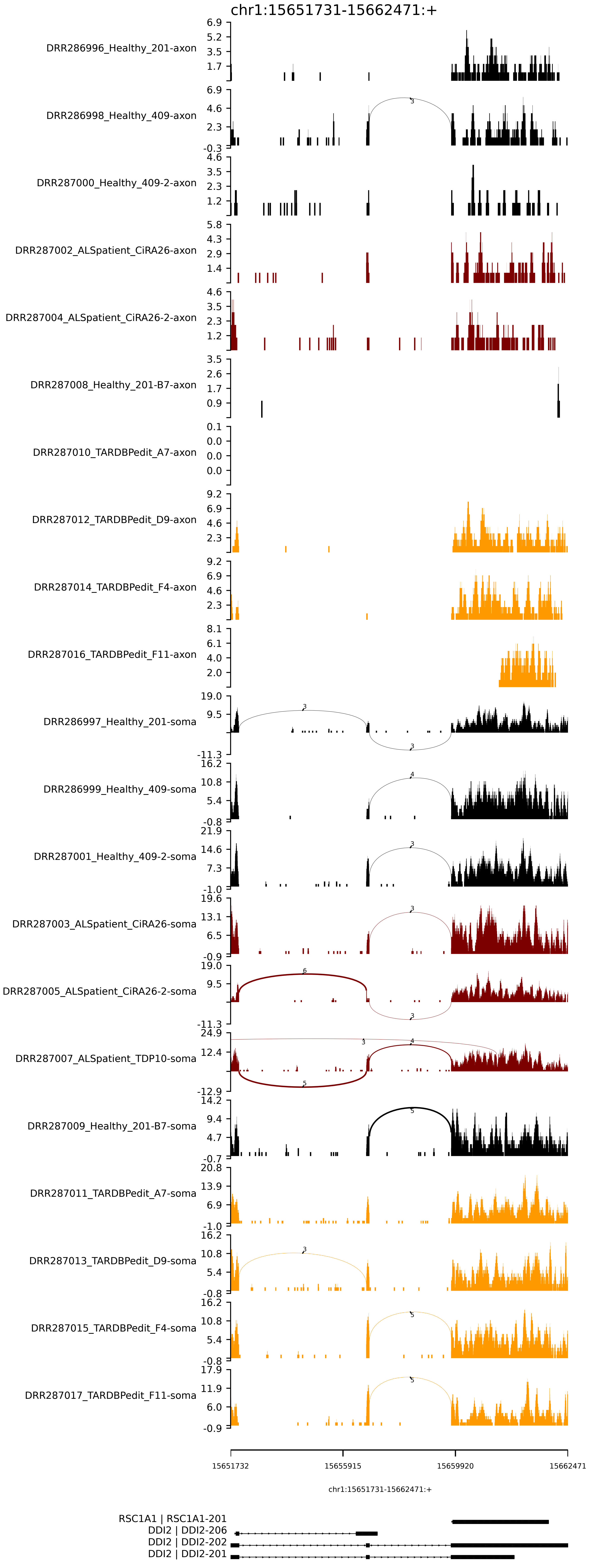
chr1:15651731-15662471:+
DRR286996_Healthy_201-axon
DRR286998_Healthy_409-axon
DRR287000_Healthy_409-2-axon
DRR287002_ALSpatient_CiRA26-axon
DRR287004_ALSpatient_CiRA26-2-axon
DRR287008_Healthy_201-B7-axon
DRR287010_TARDBPedit_A7-axon
DRR287012_TARDBPedit_D9-axon
DRR287014_TARDBPedit_F4-axon
DRR287016_TARDBPedit_F11-axon
DRR286997_Healthy_201-soma
DRR286999_Healthy_409-soma
DRR287001_Healthy_409-2-soma
DRR287003_ALSpatient_CiRA26-soma
DRR287005_ALSpatient_CiRA26-2-soma
DRR287007_ALSpatient_TDP10-soma
DRR287009_Healthy_201-B7-soma
DRR287011_TARDBPedit_A7-soma
DRR287013_TARDBPedit_D9-soma
DRR287015_TARDBPedit_F4-soma
DRR287017_TARDBPedit_F11-soma
15651732
15655915
15659920
15662471
chr1:15651731-15662471:+
RSC1A1 | RSC1A1-201
DDI2 | DDI2-206
DDI2 | DDI2-202
DDI2 | DDI2-201

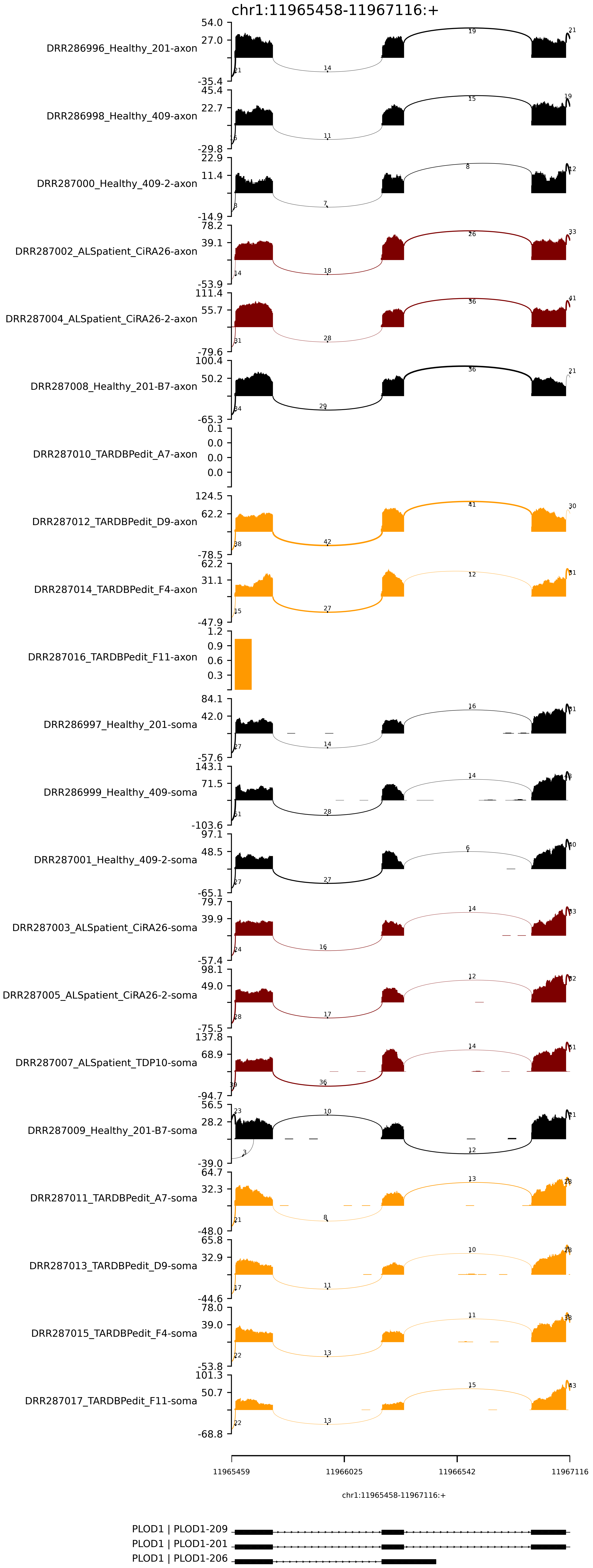

chr1:11965458-11967116:+
DRR286996_Healthy_201-axon
DRR286998_Healthy_409-axon
DRR287000_Healthy_409-2-axon
DRR287002_ALSpatient_CiRA26-axon
DRR287004_ALSpatient_CiRA26-2-axon
DRR287008_Healthy_201-B7-axon
DRR287010_TARDBPedit_A7-axon
DRR287012_TARDBPedit_D9-axon
DRR287014_TARDBPedit_F4-axon
DRR287016_TARDBPedit_F11-axon
DRR286997_Healthy_201-soma
DRR286999_Healthy_409-soma
DRR287001_Healthy_409-2-soma
DRR287003_ALSpatient_CiRA26-soma
DRR287005_ALSpatient_CiRA26-2-soma
DRR287007_ALSpatient_TDP10-soma
DRR287009_Healthy_201-B7-soma
DRR287011_TARDBPedit_A7-soma
DRR287013_TARDBPedit_D9-soma
DRR287015_TARDBPedit_F4-soma
DRR287017_TARDBPedit_F11-soma
11965459
11966025
11966542
11967116
chr1:11965458-11967116:+
PLOD1 | PLOD1-209
PLOD1 | PLOD1-201
PLOD1 | PLOD1-206

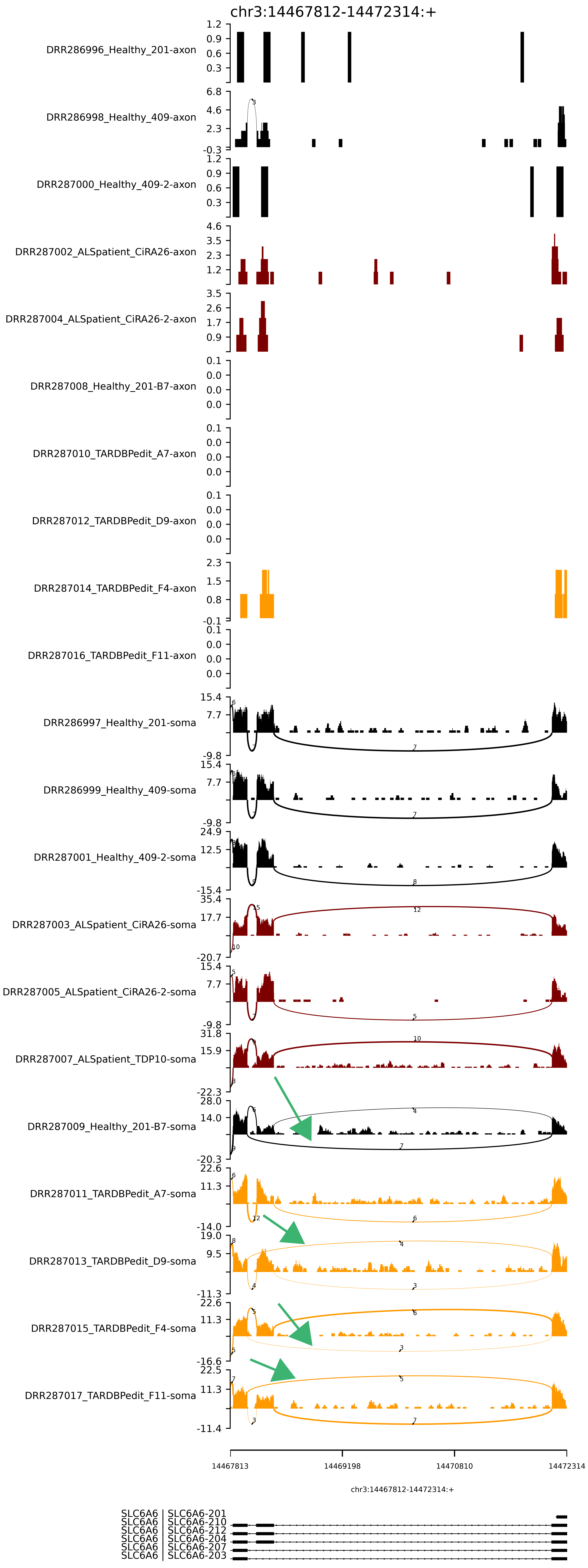
chr3:14467812-14472314:+
DRR286996_Healthy_201-axon
DRR286998_Healthy_409-axon
DRR287000_Healthy_409-2-axon
DRR287002_ALSpatient_CiRA26-axon
DRR287004_ALSpatient_CiRA26-2-axon
DRR287008_Healthy_201-B7-axon
DRR287010_TARDBPedit_A7-axon
DRR287012_TARDBPedit_D9-axon
DRR287014_TARDBPedit_F4-axon
DRR287016_TARDBPedit_F11-axon
DRR286997_Healthy_201-soma
DRR286999_Healthy_409-soma
DRR287001_Healthy_409-2-soma
DRR287003_ALSpatient_CiRA26-soma
DRR287005_ALSpatient_CiRA26-2-soma
DRR287007_ALSpatient_TDP10-soma
DRR287009_Healthy_201-B7-soma
DRR287011_TARDBPedit_A7-soma
DRR287013_TARDBPedit_D9-soma
DRR287015_TARDBPedit_F4-soma
DRR287017_TARDBPedit_F11-soma
14467813
14469198
14470810
14472314
chr3:14467812-14472314:+
SLC6A6 | SLC6A6-201
SLC6A6 | SLC6A6-210
SLC6A6 | SLC6A6-212
SLC6A6 | SLC6A6-204
SLC6A6 | SLC6A6-207
SLC6A6 | SLC6A6-203

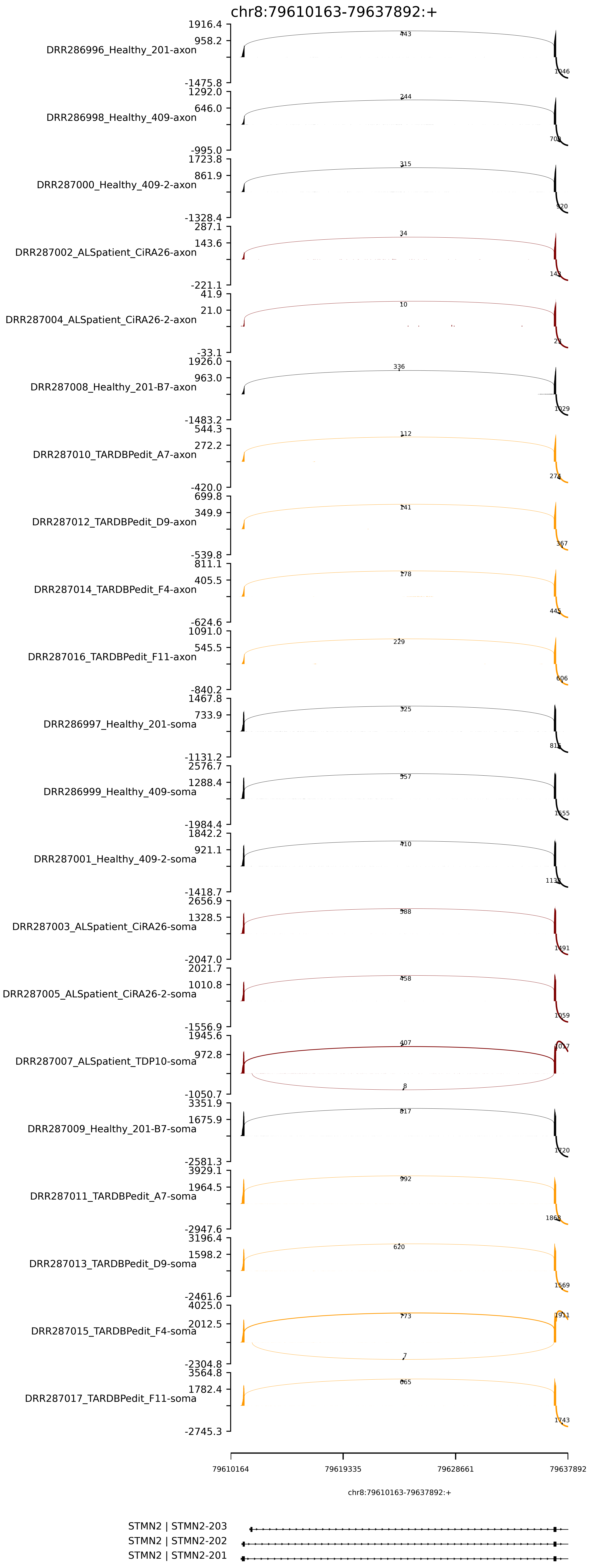
chr8:79610163-79637892:+
DRR286996_Healthy_201-axon
1916.4
958.2
-1475.8
443
1046
DRR286998_Healthy_409-axon
1292.0
646.0
-995.0
244
708
DRR287000_Healthy_409-2-axon
1723.8
861.9
-1328.4
315
920
DRR287002_ALSpatient_CiRA26-axon
287.1
143.6
-221.1
34
143
DRR287004_ALSpatient_CiRA26-2-axon
41.9
21.0
-33.1
10
23
DRR287008_Healthy_201-B7-axon
1926.0
963.0
-1483.2
336
1029
DRR287010_TARDBPedit_A7-axon
544.3
272.2
-420.0
112
274
DRR287012_TARDBPedit_D9-axon
699.8
349.9
-539.8
141
367
DRR287014_TARDBPedit_F4-axon
811.1
405.5
-624.6
178
445
DRR287016_TARDBPedit_F11-axon
1091.0
545.5
-840.2
229
606
DRR286997_Healthy_201-soma
1467.8
733.9
-1131.2
325
818
DRR286999_Healthy_409-soma
2576.7
1288.4
-1984.4
557
1555
DRR287001_Healthy_409-2-soma
1842.2
921.1
-1418.7
410
1138
DRR287003_ALSpatient_CiRA26-soma
2656.9
1328.5
-2047.0
588
1491
DRR287005_ALSpatient_CiRA26-2-soma
2021.7
1010.8
-1556.9
458
1059
DRR287007_ALSpatient_TDP10-soma
1945.6
972.8
-1050.7
407
1017
8
DRR287009_Healthy_201-B7-soma
3351.9
1675.9
-2581.3
817
1720
DRR287011_TARDBPedit_A7-soma
3929.1
1964.5
-2947.6
992
1868
DRR287013_TARDBPedit_D9-soma
3196.4
1598.2
-2461.6
620
1569
DRR287015_TARDBPedit_F4-soma
4025.0
2012.5
-2304.8
773
1911
7
DRR287017_TARDBPedit_F11-soma
3564.8
1782.4
-2745.3
665
1743
79610164
79619335
79628661
79637892
chr8:79610163-79637892:+
STMN2 | STMN2-203
STMN2 | STMN2-202
STMN2 | STMN2-201

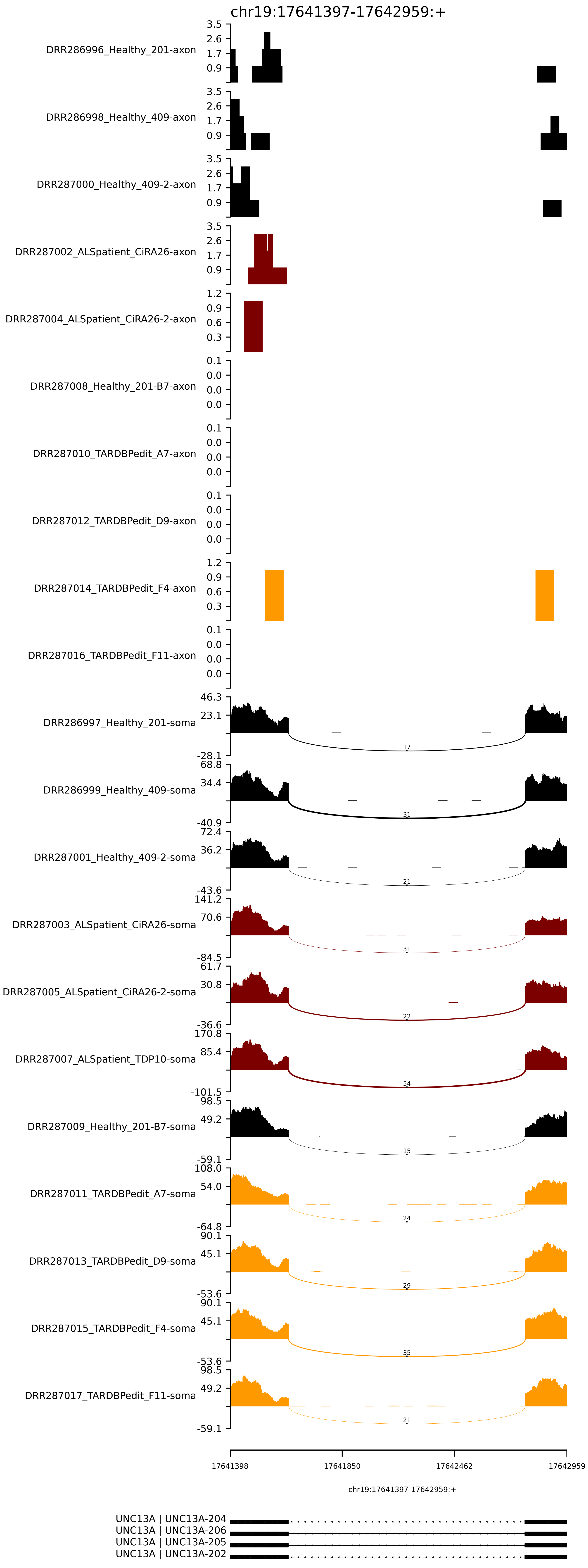

chr19:17641397-17642959:+
DRR286996_Healthy_201-axon
DRR286998_Healthy_409-axon
DRR287000_Healthy_409-2-axon
DRR287002_ALSpatient_CiRA26-axon
DRR287004_ALSpatient_CiRA26-2-axon
DRR287008_Healthy_201-B7-axon
DRR287010_TARDBPedit_A7-axon
DRR287012_TARDBPedit_D9-axon
DRR287014_TARDBPedit_F4-axon
DRR287016_TARDBPedit_F11-axon
DRR286997_Healthy_201-soma
DRR286999_Healthy_409-soma
DRR287001_Healthy_409-2-soma
DRR287003_ALSpatient_CiRA26-soma
DRR287005_ALSpatient_CiRA26-2-soma
DRR287007_ALSpatient_TDP10-soma
DRR287009_Healthy_201-B7-soma
DRR287011_TARDBPedit_A7-soma
DRR287013_TARDBPedit_D9-soma
DRR287015_TARDBPedit_F4-soma
DRR287017_TARDBPedit_F11-soma
17
31
21
31
22
54
15
24
29
35
21
17641398
17641850
17642462
17642959
chr19:17641397-17642959:+
UNC13A | UNC13A-204
UNC13A | UNC13A-206
UNC13A | UNC13A-205
UNC13A | UNC13A-202

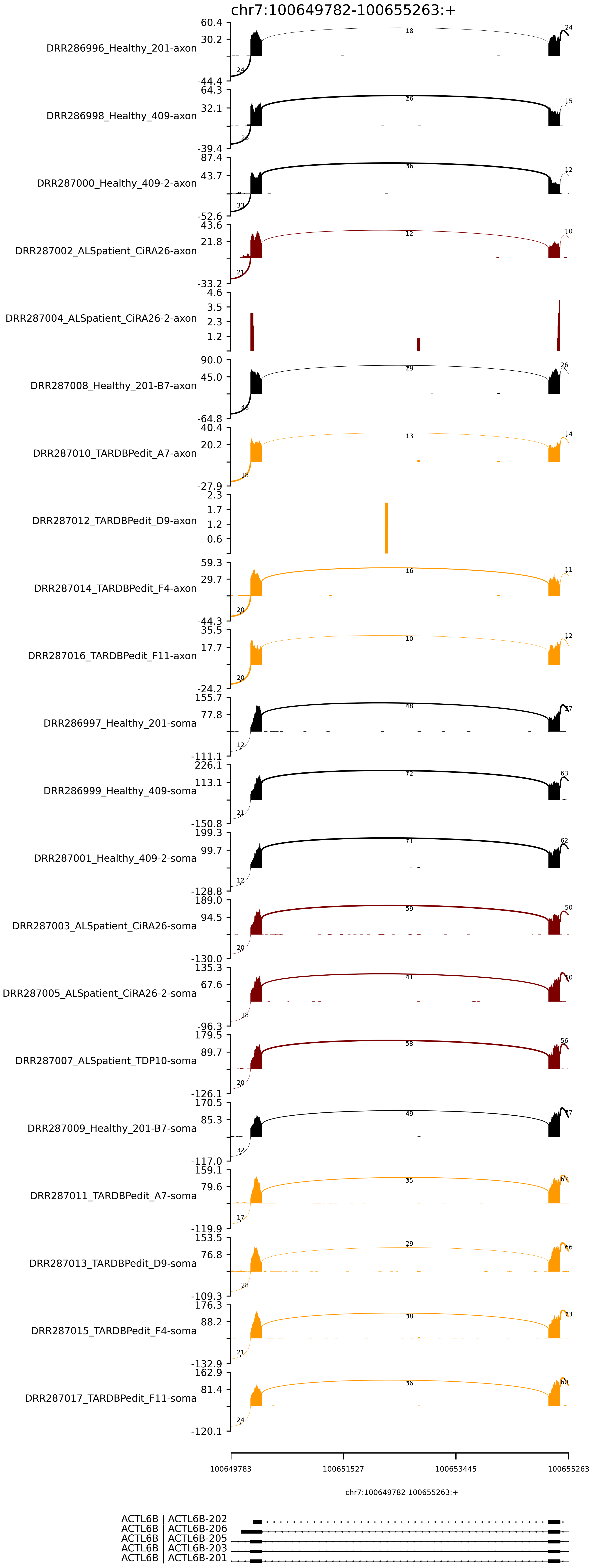
chr7:100649782-100655263:+
DRR286996_Healthy_201-axon
DRR286998_Healthy_409-axon
DRR287000_Healthy_409-2-axon
DRR287002_ALSpatient_CiRA26-axon
DRR287004_ALSpatient_CiRA26-2-axon
DRR287008_Healthy_201-B7-axon
DRR287010_TARDBPedit_A7-axon
DRR287012_TARDBPedit_D9-axon
DRR287014_TARDBPedit_F4-axon
DRR287016_TARDBPedit_F11-axon
DRR286997_Healthy_201-soma
DRR286999_Healthy_409-soma
DRR287001_Healthy_409-2-soma
DRR287003_ALSpatient_CiRA26-soma
DRR287005_ALSpatient_CiRA26-2-soma
DRR287007_ALSpatient_TDP10-soma
DRR287009_Healthy_201-B7-soma
DRR287011_TARDBPedit_A7-soma
DRR287013_TARDBPedit_D9-soma
DRR287015_TARDBPedit_F4-soma
DRR287017_TARDBPedit_F11-soma
100649783
100651527
100653445
100655263
chr7:100649782-100655263:+
ACTL6B | ACTL6B-202
ACTL6B | ACTL6B-206
ACTL6B | ACTL6B-205
ACTL6B | ACTL6B-203
ACTL6B | ACTL6B-201

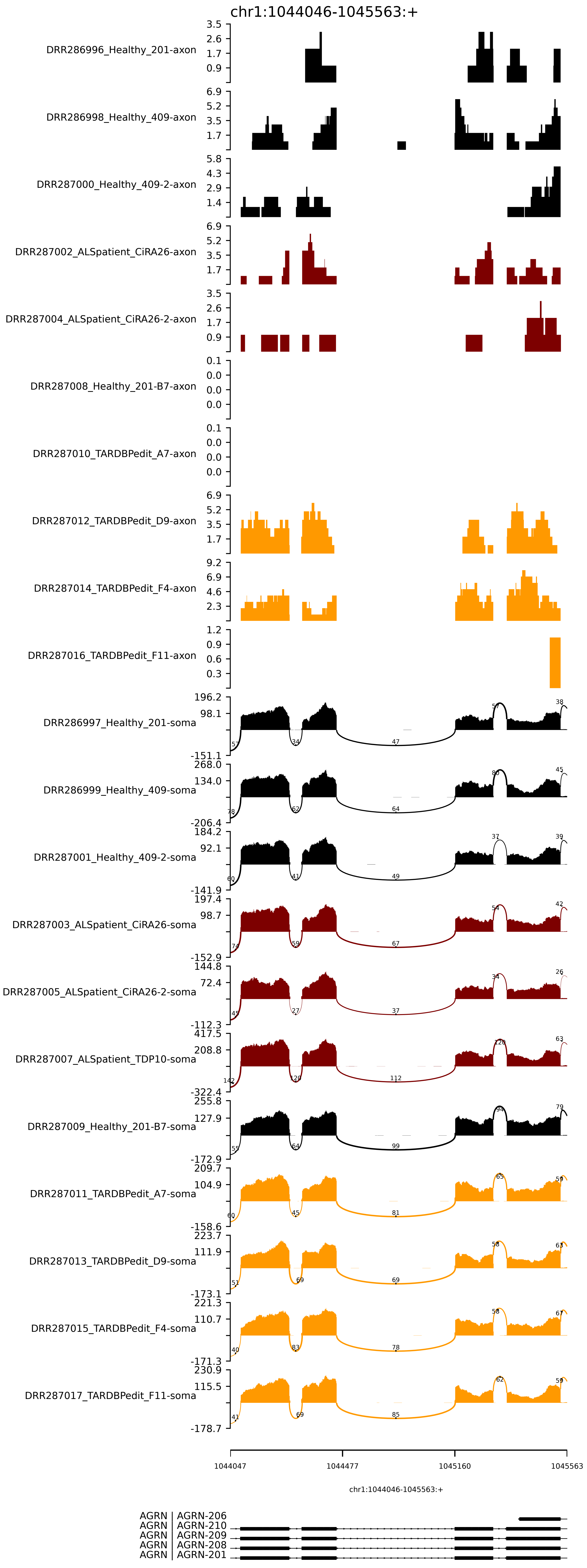
chr1:1044046-1045563:+
DRR286996_Healthy_201-axon
DRR286998_Healthy_409-axon
DRR287000_Healthy_409-2-axon
DRR287002_ALSpatient_CiRA26-axon
DRR287004_ALSpatient_CiRA26-2-axon
DRR287008_Healthy_201-B7-axon
DRR287010_TARDBPedit_A7-axon
DRR287012_TARDBPedit_D9-axon
DRR287014_TARDBPedit_F4-axon
DRR287016_TARDBPedit_F11-axon
DRR286997_Healthy_201-soma
DRR286999_Healthy_409-soma
DRR287001_Healthy_409-2-soma
DRR287003_ALSpatient_CiRA26-soma
DRR287005_ALSpatient_CiRA26-2-soma
DRR287007_ALSpatient_TDP10-soma
DRR287009_Healthy_201-B7-soma
DRR287011_TARDBPedit_A7-soma
DRR287013_TARDBPedit_D9-soma
DRR287015_TARDBPedit_F4-soma
DRR287017_TARDBPedit_F11-soma
1044047
1044477
1045160
1045563
chr1:1044046-1045563:+
AGRN | AGRN-206
AGRN | AGRN-210
AGRN | AGRN-209
AGRN | AGRN-208
AGRN | AGRN-201

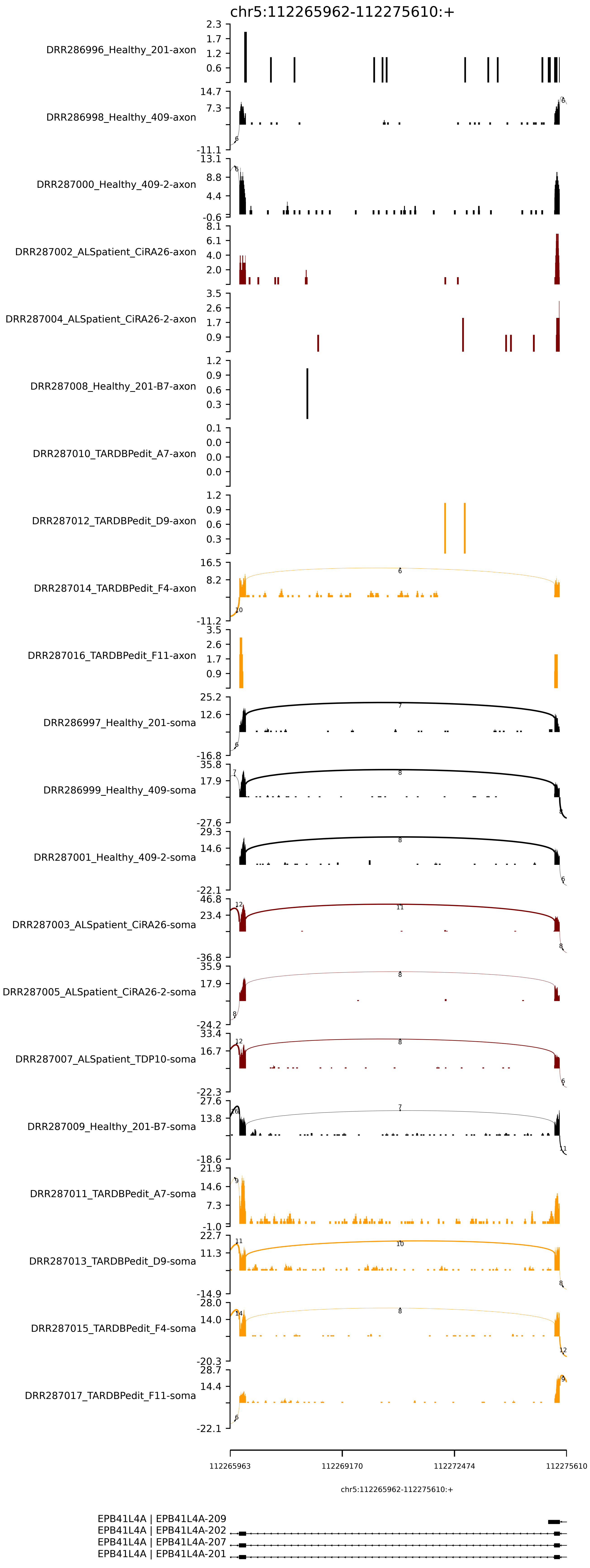
chr5:112265962-112275610:+
DRR286996_Healthy_201-axon
DRR286998_Healthy_409-axon
DRR287000_Healthy_409-2-axon
DRR287002_ALSpatient_CiRA26-axon
DRR287004_ALSpatient_CiRA26-2-axon
DRR287008_Healthy_201-B7-axon
DRR287010_TARDBPedit_A7-axon
DRR287012_TARDBPedit_D9-axon
DRR287014_TARDBPedit_F4-axon
DRR287016_TARDBPedit_F11-axon
DRR286997_Healthy_201-soma
DRR286999_Healthy_409-soma
DRR287001_Healthy_409-2-soma
DRR287003_ALSpatient_CiRA26-soma
DRR287005_ALSpatient_CiRA26-2-soma
DRR287007_ALSpatient_TDP10-soma
DRR287009_Healthy_201-B7-soma
DRR287011_TARDBPedit_A7-soma
DRR287013_TARDBPedit_D9-soma
DRR287015_TARDBPedit_F4-soma
DRR287017_TARDBPedit_F11-soma
112265963
112269170
112272474
112275610
chr5:112265962-112275610:+
EPB41L4A | EPB41L4A-209
EPB41L4A | EPB41L4A-202
EPB41L4A | EPB41L4A-207
EPB41L4A | EPB41L4A-201

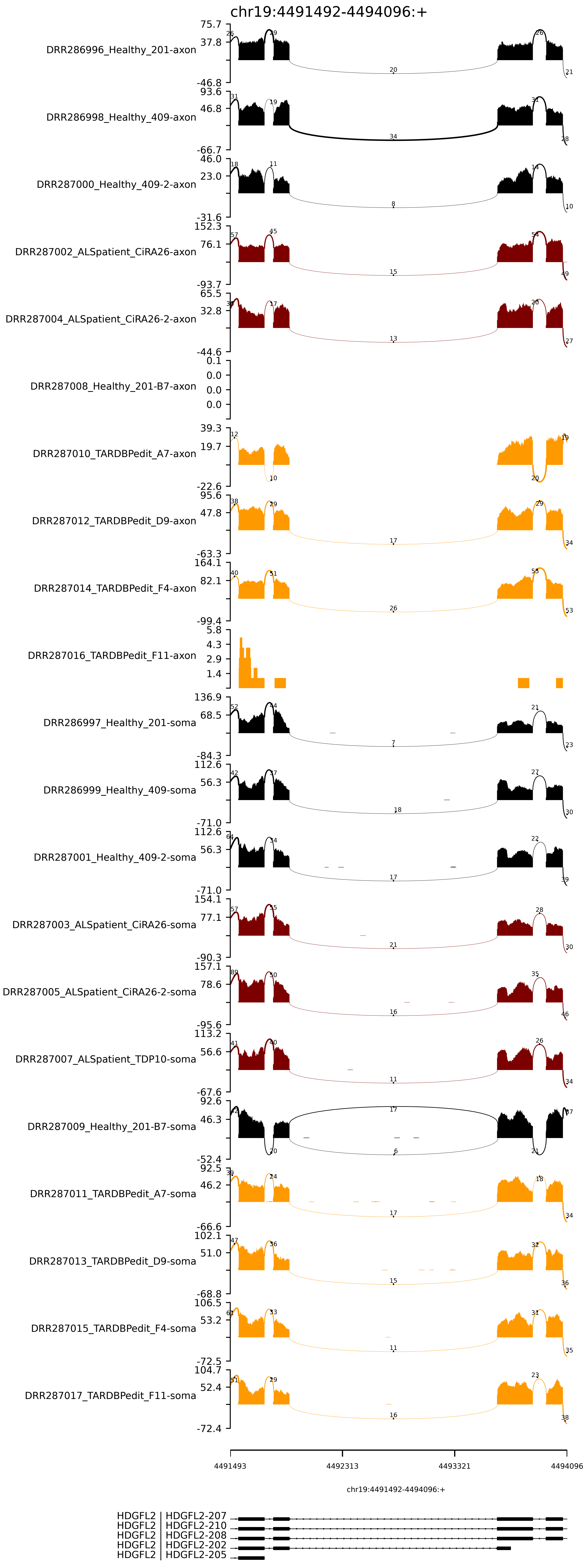

chr19:4491492-4494096:+
DRR286996_Healthy_201-axon
DRR286998_Healthy_409-axon
DRR287000_Healthy_409-2-axon
DRR287002_ALSpatient_CiRA26-axon
DRR287004_ALSpatient_CiRA26-2-axon
DRR287008_Healthy_201-B7-axon
DRR287010_TARDBPedit_A7-axon
DRR287012_TARDBPedit_D9-axon
DRR287014_TARDBPedit_F4-axon
DRR287016_TARDBPedit_F11-axon
DRR286997_Healthy_201-soma
DRR286999_Healthy_409-soma
DRR287001_Healthy_409-2-soma
DRR287003_ALSpatient_CiRA26-soma
DRR287005_ALSpatient_CiRA26-2-soma
DRR287007_ALSpatient_TDP10-soma
DRR287009_Healthy_201-B7-soma
DRR287011_TARDBPedit_A7-soma
DRR287013_TARDBPedit_D9-soma
DRR287015_TARDBPedit_F4-soma
DRR287017_TARDBPedit_F11-soma
4491493
4492313
4493321
4494096
chr19:4491492-4494096:+
HDGFL2 | HDGFL2-207
HDGFL2 | HDGFL2-210
HDGFL2 | HDGFL2-208
HDGFL2 | HDGFL2-202
HDGFL2 | HDGFL2-205

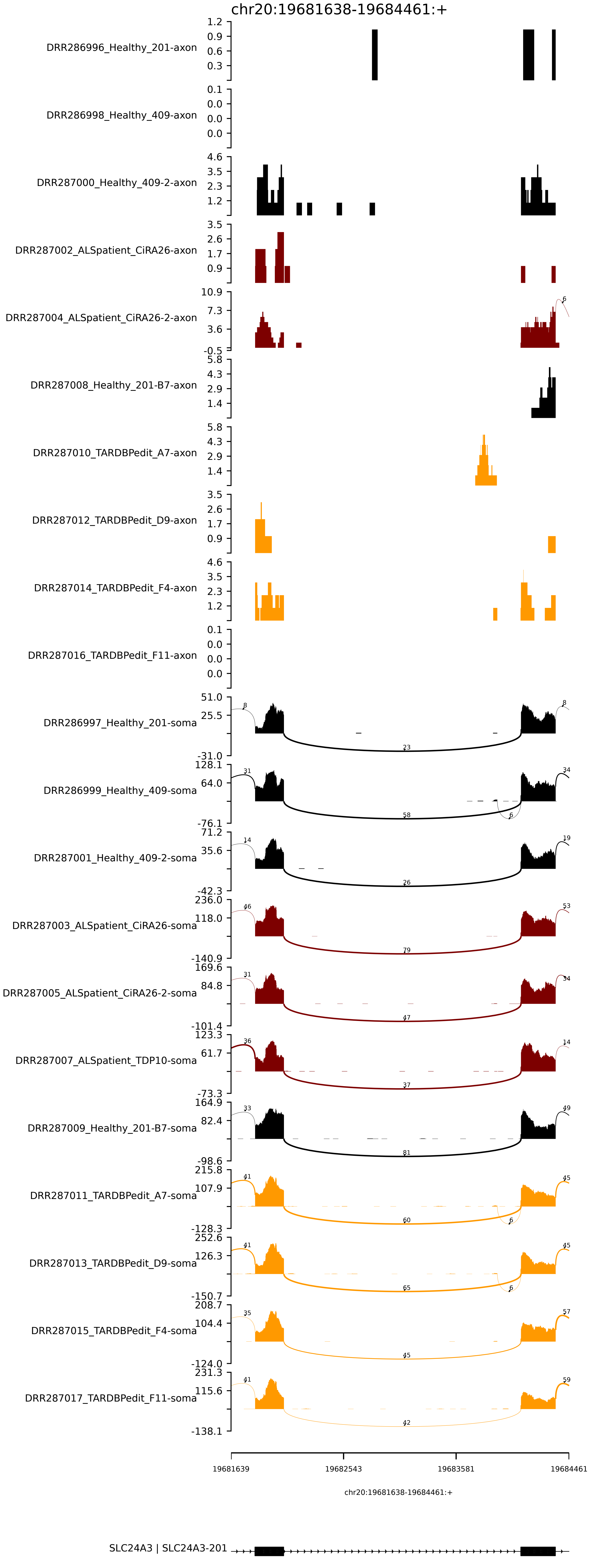

chr20:19681638-19684461:+
DRR286996_Healthy_201-axon
DRR286998_Healthy_409-axon
DRR287000_Healthy_409-2-axon
DRR287002_ALSpatient_CiRA26-axon
DRR287004_ALSpatient_CiRA26-2-axon
DRR287008_Healthy_201-B7-axon
DRR287010_TARDBPedit_A7-axon
DRR287012_TARDBPedit_D9-axon
DRR287014_TARDBPedit_F4-axon
DRR287016_TARDBPedit_F11-axon
DRR286997_Healthy_201-soma
DRR286999_Healthy_409-soma
DRR287001_Healthy_409-2-soma
DRR287003_ALSpatient_CiRA26-soma
DRR287005_ALSpatient_CiRA26-2-soma
DRR287007_ALSpatient_TDP10-soma
DRR287009_Healthy_201-B7-soma
DRR287011_TARDBPedit_A7-soma
DRR287013_TARDBPedit_D9-soma
DRR287015_TARDBPedit_F4-soma
DRR287017_TARDBPedit_F11-soma
19681639
19682543
19683581
19684461
chr20:19681638-19684461:+
SLC24A3 | SLC24A3-201

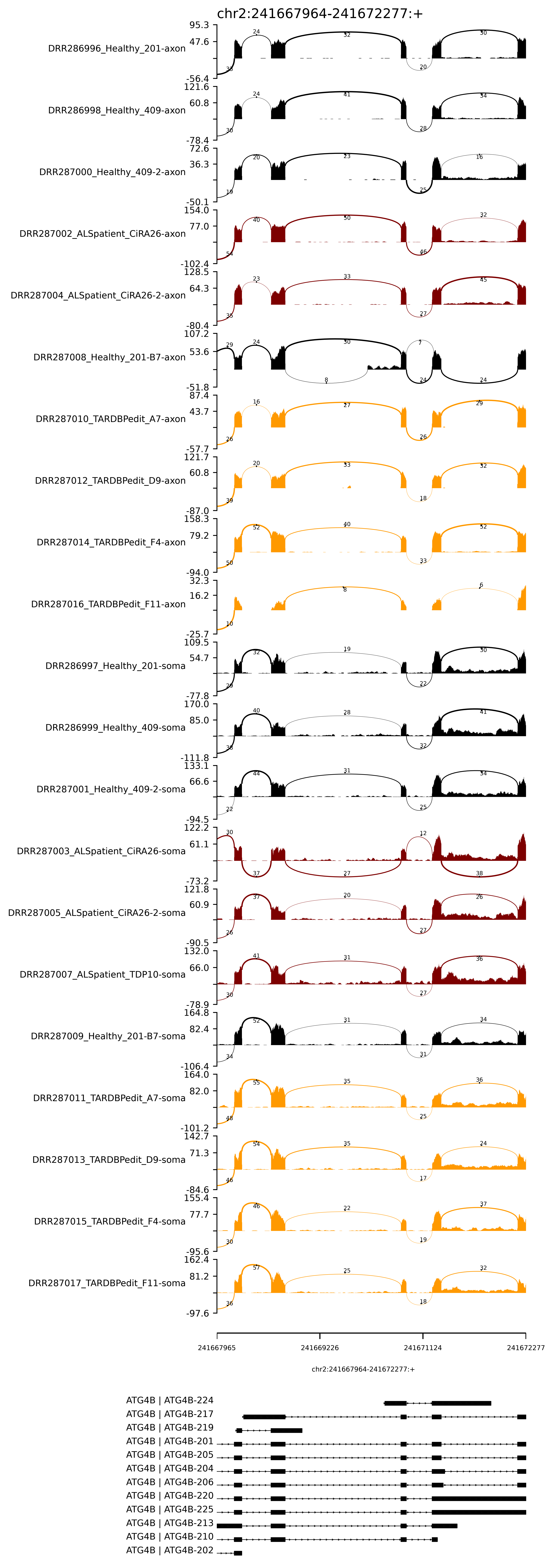
chr2:241667964-241672277:+
DRR286996_Healthy_201-axon
DRR286998_Healthy_409-axon
DRR287000_Healthy_409-2-axon
DRR287002_ALSpatient_CiRA26-axon
DRR287004_ALSpatient_CiRA26-2-axon
DRR287008_Healthy_201-B7-axon
DRR287010_TARDBPedit_A7-axon
DRR287012_TARDBPedit_D9-axon
DRR287014_TARDBPedit_F4-axon
DRR287016_TARDBPedit_F11-axon
DRR286997_Healthy_201-soma
DRR286999_Healthy_409-soma
DRR287001_Healthy_409-2-soma
DRR287003_ALSpatient_CiRA26-soma
DRR287005_ALSpatient_CiRA26-2-soma
DRR287007_ALSpatient_TDP10-soma
DRR287009_Healthy_201-B7-soma
DRR287011_TARDBPedit_A7-soma
DRR287013_TARDBPedit_D9-soma
DRR287015_TARDBPedit_F4-soma
DRR287017_TARDBPedit_F11-soma
241667965
241669226
241671124
241672277
chr2:241667964-241672277:+
ATG4B | ATG4B-224
ATG4B | ATG4B-217
ATG4B | ATG4B-219
ATG4B | ATG4B-201
ATG4B | ATG4B-205
ATG4B | ATG4B-204
ATG4B | ATG4B-206
ATG4B | ATG4B-220
ATG4B | ATG4B-225
ATG4B | ATG4B-213
ATG4B | ATG4B-210
ATG4B | ATG4B-202

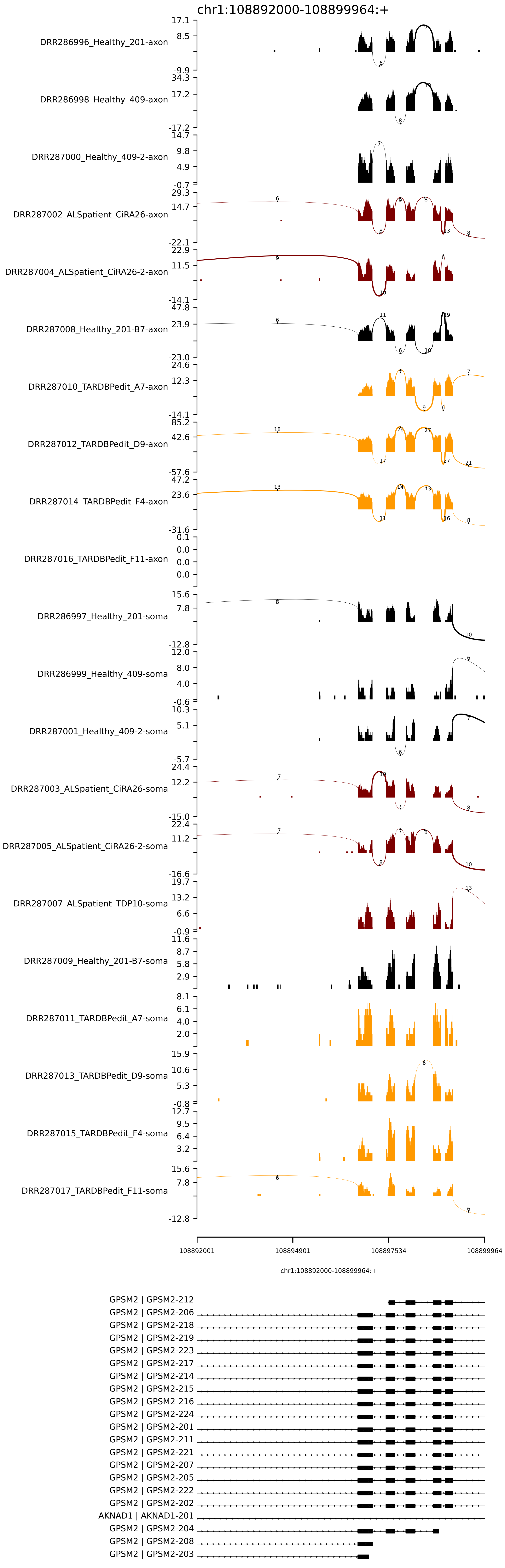
chr1:108892000-108899964:+
DRR286996_Healthy_201-axon
DRR286998_Healthy_409-axon
DRR287000_Healthy_409-2-axon
DRR287002_ALSpatient_CiRA26-axon
DRR287004_ALSpatient_CiRA26-2-axon
DRR287008_Healthy_201-B7-axon
DRR287010_TARDBPedit_A7-axon
DRR287012_TARDBPedit_D9-axon
DRR287014_TARDBPedit_F4-axon
DRR287016_TARDBPedit_F11-axon
DRR286997_Healthy_201-soma
DRR286999_Healthy_409-soma
DRR287001_Healthy_409-2-soma
DRR287003_ALSpatient_CiRA26-soma
DRR287005_ALSpatient_CiRA26-2-soma
DRR287007_ALSpatient_TDP10-soma
DRR287009_Healthy_201-B7-soma
DRR287011_TARDBPedit_A7-soma
DRR287013_TARDBPedit_D9-soma
DRR287015_TARDBPedit_F4-soma
DRR287017_TARDBPedit_F11-soma
108892001
108894901
108897534
108899964
chr1:108892000-108899964:+
GPSM2 | GPSM2-212
GPSM2 | GPSM2-206
GPSM2 | GPSM2-218
GPSM2 | GPSM2-219
GPSM2 | GPSM2-223
GPSM2 | GPSM2-217
GPSM2 | GPSM2-214
GPSM2 | GPSM2-215
GPSM2 | GPSM2-216
GPSM2 | GPSM2-224
GPSM2 | GPSM2-201
GPSM2 | GPSM2-211
GPSM2 | GPSM2-221
GPSM2 | GPSM2-207
GPSM2 | GPSM2-205
GPSM2 | GPSM2-222
GPSM2 | GPSM2-202
AKNAD1 | AKNAD1-201
GPSM2 | GPSM2-204
GPSM2 | GPSM2-208
GPSM2 | GPSM2-203

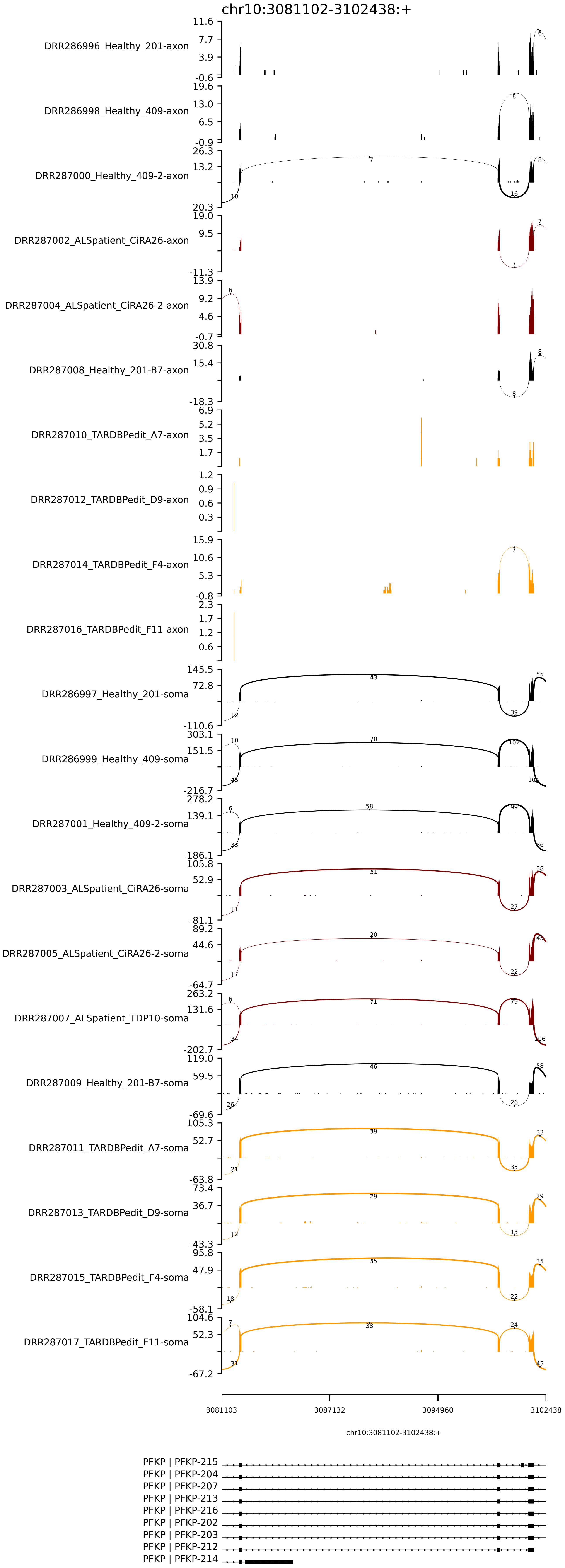
chr10:3081102-3102438:+
DRR286996_Healthy_201-axon
DRR286998_Healthy_409-axon
DRR287000_Healthy_409-2-axon
DRR287002_ALSpatient_CiRA26-axon
DRR287004_ALSpatient_CiRA26-2-axon
DRR287008_Healthy_201-B7-axon
DRR287010_TARDBPedit_A7-axon
DRR287012_TARDBPedit_D9-axon
DRR287014_TARDBPedit_F4-axon
DRR287016_TARDBPedit_F11-axon
DRR286997_Healthy_201-soma
DRR286999_Healthy_409-soma
DRR287001_Healthy_409-2-soma
DRR287003_ALSpatient_CiRA26-soma
DRR287005_ALSpatient_CiRA26-2-soma
DRR287007_ALSpatient_TDP10-soma
DRR287009_Healthy_201-B7-soma
DRR287011_TARDBPedit_A7-soma
DRR287013_TARDBPedit_D9-soma
DRR287015_TARDBPedit_F4-soma
DRR287017_TARDBPedit_F11-soma
3081103
3087132
3094960
3102438
chr10:3081102-3102438:+
PFKP | PFKP-215
PFKP | PFKP-204
PFKP | PFKP-207
PFKP | PFKP-213
PFKP | PFKP-216
PFKP | PFKP-202
PFKP | PFKP-203
PFKP | PFKP-212
PFKP | PFKP-214
