# Supplementary material for: Elevated nuclear TDP-43 induces constitutive exon skipping
Source: Mol Neurodegener. 2024 Jun 9;19:45. doi: 10.1186/s13024-024-00732-w (PMC11163724; doi:10.1186/s13024-024-00732-w)
Supplement: Supplementary file 9 — Supplementary Material 9 [file 13024_2024_732_MOESM9_ESM.pdf]

Gene	Coordinates	Splicing Type	Data
BMPR1A	chr10:86921303-86924982	skiptic exon	Carmen-Orozco, et al. 2023
CANX	chr5:179698416-179706991	skiptic exon	Carmen-Orozco, et al. 2023
COQ5	chr12:120516319-120526842	skiptic exon	Carmen-Orozco, et al. 2023
ELP2	chr18:36166598-36171437	skiptic exon	Carmen-Orozco, et al. 2023
HYOU1	chr11:119052044-119052857	skiptic exon	Carmen-Orozco, et al. 2023
MYBBP1A	chr17:4544952-4545777	skiptic exon	Carmen-Orozco, et al. 2023
NUP93	chr16:56838753-56841937	skiptic exon	Carmen-Orozco, et al. 2023
SCN9A	chr2:166272081-166280656	skiptic exon	Carmen-Orozco, et al. 2023
SESN3	chr11:95185056-95191820	skiptic exon	Carmen-Orozco, et al. 2023
SLC35A5	chr3:112562822-112571073	skiptic exon	Carmen-Orozco, et al. 2023
TESK1	chr9:35606765-35607748	skiptic exon	Carmen-Orozco, et al. 2023
VARS2	chr6:30916103-30917282	skiptic exon	Carmen-Orozco, et al. 2023
WSCD1	chr17:6080025-6095711	skiptic exon	Carmen-Orozco, et al. 2023
XPNPEP1	chr10:109883737-109888276	skiptic exon	Carmen-Orozco, et al. 2023
DDI2	chr1:15651731-15662471	skiptic exon	Carmen-Orozco, et al. 2023
PLOD1	chr1:11965458-11967116	skiptic exon	Fratta, et al. 2018
SLC6A6	chr3:14467812-14472314	skiptic exon	Fratta, et al. 2018
ACTL6B	chr7:100649782-100655263	cryptic exon	Irwin, et al. 2024
AGRN	chr1:1044046-1045563	cryptic exon	Irwin, et al. 2025
EPB41L4A	chr5:112265962-112275610	cryptic exon	Irwin, et al. 2026
HDGFL2	chr19:4491492-4494096	cryptic exon	Irwin, et al. 2027
SLC24A3	chr20:19681638-19684461	cryptic exon	Irwin, et al. 2028
STMN2	chr8:79610163-79637892	cryptic exon	Klim, et al. 2019, Melamed et al. 2019
UNC13A	chr19:17641397-17642959	cryptic exon	Rosa Ma, et al. 2022
ATG4B	chr2:241667964-241672277	cryptic exon	Ling, et al. 2015
GPSM2	chr1:108892000-108899964	cryptic exon	Ling, et al. 2015
PFKP	chr10:3081102-3102438	cryptic exon	Ling, et al. 2015

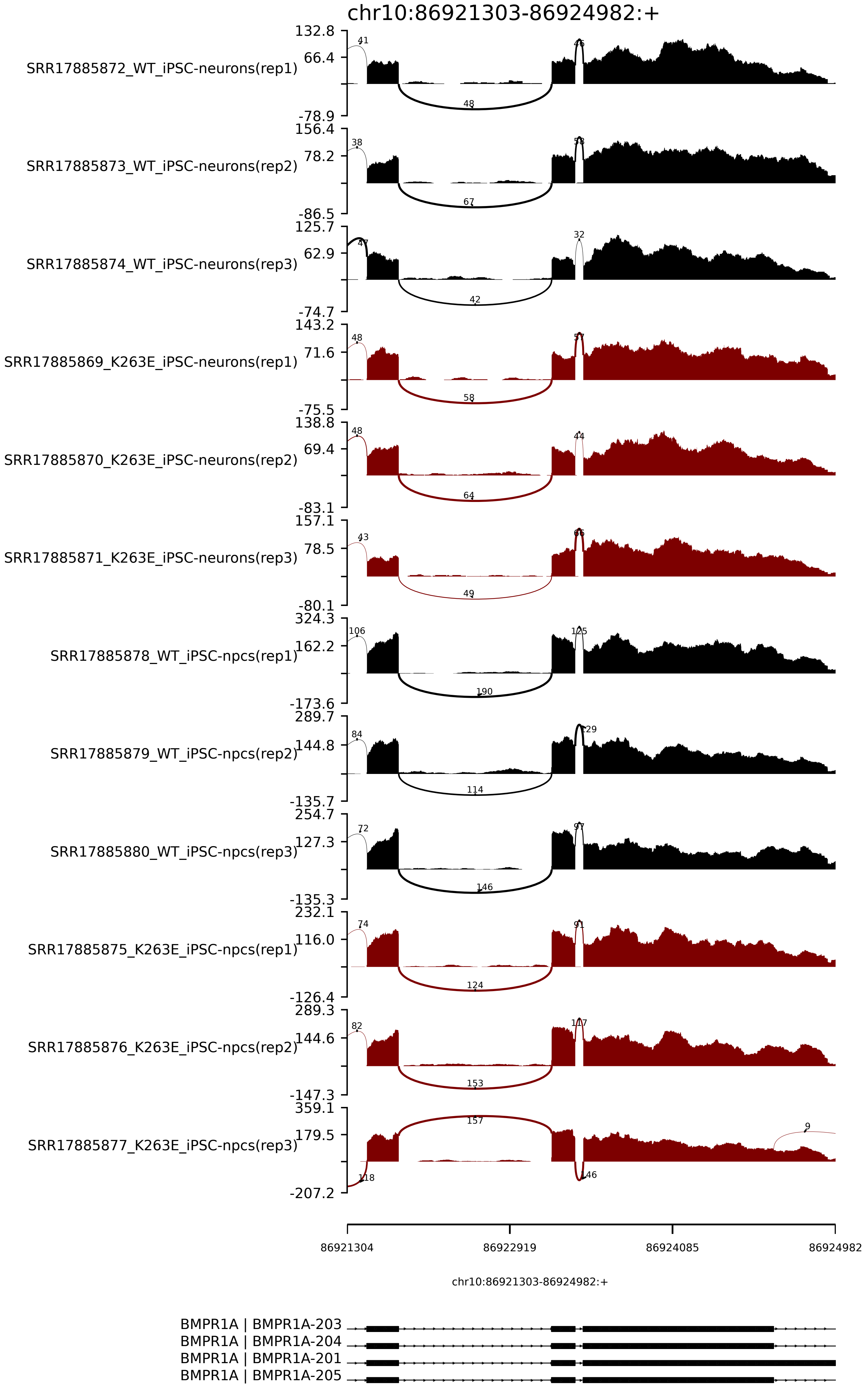

chr10:86921303-86924982:+
SRR17885872_WT_iPSC-neurons(rep1)
SRR17885873_WT_iPSC-neurons(rep2)
SRR17885874_WT_iPSC-neurons(rep3)
SRR17885869_K263E_iPSC-neurons(rep1)
SRR17885870_K263E_iPSC-neurons(rep2)
SRR17885871_K263E_iPSC-neurons(rep3)
SRR17885878_WT_iPSC-npcs(rep1)
SRR17885879_WT_iPSC-npcs(rep2)
SRR17885880_WT_iPSC-npcs(rep3)
SRR17885875_K263E_iPSC-npcs(rep1)
SRR17885876_K263E_iPSC-npcs(rep2)
SRR17885877_K263E_iPSC-npcs(rep3)
86921304
86922919
86924085
86924982
chr10:86921303-86924982:+
BMPR1A | BMPR1A-203
BMPR1A | BMPR1A-204
BMPR1A | BMPR1A-201
BMPR1A | BMPR1A-205

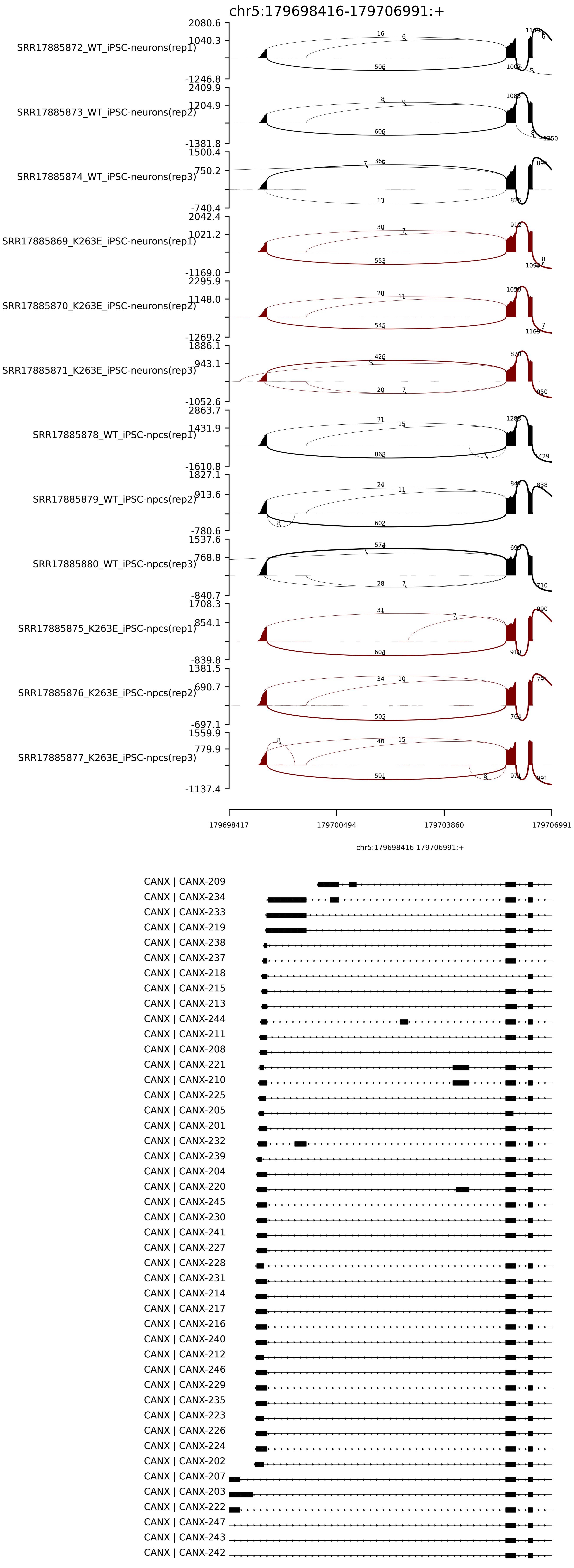
chr5:179698416-179706991:+
SRR17885872_WT_iPSC-neurons(rep1)
2080.6
1040.3
-1246.8
SRR17885873_WT_iPSC-neurons(rep2)
2409.9
1204.9
-1381.8
SRR17885874_WT_iPSC-neurons(rep3)
1500.4
750.2
-740.4
SRR17885869_K263E_iPSC-neurons(rep1)
2042.4
1021.2
-1169.0
SRR17885870_K263E_iPSC-neurons(rep2)
2295.9
1148.0
-1269.2
SRR17885871_K263E_iPSC-neurons(rep3)
1886.1
943.1
-1052.6
SRR17885878_WT_iPSC-npcs(rep1)
2863.7
1431.9
-1610.8
SRR17885879_WT_iPSC-npcs(rep2)
1827.1
913.6
-780.6
SRR17885880_WT_iPSC-npcs(rep3)
1537.6
768.8
-840.7
SRR17885875_K263E_iPSC-npcs(rep1)
1708.3
854.1
-839.8
SRR17885876_K263E_iPSC-npcs(rep2)
1381.5
690.7
-697.1
SRR17885877_K263E_iPSC-npcs(rep3)
1559.9
779.9
-1137.4
179698417
179700494
179703860
179706991
chr5:179698416-179706991:+
CANX | CANX-209
CANX | CANX-234
CANX | CANX-233
CANX | CANX-219
CANX | CANX-238
CANX | CANX-237
CANX | CANX-218
CANX | CANX-215
CANX | CANX-213
CANX | CANX-244
CANX | CANX-211
CANX | CANX-208
CANX | CANX-221
CANX | CANX-210
CANX | CANX-225
CANX | CANX-205
CANX | CANX-201
CANX | CANX-232
CANX | CANX-239
CANX | CANX-204
CANX | CANX-220
CANX | CANX-245
CANX | CANX-230
CANX | CANX-241
CANX | CANX-227
CANX | CANX-228
CANX | CANX-231
CANX | CANX-214
CANX | CANX-217
CANX | CANX-216
CANX | CANX-240
CANX | CANX-212
CANX | CANX-246
CANX | CANX-229
CANX | CANX-235
CANX | CANX-223
CANX | CANX-226
CANX | CANX-224
CANX | CANX-202
CANX | CANX-207
CANX | CANX-203
CANX | CANX-222
CANX | CANX-247
CANX | CANX-243
CANX | CANX-242

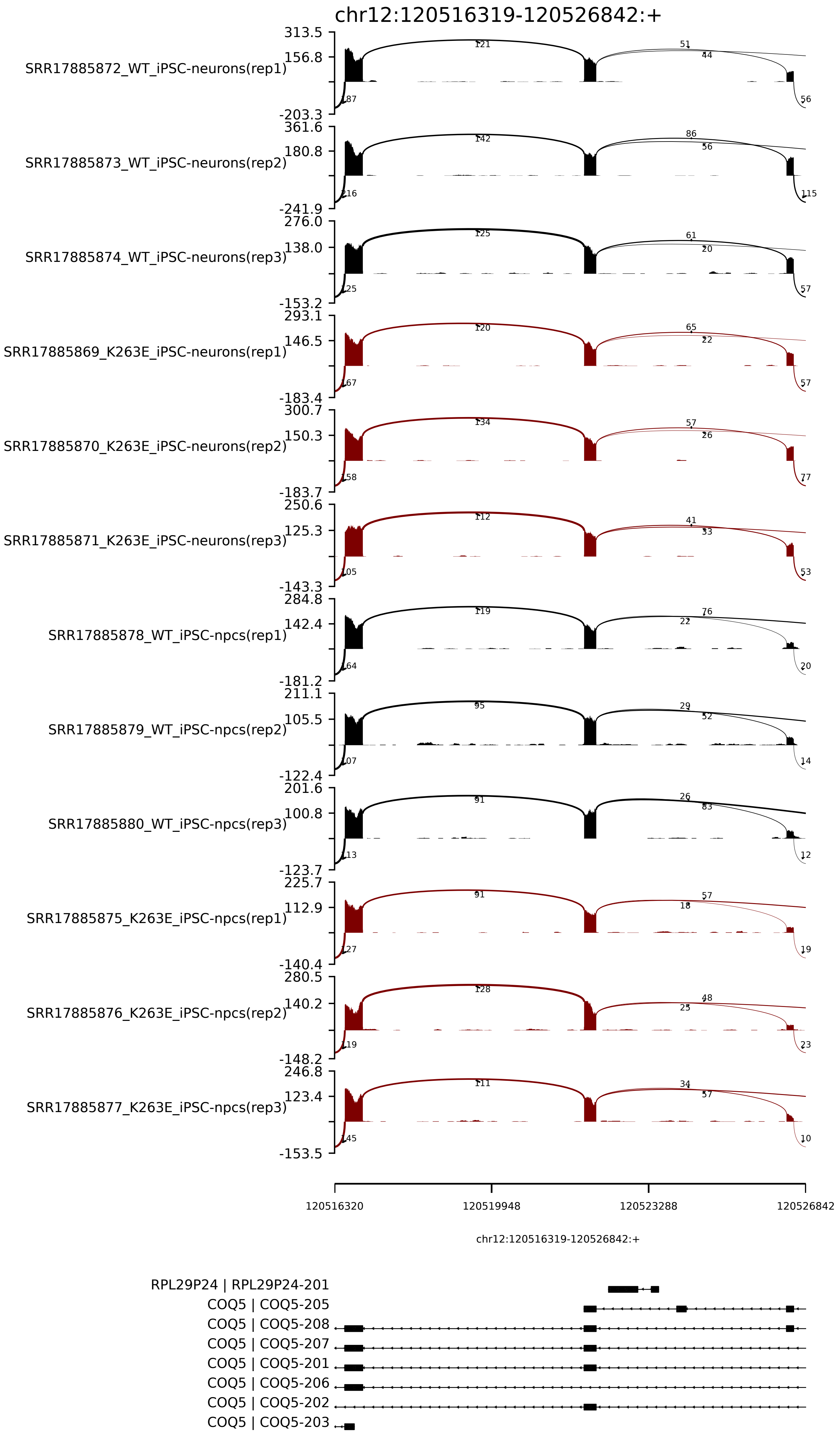
chr12:120516319-120526842:+
SRR17885872_WT_iPSC-neurons(rep1)
SRR17885873_WT_iPSC-neurons(rep2)
SRR17885874_WT_iPSC-neurons(rep3)
SRR17885869_K263E_iPSC-neurons(rep1)
SRR17885870_K263E_iPSC-neurons(rep2)
SRR17885871_K263E_iPSC-neurons(rep3)
SRR17885878_WT_iPSC-npcs(rep1)
SRR17885879_WT_iPSC-npcs(rep2)
SRR17885880_WT_iPSC-npcs(rep3)
SRR17885875_K263E_iPSC-npcs(rep1)
SRR17885876_K263E_iPSC-npcs(rep2)
SRR17885877_K263E_iPSC-npcs(rep3)
120516320
120519948
120523288
120526842
chr12:120516319-120526842:+
RPL29P24 | RPL29P24-201
COQ5 | COQ5-205
COQ5 | COQ5-208
COQ5 | COQ5-207
COQ5 | COQ5-201
COQ5 | COQ5-206
COQ5 | COQ5-202
COQ5 | COQ5-203

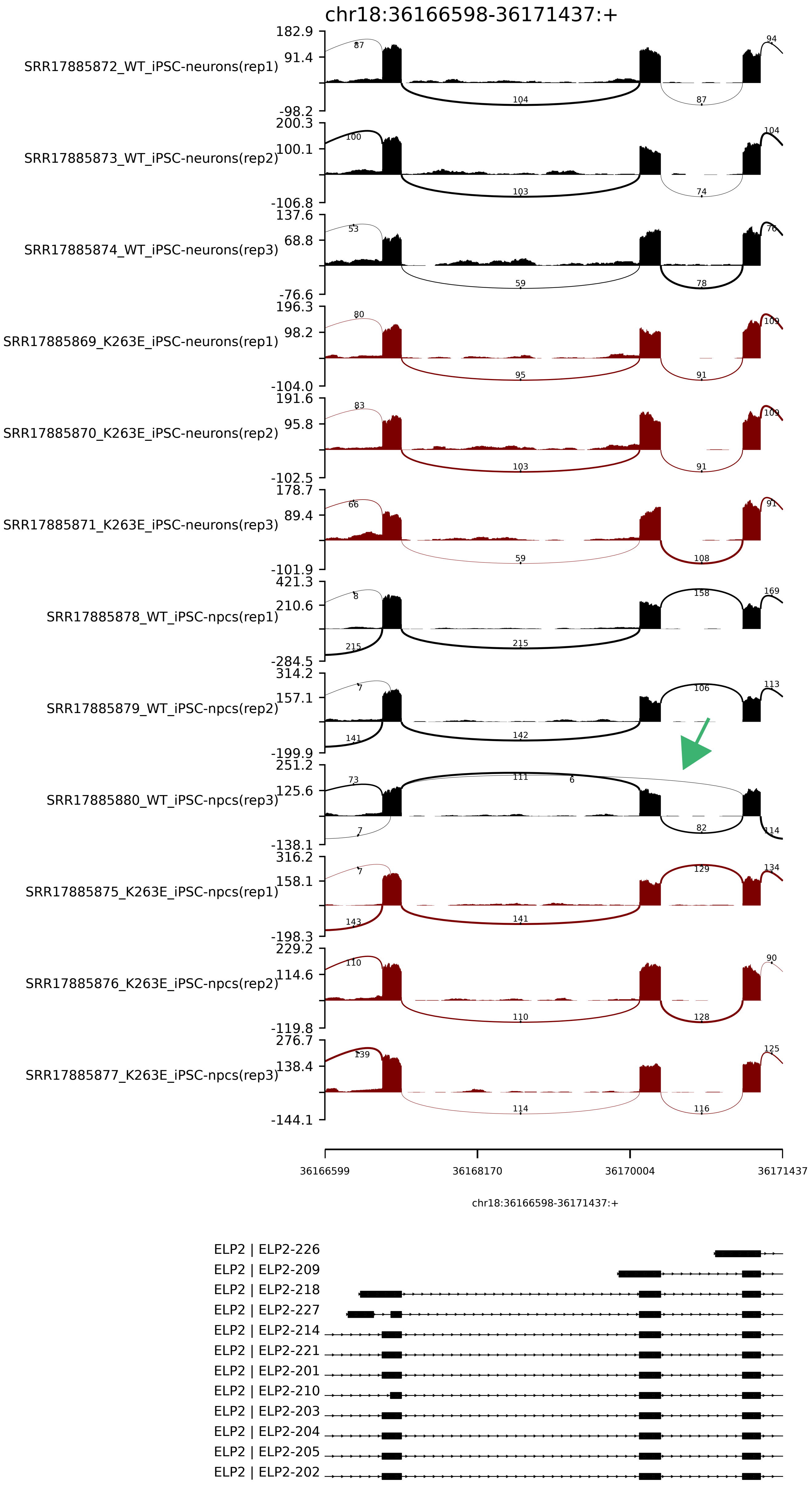
chr18:36166598-36171437:+
SRR17885872_WT_iPSC-neurons(rep1)
SRR17885873_WT_iPSC-neurons(rep2)
SRR17885874_WT_iPSC-neurons(rep3)
SRR17885869_K263E_iPSC-neurons(rep1)
SRR17885870_K263E_iPSC-neurons(rep2)
SRR17885871_K263E_iPSC-neurons(rep3)
SRR17885878_WT_iPSC-npcs(rep1)
SRR17885879_WT_iPSC-npcs(rep2)
SRR17885880_WT_iPSC-npcs(rep3)
SRR17885875_K263E_iPSC-npcs(rep1)
SRR17885876_K263E_iPSC-npcs(rep2)
SRR17885877_K263E_iPSC-npcs(rep3)
36166599
36168170
36170004
36171437
chr18:36166598-36171437:+
ELP2 | ELP2-226
ELP2 | ELP2-209
ELP2 | ELP2-218
ELP2 | ELP2-227
ELP2 | ELP2-214
ELP2 | ELP2-221
ELP2 | ELP2-201
ELP2 | ELP2-210
ELP2 | ELP2-203
ELP2 | ELP2-204
ELP2 | ELP2-205
ELP2 | ELP2-202

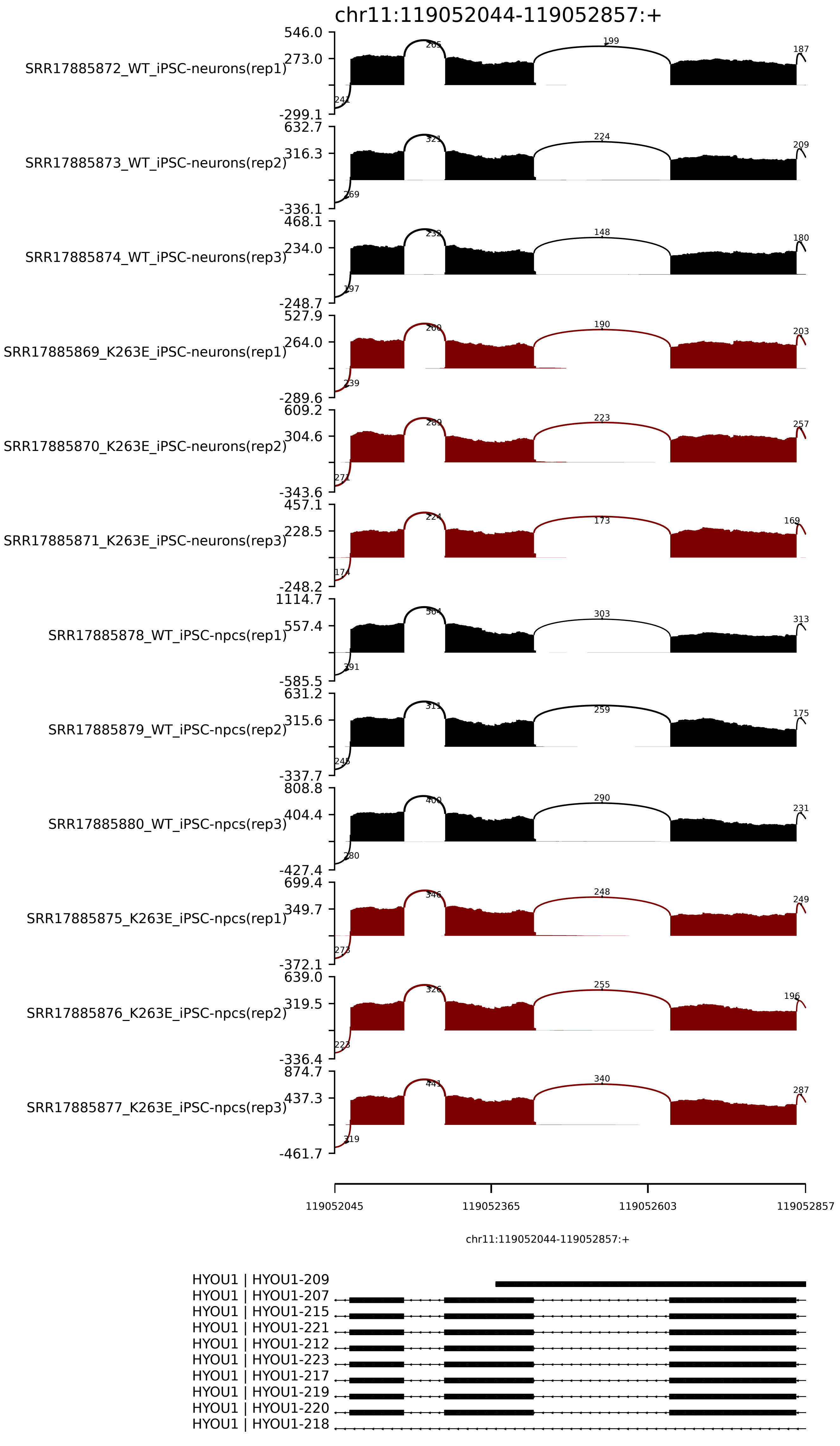

chr11:119052044-119052857:+
SRR17885872_WT_iPSC-neurons(rep1)
SRR17885873_WT_iPSC-neurons(rep2)
SRR17885874_WT_iPSC-neurons(rep3)
SRR17885869_K263E_iPSC-neurons(rep1)
SRR17885870_K263E_iPSC-neurons(rep2)
SRR17885871_K263E_iPSC-neurons(rep3)
SRR17885878_WT_iPSC-npcs(rep1)
SRR17885879_WT_iPSC-npcs(rep2)
SRR17885880_WT_iPSC-npcs(rep3)
SRR17885875_K263E_iPSC-npcs(rep1)
SRR17885876_K263E_iPSC-npcs(rep2)
SRR17885877_K263E_iPSC-npcs(rep3)
119052045
119052365
119052603
119052857
chr11:119052044-119052857:+
HYOU1 | HYOU1-209
HYOU1 | HYOU1-207
HYOU1 | HYOU1-215
HYOU1 | HYOU1-221
HYOU1 | HYOU1-212
HYOU1 | HYOU1-223
HYOU1 | HYOU1-217
HYOU1 | HYOU1-219
HYOU1 | HYOU1-220
HYOU1 | HYOU1-218

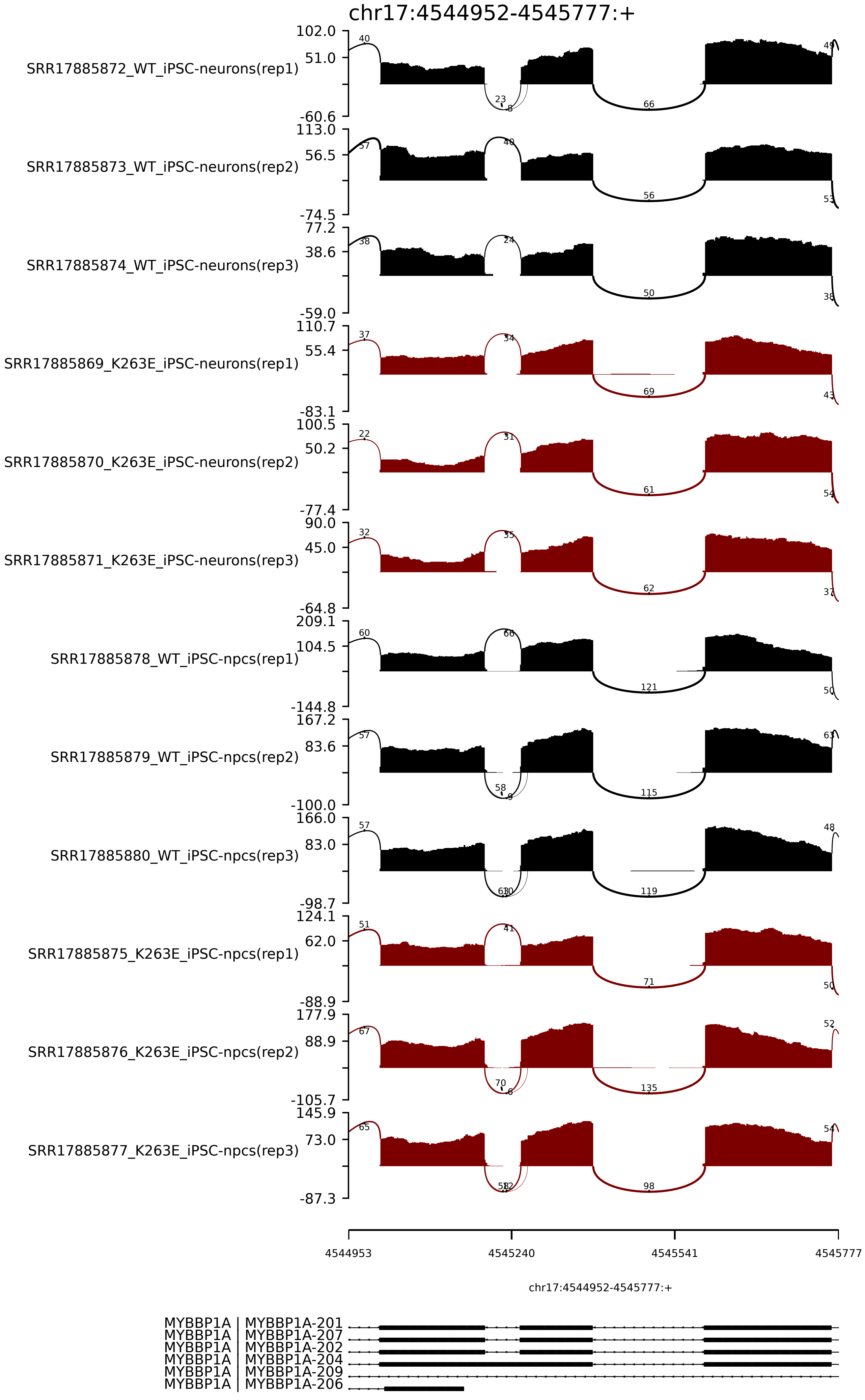
chr17:4544952-4545777:+
SRR17885872_WT_iPSC-neurons(rep1)
102.0
51.0
-60.6
40
23
8
66
49
SRR17885873_WT_iPSC-neurons(rep2)
113.0
56.5
-74.5
57
40
56
53
SRR17885874_WT_iPSC-neurons(rep3)
77.2
38.6
-59.0
38
24
50
38
SRR17885869_K263E_iPSC-neurons(rep1)
110.7
55.4
-83.1
37
34
69
43
SRR17885870_K263E_iPSC-neurons(rep2)
100.5
50.2
-77.4
22
31
61
54
SRR17885871_K263E_iPSC-neurons(rep3)
90.0
45.0
-64.8
32
35
62
37
SRR17885878_WT_iPSC-npcs(rep1)
209.1
104.5
-144.8
60
66
121
50
SRR17885879_WT_iPSC-npcs(rep2)
167.2
83.6
-100.0
57
58
9
115
63
SRR17885880_WT_iPSC-npcs(rep3)
166.0
83.0
-98.7
57
119
48
SRR17885875_K263E_iPSC-npcs(rep1)
124.1
62.0
-88.9
51
41
71
50
SRR17885876_K263E_iPSC-npcs(rep2)
177.9
88.9
-105.7
67
70
6
135
52
SRR17885877_K263E_iPSC-npcs(rep3)
145.9
73.0
-87.3
65
98
54
4544953
4545240
4545541
4545777
chr17:4544952-4545777:+
MYBBP1A | MYBBP1A-201
MYBBP1A | MYBBP1A-207
MYBBP1A | MYBBP1A-202
MYBBP1A | MYBBP1A-204
MYBBP1A | MYBBP1A-209
MYBBP1A | MYBBP1A-206

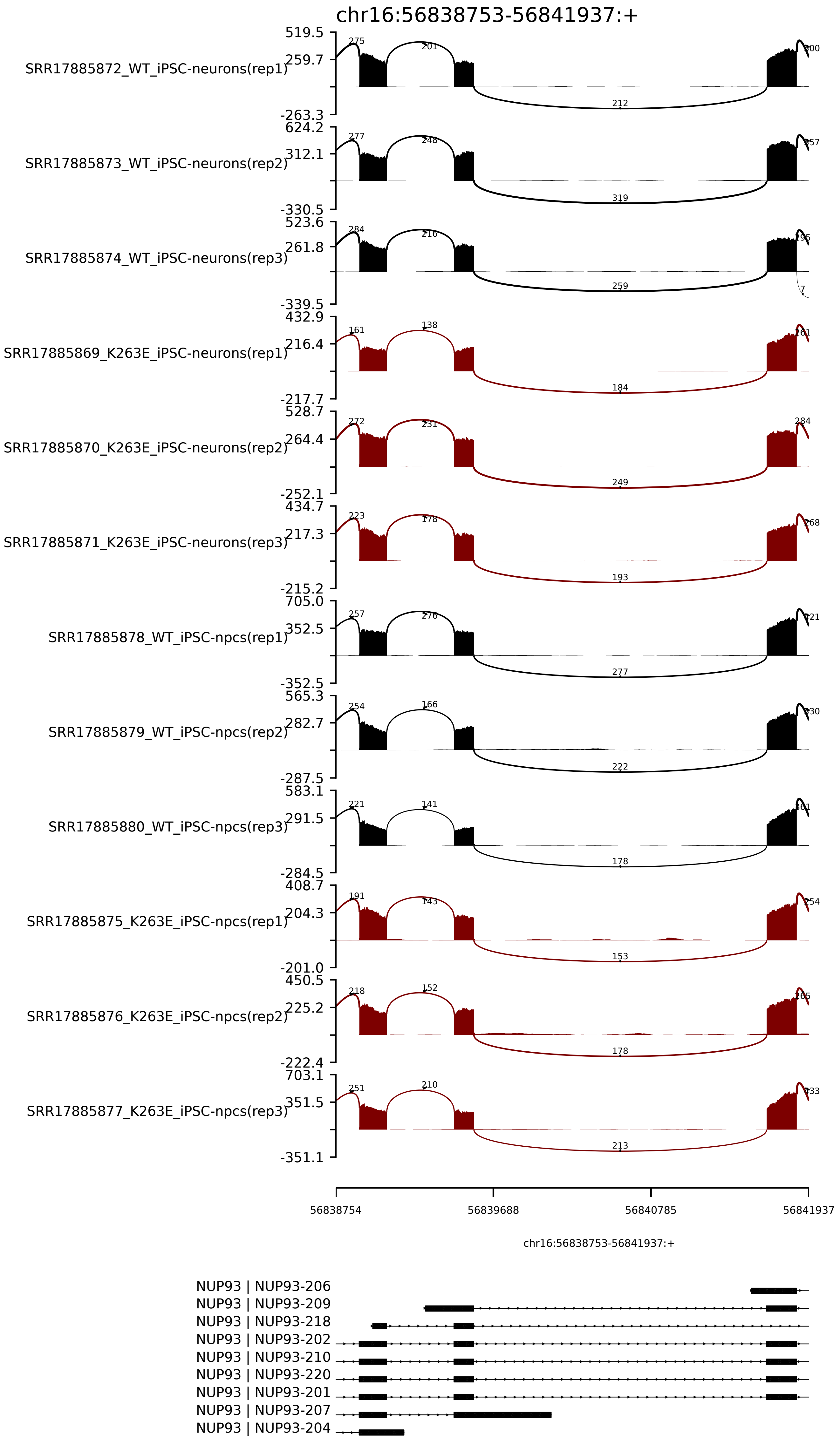

chr16:56838753-56841937:+
SRR17885872_WT_iPSC-neurons(rep1)
SRR17885873_WT_iPSC-neurons(rep2)
SRR17885874_WT_iPSC-neurons(rep3)
SRR17885869_K263E_iPSC-neurons(rep1)
SRR17885870_K263E_iPSC-neurons(rep2)
SRR17885871_K263E_iPSC-neurons(rep3)
SRR17885878_WT_iPSC-npcs(rep1)
SRR17885879_WT_iPSC-npcs(rep2)
SRR17885880_WT_iPSC-npcs(rep3)
SRR17885875_K263E_iPSC-npcs(rep1)
SRR17885876_K263E_iPSC-npcs(rep2)
SRR17885877_K263E_iPSC-npcs(rep3)
56838754
56839688
56840785
56841937
chr16:56838753-56841937:+
NUP93 | NUP93-206
NUP93 | NUP93-209
NUP93 | NUP93-218
NUP93 | NUP93-202
NUP93 | NUP93-210
NUP93 | NUP93-220
NUP93 | NUP93-201
NUP93 | NUP93-207
NUP93 | NUP93-204

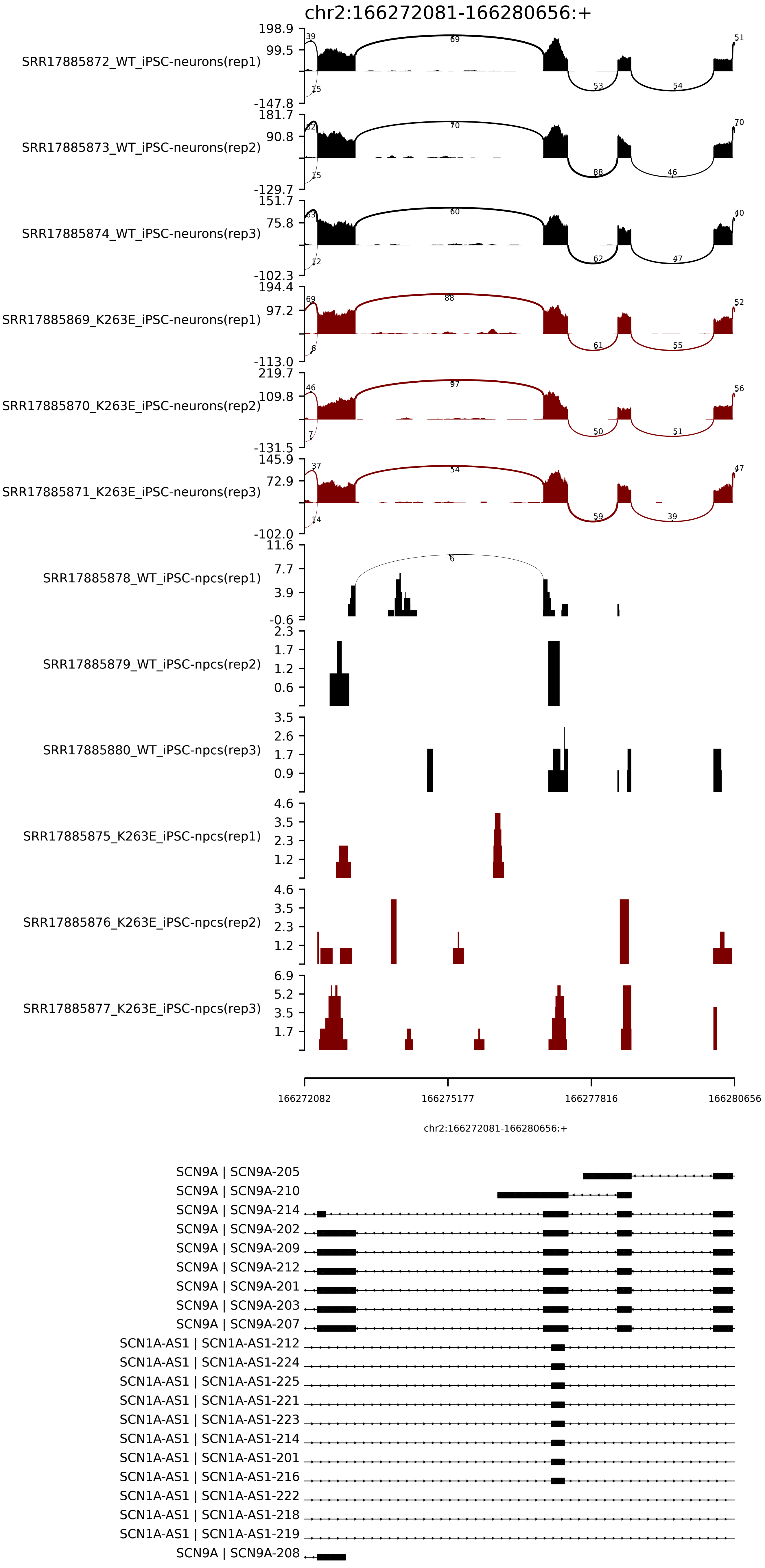
chr2:166272081-166280656:+
SRR17885872_WT_iPSC-neurons(rep1)
SRR17885873_WT_iPSC-neurons(rep2)
SRR17885874_WT_iPSC-neurons(rep3)
SRR17885869_K263E_iPSC-neurons(rep1)
SRR17885870_K263E_iPSC-neurons(rep2)
SRR17885871_K263E_iPSC-neurons(rep3)
SRR17885878_WT_iPSC-npcs(rep1)
SRR17885879_WT_iPSC-npcs(rep2)
SRR17885880_WT_iPSC-npcs(rep3)
SRR17885875_K263E_iPSC-npcs(rep1)
SRR17885876_K263E_iPSC-npcs(rep2)
SRR17885877_K263E_iPSC-npcs(rep3)
166272082
166275177
166277816
166280656
chr2:166272081-166280656:+
SCN9A | SCN9A-205
SCN9A | SCN9A-210
SCN9A | SCN9A-214
SCN9A | SCN9A-202
SCN9A | SCN9A-209
SCN9A | SCN9A-212
SCN9A | SCN9A-201
SCN9A | SCN9A-203
SCN9A | SCN9A-207
SCN1A-AS1 | SCN1A-AS1-212
SCN1A-AS1 | SCN1A-AS1-224
SCN1A-AS1 | SCN1A-AS1-225
SCN1A-AS1 | SCN1A-AS1-221
SCN1A-AS1 | SCN1A-AS1-223
SCN1A-AS1 | SCN1A-AS1-214
SCN1A-AS1 | SCN1A-AS1-201
SCN1A-AS1 | SCN1A-AS1-216
SCN1A-AS1 | SCN1A-AS1-222
SCN1A-AS1 | SCN1A-AS1-218
SCN1A-AS1 | SCN1A-AS1-219
SCN9A | SCN9A-208

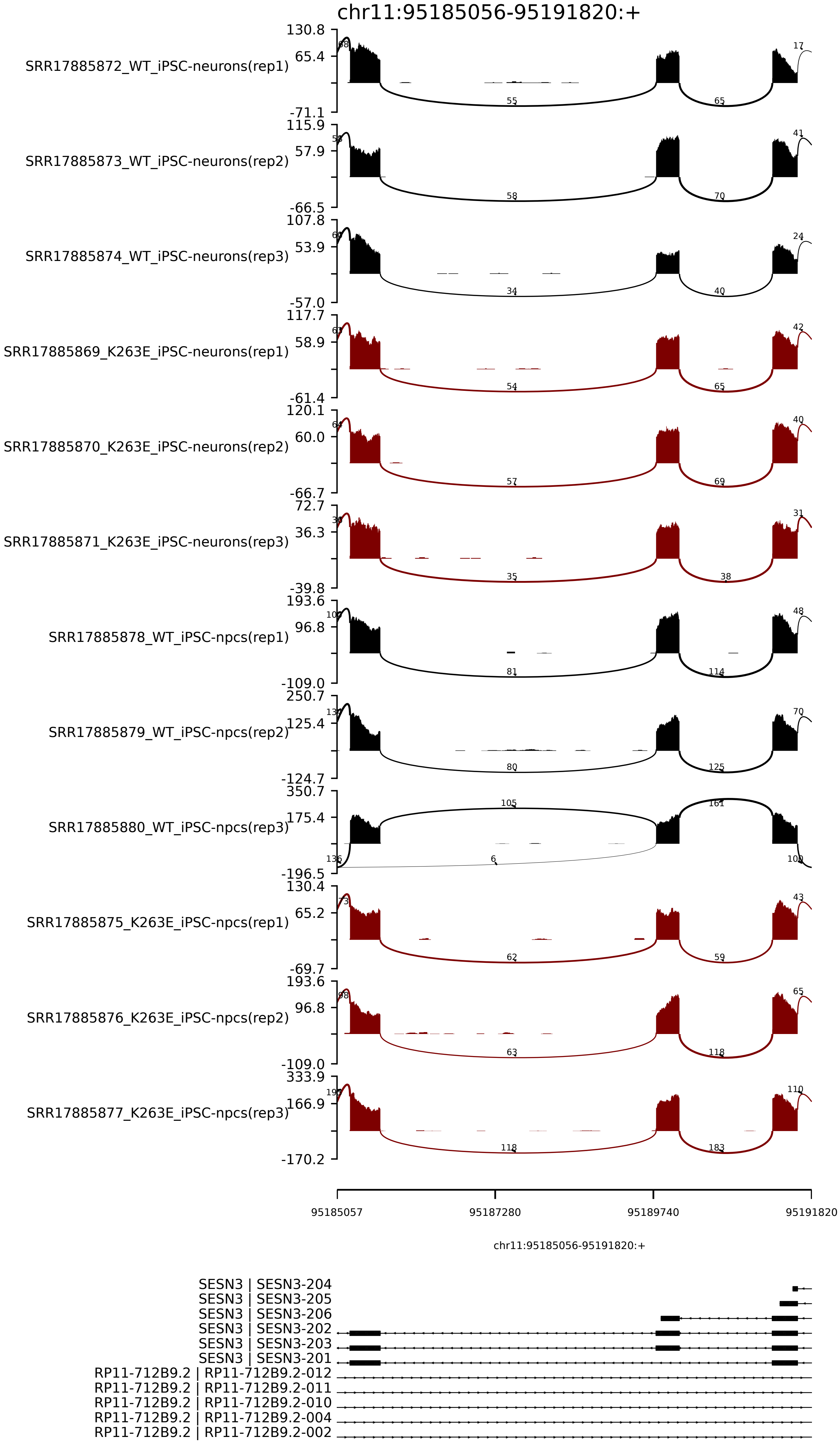
chr11:95185056-95191820:+
SRR17885872_WT_iPSC-neurons(rep1)
SRR17885873_WT_iPSC-neurons(rep2)
SRR17885874_WT_iPSC-neurons(rep3)
SRR17885869_K263E_iPSC-neurons(rep1)
SRR17885870_K263E_iPSC-neurons(rep2)
SRR17885871_K263E_iPSC-neurons(rep3)
SRR17885878_WT_iPSC-npcs(rep1)
SRR17885879_WT_iPSC-npcs(rep2)
SRR17885880_WT_iPSC-npcs(rep3)
SRR17885875_K263E_iPSC-npcs(rep1)
SRR17885876_K263E_iPSC-npcs(rep2)
SRR17885877_K263E_iPSC-npcs(rep3)
130.8
65.4
-71.1
115.9
57.9
-66.5
107.8
53.9
-57.0
117.7
58.9
-61.4
120.1
60.0
-66.7
72.7
36.3
-39.8
193.6
96.8
-109.0
250.7
125.4
-124.7
350.7
175.4
-196.5
130.4
65.2
-69.7
193.6
96.8
-109.0
333.9
166.9
-170.2
95185057
95187280
95189740
95191820
chr11:95185056-95191820:+
SESN3 | SESN3-204
SESN3 | SESN3-205
SESN3 | SESN3-206
SESN3 | SESN3-202
SESN3 | SESN3-203
SESN3 | SESN3-201
RP11-712B9.2 | RP11-712B9.2-012
RP11-712B9.2 | RP11-712B9.2-011
RP11-712B9.2 | RP11-712B9.2-010
RP11-712B9.2 | RP11-712B9.2-004
RP11-712B9.2 | RP11-712B9.2-002

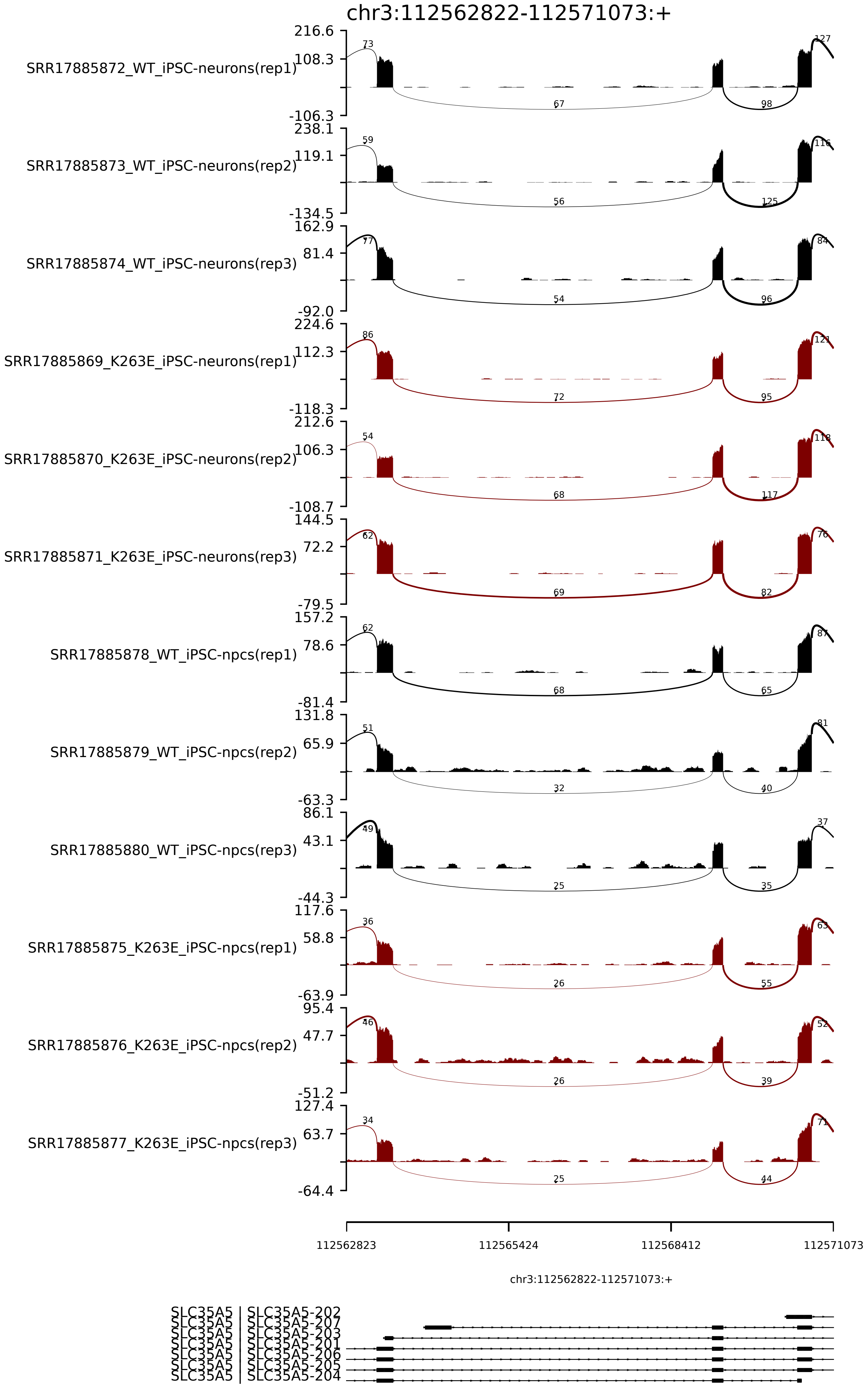

chr3:112562822-112571073:+
SRR17885872_WT_iPSC-neurons(rep1)
SRR17885873_WT_iPSC-neurons(rep2)
SRR17885874_WT_iPSC-neurons(rep3)
SRR17885869_K263E_iPSC-neurons(rep1)
SRR17885870_K263E_iPSC-neurons(rep2)
SRR17885871_K263E_iPSC-neurons(rep3)
SRR17885878_WT_iPSC-npcs(rep1)
SRR17885879_WT_iPSC-npcs(rep2)
SRR17885880_WT_iPSC-npcs(rep3)
SRR17885875_K263E_iPSC-npcs(rep1)
SRR17885876_K263E_iPSC-npcs(rep2)
SRR17885877_K263E_iPSC-npcs(rep3)
112562823
112565424
112568412
112571073
chr3:112562822-112571073:+
SLC35A5 | SLC35A5-202
SLC35A5 | SLC35A5-207
SLC35A5 | SLC35A5-203
SLC35A5 | SLC35A5-201
SLC35A5 | SLC35A5-206
SLC35A5 | SLC35A5-205
SLC35A5 | SLC35A5-204

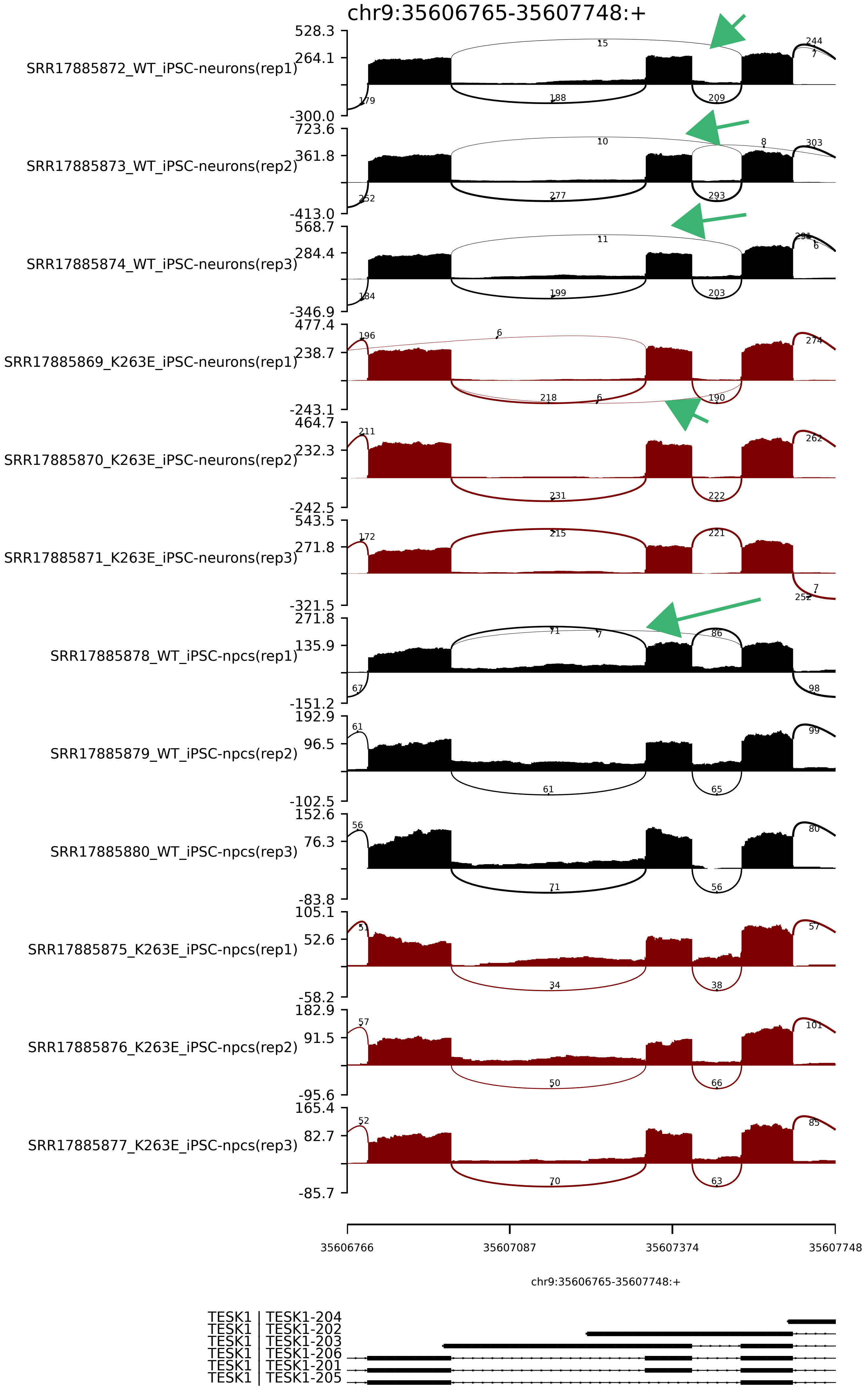

chr9:35606765-35607748:+
SRR17885872_WT_iPSC-neurons(rep1)
SRR17885873_WT_iPSC-neurons(rep2)
SRR17885874_WT_iPSC-neurons(rep3)
SRR17885869_K263E_iPSC-neurons(rep1)
SRR17885870_K263E_iPSC-neurons(rep2)
SRR17885871_K263E_iPSC-neurons(rep3)
SRR17885878_WT_iPSC-npcs(rep1)
SRR17885879_WT_iPSC-npcs(rep2)
SRR17885880_WT_iPSC-npcs(rep3)
SRR17885875_K263E_iPSC-npcs(rep1)
SRR17885876_K263E_iPSC-npcs(rep2)
SRR17885877_K263E_iPSC-npcs(rep3)
35606766
35607087
35607374
35607748
chr9:35606765-35607748:+
TESK1 | TESK1-204
TESK1 | TESK1-202
TESK1 | TESK1-203
TESK1 | TESK1-206
TESK1 | TESK1-201
TESK1 | TESK1-205

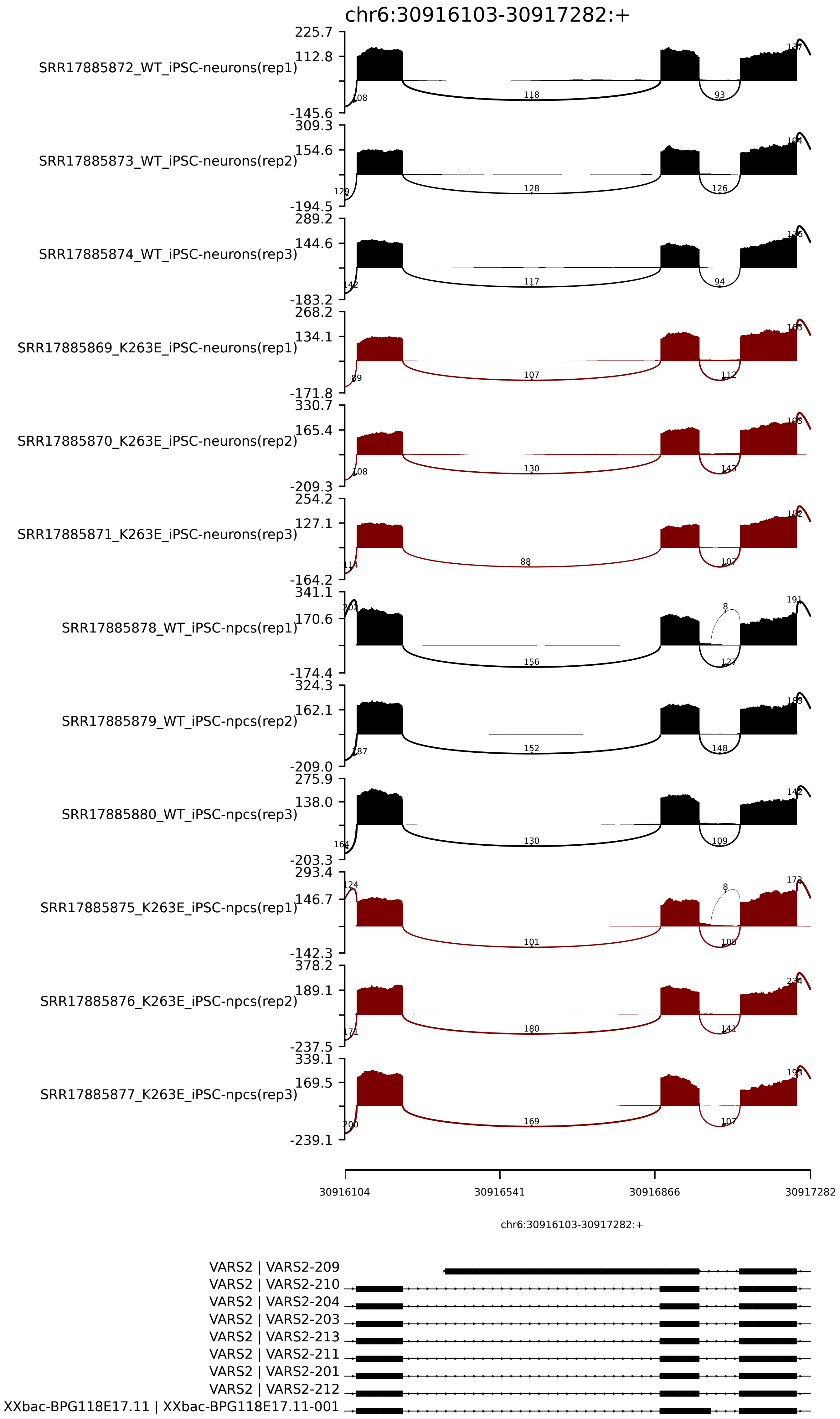
chr6:30916103-30917282:+
SRR17885872_WT_iPSC-neurons(rep1)
225.7
112.8
-145.6
108
118
93
137
SRR17885873_WT_iPSC-neurons(rep2)
309.3
154.6
-194.5
129
128
126
194
SRR17885874_WT_iPSC-neurons(rep3)
289.2
144.6
-183.2
142
117
94
176
SRR17885869_K263E_iPSC-neurons(rep1)
268.2
134.1
-171.8
89
107
112
163
SRR17885870_K263E_iPSC-neurons(rep2)
330.7
165.4
-209.3
108
130
143
193
SRR17885871_K263E_iPSC-neurons(rep3)
254.2
127.1
-164.2
114
88
107
152
SRR17885878_WT_iPSC-npcs(rep1)
341.1
170.6
-174.4
202
8
191
156
127
SRR17885879_WT_iPSC-npcs(rep2)
324.3
162.1
-209.0
187
152
148
183
SRR17885880_WT_iPSC-npcs(rep3)
275.9
138.0
-203.3
164
130
109
142
SRR17885875_K263E_iPSC-npcs(rep1)
293.4
146.7
-142.3
124
8
172
101
105
SRR17885876_K263E_iPSC-npcs(rep2)
378.2
189.1
-237.5
171
180
141
234
SRR17885877_K263E_iPSC-npcs(rep3)
339.1
169.5
-239.1
200
169
107
193
30916104
30916541
30916866
30917282
chr6:30916103-30917282:+
VARS2 | VARS2-209
VARS2 | VARS2-210
VARS2 | VARS2-204
VARS2 | VARS2-203
VARS2 | VARS2-213
VARS2 | VARS2-211
VARS2 | VARS2-201
VARS2 | VARS2-212
XXbac-BPG118E17.11 | XXbac-BPG118E17.11-001

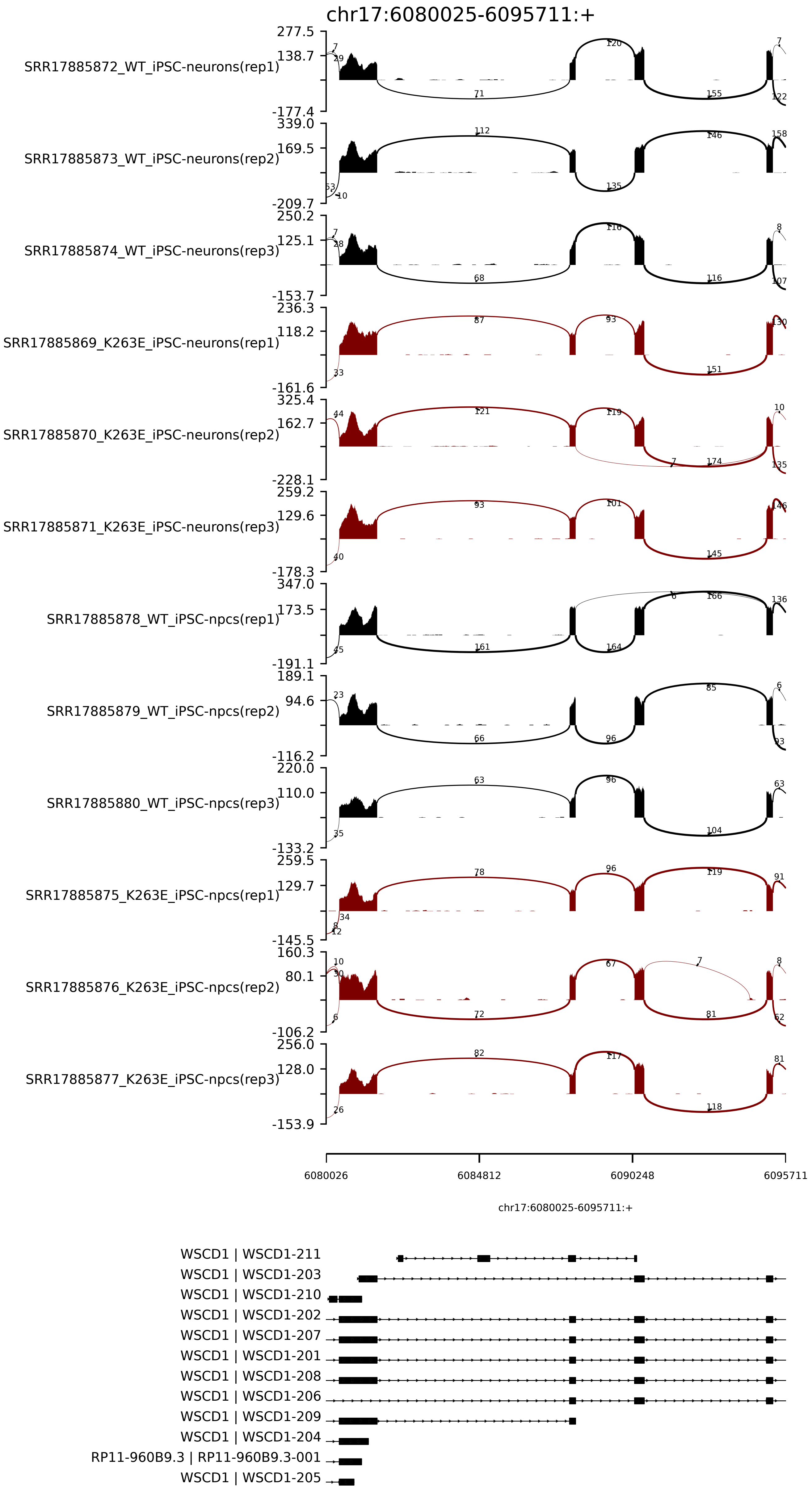
chr17:6080025-6095711:+
SRR17885872_WT_iPSC-neurons(rep1)
SRR17885873_WT_iPSC-neurons(rep2)
SRR17885874_WT_iPSC-neurons(rep3)
SRR17885869_K263E_iPSC-neurons(rep1)
SRR17885870_K263E_iPSC-neurons(rep2)
SRR17885871_K263E_iPSC-neurons(rep3)
SRR17885878_WT_iPSC-npcs(rep1)
SRR17885879_WT_iPSC-npcs(rep2)
SRR17885880_WT_iPSC-npcs(rep3)
SRR17885875_K263E_iPSC-npcs(rep1)
SRR17885876_K263E_iPSC-npcs(rep2)
SRR17885877_K263E_iPSC-npcs(rep3)
6080026
6084812
6090248
6095711
chr17:6080025-6095711:+
WSCD1 | WSCD1-211
WSCD1 | WSCD1-203
WSCD1 | WSCD1-210
WSCD1 | WSCD1-202
WSCD1 | WSCD1-207
WSCD1 | WSCD1-201
WSCD1 | WSCD1-208
WSCD1 | WSCD1-206
WSCD1 | WSCD1-209
WSCD1 | WSCD1-204
RP11-960B9.3 | RP11-960B9.3-001
WSCD1 | WSCD1-205

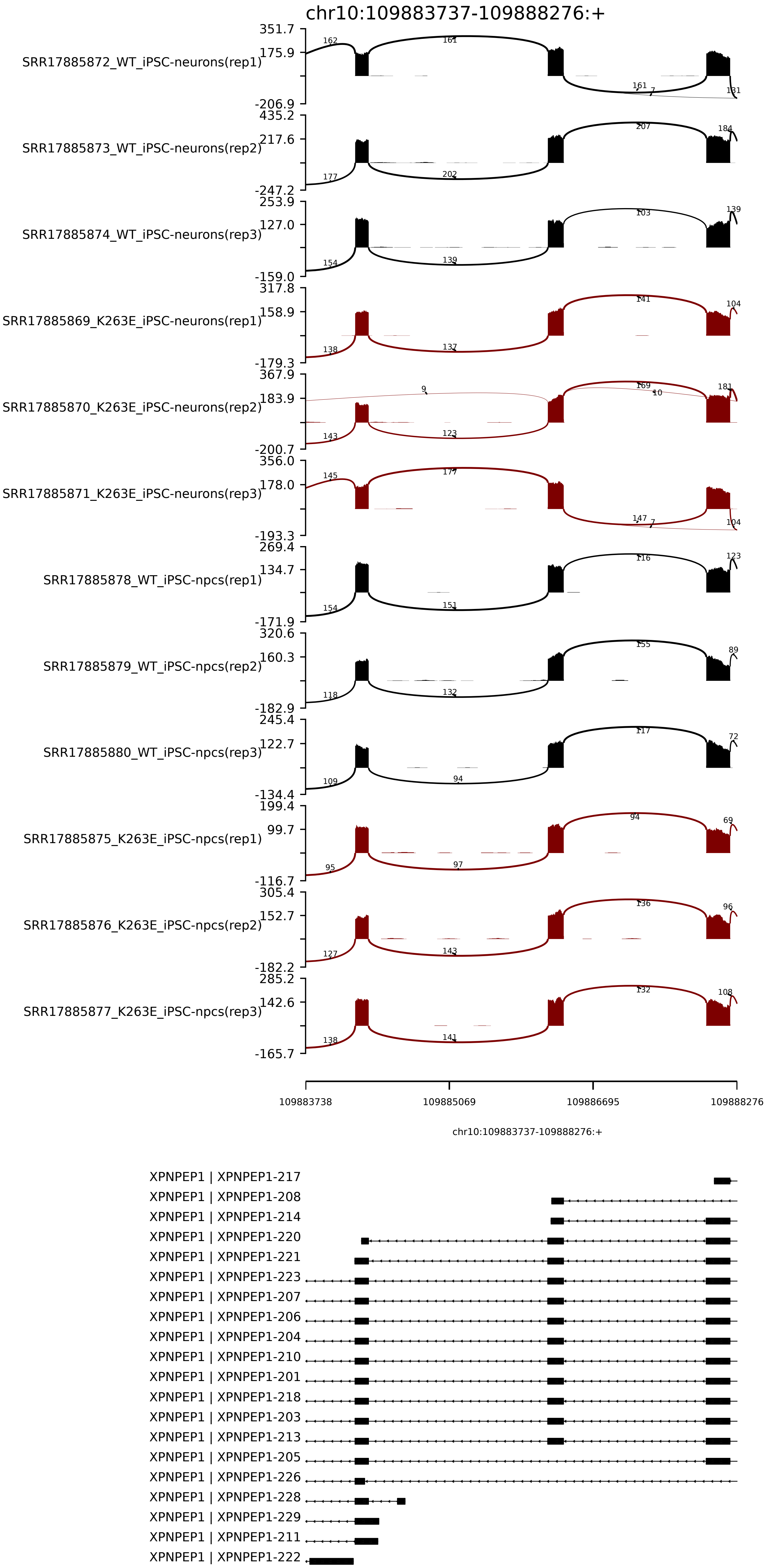
chr10:109883737-109888276:+
SRR17885872_WT_iPSC-neurons(rep1)
SRR17885873_WT_iPSC-neurons(rep2)
SRR17885874_WT_iPSC-neurons(rep3)
SRR17885869_K263E_iPSC-neurons(rep1)
SRR17885870_K263E_iPSC-neurons(rep2)
SRR17885871_K263E_iPSC-neurons(rep3)
SRR17885878_WT_iPSC-npcs(rep1)
SRR17885879_WT_iPSC-npcs(rep2)
SRR17885880_WT_iPSC-npcs(rep3)
SRR17885875_K263E_iPSC-npcs(rep1)
SRR17885876_K263E_iPSC-npcs(rep2)
SRR17885877_K263E_iPSC-npcs(rep3)
109883738
109885069
109886695
109888276
chr10:109883737-109888276:+
XPNPEP1 | XPNPEP1-217
XPNPEP1 | XPNPEP1-208
XPNPEP1 | XPNPEP1-214
XPNPEP1 | XPNPEP1-220
XPNPEP1 | XPNPEP1-221
XPNPEP1 | XPNPEP1-223
XPNPEP1 | XPNPEP1-207
XPNPEP1 | XPNPEP1-206
XPNPEP1 | XPNPEP1-204
XPNPEP1 | XPNPEP1-210
XPNPEP1 | XPNPEP1-201
XPNPEP1 | XPNPEP1-218
XPNPEP1 | XPNPEP1-203
XPNPEP1 | XPNPEP1-213
XPNPEP1 | XPNPEP1-205
XPNPEP1 | XPNPEP1-226
XPNPEP1 | XPNPEP1-228
XPNPEP1 | XPNPEP1-229
XPNPEP1 | XPNPEP1-211
XPNPEP1 | XPNPEP1-222

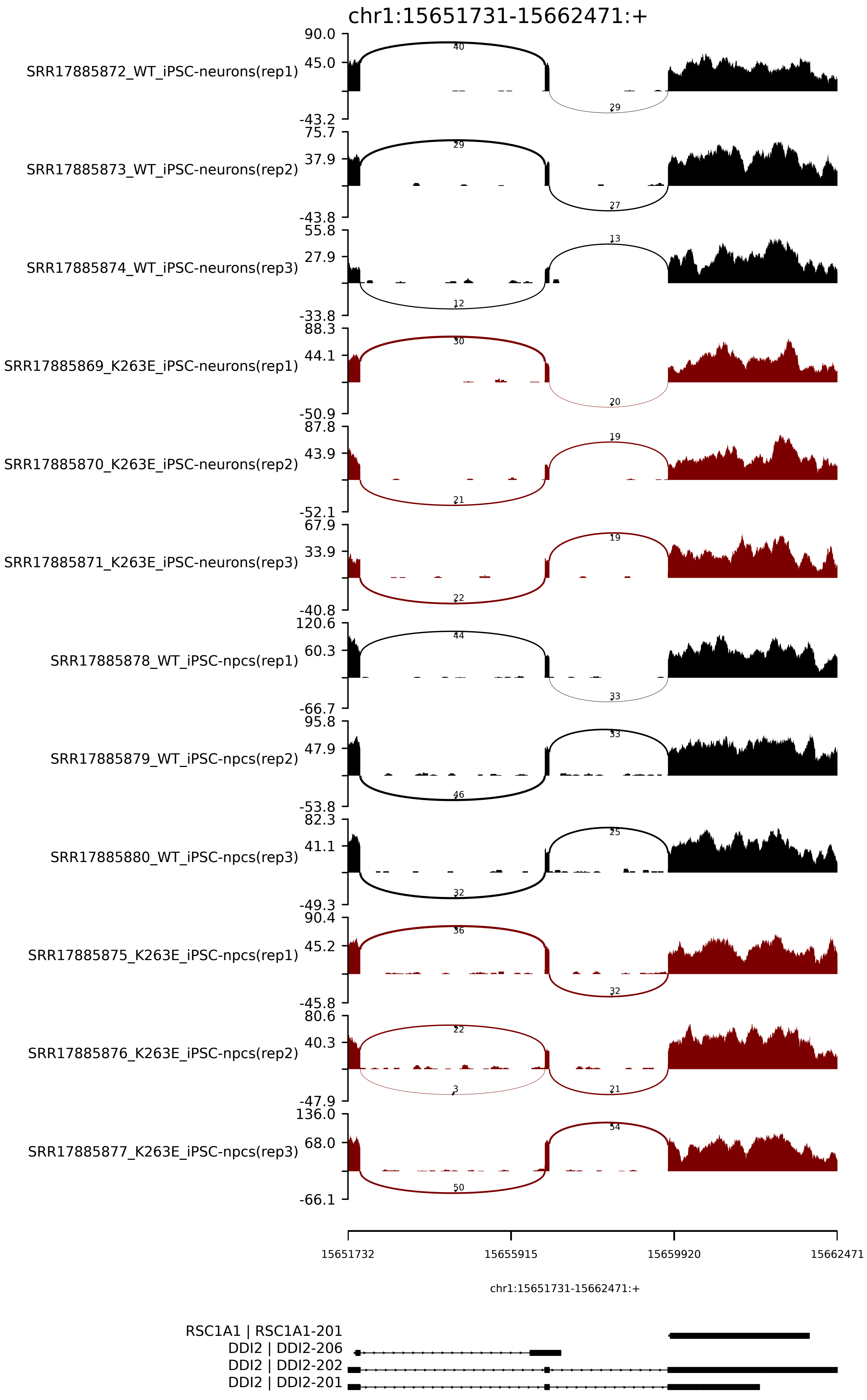

chr1:15651731-15662471:+
SRR17885872_WT_iPSC-neurons(rep1)
SRR17885873_WT_iPSC-neurons(rep2)
SRR17885874_WT_iPSC-neurons(rep3)
SRR17885869_K263E_iPSC-neurons(rep1)
SRR17885870_K263E_iPSC-neurons(rep2)
SRR17885871_K263E_iPSC-neurons(rep3)
SRR17885878_WT_iPSC-npcs(rep1)
SRR17885879_WT_iPSC-npcs(rep2)
SRR17885880_WT_iPSC-npcs(rep3)
SRR17885875_K263E_iPSC-npcs(rep1)
SRR17885876_K263E_iPSC-npcs(rep2)
SRR17885877_K263E_iPSC-npcs(rep3)
15651732
15655915
15659920
15662471
chr1:15651731-15662471:+
RSC1A1 | RSC1A1-201
DDI2 | DDI2-206
DDI2 | DDI2-202
DDI2 | DDI2-201

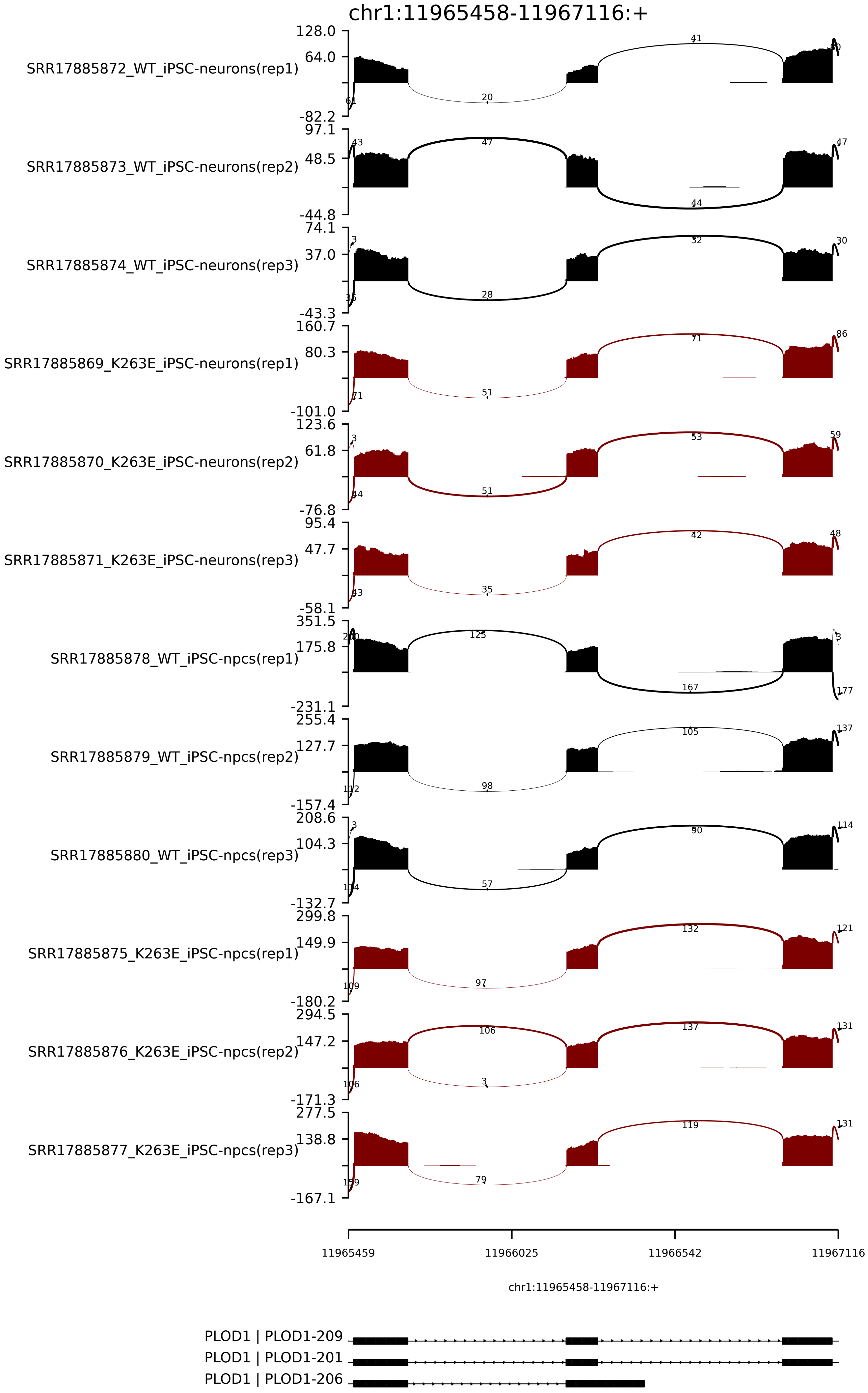
chr1:11965458-11967116:+
SRR17885872_WT_iPSC-neurons(rep1)
128.0
64.0
-82.2
41
30
61
20
SRR17885873_WT_iPSC-neurons(rep2)
97.1
48.5
-44.8
43
47
47
44
SRR17885874_WT_iPSC-neurons(rep3)
74.1
37.0
-43.3
3
32
30
36
28
SRR17885869_K263E_iPSC-neurons(rep1)
160.7
80.3
-101.0
71
86
71
51
SRR17885870_K263E_iPSC-neurons(rep2)
123.6
61.8
-76.8
3
53
59
44
51
SRR17885871_K263E_iPSC-neurons(rep3)
95.4
47.7
-58.1
42
48
43
35
SRR17885878_WT_iPSC-npcs(rep1)
351.5
175.8
-231.1
210
125
3
167
177
SRR17885879_WT_iPSC-npcs(rep2)
255.4
127.7
-157.4
105
137
112
98
SRR17885880_WT_iPSC-npcs(rep3)
208.6
104.3
-132.7
3
90
114
114
57
SRR17885875_K263E_iPSC-npcs(rep1)
299.8
149.9
-180.2
132
121
109
97
SRR17885876_K263E_iPSC-npcs(rep2)
294.5
147.2
-171.3
106
137
131
106
3
SRR17885877_K263E_iPSC-npcs(rep3)
277.5
138.8
-167.1
119
131
159
79
11965459
11966025
11966542
11967116
chr1:11965458-11967116:+
PLOD1 | PLOD1-209
PLOD1 | PLOD1-201
PLOD1 | PLOD1-206

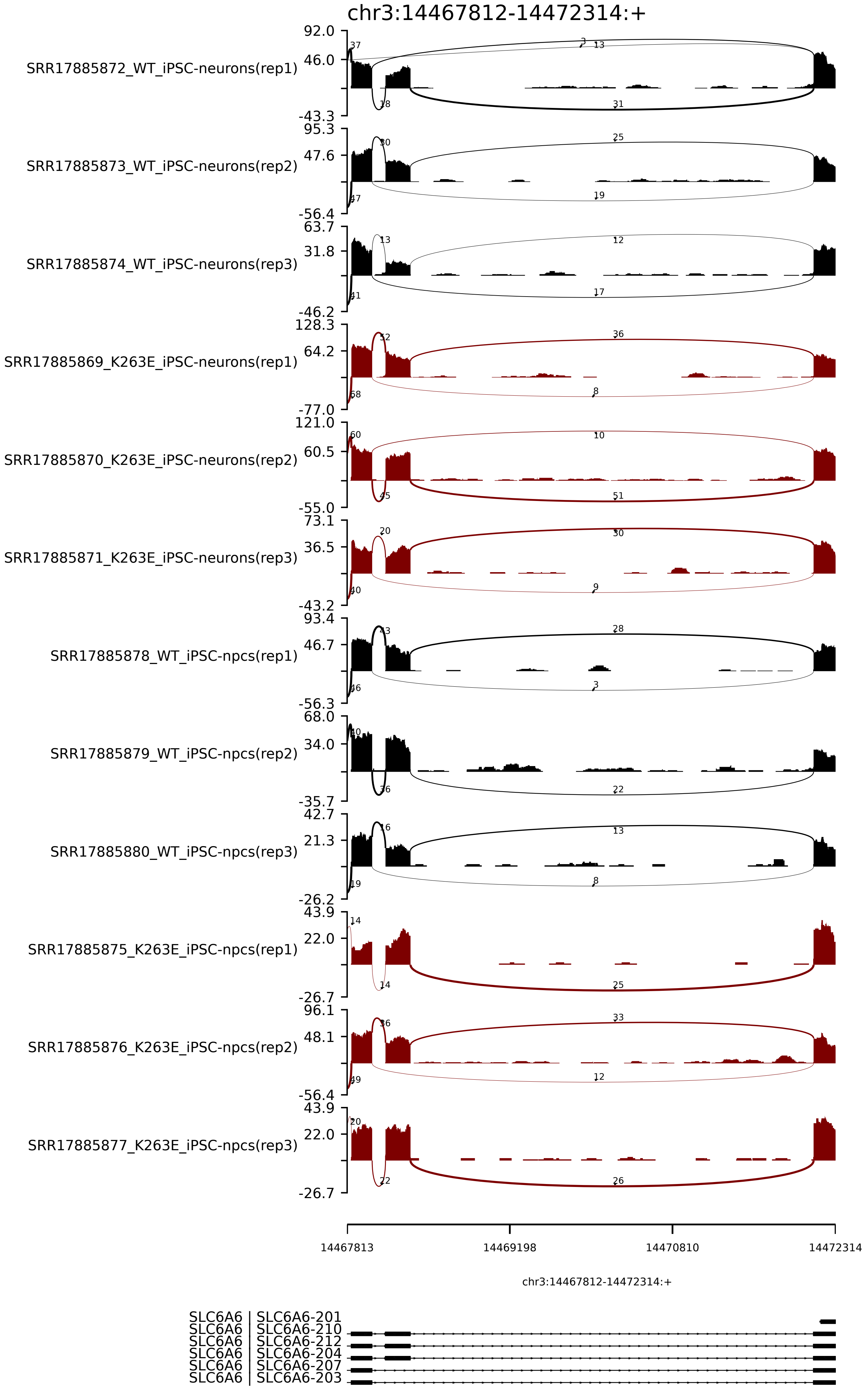
chr3:14467812-14472314:+
SRR17885872_WT_iPSC-neurons(rep1)
SRR17885873_WT_iPSC-neurons(rep2)
SRR17885874_WT_iPSC-neurons(rep3)
SRR17885869_K263E_iPSC-neurons(rep1)
SRR17885870_K263E_iPSC-neurons(rep2)
SRR17885871_K263E_iPSC-neurons(rep3)
SRR17885878_WT_iPSC-npcs(rep1)
SRR17885879_WT_iPSC-npcs(rep2)
SRR17885880_WT_iPSC-npcs(rep3)
SRR17885875_K263E_iPSC-npcs(rep1)
SRR17885876_K263E_iPSC-npcs(rep2)
SRR17885877_K263E_iPSC-npcs(rep3)
14467813
14469198
14470810
14472314
chr3:14467812-14472314:+
SLC6A6 | SLC6A6-201
SLC6A6 | SLC6A6-210
SLC6A6 | SLC6A6-212
SLC6A6 | SLC6A6-204
SLC6A6 | SLC6A6-207
SLC6A6 | SLC6A6-203

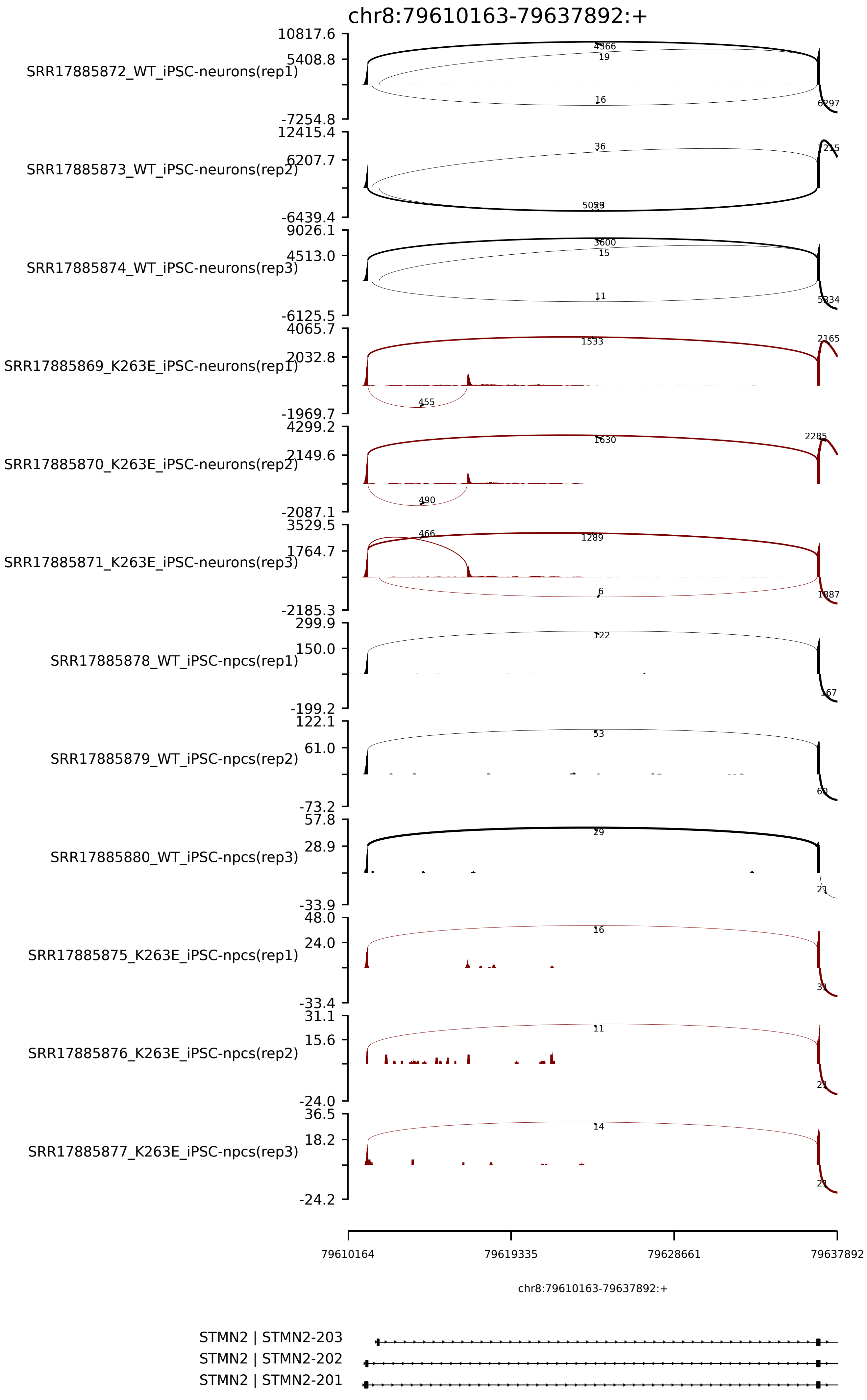

chr8:79610163-79637892:+
SRR17885872_WT_iPSC-neurons(rep1)
SRR17885873_WT_iPSC-neurons(rep2)
SRR17885874_WT_iPSC-neurons(rep3)
SRR17885869_K263E_iPSC-neurons(rep1)
SRR17885870_K263E_iPSC-neurons(rep2)
SRR17885871_K263E_iPSC-neurons(rep3)
SRR17885878_WT_iPSC-npcs(rep1)
SRR17885879_WT_iPSC-npcs(rep2)
SRR17885880_WT_iPSC-npcs(rep3)
SRR17885875_K263E_iPSC-npcs(rep1)
SRR17885876_K263E_iPSC-npcs(rep2)
SRR17885877_K263E_iPSC-npcs(rep3)
79610164
79619335
79628661
79637892
chr8:79610163-79637892:+
STMN2 | STMN2-203
STMN2 | STMN2-202
STMN2 | STMN2-201

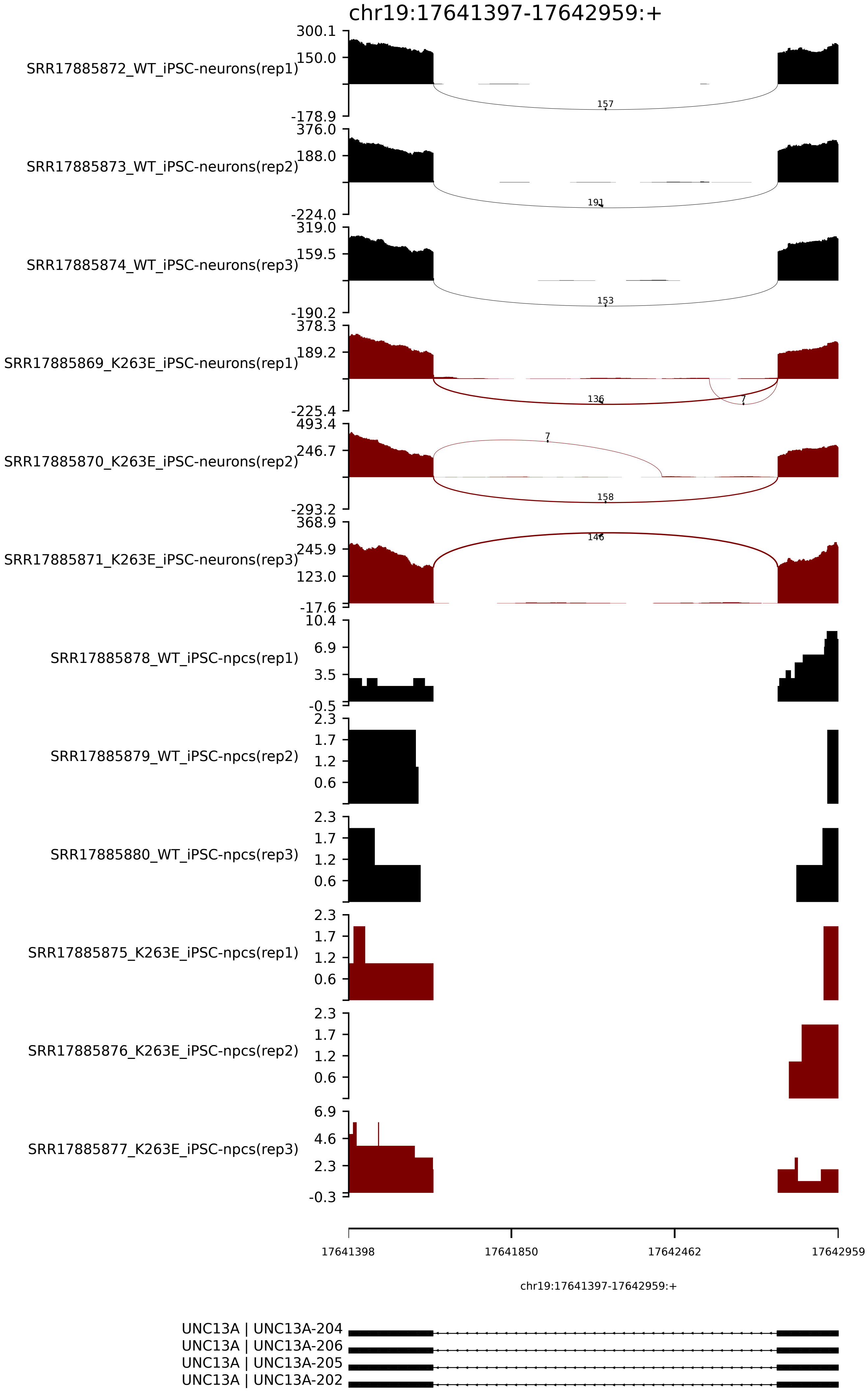
chr19:17641397-17642959:+
SRR17885872_WT_iPSC-neurons(rep1)
300.1
150.0
-178.9
157
SRR17885873_WT_iPSC-neurons(rep2)
376.0
188.0
-224.0
191
SRR17885874_WT_iPSC-neurons(rep3)
319.0
159.5
-190.2
153
SRR17885869_K263E_iPSC-neurons(rep1)
378.3
189.2
-225.4
136
7
SRR17885870_K263E_iPSC-neurons(rep2)
493.4
246.7
-293.2
7
158
SRR17885871_K263E_iPSC-neurons(rep3)
368.9
245.9
123.0
-17.6
146
SRR17885878_WT_iPSC-npcs(rep1)
10.4
6.9
3.5
-0.5
SRR17885879_WT_iPSC-npcs(rep2)
2.3
1.7
1.2
0.6
SRR17885880_WT_iPSC-npcs(rep3)
2.3
1.7
1.2
0.6
SRR17885875_K263E_iPSC-npcs(rep1)
2.3
1.7
1.2
0.6
SRR17885876_K263E_iPSC-npcs(rep2)
2.3
1.7
1.2
0.6
SRR17885877_K263E_iPSC-npcs(rep3)
6.9
4.6
2.3
-0.3
17641398
17641850
17642462
17642959
chr19:17641397-17642959:+
UNC13A | UNC13A-204
UNC13A | UNC13A-206
UNC13A | UNC13A-205
UNC13A | UNC13A-202

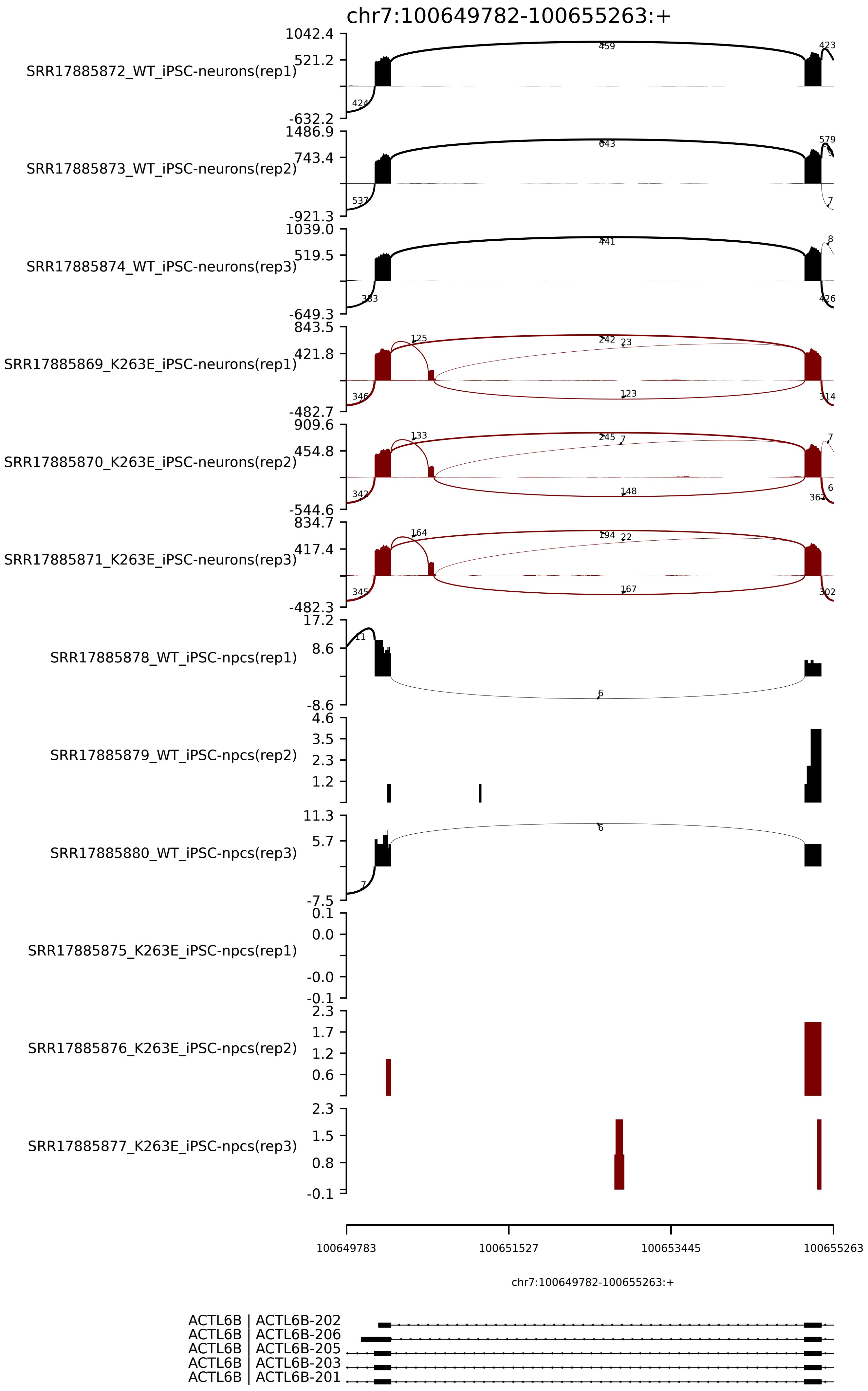

chr7:100649782-100655263:+
SRR17885872_WT_iPSC-neurons(rep1)
SRR17885873_WT_iPSC-neurons(rep2)
SRR17885874_WT_iPSC-neurons(rep3)
SRR17885869_K263E_iPSC-neurons(rep1)
SRR17885870_K263E_iPSC-neurons(rep2)
SRR17885871_K263E_iPSC-neurons(rep3)
SRR17885878_WT_iPSC-npcs(rep1)
SRR17885879_WT_iPSC-npcs(rep2)
SRR17885880_WT_iPSC-npcs(rep3)
SRR17885875_K263E_iPSC-npcs(rep1)
SRR17885876_K263E_iPSC-npcs(rep2)
SRR17885877_K263E_iPSC-npcs(rep3)
100649783
100651527
100653445
100655263
chr7:100649782-100655263:+
ACTL6B | ACTL6B-202
ACTL6B | ACTL6B-206
ACTL6B | ACTL6B-205
ACTL6B | ACTL6B-203
ACTL6B | ACTL6B-201

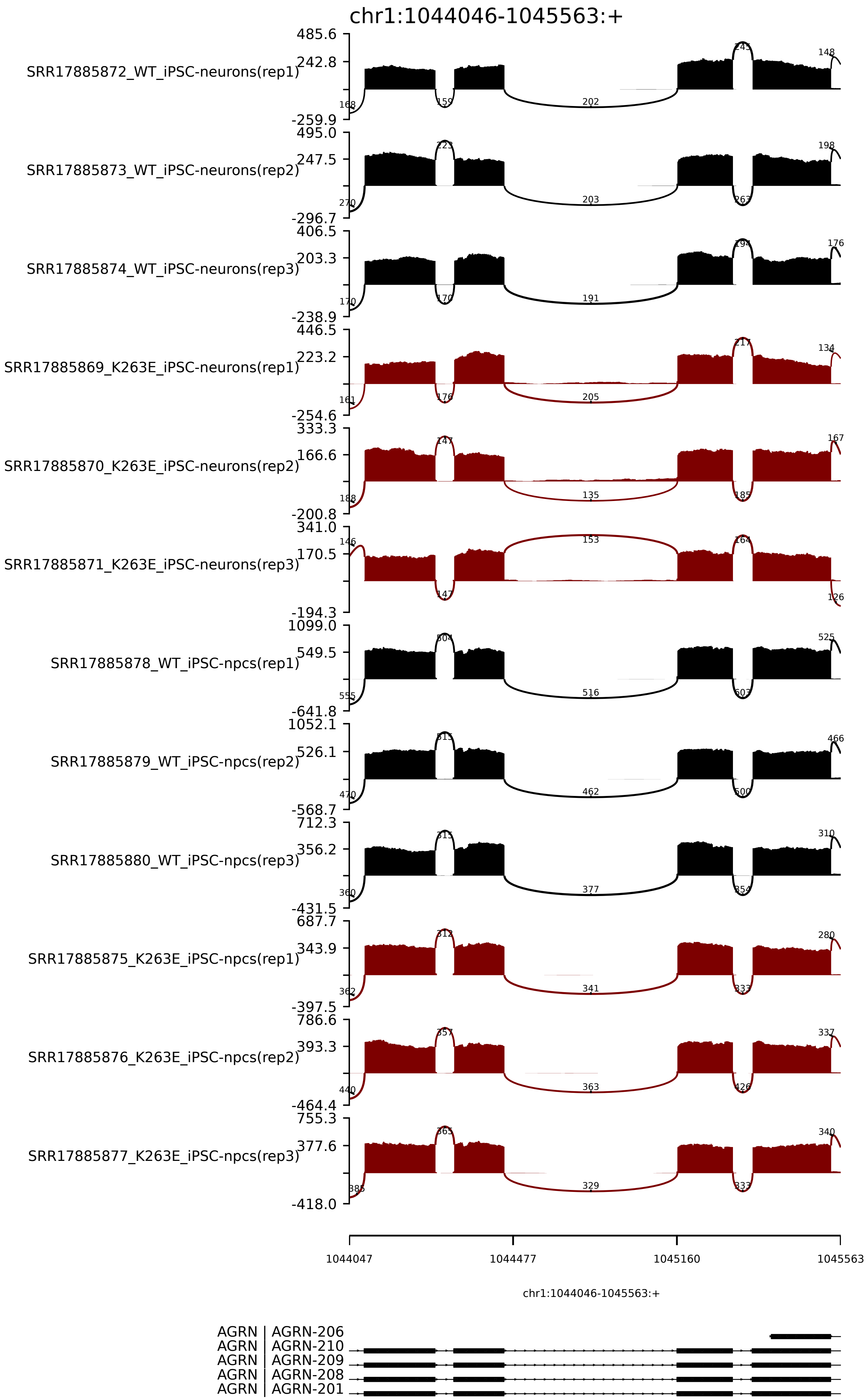
chr1:1044046-1045563:+
485.6
242.8
-259.9
SRR17885872_WT_iPSC-neurons(rep1)
243
148
168
159
202
495.0
247.5
-296.7
SRR17885873_WT_iPSC-neurons(rep2)
223
198
270
203
263
406.5
203.3
-238.9
SRR17885874_WT_iPSC-neurons(rep3)
194
176
170
170
191
446.5
223.2
-254.6
SRR17885869_K263E_iPSC-neurons(rep1)
217
134
161
176
205
333.3
166.6
-200.8
SRR17885870_K263E_iPSC-neurons(rep2)
147
167
188
135
185
341.0
170.5
-194.3
SRR17885871_K263E_iPSC-neurons(rep3)
146
153
164
147
126
1099.0
549.5
-641.8
SRR17885878_WT_iPSC-npcs(rep1)
504
525
555
516
503
1052.1
526.1
-568.7
SRR17885879_WT_iPSC-npcs(rep2)
515
466
470
462
500
712.3
356.2
-431.5
SRR17885880_WT_iPSC-npcs(rep3)
315
310
360
377
354
687.7
343.9
-397.5
SRR17885875_K263E_iPSC-npcs(rep1)
312
280
362
341
333
786.6
393.3
-464.4
SRR17885876_K263E_iPSC-npcs(rep2)
357
337
440
363
426
755.3
377.6
-418.0
SRR17885877_K263E_iPSC-npcs(rep3)
365
340
385
329
333
1044047
1044477
1045160
1045563
chr1:1044046-1045563:+
AGRN | AGRN-206
AGRN | AGRN-210
AGRN | AGRN-209
AGRN | AGRN-208
AGRN | AGRN-201

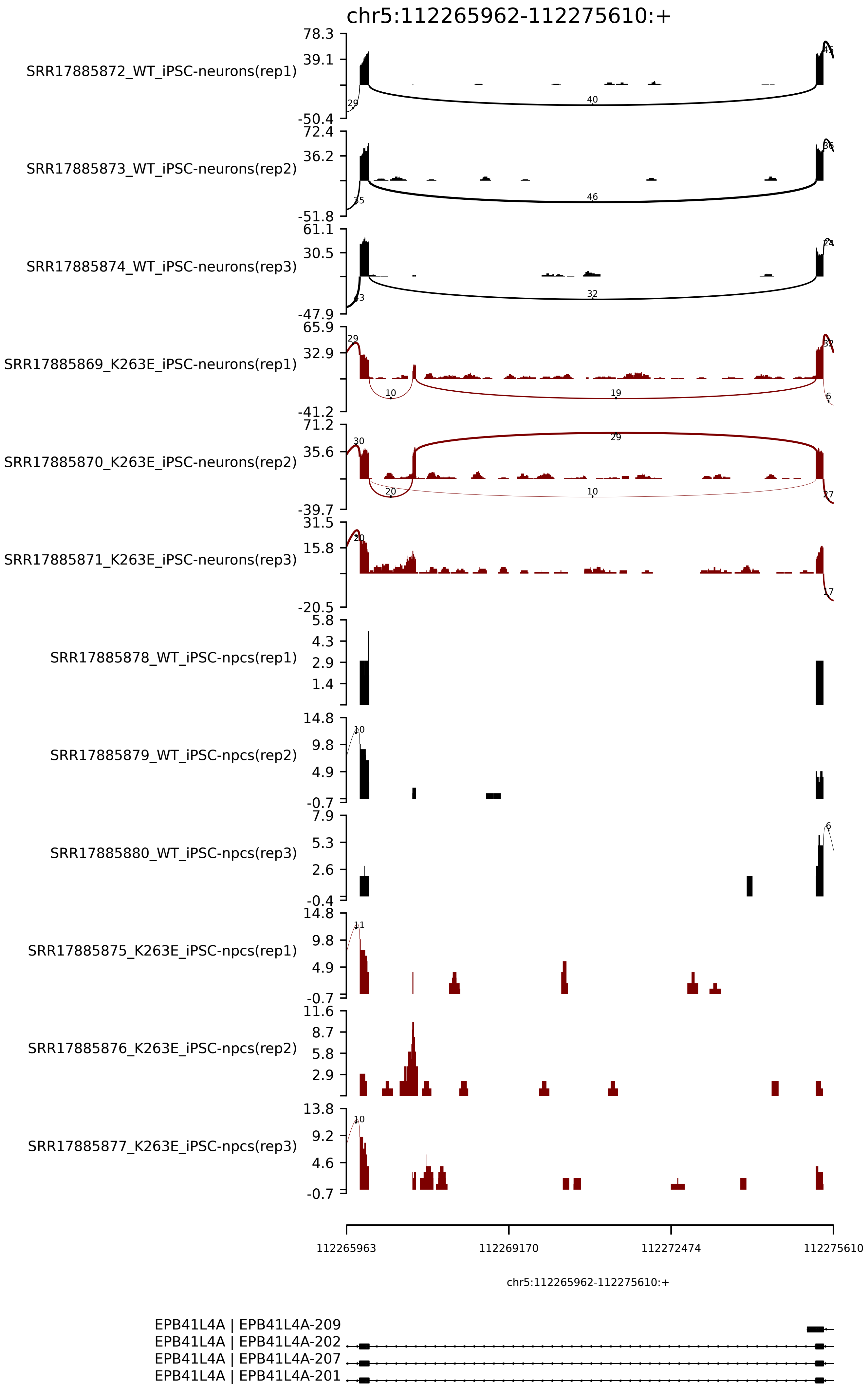

chr5:112265962-112275610:+
SRR17885872_WT_iPSC-neurons(rep1)
SRR17885873_WT_iPSC-neurons(rep2)
SRR17885874_WT_iPSC-neurons(rep3)
SRR17885869_K263E_iPSC-neurons(rep1)
SRR17885870_K263E_iPSC-neurons(rep2)
SRR17885871_K263E_iPSC-neurons(rep3)
SRR17885878_WT_iPSC-npcs(rep1)
SRR17885879_WT_iPSC-npcs(rep2)
SRR17885880_WT_iPSC-npcs(rep3)
SRR17885875_K263E_iPSC-npcs(rep1)
SRR17885876_K263E_iPSC-npcs(rep2)
SRR17885877_K263E_iPSC-npcs(rep3)
112265963
112269170
112272474
112275610
chr5:112265962-112275610:+
EPB41L4A | EPB41L4A-209
EPB41L4A | EPB41L4A-202
EPB41L4A | EPB41L4A-207
EPB41L4A | EPB41L4A-201

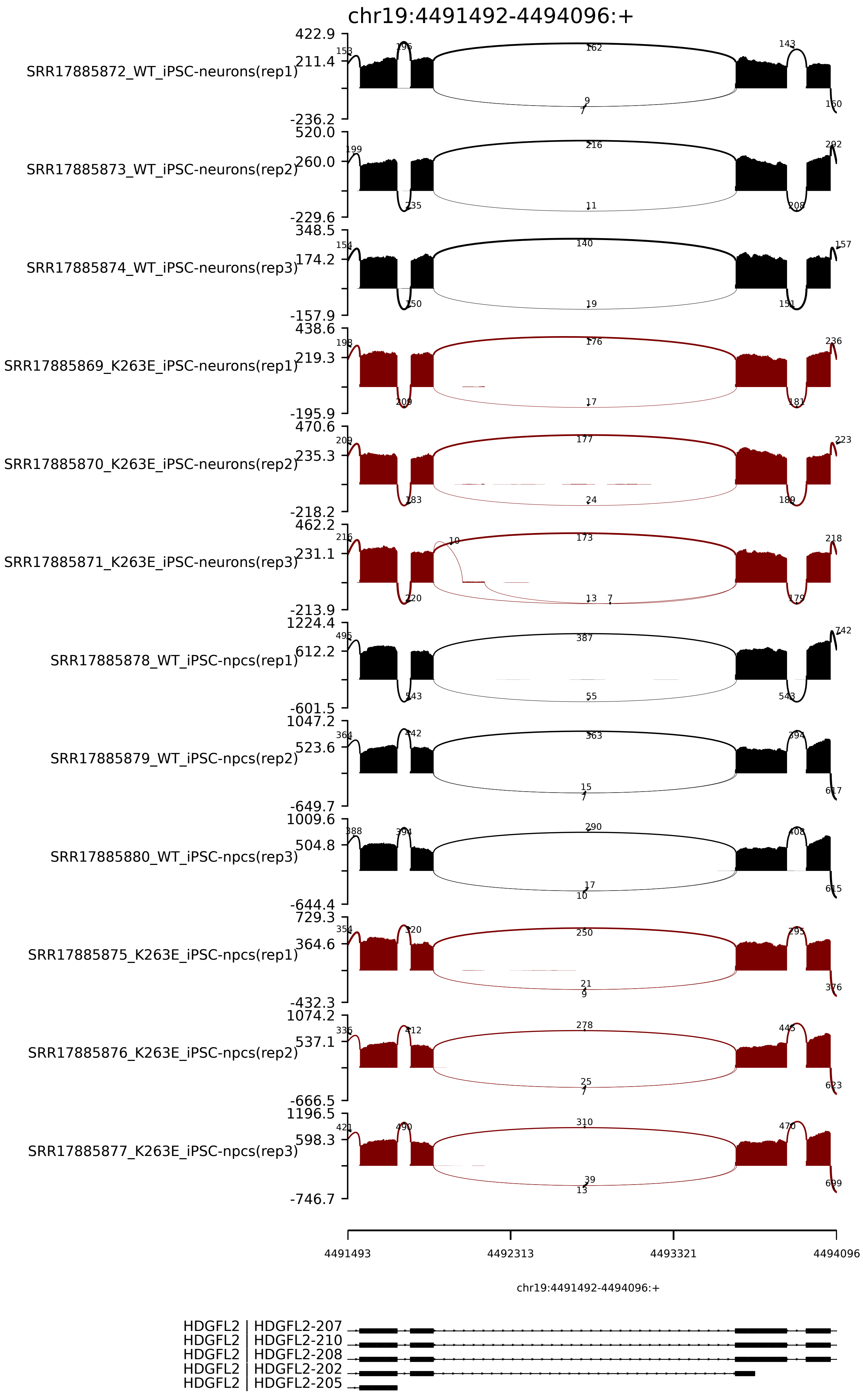
chr19:4491492-4494096:+
SRR17885872_WT_iPSC-neurons(rep1)
422.9
211.4
-236.2
SRR17885873_WT_iPSC-neurons(rep2)
520.0
260.0
-229.6
SRR17885874_WT_iPSC-neurons(rep3)
348.5
174.2
-157.9
SRR17885869_K263E_iPSC-neurons(rep1)
438.6
219.3
-195.9
SRR17885870_K263E_iPSC-neurons(rep2)
470.6
235.3
-218.2
SRR17885871_K263E_iPSC-neurons(rep3)
462.2
231.1
-213.9
SRR17885878_WT_iPSC-npcs(rep1)
1224.4
612.2
-601.5
SRR17885879_WT_iPSC-npcs(rep2)
1047.2
523.6
-649.7
SRR17885880_WT_iPSC-npcs(rep3)
1009.6
504.8
-644.4
SRR17885875_K263E_iPSC-npcs(rep1)
729.3
364.6
-432.3
SRR17885876_K263E_iPSC-npcs(rep2)
1074.2
537.1
-666.5
SRR17885877_K263E_iPSC-npcs(rep3)
1196.5
598.3
-746.7
4491493
4492313
4493321
4494096
chr19:4491492-4494096:+
HDGFL2 | HDGFL2-207
HDGFL2 | HDGFL2-210
HDGFL2 | HDGFL2-208
HDGFL2 | HDGFL2-202
HDGFL2 | HDGFL2-205

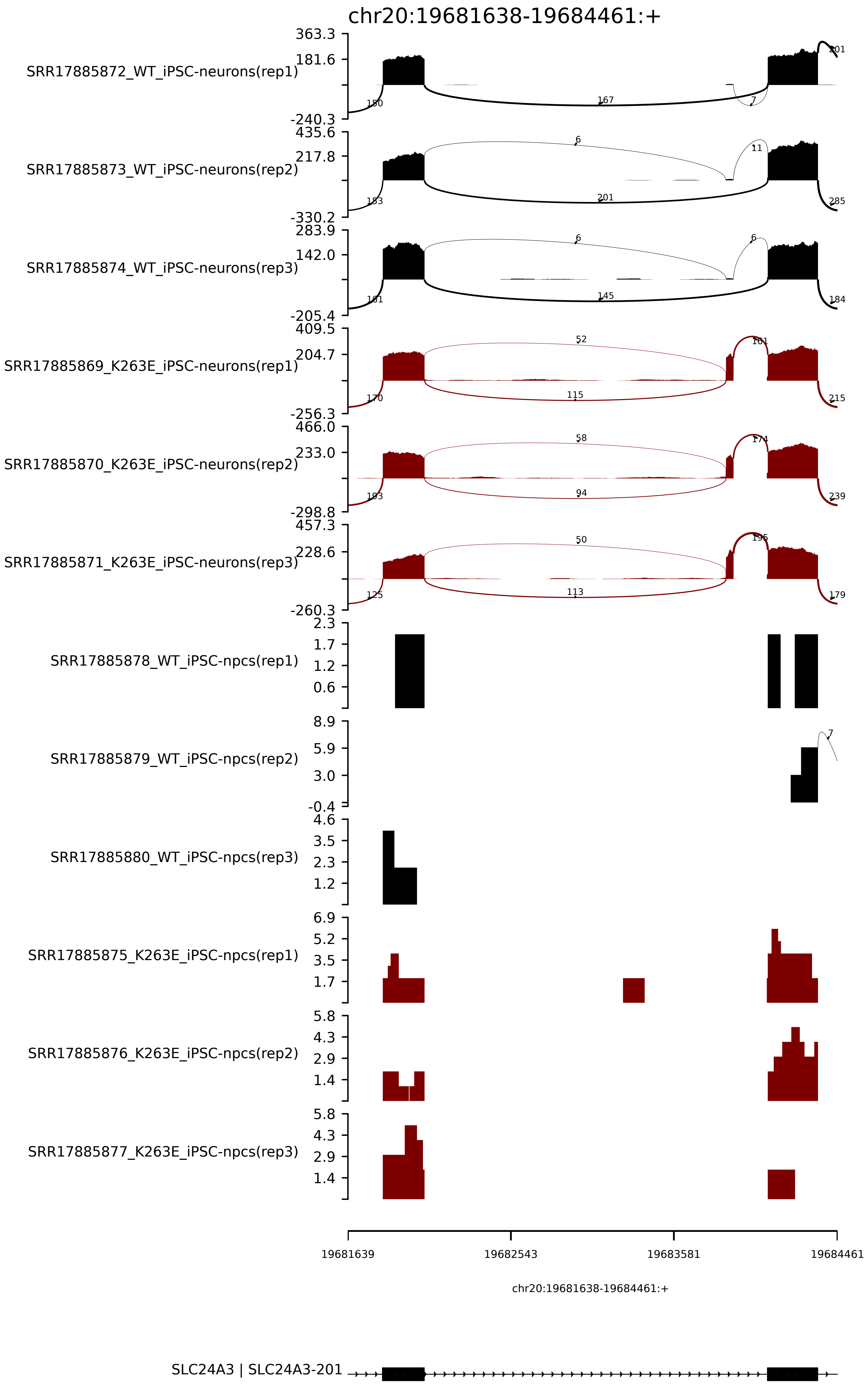
chr20:19681638-19684461:+
SRR17885872_WT_iPSC-neurons(rep1)
SRR17885873_WT_iPSC-neurons(rep2)
SRR17885874_WT_iPSC-neurons(rep3)
SRR17885869_K263E_iPSC-neurons(rep1)
SRR17885870_K263E_iPSC-neurons(rep2)
SRR17885871_K263E_iPSC-neurons(rep3)
SRR17885878_WT_iPSC-npcs(rep1)
SRR17885879_WT_iPSC-npcs(rep2)
SRR17885880_WT_iPSC-npcs(rep3)
SRR17885875_K263E_iPSC-npcs(rep1)
SRR17885876_K263E_iPSC-npcs(rep2)
SRR17885877_K263E_iPSC-npcs(rep3)
19681639
19682543
19683581
19684461
chr20:19681638-19684461:+
SLC24A3 | SLC24A3-201

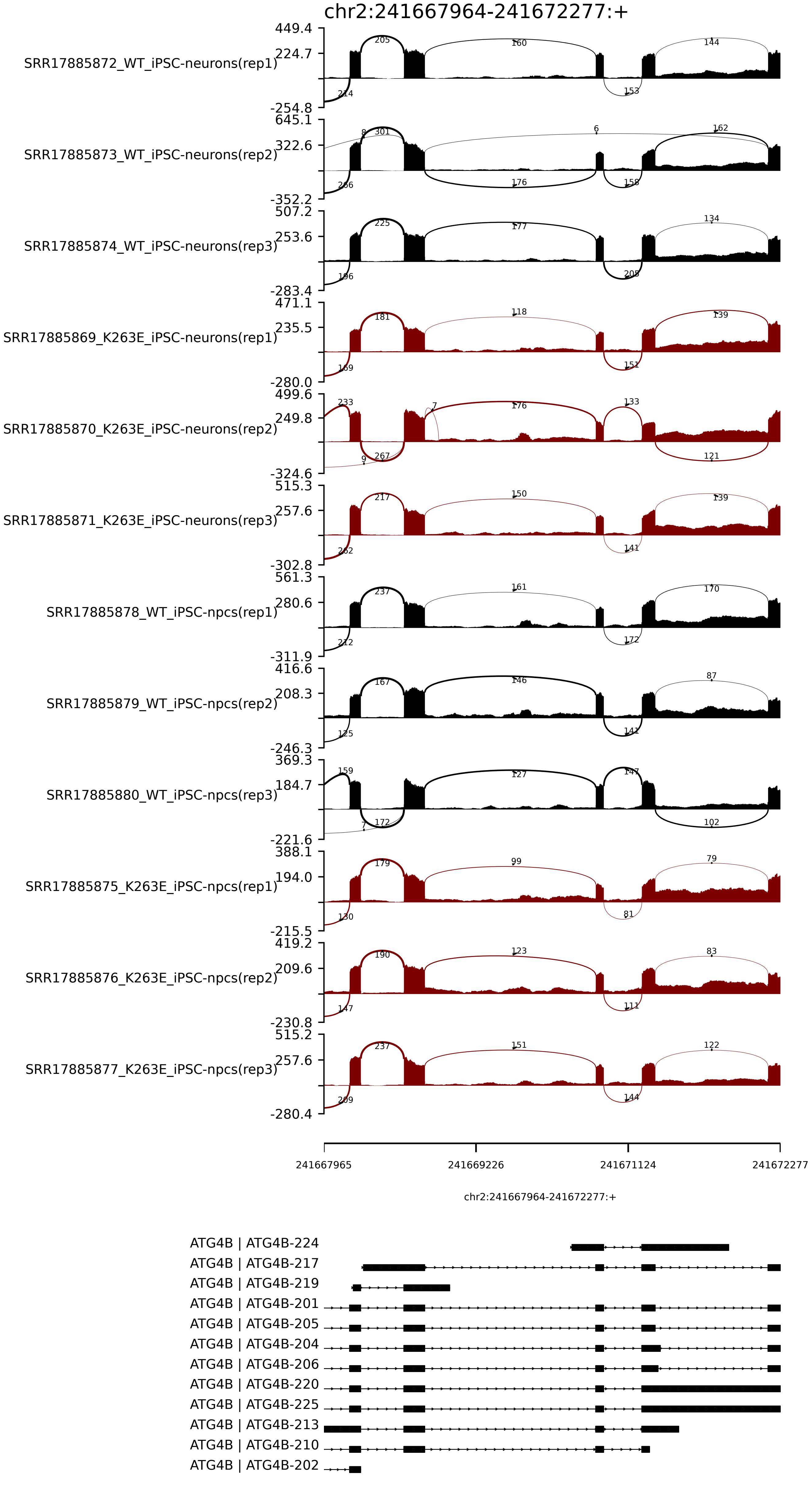

chr2:241667964-241672277:+
SRR17885872_WT_iPSC-neurons(rep1)
SRR17885873_WT_iPSC-neurons(rep2)
SRR17885874_WT_iPSC-neurons(rep3)
SRR17885869_K263E_iPSC-neurons(rep1)
SRR17885870_K263E_iPSC-neurons(rep2)
SRR17885871_K263E_iPSC-neurons(rep3)
SRR17885878_WT_iPSC-npcs(rep1)
SRR17885879_WT_iPSC-npcs(rep2)
SRR17885880_WT_iPSC-npcs(rep3)
SRR17885875_K263E_iPSC-npcs(rep1)
SRR17885876_K263E_iPSC-npcs(rep2)
SRR17885877_K263E_iPSC-npcs(rep3)
241667965
241669226
241671124
241672277
chr2:241667964-241672277:+
ATG4B | ATG4B-224
ATG4B | ATG4B-217
ATG4B | ATG4B-219
ATG4B | ATG4B-201
ATG4B | ATG4B-205
ATG4B | ATG4B-204
ATG4B | ATG4B-206
ATG4B | ATG4B-220
ATG4B | ATG4B-225
ATG4B | ATG4B-213
ATG4B | ATG4B-210
ATG4B | ATG4B-202

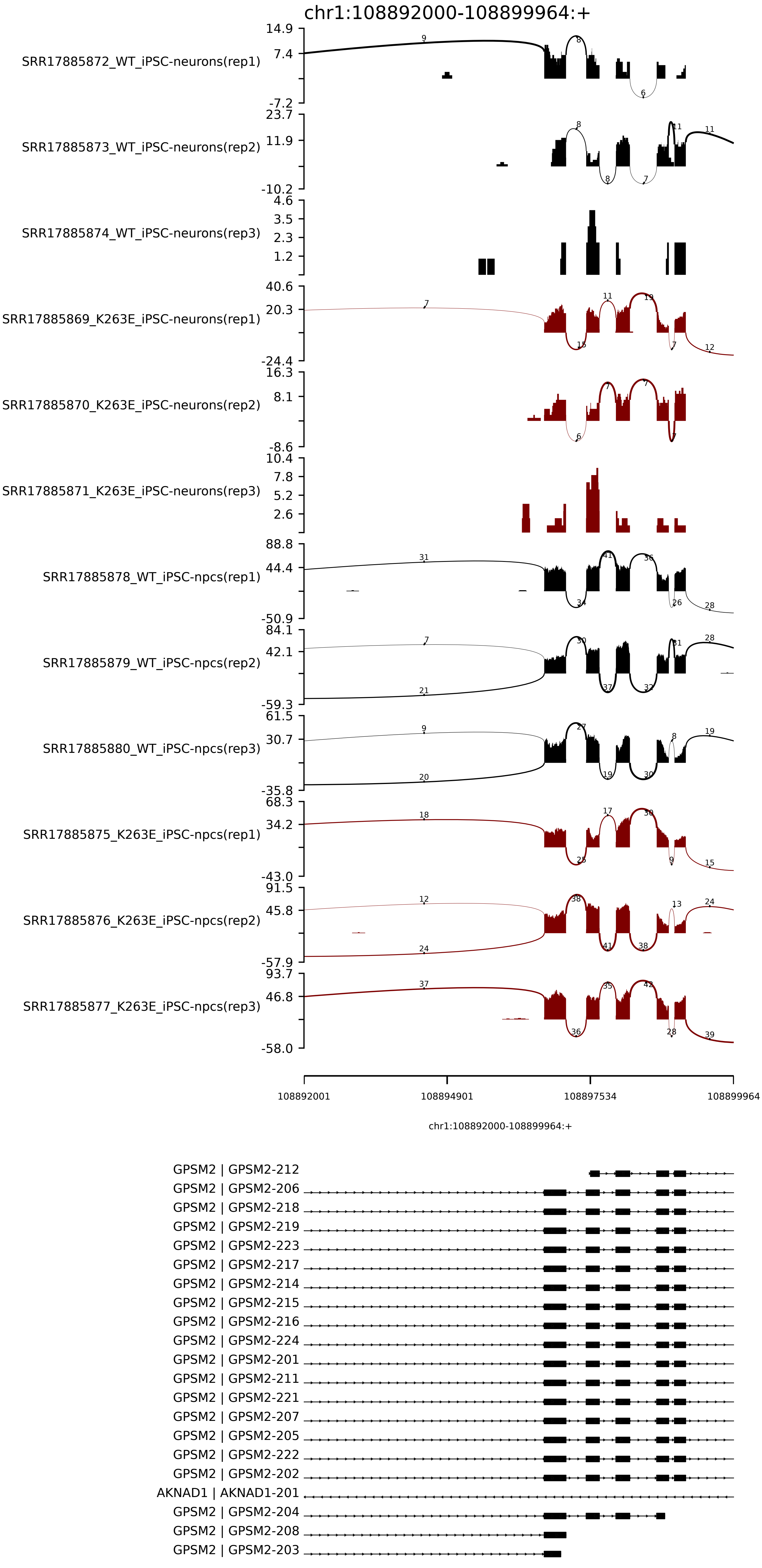
chr1:108892000-108899964:+
SRR17885872_WT_iPSC-neurons(rep1)
14.9
7.4
-7.2
SRR17885873_WT_iPSC-neurons(rep2)
23.7
11.9
-10.2
SRR17885874_WT_iPSC-neurons(rep3)
4.6
3.5
2.3
1.2
SRR17885869_K263E_iPSC-neurons(rep1)
40.6
20.3
-24.4
SRR17885870_K263E_iPSC-neurons(rep2)
16.3
8.1
-8.6
SRR17885871_K263E_iPSC-neurons(rep3)
10.4
7.8
5.2
2.6
SRR17885878_WT_iPSC-npcs(rep1)
88.8
44.4
-50.9
SRR17885879_WT_iPSC-npcs(rep2)
84.1
42.1
-59.3
SRR17885880_WT_iPSC-npcs(rep3)
61.5
30.7
-35.8
SRR17885875_K263E_iPSC-npcs(rep1)
68.3
34.2
-43.0
SRR17885876_K263E_iPSC-npcs(rep2)
91.5
45.8
-57.9
SRR17885877_K263E_iPSC-npcs(rep3)
93.7
46.8
-58.0
108892001
108894901
108897534
108899964
chr1:108892000-108899964:+
GPSM2 | GPSM2-212
GPSM2 | GPSM2-206
GPSM2 | GPSM2-218
GPSM2 | GPSM2-219
GPSM2 | GPSM2-223
GPSM2 | GPSM2-217
GPSM2 | GPSM2-214
GPSM2 | GPSM2-215
GPSM2 | GPSM2-216
GPSM2 | GPSM2-224
GPSM2 | GPSM2-201
GPSM2 | GPSM2-211
GPSM2 | GPSM2-221
GPSM2 | GPSM2-207
GPSM2 | GPSM2-205
GPSM2 | GPSM2-222
GPSM2 | GPSM2-202
AKNAD1 | AKNAD1-201
GPSM2 | GPSM2-204
GPSM2 | GPSM2-208
GPSM2 | GPSM2-203

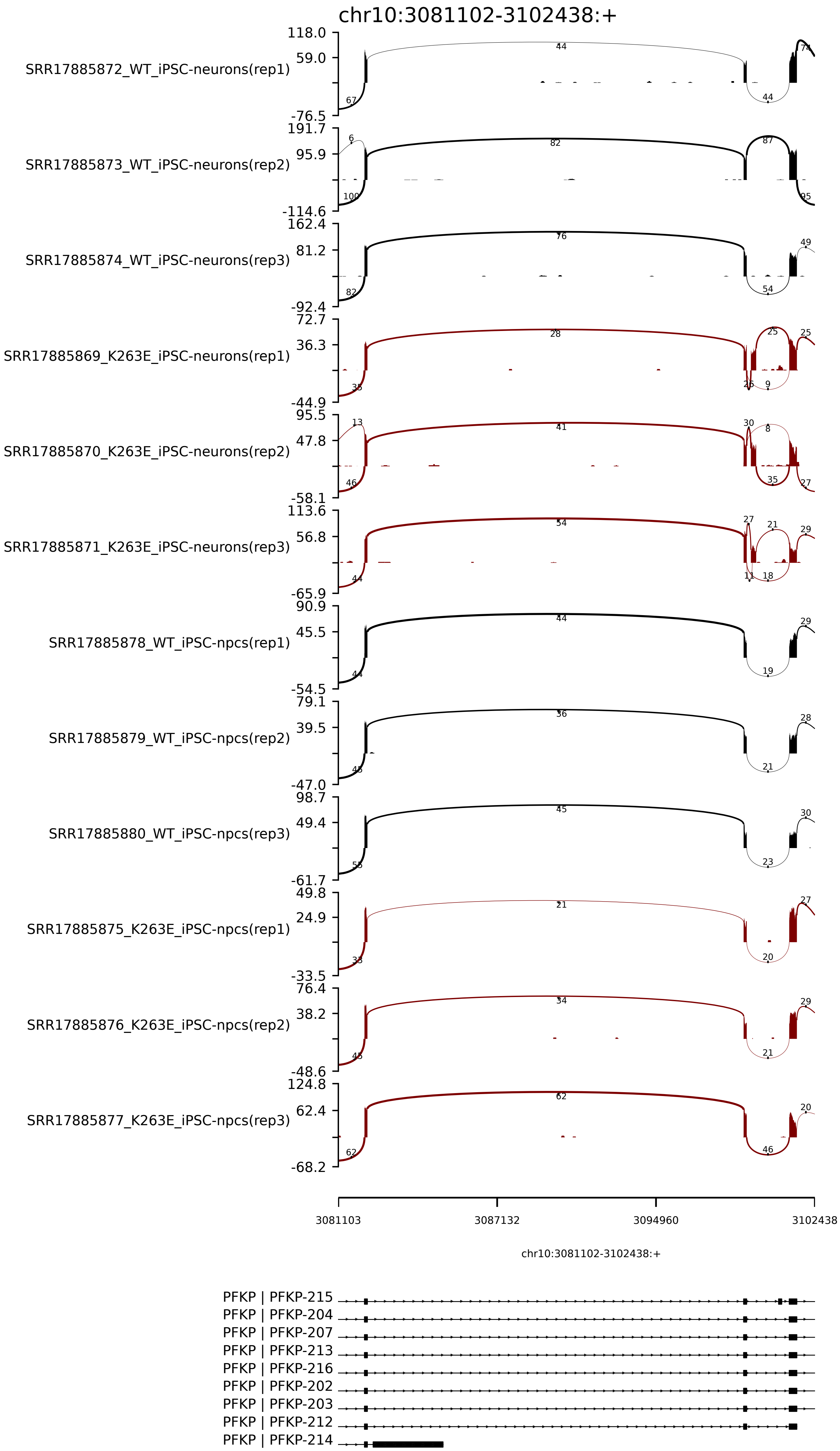
chr10:3081102-3102438:+
SRR17885872_WT_iPSC-neurons(rep1)
118.0
59.0
-76.5
SRR17885873_WT_iPSC-neurons(rep2)
191.7
95.9
-114.6
SRR17885874_WT_iPSC-neurons(rep3)
162.4
81.2
-92.4
SRR17885869_K263E_iPSC-neurons(rep1)
72.7
36.3
-44.9
SRR17885870_K263E_iPSC-neurons(rep2)
95.5
47.8
-58.1
SRR17885871_K263E_iPSC-neurons(rep3)
113.6
56.8
-65.9
SRR17885878_WT_iPSC-npcs(rep1)
90.9
45.5
-54.5
SRR17885879_WT_iPSC-npcs(rep2)
79.1
39.5
-47.0
SRR17885880_WT_iPSC-npcs(rep3)
98.7
49.4
-61.7
SRR17885875_K263E_iPSC-npcs(rep1)
49.8
24.9
-33.5
SRR17885876_K263E_iPSC-npcs(rep2)
76.4
38.2
-48.6
SRR17885877_K263E_iPSC-npcs(rep3)
124.8
62.4
-68.2
3081103
3087132
3094960
3102438
chr10:3081102-3102438:+
PFKP | PFKP-215
PFKP | PFKP-204
PFKP | PFKP-207
PFKP | PFKP-213
PFKP | PFKP-216
PFKP | PFKP-202
PFKP | PFKP-203
PFKP | PFKP-212
PFKP | PFKP-214
